# Structure-guided engineering of immunotherapies targeting TRBC1 and TRBC2 in T cell malignancies

**DOI:** 10.1038/s41467-024-45854-3

**Published:** 2024-02-21

**Authors:** Mathieu Ferrari, Matteo Righi, Vania Baldan, Patrycja Wawrzyniecka, Anna Bulek, Alexander Kinna, Biao Ma, Reyisa Bughda, Zulaikha Akbar, Saket Srivastava, Isaac Gannon, Mathew Robson, James Sillibourne, Ram Jha, Mohamed El-Kholy, Oliver Muhammad Amin, Evangelia Kokalaki, Mohammed Amin Banani, Rehan Hussain, William Day, Wen Chean Lim, Priyanka Ghongane, Jade R. Hopkins, Dennis Jungherz, Marco Herling, Martin Welin, Sachin Surade, Michael Dyson, John McCafferty, Derek Logan, Shaun Cordoba, Simon Thomas, Andrew Sewell, Paul Maciocia, Shimobi Onuoha, Martin Pule

**Affiliations:** 1 Autolus Therapeutics, London, UK; 2 Cardiff University School of Medicine; Heath Park, Cardiff, UK; 3 Department of Hematology, Cell Therapy, Hemostaseology, and Infectious Diseases, University of Leipzig Medical Centre, Leipzig, Germany; 4 Saromics Inc., Lund, Sweden; 5 Iontas Ltd., Pampisford, Cambridge, UK; 6 Cancer Institute; University College London, London, UK

**Keywords:** Cancer immunotherapy, Antibody therapy, T-cell lymphoma, T-cell receptor

## Abstract

Peripheral T cell lymphomas are typically aggressive with a poor prognosis. Unlike other hematologic malignancies, the lack of target antigens to discriminate healthy from malignant cells limits the efficacy of immunotherapeutic approaches. The T cell receptor expresses one of two highly homologous chains [T cell receptor β-chain constant (TRBC) domains 1 and 2] in a mutually exclusive manner, making it a promising target. Here we demonstrate specificity redirection by rational design using structure-guided computational biology to generate a TRBC2-specific antibody (KFN), complementing the antibody previously described by our laboratory with unique TRBC1 specificity (Jovi-1) in targeting broader spectrum of T cell malignancies clonally expressing either of the two chains. This permits generation of paired reagents (chimeric antigen receptor-T cells) specific for TRBC1 and TRBC2, with preclinical evidence to support their efficacy in T cell malignancies.

## Abstract (web summary)

The T cell receptor β-chain is expressed in two isoforms, TRBC1 and TRBC2, with clonally expanded mature T cell lymphomas expressing one of them exclusively, while healthy T cells randomly express either TRBC1 or TRBC2. Here authors show structure-based design of a TRBC2-specific antibody, and depletion of malignant T cells carrying TRBC1 or TRBC2 with CAR-T cells against the cognate receptor chain in murine models.

## Introduction

Mature T cell lymphomas represent 10–15% of non-Hodgkin lymphomas^1^ and generally have aggressive clinical features and a poor prognosis^2,3^. Unlike in B cell lymphomas, where pan B cell targeting and subsequent aplasia is clinically manageable^4^, an analogous approach in T cell malignancies is prohibitively toxic since depletion of the entire normal T cell compartment results in profound immunosuppression. Consequently, antibody-based therapeutic approaches have not been widely applied to T cell malignancies.

The T cell receptor (TCR) is expressed by the majority of mature T cell lymphomas (and ~30% of T cell acute lymphoblastic leukemias)^5,6^. The TCR comprises a heterodimeric protein complex of two chains, TCRα and TCRβ^7^. An ancestral duplication of the β-chain constant gene results in the expression of one of two highly homologous chains [T cell receptor β-chain constant (TRBC) domains 1 and 2] in a mutually exclusive manner following TCR locus rearrangement^7,8^. We previously described the development of a chimeric antigen receptor (CAR)-T cell product based on the anti-TRBC1 antibody, Jovi-1^9^, which is undergoing clinical evaluation in a Phase I/II trial (NCT03590574). This strategy allows the selective targeting and depletion of T cells carrying the TRBC1 chain, both healthy and malignant, while sparing healthy T cells expressing TRBC2: thereby preserving T cell-mediated immune responses.

**Fig. 1:**
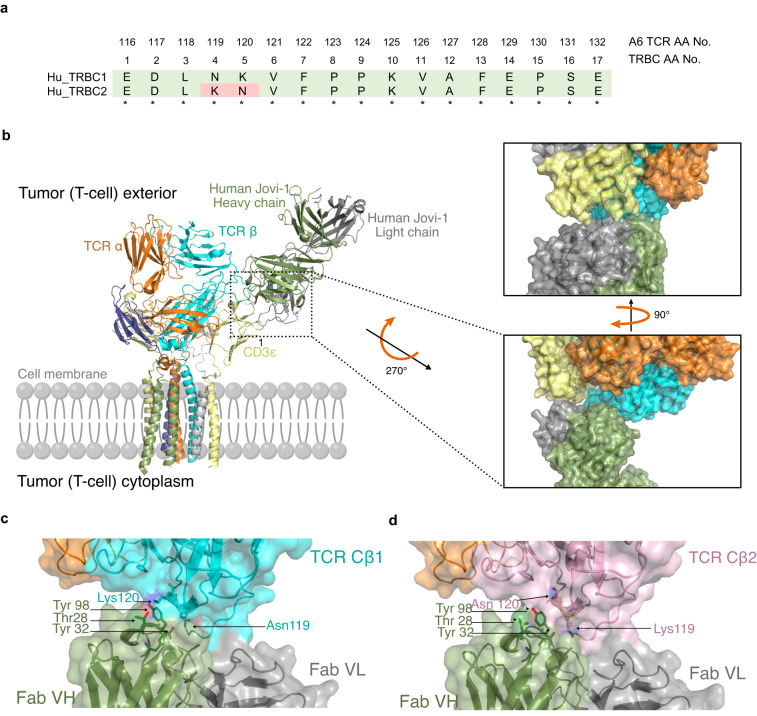
Structural modeling and interaction between TCR-targeting antibodies and TRBC1/2. **a** Homology between human TRBC1 and TRBC2. Numbering according to constant region of proteins (TRBC1, UniProtKB - P01850 and TRBC2, UniProtKB - A0A5B9) Corresponding positions of amino acids from the β chain of the A6 TCR used in this study are also shown. **b** Superimposition of HuJovi-1 Fab-TCR complex on TCR CD3 complex structure showing how specificity for TRBC is mediated in the context of the CD3 sheath. Right: Close-up of the interface between TCR complex and HuJovi-1 Fab. The Fab heavy and light chains are shown in green and gray, respectively. Despite proximity to CD3ε (yellow), neither the heavy nor the light chains form appropriate shape complementarity to interact with the CD3ε subunit of the TCR complex. **c** Molecular interface of the interaction between HuJovi-1 and TRBC1 (PDB ID 7AMP). Three key amino acids (Thr28, Tyr32, and Tyr98) within HuJovi-1 drive the specificity for TRBC1 (all antibody aa positions are referring to Kabat numbering scheme^12^). Thr28 of HuJovi-1 mediates contact with Lys119, while Tyr32 mediates contact with Asn119. Tyr98 lies across the binding pocket and forms interactions with both Asn119 and Lys120. **d** Interaction between HuJovi-1 and TRBC2 (PDB ID 7AMQ). Inversion of Lys and Asn at positions 119 and 120 of the TCR β-chain removes the interacting partners of Thr28 and Ty32 in the antibody.

TRBC1 and TRBC2 differ by only four amino acid mutations in the extracellular domain, two of which are easily accessible to a cellular immunotherapy approach (Fig. 1a)^9^. The surrounding sequence is identical between the two isoforms and the folded structure remains largely unchanged (Supplementary Fig. 1). As Jovi-1 showed optimal TRBC1 binding specificity, we use knowledge of the epitope contact interface and binding angle for the generation of a TRBC2 binder using structure-guided computational biology. This approach is challenging from a protein engineering perspective and few reports have described a specificity switch between highly homologous targets. Combining rationally-guided mutations within the complementarity determining region (CDR) loops, we engineer a complete specificity switch, while maintaining epitope targeting and the overall complex structure. We further engineer the newly developed TRBC2 binder in a CAR format, optimizing spacer, transmembrane and signaling domains to generate the optimal aTRBC2 CAR-T cell to complement the Jovi-1 CAR.

## Results

### Humanization of Jovi-1

The previously described Jovi-1 (MuJovi-1) antibody was humanized (HuJovi-1) using in silico homology modelling and CDR grafting techniques (Supplementary Table 1). The humanized antibody showed comparable binding kinetics to the murine Jovi-1 for human TRBC1, with KD values of 1.6 and 2.6 nM for Jovi-1 and HuJovi-1, respectively (Supplementary Fig. 2 and Table 1). As expected, HuJovi-1 maintained TRBC1 specificity with no measurable binding to TRBC2. The HuJovi-1 was cloned in a second-generation CAR, showing increased cell-surface expression compared to MuJovi-1 (Supplementary Fig. 3). When tested against endogenous and engineered cell lines expressing either TRBC1 or TRBC2, or donor derived T cells (Supplementary Fig. 4), the HuJovi-1 CAR demonstrated selective killing of TRBC1^+^ target cell populations with equivalent potency to the original MuJovi-1 CAR (Supplementary Fig. 5). Similarly, the HuJovi-1 CAR showed comparable tumor control to MuJovi-1 CAR in a Jurkat TRBC1 NOD SCID gamma (NSG) mouse model (Supplementary Figs. 6 and 7).

**Table 1:** SPR kinetic affinities

Clone	Mutation	TRBC1			TRBC2			Tm (°C)
		ka (1/M)	kd (1/s)	KD (M)	ka (1/M)	kd (1/s)	KD (M)	
Jovi-1	N/A	3.57E + 05	5.73E-04	1.603E-09	1.19E + 06	1.21E + 00	1.02E-06	N/A
HuJovi-1	N/A	2.38E + 05	6.18E-04	2.57E-09	6.32E + 05	1.29E + 00	1.29E-05	70.7 ( ± 0.1)
HuJovi-1 scFv-Fc	N/A	4.04E + 05	0.002996	7.51E-09	N/A	N/A	N/A	72.2 ( ± 0.0)
Mut1	VH T28K	1.50E + 05	0.001003	6.69E-09	7.82E + 04	0.5853	7.48E-06	70.7 ( ± 0.2)
Mut2	VH T28K, Y32F	1.78E + 05	0.06892	3.87E-07	1.25E + 05	0.1248	9.99E-07	71.7 ( ± 0.1)
Mut3 (KFN)	VH T28K, Y32F, A96N	8.58E + 04	0.155	1.96E-06	8.30E + 04	0.0386	4.79E-07	74.3 ( ± 0.1)
KFN scFv-Fc	VH T28K, Y32F, A96N	N/A	N/A	N/A	1.01E + 05	0.09947	1.17E-06	77.1 ( ± 0.1)
Mut4 (RFN)	VH T28R, Y32F, A96N	8.68E + 04	0.1253	1.44E-06	7.76E + 04	0.045	5.815E-07	73.8 ( ± 0.1)
Mut5	VH Y32F	1.07E + 05	0.04574	4.26E-07	N/A	N/A	N/A	-
Mut6	VH A96N	1.43E + 05	0.001968	1.38E-08	5129	0.001943	3.788E-07	-
Mut7	VH T28K, A96N	1.43E + 05	0.005239	3.65E-08	4.20E + 05	0.1661	3.952E-07	-
Anti HEL	N/A	N/A	N/A	N/A	N/A	N/A	N/A	70.7 ( ± 0.2)

### Crystal structure of HuJovi-1–TCR complex

We determined the crystal structure of the HuJovi-1 Fab in complex with engineered C1 and C2 α-β TCRs to 2.7 and 2.4 Å resolution, respectively (Supplementary Table 2). Superimposition of TCR-bound HuJovi-1 with the recently solved TCR-CD3 complex^10^ (Fig. 1b) showed that a small difference in binding angle relative to that of the CD3 targeting antibody OKT3^11^ enabled HuJovi-1 to target the β-chain specifically without effects on the TCR complex. While OKT3 was shown to form contacts with CD3 epsilon and TRBC^10^, there was limited shape or charge complementarity in the areas of proximity between HuJovi-1 and other components of the sheath.

Mutational studies indicated that Jovi-1 binds to TRBC1 via interaction with the lysine (Lys) and asparagine (Asn) residues at positions four and five (corresponding to residues Asn119 and Lys120 on the β-chain of the A6 TCR used)^9^ (Fig. 1b). Analysis of the interface between HuJovi-1 and TRBC1 (Fig. 1c) showed that most of the interactions were mediated through residues in CDR1 [Threonine (Thr)28 and Tyrosine (Tyr)32] and CDR3 (Tyr98) of the antibody heavy chain (all antibody aa positions are referring to Kabat numbering scheme^12^). As expected, the antibody binds to the TCR β-chain via residues 119–120 (A6 TCR numbering) with additional contacts made at residues 223–228. Thr28 on the CDR1 of HuJovi-1 forms interactions with Lys120, which lies in an extended rotamer conformation. The proximity of the OG1 atom of Thr28 to the NZ atom of Lys120 on TRBC1 suggests that the two atoms form hydrogen bonds to stabilize the otherwise weak interactions. This interaction is further stabilized via additional hydrogen bonding between the OH atom of Tyr32 and the ND2 atom of Asn119. Finally, Tyr98 mediates contacts with both Asn119 and Lys120, predominantly through atoms in the backbones of the two amino acids.

The solved structure of HuJovi-1 in complex with TRBC2 further explains the antibody specificity at a molecular level (Fig. 1d). Inversion of Asn119 and Lys120 removes the hydrogen bonding potential with Thr28 on HuJovi-1, which adopts a rotated conformation relative to HuJovi-1 in complex with TRBC1. The Lys120 side chain is therefore no longer able to form a bond with Thr28 and adopts a more kinked rotamer conformation accommodated within the binding pocket. Taken together, these differences at the molecular interface are responsible for the remarkable selectivity of Jovi-1 for TRBC1.

### Structural engineering of anti-TRBC2 mAb

To understand if HuJovi-1 could be modified to generate an antibody with greater specificity to TRBC2, in silico residue scanning mutagenesis was performed on the two structures. First, scanning was performed on residues in CDR1 and CDR3 of the antibody. To account for inherent noise within the modeling system, amino acid substitutions with a delta affinity (δaff) of < −5 K^o^ (indicating a predicted increase in affinity) and a delta stability (δstab) of ≤10 K^o^ were evaluated (Supplementary Table 3).

**Fig. 2:**
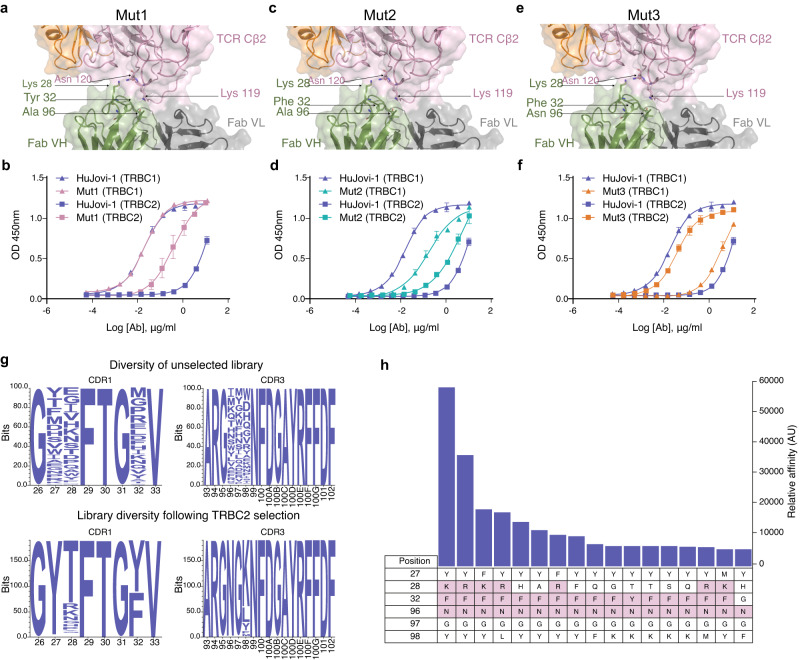
CDR mutagenesis of HuJovi-1 for TRBC2 specificity. **a** Molecular model of HuJovi-1 (Lys28 mutant; Mut1) in the context of TRBC2, suggesting that this residue can form a hydrogen bond with Lys119 in TRBC2. **b** ELISA binding of Thr28 mutant (pink) and HuJovi-1 (blue) to TRBC1 (triangles) and TRBC2 (squares). **c** Molecular model of HuJovi-1 (Lys28/Phe32; Mut2) in the context of TRBC2. **d** ELISA binding of Lys28/Phe32 Mut2 (green) and HuJovi-1 (blue) to TRBC1 (triangles) and TRBC2 (squares). **e** Molecular model of HuJovi-1 (Lys28/Phe32/Asn96; Mut3 – KFN) (PDB ID 7AMS). **f** ELISA binding of KFN (orange) and HuJovi-1 (blue) to TRBC1 (triangles) and TRBC2 (squares). **d**–**f** *n* = 3 technical replicates. Data shown as mean ± SD with non-linear fit, sigmoidal 4PL model. **g** Generation and interrogation of a phage display library based on HuJovi-1 structural analysis. Top: Amino acid distribution plot of residues in HuJovi-1 CDRs that were randomized to generate the library. Bottom: Frequency distribution of amino acids of 189 unique TRBC2-specific clones that were obtained from the library after selection. **h** Affinity ELISA showing the 17 highest affinity TRBC2 binding clones obtained after selection. (all antibody aa positions are referring to Kabat numbering scheme^12^). Source data are provided as a Source Data file.

Modelling suggested that mutations of Thr28Lys (Mut1) correlated with a strong increase in affinity in the TRBC2 model (−13 K^o^) and a reduced affinity towards TRBC1 (+3 K^o^) (Fig. 2a, b, Supplementary Table 3). Further analysis of the epitope suggested that a reduction in TRBC1 binding could be obtained through mutation of Tyr32. If the OH group within this amino acid could form a bonding pair with Asn119 in TRBC1, we hypothesized that the removal of this group would correspond to a drop in affinity towards the TRBC1 protein. As Tyr32 forms several other backbone contacts, we selected the residue phenylalanine (Phe), which differs from Tyr by the absence of a single OH group, to remove the binding potential to TRBC1 while maintaining the antibody epitope structure required for shape complementarity.

Interestingly, generation of the Thr28Lys, Tyr32Phe double mutant (Mut2) showed not only decreased affinity towards TRBC1 but an increased affinity towards TRBC2, making the antibody TRBC agnostic (Fig. 2c, d, Table 1). Finally, to engage the free Lys at position 119 of TRBC2 by amino acid substitutions within CDR3 that lay close to the binding pocket, small, targeted libraries were generated based on the Thr28Lys, Tyr32Phe mutation. Through this search, the mutation of alanine (Ala)96Asn demonstrated the highest binding towards TRBC2 with minimal binding to TRBC1. The Thr28Lys, Tyr32Phe, Ala96Asn triple mutant (Mut3) was termed KFN (Fig. 2e, f, Table 1 and Supplementary Table 1).

This rational design strategy has limitations in that the search space used is relatively small. To confirm that further modifications outside of this search space would not yield improved results, we generated several hundred protein variants with putative enhanced specificity towards TRBC2 (Supplementary Fig. 8). However no further mutations were identified that improved binding towards TRBC2 over KFN.

To avoid potential bias in computational design, a phage display library was designed to test if further mutational combinations could lead to improved binding affinity. Contact residues, in addition to residues within a 5 Å search space of the Asn and Lys motifs that form the TRBC1/2 epitope, were selected. A library of 1 × 10^8^ unique clones was obtained and interrogated against TRBC2 using TRBC1 as a negative selection antigen to drive specificity (Supplementary Fig. 9). Analysis of the library pre- and post-panning suggested a bias distribution towards certain amino acids essential for TRBC2 specificity. Interestingly, a mutation at position 96 to Asn was essential for TRBC2 binding, demonstrating the importance of this amino acid substitution. As expected, within position 32, Phe was the only accepted substitution other than Tyr. Position 28 showed a greater degree of variance, with Thr28 being predominantly returned; Lys and Arg were the second and third highest represented amino acids, respectively. Using an affinity ELISA, the antibodies with the highest differential affinity between TRBC2 and TRBC1 were the KFN and RFN (Arg28/Phe32/Asn96; Mut4) variants, with KFN displaying the strongest TRBC2 affinity and highest specificity between the two clones, further validating the rational design approach used (Fig. 2g, h and Table 1).

### High specificity of anti-TRBC2 clone KFN

**Fig. 3:**
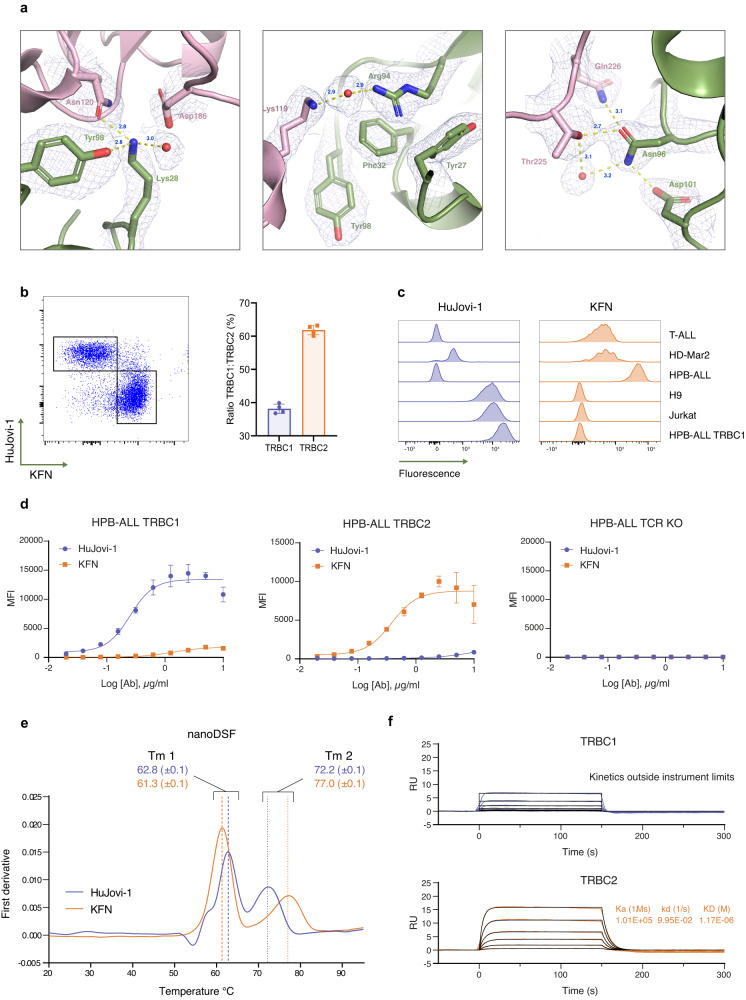
Biophysical characterization of KFN antibody compared with HuJovi-1. **a** Interface of KFN-TRBC2 from solved crystal structure (PDB ID 7AMS). The Fab heavy chain is colored in green while the TCR β-chain is in pink. The electron density is shown as a chicken wire net. Panels show the region around the Lys28 residue (left), Phe32 residue (center), and the Asn96 residue (right). **b** Left: Dot plot of a representative staining of healthy donor PBMC with HuJovi-1 and KFN IgG antibodies. Right: Distribution of TRBC1^+^ and TRBC2^+^ PBMC in four healthy donors (*n* = 4 biologically independent samples), as determined by HuJovi-1/KFN IgG staining. Data shown as mean ± SD. **c** Staining of TCR^+^ cell lines with HuJovi-1 (left) and KFN (right). **d** Staining of HPB-ALL TRBC1, HPB-ALL TRBC2, or HPB-ALL TCR KO cell lines with a titration of HuJovi-1 (blue) and KFN (orange) IgG. *n* = 4 technical replicates. Data shown as mean ± SD with non-linear fit, sigmoidal 4PL model. **e** Differential scanning calorimetry demonstrating similar stabilities of HuJovi-1 (blue) and KFN (orange) scFv-Fc antibodies. *n* = 2 technical replicates. Data shown as mean ± SD. **f** Kinetic profile of KFN scFv-Fc on recombinant TRBC1 (blue) and TRBC2 (orange). Source data are provided as a Source Data file.

To understand how the specificity of the KFN antibody was mediated, we solved its crystal structure in complex with TRBC1- and TRBC2-containing TCRs (Fig. 3a). When bound to TRBC2, the Lys28 (previously Thr) side chain nitrogen formed a 2.8 Å long hydrogen bond with OD1 of Asn120 on the TCR β-chain. Phe32 lost the hydrogen bond that the Tyr residue side chain oxygen (Oη) could make with the NZ of Lys119 on TCRβ, while hydrophobic and important spatial interactions were kept. Interestingly, the Asn96 residue gained hydrogen bond interactions through its side chain oxygen (OG1) to both the side chain oxygen (OG1) of Thr225 in the TCRβ at a distance of 2.7 Å and to the side chain amide group (Nε2) of Gln226 (3.11 Å; Fig. 3a). While these residues are present in both TRBC1 and TRBC2, it is likely that a small shift in the loop conformation in the context of TRBC1 was less favorable versus TRBC2, which accounted for the large change in binding observed with KFN to TRBC2 versus TRBC1. A separate structure of the KFN antibody in complex with TRBC1 (Supplementary Fig. 10) showed that none of the newly incorporated residues formed additional contacts in TRBC1.

The HuJovi-1 and KFN antibodies identified distinct T cell populations within peripheral blood mononuclear cells (PBMCs) from healthy donors at a ratio of 38:62 TRBC1:TRBC2 (Fig. 3b, Supplementary Fig. 11), in accordance with the expected 40:60 TRBC1:TRBC2 ratio^9^. The specificity of KFN was further tested using flow cytometry against TCR-expressing cell lines, showing binding only to those expressing TRBC2 (Fig. 3c). Dose/response binding on cells expressing equivalent TRBC1 or TRBC2 density, showed high specificity towards TRBC2-expressing cells with little binding on TRBC1-expressing cells, with the opposite observed for HuJovi-1 (Fig. 3d). While the antibody-binding specificity appeared high, the affinity measured in SPR experiments suggested a lower TRBC1:TRBC2 affinity differential than expected through ELISA and flow cytometry (Figs. 2f and 3d), with a modest 4-fold shift using a 1:1 Langmuir binding model on antibodies in (Ig)G format (Table 1). A > 100-fold affinity differential TRBC1:TRBC2 was measured when a bi-paratopic engagement was allowed, more closely aligned with observations measured by flow cytometry (Supplementary Fig. 12). It is likely that binding mediated by avidity effects increased the discriminating power, which would also likely be the case in therapeutic applications where antigen density is high and multiple CAR-antigen engagements are expected. KFN showed comparable stability and aggregation propensity as HuJovi-1 both in IgG and single-chain variable fragment-fragment crystallizable (scFv-Fc) format (Fig. 3e, Supplementary Fig. 13) and demonstrated good specificity for TRBC2 in scFv-Fc format, due to unmeasurable affinities for the non-target antigen (Fig. 3f, Table 1).

### In vitro therapeutic efficacy of TCRβ targeted CAR-T cells

**Fig. 4:**
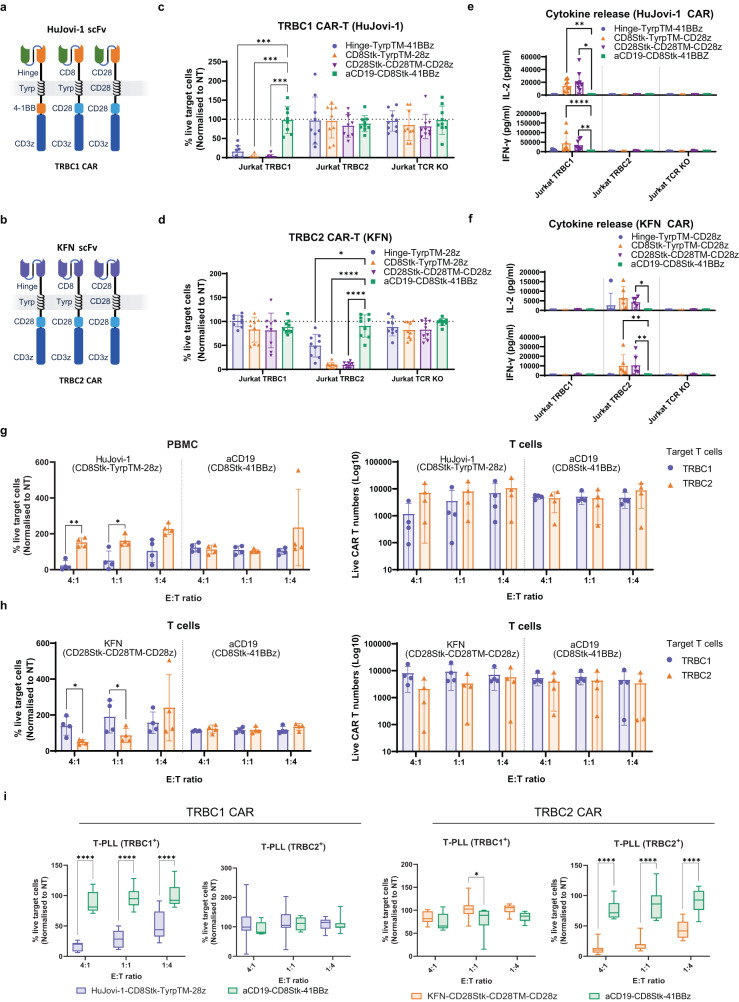
Functional characterization of HuJovi-1 and KFN CAR. Schematic of anti-TRBC1 (**a**) and anti-TRBC2 (**b**) CAR architectures. Flow cytometry-based killing of Jurkat TRBC1, Jurkat TRBC2, or Jurkat TCR KO cells by HuJovi-1 (**c**) and KFN (**d**) CAR-T cells at 1:8 E:T ratio, 72 h; donor *n* = 9 (*n* = 8 for Jurkat TRBC1 in (**c**) biologically independent samples. *p < 0.05, ***p < 0.001, ****p < 0.0001 by two-way ANOVA and Dunnett’s test for multiple comparisons versus aCD19 CAR. Data shown as mean ± SD. IFN-γ and IL-2 secretion by HuJovi-1 (**e**) and KFN (**f**) –based CARs against Jurkat TRBC1, TRBC2 and TCR KO. Donor *n* = 6 (*n* = 9 for Jurkat TRBC1) biologically independent samples for TRBC1 CAR, and *n* = 3 (*n* = 6 for Jurkat TRBC2) biologically independent samples for TRBC2 CAR. *p < 0.05, **p < 0.01, ****p < 0.0001 by two-way ANOVA and Dunnett’s test for multiple comparisons versus aCD19 CAR. Data shown as mean ± SD. Sorted healthy donor T cells (TRBC1^+^ and TRBC2^+^) forward (left) and reverse (right) killing for HuJovi-1 (**g**) and KFN (**h**) –based CARs at 4:1, 1:1 and 1:4 E:T ratios, 72 h. Donor *n* = 4 biologically independent samples, *p < 0.05, **p < 0.01, two-way ANOVA and Sidak’s post-test for multiple comparisons TRBC1 versus TRBC2. Data shown as mean ± SD. **i** FACS-based killing of primary T-PLL tumor samples (TRBC1 *n* = 3, TRBC2 *n* = 3) at 4:1, 1:1 and 1:4 E:T ratios, 72 h. PBMC donor *n* = 8 (T-PLL TRBC1^+^) and *n* = 7 (T-PLL TRBC2^+^) biologically independent samples for HuJovi-1 CAR and *n* = 8 (T-PLL TRBC1^+^) and *n* = 11 (T-PLL TRBC2^+^) biologically independent samples for KFN CAR. **p > 0.01, ***p < 0.001, ****P < 0.0001, two-way ANOVA and Sidak’s post-test for multiple comparisons versus aCD19 CAR. T-PLL = T cell prolymphocytic leukemia. Data shown as min to max box and whiskers. Source data and exact *p* values are provided as a Source Data file.

Due to the diverse kinetic profiles displayed by the KFN and HuJovi-1 scFvs, we hypothesized that different CAR architectures might be needed to convey optimal activity, given that binding kinetics may influence CAR function. KFN and HuJovi1 were tested in CARs with different spacers (IgG1 hinge, CD8 stalk and CD28 stalk), transmembrane domains (Tyrp and CD28) and endodomains (4-1BBz and CD28z) in a second-generation CAR architecture (Fig. 4a, b). An anti-CD19 CAR (CD8Stk-4-1BBz) was included as negative control.

TRBC1^+^ or TRBC2^+^ T cells isolated from PBMCs were transduced with KFN or HuJovi-1 CARs respectively. CAR expression was first determined by anti-idiotype staining. CARs showed comparable surface expression levels between HuJovi-1 and KFN CARs, with the CD28Stk-CD28TM CARs having the highest surface expression (Supplementary Figs.14 and 15).

T cells transduced with the HuJovi-1 and KFN CARs were co-cultured with cell lines that either endogenously expressed TRBC1 (Jurkat and H9) or TRBC2 (HPB-ALL, T-ALL1 and HD-MAR2), or were engineered to express TRBC1, TRBC2 or TCR KO (Jurkat and HPB-ALL). The HuJovi-1 and KFN CARs killed TRBC1 or TRBC2-expressing cells at 1:8 effector: target (E:T) ratio respectively, with limited cross-reactivity towards the non-target cell lines (Fig. 4c, d and Supplementary Figs. 14c, 15d). Among the architectures tested, the CD8Stk-TyrpTM-CD28z and CD28z-CD28TM-CD28z showed selective killing, proliferation, the highest levels of selective cytokine release (e.g., IL-2, IL-4, IL17a, IFNγ and TNFα) and selective inflammatory mediators (Fig. 4e, f and Supplementary Figs. 14c and 15d). KFN-CD28Stk-CD28TM-CD28z and HuJovi-1-CD8Stk-TyrpTM-CD28z were selected for further study given their selectivity and potency.

Reverse killing (cytolysis of CAR T cells by cognate TCR T-cells) has been reported with CAR T cells directed against the TCRγδ^13^. Hence, we next determined whether KFN or HuJovi-1 CARs were susceptible to reverse killing. KFN and HuJovi-1 CAR T cells were able to kill cognate healthy donor derived T cells (TRBC2^+^ and TRBC1^+^, respectively), showing efficient and specific killing of target cells with no indication of reverse kill even at low E:T ratio (Fig. 4g, h and Supplementary Fig. 16a). Co-culture with CAR-transduced T cells revealed transfer of antigens between target and effector cells. This exchange was accompanied by a decrease in TCR surface expression and an increase of the RQR8 marker on target T cells; and an increase in CellTrace Violet marker on HuJovi-1 and KFN CAR T cells, when co-cultured with TRBC1 or TRBC2 PBMC, respectively. This was suggestive of 2-way trogocytosis (Supplementary Fig. 17).

The CAR constructs were also tested against a number of patient-derived TRBC1 or TRBC2 T-PLL primary tumors. Both CAR-T products showed efficient and specific killing of the tumors carrying the respective TCR beta chain target (Fig. 4i and Supplementary Fig. 16b).

Analysis of the phenotypic profile for the lead architectures of HuJovi-1 and KFN CARs showed comparable levels of naïve/stem cell memory T cells (Tn), central memory (Tcm), effector memory (Tem), and effector memory cells re-expressing CD45RA (Temra). Simulation of contaminant cognate effector cells in the product showed an increase in Tem for both HuJovi-1 and KFN CAR, accompanied by a reduction in Tn and Tcm, compared to the pure product, while no significant change was detected for the aCD19 control CAR (Supplementary Fig. 18a). Similarly, high % of contaminant cells also caused an increase in the % of effector cells expressing at least one exhaustion marker (Supplementary Fig. 18b), providing further insights on the product profile for different manufacturing efficiencies.

### In vivo testing of TCRβ targeted CAR T cells

**Fig. 5:**
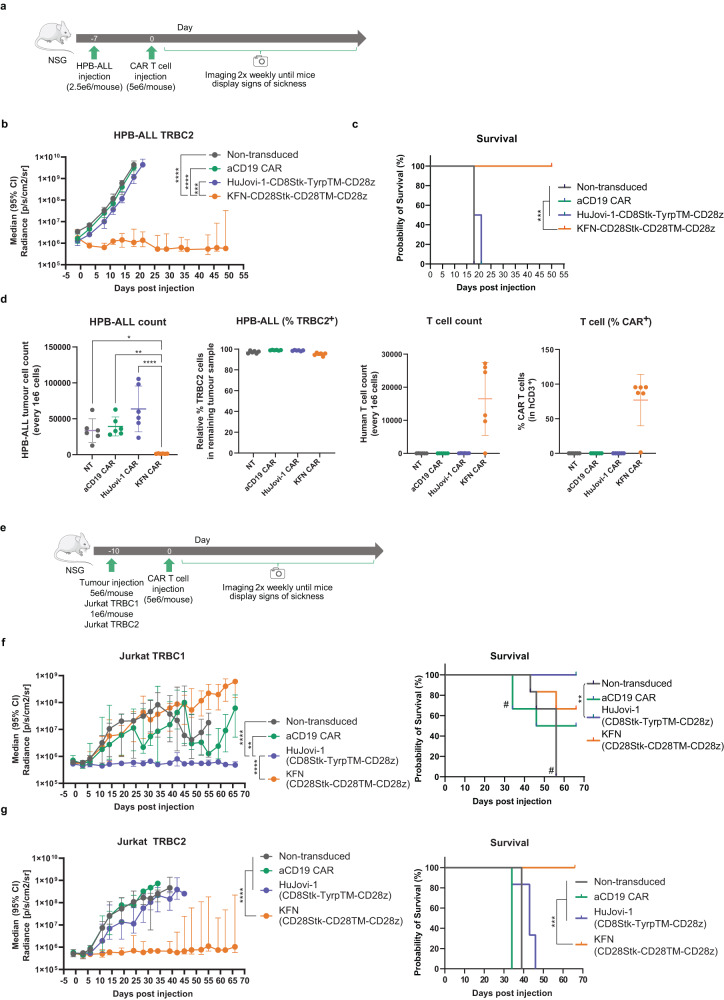
In vivo characterization of HuJovi-1 CAR and KFN CAR. **a** Schematic of NSG model with 2.5e6 HPB-ALL cells/animal (*n* = 6/group). **b** Median total flux radiance of HPB-ALL TRBC2 tumor burden (from **a**). Two-way ANOVA with Dunnett’s post test against KFN CAR, ***p < 0.001, ****p < 0.0001. Data shown as median with 95% CI. **c** Kaplan-Meier survival curve (cut-off 1e9 p/s/cm^2^/sr) (from **a**). Log-rank test ***p < 0.001. **d** (from left) HPB-ALL count in CAR treated mice; % of TRBC2 expressing cells in residual HPB-ALL population; human T cell count (CD3^+^) in treated mice; % RQR8^+^ (CAR) T cells in CD3^+^ population. One-way ANOVA with Dunnett’s post-test. *P < 0.05, **P < 0.01, ****P < 0.0001. Data shown as mean ± SD. **e** Schematic of NSG model with 5e6 Jurkat TRBC1 or 1e6 Jurkat TRBC2 cells/animal (*n* = 6/group). **f** (left) Median total radiance of Jurkat TRBC1 tumor burden. Two-way ANOVA with Dunnett’s post-test against HuJovi-1 CAR, **p < 0.01. Data shown as median with 95% CI. (right) Kaplan-Meier survival curve (cut-off 1e9 p/s/cm^2^/sr). # 1 mouse from aCD19 CAR at d34 and remaining mice from the non-transduced group at d55, sacrificed due to xenogeneic graft versus host disease. Log-rank test **p < 0.01. **g** (left) Median total radiance of Jurkat TRBC2 tumor burden. Two-way ANOVA with Dunnett’s post-test against KFN CAR, ****p < 0.0001. Data shown as median with 95% CI. (right) Kaplan-Meier survival curve (cut-off 1e9 p/s/cm^2^/sr). Log-rank test ***p < 0.001. Source data and exact *p* values are provided as a Source Data file.

To test the efficacy of CAR-T cells in mouse models of T cell malignancies, NSG mice were intravenously injected with Jurkat (TRBC1^+^ or TRBC2^+^) and HPB-ALL (TRBC2^+^) T cells, modified to express firefly luciferase (FLuc). Cells were stably engrafted in the bone marrow of all injected animals prior to administration of T cells expressing aTRBC2 CAR (KFN-CD28Stk-CD28TM-CD28z) or aTRBC1 CAR (HuJovi-1-CD8Stk-TyrpTM-CD28z). KFN CAR-T cells controlled tumor in the HPB-ALL TRBC2 and Jurkat TRBC2 models, in contrast to HuJovi-1 CAR and aCD19 CAR and non-transduced T cell controls (Fig. 5 and Supplementary Figs. 19–22). This response also translated into a survival advantage for KFN CAR-treated mice in both mouse models (Fig. 5c, g). Analysis of bone marrow (BM) tumor content revealed significantly lower tumor burden in the HPB-ALL TRBC2 for KFN CAR at day 50 (Fig. 5d), with only one animal in the HPB-ALL and Jurkat TRBC2 models showing expanding tumor cells by BLI (Supplementary Figs. 19–22). Conversely, the KFN CAR failed to control tumor growth in the Jurkat TRBC1 model (Fig. 5d–f and Supplementary Figs. 21 and 22), where instead the HuJovi-1 CAR treated mice showed consistently low tumor burden and event-free survival for the duration of the observation period (Fig. 5d–f and Supplementary Fig. 22a). In this case, the HuJovi-1 CAR cohort showed significantly lower Jurkat TRBC1 tumor burden in the BM at day 66 compared to KFN CAR cohort (Supplementary Fig. 22b). aCD19 CAR treated mice demonstrated progression of both TRBC1^+^ and TRBC2^+^ engrafted tumors.

## Discussion

Exploiting TRBC1 and TRBC2 expression on clonal T cell malignancies offers a new therapeutic approach for a group of aggressive diseases with poor survival outcomes, without causing profound T cell aplasia. Other treatment approaches for T cell malignancies that target pan-T cell antigens, such as CD4^14^, CD5^15,16^, CD7^17,18^, CD37^19^, and CCR4^20^, have been described and are in development, but could be overly immunosuppressive in a clinical setting. Further, antigen escape by lineage switch between TRBC1 and TRBC2 is an unlikely scenario especially in mature T-cell lymphomas such as PTCL and ATCL, where the malignant cells have already undergone TCRβ rearrangement and commitment to allelic exclusion^21^. We previously demonstrated the utility of a TCR-targeting approach using TRBC1-specific CAR-T cells^9^, with a clinical trial currently undergoing a phase I/II study (NCT03590574). However, over 60% of T cell lymphomas are predicted to express TRBC2. Thus, to facilitate a viable treatment strategy, TRBC2-targeting agents were sought.

Our previous mutational studies suggested that a single amino acid inversion at positions four and five of the constant β-chain provides an accessible discriminating portion between TRBC1 and TRBC2^9^. The anti-TRBC1 binder Jovi-1 was originally obtained via immunization of human TCRβ transgenic mice^22^. We hypothesized that it would be possible to redirect the specificity of Jovi-1 from TRBC1 to TRBC2 by modifying the antibody paratope.

The generation of antibodies with exquisite specificities has been well documented. Of note are the site-directed mutagenesis and phage display approaches utilized to convert the specificity of small molecule-targeting antibodies^23–25^. In these examples, however, the interaction with a small molecule only involved a limited number of contact point possibilities, facilitating the specificity redirection by paratope mutagenesis. The binding region of Jovi-1 for TRBC1 forms instead a conformational epitope on a heterodimeric protein that is expressed on the cell surface within a large complex. On such structures there is a greater chance of incurring contact events outside the target epitope with consequent cross-reactivity^26^.

We sought to generate specificity to TRBC2 by an understanding of the antibody-antigen complex of the highly related protein TRBC1. We solved the crystal structure of HuJovi-1 in complex with TRBC1 and TRBC2, confirming the TRBC1 Asn119-Lys120 involvement. With this information, we rationally designed a set of mutants of HuJovi-1 within the CDR1 and CDR3 to complement the change in orientation of the Lys/Asn side chains on TRBC2. This was validated via exhaustive phage display screening using a combinatorial library of residues within 5 Å of the TRBC2 Lys119-Asn120. The mutant, KFN, showed a 3-log decrease in TRBC1 affinity and a 15-fold increase in TRBC2 affinity compared with the parent antibody, with a 4-fold TRBC1/TRBC2 differential. The differential binding was measured at 100-fold when avidity was considered. The solved crystal structure for KFN/TRBC2 complex showed an identical binding angle compared with HuJovi-1/TRBC1, indicating that the CDR mutations and specificity switch preserved the paratope conformation that was initially matured in vivo during Jovi-1 selection.

In the context of a CAR, the identical geometry of the interaction between HuJovi-1 /KFN and TRBC1/2 should translate into a similar immunological synapse. However, we hypothesized that the differences in binding affinities between HuJovi-1 and KFN might require different CAR architecture for optimal function against cognate target antigen density. A functional exploration of different architectures showed that CD28Stk-CD28TM-CD28z was optimal for KFN, but CD8Stk-TyrpTM-CD28z was optimal for HuJovi-1. Notably, the CD28Stk-CD28TM-CD28z showed higher surface CAR density compared to the other architectures tested, which may be due to the stabilizing effect of the CD28TM domain^27^. Additionally, the CD28Stk region has been shown to reduce the target antigen density threshold for CAR-T activity^28^, possibly due to heterodimerization with endogenous CD28^29,30^. These factors may compensate for the lower affinity of KFN.

The optimized CARs showed selective cytotoxicity against the respective target cell lines, with enhanced cytokine secretion, such as IL2, IFNγ, TNFα, IL4, IL17a and cytolytic mediators, when co-cultured with TRBC1 or TRBC2 expressing cell lines for HuJovi-1 and KFN CAR, respectively. Similarly, selective cytotoxic activity was also shown for primary tumor samples carrying the respective target TCRβ chain. Notably, targeting the TCR carries potential limitation. We have recently shown that TCRγδ-directed CAR-T cells are susceptible to reverse killing by γδ T cells, manifested at low E:T ratios^13^. A similar scenario is also possible when targeting TCRαβ. However, co-culturing of aTRBC1 and aTRBC2 CAR-T cells with healthy T cells at high to low E:T ratios demonstrated efficient forward killing (CAR-T to T cells, at 4:1 and 1:1 E:T ratios) without evidence of reverse killing. Additionally, both CARs demonstrated selective killing capacity, sparing the non-cognate TCR expressing peripheral blood T cells. This difference with TCRγδ targeting may be due to more rapid T cell killing with CD28z CARs.

Unlike pan-T cell antigen targeting approaches, TRBC1/2 targeting spares depletion of the entire T cell compartment. However, fratricide, and subsequent T-cell exhaustion, is still possible^18^. To reduce this effect, we have devised a strategy to select TRBC1^+^ or TRBC2^+^ T cells for the generation of anti-TRBC2 and anti-TRBC1 CAR T cells, respectively. This strategy limits chances of cognate antigen encounter before infusion in the patient. Indeed, baseline analysis of KFN and HuJovi-1 CARs showed high levels of Tcm with low exhaustion marker expression. However, the process may still contain residual cognate antigen expressing cells, and high % of contaminant cells were associated to an increase in Tem and exhaustion markers. Further optimization, such as incorporation of dasatinib inhibition during manufacture, may improve CAR-T production^31^. Fratricide could also arise from trogocytosis, a process involving stripping of membranes from target cells by CAR T cells. As previously reported for several CAR T cell products^32–35^, trogocytosis can contribute to diminished in vivo persistence by stimulating fratricide against antigen-positive CAR T cells. Additionally, it may also facilitate tumor escape due to transient downregulation of target antigen. In vitro assessment suggested trogocytosis for both HuJovi-1 and KFN CARs, with a lower incidence for the latter CAR possibly due to a faster off-rate^33^. However, clinical relevance and impact on efficacy and long-term persistence are yet to be fully elucidated.

Finally, in vivo testing against TRBC1 and TRBC2 expressing T cell lines in NSG mice (HPB-ALL endogenous TRBC2, Jurkat endogenous TRBC1, and Jurkat engineered TRBC2) showed selective tumor growth control by HuJovi-1 CD8Stk-TyrpTM-CD28z and KFN CD28Stk-CD28TM-CD28z CAR T cells, respectively, without affecting the non-target tumor cells. KFN CD28Stk-CD28TM-CD28z CAR T cells effectively eliminated the natively TRBC2^+^ HBP-ALL and the engineered TRBC2 Jurkat lines from NSG mice with a clear survival advantage, supporting clinical development of this new receptor.

In conclusion, we have shown the structure of the interaction between humanized Jovi-1 and TRBC1 using high resolution crystallography. We confirm that recognition of the Asn/Lys (the inversion of which is the sole targetable difference with TRBC2) is responsible for TRBC1 selectivity and have determined the molecular basis for this remarkable selectivity. Further, we have used the molecular interaction analysis to rationally design the antibody paratope to flip specificity to TRBC2. This is the first description of a TRBC2 antibody, and the design strategy may act as a paradigm for future engineering of antibody specificity in the context of highly homologous epitopes. Optimized CARs based on HuJovi-1 and KFN were shown to be active against TRBC1/2 cognate cell lines in vitro and in vivo, and against primary disease samples. Development of a TRBC2 binder will now allow the possibility of targeting nearly all cases of mature T cell lymphomas, with TRBC1/2 based therapeutics opening the possibility of a widely applicable immunotherapeutic approach in this area of unmet medical need.

## Methods

### Expression and purification of recombinant T cell receptor (TCR)

Recombinant TCRαβ1 and TCRαβ2 for crystallography were expressed in *E. coli* BL21 DE3 pLysS cells and purified from inclusion bodies. Plasmids coding for TCRα, TCRβ1, and TCRβ2 chains were transformed into bacteria and expression induced via Isopropyl-β-D-thiogalactoside (IPTG) for 3 h at 37 °C. Inclusion bodies were purified by lysing cell pellets with BugBuster (Sigma, catalog no. 70584). Pellets were then washed in Triton buffer (1% Triton X100, 50 mM Tris pH 8.1, 100 mM NaCl) and resuspended in 50 mM Tris pH 8.1, 100 mM NaCl, and 10 mM ethylenediaminetetraacetic acid (EDTA). TCRα and TCRβ1 or TCRβ2 were denatured in guanidine buffer [6 M guanidine, 50 mM Tris pH 8.1, 100 mM NaCl, 10 mM EDTA, and 10 mM dithiothreitol (DTT)] and mixed at 37 °C before refolding by rapid dilution in refolding buffer (25 ml 2 M Tris pH 8.1, 42.13 g L-arginine, 150.2 g Urea, 2 ml 0.5 M EDTA, 0.37 g β-mercaptoethylamine, and 0.21 g cysteamine in 500 ml) at 60 mg/ml for 10 min. After dialysis in 10 mM Tris pH 8.1, TCRαβ complexes were purified by ion exchange (IEX) chromatography using Poros 50 HQ using an NaCl gradient on an Äkta™ pure instrument. Fractions containing the complexed TCR were further purified using size exclusion chromatography (SEC) on a HiLoad Superdex 75 16-600 column. Quality control of purified TCR was performed via SEC using a Superdex 200 10/300 GL column in phosphate-buffered saline (PBS) by loading 100 μl of sample run at 0.5 ml/min. Mammalian-expressed TCR were produced by transient transfection on ExpiCHO™ cells with plasmids carrying the α and β chains linked by a 2 A peptide. Transfection and production of supernatant was performed by Evitria AG (Switzerland). Supernatant was purified using HiTrap TALON crude (GE Healthcare, catalog no. 28-9537-67) metal affinity chromatography. A HiTrap TALON crude 5 ml column was equilibrated with 5 column volumes (CV) of running buffer (300 mM NaCl, 50 mM NaPO4, pH 7.4). Supernatant was applied to the column using an Äkta™ pure system at a flow rate of 1 ml/min. Following application of the supernatant, the column was washed with 15 CV of running buffer. The column was washed with 3 CV of elution buffer (300 mM NaCl, 50 mM NaPO4, 300 mM imidazole pH 7.4) at 4%, followed by 7 CV of 7% elution buffer. The sample was then eluted from the column with 3 CV of 100% elution buffer at 1 ml/min and collected on a 96-well plate using a fraction collector unit. Fractions containing the protein were pooled and further purified via SEC on a HiLoad Superdex 75 16-600 column in PBS pH 7.4. Fractions containing the protein at the expected molecular weight (MW; 50 kDa) were pooled and stored at −80 °C.

### Expression and purification of antibodies

Antibodies were expressed by transient transfection in ExpiCHO^TM^ cells as human immunoglobulin (Ig)G1 and single chain variable fragment-fragment crystallizable (scFv-Fc) carrying a murine IgG2a Fc domain. Supernatant from transfected ExpiCHO^TM^ cells was purified using HiTrap MabSelect SuRe (GE Healthcare, catalog no. 11-0034-93) affinity chromatography. A HiTrap MabSelect SuRe 1 ml column was equilibrated with 5 CV of PBS pH 7.4. The supernatant was applied to the column using an Akta™ pure system at a flow rate of 1 ml/min. Following application of the supernatant, the column was washed with 20 CV of PBS. The sample was then eluted from the column with 3 ml of IgG elution buffer (Pierce, catalog no. 21004) at 1 ml/min and directly loaded onto two HiTrap 5 ml desalting columns, previously equilibrated in PBS, and collected on a 96-well plate using a fraction collector unit. Fractions containing the protein were pooled and analysed via sodium dodecyl sulphate and polyacrylamide gel (SDS-PAGE). The material was divided into aliquots and stored at −80 °C. Antibodies were further characterized by SEC on a Superdex 200 increase 5/150 GL in PBS at a flow rate of 0.2 ml/min. Apparent MW was obtained by interpolation with protein standards (GE healthcare, catalog no. 28403842). For crystallography studies, HuJovi-1 and KFN IgG antibodies were converted to fragment antigen binding (Fab) fragments using a Pierce Fab preparation kit (Thermo Fisher, catalog no. 44985) according to the manufacturer’s recommendations. The Fab fragment was purified by negative selection on a MabSelect SuRe column using an Akta™ pure instrument. The Fab product was determined by SDS-PAGE to demonstrate >95% purity.

### Differential scanning fluorimetry

Purified antibodies were loaded onto glass capillaries (Nanotemper) and scanned at 330 and 350 nm using a Prometheus NT.48 instrument (Nanotemper) with a temperature ramp of 1 °C/min from 20 to 95 °C. Melting temperature (Tm) was calculated as the first derivative of the 350 nm/330 nm ratio.

### CDR grafting of Jovi-1

CDR regions of αTRBC1 Jovi-1 were defined based on the KABAT numbering system^36^ and inserted in silico into the human frameworks selected for humanized heavy and light chains (IGHV1-2*02 and IGKV2-29*02, respectively) using Bioluminate suite (Schrödinger, LLC, New York, NY,). Humanized VH and VL sequences were synthesized as G blocks™ and sub-cloned into the AbVec heavy and AbVec kappa expression vectors^37^. Heavy and light chain vectors were co-transfected into HEK293T cells (ATCC CRL-3216) to make full-length antibody. The antibody was purified as described above.

### Structural analysis of antibody TCR complexes

For the A6 TCR [T cell receptor β-chain constant domain 1 (TRBC1)] – HuJovi-1a Fab complex, 11.2 mg/ml human TCRαβ1 – Fab complex solution in buffer (1 x PBS) was used for crystallization. Initial crystals were obtained using the Stura Footprint and Macrosol screen (Molecular Dimensions) under condition H9 containing 5 mM Zn acetate, 0.1 M sodium cacodylate pH 6.5, 9% polyethylene glycol (PEG) 8000. This condition was optimized and a seed solution was prepared. The seeding solution was then used to improve the size of crystals and to obtain single crystals. By using seeding and experimentally optimized conditions, together with an additive screen (Hampton Research), three-dimensional crystals could be obtained. These crystals were further improved using larger crystallization drops (1 µl complex, 1 µl reservoir solution, 0.25 µl seed stock) using a NeXtal plate. The final crystallization condition was 0.1 M sodium cacodylate pH 6.0, 3% PEG 8000, 5 mM Zn acetate, and 4% benzamidine. The crystals used for data collection are shown in Fig. 2. A crystal was dipped in a cryogenic solution containing 0.1 M sodium cacodylate pH 6.0, 5% PEG 8000, 5 mM Zn acetate, 4% benzamidine, and 30% glycerol. Data were collected at 100 K at station I04-1, Diamond Light Source (Didcot, England) (λ = 0.9282 Å) equipped with a Pilatus 6M-F detector. The data set was collected using an exposure time of 0.04 s and an oscillation of 0.10° per image. The data were processed using the XIA2^38^ pipeline to 2.64 Å in space group C2.

Similar methods to those described above were used for structural analysis of A6 TCR (TRBC2) – HuJovi-1, A6 TCR (TRBC1) – KFN, and A6 TCR (TRBC2) – KFN. The TCR – antibody Fab complexes were prepared by mixing equimolar amounts of the TCR and Fab solution in buffer (1 x PBS). The measured concentration of the complex used for crystallization was 10.4 mg/ml, 10.8 mg/ml, and 9.3 mg/ml, respectively. Crystals were obtained using PEG 8000 (5% for HuJovi-1/TRBC2 and 9% for KFN/TRBC1 and KFN/TRBC2), 0.1 M sodium cacodylate (pH 7.4, 6.6, and 6.2, respectively), 0.005 M ZnCl2, and benzamidine HCl (4% for HuJovi-1/TRBC2 and 2% for KFN/TRBC1 and KFN/TRBC2). Crystals from the previous complex were used as seeds in the crystallization trials. For HuJovi-1/TRBC2, five different commercial screens did not yield any crystals. For KFN/TRBC2, the crystal used for data collection are shown in Fig. 3. A crystal was dipped in a cryogenic solution containing 0.1 M sodium cacodylate pH 6.2, 9% PEG 8000, 5 mM ZnCl2, 2% benzamidine, and 30% glycerol. Data for HuJovi-1/TRBC2, KFN/TRBC1 and KFN/TRBC2 were collected at 100 K at stations I04, I03, and I04, respectively, Diamond Light Source (Didcot, England) (λ = 0.9795, 0.9762, and 0.9795 Å) equipped with a Pilatus 6M-F detector. The data sets were collected using an exposure time of 0.04 s and an oscillation of 0.10° per image. For HuJovi-1/TRBC2, the data were processed using XDS^39^ and Aimless^40^ to 2.35 Å in space group C2. to 1.95 Å in space group C2. The crystal diffracts to 3.0 Å in the worst direction but to 1.85 Å in the best direction; however, the overall spherical completeness was 99.8 % to 2.3 Å. For KFN/TRBC2, the data were processed using XDS^39^ and the XIA2-dials pipeline^38^ to 2.42 Å in space group C2.

Protein modelling and *in-silico* analysis was performed using the Bioluminate suite. All complexes were pre-processed using a protocol that included addition of missing side chains (if required), calculation of hydrogen atom positions, hydrogen-bond network optimization, and energy minimization using the Protein Preparation Wizard tool^41^. PROPKA was used for the assignment of protonation states and Prot Assign was used for hydrogen bond optimization. Predicted changes in binding affinity were computed using the residue scanning functionality in BioLuminate^42^, with the antibody selected as the ligand and the TCR selected as the receptor.

### ELISA

Binding characterization was performed using high binding streptavidin plates (Pierce, catalog no. 15500) coated with 1 μg/ml of recombinant biotinylated TRBC1 or TRBC2. Plates were incubated for 1 h at room temperature (RT) and then washed three times using PBS 0.05% Tween. Primary antibody 50 μl/well was added starting at 10 μg/ml with 3-fold serial dilutions and incubated for 1 h at RT. Plates were washed three times using PBS 0.05% Tween and the secondary anti-human horse radish peroxidase conjugated antibody (Jackson ImmunoResearch) diluted 1:3000 in PBS, and 0.5% bovine serum albumin (BSA) was added. Plates were incubated at room temperature for 1 h, washed three times, and developed with 1-step TMB Ultra substrate (ThermoFisher scientific, catalog no. 34029) for 1 min before stopping with 45 μL H_2_SO_4_. Plates were read at a wavelength of 450 nm on a Varioskan instrument (ThermoFisher scientific).

### Phage display

A HuJovi-1 single chain fragment variable library was synthesized (Twist Bioscience) with randomizations at positions at 27, 28, 32, 96, 97, and 98 of the variable heavy chain. Non-redundant randomization was designed to exclude cysteine but included the original amino acid present at each position. The double stranded deoxyribonucleic acid (DNA) library was ligated into pSANG10 and transformed into electrocompetent TG1 cells. A library size of 3.2 × 10^8^ was achieved covering the theoretical diversity of the library of approximately 5 × 10^7^. A total of 92 clones were randomly selected and sequenced. All 92 sequences were found to be unique.

Phage-display selections were carried out on a combination of TRBC1 and TRBC2 TCR proteins with alternative variable regions (HA1.7 and Mart1). A total of 24 solution phase selections were carried out employing various degrees of stringency. All 24 phage-display populations were tested for their ability to bind to TRBC1- and TRBC2-containing TCRs.

For polyclonal and monoclonal phage ELISA, 20-24 clones from each of the second round of selections were chosen. Monoclonal and polyclonal phage ELISAs were performed against the following antigens: TRBC1-HA1.7-biotinylated, TRBC2-HA1.7-biotnylated, TRBC1-MART1-biotinylated, TRBC2-MART1-biotinylated, and streptavidin.

### Surface plasmon resonance

Recombinant anti-TCR antibodies in human IgG1 format were immobilized with anti-human capture kit (GE Healthcare, catalog no. BR100839) to a density of 200 RU using a Biacore T200 instrument and captured on flow cells two, three, and four on a Series S CM5 sensor chip (GE Healthcare, catalog no. 29104988). Anti-TCR antibodies in scFv-Fc format were captured on flow cells two, three, and four on a Series S Protein A chip (GE Healthcare) to a density of 50 RU using a Biacore T200 instrument. A 0.01 M HEPES pH 7.4, 0.15 M NaCl, 0.005% v/v surfactant P20 (HBS-P + ) buffer was used as a running buffer in all experimental conditions. Recombinant, purified TRBC1 and TRBC2 at known concentrations were used as the ‘analyte’ and injected over the respective flow cells with a 150-s contact time and a 300-s dissociation time (500 s for scFv-Fc antibodies) at a 30 μl/min flow rate with a constant temperature of 25 °C. In each experiment, flow cell one was unmodified and used for reference subtraction. A ‘zero concentration’ sensogram of buffer alone was used as a double reference subtraction to account for drift. Data were fit to a 1:1 Langmuir binding model. Since a capture system was used, a local maximal analytical response (Rmax) parameter was used for the data fitting in each case.

### Generation of TRC-KO cells by CRISPR/ Cas-9 Nicking Strategy

TCR negative versions of Jurkat cell lines were generated using clustered regularly interspaced short palindromic repeats (CRISPR)/Cas9 genome engineering. Guide RNAs (gRNA) were designed targeting exon 1 of the TRBC1 gene, choosing the guides with the top quality and least off-target score; gRNA 1: CUUUCCAGAGGACCUGAACA. TCR negative versions of HBP-ALL cell lines were generated using CRISPR/Cas9 genome engineering. Guide RNAs (gRNA) were designed targeting exon 1 of the TRBC2 gene, choosing the guides with the top quality and least off-target score; gRNA 1: AACACGUUUUUCAGGUCCUC. Cells were electroporated using AMAXA Nucleofector (Lonza). TCR KO was confirmed by flow cytometry.

### Flow cytometry

HPB-ALL TRBC2+ and the TRBC1+ or TCR KO HPB-ALL cells engineered from the TRBC2+ population via CRISPR/Cas9 homology-directed genome editing, were incubated (1 × 10^5^ cells/well) with the test antibody at a concentration of 10 μg/ml and 50 μl/well with a 2-fold serial dilution for 30 min at RT. Cells were washed three times in PBS to remove unbound antibodies. Protein-labeled cells were stained using anti-human IgG H + L Alexa fluor 647 conjugated antibody (Invitrogen) at 2 μg/ml dilution in PBS for 20 min at RT. After washing off unbound antibodies, cells were stained with 7AAD viability dye (Biolegend) at 1:50 dilution in PBS. Cells stained with scFv-Fc antibodies were treated with Sytox Blue viability dye at 1:1000 dilution. Stained samples were acquired using a MacsQuant10 instrument and analyzed on FlowJo software. Co-staining of human PBMCs was carried out using HuJovi-1 in human IgG1 Fc and biotinylated KFN in murine IgG2a Fc.

### Retroviral supernatant production

4.5 × 10^6^ HEK-293T cells were transiently transfected with an RD114 envelope expression plasmid (RDF, a gift from M. Collins, University College London), a Gag-pol expression plasmid (PeqPam-env, a gift from E. Vanin, Baylor College of Medicine), and the transgene of interest expressed in a retroviral (SFG) vector plasmid at a ratio of 1:1.5:1.5 (total DNA = 12.5 µg). Transfections were performed with GeneJuice® (Millipore; 70967) according to the manufacturer’s instructions and viral supernatants were harvested 48 h post transfection and stored at −80 °C.

### Calculation of functional retroviral titers

Functional viral titers of retroviral supernatant were calculated using frozen supernatant on 293 T cells, in the presence of 7 μg/mL of Polybrene (TR-1003, Sigma-Aldrich), where 1 × 10^4^ 293 T were seeded and then cultured at 37 °C, 5% CO2 for 72 h. Transduced cells were identified by measuring for RQR8 expression. Viral titers were calculated with T cells that were less than 20% transduced.

### Isolation of TRBC1^+^ and TRBC2^+^ primary T cells

Leucocyte cones of healthy donors were purchased from National Health Service Blood and Transplant (NHSBT, UK), with consent for non-clinical use. Work was performed under approval of the Human Tissue Authority (HTA license 12642). Whole blood was extracted from each cone and diluted to 50 mL with sterile PBS. PBMCs were isolated by Ficoll gradient centrifugation using SepMate 50 (85450, StemCell) and Ficoll® Paque Plus (GE17-1440-02, Merk) layering 25 mL of whole blood mixture to each SepMate 50. The cells were centrifuged at 1200 *g* for 20 min. The buffy coat was extracted and washed twice with sterile PBS. PBMCs were resuspended at 2 × 10^7^/mL in cell separation buffer (20144, StemCell) and incubated with 3 μg/2 × 10^5^ cells of biotinylated JOVI (ANC-101-030, Ancell) for 10 min. Samples were centrifuged at 400 *g* for 5 min and then washed with separation buffer before following EasySep™ Release Human Biotin Positive Selection Kit (17653, StemCell) protocol. The unbound (TRBC2^+^) fraction were harvested from the first incubation on the magnetic rack. The bound (TRBC1^+^) fraction was collected by following the protocol as stated. Isolation was confirmed via flow cytometry, staining with aCD3-PE/Cy7 (317334, Biolegend) and Streptavidin-APC (405243, Biolegend).

### Retroviral transduction of primary human T cells

Isolated TRBC1^+^ or TRBC2^+^ T cells were resuspended at 1 × 10^6^ cells/mL in R10 and stimulated with TransAct (Miltenyi Biotec; 130-111-160), 10 ng/ml IL-7 (Miltenyi Biotec; 130-095-367) and 10 ng/ml IL-15 (Miltenyi Biotec; 130-095-760). 24 h after, cells were collected, plated at a density of 1 × 10^6^ cells per well (1 mL) on retronectin-coated (Takara, T100B) 6-well plates in the presence of retroviral supernatant at an MOI of 1. Total volume was adjusted to 3 mL using R10 supplemented with 10 ng/ml IL-7 (Miltenyi Biotec; 130-095-367) and 10 ng/ml IL-15 (Miltenyi Biotec; 130-095-760). The plate were centrifuged at 1000 *g* for 40 min. 24 h post spinoculation, the T cells were harvested and re-plated in complete R10 media supplemented with 10 ng/ml IL-7 (Miltenyi Biotec; 130-095-367) and 10 ng/ml IL-15 (Miltenyi Biotec; 130-095-760). Transduction efficiency was determined on day 5 after transduction, and further experiments were commenced on days 5–9 after transduction. CAR expression was assessed by staining with aCD3-PE/Cy7 (317334, Biolegend) and QBend10 APC (FAB7227A, R&D System).

### Cell lines

HEK-293T (ATCC CRL3216), were cultured in I10 medium consisting of Iscove’s modified Dulbecco’s medium (IMDM, Gibco) supplemented with 10% fetal bovine serum (FBS, HyClone, Thermo Scientific) and 2 mM GlutaMAX (Sigma). Jurkat (ATCC TIB-152) TRBC1^+^, TRBC2^+^, KO, HPB-ALL (DSMZ ACC-483) TRBC1^+^, TRBC2^+^, KO, H9 (ATCC HTB-176), HD-MAR (DSMZ ACC-685) and T-ALL1 (DSMZ ACC-521) cell lines were cultured in R10 medium consisting of RPMI (RPMI-1640, Gibco) supplemented with 10% FBS (HyClone, Thermo Scientific) and 2 mM GlutaMAX. HEK-293T, Jurkat TRBC1^+^, H9 were obtained from the American Type Culture Collection. HD-MAR, T-ALL1 and HPB-ALL were obtained from DSMZ-German Collection of Microorganisms and Cell Cultures.

### Retroviral transduction of TCR KO cell line

TCR KO Jurkat and HPB ALL cells were collected, plated at a density of 1 × 10^6^ cells per well (1 mL) on retronectin-coated (Takara, T100B) 6-well plates in the presence of retroviral supernatant expressing TRBC2 or TRBC1 respectively. Total volume was brought to 3 mL.

The plates were centrifuged at 1000 *g* for 40 min. 24 h post spinoculation, the cells were harvested and re-challenged with fresh viral supernatant. TRBC1 or TRBC2 expression was assessed by staining with in house produced aTRBC1 (Jovi) or aTRBC2 (KFN).

### Human CAR T cells FACS based cytotoxicity assay co-culture

Mock (Non-Transduced PBMCs) and CAR-transduced T cells were co-cultured with TRBC1^+^, TRBC2^+^ or TCR KO Jurkat or HBP-ALL, TRBC1^+^H9, TRBC2^+^ HD-MAR and T-ALL1 target cells. Target cells were labeled with CellTrace™ CFSE (C34554, ThermoFisher Scientific) following manufacturer instructions. Mock and CAR-transduced T cells were labeled with CellTrace™ Violet (C34557, ThermoFisher Scientific) following manufacturer instructions. Effector and target cells were mixed to reach an E:T ratio of 1:4, 1:8, 1:16 and 1:32. 72 h after co-culture live cell data were collected via Flow cytometry using the MacsQuantX flow cytometer (Milteny). Data analysis was conducted using FlowJo v10 (Treestar, RRID:SCR_008520). Percentage of live cells was calculated relative to the number of live target cells after co-culture with non-transduced T cells. Dot plot data available as Source data file (Source Data file 2–4).

### FACS based cytotoxicity assay co-culture with autologous healthy primary T cells

Mock (Non-Transduced PBMCs) and CAR-transduced T cells were co-cultured with autologous TRBC1^+^ and TRBC2^+^ non transduced T cells. Target cells were labeled with CellTrace™ CFSE (C34554, ThermoFisher Scientific) following manufacturer’s instructions. Effector mock and CAR-transduced T cells were labeled with CellTrace™ Violet (C34557, ThermoFisher Scientific) following manufacturer’s instructions. Effector and target cells were mixed to reach an E:T ratio of 1:4, 1:1 and 4:1. 72 h after co-culture live cell data were collected via Flow cytometry using the MacsQuantX flow cytometer (Miltenyi). Data analysis was conducted using FlowJo v10 (Treestar, RRID:SCR_008520). Percentage of live cells was calculated relative to the number of live target cells after co-culture with non-transduced T cells. Dot plot data available as Source data file (Source Data file 2–4).

### FACS based cytotoxicity assay co-culture with Primary T-PLL tumor samples

Mock (Non-Transduced PBMCs) and CAR-transduced T cells were co-cultured with either TRBC1^+^ or TRBC2^+^ human primary T-PLL tumor. Human primary T-PLL tumors were obtained from patients with T-PLL at the University Hospital of Cologne. Written informed consent according to the Declaration of Helsinki was provided by all T-PLL patients. Collection and use of their samples within the prospective T-PLL registry (NCT02863692) as part of the GCLLSG (Cologne, Germany) were approved for research purposes by the local ethics committee (#11-319), work was performed under approval of the Human Tissue Authority (HTA license 12642). Prior to use, the cells were defrosted and rested for 2 h at 37 C, at 2 × 10^6^ cells/mL. Target cells were then labeled with CellTrace™ CFSE (C34554, ThermoFisher Scientific) following manufacturer’s instructions. Mock and CAR-transduced T cells were labeled with CellTrace™ Violet (C34557, ThermoFisher Scientific) following manufacturer’s instructions. Effector and target cells were mixed to reach an E:T ratio of 1:4, 1:1 and 4:1. 72 h after co-culture live cell data were collected via flow cytometry using the MacsQuantX flow cytometer (Milteny). Data analysis was conducted using FlowJo v10 (Treestar, RRID:SCR_008520). Percentage of live cells was calculated relative to the number of live target cells after co-culture with non-transduced T cells. Dot plot data available as Source data file (Source Data file 2–4).

### Analysis of cytokine production via Cytokine bead array

Supernatants from 72 h co-culture were analyzed for multi-cytokine production utilizing LEGENDplex™ Human CD8/NK Panel (13-plex) (741065, Biolegend) according to manufacturer’s protocol. The samples were detected using LSRFortessa X20 (BD Biosciences) and analyzed using LEGENDplex™ Data Analysis Software Suite (Biolegend).

### T cell Immunophenotyping

Mock and CAR-transduced T cells were co-cultured with TRBC1^+^, TRBC2^+^ or TCR KO Jurkat. Target cells were then labeled with CellTrace™ CFSE (C34554, ThermoFisher Scientific) following manufacturer instructions. Effector and target cells were mixed at 1:1 E:T ratio for 72 h. Samples were harvested from cell culture plates and prepared for staining in 96-well plates (Greiner Bio-one). Cells were put through multiple rounds of staining, incubation and wash procedures in accordance with standard operating procedures, using a 16 color flow cytometry panel as indicated below. Cells were finally fixed (88-8824-00, eBiosciences) before being acquired on the LSRFortessa X20 (BD Biosciences). FMX controls (fluorescence minus multiple) were run alongside fully stained samples to assist in setting gates upon analysis.

**Table:** 

HLA-DR	564040	Becton Dickinson
CD8	612942	Becton Dickinson
CD27	741833	Becton Dickinson
CD4	612887	Becton Dickinson
CD45RA	566114	Becton Dickinson
CD57	393304	Biolegend
CD62L	563808	Becton Dickinson
TIM3	565566	Becton Dickinson
CD25	563701	Becton Dickinson
CFSE	C34554	ThermoFisher
TIGIT	46-9500-42	Invitrogen
RQR8	FAB7227A	R&D systems
CCR7	353236	Biolegend
PD-1	561272	Becton Dickinson
LAG3	369304	Biolegend
FVS780	565388	Becton Dickinson

### Antigen density

TCR density on healthy human T cells or TRBC1^+^ and TRBC2^+^ cell line was determined using anti TCR alpha/beta Monoclonal Antibody (WT31) PE conjugated (12-9955-42, eBioscience), 5 μg/sample. To convert the PE MFI value to the number of PE molecules bound per cell the BD Biosciences QuantiBRITE (340495, BD Bioscience) system was used. QuantiBRITE beads and stained cells were acquired the same day and data analyzed following manufacturer instructions.

### Trogocytosis and spiking assay

100 × 10^6^ Defrosted PBMCs were counted and resuspended at 10 × 10^6^ cells/mL of PBS containing 5 µg/mL of anti TRBC1-JOVI.1 Human IgG1-Fc (in house) and 5 µg/mL anti TRBC2 (in house) Murine IgG2a-Fc. The cells were stained for 10 min at RT and washed once with PBS. The cell were stained with secondary antibodies at a concentration of 10 × 10^6^ cells/mL of PBS containing 2 µg/mL of Goat anti-Human IgG (H + L) Cross-Adsorbed Secondary Antibody, Alexa Fluor™ 647 (Invitrogen, A-21445) and 2 µg/mL of Goat anti-Mouse IgG2a Cross-Adsorbed Secondary Antibody, Alexa Fluor™ 488 (Invitrogen, A-21131) and 1 µL/mL of eBioscience™ Fixable Viability Dye eFluor™ 450 (Invitrogen, 65-0863-14). The cells were stained for 10 min and then washed once with PBS. The cells were then counted and resuspended at 10 × 10^6^ cells/mL of EasySep™ Buffer (StemCell Tech, 20144) containing Antibiotic-Antimycotic (Gibco, 15240096). The cells were sorted with BD FACSMelody™ cell sorter (BD Biosciences) for the stringent expression of either TRBC1 or TRBC2, using 100 µm nozzle at 20 psi and adjust efficiency to 70% or higher. The cells were collected in complete media R10 supplemented with Antibiotic-Antimycotic (Gibco, 15240096) and kept on ice after sorting until all sample tubes are done. Sorted TRBC1^+^ or TRBC2^+^ T cells were then resuspended at 1 × 10^6^ cells/mL in R10 and stimulated with TransAct (Miltenyi Biotec; 130-111-160), 10 ng/ml IL-7 (Miltenyi Biotec; 130-095-367) and 10 ng/ml IL-15 (Miltenyi Biotec; 130-095-760). 48 h after, cells were collected, plated at a density of 1 × 10^6^ cells per well (1 mL) on retronectin-coated (Takara, T100B) 6-well plates in the presence of retroviral supernatant.

For trogocytosis, mock (Non Transduced PBMCs) and CAR-transduced T cells were co-cultured with autologous TRBC1^+^ and TRBC2^+^ non transduced T cells. Target cells were labeled with CellTrace™ Violet (C34557, ThermoFisher Scientific) following manufacturer instructions. Effector and target cells were mixed to reach a 1:1 E:T ratio, and incubated for 30 min, 1 h, 4 h and 24 h. Staining steps were performed at RT for 12 min, with PBS washes between steps. The cells were stained using 5 µg/mL biotinylated anti-TRBC2 murine IgG2a-Fc (in house) for 10 min at RT. The cells were then stained with FITC anti-human TCR Cβ1 Antibody (Biolegend, 383510), Streptavidin-PE (Biolegend, 405204), RQR8 specific anti Human CD34 APC-conjugated Antibody (R&D System, FAB7227A) and eBioscience™ Fixable Viability Dye eFluor™ 780 (Invitrogen, 65-0865-14). Cells were stained for 10 min at RT and washed once with PBS. The samples were stained in 96 well plate and resuspended in 100 μL of PBS. Flow cytometry was performed using the MacsQuantX flow cytometer (Miltenyi), running 50 μL of cell suspension. Data analysis was conducted using FlowJo v10 (Treestar, RRID:SCR_008520). Target cells were identified by negativity for RQR8 and positivity for CellTrace™ Violet. Effector cells were evaluated by negativity for CellTrace™ Violet. CAR T cells were evaluated by the positivity for anti CD34.

For spiking assay, mock and CAR-transduced T cells were co-cultured with autologous TRBC1^+^ and TRBC2^+^ non transduced T cells. Target cells were labeled with CellTrace™ Violet following manufacturer instructions. TRBC1^+^ target cells were added to mock and CAR-transduced TRBC2 cells to reach a percentage of 0%, 1%, 5% and 10%. Similarly, TRBC2^+^ target cells were added to mock and CAR-transduced TRBC1 cells. The cells were incubated for 6 days and then analyzed for memory phenotype and exhaustion phenotype. Staining steps were performed at RT for 12 min, with PBS washes between steps. Transduced cells were identified by RQR8 positivity utilizing a CD34-specific antibody, and live cells selected via 7AAD live dead cell dye exclusion. Memory and exhaustion phenotype were evaluated in CD3^+^ (Milteny, 130-113-142) CAR T cells based on the expression of CD45RA (Mylteny, 130-117-747), CCR7 (Miltenyi, 130-119-583), PD-1 (Miltenyi, 130-130-654), CXCR5 (Miltenyi, 130-122-795), and TIM-3 (Miltenyi, 130-121-334). The samples were stained in 96 well plate and resuspended in 100 μL of PBS. Flow cytometry was performed using the MacsQuantX flow cytometer (Miltenyi), running 50 μL of cell suspension. Data analysis was conducted using FlowJo v10 (Treestar, RRID:SCR_008520).

### Animal study approval

All procedures in this study gained the approval of The Animal Welfare and Ethical Review Body and the United Kingdom Home Office (Autolus PPL No. P244BBE6B). All procedures are performed in accordance with the United Kingdom Home Office Animals (Scientific Procedures) Act 1986 and in adherence to Imperial College London or Autolus SOPs. This study is necessary and justifiable with due consideration to the ‘3Rs’ (the reduction, refinement, and replacement of animals in research). All animals were co-housed in a specific pathogen free facility.

### Establishment of T cell acute lymphoblastic leukemia (T-ALL) xenograft mouse models

For the generation of the Jurkat T-ALL model 10 to 14-week-old female NSG mice (NOD scid gamma; NOD.Cg-Prkdc^scid^Il2rg^tm1Wjl^/SzJ) (Charles River 614NSG) received intravenous injections of 5 × 10^6^/mouse firefly luciferase-transduced TRBC1^+^ or 1 × 10^6^/mouse firefly luciferase-transduced TRBC2^+^ Jurkat cells at day -10 relative to CAR-T infusion. On day 0, mice (*n* = 6/cohort) received intravenous infusions of either 1 × 10^6^ (Supplementary Figs. 6, 7) or 5 × 10^6^ (Fig. 5e–g) transduced CAR-T cells.

For the generation of the HPB-ALL T-ALL model 10 to 14-week-old female NSG mice (NOD SCID gamma; NOD.Cg-Prkdc^scid^Il2rg^tm1Wjl^/SzJ) (Charles River 614NSG) received intravenous injections of 2.5 × 10^6^/mouse luciferase-transduced HPB-ALL day -7 relative to CAR-T infusion. On day 0, mice (*n* = 6/cohort) received intravenous infusions of 5 × 10^6^ transduced CAR-T cells (Fig. 5a–c and Supplementary Figs. 18–19).

The mice were imaged bi-weekly for bioluminescence signal from tumor cells using the IVIS® system (IVIS, Xenogen Corporation, Alameda, CA) 10–15 min after 150 mg/kg D-luciferin (Xenogen) per mouse was injected intraperitoneally. Mice were euthanized if body weight loss ≥20% or high tumor burden was reached (1e10 BLI). Animals were sacrificed by Isoflurane inhalation followed by cervical dislocation. Bone marrow from lower limbs was collected at the time of euthanasia for tumor and CAR-T cell tracking.

In vivo experiments were performed once for each of the Jurkat TRBC1, Jurkat TRBC2, HPB-ALL TRBC1 and HPB-ALL TRBC2 mouse models reported.

### Flow cytometry analysis of mouse bone marrow

Animals were sacrificed by Isoflurane inhalation followed by cervical dislocation. Bone marrow was collected by centrifugation of lower limb bones, after the epiphyses were cut. Erythrocytes were lysed using 500 μL of ACK lysis buffer (Sigma, A1049201) for 5 min at RT. Cells were strained through a 70 μm cell strainer. 1 × 10^6^ cells were aliquoted for phenotyping by flow cytometry. First, the Fc receptor was blocked using anti-CD32/CD16 (BioLegend, 156604, RRID: AB_2783138) to avoid non-specific binding. Transduced CAR-T cell populations were identified based on the expression of CD45 (Biolegend, 103132, RRID: AB_893340), CD3 (Biolegend, 100236, RRID:AB_2561456), CD4 (Biolegend, 116004, RRID:AB_313688), RQR8 (R&D, FAB7227A, R&D System), CAR (aJOVI/KFN idiotype) and the absence of CD11b (Biolegend, 101216, RRID, AB_312799). The identification of tumors was performed by the detection of mCLOVER and either TRBC1 or TRBC2.

The samples were stained in 96 well plates and resuspended in 100 μL of PBS. Flow cytometry was performed using the MacsQuantX flow cytometer (Miltenyi), running 50 μL of cell suspension. Data analysis was conducted using FlowJo v10 (Treestar, RRID:SCR_008520).

### Statistical analysis

Data were presented as mean ± SD unless indicated otherwise. Graphs and statistics were generated using Prism 9.0 software for Windows (Graphpad Software Inc., La Jolla, CA, RRID:SCR_000306). The differences between means were calculated using two-tailed unpaired *t*-test, one-way ANOVA, and two-way ANOVA. Dunnett’s, Sidak’s or Tukey’s correction for multiple comparisons was used to calculate adjusted p-value when appropriate.

Specific statistical test used for each figure was described in the corresponding figure legend and Source data file. Survival determined from the time of tumor cell injection was analyzed by the Kaplan–Meier method and differences in survival between groups were compared by the log-rank test.

*P* values: ns *P* > 0.05, *P ≤ 0.05, **P ≤ 0.01, ***P ≤ 0.001, ****P ≤ 0.0001.

### Reporting summary

Further information on research design is available in the Nature Portfolio Reporting Summary linked to this article.

## Supplementary information

Supplementary Information

Reporting Summary

## Source data

Source Data

## Data Availability

All data is available in the manuscript, the supplementary information, or Source Data file. Crystal structure co-ordinates have been deposited at the protein data bank (PDB) with the following ID numbers: 7AMP, 7AMQ, 7AMR and 7AMS. Source data are provided as a Source Data file with this paper. Source data are provided with this paper.
