# Supplementary material for: Structure-guided engineering of immunotherapies targeting TRBC1 and TRBC2 in T cell malignancies
Source: Nat Commun. 2024 Feb 21;15:1583. doi: 10.1038/s41467-024-45854-3 (PMC10881500; doi:10.1038/s41467-024-45854-3)
Supplement: Supplementary file 1 — Supplementary Information [file 41467_2024_45854_MOESM1_ESM.pdf]

Structure-Guided Engineering of Immunotherapies Targeting TRBC1 and TRBC2 in T Cell Malignancies

Supplementary Materials

Supplementary Figures

Supplementary Fig. 1: TRBC1/TRBC2 homology

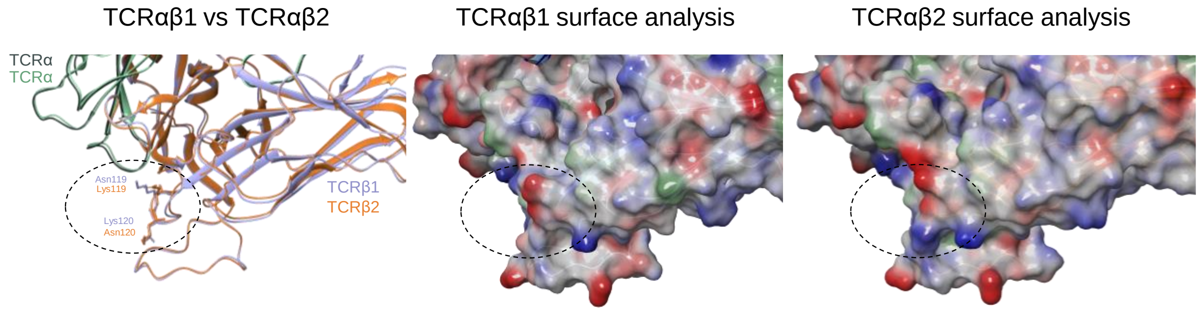

Overlay of TCR proteins containing the αβ1 (blue) or αβ2 (orange) chains showing almost complete overlap with the exception of the Asn119/Lys120 and Lys119/Asn120 for TRBC1 and TRBC2, respectively (dashed circle). Central and right panels show surface analysis of TCRαβ1 and TCRαβ2, showing very comparable surface charge (blue=positive and red=negative) and hydrophobic patch (green) distribution.

Supplementary Fig. 2: SPR humanized Jovi-1

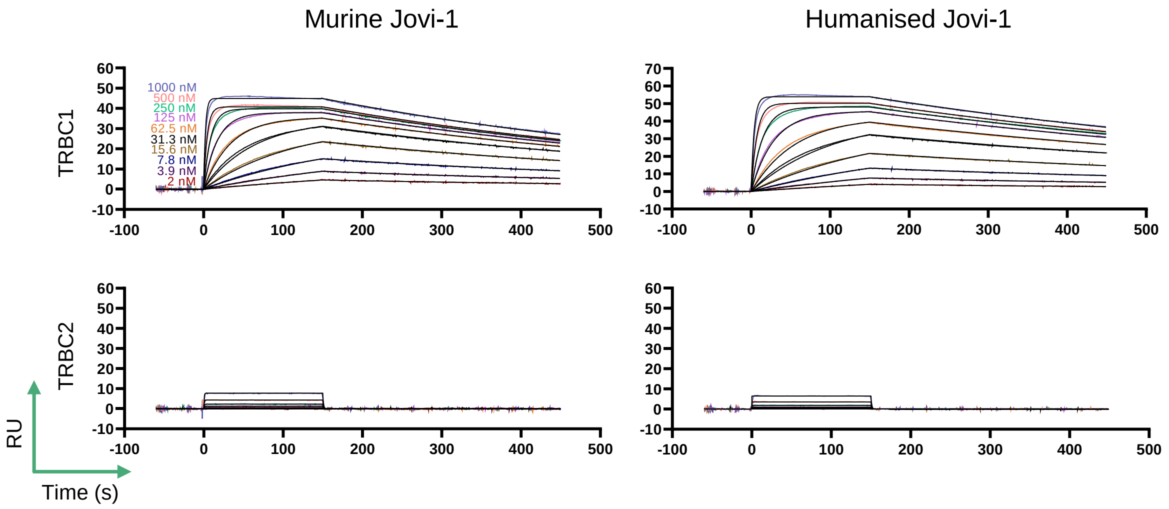

Affinity determination of murine (Mu)Jovi-1 and HuJovi-1 binding to TRBC1 (top) and TRBC2 (bottom) recombinant TCR HA1.7. Jovi-1/TRBC1 KD = 1.60 nM, HuJovi-1/TRBC1 KD = 2.57 nM. Source data are provided as a Source Data file.

Supplementary Fig. 3 Humanized Jovi-1 CAR expression profile

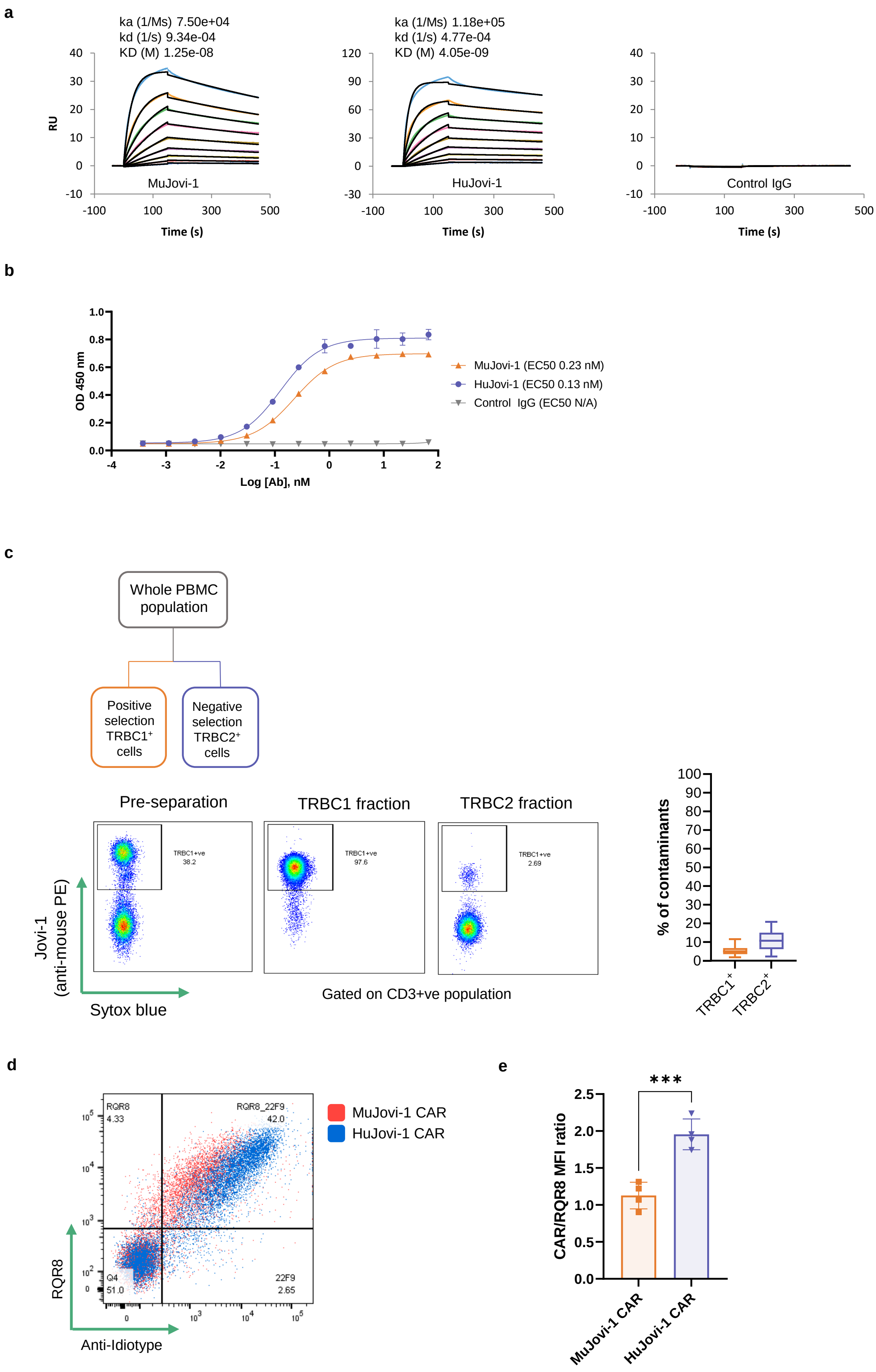

a, Surface plasmon resonance affinity kinetics of anti-idiotype antibody for MuJovi-1 (left), HuJovi-1 (center) or non-related antibody (right). **b**, ELISA binding titration of anti-idiotype antibody for MuJovi-1, HuJovi-1 or non-related control antibody in human IgG1 format. Anti-idiotype detected with Anti-rabbit IgG HRP conjugated secondary antibody. EC50 value obtained with Graphpad Prism v9. n=2 technical replicate, data shown as mean ± SD. **c**, Overview of PBMC sorting in TRBC1^{+} and TRBC2^{+} populations (top). (Bottom) Human PBMCs are first sorted using biotinylated muJovi-1 antibody via magnetic biotin selection kit. Unbound fraction corresponding to TRBC2^{+} cells. Bound fraction is eluted from beads and corresponds to TRBC1^{+} population. Staining obtained with muJovi-1 PE conjugated on CD3^{+} gated population. (Right) % of contaminating TRBC1^{+} (orange) or TRBC2^{+} (blue) cells from 27 individual processed donors. Data shown as box and whiskers min to max. **d**, Example of flow cytometry analysis of PBMC transduction efficiency for MuJovi-1 CAR (red) and HuJovi-1 CAR (blue). Transduction and expression of CAR detected with anti-CD34 Ab (for RQR8 marker) and anti-idiotype (for CAR). Similar degree of transduction efficiency was determined for both CAR constructs. **e**, Ratio of RQR8 (via anti-CD34) and CAR (via anti-idiotype) MFI signal for MuJovi-1 (orange) and HuJovi-1 (blue) CAR constructs. Significantly higher expression detected for HuJovi-1 CAR. Unpaired t test, *** $p < 0.001$, n=4 individual PBMC donors. Data shown as mean ± SD. Source data are provided as a Source Data file.

Supplementary Fig. 4. TCR expression

a

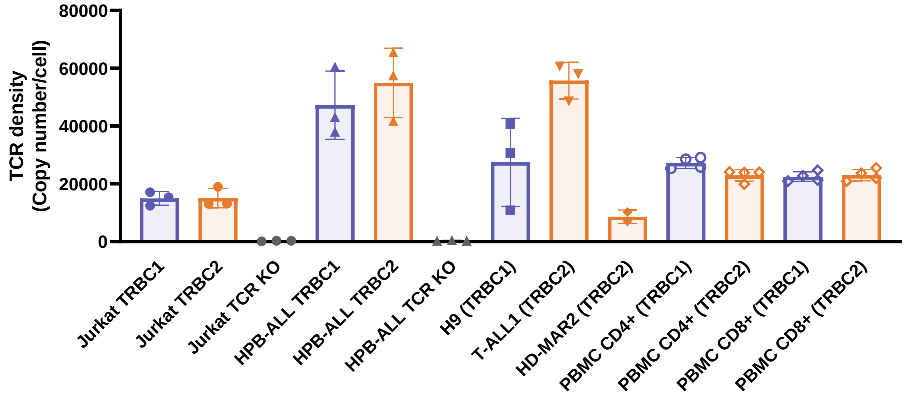

b

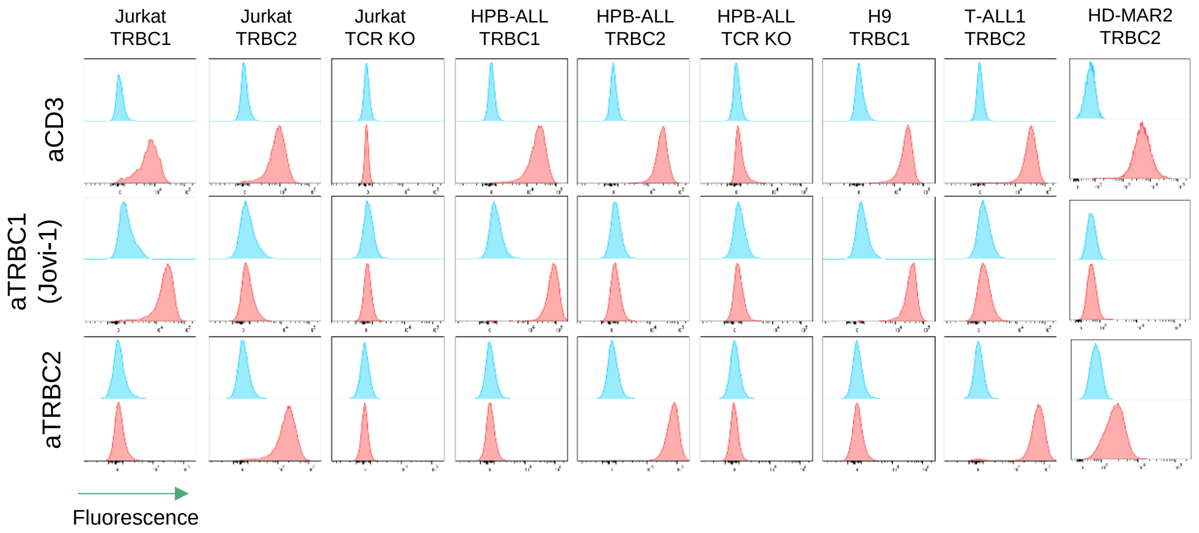

c

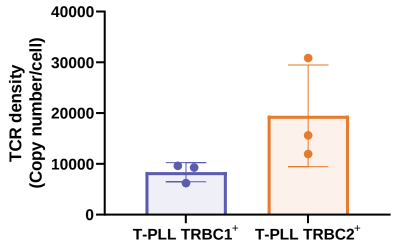

a, TCR expression levels on endogenously expressing cell lines Jurkat TRBC1 (n=3 technical replicate), H9 TRBC1 (n=3 technical replicate), HPB-ALL TRBC2 (n=3 technical replicate), T-ALL1 TRBC2 (n=3 technical replicate), HD-MAR2 TRBC2 (n=2 technical replicate), engineered cell lines (n=3 biologically independent samples) and healthy human PBMC CD4+ and CD8+ (n=4 biologically independent samples). TCR detected with antibody clone WT31.

Data shown as mean ± SD**.** **b**, TRBC chain profiling on T cell lines. TCR expression detected with anti-CD3ε, TRBC1 expression detected with MuJovi-1 and TRBC2 expression detected with modified KFN aTRBC2 antibody (biotin). **c**, TCR expression levels on primary T-PLL tumor samples TRBC1$^+$ (blue, n=3 biologically independent samples) and TRBC2$^+$ (orange, n=3 biologically independent samples). TCR detected with antibody clone WT31. Data shown as mean ± SD. Range of TCR$^+$ cells within samples was 79.8-96.3% and 95.6-97.9% for T-PLL TRBC1$^+$ and T-PLL TRBC2$^+$, respectively. Source data are provided as a Source Data file.

Supplementary Fig. 5. Characterization of HuJovi-1 CAR

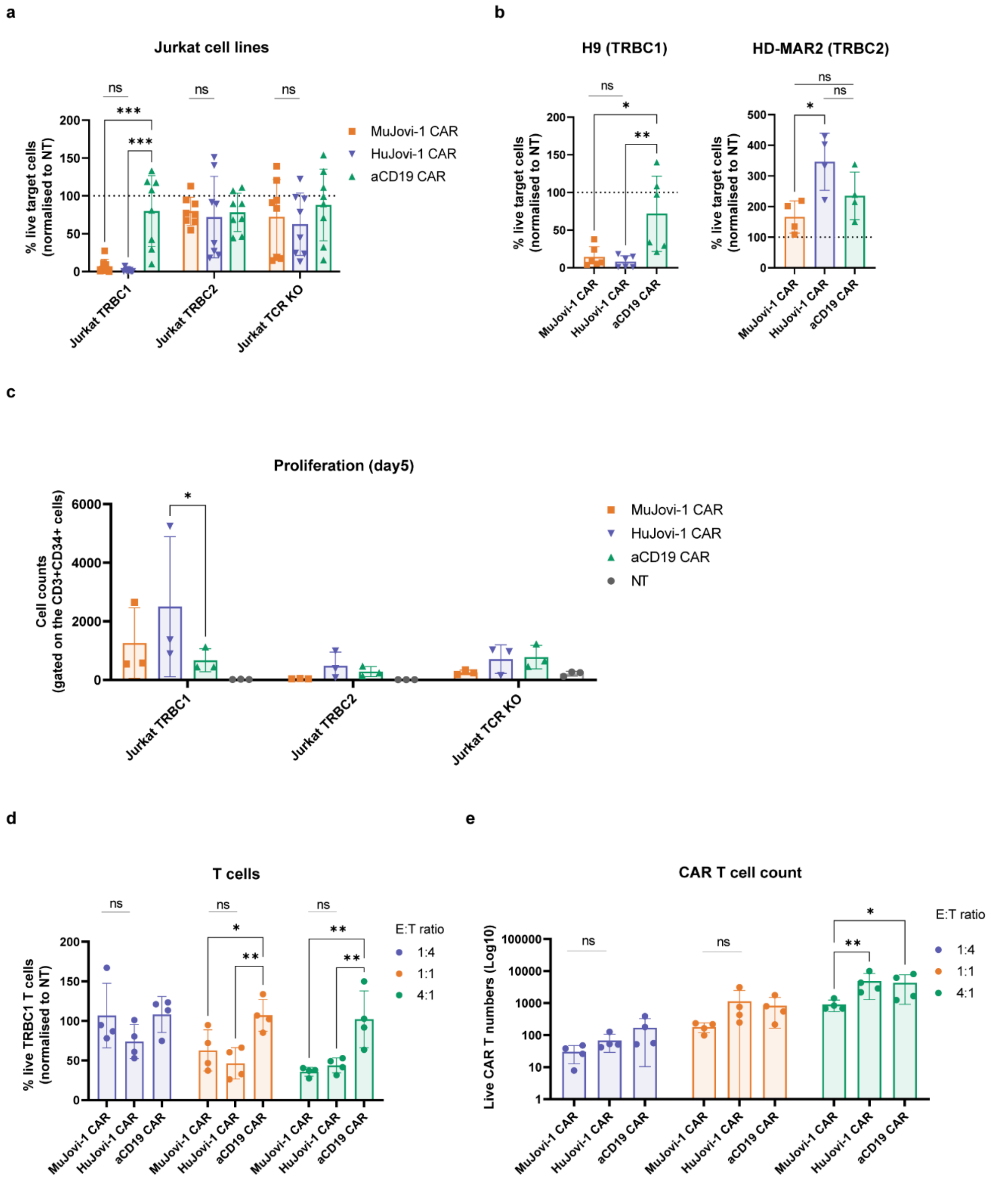

Cytotoxicity assay showing efficacy of MuJovi-1 CAR (orange) compared with HuJovi-1 CAR (blue) on WT and engineered Jurkat cell lines (n=8 biologically independent samples) (**a**), on H9 TRBC1 (n=6 biologically independent samples), and HD-MAR2 TRBC2 (n=4 biologically independent samples). Data shown as mean ± SD. (**b**). Non-targeting aCD19 CAR (green) was used as a negative control CAR construct (1:4 E:T ratio, 72h). No killing was observed above background for TCR KO or TRBC2^{+} cells. Efficacy of HuJovi-1 CAR was equivalent to the murine CAR. * $p < 0.05$, ** $p < 0.01$, *** $p < 0.001$. Two-way ANOVA with Dunnett's post

test. Data shown as mean ± SD. Ns, not significant; NT, Non-transduced CAR T cells. **c**, CAR T cell expansion following 5 days incubation with Jurkat target cells at 1:4 E:T ratio (n=3 biologically independent samples). Data shown as mean ± SD. Two-way ANOVA with Dunnett's post-test against aCD19 CAR cohort. * $p < 0.05$. **d**, Cytotoxicity assay for MuJovi-1 and HuJovi-1 CARs against healthy T cells (TRBC1^{+}) at 72h with 1:4, 1:1 and 4:1 E:T ratios (n=4). **: $p < 0.01$, *: $p < 0.05$. Two way ANOVA with Dunnett's post test. Data shown as mean ± SD. Ns, not significant; NT, Non-transduced CAR T cells. **e**, CAR T cell count at 72h post incubation with healthy donor T cells (in **d**) at 4:1, 1:1 and 1:4 E:T ratios (n=4 biologically independent samples). No reduction in cell count for HuJovi-1 CAR compared to aCD19 CAR (i.e. no reverse killing). Data shown as mean ± SD. * $p < 0.05$, ** $p < 0.01$. Two-way ANOVA with Turkey's post test. Ns = not significant. Source data and exact p-values are provided as a Source Data file.

Supplementary Fig. 6. *In vivo* characterization of HuJovi-1 CAR

a

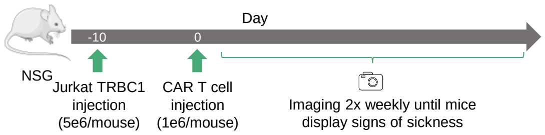

b

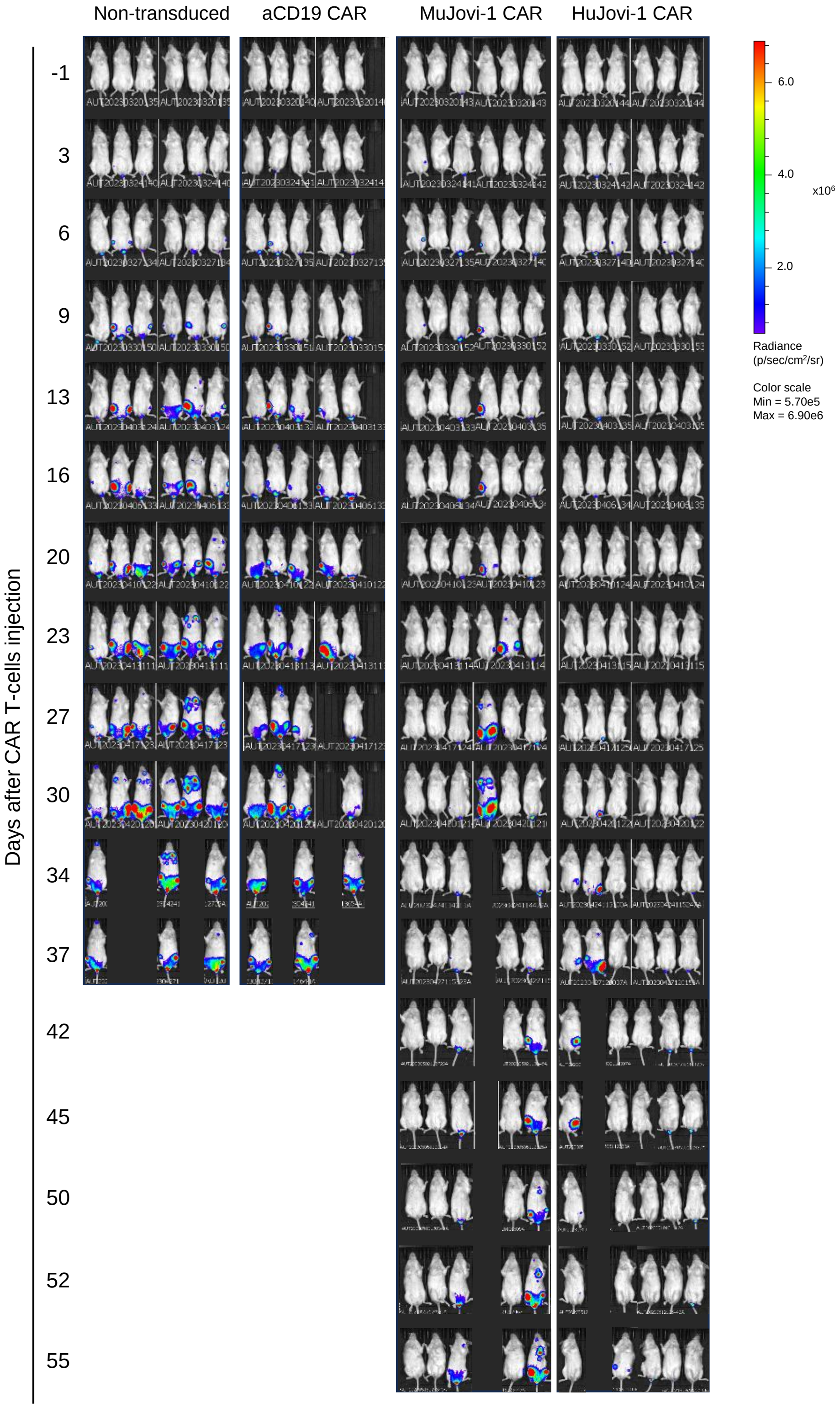

a, Schematic of NSG model with 5e6 Jurkat TRBC1^{+} cells/animal, treated with 1e6 CAR T cells/mouse (n = 6/group, aCD19 CAR n = 5). **b**, Total radiance bioluminescent imaging (BLI) of treated mice.

Supplementary Fig. 7. In vivo characterization of HuJovi-1 CAR

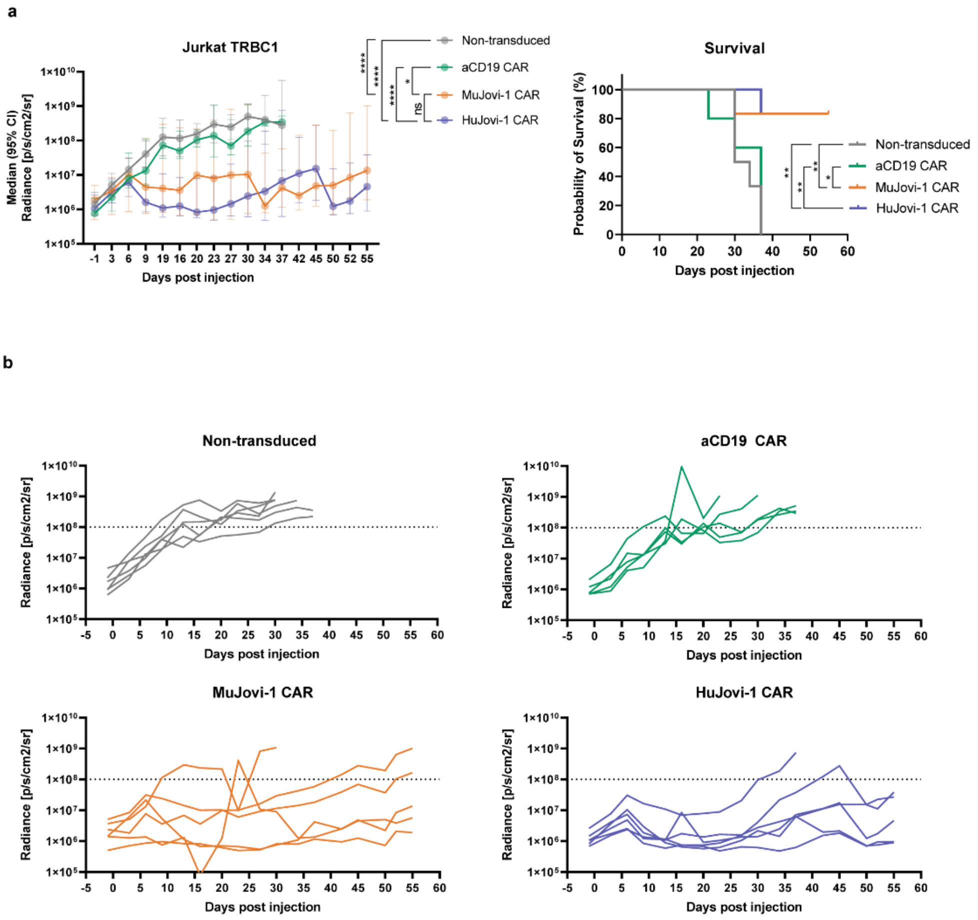

a, Median total radiance BLI of Jurkat $TRBC1^+$ tumor burden post CAR-T cell injection (n = 6, aCD19 CAR n = 5) (left) (Data shown as median with 95% CI) and Kaplan-Meier survival curve (cut-off 1e9 p/s/cm^2/sr) (right). Two-way ANOVA with Sidak's post test ns = non-significant. Log-rank test * $p < 0.05$, ** $p < 0.01$. **b**, Individual mouse total flux radiance BLI of Jurkat $TRBC1^+$ tumor burden (in **a**) post CAR-T cell injection (n = 6, aCD19 CAR n = 5). Source data and exact p-values are provided as a Source Data file.

Supplementary Fig. 8: Affinity profile of HuJovi-1 derived clones

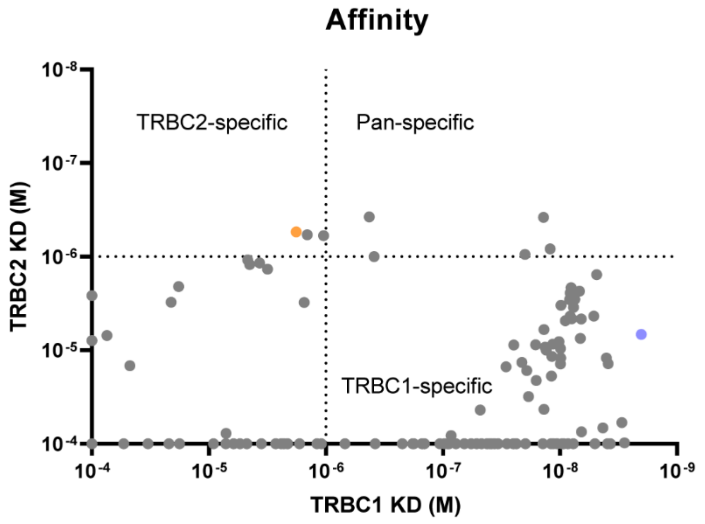

Affinity (KD = M) of *in silico*-derived HuJovi-1 mutants towards TRBC1 and TRBC2. Orange = KFN (Mut3); blue = HuJovi-1; grey = HuJovi-1 mutants with mutations on positions 26, 27, 28, 31, 32, 94, 96, 97 and 98 of the heavy chain (Kabat numbering). When kinetic could not be measured, value was fixed at 1e-4. Source data provided as a Source Data file.

Supplementary Fig. 9: Identification of HuJovi-1 variants with increased TRBC2 binding affinity using phage display.

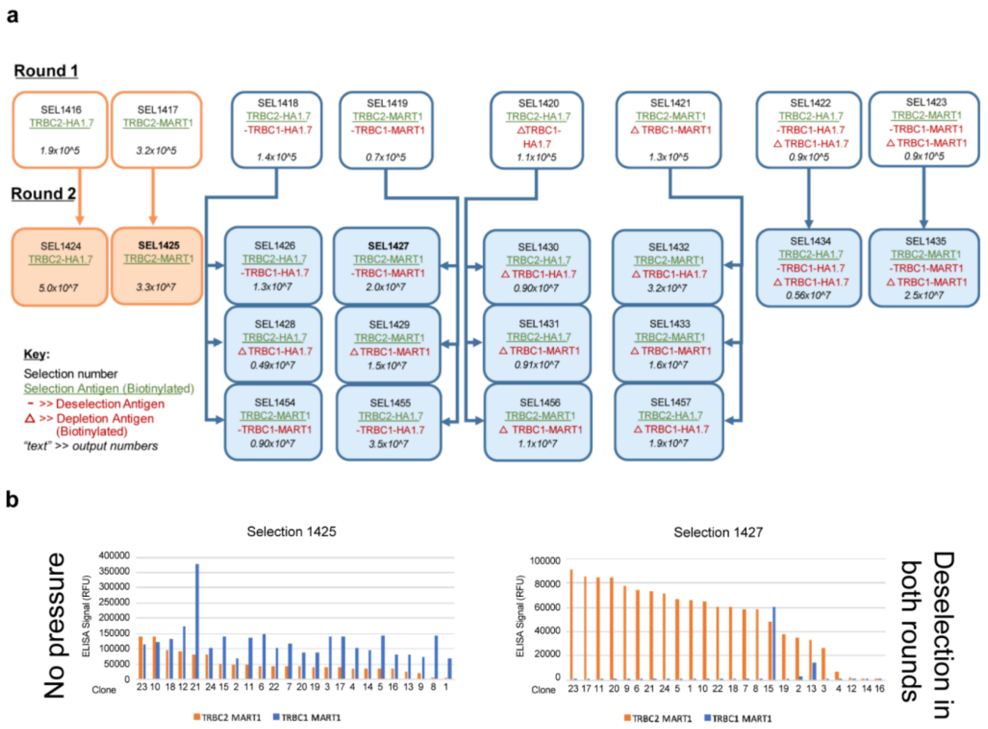

a, Detailed selection strategy for TRBC2 enrichment from structural design phage library. For each panning strategy, the selection antigen is shown in green and the de-selection antigen is shown in red. Δ indicates depletion. Round one input titers are shown while output titers are shown for round two. **b**, Representative phage ELISA output from two panning strategies. Left: 1425 was selected for TRBC2 with no pressure for TRBC1, while 1427 was selected under selection pressure from TRBC1 for both rounds one and two.

Supplementary Fig. 10: Molecular interface between KFN and TRBC1.

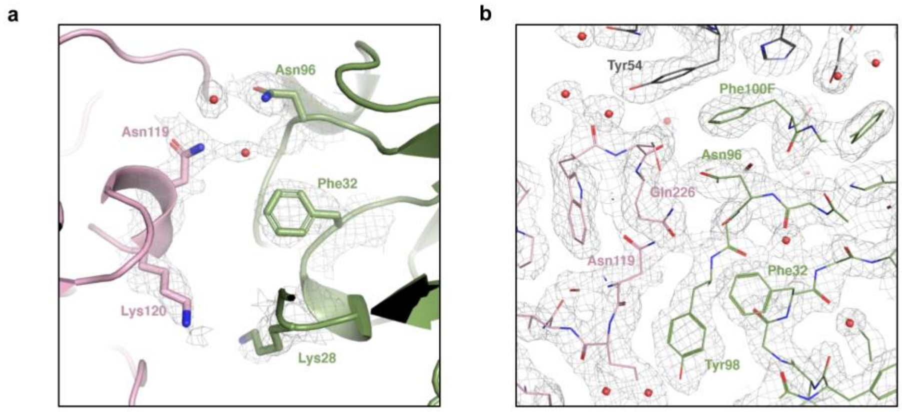

a, Close-up view of the interactions between the TRBC1 and the CDRs of the KFN Fab (PDB ID 7AMR). The TCR β-chain is colored in pink, the heavy chain is colored in light green. Residues Asn119 and Lys120, of the TCR β-chain, and Lys28, Phe32 and Asn96 (Kabat) are shown as stick representations. **b**, Electron density of the CDRs around the epitope of the TCR. The TCR β-chain is colored in pink while the heavy and light chains are colored in green and grey, respectively.

Supplementary Fig. 11: PBMC TRBC1/TRBC2 staining.

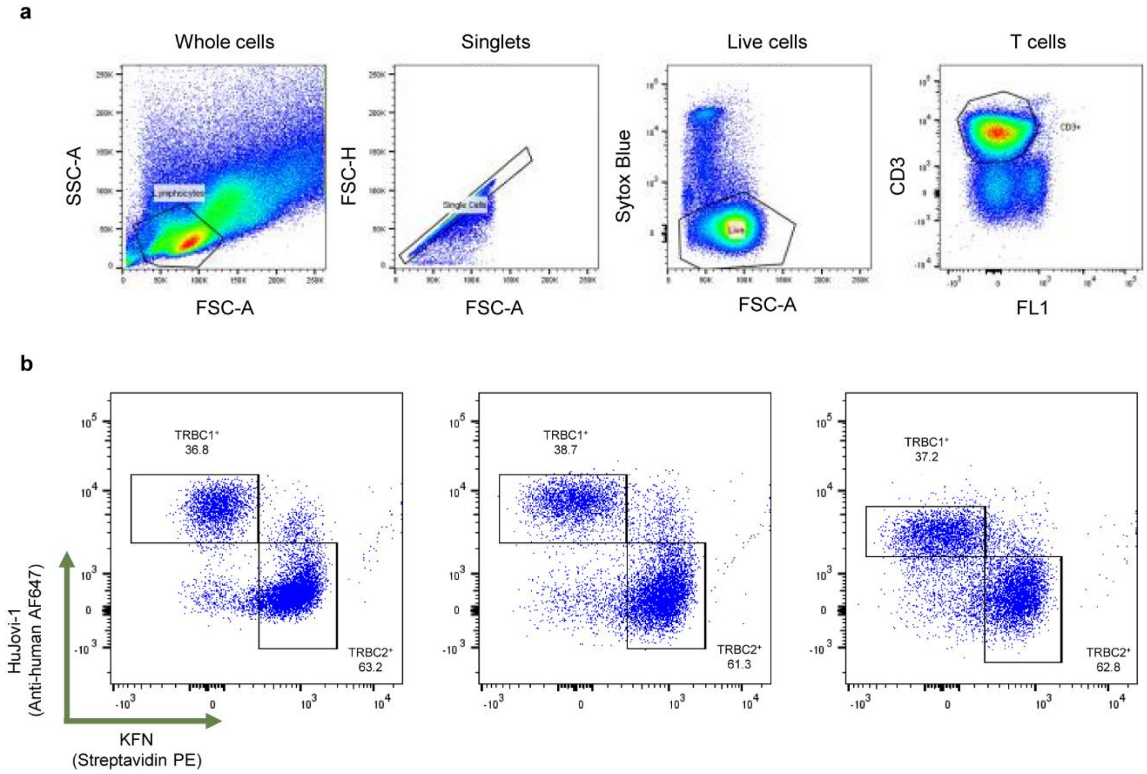

a, Gating strategy for human PBMC CD3^{+}. **b**, Dot plot of three independent stainings of healthy donor PBMCs with HuJovi-1 IgG (anti-human AF647) and biotinylated KFN IgG muFc (Streptavidin-PE). % of TRBC1^{+} relative to TRBC2^{+} populations in gated area depicted in graphs.

Supplementary Fig. 12: Kinetic binding profiles of Jovi-1 and KFN antibodies to TRBC1 and TRBC2.

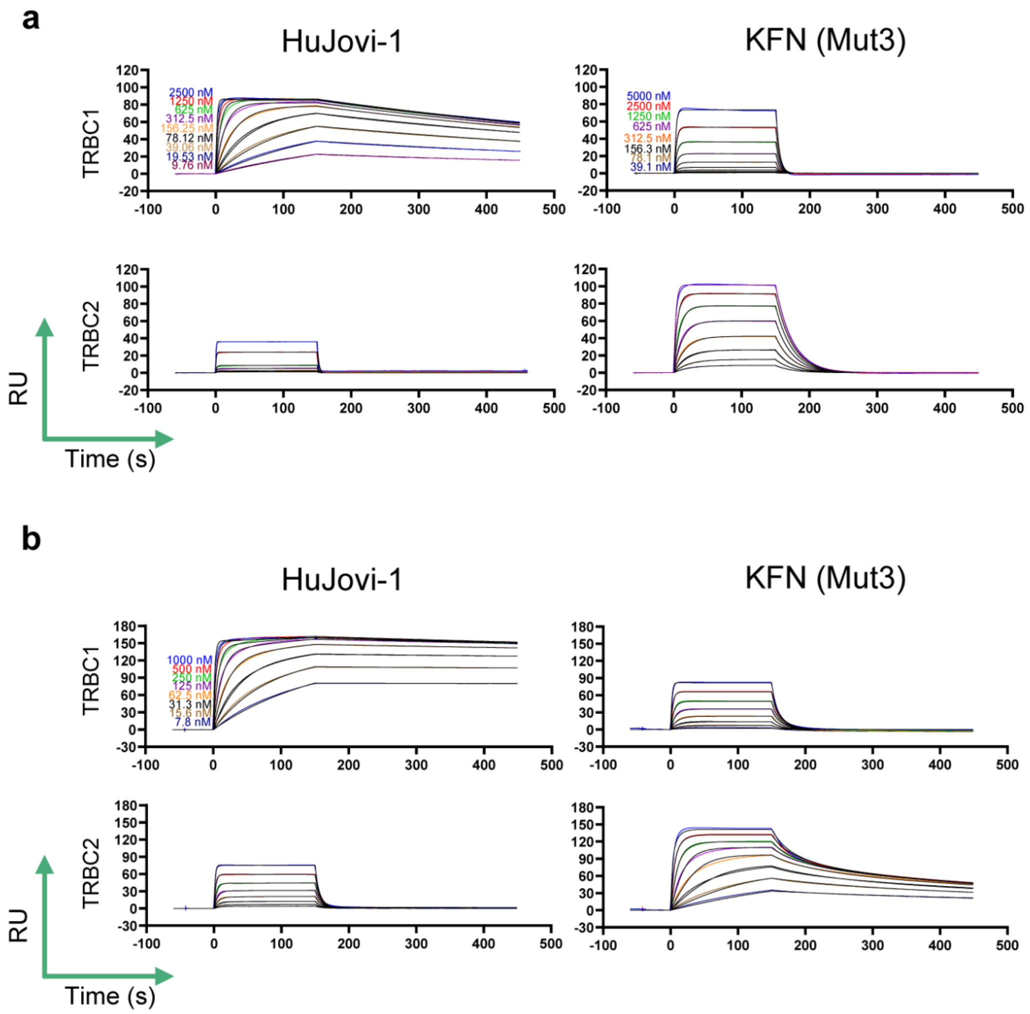

a, Kinetic profiles of HuJovi-1 and KFN for TRBC1 and TRBC2 measured by SPR according to a 1:1 Langmuir model. **b**, Reverse kinetic profiles of chip immobilized TRBC1 and TRBC2 against huJovi-1 and KFN IgG, to determine avidity impact on the interaction (Bivalent analyte fit). Source data provided as a Source Data file.

Supplementary Fig. 13: Solubility and stability of TRBC1 and 2 antibodies.

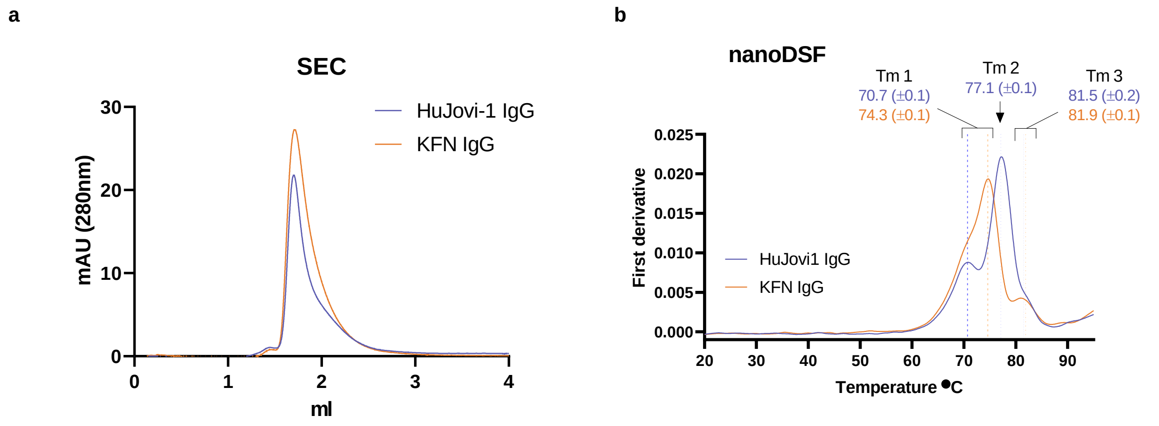

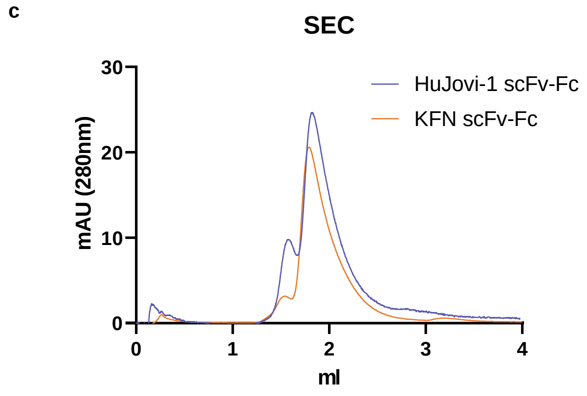

Clone	Aggregates by SEC (%)	Apparent MW by SEC (kDa)	Tm (°C)
HuJovi-1 IgG	2.6 (±0.09)	233 (±4)	70.7 (±0.1)
HuJovi-1 scFv-Fc	12.2 (±3)	142 (±1)	62.8 (±0.1)
KFN IgG	0.54 (±0.5)	220 (±1)	74.3 (±0.1)
KFN scFv-Fc	4.8 (±2.1)	148 (±14)	61.3 (±0.1)

Solubility (**a**) and stability (**b**) of HuJovi-1 and KFN antibodies in IgG format. **c,** Solubility of HuJovi-1 and KFN antibodies in scFv-Fc format. Summary of stability parameters in the two antibody formats. Data expressed as mean ± SD. Source data provided as a Source Data file.

Supplementary Fig. 14. Functional characterization of HuJovi-1 CAR

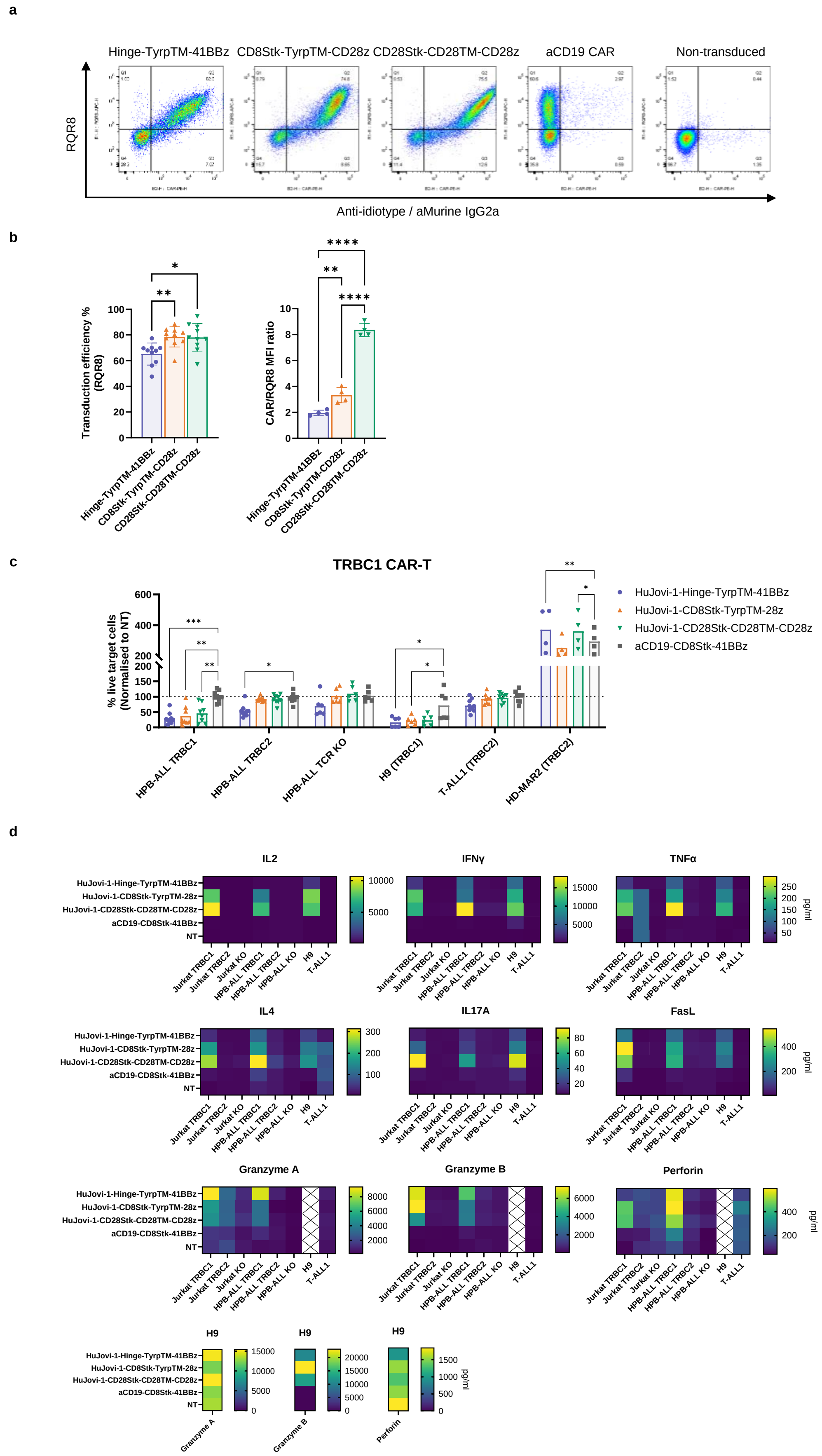

a, Representative dot plot for HuJovi-1 CAR T cells stained with anti-CD34 (for RQR8 marker protein) and anti-idiotype (for CAR). **b**, % transduction efficiency based on $RQR8^+$ CAR T cell population by flow cytometry (left, n = 10 biologically independent samples) and surface CAR expression as CAR (anti-idiotype)/RQR8 (anti-CD34) ratio (right, n= 4 biologically independent samples). Data shown as mean ± SD. One-way ANOVA with Tukey's post test, * p < 0.05, ** p < 0.01, **** p < 0.0001. **c**, FACS-based killing of HPB-ALL TRBC1 (n=9 biologically independent samples), HPB-ALL TRBC2 (n=9 biologically independent samples), HPB-ALL TCR KO (n=6 biologically independent samples), H9 TRBC1 (n=6 biologically independent samples), T-ALL1 TRBC2 (n=9 biologically independent samples) and HD-MAR2 TRBC2 (n=4 biologically independent samples) cell lines by HuJovi-1 CAR T cells at 1:8 E:T ratio, 72h. Data shown as mean ± SD. *p < 0.05, ** p <0.01, *** p>0.001 by two-way ANOVA and Dunnett's test for multiple comparisons versus aCD19 CAR. **d**, Cytometric bead array assay measurement for cytokine and cytolytic mediators by HuJovi-1 CAR-T cells against target cell lines. H9 values for Granzyme A, Granzyme B and Perforin were plotted separately due to high constitutive expression. Data presented as geometric mean. Source data and exact p-values are provided as a Source Data file.

Supplementary Fig. 15. Functional characterization of KFN CAR

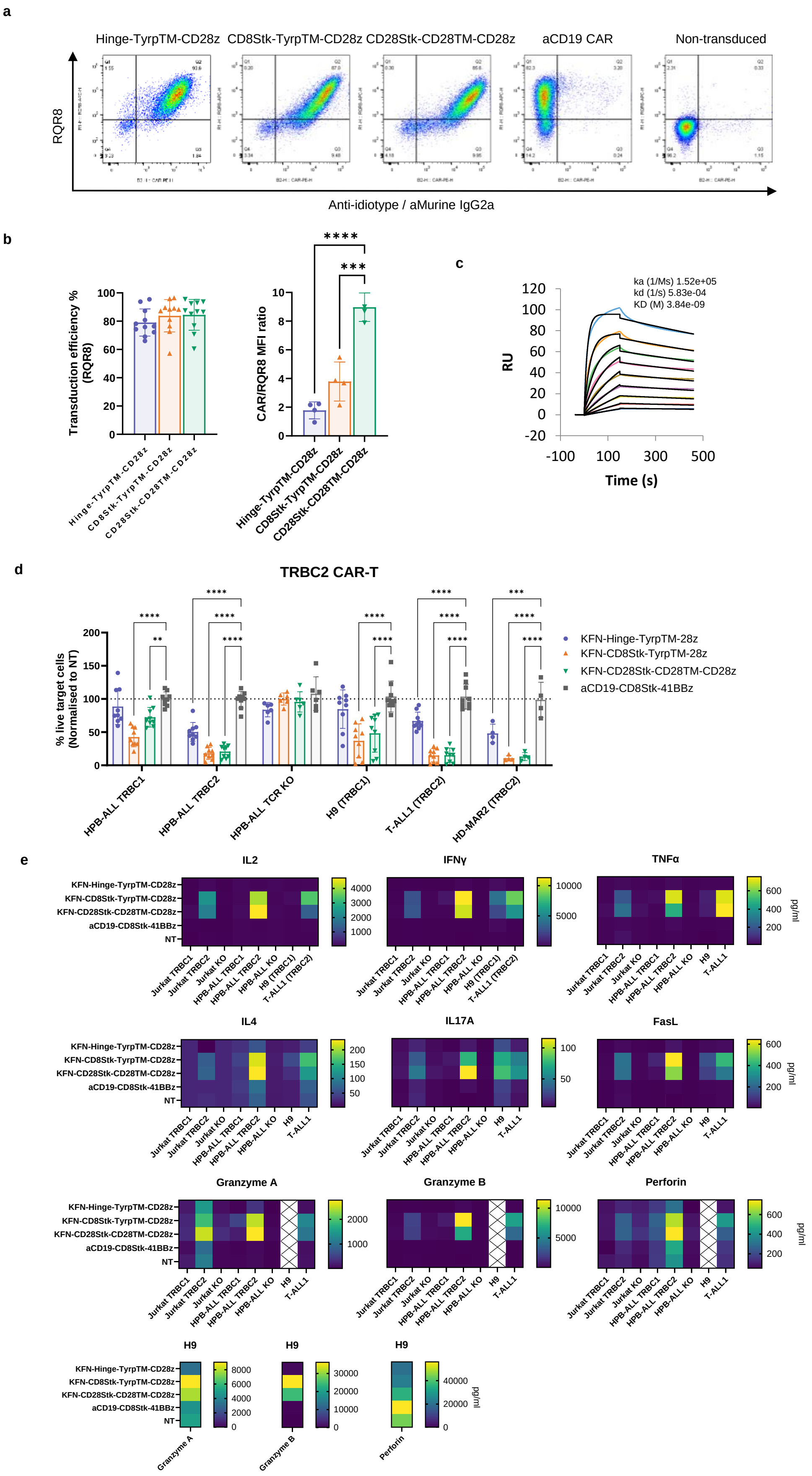

a, Representative dot plot for KFN CAR-T cells stained with anti-CD34 (for RQR8 marker protein) and anti-idiotype (for CAR). **b**, % transduction efficiency based on RQR8$^+$ CAR-T cell population by flow cytometry (left, n = 11 biologically independent samples) and surface CAR expression as CAR (anti-idiotype)/RQR8 (anti-CD34) ratio (right, n = 4 biologically independent samples). Data shown as mean ± SD. One-way ANOVA with Tukey's post test, *** $p < 0.001$, **** $p < 0.0001$. **c**, Surface plasmon resonance (SPR) affinity kinetics of anti-idiotype antibody for KFN antibody. **d**, Flow cytometry-based killing of HPB-ALL TRBC1 (n=9 biologically independent samples), HPB-ALL TRBC2 (n=9 biologically independent samples), HPB-ALL TCR KO (n=6 biologically independent samples), H9 TRBC1 (n=6 biologically independent samples), T-ALL1 TRBC2 (n=9 biologically independent samples) and HD-MAR2 TRBC2 (n=4 biologically independent samples) cell lines by HuJovi-1 CAR-T cells at 1:8 E:T ratio, 72h. Data shown as mean ± SD. *$p < 0.05$, ** $p < 0.01$, *** $p < 0.001$ by two-way ANOVA and Dunnett's test for multiple comparisons versus aCD19 CAR. **e**, Cytometric bead array assay measurement for cytokine and cytolytic mediators by HuJovi-1 CAR-T cells against target cell lines. H9 values for Granzyme A, Granzyme B and Perforin were plotted separately due to high constitutive expression. Data presented as geometric mean. Source data and exact p-values are provided as a Source Data file.

Supplementary Fig. 16. Gating strategy for flow cytometry-based killing assay

a

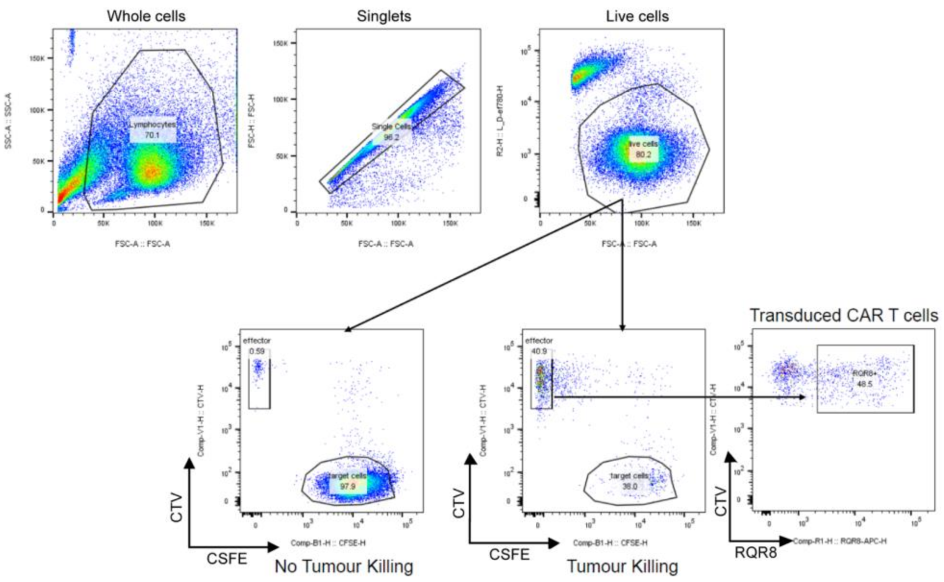

b

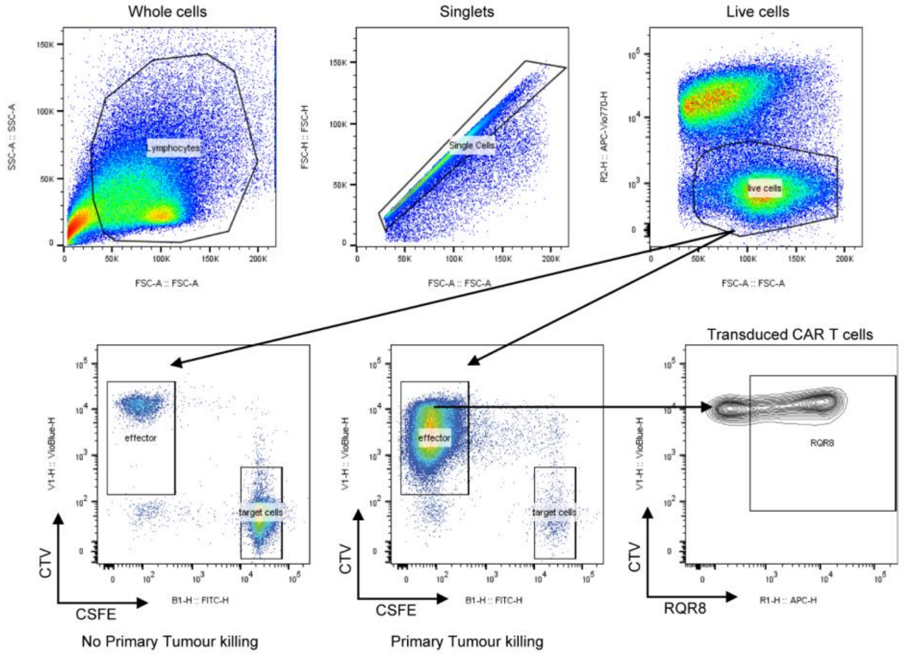

Representative gating strategy for flow-cytometry-based killing assay against target cell lines and normal T cells (**a**) or primary tumor samples (**b**).

Supplementary Fig. 17. CAR T cell trogocytosis

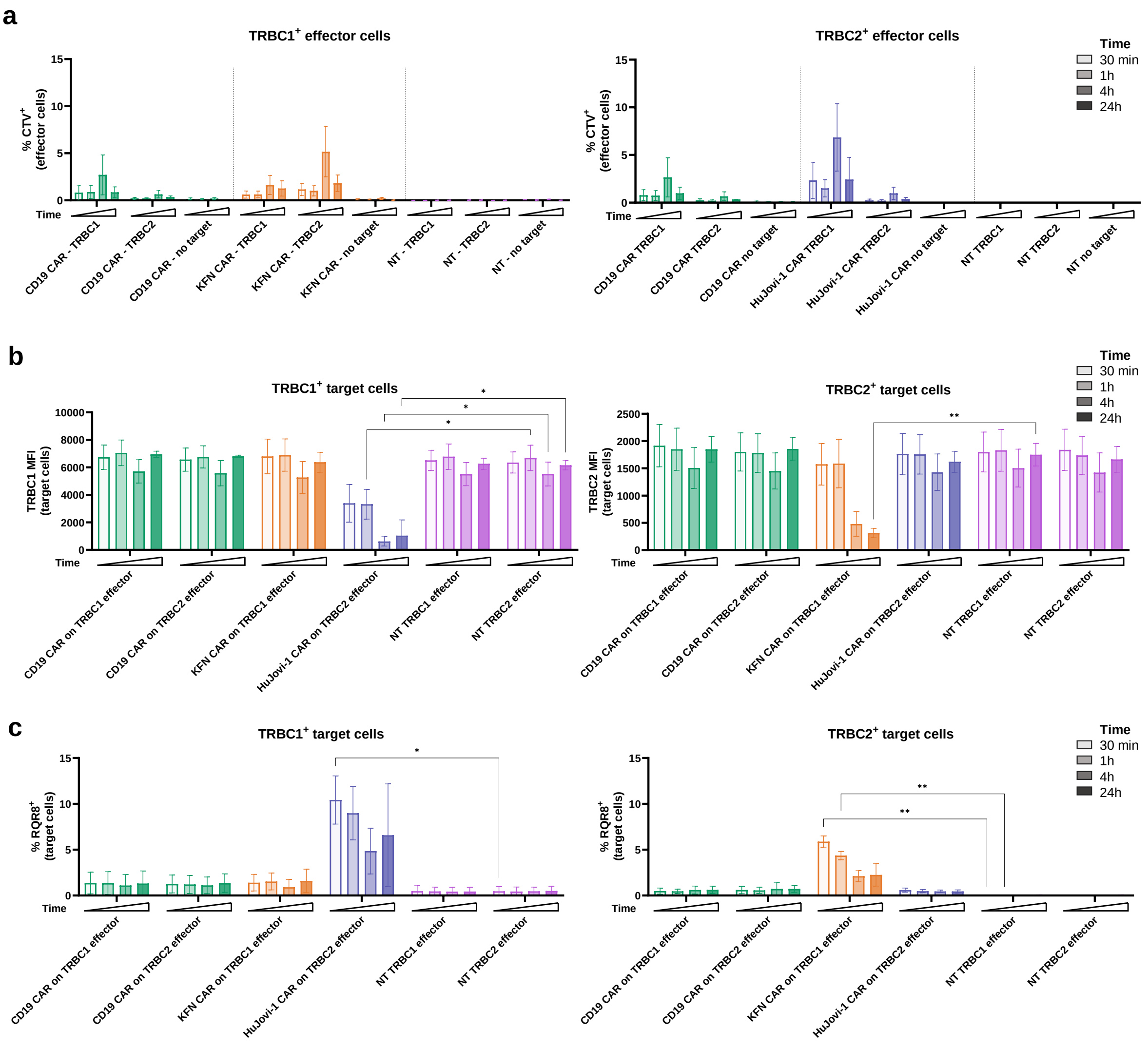

a, Co-culture of CD19 CAR (green), KFN CAR (orange) and NT PBMC (purple) on TRBC1 effector cells (left) or CD19 CAR (green), HuJovi-1 CAR (Blue) and non-transduced (NT) PBMC (purple) on TRBC2 effector cells (right) with CellTrace Violet (CTV) labelled TRBC1$^+$, TRBC2$^+$ PBMC or in the absence of target cells. Graphs show CTV uptake by effector cells following 30 minutes, 1h, 4h or 24h co-culture with target cells (1:1 E:T ratio, n = 3 biologically independent samples). Data shown as mean ± SD. **b,** Co-culture of CD19 CAR (green), KFN CAR (orange), HuJovi-1 CAR (blue) or NT PBMC (purple) with TRBC1$^+$ (left) or TRBC2$^+$ (right) target cells. Graphs show TRBC1 (left) or TRBC2 (right) downregulation from TRBC1$^+$ or TRBC2$^+$ target cells following 30 minutes, 1h, 4h or 24h co-culture with HuJovi-1 CAR or KFN CAR, respectively (1:1 E:T ratio, n = 3 biologically independent samples). Data shown as mean ± SD. Two-way ANOVA with Dunnett's post-test against NT effector cells, * $p < 0.05$, ** $p < 0.01$. **c,** Co-culture of CD19 CAR (green), KFN CAR (orange), HuJovi-1 CAR (blue) or NT PBMC (purple) with TRBC1$^+$ (left) or TRBC2$^+$ (right) target cells. Graphs show transduction marker RQR8 uptake by target cells following 30 minutes, 1h, 4h or 24h co-culture with effector cells (1:1 E:T ratio, n = 3 biologically independent samples).

Data shown as mean ± SD. Two-way ANOVA with Dunnett's post-test against NT effector cells, * $p < 0.05$, ** $p < 0.01$. Source data and exact p-values are provided as a Source Data file.

Supplementary Fig. 18. In vitro CAR-T profiling

a

b

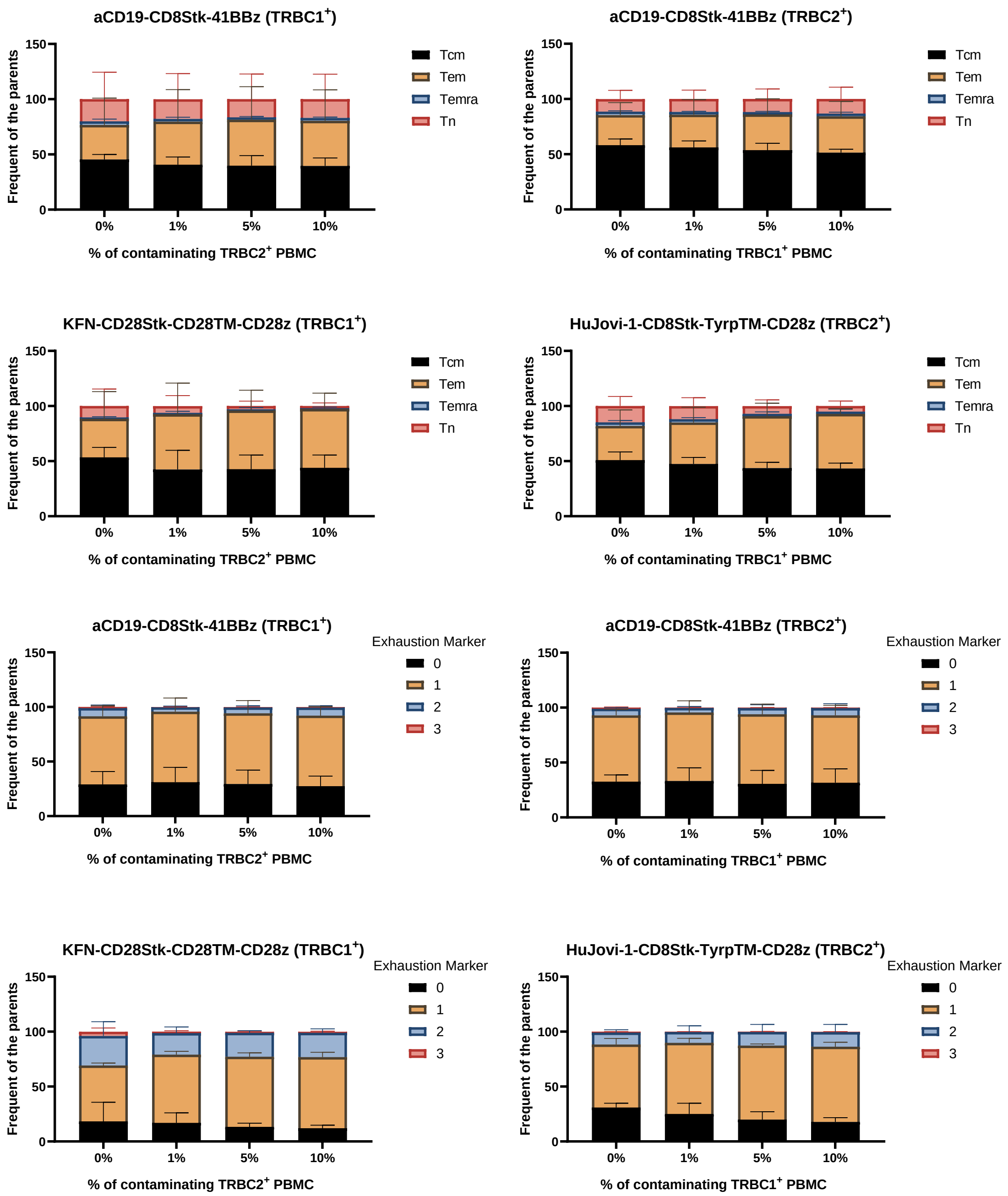

a, Differentiation profile of CAR-T cells for sorted TRBC1^{+} PBMC (KFN CAR and aCD19 CAR) and TRBC2^{+} PBMC (HuJovi-1 CAR and aCD19 CAR), incubated for 6 days with 0, 1, 5 and 10% contaminating TRBC2^{+} PBMC (for KFN CAR and aCD19 CAR) or TRBC1^{+} PBMC (for HuJovi-1 CAR and aCD19 CAR). Data shown as mean ± SD. Tcm = T central memory, Tem = T effector memory, Temra = terminally differentiated effector memory cells, Tn = naïve T cell. n = 3 biologically independent samples. **b**, Exhaustion profile of CAR T

cells for sorted TRBC1^{+} PBMC (KFN CAR and aCD19 CAR) and TRBC2^{+} PBMC (HuJovi-1 CAR and aCD19 CAR) based on the expression of 0, 1, 2 or 3 antigens between PD-1, TIM3 and CXCR5. Cells were incubated for 6 days with 0, 1, 5 or 10% contaminating TRBC2^{+} PBMC (for KFN CAR and aCD19 CAR) or TRBC1^{+} PBMC (for HuJovi-1 CAR and aCD19 CAR). n = 3 biologically independent samples. Data shown as mean ± SD. Source data provided as a Source Data file.

Supplementary Fig. 19. Bioluminescent imaging for NSG HPB-ALL *in vivo* model

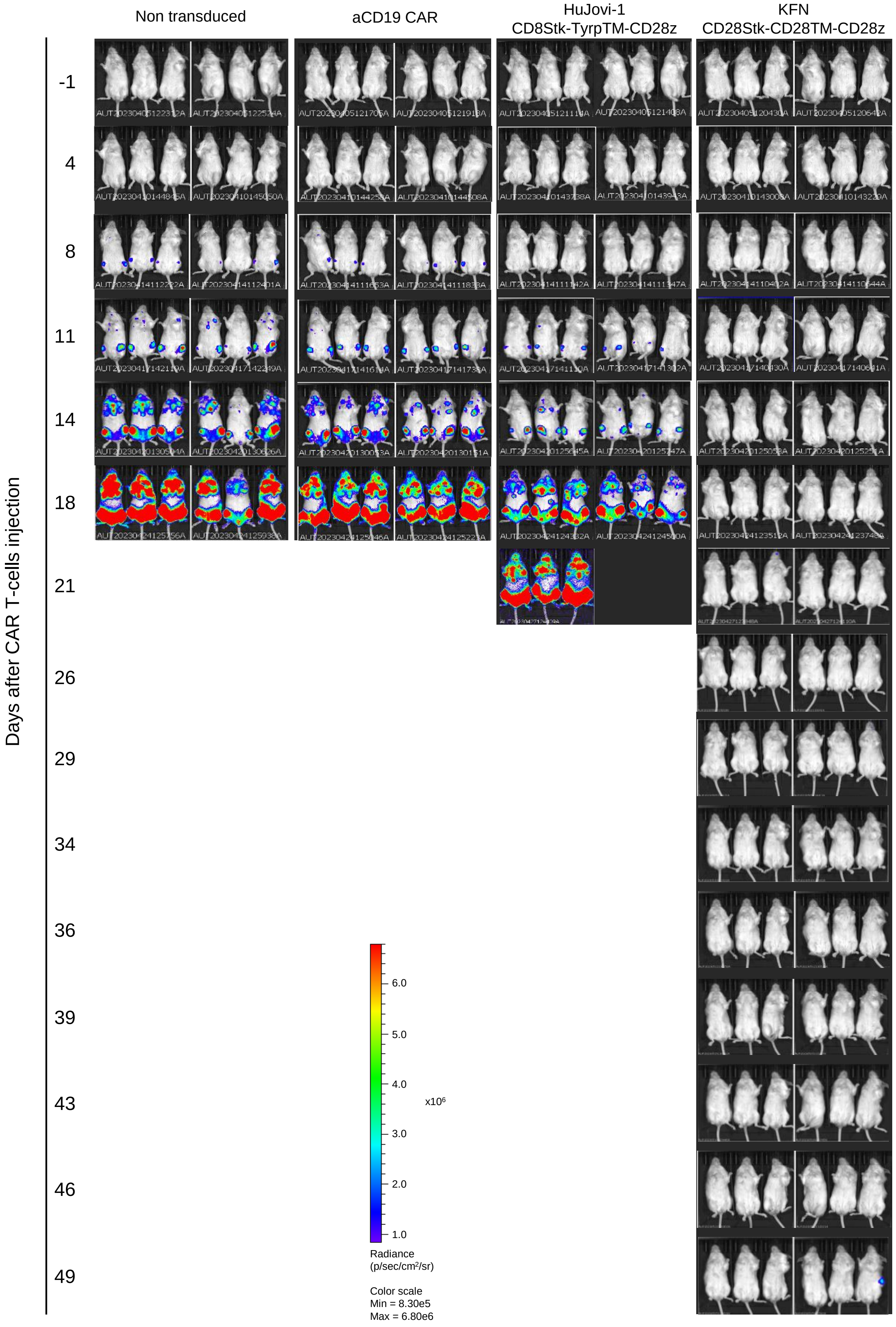

Total radiance bioluminescent imaging (BLI) of NSG mice engrafted with HPB-ALL TRBC2 cell line and treated with KFN-CD28Stk-CD28TM-CD28z, HuJovi1-CD8Stk-TyrpTM-CD28z, aCD19 CAR T cells or non-transduced PBMC, at 5e6 CAR T cells/mouse.

Supplementary Fig. 20. Analysis of NSG HPB-ALL *in vivo* model

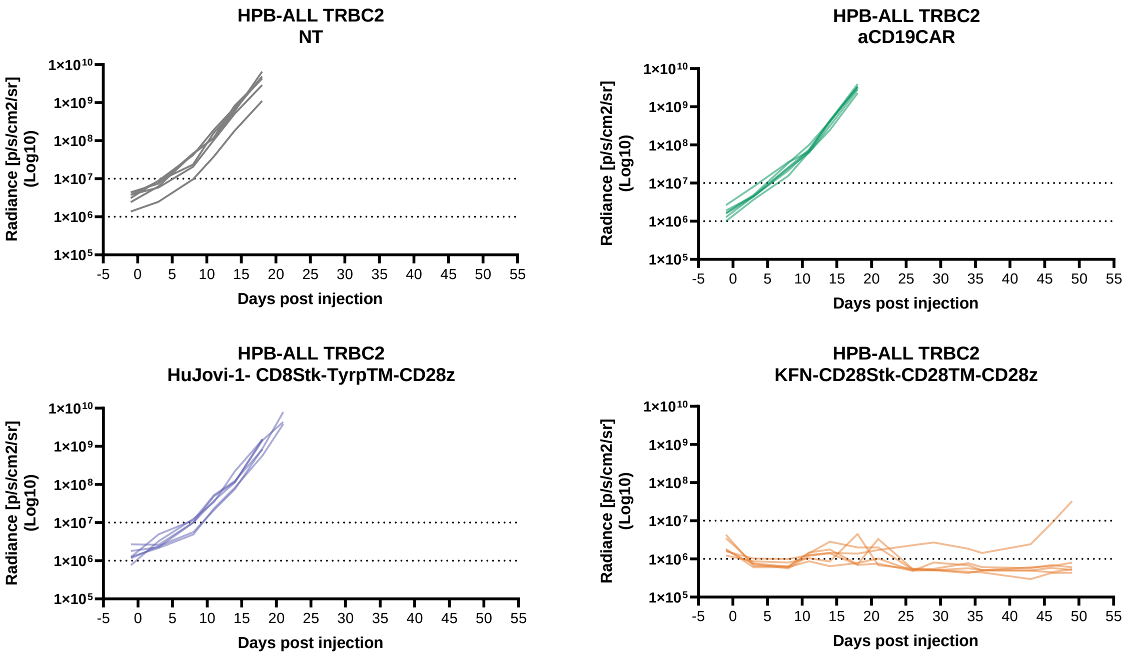

Individual mouse total radiance BLI of HPB-ALL TRBC2^{+} tumor burden post CAR-T cells (n = 6). Source data provided as a Source Data file.

Supplementary Fig. 21. Bioluminescent imaging for NSG Jurkat TRBC1/TRBC2 *in vivo* model

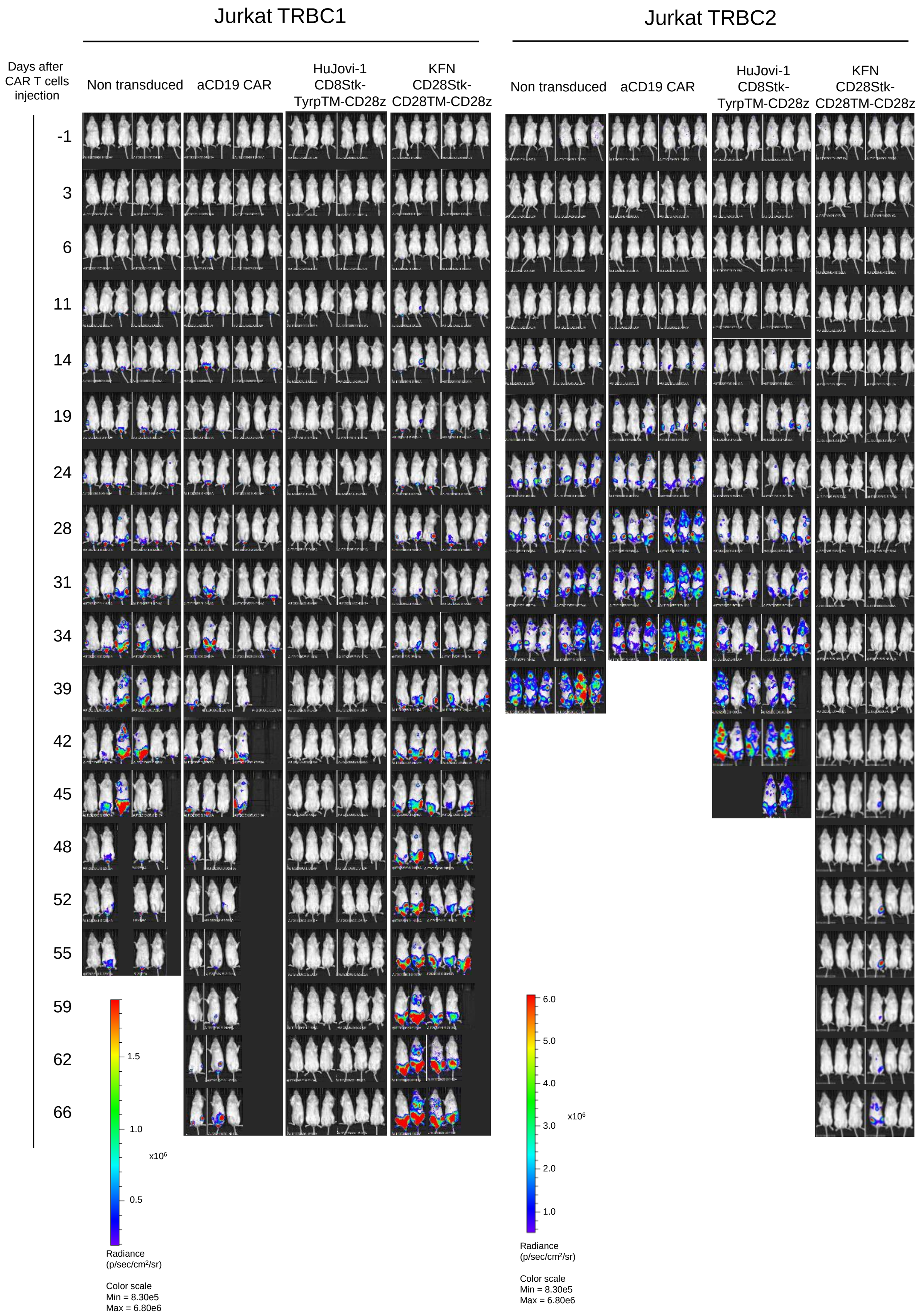

Total radiance BLI of NSG mice engrafted with Jurkat TRBC1 (left) or Jurkat TRBC2 (right) cell lines and treated with KFN-CD28Stk-CD28TM-CD28z, HuJovi1-CD8stk-TyrpTM-CD28z, aCD19 CAR-T cells or non-transduced PBMC, at 5e6 CAR T cells/mouse.

Supplementary Fig. 22. Analysis of NSG Jurkat TRBC1/TRBC2 *in vivo* model

a

NSG - Jurkat TRBC1

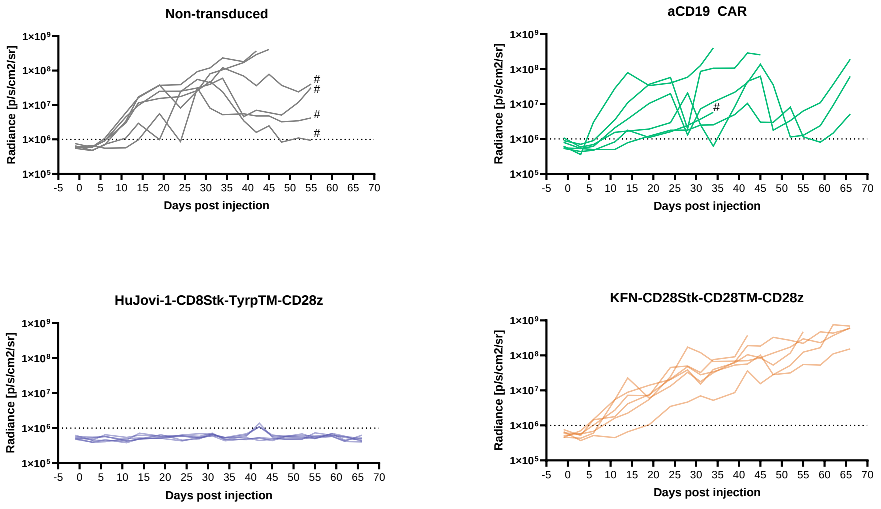

b

NSG - Jurkat TRBC2

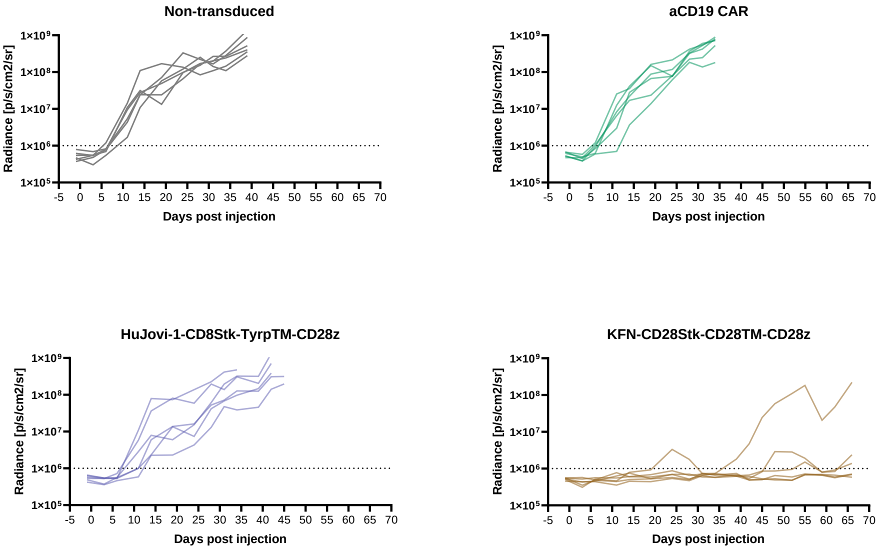

a, Individual mouse total radiance BLI of Jurkat TRBC1$^+$ tumor burden post CAR-T cells (n = 6). # xeno-GvHD event. **b**, Individual mouse total radiance BLI of Jurkat TRBC2$^+$ tumor burden post CAR-T cells (n = 6). Source data are provided as a Source Data file.

Supplementary Tables

Supplementary Table 1: Antibody sequences.

Clone	Molecule	Chain	Sequence
MuJovi-1	Protein	Heavy chain	EVRLQQSGPDLIKPGASVKMSCKASGYTFTGYVMHWVKQRPGQGLEWIGF<br>INPYNDDIQSNERFRGKATLTSDKSSTTAYMELSSLTSEDSAVYYCARGAG<br>YNFDGAYRFFDFWGQGTTLTVSSASTKGPSVFPLAPSSKSTSGGTAALGCL<br>VKDYFPEPVTVSWNSGALTSGVHTFPAVLQSSGLYSLSSVVTVPSSSLGTQT<br>YICNVNHKPSNTKVDKKVEPKSCDKTHTCPPCPAPELLGGPSVFLFPPKPKD<br>TLMISRTPEVTCVVVDVSHEDPEVKFNWYVDGVEVHNAKTKPREEQYNST<br>YRVVSVLTVLHQDWLNGKEYKCKVSNKALPAPIEKTISKAKGQPREPQVY<br>TLPPSRDELTKNQVSLTCLVKGFYPSDIAVEWESNGQPENNYKTTPPVLDSD<br>GSFFLYSKLTVDKSRWQQGNVFSCSVMHEALHNHYTQKSLSLSPGK
		Light chain	DVVMTQSPLSLPVSLGDQASISCRSSQRLVHSNGNTYLHWYLQKPGQSPKL<br>LIYRVSNRFPGVPDRFSGSGSGTDFTLKISRVEAEDLGIYFCSQSTHVPYTFG<br>GGTKLEIKRTVAAPSVFIFPPSDEQLKSGTASVVCLLNNFYPREAKVQWKV<br>DNALQSGNSQESVTEQDSKDSTYSLSSTLTLSKADYEKHKVYACEVTHQGL<br>SSPVTKSFNRGEC
	DNA	Heavy chain	GAGGTGCGGCTGCAGCAGAGCGGCCCTGACCTGATCAAGCCCGGCGCC<br>AGCGTGAAGATGAGCTGCAAGGCCAGCGGCTACACCTTCACCGGCTAC<br>GTGATGCACTGGGTGAAGCAGCGGCCTGGCCAGGGCCTGGAGTGGATC<br>GGCTTCATCAACCCTTACAACGACGACATCCAGAGCAACGAGCGGTTCC<br>GCGGCAAGGCCACCCTGACCAGCGACAAGAGCAGCACCACCGCCTACA<br>TGGAGCTGAGCAGCCTGACCAGCGAGGACAGCGCCGTGTACTACTGCG<br>CCCGCGGAGCCGGCTACAACTTCGACGGCGCCTACCGGTTCTTCGACTT<br>CTGGGGCCAGGGCACCACCCTGACCGTGAGCTCAgcgTCGACCAAGGGC<br>CCATCGGTCTTCCCCCTGGCACCCTCCTCCAAGAGCACCTCTGGGGGCA<br>CAGCGGCCCTGGGCTGCCTGGTCAAGGACTACTTCCCCGAACCTGTGAC<br>GGTCTCGTGGAACTCAGGCGCCCTGACCAGCGGCGTGCACACCTTCCCG<br>GCTGTCCTACAGTCCTCAGGACTCTACTCCCTCAGCAGCGTGGTGACCG<br>TGCCCTCCAGCAGCTTGGGCACCCAGACCTACATCTGCAACGTGAATCA<br>CAAGCCCAGCAACACCAAGGTGGACAAGAAAGTTGAGCCCAAATCTTG

			TGACAAAACTCACACATGCCCACCGTGCCCAGCACCTGAACTCCTGGGG GGACCGTCAGTCTTCCTCTTCCCCCCAAAACCCAAGGACACCCTCATGA TCTCCCGGACCCCTGAGGTCACATGCGTGGTGGTGGACGTGAGCCACGA AGACCCTGAGGTCAAGTTCAACTGGTACGTGGACGGCGTGGAGGTGCAT AATGCCAAGACAAAGCCGCGGGAGGAGCAGTACAACAGCACGTACCGT GTGGTCAGCGTCCTCACCGTCCTGCACCAGGACTGGCTGAATGGCAAGG AGTACAAGTGCAAGGTCTCCAACAAAGCCCTCCCAGCCCCCATCGAGAA AACCATCTCCAAAGCCAAAGGGCAGCCCCGAGAACCACAGGTGTACAC CCTGCCCCCATCCCGGGATGAGCTGACCAAGAACCAGGTCAGCCTGACC TGCCTGGTCAAAGGCTTCTATCCCAGCGACATCGCCGTGGAGTGGGAGA GCAATGGGCAGCCGGAGAACAACTACAAGACCACGCCTCCCGTGCTGG ACTCCGACGGCTCCTTCTTCCTCTACAGCAAGCTCACCGTGGACAAGAG CAGGTGGCAGCAGGGGAACGTCTTCTCATGCTCCGTGATGCATGAGGCT CTGCACAACCACTACACGCAGAAGAGCCTCTCCCTGTCTCCGGGTAAA
		Light chain	GACGTGGTGATGACCCAGAGCCCACTGAGCCTGCCCGTGAGCCTGGGCG ACCAGGCCAGCATCAGCTGCCGGAGCAGCCAGCGGCTGGTGCACAGCA ACGGCAACACCTACCTGCACTGGTACCTGCAGAAGCCCGGCCAGAGCCC TAAGCTGCTGATCTACCGGGTGAGCAACCGGTTCCCTGGCGTGCCCGAC CGGTTCAGCGGCAGCGGCAGCGGCACCGACTTCACCCTGAAGATCAGCC GGGTGGAGGCCGAGGACCTGGGCATCTACTTCTGCAGCCAGAGCACCC ACGTGCCCTACACCTTCGGAGGCGGCACCAAGCTGGAGATCAAGCGGA CGGTGGCTGCACCATCTGTCTTCATCTTCCCGCCATCTGATGAGCAGTTG AAATCTGGAACTGCCTCTGTTGTGTGCCTGCTGAATAACTTCTATCCCAG AGAGGCCAAAGTACAGTGGAAGGTGGATAACGCCCTCCAATCGGGTAA CTCCCAGGAGAGTGTCACAGAGCAGGACAGCAAGGACAGCACCTACAG CCTCAGCAGCACCCTGACGCTGAGCAAAGCAGACTACGAGAAACACAA AGTCTACGCCTGCGAAGTCACCCATCAGGGCCTGAGCTCGCCCGTCACA AAGAGCTTCAACAGGGGAGAGTGT
HuJovi-1	Protein	Heavy chain	QVQLVQSGAEVKKPGASVKVSCKASGYTFTGYVMHWVRQAPGQGLEWM GFINPYNDDIQSNERFRGRVTMTRDTSISTAYMELSRLRSDDTAVYYCARG AGYNFDGAYRFFDFWGQGTMVTVSSASTKGPSVFPLAPSSKSTSGGTAALG CLVKDYFPEPVTVSWNSGALTSGVHTFPAVLQSSGLYSLSSVVTVPSSSLGT QTYICNVNHKPSNTKVDKKVEPKSCDKTHTCPPCPAPELLGGPSVFLFPPKP KDTLMISRTPEVTCVVVDVSHEDPEVKFNWYVDGVEVHNAKTKPREEQYN STYRVVSVLTVLHQDWLNGKEYKCKVSNKALPAPIEKTISKAKGQPREPQV

			YTLPPSRDELTKNQVSLTCLVKGFYPSDIAVEWESNGQPENNYKTTPPVLDS<br>DGSFFLYSKLTVDKSRWQQGNVFSCSVMHEALHNHYTQKSLSLSPGK
		Light chain	DIVMTQSPLSLPVTPGEPASISCRSSQRLVHSNGNTYLHWYLQKPGQSPRLLI<br>YRVSNRFPGVPDRFSGSGSGTDFTLKISRVEAEDVGVYYCSQSTHVPYTFG<br>QGTKLEIKRTVAAPSVFIFPPSDEQLKSGTASVVCLLNNFYPREAKVQWKV<br>DNALQSGNSQESVTEQDSKDSTYSLSSTLTLSKADYEKHKVYACEVTHQGL<br>SSPVTKSFNRGEC
	DNA	Heavy chain	CAGGTGCAGCTGGTGCAGTCTGGCGCCGAAGTGAAGAAACCAGGCGCC<br>AGCGTGAAGGTGTCCTGCAAGGCCAGCGGCTACACCTTTACCGGCTACG<br>TGATGCACTGGGTGCGCCAGGCTCCAGGCCAGGGACTGGAATGGATGG<br>GCTTCATCAACCCCTACAACGACGACATCCAGAGCAACGAGCGGTTCCG<br>GGGCAGAGTGACCATGACCAGAGACACCAGCATCAGCACCGCCTACAT<br>GGAACTGAGCCGGCTGAGAAGCGACGACACCGCCGTGTACTACTGCGC<br>CAGAGGCGCCGGATACAACTTCGACGGCGCCTACAGATTCTTCGACTTC<br>TGGGGCCAGGGCACAATGGTCACCGTGTCCTCTGCGTCGACCAAGGGCC<br>CATCGGTCTTCCCCCTGGCACCCTCCTCCAAGAGCACCTCTGGGGGCAC<br>AGCGGCCCTGGGCTGCCTGGTCAAGGACTACTTCCCCGAACCTGTGACG<br>GTCTCGTGGAACTCAGGCGCCCTGACCAGCGGCGTGCACACCTTCCCGG<br>CTGTCCTACAGTCCTCAGGACTCTACTCCCTCAGCAGCGTGGTGACCGT<br>GCCCTCCAGCAGCTTGGGCACCCAGACCTACATCTGCAACGTGAATCAC<br>AAGCCCAGCAACACCAAGGTGGACAAGAAAGTTGAGCCCAAATCTTGT<br>GACAAAACTCACACATGCCCACCGTGCCCAGCACCTGAACTCCTGGGGG<br>GACCGTCAGTCTTCCTCTTCCCCCCAAAACCCAAGGACACCCTCATGAT<br>CTCCCGGACCCCTGAGGTCACATGCGTGGTGGTGGACGTGAGCCACGAA<br>GACCCTGAGGTCAAGTTCAACTGGTACGTGGACGGCGTGGAGGTGCATA<br>ATGCCAAGACAAAGCCGCGGGAGGAGCAGTACAACAGCACGTACCGTG<br>TGGTCAGCGTCCTCACCGTCCTGCACCAGGACTGGCTGAATGGCAAGGA<br>GTACAAGTGCAAGGTCTCCAACAAAGCCCTCCCAGCCCCCATCGAGAAA<br>ACCATCTCCAAAGCCAAAGGGCAGCCCCGAGAACCACAGGTGTACACC<br>CTGCCCCCATCCCGGGATGAGCTGACCAAGAACCAGGTCAGCCTGACCT<br>GCCTGGTCAAAGGCTTCTATCCCAGCGACATCGCCGTGGAGTGGGAGAG<br>CAATGGGCAGCCGGAGAACAACTACAAGACCACGCCTCCCGTGCTGGA<br>CTCCGACGGCTCCTTCTTCCTCTACAGCAAGCTCACCGTGGACAAGAGC<br>AGGTGGCAGCAGGGGAACGTCTTCTCATGCTCCGTGATGCATGAGGCTC<br>TGCACAACCACTACACGCAGAAGAGCCTCTCCCTGTCTCCGGGTAAA

		Light chain	GACATCGTGATGACCCAGAGCCCCCTGAGCCTGCCTGTGACACCTGGCGAACCTGCCAGCATCAGCTGCCGGTCTAGCCAGAGACTGGTGCACAGCAACGGCAACACCTACCTGCACTGGTATCTGCAGAAGCCCGGCCAGTCCCCCAGACTGCTGATCTACCGGGTGTCCAACAGATTCCCCGGCGTGCCCGATAGATTCAGCGGCTCTGGCAGCGGCACCGACTTCACCCTGAAGATCTCCCGGGTGGAAGCCGAGGACGTGGGCGTGTACTACTGCAGCCAGAGCACCCACGTGCCCTACACCTTTGGCCAGGGCACCAAGCTGGAAATCAAGCGTACGGTGGCTGCACCATCTGTCTTCATCTTCCCGCCATCTGATGAGCAGTTGAAATCTGGAACTGCCTCTGTTGTGTGCCTGCTGAATAACTTCTATCCCAGAGAGGCCAAAGTACAGTGGAAGGTGGATAACGCCCTCCAATCGGGTAACTCCCAGGAGAGTGTCACAGAGCAGGACAGCAAGGACAGCACCTACAGCCTCAGCAGCACCCTGACGCTGAGCAAAGCAGACTACGAGAAACACAAAGTCTACGCCTGCGAAGTCACCCATCAGGGCCTGAGCTCGCCCGTCACAAAGAGCTTCAACAGGGGAGAGTGT
KFN (Mut3)	Protein	Heavy chain	QVQLVQSGAEVKKPGASVKVSCKASGYKFTGFVMHWVRQAPGQGLEWMGFINPYNDDIQSNERFRGRVTMTRDTSISTAYMELSRLRSDDTAVYYCARGNGYNFDGAYRFFDFWGQGTMVTVSSASTKGPSVFPLAPSSKSTSGGTAALGCLVKDYFPEPVTVSWNSGALTSGVHTFPAVLQSSGLYSLSSVVTVPSSSLGTQTYICNVNHKPSNTKVDKKVEPKSCDKTHTCPPCPAPELLGGPSVFLFPPKPKDTLMISRTPEVTCVVVDVSHEDPEVKFNWYVDGVEVHNAKTKPREEQYNSTYRVVSVLTVLHQDWLNGKEYKCKVSNKALPAPIEKTISKAKGQPREPQVYTLPPSRDELTKNQVSLTCLVKGFYPSDIAVEWESNGQPENNYKTTPPVLDSDGSFFLYSKLTVDKSRWQQGNVFSCSVMHEALHNHYTQKSLSLSPGK
		Light chain	DIVMTQSPLSLPVTPGEPASISCRSSQRLVHSNGNTYLHWYLQKPGQSPRLLIYRVSNRFPGVPDRFSGSGSGTDFTLKISRVEAEDVGVYYCSQSTHVPYTFGQGTKLEIKRTVAAPSVFIFPPSDEQLKSGTASVVCLLNNFYPREAKVQWKVDNALQSGNSQESVTEQDSKDSTYSLSSTLTLSKADYEKHKVYACEVTHQGLSSPVTKSFNRGEC
	DNA	Heavy chain	CAGGTGCAGCTGGTGCAGTCTGGCGCCGAAGTGAAGAAACCAGGCGCCAGCGTGAAGGTGTCCTGCAAGGCCAGCGGCTACaagTTTACCGGCtttGTGATGCACTGGGTGCGCCAGGCTCCAGGCCAGGGACTGGAATGGATGGGCTTCATCAACCCCTACAACGACGACATCCAGAGCAACGAGCGGTTCCGGGGCAGAGTGACCATGACCAGAGACACCAGCATCAGCACCGCCTACATGGAACTGAGCCGGCTGAGAAGCGACGACACCGCCGTGTACTACTGCGCCAGAGGCAACGGATACAACTTCGACGGCGCCTACAGATTCTTCGACTTCTGGGGCCAGGGCACAATGGTCACCGTGTCCTCTGCGTCGACCAAGGGCCCA

			TCGGTCTTCCCCCTGGCACCCTCCTCCAAGAGCACCTCTGGGGGCACAG<br>CGGCCCTGGGCTGCCTGGTCAAGGACTACTTCCCCGAACCTGTGACGGT<br>CTCGTGGAACTCAGGCGCCCTGACCAGCGGCGTGCACACCTTCCCGGCT<br>GTCCTACAGTCCTCAGGACTCTACTCCCTCAGCAGCGTGGTGACCGTGC<br>CCTCCAGCAGCTTGGGCACCCAGACCTACATCTGCAACGTGAATCACAA<br>GCCCAGCAACACCAAGGTGGACAAGAAAGTTGAGCCCAAATCTTGTGA<br>CAAAACTCACACATGCCCACCGTGCCCAGCACCTGAACTCCTGGGGGGA<br>CCGTCAGTCTTCCTCTTCCCCCCAAAACCCAAGGACACCCTCATGATCTC<br>CCGGACCCCTGAGGTCACATGCGTGGTGGTGGACGTGAGCCACGAAGA<br>CCCTGAGGTCAAGTTCAACTGGTACGTGGACGGCGTGGAGGTGCATAAT<br>GCCAAGACAAAGCCGCGGGAGGAGCAGTACAACAGCACGTACCGTGTG<br>GTCAGCGTCCTCACCGTCCTGCACCAGGACTGGCTGAATGGCAAGGAGT<br>ACAAGTGCAAGGTCTCCAACAAAGCCCTCCCAGCCCCCATCGAGAAAA<br>CCATCTCCAAAGCCAAAGGGCAGCCCCGAGAACCACAGGTGTACACCC<br>TGCCCCCATCCCGGGATGAGCTGACCAAGAACCAGGTCAGCCTGACCTG<br>CCTGGTCAAAGGCTTCTATCCCAGCGACATCGCCGTGGAGTGGGAGAGC<br>AATGGGCAGCCGGAGAACAACTACAAGACCACGCCTCCCGTGCTGGAC<br>TCCGACGGCTCCTTCTTCCTCTACAGCAAGCTCACCGTGGACAAGAGCA<br>GGTGGCAGCAGGGGAACGTCTTCTCATGCTCCGTGATGCATGAGGCTCT<br>GCACAACCACTACACGCAGAAGAGCCTCTCCCTGTCTCCGGGTAAA
		Light chain	GACATCGTGATGACCCAGAGCCCCCTGAGCCTGCCTGTGACACCTGGCG<br>AACCTGCCAGCATCAGCTGCCGGTCTAGCCAGAGACTGGTGCACAGCAA<br>CGGCAACACCTACCTGCACTGGTATCTGCAGAAGCCCGGCCAGTCCCCC<br>AGACTGCTGATCTACCGGGTGTCCAACAGATTCCCCGGCGTGCCCGATA<br>GATTCAGCGGCTCTGGCAGCGGCACCGACTTCACCCTGAAGATCTCCCG<br>GGTGGAAGCCGAGGACGTGGGCGTGTACTACTGCAGCCAGAGCACCCA<br>CGTGCCCTACACCTTTGGCCAGGGCACCAAGCTGGAAATCAAGCGTACG<br>GTGGCTGCACCATCTGTCTTCATCTTCCCGCCATCTGATGAGCAGTTGAA<br>ATCTGGAACTGCCTCTGTTGTGTGCCTGCTGAATAACTTCTATCCCAGAG<br>AGGCCAAAGTACAGTGGAAGGTGGATAACGCCCTCCAATCGGGTAACT<br>CCCAGGAGAGTGTCACAGAGCAGGACAGCAAGGACAGCACCTACAGCC<br>TCAGCAGCACCCTGACGCTGAGCAAAGCAGACTACGAGAAACACAAAG<br>TCTACGCCTGCGAAGTCACCCATCAGGGCCTGAGCTCGCCCGTCACAAA<br>GAGCTTCAACAGGGGAGAGTGT
RFN (Mut4)	Protein	Heavy chain	QVQLVQSGAEVKKPGASVKVSCKASGYRFTGFVMHWVRQAPGQGLEWM<br>GFINPYNDDIQSNERFRGRVTMTRDTSISTAYMELSRLRSDDTAVYYCARG<br>NGYNFDGAYRFFDFWGQGTMVTVSSASTKGPSVFPLAPSSKSTSGGTAALG

			CLVKDYFPEPVTVSWNSGALTSGVHTFPAVLQSSGLYSLSSVVTVPSSSLGT QTYICNVNHKPSNTKVDKKVEPKSCDKTHTCPPCPAPELLGGPSVFLFPPKP KDTLMISRTPEVTCVVVDVSHEDPEVKFNWYVDGVEVHNAKTKPREEQYN STYRVVSVLTVLHQDWLNGKEYKCKVSNKALPAPIEKTISKAKGQPREPQV YTLPPSRDELTKNQVSLTCLVKGFYPSDIAVEWESNGQPENNYKTTPPVLDS DGSFFLYSKLTVDKSRWQQGNVFSCSVMHEALHNHYTQKSLSLSPGK
		Light chain	DIVMTQSPLSLPVTPGEPASISCRSSQRLVHSNGNTYLHWYLQKPGQSPRLLI YRVSNRFPGVPDRFSGSGSGTDFTLKISRVEAEDVGVYYCSQSTHVPYTFG QGTKLEIKRTVAAPSVFIFPPSDEQLKSGTASVVCLLNNFYPREAKVQWKV DNALQSGNSQESVTEQDSKDSTYSLSSTLTLSKADYEKHKVYACEVTHQGL SSPVTKSFNRGEC
	DNA	Heavy chain	CAGGTGCAGCTGGTGCAGTCTGGCGCCGAAGTGAAGAAACCAGGCGCC AGCGTGAAGGTGTCCTGCAAGGCCAGCGGCTACCGCTTTACCGGCTTTG TGATGCACTGGGTGCGCCAGGCTCCAGGCCAGGGACTGGAATGGATGG GCTTCATCAACCCCTACAACGACGACATCCAGAGCAACGAGCGGTTCCG GGGCAGAGTGACCATGACCAGAGACACCAGCATCAGCACCGCCTACAT GGAACTGAGCCGGCTGAGAAGCGACGACACCGCCGTGTACTACTGCGC CAGAGGCAACGGATACAACTTCGACGGCGCCTACAGATTCTTCGACTTC TGGGGCCAGGGCACAATGGTCACCGTGTCCTCCGCGTCGACCAAGGGCC CATCGGTCTTCCCCCTGGCACCCTCCTCCAAGAGCACCTCTGGGGGCAC AGCGGCCCTGGGCTGCCTGGTCAAGGACTACTTCCCCGAACCTGTGACG GTCTCGTGGAACTCAGGCGCCCTGACCAGCGGCGTGCACACCTTCCCGG CTGTCCTACAGTCCTCAGGACTCTACTCCCTCAGCAGCGTGGTGACCGT GCCCTCCAGCAGCTTGGGCACCCAGACCTACATCTGCAACGTGAATCAC AAGCCCAGCAACACCAAGGTGGACAAGAAAGTTGAGCCCAAATCTTGT GACAAAACTCACACATGCCCACCGTGCCCAGCACCTGAACTCCTGGGGG GACCGTCAGTCTTCCTCTTCCCCCCAAAACCCAAGGACACCCTCATGAT CTCCCGGACCCCTGAGGTCACATGCGTGGTGGTGGACGTGAGCCACGAA GACCCTGAGGTCAAGTTCAACTGGTACGTGGACGGCGTGGAGGTGCATA ATGCCAAGACAAAGCCGCGGGAGGAGCAGTACAACAGCACGTACCGTG TGGTCAGCGTCCTCACCGTCCTGCACCAGGACTGGCTGAATGGCAAGGA GTACAAGTGCAAGGTCTCCAACAAAGCCCTCCCAGCCCCCATCGAGAAA ACCATCTCCAAAGCCAAAGGGCAGCCCCGAGAACCACAGGTGTACACC CTGCCCCCATCCCGGGATGAGCTGACCAAGAACCAGGTCAGCCTGACCT GCCTGGTCAAAGGCTTCTATCCCAGCGACATCGCCGTGGAGTGGGAGAG CAATGGGCAGCCGGAGAACAACTACAAGACCACGCCTCCCGTGCTGGA CTCCGACGGCTCCTTCTTCCTCTACAGCAAGCTCACCGTGGACAAGAGC

			AGGTGGCAGCAGGGGAACGTCTTCTCATGCTCCGTGATGCATGAGGCTC TGCACAACCACTACACGCAGAAGAGCCTCTCCCTGTCTCCGGGTAAA
		Light chain	GACATCGTGATGACCCAGAGCCCCCTGAGCCTGCCTGTGACACCTGGCG AACCTGCCAGCATCAGCTGCCGGTCTAGCCAGAGACTGGTGCACAGCAA CGGCAACACCTACCTGCACTGGTATCTGCAGAAGCCCGGCCAGTCCCCC AGACTGCTGATCTACCGGGTGTCCAACAGATTCCCCGGCGTGCCCGATA GATTCAGCGGCTCTGGCAGCGGCACCGACTTCACCCTGAAGATCTCCCG GGTGGAAGCCGAGGACGTGGGCGTGTACTACTGCAGCCAGAGCACCCA CGTGCCCTACACCTTTGGCCAGGGCACCAAGCTGGAAATCAAGCGTACG GTGGCTGCACCATCTGTCTTCATCTTCCCGCCATCTGATGAGCAGTTGAA ATCTGGAACTGCCTCTGTTGTGTGCCTGCTGAATAACTTCTATCCCAGAG AGGCCAAAGTACAGTGGAAGGTGGATAACGCCCTCCAATCGGGTAACT CCCAGGAGAGTGTCACAGAGCAGGACAGCAAGGACAGCACCTACAGCC TCAGCAGCACCCTGACGCTGAGCAAAGCAGACTACGAGAAACACAAAG TCTACGCCTGCGAAGTCACCCATCAGGGCCTGAGCTCGCCCGTCACAAA GAGCTTCAACAGGGGAGAGTGT

Supplementary Table 2: X-ray data collection and refinement statistics.

	**HuJovi-1/TRBC1 (7AMP)**	**HuJovi-1/TRBC2 (7AMQ)**	**KFN/TRBC2 (7AMS)**	**KFN/TRBC1 (7AMR)**
Resolution (Å)	54.44–2.64 (2.69–2.64)	47.8–2.35 (2.43–2.35)	43.4–2.42 (2.46–2.42)	45.7–1.95 (2.13–1.95)‡
Wavelength (Å)	0.9282	0.9795	0.9795	0.9762
Space group	C2	C2	C2	C2
Unit cell (Å)	a=117.4<br>b=91.1<br>c=125.3<br>β=93.8	a = 114.5<br>b = 91.8<br>c = 123.4<br>β=94.6	a = 114.8<br>b = 91.2<br>c = 123.3<br>β=94.1	a = 115.7<br>b = 91.0<br>c = 123.3<br>β=93.9
Completeness (%)	99.9 (99.8)	99.6 (96.8)	99.9 (97.8)	ellipsoidal: 93.8 (87.8)<br>spherical: 53.7 (11.6)
Redundancy	6.1 (6.3)	5.1 (4.3)	5.0 (5.1)	5.7 (4.9)
No. of observations/unique reflections	237,232 (38,805)	267,467 (52,746)	242,685 (48,522)	287,266 (49,996)
<I/σ(I)>	9.9 (0.93)	8.3 (1.0)	7.0 (1.1)	10.4 (1.5)
CC(1/2) (%)	99.8 (62.5)	99.6 (71.2)	99.0 (71.3)	99.6 (52.4)
R_{merge}(I) (%)	11.8 (166.9)	13.2 (122.8)	17.5 (165.9)	12.5 (101.8)
R_{model}(F) R_{free}(F) (%)	23.9/28.9 (47.4)/(46.9)	21.6/25.6 (34.9)/(35.2)	19.9/24.6 (31.4)/(44.3)	17.9/22.5 (28.6)/(21.7)
No. of non-hydrogen atoms	6,858	7,287	7,382	7,539
No. of water molecules	20	502	557	683
Rms deviations from ideal geometry: bond lengths (Å)	0.007	0.007	0.007	0.10
Bond angles (degrees)	1.16	1.00	0.98	1.19
Mean B-factor all atoms (Å^2)*	85.1	56.3	47.6	32.7
Mean B-factor protein chains A,B,H,L (Å^2)*	81.8, 85.6, 87.7, 85.1	60.3, 57.0, 54.7, 51.6	53.7, 49.9, 45.4, 43.0	37.5, 34.2, 30.2, 27.6
Mean B-factor (solvent Å^2)*	54.6	57.8	47.6	37.4
Mean B-factor others (Å^2)	79.6	66.4	56.6	41.4
**Ramachandran plot quality#**				
Favored regions (%)	94.4	98.0	97.1	97.4
Allowed regions (%)	5.3	1.8	2.7	2.3
Outliers (%)	0.3	0.2	0.2	0.3

Figures in parentheses are for the highest resolution shell. Other relevant quality indicators can be easily extracted from the PDB file header. *Calculated with Moleman[1]; #calculated using a local Molprobity server[2]; ‡processed anisotropically with autoPROC/STARANISO. The anisotropic resolution limits were 1.85 Å, 2.3 Å, and 3.0 Å in the three principal directions of the anisotropic ellipsoid. When processed isotropically, the data gave a resolution of 2.56 Å with 100% completeness.

Supplementary Table 3: *In silico* mutagenesis of HuJovi-1 binding to TRBC2.

Residue	Original	Mutated	δ Affinity TRBC2	δ Affinity TRBC1	Net δ Affinity
H:28	THR	LYS	-13.32	-3.35	-9.97
H:28	THR	ARG	-12.41	-2.3	-10.11
H:96	ALA	ASN	-11.73	-0.62	-11.11
H:99	ASN	ARG	-11.21	-2.41	-8.8
H:35	HIE	TRP	-11.2	-3.42	-7.78
H:28	THR	HIP	-10.93	-15.51	4.58
H:99	ASN	TYR	-7.98	0.09	-8.07
H:100F	PHE	ARG	-7.93	-19.61	11.68
H:100A	ASP	ASN	-6.61	-2.24	-4.37
H:98	TYR	ARG	-6.27	1.33	-7.6
H:60	ASN	HIP	-5.77	-1.1	-4.67
H:100F	PHE	LYS	-5.08	-7.18	2.1
H:58	GLN	LYS	-4.98	0.39	-5.37
H:96	ALA	GLN	-4.95	-2.42	-2.53
H:31	GLY	MET	-4.94	1.71	-6.65
H:100F	PHE	HIP	-4.89	-8.78	3.89
H:99	ASN	GLN	-4.87	0.23	-5.1
H:100A	ASP	LEU	-4.65	-1.32	-3.33
H:31	GLY	LYS	-4.64	-1.4	-3.24
H:100G	PHE	ARG	-4.53	-1.56	-2.97
H:28	THR	MET	-4.31	4.25	-8.56
H:101	ASP	MET	-4.18	-6.36	2.18
H:28	THR	ILE	-4.15	3.96	-8.11
H:95	GLY	HIP	-4.11	-1.54	-2.57
H:60	ASN	TRP	-4.1	-1.1	-3
H:98	TYR	HIP	-4.07	-5.07	1
H:100B	GLY	PHE	-4.04	-3.27	-0.77
H:100B	GLY	ARG	-4.02	-2.18	-1.84
H:99	ASN	MET	-3.77	-0.1	-3.67
H:31	GLY	HID	-3.54	0.97	-4.51
H:52	ASN	PHE	-3.49	-0.39	-3.1
H:96	ALA	THR	-3.42	-1.81	-1.61
H:28	THR	TRP	-3.36	-1.02	-2.34
H:101	ASP	ILE	-3.3	-4.42	1.12
H:28	THR	TYR	-3.23	-0.33	-2.9
H:100A	ASP	THR	-3.2	-2.86	-0.34
H:95	GLY	ARG	-3.08	12.27	-15.35
H:96	ALA	HID	-3.05	-3.33	0.28

In silico identification of Hu-Jovi-1 variants with highest affinity increase to TRBC2. Three color grading: Red = high δ affinity to TRBC2 or low δ affinity to TRBC1. Green = high δ

affinity to TRBC1 or low δ affinity to TRBC2. Net δ affinity = difference between δ affinity to TRBC2 and δ affinity to TRBC1. Kabat numbering.

Supplementary References

1. Kleywegt, G. J. & Jones, T. A. Phi/Psi-chology: Ramachandran revisited. *Structure* **4**, 1395–1400 (1996).

2. Chen, V. B. *et al. MolProbity* : all-atom structure validation for macromolecular crystallography. *Acta Crystallogr.* *D Biol. Crystallogr.* **66**, 12–21 (2010).
