# Supplementary material for: Structure-guided engineering of immunotherapies targeting TRBC1 and TRBC2 in T cell malignancies
Source: Nat Commun. 2024 Feb 21;15:1583. doi: 10.1038/s41467-024-45854-3 (PMC10881500; doi:10.1038/s41467-024-45854-3)
Supplement: Supplementary file 2 — Reporting Summary [file 41467_2024_45854_MOESM2_ESM.pdf]

nature portfolio

Corresponding author(s): Martin Pule

Last updated by author(s): Jan 19, 2024

Reporting Summary

Nature Portfolio wishes to improve the reproducibility of the work that we publish. This form provides structure for consistency and transparency in reporting. For further information on Nature Portfolio policies, see our Editorial Policies and the Editorial Policy Checklist.

Statistics

For all statistical analyses, confirm that the following items are present in the figure legend, table legend, main text, or Methods section.

n/a	Confirmed	
☐	☒	The exact sample size (n) for each experimental group/condition, given as a discrete number and unit of measurement
☐	☒	A statement on whether measurements were taken from distinct samples or whether the same sample was measured repeatedly
☐	☒	The statistical test(s) used AND whether they are one- or two-sided<br>*Only common tests should be described solely by name; describe more complex techniques in the Methods section.*
☐	☒	A description of all covariates tested
☐	☒	A description of any assumptions or corrections, such as tests of normality and adjustment for multiple comparisons
☐	☒	A full description of the statistical parameters including central tendency (e.g. means) or other basic estimates (e.g. regression coefficient) AND variation (e.g. standard deviation) or associated estimates of uncertainty (e.g. confidence intervals)
☐	☒	For null hypothesis testing, the test statistic (e.g. F, t, r) with confidence intervals, effect sizes, degrees of freedom and P value noted<br>*Give P values as exact values whenever suitable.*
☒	☐	For Bayesian analysis, information on the choice of priors and Markov chain Monte Carlo settings
☒	☐	For hierarchical and complex designs, identification of the appropriate level for tests and full reporting of outcomes
☒	☐	Estimates of effect sizes (e.g. Cohen's d, Pearson's r), indicating how they were calculated

Our web collection on statistics for biologists contains articles on many of the points above.

Software and code

Policy information about availability of computer code

Data collection	Protein structure data obtained at Diamond Light Source (Didcot, England).<br>ELISA data acquired using Varioskan instrument (ThermoFisher Scientific)<br>Flow cytometry data collected using MACSQuant Analyzer 10 (Miltenyi, RRID:SCR_020268)<br>Surface plasmon resonance data acquired using Biacore T200 (Cytiva Lifesciences, RRID:SCR_019718)
Data analysis	For TRBC1/HuJovi1 protein structure, data were processed using XIA2 pipeline (Winter et. al., 2010). For HuJovi-1/TRBC2, the data were processed using XDS (Kabsch et al., 2010) and Aimless (Otwinowski and Minor et al., 1997). For KFN/TRBC1, the data were processed using autoPROC (Vonrhein et al. 2011) and anisotropically truncated using STARANISO (Tickle et al. 2018). For KFN/TRBC2, the data were processed using XDS (Kabsch et al., 2010) and the XIA2-dials pipeline (Winter et al., 2010). Protein modelling and in-silico analysis was performed using the BioLuminate suite (Schrodinger).<br>ELISA data was analysed using GraphPad Prism v9 (GraphPad Software Inc., RRID:SCR_000306).<br>Flow Cytometry data was analysed using FlowJo software v10 (Treestar, RRID: SCR_008520).<br>Surface plasmon resonance data was analysed using Biacore Insight Evaluation Software (Cytiva Lifesciences, RRID:SCR_015936) adopting a 1:1 Langmuir binding model.<br>Statistical analysis performed using GraphPad Prism v9 (GraphPad Software Inc., RRID:SCR_000306).

For manuscripts utilizing custom algorithms or software that are central to the research but not yet described in published literature, software must be made available to editors and reviewers. We strongly encourage code deposition in a community repository (e.g. GitHub). See the Nature Portfolio guidelines for submitting code & software for further information.

Data

Policy information about availability of data

All manuscripts must include a data availability statement. This statement should provide the following information, where applicable:

- Accession codes, unique identifiers, or web links for publicly available datasets
- A description of any restrictions on data availability
- For clinical datasets or third party data, please ensure that the statement adheres to our policy

All data is available in the manuscript or the supplementary information. Crystal structure co-ordinates have been deposited at the protein data bank (PDB) with the following ID numbers: 7AMP, 7AMQ, 7AMR and 7AMS.

Research involving human participants, their data, or biological material

Policy information about studies with human participants or human data. See also policy information about sex, gender (identity/presentation), and sexual orientation and race, ethnicity and racism.

Reporting on sex and gender	Sex and gender of the Healthy volunteers donors was not shared due to NHSBT applying the Data Protection Act 2018 and UK General Data Protection Regulation (GDPR). Patients with T-cell malignancies were anonymised with demographic data not available to researchers (University of Cologne).
Reporting on race, ethnicity, or other socially relevant groupings	Race, ethnicity of the Healthy volunteers donors was not shared due to NHSBT applying the Data Protection Act 2018 and UK General Data Protection Regulation (GDPR). Patients with T-cell malignancies were anonymised with demographic data not available to researchers (University of Cologne).
Population characteristics	Healthy volunteers donors and patients with T-PLL malignancy.
Recruitment	Healthy volunteers donors were purchased from National Health Service Blood and Transplant (NHSBT, UK). Patients with T-cell malignancies were recruited within the T-PLL registry NCT 028636692 (anonymised with demographic data not available to researchers) and sample use approved for research purposes by the local ethics committee (University of Cologne #11-319)
Ethics oversight	Samples were collected, processed and analysed according to Autolus procedures (according to Human Tissue Authority licence 12642, UK) and University of Cologne ethics.

Note that full information on the approval of the study protocol must also be provided in the manuscript.

Field-specific reporting

Please select the one below that is the best fit for your research. If you are not sure, read the appropriate sections before making your selection.

☒ Life sciences ☐ Behavioural & social sciences ☐ Ecological, evolutionary & environmental sciences

For a reference copy of the document with all sections, see nature.com/documents/nr-reporting-summary-flat.pdf

Life sciences study design

All studies must disclose on these points even when the disclosure is negative.

Sample size	Cohort sizes were based on number required to demonstrate 90% reduction in tumour bioluminescence, 95% confidence with 80% power.
Data exclusions	No exclusions were pre-specified or included for the survival or specificity studies.<br>For one in vivo experiment (Jurkat TRBC1 and Jurkat TRBC2 NSG model), bone marrow samples were excluded from analysis due to insufficient cell recovery (xeno-GvHD)
Replication	In vitro: All CAR T cell data are reported as replicates using biologically independent PBMC donors. Sample size is specified in the figure legend and Methods section. In vivo: experiments were performed once for each of the Jurkat TRBC1, Jurkat TRBC2, HPB-ALL TRBC1 and HPB-ALL TRBC2 mouse models reported, using 6 animals per group.
Randomization	All mice were scanned to measure BLI counts on D-1 (related to CAR T-cell injection) and ranked in order of total flux, after which cohorts are randomly selected with a similar total group BLI average.
Blinding	Blinding was not performed as differences between control/ treated mice were so marked that this would have been futile, and manpower issues did not allow this.

Reporting for specific materials, systems and methods

We require information from authors about some types of materials, experimental systems and methods used in many studies. Here, indicate whether each material, system or method listed is relevant to your study. If you are not sure if a list item applies to your research, read the appropriate section before selecting a response.

Materials & experimental systems

n/a	Involved in the study
	☒ Antibodies
	☒ Eukaryotic cell lines
☒	Palaeontology and archaeology
	☒ Animals and other organisms
☒	Clinical data
☒	Dual use research of concern
☒	Plants

Methods

n/a	Involved in the study
☒	ChIP-seq
	☒ Flow cytometry
☒	MRI-based neuroimaging

Antibodies

Antibodies used

Primary antibodies were used for the target, species and application (flow cytometry) identified by the manufacturer. Research Resource Identifiers (RRIDs) were obtained from The Antibody Registry (antibodyregistry.org):
HLA-DR (Cat# 564040, BD Biosciences RRID:AB_2738558, Clone G46-6, 1:250)
CD8 (Cat# 612942, Becton Dickinson, RRID:AB_2870223, Clone RPA-T8, 1:1000)
CD27 (Cat# 741833, Becton Dickinson, RRID:AB_2871168, Clone M-T271, 1:500)
CD4 (Cat# 612887, Becton Dickinson, RRID:AB_2870176, Clone SK3, 1:250)
CD45RA (Cat# 566114, Becton Dickinson, RRID:AB_2739516, Clone HI100, 1:1000)
CD57 (Cat# 393304, Biolegend, RRID:AB_2728425, Clone QA17A04, 1:1000)
CD62L (Cat# 563808, Becton Dickinson, RRID:AB_2738433, Clone DREG-56, 1:500)
TIM3 (Cat# 565566, Becton Dickinson, RRID:AB_2744370, Clone 7D3, 1:1000)
CD25 (Cat# 563701, Becton Dickinson, RRID:AB_2744338, Clone M-A251, 1:500)
CFSE (Cat# C34554, ThermoFisher, 1:1000)
TIGIT (Cat# 46-9500-42, Invitrogen, RRID:AB_10853679, Clone MBSA43, 1:1000)
CCR7 (Cat# 353236, Biolegend, RRID:AB_2563640, Clone G043H7, 1:1000)
PD-1 (Cat# 561272, Becton Dickinson, RRID:AB_10611585, Clone N/A, 1:1000)
LAG3 (Cat# 369304, Biolegend, RRID:AB_2566480, Clone 11C3C65, 1:500)
FVS780 (Cat# 565388, Becton Dickinson, 1:1000)
Jovi1 (Cat# ANC-101-030, Ancell, lone JOVI-1, 1:1000)
Humanized Jovi 1 and variants were in house generated
Anti-idiotype for Jovi-1 was in house generated
aCD3 (Cat# 317334, Biolegend, RRID:AB_2561452, Clone OKT3, 1:1000)
Streptavidin (Cat# 405243, Biolegend, 1:1000)
Anti-CD34 (Cat# 343607, Biolegend, RRID:AB_2074356, Clone 561, 1:200)
anti TCR alpha/beta Monoclonal Antibody (WT31) PE conjugated (Cat# 12-9955-42, eBioscience, RRID:AB_10596819, Clone WT31, 1:1000)
Goat anti-Human IgG (H+L) Cross-Adsorbed Secondary Antibody, Alexa Fluor™ 647 (Cat# A-21445, Invitrogen, RRID:AB_2535862, Clone N/A, 1:1000)
Goat anti-Mouse IgG2a Cross-Adsorbed Secondary Antibody, Alexa Fluor™ 488 (Cat# A-21131, Invitrogen, RRID:AB_2535771, Clone N/A, 1:1000)
HuAnti-CD34 (Cat# FAB7227A, R&D System, RRID:AB_10972777, Clone QBend10, 1:200)
Anti CD3 (Milteny, Cat# 130-113-142, RRID:AB_2725970, Clone REA613, 1:500)
Anti CD45RA (Mylteny, Cat# 130-117-747, RRID:AB_2733126, Clone REA1047, 1:500)
Anti CCR7 (Miltenyi, Cat# 130-119-583, RRID:AB_2751741, Clone REA546, 1:500)
Anti CXCR5 (Miltenyi, Cat# 130-122-795, RRID:AB_2811386, Clone REA103, 1:500)
Anti TIM-3 (Miltenyi, Cat# 130-121-334, RRID:AB_2784165, Clone REA635, 1:500)

Validation

The antibodies used in this study are commercially available and were used for the applications validated by manufacturers. Validation statements can be found on manufacturer websites.

Eukaryotic cell lines

Policy information about cell lines and Sex and Gender in Research

Cell line source(s)

HEK-293T, Jurkat TRBC1+, H9, T-ALL1 were obtained from the American Type Culture Collection. HD-MAR and HPB-ALL were obtained from DSMZ-German Collection of Microorganisms and Cell Cultures.

Authentication

Cell lines were obtained from cell bank repositories. Engineered cell lines were validated by flow cytometric stainings

Mycoplasma contamination

Cell lines were tested negative for mycoplasma contamination

Commonly misidentified lines
(See ICLAC register)

No commonly misidentified cell lines were used in the study.

Animals and other research organisms

Policy information about studies involving animals; ARRIVE guidelines recommended for reporting animal research, and Sex and Gender in Research

Laboratory animals	NSG mice (NOD scid gamma; NOD.Cg-PrkdcscidIl2rgtm1Wjl/SzJ) of 14 weeks of age (Charles River, 614NSG).<br>The enclosure used were IVC (Individually Ventilated Cage) and the respective sizes follows Table 1.1 of Annex III to Directive 2010/63/EU. The mice followed a 12-hour light-dark cycle. According to UK Home Office guidelines, the room temperature was kept stable at 20–24°C (68–75°F), and sufficient nesting material was provided to enables mice to create good nests, which can reach temperatures of 30–32°C (86–90°F). The relative humidity was kept at 45 to 65%, with the number of air changes per hour kept between 25 and 120. Food and water were provided ad libitum.
Wild animals	No wild animals were used in the study.
Reporting on sex	Female mice were used
Field-collected samples	No field collected samples were used in the study.
Ethics oversight	All procedures in this study gained the approval of The Animal Welfare and Ethical Review Body and the United Kingdom Home Office (Autolus PPL No. PP7328369). All procedures are performed in accordance with the United Kingdom Home Office Animals (Scientific Procedures) Act 1986 and in adherence to Imperial College London or Autolus PPL (PP7328369).

Note that full information on the approval of the study protocol must also be provided in the manuscript.

Flow Cytometry

Plots

Confirm that:

☒ The axis labels state the marker and fluorochrome used (e.g. CD4-FITC).

☒ The axis scales are clearly visible. Include numbers along axes only for bottom left plot of group (a 'group' is an analysis of identical markers).

☒ All plots are contour plots with outliers or pseudocolor plots.

☒ A numerical value for number of cells or percentage (with statistics) is provided.

Methodology

Sample preparation	Specific sample preparation procedures are reported in Materials and methods. Primary T cells were isolated from leucocyte cones of healthy donors via Ficoll gradient separation. Bone marrow cells were collected by centrifugation of lower limb bones.
Instrument	MacsQuant10 Miltenyi
Software	FlowJo v10 (Treestar, RRID:SCR_008520)
Cell population abundance	For live target and effector cells, data was reported as % relative to co-culture conditions with non-transduced PBMC.
Gating strategy	Gating strategies are reported in Supplementary material.<br>Generally, cells were first gated for lymphocyte population based on SSC-A and FSC-A. Singlets were identified by FSC-H and FSC-A. Live cells were identified using a viability die (7AAD or Sytox blue)

☒ Tick this box to confirm that a figure exemplifying the gating strategy is provided in the Supplementary Information.
