# Supplementary material for: Structure-guided engineering of immunotherapies targeting TRBC1 and TRBC2 in T cell malignancies
Source: Nat Commun. 2024 Feb 21;15:1583. doi: 10.1038/s41467-024-45854-3 (PMC10881500; doi:10.1038/s41467-024-45854-3)

Plate1_KFN_vs_Jurkat_TRBC1

Well	Target	E:T ratio	Donor	CAR construct
**1B**	Jurkat TRBC 1+ve	1:4	Donor 1	KFN_Hinge_28z
**1D**	Jurkat TRBC 1+ve	1:4	Donor 1	KFN_CD8STK_28z
**1E**	Jurkat TRBC 1+ve	1:4	Donor 1	KFN_CD28STK_CD28TM_28z
**1F**	Jurkat TRBC 1+ve	1:4	Donor 1	aCD19-CAR
**1G**	Jurkat TRBC 1+ve	1:4	Donor 1	Non-transduced
**1H**	Jurkat TRBC 1+ve	N/A	N/A	N/A
**2B**	Jurkat TRBC 1+ve	1:8	Donor 1	KFN_Hinge_28z
**2D**	Jurkat TRBC 1+ve	1:8	Donor 1	KFN_CD8STK_28z
**2E**	Jurkat TRBC 1+ve	1:8	Donor 1	KFN_CD28STK_CD28TM_28z
**2F**	Jurkat TRBC 1+ve	1:8	Donor 1	aCD19-CAR
**2G**	Jurkat TRBC 1+ve	1:8	Donor 1	Non-transduced
**2H**	Jurkat TRBC 1+ve	N/A	N/A	N/A
**5B**	Jurkat TRBC 1+ve	1:4	Donor 2	KFN_Hinge_28z
**5D**	Jurkat TRBC 1+ve	1:4	Donor 2	KFN_CD8STK_28z
**5E**	Jurkat TRBC 1+ve	1:4	Donor 2	KFN_CD28STK_CD28TM_28z
**5F**	Jurkat TRBC 1+ve	1:4	Donor 2	aCD19-CAR
**5G**	Jurkat TRBC 1+ve	1:4	Donor 2	Non-transduced
**5H**	Jurkat TRBC 1+ve	N/A	N/A	N/A
**6B**	Jurkat TRBC 1+ve	1:8	Donor 2	KFN_Hinge_28z
**6D**	Jurkat TRBC 1+ve	1:8	Donor 2	KFN_CD8STK_28z
**6E**	Jurkat TRBC 1+ve	1:8	Donor 2	KFN_CD28STK_CD28TM_28z
**6F**	Jurkat TRBC 1+ve	1:8	Donor 2	aCD19-CAR
**6G**	Jurkat TRBC 1+ve	1:8	Donor 2	Non-transduced
**6H**	Jurkat TRBC 1+ve	N/A	N/A	N/A
**9B**	Jurkat TRBC 1+ve	1:4	Donor 3	KFN_Hinge_28z
**9D**	Jurkat TRBC 1+ve	1:4	Donor 3	KFN_CD8STK_28z
**9E**	Jurkat TRBC 1+ve	1:4	Donor 3	KFN_CD28STK_CD28TM_28z
**9F**	Jurkat TRBC 1+ve	1:4	Donor 3	aCD19-CAR
**9G**	Jurkat TRBC 1+ve	1:4	Donor 3	Non-transduced
**9H**	Jurkat TRBC 1+ve	N/A	N/A	N/A
**10B**	Jurkat TRBC 1+ve	1:8	Donor 3	KFN_Hinge_28z
**10D**	Jurkat TRBC 1+ve	1:8	Donor 3	KFN_CD8STK_28z
**10E**	Jurkat TRBC 1+ve	1:8	Donor 3	KFN_CD28STK_CD28TM_28z
**10F**	Jurkat TRBC 1+ve	1:8	Donor 3	aCD19-CAR
**10G**	Jurkat TRBC 1+ve	1:8	Donor 3	Non-transduced
**10H**	Jurkat TRBC 1+ve	N/A	N/A	N/A

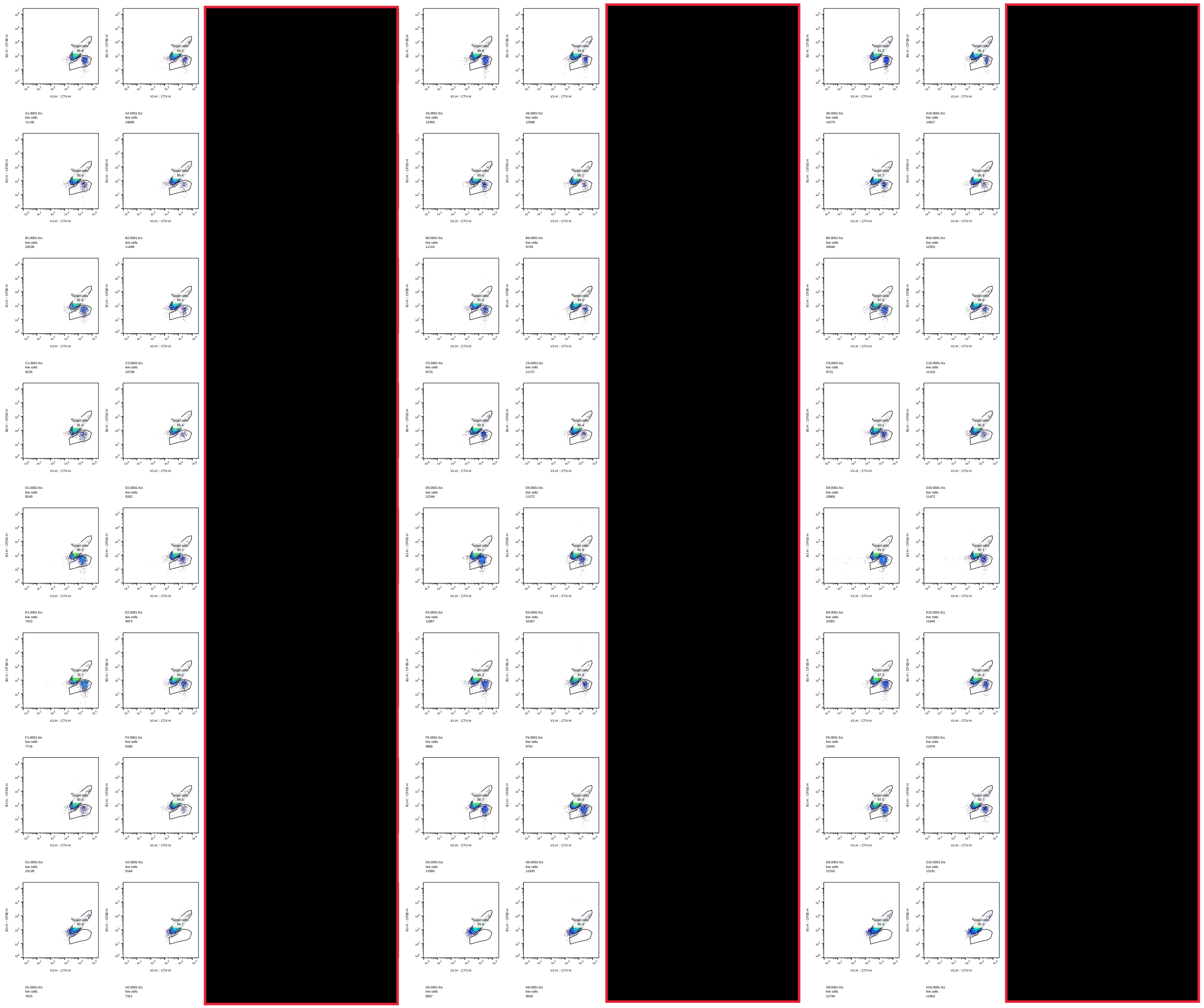

Plate2_KFN_vs_Jurkat_TRBC2

Well	Target	E:T ratio	Donor	CAR construct
**1B**	Jurkat TRBC 2+ve	1:4	Donor 1	KFN_Hinge_28z
**1D**	Jurkat TRBC 2+ve	1:4	Donor 1	KFN_CD8STK_28z
**1E**	Jurkat TRBC 2+ve	1:4	Donor 1	KFN_CD28STK_CD28TM_28z
**1F**	Jurkat TRBC 2+ve	1:4	Donor 1	aCD19-CAR
**1G**	Jurkat TRBC 2+ve	1:4	Donor 1	Non-transduced
**1H**	Jurkat TRBC 2+ve	N/A	N/A	N/A
**2B**	Jurkat TRBC 2+ve	1:8	Donor 1	KFN_Hinge_28z
**2D**	Jurkat TRBC 2+ve	1:8	Donor 1	KFN_CD8STK_28z
**2E**	Jurkat TRBC 2+ve	1:8	Donor 1	KFN_CD28STK_CD28TM_28z
**2F**	Jurkat TRBC 2+ve	1:8	Donor 1	aCD19-CAR
**2G**	Jurkat TRBC 2+ve	1:8	Donor 1	Non-transduced
**2H**	Jurkat TRBC 2+ve	N/A	N/A	N/A
**5B**	Jurkat TRBC 2+ve	1:4	Donor 2	KFN_Hinge_28z
**5D**	Jurkat TRBC 2+ve	1:4	Donor 2	KFN_CD8STK_28z
**5E**	Jurkat TRBC 2+ve	1:4	Donor 2	KFN_CD28STK_CD28TM_28z
**5F**	Jurkat TRBC 2+ve	1:4	Donor 2	aCD19-CAR
**5G**	Jurkat TRBC 2+ve	1:4	Donor 2	Non-transduced
**5H**	Jurkat TRBC 2+ve	N/A	N/A	N/A
**6B**	Jurkat TRBC 2+ve	1:8	Donor 2	KFN_Hinge_28z
**6D**	Jurkat TRBC 2+ve	1:8	Donor 2	KFN_CD8STK_28z
**6E**	Jurkat TRBC 2+ve	1:8	Donor 2	KFN_CD28STK_CD28TM_28z
**6F**	Jurkat TRBC 2+ve	1:8	Donor 2	aCD19-CAR
**6G**	Jurkat TRBC 2+ve	1:8	Donor 2	Non-transduced
**6H**	Jurkat TRBC 2+ve	N/A	N/A	N/A
**9B**	Jurkat TRBC 2+ve	1:4	Donor 3	KFN_Hinge_28z
**9D**	Jurkat TRBC 2+ve	1:4	Donor 3	KFN_CD8STK_28z
**9E**	Jurkat TRBC 2+ve	1:4	Donor 3	KFN_CD28STK_CD28TM_28z
**9F**	Jurkat TRBC 2+ve	1:4	Donor 3	aCD19-CAR
**9G**	Jurkat TRBC 2+ve	1:4	Donor 3	Non-transduced
**9H**	Jurkat TRBC 2+ve	N/A	N/A	N/A
**10B**	Jurkat TRBC 2+ve	1:8	Donor 3	KFN_Hinge_28z
**10D**	Jurkat TRBC 2+ve	1:8	Donor 3	KFN_CD8STK_28z
**10E**	Jurkat TRBC 2+ve	1:8	Donor 3	KFN_CD28STK_CD28TM_28z
**10F**	Jurkat TRBC 2+ve	1:8	Donor 3	aCD19-CAR
**10G**	Jurkat TRBC 2+ve	1:8	Donor 3	Non-transduced
**10H**	Jurkat TRBC 2+ve	N/A	N/A	N/A

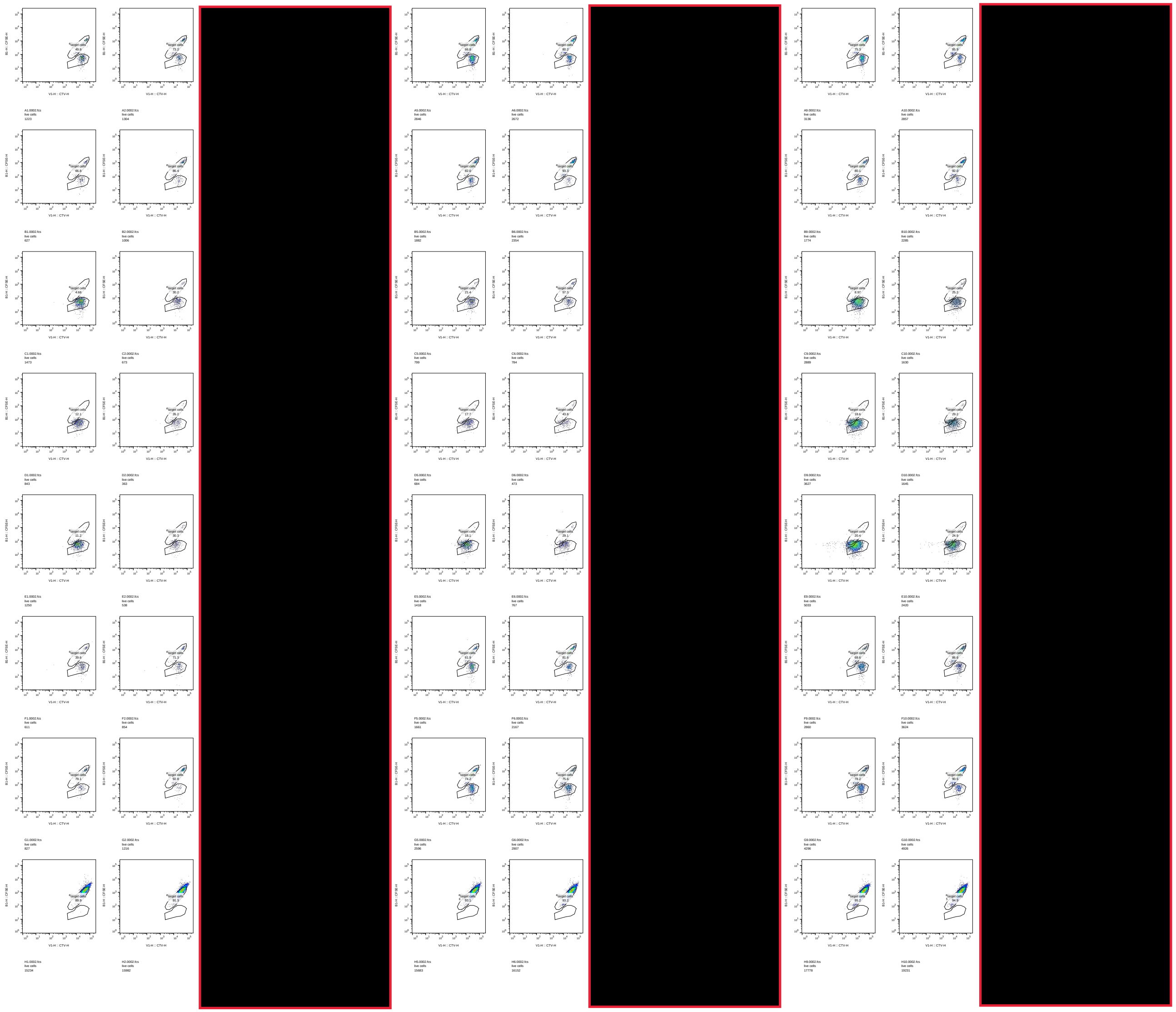

Plate3_KFN_vs_Jurkat_TRBC-KO

Well	Target	E:T ratio	Donor	CAR construct
1B	Jurkat TRBC KO	1:4	Donor 1	KFN_Hinge_28z
1D	Jurkat TRBC KO	1:4	Donor 1	KFN_CD8STK_28z
1E	Jurkat TRBC KO	1:4	Donor 1	KFN_CD28STK_CD28TM_28z
1F	Jurkat TRBC KO	1:4	Donor 1	aCD19-CAR
1G	Jurkat TRBC KO	1:4	Donor 1	Non-transduced
1H	Jurkat TRBC KO	N/A	N/A	N/A
2B	Jurkat TRBC KO	1:8	Donor 1	KFN_Hinge_28z
2D	Jurkat TRBC KO	1:8	Donor 1	KFN_CD8STK_28z
2E	Jurkat TRBC KO	1:8	Donor 1	KFN_CD28STK_CD28TM_28z
2F	Jurkat TRBC KO	1:8	Donor 1	aCD19-CAR
2G	Jurkat TRBC KO	1:8	Donor 1	Non-transduced
2H	Jurkat TRBC KO	N/A	N/A	N/A
5B	Jurkat TRBC KO	1:4	Donor 2	KFN_Hinge_28z
5D	Jurkat TRBC KO	1:4	Donor 2	KFN_CD8STK_28z
5E	Jurkat TRBC KO	1:4	Donor 2	KFN_CD28STK_CD28TM_28z
5F	Jurkat TRBC KO	1:4	Donor 2	aCD19-CAR
5G	Jurkat TRBC KO	1:4	Donor 2	Non-transduced
5H	Jurkat TRBC KO	N/A	N/A	N/A
6B	Jurkat TRBC KO	1:8	Donor 2	KFN_Hinge_28z
6D	Jurkat TRBC KO	1:8	Donor 2	KFN_CD8STK_28z
6E	Jurkat TRBC KO	1:8	Donor 2	KFN_CD28STK_CD28TM_28z
6F	Jurkat TRBC KO	1:8	Donor 2	aCD19-CAR
6G	Jurkat TRBC KO	1:8	Donor 2	Non-transduced
6H	Jurkat TRBC KO	N/A	N/A	N/A
9B	Jurkat TRBC KO	1:4	Donor 3	KFN_Hinge_28z
9D	Jurkat TRBC KO	1:4	Donor 3	KFN_CD8STK_28z
9E	Jurkat TRBC KO	1:4	Donor 3	KFN_CD28STK_CD28TM_28z
9F	Jurkat TRBC KO	1:4	Donor 3	aCD19-CAR
9G	Jurkat TRBC KO	1:4	Donor 3	Non-transduced
9H	Jurkat TRBC KO	N/A	N/A	N/A
10B	Jurkat TRBC KO	1:8	Donor 3	KFN_Hinge_28z
10D	Jurkat TRBC KO	1:8	Donor 3	KFN_CD8STK_28z
10E	Jurkat TRBC KO	1:8	Donor 3	KFN_CD28STK_CD28TM_28z
10F	Jurkat TRBC KO	1:8	Donor 3	aCD19-CAR
10G	Jurkat TRBC KO	1:8	Donor 3	Non-transduced
10H	Jurkat TRBC KO	N/A	N/A	N/A

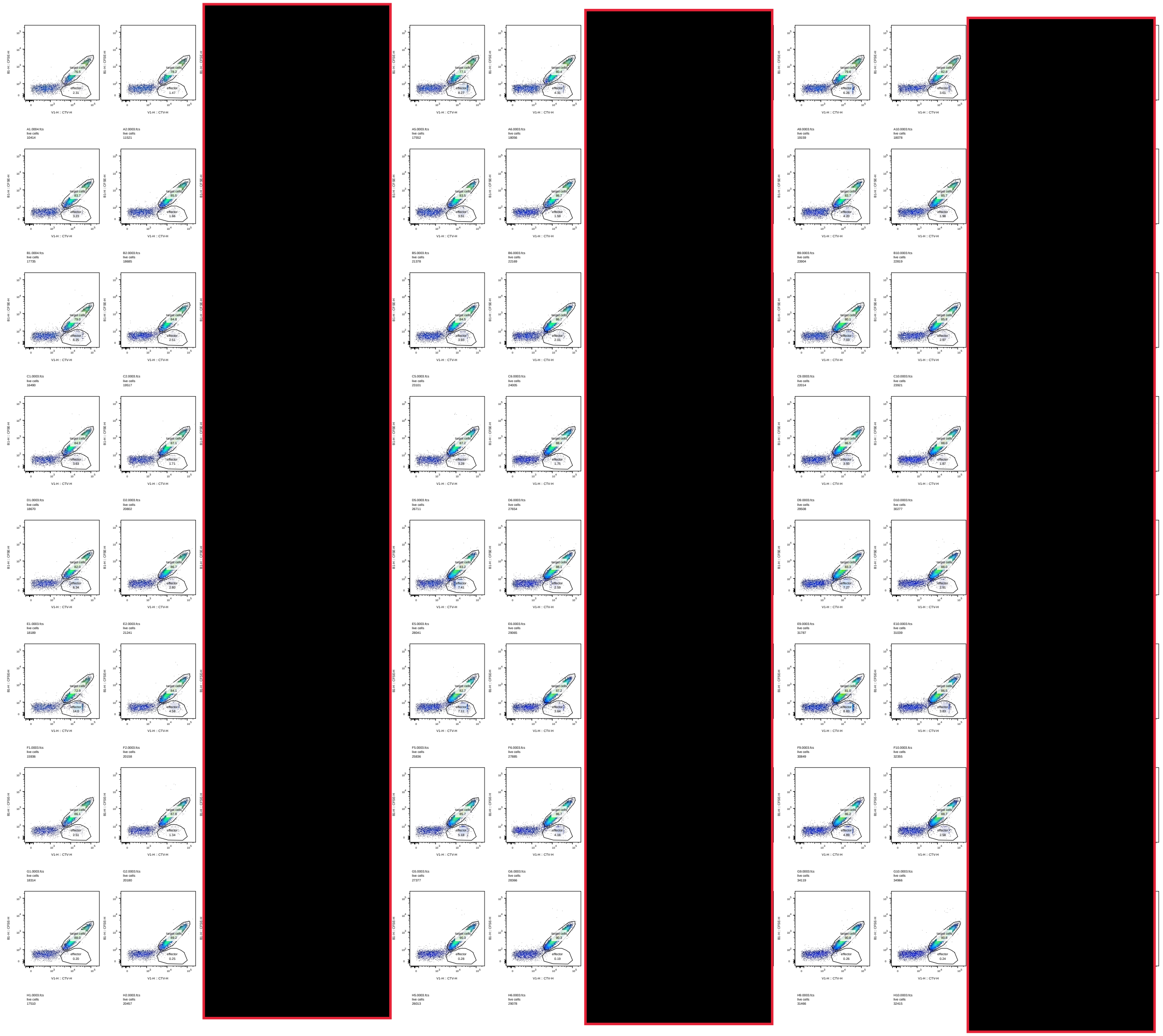

[redacted]

[redacted]

[redacted]

Plate4_KFN_vs_HPB_TRBC1

**Well**	**Target**	**E:T ratio**	**Donor**	**CAR construct**
**1B**	HPB TRBC1+ve	1:4	Donor 1	KFN_Hinge_28z
**1D**	HPB TRBC1+ve	1:4	Donor 1	KFN_CD8STK_28z
**1E**	HPB TRBC1+ve	1:4	Donor 1	KFN_CD28STK_CD28TM_28z
**1F**	HPB TRBC1+ve	1:4	Donor 1	aCD19-CAR
**1G**	HPB TRBC1+ve	1:4	Donor 1	Non-transduced
**1H**	HPB TRBC1+ve	N/A	N/A	N/A
**2B**	HPB TRBC1+ve	1:8	Donor 1	KFN_Hinge_28z
**2D**	HPB TRBC1+ve	1:8	Donor 1	KFN_CD8STK_28z
**2E**	HPB TRBC1+ve	1:8	Donor 1	KFN_CD28STK_CD28TM_28z
**2F**	HPB TRBC1+ve	1:8	Donor 1	aCD19-CAR
**2G**	HPB TRBC1+ve	1:8	Donor 1	Non-transduced
**2H**	HPB TRBC1+ve	N/A	N/A	N/A
**5B**	HPB TRBC1+ve	1:4	Donor 2	KFN_Hinge_28z
**5D**	HPB TRBC1+ve	1:4	Donor 2	KFN_CD8STK_28z
**5E**	HPB TRBC1+ve	1:4	Donor 2	KFN_CD28STK_CD28TM_28z
**5F**	HPB TRBC1+ve	1:4	Donor 2	aCD19-CAR
**5G**	HPB TRBC1+ve	1:4	Donor 2	Non-transduced
**5H**	HPB TRBC1+ve	N/A	N/A	N/A
**6B**	HPB TRBC1+ve	1:8	Donor 2	KFN_Hinge_28z
**6D**	HPB TRBC1+ve	1:8	Donor 2	KFN_CD8STK_28z
**6E**	HPB TRBC1+ve	1:8	Donor 2	KFN_CD28STK_CD28TM_28z
**6F**	HPB TRBC1+ve	1:8	Donor 2	aCD19-CAR
**6G**	HPB TRBC1+ve	1:8	Donor 2	Non-transduced
**6H**	HPB TRBC1+ve	N/A	N/A	N/A
**9B**	HPB TRBC1+ve	1:4	Donor 3	KFN_Hinge_28z
**9D**	HPB TRBC1+ve	1:4	Donor 3	KFN_CD8STK_28z
**9E**	HPB TRBC1+ve	1:4	Donor 3	KFN_CD28STK_CD28TM_28z
**9F**	HPB TRBC1+ve	1:4	Donor 3	aCD19-CAR
**9G**	HPB TRBC1+ve	1:4	Donor 3	Non-transduced
**9H**	HPB TRBC1+ve	N/A	N/A	N/A
**10B**	HPB TRBC1+ve	1:8	Donor 3	KFN_Hinge_28z
**10D**	HPB TRBC1+ve	1:8	Donor 3	KFN_CD8STK_28z
**10E**	HPB TRBC1+ve	1:8	Donor 3	KFN_CD28STK_CD28TM_28z
**10F**	HPB TRBC1+ve	1:8	Donor 3	aCD19-CAR
**10G**	HPB TRBC1+ve	1:8	Donor 3	Non-transduced
**10H**	HPB TRBC1+ve	N/A	N/A	N/A

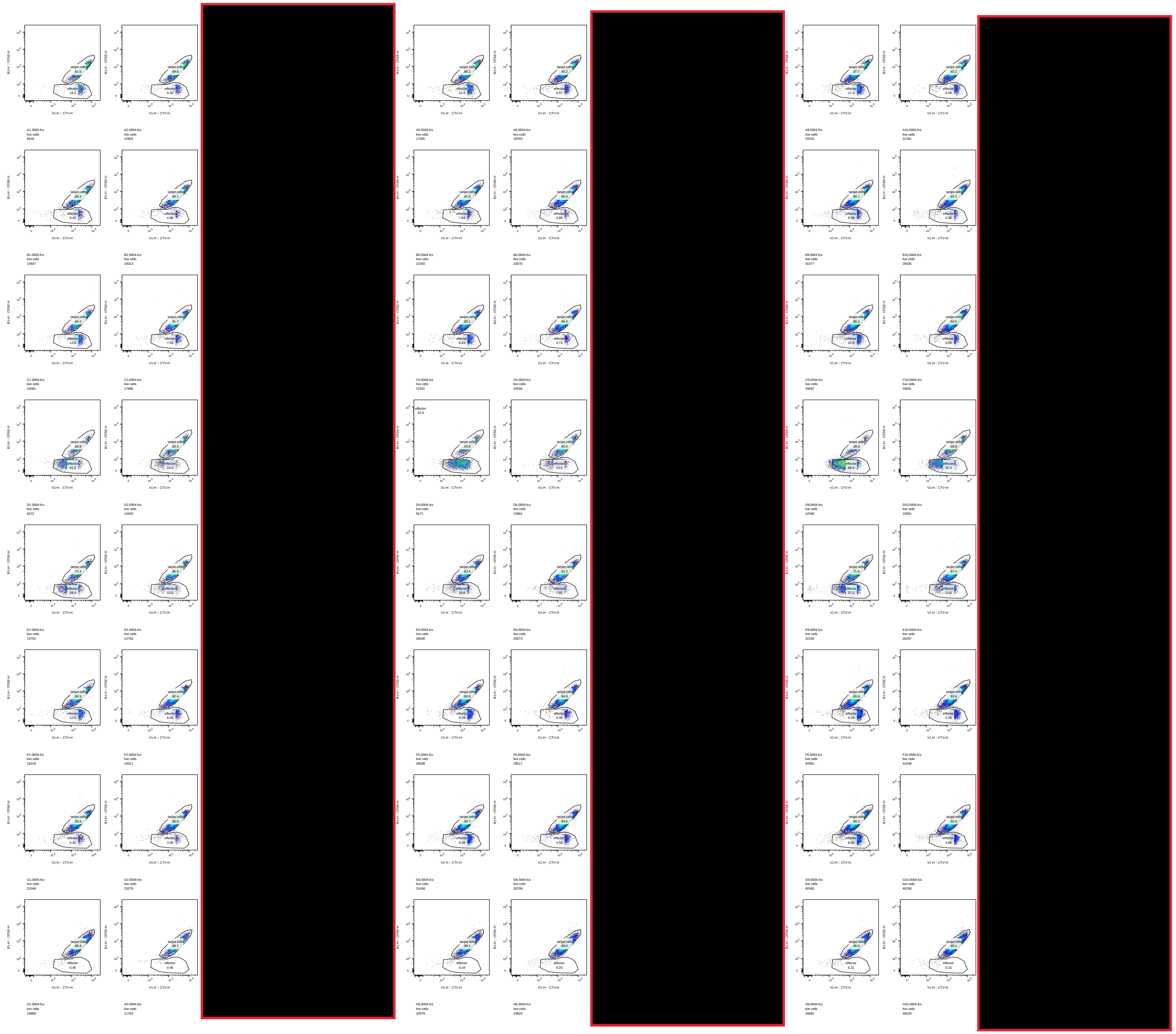

Plate5_KFN_vs_HPB_TRBC2

Well	Target	E:T ratio	Donor	CAR construct
**1B**	HPB TRBC2+ve	1:4	Donor 1	KFN_Hinge_28z
**1D**	HPB TRBC2+ve	1:4	Donor 1	KFN_CD8STK_28z
**1E**	HPB TRBC2+ve	1:4	Donor 1	KFN_CD28STK_CD28TM_28z
**1F**	HPB TRBC2+ve	1:4	Donor 1	aCD19-CAR
**1G**	HPB TRBC2+ve	1:4	Donor 1	Non-transduced
**1H**	HPB TRBC2+ve	N/A	N/A	N/A
**2B**	HPB TRBC2+ve	1:8	Donor 1	KFN_Hinge_28z
**2D**	HPB TRBC2+ve	1:8	Donor 1	KFN_CD8STK_28z
**2E**	HPB TRBC2+ve	1:8	Donor 1	KFN_CD28STK_CD28TM_28z
**2F**	HPB TRBC2+ve	1:8	Donor 1	aCD19-CAR
**2G**	HPB TRBC2+ve	1:8	Donor 1	Non-transduced
**2H**	HPB TRBC2+ve	N/A	N/A	N/A
**5B**	HPB TRBC2+ve	1:4	Donor 2	KFN_Hinge_28z
**5D**	HPB TRBC2+ve	1:4	Donor 2	KFN_CD8STK_28z
**5E**	HPB TRBC2+ve	1:4	Donor 2	KFN_CD28STK_CD28TM_28z
**5F**	HPB TRBC2+ve	1:4	Donor 2	aCD19-CAR
**5G**	HPB TRBC2+ve	1:4	Donor 2	Non-transduced
**5H**	HPB TRBC2+ve	N/A	N/A	N/A
**6B**	HPB TRBC2+ve	1:8	Donor 2	KFN_Hinge_28z
**6D**	HPB TRBC2+ve	1:8	Donor 2	KFN_CD8STK_28z
**6E**	HPB TRBC2+ve	1:8	Donor 2	KFN_CD28STK_CD28TM_28z
**6F**	HPB TRBC2+ve	1:8	Donor 2	aCD19-CAR
**6G**	HPB TRBC2+ve	1:8	Donor 2	Non-transduced
**6H**	HPB TRBC2+ve	N/A	N/A	N/A
**9B**	HPB TRBC2+ve	1:4	Donor 3	KFN_Hinge_28z
**9D**	HPB TRBC2+ve	1:4	Donor 3	KFN_CD8STK_28z
**9E**	HPB TRBC2+ve	1:4	Donor 3	KFN_CD28STK_CD28TM_28z
**9F**	HPB TRBC2+ve	1:4	Donor 3	aCD19-CAR
**9G**	HPB TRBC2+ve	1:4	Donor 3	Non-transduced
**9H**	HPB TRBC2+ve	N/A	N/A	N/A
**10B**	HPB TRBC2+ve	1:8	Donor 3	KFN_Hinge_28z
**10D**	HPB TRBC2+ve	1:8	Donor 3	KFN_CD8STK_28z
**10E**	HPB TRBC2+ve	1:8	Donor 3	KFN_CD28STK_CD28TM_28z
**10F**	HPB TRBC2+ve	1:8	Donor 3	aCD19-CAR
**10G**	HPB TRBC2+ve	1:8	Donor 3	Non-transduced
**10H**	HPB TRBC2+ve	N/A	N/A	N/A

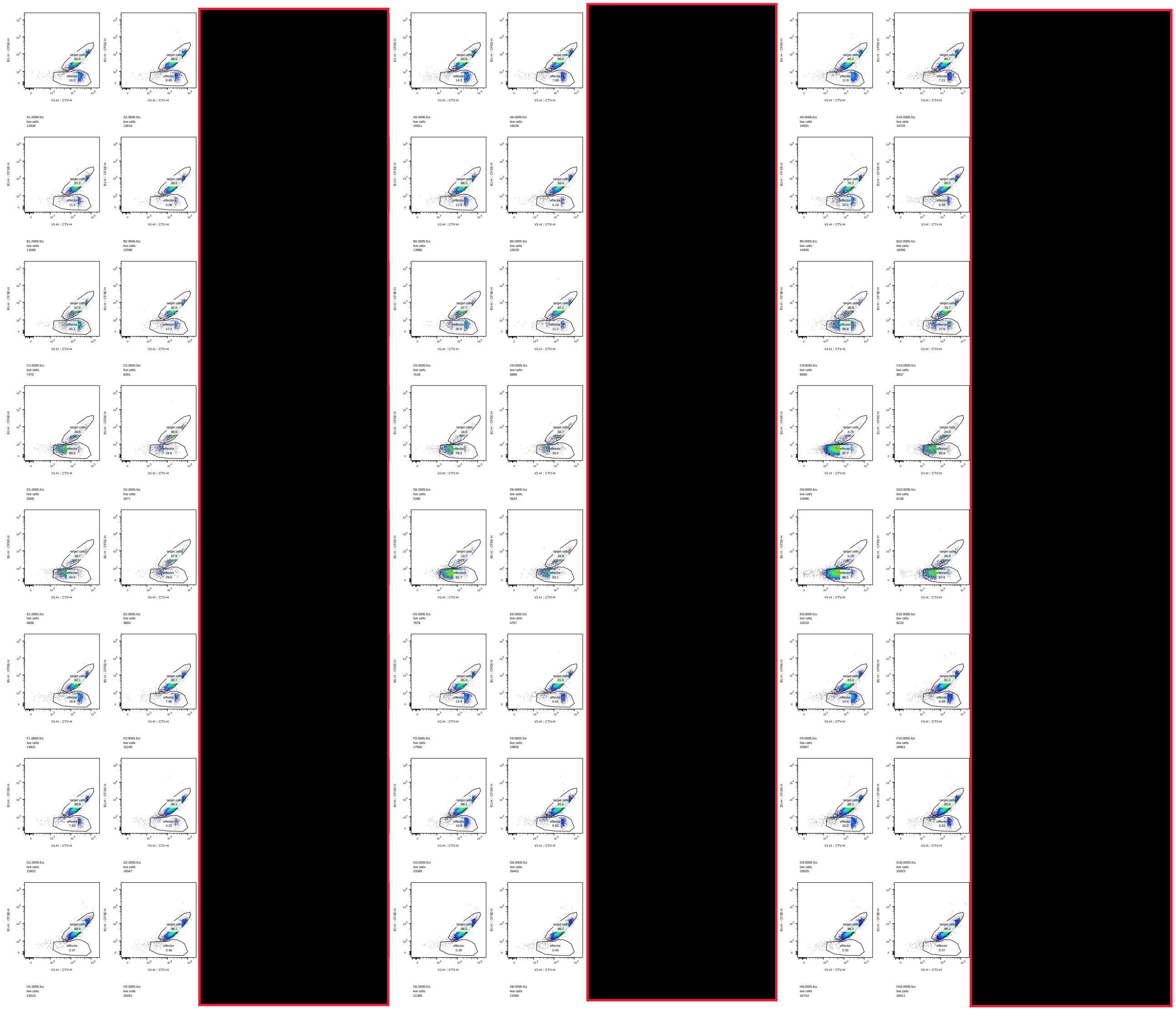

Plate6_KFN_vs_HPB_TRBC-KO

Well	Target	E:T ratio	Donor	CAR construct
**1B**	HPB TRBC KO	1:4	Donor 1	KFN_Hinge_28z
**1D**	HPB TRBC KO	1:4	Donor 1	KFN_CD8STK_28z
**1E**	HPB TRBC KO	1:4	Donor 1	KFN_CD28STK_CD28TM_28z
**1F**	HPB TRBC KO	1:4	Donor 1	aCD19-CAR
**1G**	HPB TRBC KO	1:4	Donor 1	Non-transduced
**1H**	HPB TRBC KO	N/A	N/A	N/A
**2B**	HPB TRBC KO	1:8	Donor 1	KFN_Hinge_28z
**2D**	HPB TRBC KO	1:8	Donor 1	KFN_CD8STK_28z
**2E**	HPB TRBC KO	1:8	Donor 1	KFN_CD28STK_CD28TM_28z
**2F**	HPB TRBC KO	1:8	Donor 1	aCD19-CAR
**2G**	HPB TRBC KO	1:8	Donor 1	Non-transduced
**2H**	HPB TRBC KO	N/A	N/A	N/A
**5B**	HPB TRBC KO	1:4	Donor 2	KFN_Hinge_28z
**5D**	HPB TRBC KO	1:4	Donor 2	KFN_CD8STK_28z
**5E**	HPB TRBC KO	1:4	Donor 2	KFN_CD28STK_CD28TM_28z
**5F**	HPB TRBC KO	1:4	Donor 2	aCD19-CAR
**5G**	HPB TRBC KO	1:4	Donor 2	Non-transduced
**5H**	HPB TRBC KO	N/A	N/A	N/A
**6B**	HPB TRBC KO	1:8	Donor 2	KFN_Hinge_28z
**6D**	HPB TRBC KO	1:8	Donor 2	KFN_CD8STK_28z
**6E**	HPB TRBC KO	1:8	Donor 2	KFN_CD28STK_CD28TM_28z
**6F**	HPB TRBC KO	1:8	Donor 2	aCD19-CAR
**6G**	HPB TRBC KO	1:8	Donor 2	Non-transduced
**6H**	HPB TRBC KO	N/A	N/A	N/A
**9B**	HPB TRBC KO	1:4	Donor 3	KFN_Hinge_28z
**9D**	HPB TRBC KO	1:4	Donor 3	KFN_CD8STK_28z
**9E**	HPB TRBC KO	1:4	Donor 3	KFN_CD28STK_CD28TM_28z
**9F**	HPB TRBC KO	1:4	Donor 3	aCD19-CAR
**9G**	HPB TRBC KO	1:4	Donor 3	Non-transduced
**9H**	HPB TRBC KO	N/A	N/A	N/A
**10B**	HPB TRBC KO	1:8	Donor 3	KFN_Hinge_28z
**10D**	HPB TRBC KO	1:8	Donor 3	KFN_CD8STK_28z
**10E**	HPB TRBC KO	1:8	Donor 3	KFN_CD28STK_CD28TM_28z
**10F**	HPB TRBC KO	1:8	Donor 3	aCD19-CAR
**10G**	HPB TRBC KO	1:8	Donor 3	Non-transduced
**10H**	HPB TRBC KO	N/A	N/A	N/A

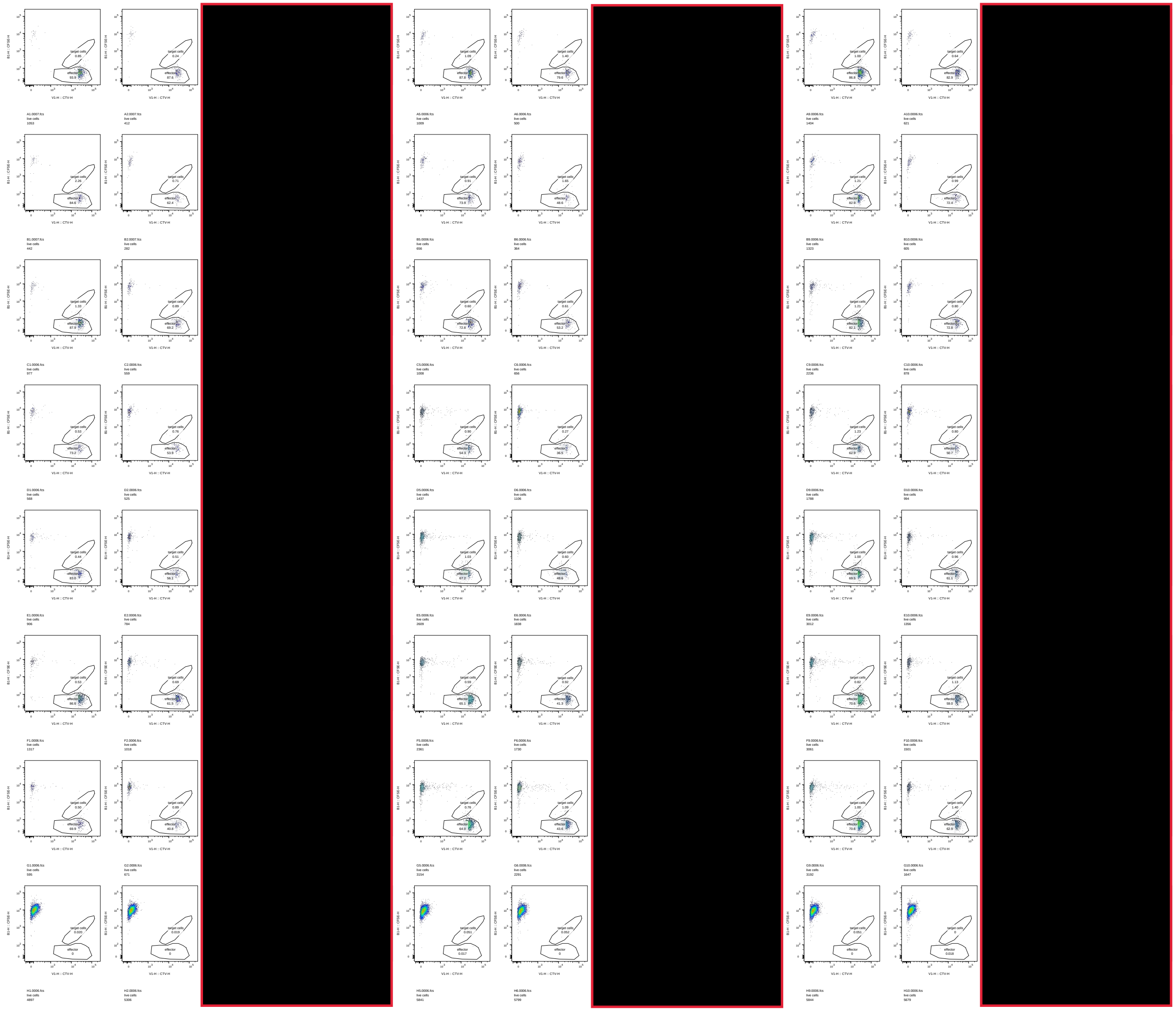

Plate7_KFN_vs_H9

**Well**	**Target**	**E:T ratio**	**Donor**	**CAR construct**
**1B**	H9	1:4	Donor 1	KFN_Hinge_28z
**1D**	H9	1:4	Donor 1	KFN_CD8STK_28z
**1E**	H9	1:4	Donor 1	KFN_CD28STK_CD28TM_28z
**1F**	H9	1:4	Donor 1	aCD19-CAR
**1G**	H9	1:4	Donor 1	Non-transduced
**1H**	H9	N/A	N/A	N/A
**2B**	H9	1:8	Donor 1	KFN_Hinge_28z
**2D**	H9	1:8	Donor 1	KFN_CD8STK_28z
**2E**	H9	1:8	Donor 1	KFN_CD28STK_CD28TM_28z
**2F**	H9	1:8	Donor 1	aCD19-CAR
**2G**	H9	1:8	Donor 1	Non-transduced
**2H**	H9	N/A	N/A	N/A
**5B**	H9	1:4	Donor 2	KFN_Hinge_28z
**5D**	H9	1:4	Donor 2	KFN_CD8STK_28z
**5E**	H9	1:4	Donor 2	KFN_CD28STK_CD28TM_28z
**5F**	H9	1:4	Donor 2	aCD19-CAR
**5G**	H9	1:4	Donor 2	Non-transduced
**5H**	H9	N/A	N/A	N/A
**6B**	H9	1:8	Donor 2	KFN_Hinge_28z
**6D**	H9	1:8	Donor 2	KFN_CD8STK_28z
**6E**	H9	1:8	Donor 2	KFN_CD28STK_CD28TM_28z
**6F**	H9	1:8	Donor 2	aCD19-CAR
**6G**	H9	1:8	Donor 2	Non-transduced
**6H**	H9	N/A	N/A	N/A
**9B**	H9	1:4	Donor 3	KFN_Hinge_28z
**9D**	H9	1:4	Donor 3	KFN_CD8STK_28z
**9E**	H9	1:4	Donor 3	KFN_CD28STK_CD28TM_28z
**9F**	H9	1:4	Donor 3	aCD19-CAR
**9G**	H9	1:4	Donor 3	Non-transduced
**9H**	H9	N/A	N/A	N/A
**10B**	H9	1:8	Donor 3	KFN_Hinge_28z
**10D**	H9	1:8	Donor 3	KFN_CD8STK_28z
**10E**	H9	1:8	Donor 3	KFN_CD28STK_CD28TM_28z
**10F**	H9	1:8	Donor 3	aCD19-CAR
**10G**	H9	1:8	Donor 3	Non-transduced
**10H**	H9	N/A	N/A	N/A

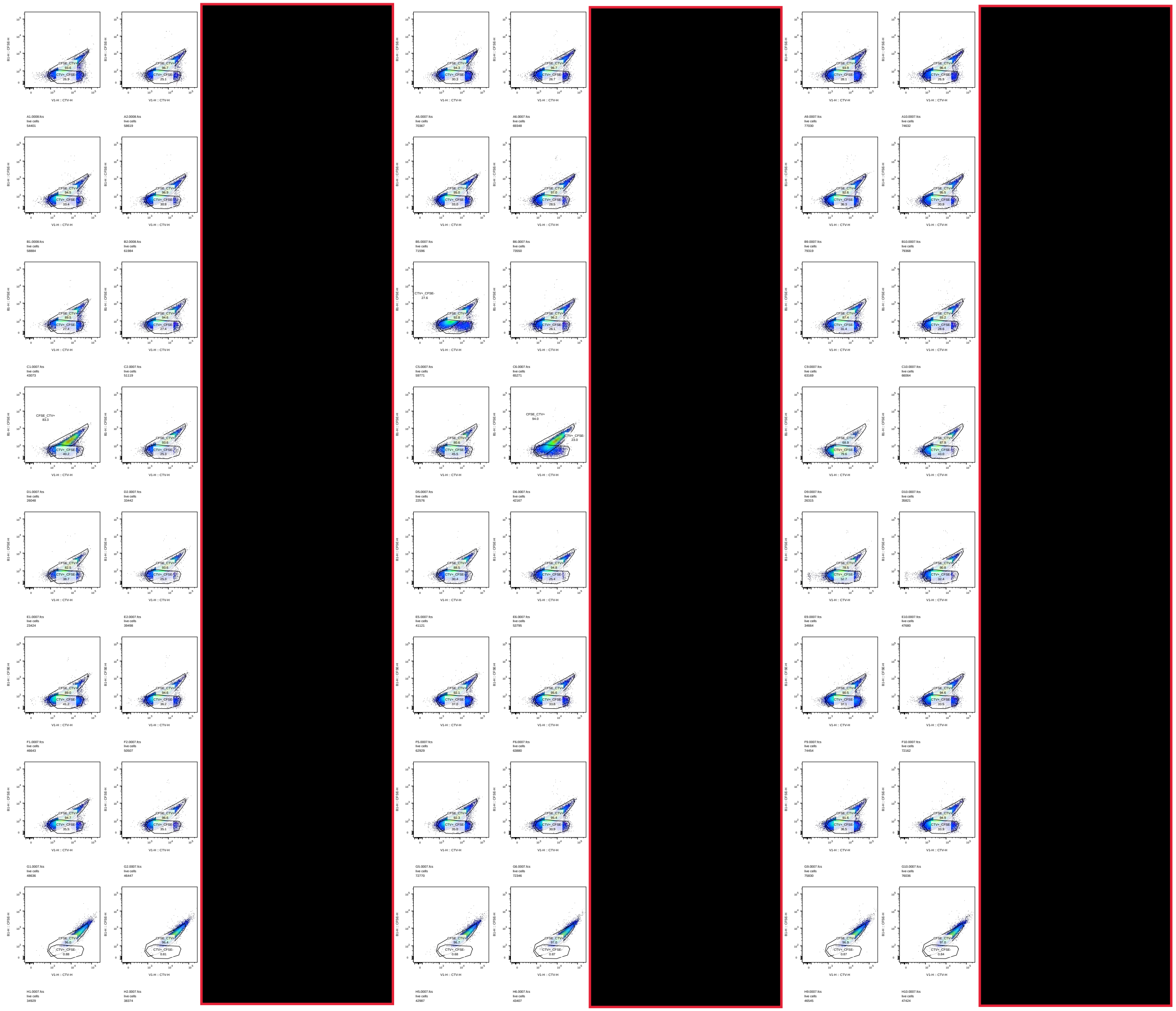

[redacted]

Plate8_KFN_vs_TALL1

Well	Target	E:T ratio	Donor	CAR construct
**1B**	T-ALL1	1:4	Donor 1	KFN_Hinge_28z
**1D**	T-ALL1	1:4	Donor 1	KFN_CD8STK_28z
**1E**	T-ALL1	1:4	Donor 1	KFN_CD28STK_CD28TM_28z
**1F**	T-ALL1	1:4	Donor 1	aCD19-CAR
**1G**	T-ALL1	1:4	Donor 1	Non-transduced
**1H**	T-ALL1	N/A	N/A	N/A
**2B**	T-ALL1	1:8	Donor 1	KFN_Hinge_28z
**2D**	T-ALL1	1:8	Donor 1	KFN_CD8STK_28z
**2E**	T-ALL1	1:8	Donor 1	KFN_CD28STK_CD28TM_28z
**2F**	T-ALL1	1:8	Donor 1	aCD19-CAR
**2G**	T-ALL1	1:8	Donor 1	Non-transduced
**2H**	T-ALL1	N/A	N/A	N/A
**5B**	T-ALL1	1:4	Donor 2	KFN_Hinge_28z
**5D**	T-ALL1	1:4	Donor 2	KFN_CD8STK_28z
**5E**	T-ALL1	1:4	Donor 2	KFN_CD28STK_CD28TM_28z
**5F**	T-ALL1	1:4	Donor 2	aCD19-CAR
**5G**	T-ALL1	1:4	Donor 2	Non-transduced
**5H**	T-ALL1	N/A	N/A	N/A
**6B**	T-ALL1	1:8	Donor 2	KFN_Hinge_28z
**6D**	T-ALL1	1:8	Donor 2	KFN_CD8STK_28z
**6E**	T-ALL1	1:8	Donor 2	KFN_CD28STK_CD28TM_28z
**6F**	T-ALL1	1:8	Donor 2	aCD19-CAR
**6G**	T-ALL1	1:8	Donor 2	Non-transduced
**6H**	T-ALL1	N/A	N/A	N/A
**9B**	T-ALL1	1:4	Donor 3	KFN_Hinge_28z
**9D**	T-ALL1	1:4	Donor 3	KFN_CD8STK_28z
**9E**	T-ALL1	1:4	Donor 3	KFN_CD28STK_CD28TM_28z
**9F**	T-ALL1	1:4	Donor 3	aCD19-CAR
**9G**	T-ALL1	1:4	Donor 3	Non-transduced
**9H**	T-ALL1	N/A	N/A	N/A
**10B**	T-ALL1	1:8	Donor 3	KFN_Hinge_28z
**10D**	T-ALL1	1:8	Donor 3	KFN_CD8STK_28z
**10E**	T-ALL1	1:8	Donor 3	KFN_CD28STK_CD28TM_28z
**10F**	T-ALL1	1:8	Donor 3	aCD19-CAR
**10G**	T-ALL1	1:8	Donor 3	Non-transduced
**10H**	T-ALL1	N/A	N/A	N/A

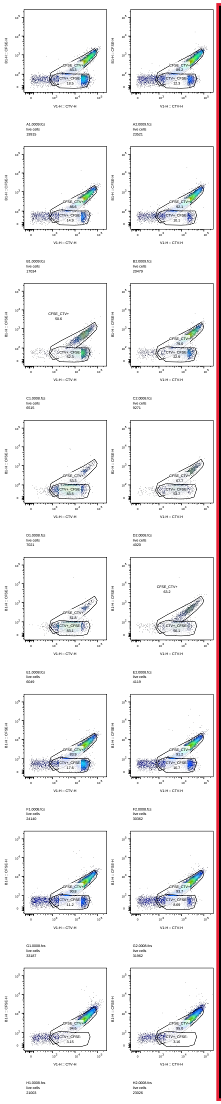

[redacted]

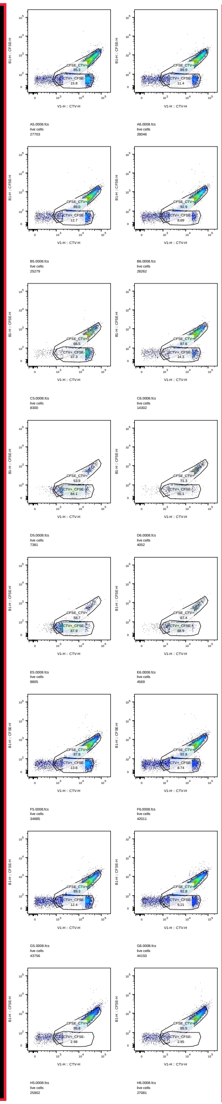

[redacted]

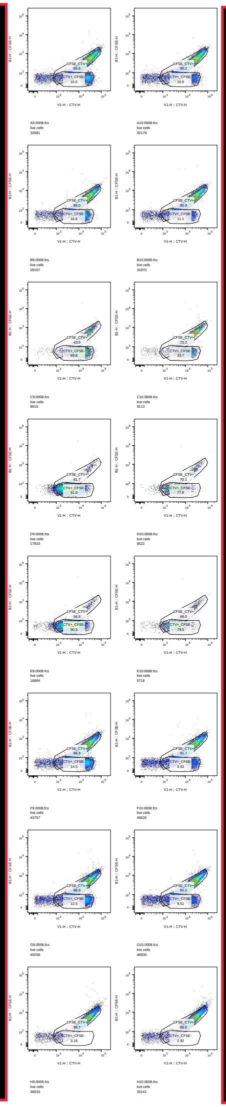

[redacted]

Plate12_JOVI_vs_Jurkat_TRBC-KO

Well	Target	E:T ratio	Donor	CAR construct
**1A**	Jurkat TRBC KO	1:4	Donor 1	JOVI_Hinge_41bbz
**1D**	Jurkat TRBC KO	1:4	Donor 1	JOVI_CD8STK_28z
**1E**	Jurkat TRBC KO	1:4	Donor 1	JOVI_CD28STK_CD28TM_28z
**1F**	Jurkat TRBC KO	1:4	Donor 1	mJOVI_Hinge_41bbz
**1G**	Jurkat TRBC KO	1:4	Donor 1	aCD19-CAR
**1H**	Jurkat TRBC KO	1:4	Donor 1	Non-transduced
**2A**	Jurkat TRBC KO	1:8	Donor 1	JOVI_Hinge_41bbz
**2D**	Jurkat TRBC KO	1:8	Donor 1	JOVI_CD8STK_28z
**2E**	Jurkat TRBC KO	1:8	Donor 1	JOVI_CD28STK_CD28TM_28z
**2F**	Jurkat TRBC KO	1:8	Donor 1	mJOVI_Hinge_41bbz
**2G**	Jurkat TRBC KO	1:8	Donor 1	aCD19-CAR
**2H**	Jurkat TRBC KO	1:8	Donor 1	Non-transduced
**5A**	Jurkat TRBC KO	1:4	Donor 2	JOVI_Hinge_41bbz
**5D**	Jurkat TRBC KO	1:4	Donor 2	JOVI_CD8STK_28z
**5E**	Jurkat TRBC KO	1:4	Donor 2	JOVI_CD28STK_CD28TM_28z
**5F**	Jurkat TRBC KO	1:4	Donor 2	mJOVI_Hinge_41bbz
**5G**	Jurkat TRBC KO	1:4	Donor 2	aCD19-CAR
**5H**	Jurkat TRBC KO	1:4	Donor 2	Non-transduced
**6A**	Jurkat TRBC KO	1:8	Donor 2	JOVI_Hinge_41bbz
**6D**	Jurkat TRBC KO	1:8	Donor 2	JOVI_CD8STK_28z
**6E**	Jurkat TRBC KO	1:8	Donor 2	JOVI_CD28STK_CD28TM_28z
**6F**	Jurkat TRBC KO	1:8	Donor 2	mJOVI_Hinge_41bbz
**6G**	Jurkat TRBC KO	1:8	Donor 2	aCD19-CAR
**6H**	Jurkat TRBC KO	1:8	Donor 2	Non-transduced
**9A**	Jurkat TRBC KO	1:4	Donor 3	JOVI_Hinge_41bbz
**9D**	Jurkat TRBC KO	1:4	Donor 3	JOVI_CD8STK_28z
**9E**	Jurkat TRBC KO	1:4	Donor 3	JOVI_CD28STK_CD28TM_28z
**9F**	Jurkat TRBC KO	1:4	Donor 3	mJOVI_Hinge_41bbz
**9G**	Jurkat TRBC KO	1:4	Donor 3	aCD19-CAR
**9H**	Jurkat TRBC KO	1:4	Donor 3	Non-transduced
**10A**	Jurkat TRBC KO	1:8	Donor 3	JOVI_Hinge_41bbz
**10D**	Jurkat TRBC KO	1:8	Donor 3	JOVI_CD8STK_28z
**10E**	Jurkat TRBC KO	1:8	Donor 3	JOVI_CD28STK_CD28TM_28z
**10F**	Jurkat TRBC KO	1:8	Donor 3	mJOVI_Hinge_41bbz
**10G**	Jurkat TRBC KO	1:8	Donor 3	aCD19-CAR
**10H**	Jurkat TRBC KO	1:8	Donor 3	Non-transduced

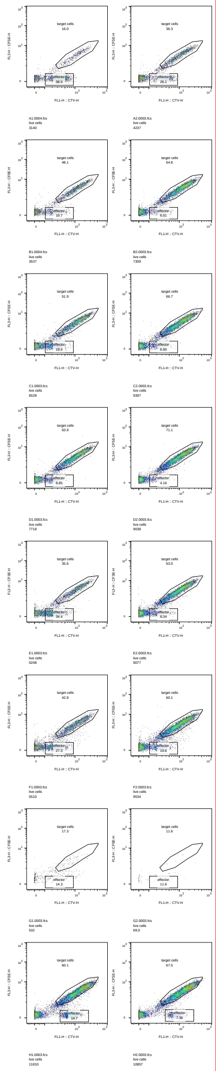

[redacted]

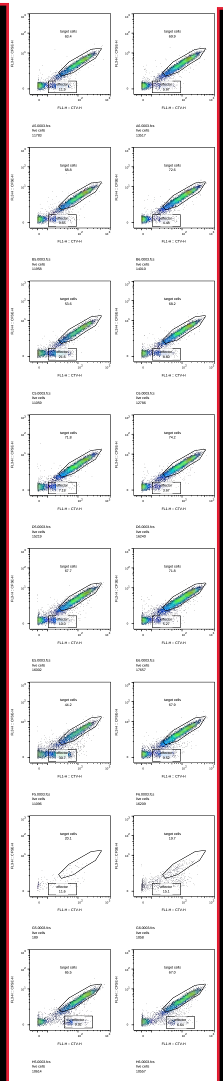

[redacted]

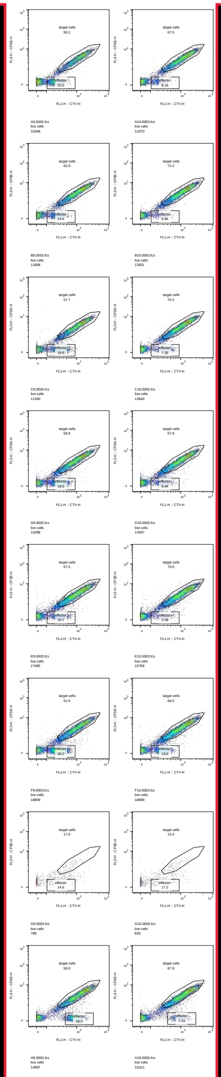

[redacted]

Plate13_JOVI_vs_HPB_TRBC1

Well	Target	E:T ratio	Donor	CAR construct
**1A**	HPB TRBC1+ve	1:4	Donor 1	JOVI_Hinge_41bbz
**1D**	HPB TRBC1+ve	1:4	Donor 1	JOVI_CD8STK_28z
**1E**	HPB TRBC1+ve	1:4	Donor 1	JOVI_CD28STK_CD28TM_28z
**1G**	HPB TRBC1+ve	1:4	Donor 1	aCD19-CAR
**1H**	HPB TRBC1+ve	1:4	Donor 1	Non-transduced
**2A**	HPB TRBC1+ve	1:8	Donor 1	JOVI_Hinge_41bbz
**2D**	HPB TRBC1+ve	1:8	Donor 1	JOVI_CD8STK_28z
**2E**	HPB TRBC1+ve	1:8	Donor 1	JOVI_CD28STK_CD28TM_28z
**2G**	HPB TRBC1+ve	1:8	Donor 1	aCD19-CAR
**2H**	HPB TRBC1+ve	1:8	Donor 1	Non-transduced
**5A**	HPB TRBC1+ve	1:4	Donor 2	JOVI_Hinge_41bbz
**5D**	HPB TRBC1+ve	1:4	Donor 2	JOVI_CD8STK_28z
**5E**	HPB TRBC1+ve	1:4	Donor 2	JOVI_CD28STK_CD28TM_28z
**5G**	HPB TRBC1+ve	1:4	Donor 2	aCD19-CAR
**5H**	HPB TRBC1+ve	1:4	Donor 2	Non-transduced
**6A**	HPB TRBC1+ve	1:8	Donor 2	JOVI_Hinge_41bbz
**6D**	HPB TRBC1+ve	1:8	Donor 2	JOVI_CD8STK_28z
**6E**	HPB TRBC1+ve	1:8	Donor 2	JOVI_CD28STK_CD28TM_28z
**6G**	HPB TRBC1+ve	1:8	Donor 2	aCD19-CAR
**6H**	HPB TRBC1+ve	1:8	Donor 2	Non-transduced
**9A**	HPB TRBC1+ve	1:4	Donor 3	JOVI_Hinge_41bbz
**9D**	HPB TRBC1+ve	1:4	Donor 3	JOVI_CD8STK_28z
**9E**	HPB TRBC1+ve	1:4	Donor 3	JOVI_CD28STK_CD28TM_28z
**9G**	HPB TRBC1+ve	1:4	Donor 3	aCD19-CAR
**9H**	HPB TRBC1+ve	1:4	Donor 3	Non-transduced
**10A**	HPB TRBC1+ve	1:8	Donor 3	JOVI_Hinge_41bbz
**10D**	HPB TRBC1+ve	1:8	Donor 3	JOVI_CD8STK_28z
**10E**	HPB TRBC1+ve	1:8	Donor 3	JOVI_CD28STK_CD28TM_28z
**10G**	HPB TRBC1+ve	1:8	Donor 3	aCD19-CAR
**10H**	HPB TRBC1+ve	1:8	Donor 3	Non-transduced

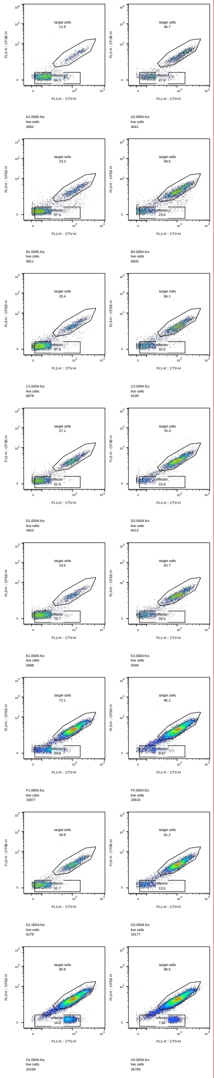

[redacted]

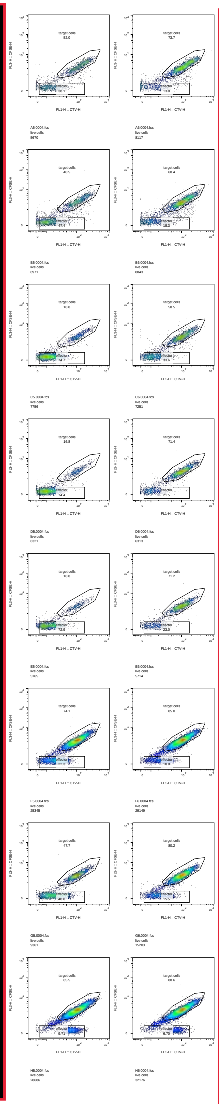

[redacted]

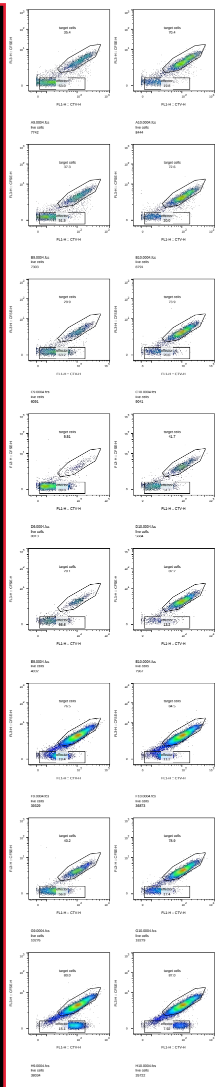

[redacted]

Plate14_JOVI_vs_HPB_TRBC2

Well	Target	E:T ratio	Donor	CAR construct
**1A**	HPB TRBC2+ve	1:4	Donor 1	JOVI_Hinge_41bbz
**1D**	HPB TRBC2+ve	1:4	Donor 1	JOVI_CD8STK_28z
**1E**	HPB TRBC2+ve	1:4	Donor 1	JOVI_CD28STK_CD28TM_28z
**1G**	HPB TRBC2+ve	1:4	Donor 1	aCD19-CAR
**1H**	HPB TRBC2+ve	1:4	Donor 1	Non-transduced
**2A**	HPB TRBC2+ve	1:8	Donor 1	JOVI_Hinge_41bbz
**2D**	HPB TRBC2+ve	1:8	Donor 1	JOVI_CD8STK_28z
**2E**	HPB TRBC2+ve	1:8	Donor 1	JOVI_CD28STK_CD28TM_28z
**2G**	HPB TRBC2+ve	1:8	Donor 1	aCD19-CAR
**2H**	HPB TRBC2+ve	1:8	Donor 1	Non-transduced
**5A**	HPB TRBC2+ve	1:4	Donor 2	JOVI_Hinge_41bbz
**5D**	HPB TRBC2+ve	1:4	Donor 2	JOVI_CD8STK_28z
**5E**	HPB TRBC2+ve	1:4	Donor 2	JOVI_CD28STK_CD28TM_28z
**5G**	HPB TRBC2+ve	1:4	Donor 2	aCD19-CAR
**5H**	HPB TRBC2+ve	1:4	Donor 2	Non-transduced
**6A**	HPB TRBC2+ve	1:8	Donor 2	JOVI_Hinge_41bbz
**6D**	HPB TRBC2+ve	1:8	Donor 2	JOVI_CD8STK_28z
**6E**	HPB TRBC2+ve	1:8	Donor 2	JOVI_CD28STK_CD28TM_28z
**6G**	HPB TRBC2+ve	1:8	Donor 2	aCD19-CAR
**6H**	HPB TRBC2+ve	1:8	Donor 2	Non-transduced
**9A**	HPB TRBC2+ve	1:4	Donor 3	JOVI_Hinge_41bbz
**9D**	HPB TRBC2+ve	1:4	Donor 3	JOVI_CD8STK_28z
**9E**	HPB TRBC2+ve	1:4	Donor 3	JOVI_CD28STK_CD28TM_28z
**9G**	HPB TRBC2+ve	1:4	Donor 3	aCD19-CAR
**9H**	HPB TRBC2+ve	1:4	Donor 3	Non-transduced
**10A**	HPB TRBC2+ve	1:8	Donor 3	JOVI_Hinge_41bbz
**10D**	HPB TRBC2+ve	1:8	Donor 3	JOVI_CD8STK_28z
**10E**	HPB TRBC2+ve	1:8	Donor 3	JOVI_CD28STK_CD28TM_28z
**10G**	HPB TRBC2+ve	1:8	Donor 3	aCD19-CAR
**10H**	HPB TRBC2+ve	1:8	Donor 3	Non-transduced

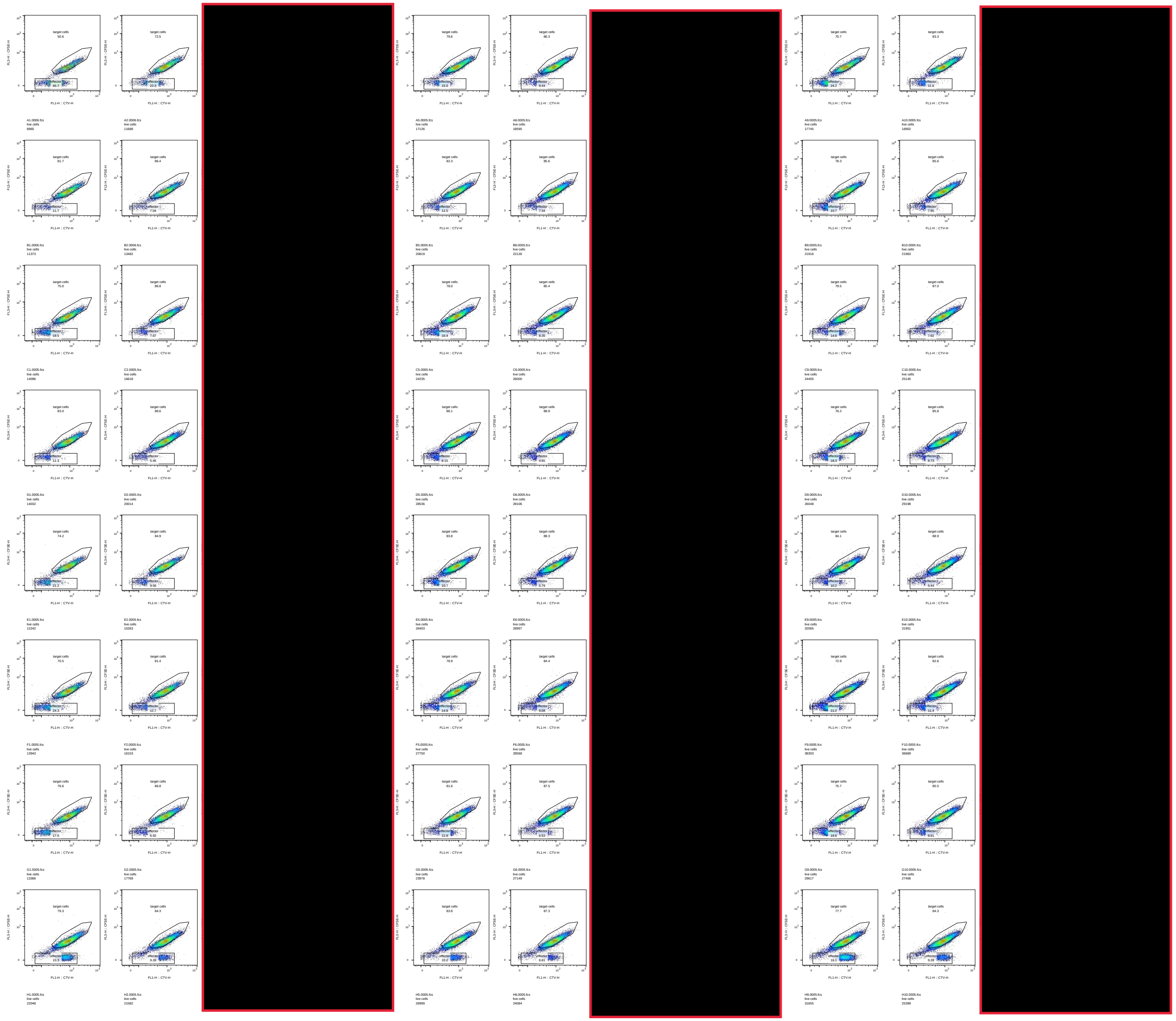

Plate15_JOVI_vs_HPB_TRBC-KO

**Well**	**Target**	**E:T ratio**	**Donor**	**CAR construct**
**1A**	HPB TRBC KO	1:4	Donor 1	JOVI_Hinge_41bbz
**1D**	HPB TRBC KO	1:4	Donor 1	JOVI_CD8STK_28z
**1E**	HPB TRBC KO	1:4	Donor 1	JOVI_CD28STK_CD28TM_28z
**1G**	HPB TRBC KO	1:4	Donor 1	aCD19-CAR
**1H**	HPB TRBC KO	1:4	Donor 1	Non-transduced
**2A**	HPB TRBC KO	1:8	Donor 1	JOVI_Hinge_41bbz
**2D**	HPB TRBC KO	1:8	Donor 1	JOVI_CD8STK_28z
**2E**	HPB TRBC KO	1:8	Donor 1	JOVI_CD28STK_CD28TM_28z
**2G**	HPB TRBC KO	1:8	Donor 1	aCD19-CAR
**2H**	HPB TRBC KO	1:8	Donor 1	Non-transduced
**5A**	HPB TRBC KO	1:4	Donor 2	JOVI_Hinge_41bbz
**5D**	HPB TRBC KO	1:4	Donor 2	JOVI_CD8STK_28z
**5E**	HPB TRBC KO	1:4	Donor 2	JOVI_CD28STK_CD28TM_28z
**5G**	HPB TRBC KO	1:4	Donor 2	aCD19-CAR
**5H**	HPB TRBC KO	1:4	Donor 2	Non-transduced
**6A**	HPB TRBC KO	1:8	Donor 2	JOVI_Hinge_41bbz
**6D**	HPB TRBC KO	1:8	Donor 2	JOVI_CD8STK_28z
**6E**	HPB TRBC KO	1:8	Donor 2	JOVI_CD28STK_CD28TM_28z
**6G**	HPB TRBC KO	1:8	Donor 2	aCD19-CAR
**6H**	HPB TRBC KO	1:8	Donor 2	Non-transduced
**9A**	HPB TRBC KO	1:4	Donor 3	JOVI_Hinge_41bbz
**9D**	HPB TRBC KO	1:4	Donor 3	JOVI_CD8STK_28z
**9E**	HPB TRBC KO	1:4	Donor 3	JOVI_CD28STK_CD28TM_28z
**9G**	HPB TRBC KO	1:4	Donor 3	aCD19-CAR
**9H**	HPB TRBC KO	1:4	Donor 3	Non-transduced
**10A**	HPB TRBC KO	1:8	Donor 3	JOVI_Hinge_41bbz
**10D**	HPB TRBC KO	1:8	Donor 3	JOVI_CD8STK_28z
**10E**	HPB TRBC KO	1:8	Donor 3	JOVI_CD28STK_CD28TM_28z
**10G**	HPB TRBC KO	1:8	Donor 3	aCD19-CAR
**10H**	HPB TRBC KO	1:8	Donor 3	Non-transduced

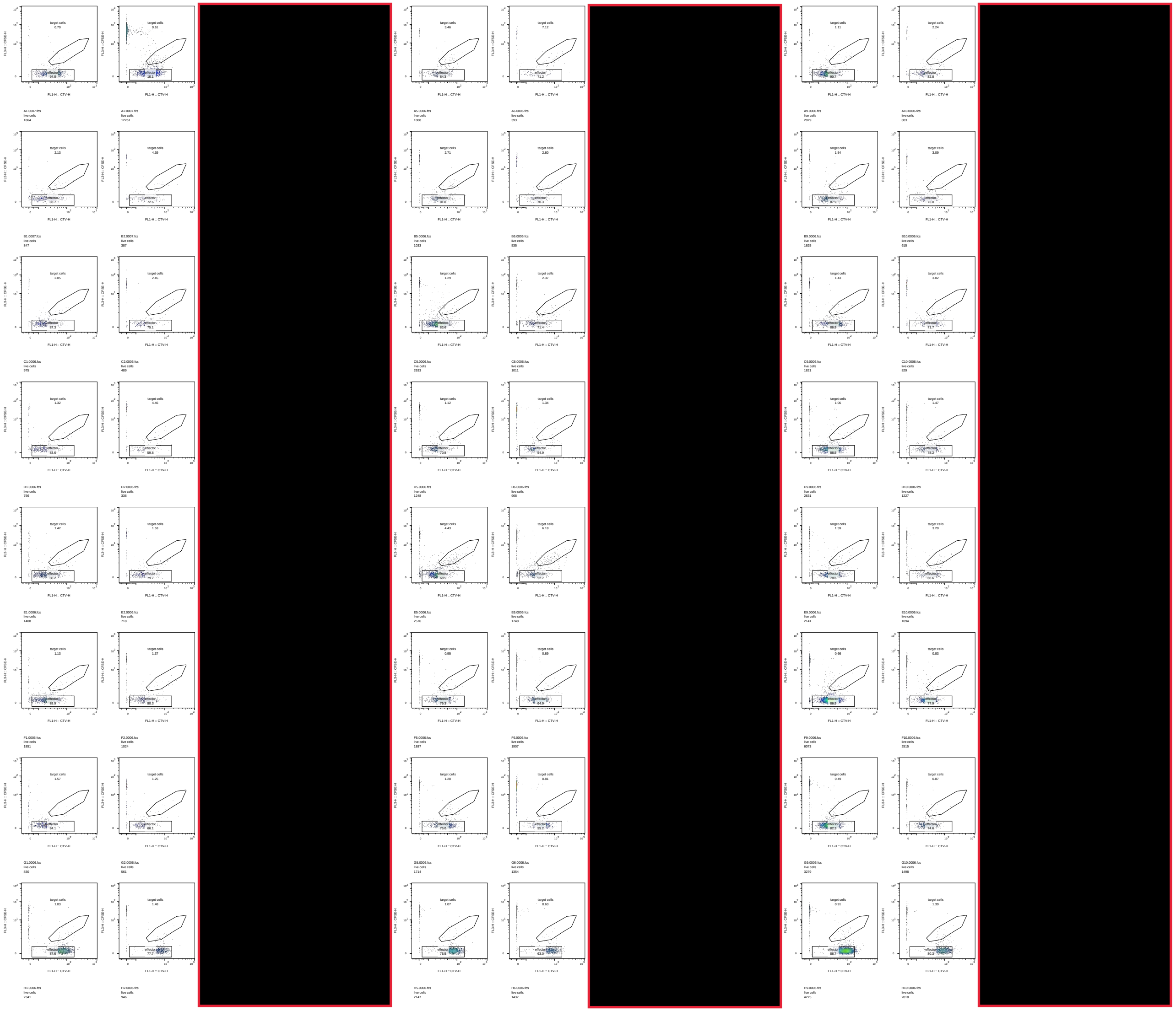

[redacted]

[redacted]

[redacted]

Plate17_JOVI_vs_TALL1

Well	Target	E:T ratio	Donor	CAR construct
**1A**	T-ALL1	1:4	Donor 1	JOVI_Hinge_41bbz
**1D**	T-ALL1	1:4	Donor 1	JOVI_CD8STK_28z
**1E**	T-ALL1	1:4	Donor 1	JOVI_CD28STK_CD28TM_28z
**1G**	T-ALL1	1:4	Donor 1	aCD19-CAR
**1H**	T-ALL1	1:4	Donor 1	Non-transduced
**2A**	T-ALL1	1:8	Donor 1	JOVI_Hinge_41bbz
**2D**	T-ALL1	1:8	Donor 1	JOVI_CD8STK_28z
**2E**	T-ALL1	1:8	Donor 1	JOVI_CD28STK_CD28TM_28z
**2G**	T-ALL1	1:8	Donor 1	aCD19-CAR
**2H**	T-ALL1	1:8	Donor 1	Non-transduced
**5A**	T-ALL1	1:4	Donor 2	JOVI_Hinge_41bbz
**5D**	T-ALL1	1:4	Donor 2	JOVI_CD8STK_28z
**5E**	T-ALL1	1:4	Donor 2	JOVI_CD28STK_CD28TM_28z
**5G**	T-ALL1	1:4	Donor 2	aCD19-CAR
**5H**	T-ALL1	1:4	Donor 2	Non-transduced
**6A**	T-ALL1	1:8	Donor 2	JOVI_Hinge_41bbz
**6D**	T-ALL1	1:8	Donor 2	JOVI_CD8STK_28z
**6E**	T-ALL1	1:8	Donor 2	JOVI_CD28STK_CD28TM_28z
**6G**	T-ALL1	1:8	Donor 2	aCD19-CAR
**6H**	T-ALL1	1:8	Donor 2	Non-transduced
**9A**	T-ALL1	1:4	Donor 3	JOVI_Hinge_41bbz
**9D**	T-ALL1	1:4	Donor 3	JOVI_CD8STK_28z
**9E**	T-ALL1	1:4	Donor 3	JOVI_CD28STK_CD28TM_28z
**9G**	T-ALL1	1:4	Donor 3	aCD19-CAR
**9H**	T-ALL1	1:4	Donor 3	Non-transduced
**10D**	T-ALL1	1:8	Donor 3	JOVI_CD8STK_28z
**10E**	T-ALL1	1:8	Donor 3	JOVI_CD28STK_CD28TM_28z
**10G**	T-ALL1	1:8	Donor 3	aCD19-CAR
**10H**	T-ALL1	1:8	Donor 3	Non-transduced

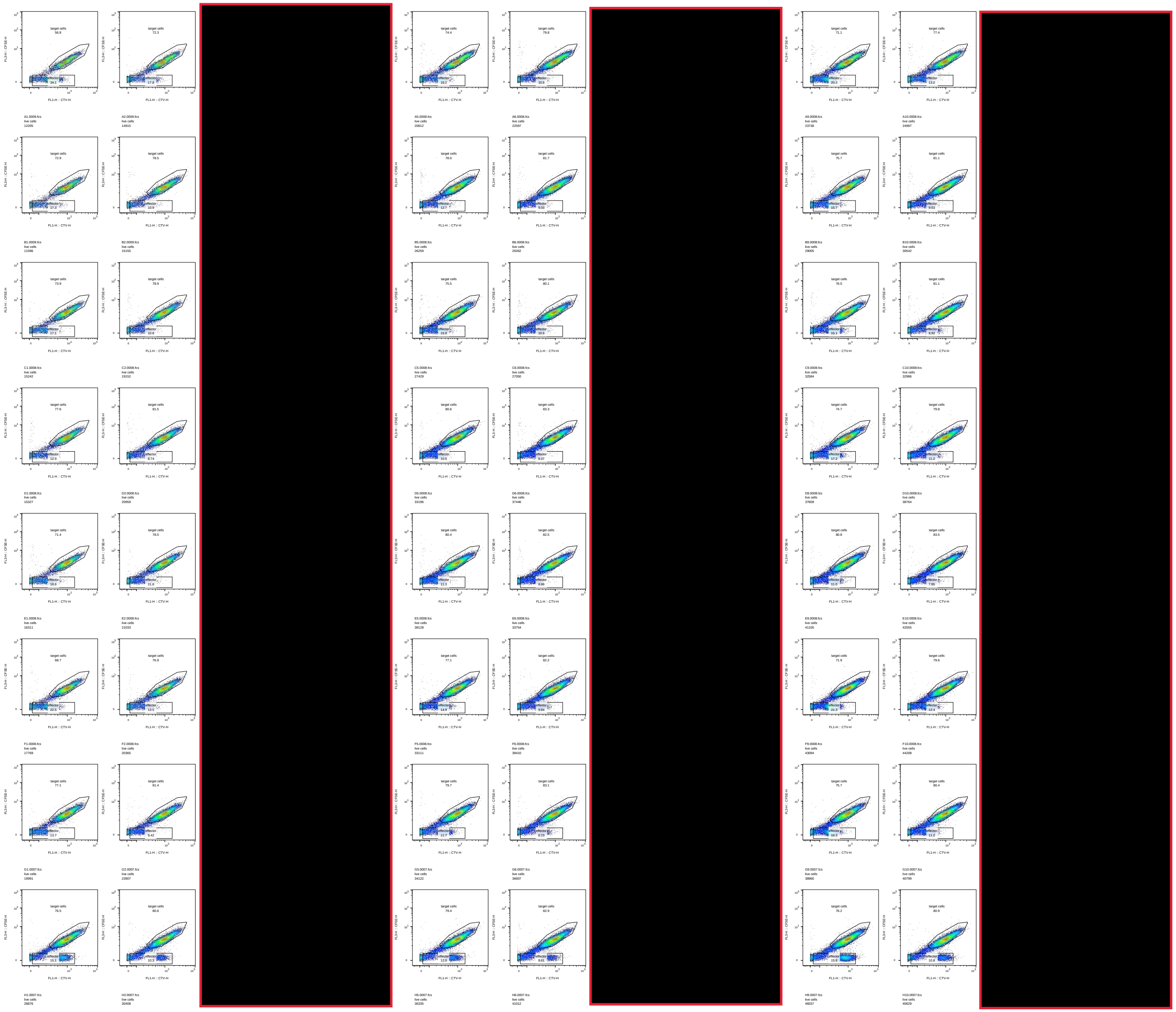

FL3-H :: CFSE-H
FL1-H :: CTV-H
target cells 56.8
effector 34.1
A1.0009.fcs
live cells
12205
target cells 72.3
effector 17.8
A2.0009.fcs
live cells
14915
target cells 72.9
effector 17.3
B1.0009.fcs
live cells
11596
target cells 78.5
effector 10.9
B2.0009.fcs
live cells
15155
target cells 73.9
effector 17.1
C1.0008.fcs
live cells
15242
target cells 78.9
effector 10.8
C2.0008.fcs
live cells
19152
target cells 77.6
effector 12.9
D1.0008.fcs
live cells
15327
target cells 81.5
effector 8.74
D2.0008.fcs
live cells
20959
target cells 71.4
effector 18.6
E1.0008.fcs
live cells
16311
target cells 78.5
effector 11.6
E2.0008.fcs
live cells
21033
target cells 68.7
effector 22.5
F1.0008.fcs
live cells
17769
target cells 76.9
effector 13.5
F2.0008.fcs
live cells
20365
target cells 77.1
effector 13.7
G1.0007.fcs
live cells
19991
target cells 81.4
effector 9.42
G2.0007.fcs
live cells
23907
target cells 76.5
effector 15.1
H1.0007.fcs
live cells
28876
target cells 80.6
effector 10.3
H2.0007.fcs
live cells
30408
[redacted]
target cells 74.4
effector 16.2
A5.0008.fcs
live cells
20812
target cells 79.8
effector 10.8
A6.0008.fcs
live cells
22597
target cells 78.0
effector 12.7
B5.0008.fcs
live cells
26259
target cells 81.7
effector 9.03
B6.0008.fcs
live cells
29262
target cells 75.5
effector 15.9
C5.0008.fcs
live cells
27429
target cells 80.1
effector 10.6
C6.0008.fcs
live cells
27050
target cells 80.6
effector 10.9
D5.0008.fcs
live cells
33196
target cells 83.3
effector 8.07
D6.0008.fcs
live cells
37446
target cells 80.4
effector 11.3
E5.0008.fcs
live cells
38128
target cells 82.5
effector 8.86
E6.0008.fcs
live cells
33754
target cells 77.1
effector 14.9
F5.0008.fcs
live cells
33111
target cells 82.2
effector 9.84
F6.0008.fcs
live cells
38410
target cells 79.7
effector 11.7
G5.0007.fcs
live cells
34122
target cells 83.1
effector 8.23
G6.0007.fcs
live cells
36607
target cells 79.4
effector 12.0
H5.0007.fcs
live cells
36335
target cells 82.9
effector 8.61
H6.0007.fcs
live cells
41012
[redacted]
target cells 71.1
effector 20.3
A9.0008.fcs
live cells
23738
target cells 77.4
effector 13.0
A10.0008.fcs
live cells
24997
target cells 75.7
effector 15.7
B9.0008.fcs
live cells
29005
target cells 81.1
effector 9.63
B10.0008.fcs
live cells
30542
target cells 76.0
effector 15.1
C9.0008.fcs
live cells
32584
target cells 81.1
effector 9.92
C10.0008.fcs
live cells
32986
target cells 74.7
effector 17.2
D9.0008.fcs
live cells
37609
target cells 79.8
effector 11.2
D10.0008.fcs
live cells
38764
target cells 80.8
effector 11.0
E9.0008.fcs
live cells
41105
target cells 83.5
effector 7.85
E10.0008.fcs
live cells
42555
target cells 71.9
effector 21.3
F9.0008.fcs
live cells
43094
target cells 79.6
effector 12.4
F10.0008.fcs
live cells
44208
target cells 75.7
effector 16.3
G9.0007.fcs
live cells
38960
target cells 80.4
effector 11.0
G10.0007.fcs
live cells
40799
target cells 76.2
effector 15.8
H9.0007.fcs
live cells
46037
target cells 80.9
effector 10.6
H10.0007.fcs
live cells
40629
[redacted]

Plate19_KFN-Prol_vs_Jurkats

Well	Target	E:T ratio	Donor	CAR construct
1B	Jurkat TRBC1+ve	1:4	Donor 4	KFN_Hinge_28z
1D	Jurkat TRBC1+ve	1:4	Donor 4	KFN_CD8STK_28z
1E	Jurkat TRBC1+ve	1:4	Donor 4	KFN_CD28STK_CD28TM_28z
1F	Jurkat TRBC1+ve	1:4	Donor 4	aCD19-CAR
1G	Jurkat TRBC1+ve	1:4	Donor 4	Non-transduced
1H	Jurkat TRBC1+ve	N/A	N/A	N/A
2B	Jurkat TRBC1+ve	1:4	Donor 5	KFN_Hinge_28z
2D	Jurkat TRBC1+ve	1:4	Donor 5	KFN_CD8STK_28z
2E	Jurkat TRBC1+ve	1:4	Donor 5	KFN_CD28STK_CD28TM_28z
2F	Jurkat TRBC1+ve	1:4	Donor 5	aCD19-CAR
2G	Jurkat TRBC1+ve	1:4	Donor 5	Non-transduced
2H	Jurkat TRBC1+ve	N/A	N/A	N/A
3B	Jurkat TRBC1+ve	1:4	Donor 6	KFN_Hinge_28z
3D	Jurkat TRBC1+ve	1:4	Donor 6	KFN_CD8STK_28z
3E	Jurkat TRBC1+ve	1:4	Donor 6	KFN_CD28STK_CD28TM_28z
3F	Jurkat TRBC1+ve	1:4	Donor 6	aCD19-CAR
3G	Jurkat TRBC1+ve	1:4	Donor 6	Non-transduced
3H	Jurkat TRBC1+ve	N/A	N/A	N/A
4B	Jurkat TRBC2+ve	1:4	Donor 4	KFN_Hinge_28z
4D	Jurkat TRBC2+ve	1:4	Donor 4	KFN_CD8STK_28z
4E	Jurkat TRBC2+ve	1:4	Donor 4	KFN_CD28STK_CD28TM_28z
4F	Jurkat TRBC2+ve	1:4	Donor 4	aCD19-CAR
4G	Jurkat TRBC2+ve	1:4	Donor 4	Non-transduced
4H	Jurkat TRBC2+ve	N/A	N/A	N/A
5B	Jurkat TRBC2+ve	1:4	Donor 5	KFN_Hinge_28z
5D	Jurkat TRBC2+ve	1:4	Donor 5	KFN_CD8STK_28z
5E	Jurkat TRBC2+ve	1:4	Donor 5	KFN_CD28STK_CD28TM_28z
5F	Jurkat TRBC2+ve	1:4	Donor 5	aCD19-CAR
5G	Jurkat TRBC2+ve	1:4	Donor 5	Non-transduced
5H	Jurkat TRBC2+ve	N/A	N/A	N/A
6B	Jurkat TRBC2+ve	1:4	Donor 6	KFN_Hinge_28z
6D	Jurkat TRBC2+ve	1:4	Donor 6	KFN_CD8STK_28z
6E	Jurkat TRBC2+ve	1:4	Donor 6	KFN_CD28STK_CD28TM_28z
6F	Jurkat TRBC2+ve	1:4	Donor 6	aCD19-CAR
6G	Jurkat TRBC2+ve	1:4	Donor 6	Non-transduced
6H	Jurkat TRBC2+ve	N/A	N/A	N/A
7B	Jurkat TRBC KO	1:4	Donor 4	KFN_Hinge_28z
7D	Jurkat TRBC KO	1:4	Donor 4	KFN_CD8STK_28z
7E	Jurkat TRBC KO	1:4	Donor 4	KFN_CD28STK_CD28TM_28z
7F	Jurkat TRBC KO	1:4	Donor 4	aCD19-CAR
7G	Jurkat TRBC KO	1:4	Donor 4	Non-transduced
7H	Jurkat TRBC KO	N/A	N/A	N/A
8B	Jurkat TRBC KO	1:4	Donor 5	KFN_Hinge_28z
8D	Jurkat TRBC KO	1:4	Donor 5	KFN_CD8STK_28z
8E	Jurkat TRBC KO	1:4	Donor 5	KFN_CD28STK_CD28TM_28z
8F	Jurkat TRBC KO	1:4	Donor 5	aCD19-CAR
8G	Jurkat TRBC KO	1:4	Donor 5	Non-transduced
8H	Jurkat TRBC KO	N/A	N/A	N/A

**9B**	Jurkat TRBC KO	1:4	Donor 6	KFN_Hinge_28z
**9D**	Jurkat TRBC KO	1:4	Donor 6	KFN_CD8STK_28z
**9E**	Jurkat TRBC KO	1:4	Donor 6	KFN_CD28STK_CD28TM_28z
**9F**	Jurkat TRBC KO	1:4	Donor 6	aCD19-CAR
**9G**	Jurkat TRBC KO	1:4	Donor 6	Non-transduced
**9H**	Jurkat TRBC KO	N/A	N/A	N/A
**10A**	N/A	N/A	N/A	N/A
**10B**	N/A	N/A	N/A	N/A
**10C**	N/A	N/A	N/A	N/A
**10D**	N/A	N/A	N/A	N/A
**10E**	N/A	N/A	N/A	N/A
**10F**	N/A	N/A	N/A	N/A
**10G**	N/A	N/A	N/A	N/A
**10H**	N/A	N/A	N/A	N/A
**11A**	N/A	N/A	N/A	N/A
**11B**	N/A	N/A	N/A	N/A
**11C**	N/A	N/A	N/A	N/A
**11D**	N/A	N/A	N/A	N/A
**11E**	N/A	N/A	N/A	N/A
**11F**	N/A	N/A	N/A	N/A
**11G**	N/A	N/A	N/A	N/A
**11H**	N/A	N/A	N/A	N/A
**12A**	N/A	N/A	N/A	N/A
**12B**	N/A	N/A	N/A	N/A
**12C**	N/A	N/A	N/A	N/A
**12D**	N/A	N/A	N/A	N/A
**12E**	N/A	N/A	N/A	N/A
**12F**	N/A	N/A	N/A	N/A
**12G**	N/A	N/A	N/A	N/A
**12H**	N/A	N/A	N/A	N/A

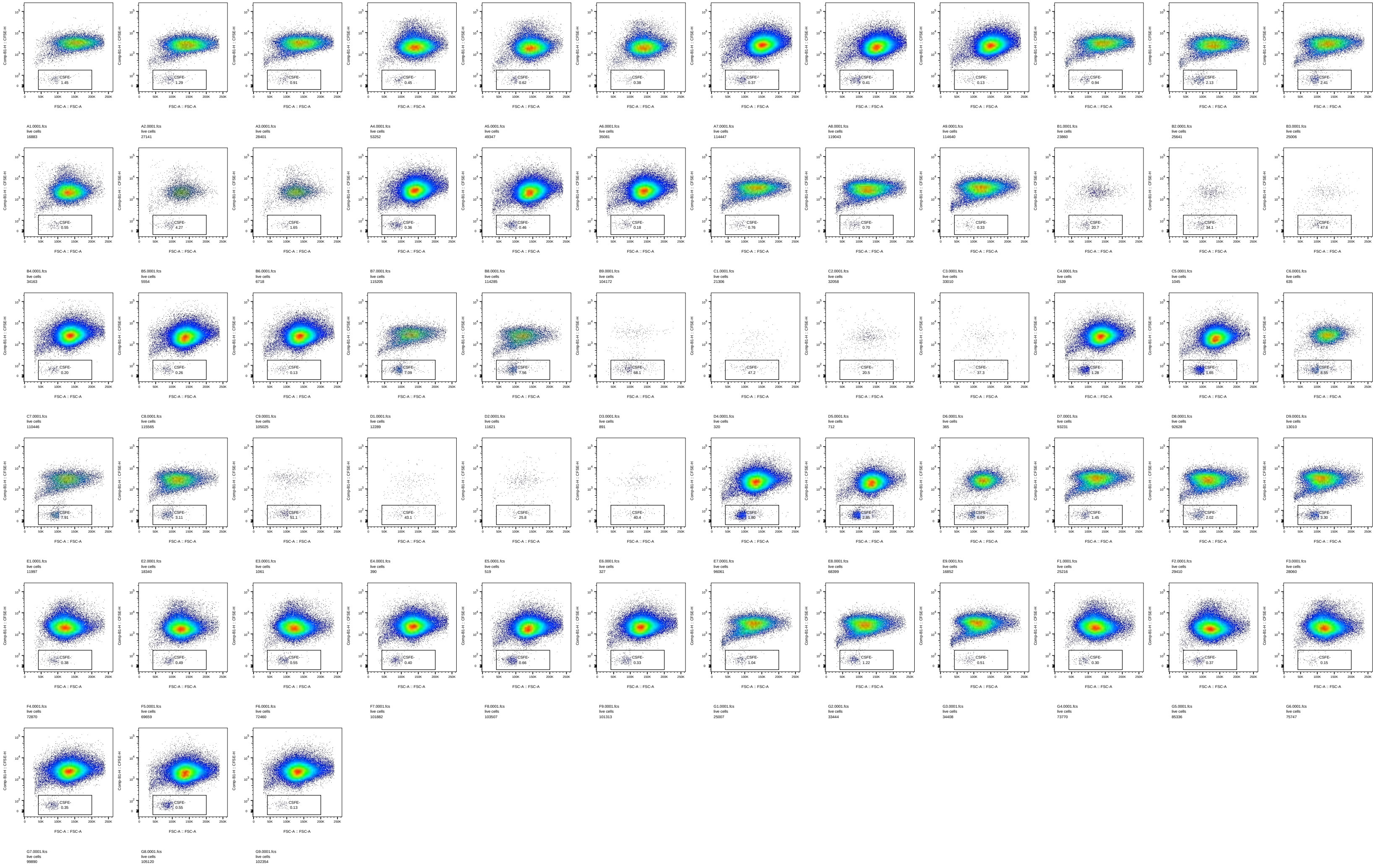

Comp-B1-H :: CFSE-H
FSC-A :: FSC-A
CSFE- 1.45
A1.0001.fcs live cells 16883
CSFE- 1.29
A2.0001.fcs live cells 27141
CSFE- 0.91
A3.0001.fcs live cells 28401
CSFE- 0.45
A4.0001.fcs live cells 53252
CSFE- 0.62
A5.0001.fcs live cells 49347
CSFE- 0.38
A6.0001.fcs live cells 35081
CSFE- 0.37
A7.0001.fcs live cells 114447
CSFE- 0.41
A8.0001.fcs live cells 119043
CSFE- 0.13
A9.0001.fcs live cells 114640
CSFE- 0.94
B1.0001.fcs live cells 23860
CSFE- 2.13
B2.0001.fcs live cells 25641
CSFE- 2.41
B3.0001.fcs live cells 25006
CSFE- 0.55
B4.0001.fcs live cells 34163
CSFE- 4.27
B5.0001.fcs live cells 5554
CSFE- 1.65
B6.0001.fcs live cells 6718
CSFE- 0.36
B7.0001.fcs live cells 115205
CSFE- 0.46
B8.0001.fcs live cells 114285
CSFE- 0.18
B9.0001.fcs live cells 104172
CSFE- 0.76
C1.0001.fcs live cells 21306
CSFE- 0.70
C2.0001.fcs live cells 32058
CSFE- 0.33
C3.0001.fcs live cells 33010
CSFE- 20.7
C4.0001.fcs live cells 1539
CSFE- 34.1
C5.0001.fcs live cells 1045
CSFE- 47.6
C6.0001.fcs live cells 635
CSFE- 0.20
C7.0001.fcs live cells 110446
CSFE- 0.26
C8.0001.fcs live cells 115565
CSFE- 0.13
C9.0001.fcs live cells 105025
CSFE- 7.09
D1.0001.fcs live cells 12289
CSFE- 7.56
D2.0001.fcs live cells 11621
CSFE- 68.1
D3.0001.fcs live cells 891
CSFE- 47.2
D4.0001.fcs live cells 320
CSFE- 20.5
D5.0001.fcs live cells 712
CSFE- 37.3
D6.0001.fcs live cells 365
CSFE- 1.28
D7.0001.fcs live cells 93231
CSFE- 1.65
D8.0001.fcs live cells 92628
CSFE- 8.55
D9.0001.fcs live cells 13010
CSFE- 7.91
E1.0001.fcs live cells 11997
CSFE- 3.11
E2.0001.fcs live cells 18340
CSFE- 51.1
E3.0001.fcs live cells 1061
CSFE- 43.1
E4.0001.fcs live cells 390
CSFE- 25.8
E5.0001.fcs live cells 519
CSFE- 40.4
E6.0001.fcs live cells 327
CSFE- 1.80
E7.0001.fcs live cells 96061
CSFE- 2.85
E8.0001.fcs live cells 68399
CSFE- 6.09
E9.0001.fcs live cells 16852
CSFE- 1.45
F1.0001.fcs live cells 25216
CSFE- 2.02
F2.0001.fcs live cells 29410
CSFE- 3.30
F3.0001.fcs live cells 28060
CSFE- 0.38
F4.0001.fcs live cells 72870
CSFE- 0.49
F5.0001.fcs live cells 69659
CSFE- 0.55
F6.0001.fcs live cells 72460
CSFE- 0.40
F7.0001.fcs live cells 101882
CSFE- 0.66
F8.0001.fcs live cells 103507
CSFE- 0.33
F9.0001.fcs live cells 101313
CSFE- 1.04
G1.0001.fcs live cells 25007
CSFE- 1.22
G2.0001.fcs live cells 33444
CSFE- 0.51
G3.0001.fcs live cells 34408
CSFE- 0.30
G4.0001.fcs live cells 73770
CSFE- 0.37
G5.0001.fcs live cells 85336
CSFE- 0.15
G6.0001.fcs live cells 75747
CSFE- 0.35
G7.0001.fcs live cells 99890
CSFE- 0.55
G8.0001.fcs live cells 105120
CSFE- 0.13
G9.0001.fcs live cells 102354

Plate20_KFN-Prol_vs_HPB

Well	Target	E:T ratio	Donor	CAR construct
1B	HPB TRBC1+ve	1:4	Donor 4	KFN_Hinge_28z
1D	HPB TRBC1+ve	1:4	Donor 4	KFN_CD8STK_28z
1E	HPB TRBC1+ve	1:4	Donor 4	KFN_CD28STK_CD28TM_28z
1F	HPB TRBC1+ve	1:4	Donor 4	aCD19-CAR
1G	HPB TRBC1+ve	1:4	Donor 4	Non-transduced
1H	HPB TRBC1+ve	N/A	N/A	N/A
2B	HPB TRBC1+ve	1:4	Donor 5	KFN_Hinge_28z
2D	HPB TRBC1+ve	1:4	Donor 5	KFN_CD8STK_28z
2E	HPB TRBC1+ve	1:4	Donor 5	KFN_CD28STK_CD28TM_28z
2F	HPB TRBC1+ve	1:4	Donor 5	aCD19-CAR
2G	HPB TRBC1+ve	1:4	Donor 5	Non-transduced
2H	HPB TRBC1+ve	N/A	N/A	N/A
3B	HPB TRBC1+ve	1:4	Donor 6	KFN_Hinge_28z
3D	HPB TRBC1+ve	1:4	Donor 6	KFN_CD8STK_28z
3E	HPB TRBC1+ve	1:4	Donor 6	KFN_CD28STK_CD28TM_28z
3F	HPB TRBC1+ve	1:4	Donor 6	aCD19-CAR
3G	HPB TRBC1+ve	1:4	Donor 6	Non-transduced
3H	HPB TRBC1+ve	N/A	N/A	N/A
4B	HPB TRBC2+ve	1:4	Donor 4	KFN_Hinge_28z
4D	HPB TRBC2+ve	1:4	Donor 4	KFN_CD8STK_28z
4E	HPB TRBC2+ve	1:4	Donor 4	KFN_CD28STK_CD28TM_28z
4F	HPB TRBC2+ve	1:4	Donor 4	aCD19-CAR
4G	HPB TRBC2+ve	1:4	Donor 4	Non-transduced
4H	HPB TRBC2+ve	N/A	N/A	N/A
5B	HPB TRBC2+ve	1:4	Donor 5	KFN_Hinge_28z
5D	HPB TRBC2+ve	1:4	Donor 5	KFN_CD8STK_28z
5E	HPB TRBC2+ve	1:4	Donor 5	KFN_CD28STK_CD28TM_28z
5F	HPB TRBC2+ve	1:4	Donor 5	aCD19-CAR
5G	HPB TRBC2+ve	1:4	Donor 5	Non-transduced
5H	HPB TRBC2+ve	N/A	N/A	N/A
6B	HPB TRBC2+ve	1:4	Donor 6	KFN_Hinge_28z
6D	HPB TRBC2+ve	1:4	Donor 6	KFN_CD8STK_28z
6E	HPB TRBC2+ve	1:4	Donor 6	KFN_CD28STK_CD28TM_28z
6F	HPB TRBC2+ve	1:4	Donor 6	aCD19-CAR
6G	HPB TRBC2+ve	1:4	Donor 6	Non-transduced
6H	HPB TRBC2+ve	N/A	N/A	N/A
7B	HPB TRBC KO	1:4	Donor 4	KFN_Hinge_28z
7D	HPB TRBC KO	1:4	Donor 4	KFN_CD8STK_28z
7E	HPB TRBC KO	1:4	Donor 4	KFN_CD28STK_CD28TM_28z
7F	HPB TRBC KO	1:4	Donor 4	aCD19-CAR
7G	HPB TRBC KO	1:4	Donor 4	Non-transduced
7H	HPB TRBC KO	N/A	N/A	N/A
8B	HPB TRBC KO	1:4	Donor 5	KFN_Hinge_28z
8D	HPB TRBC KO	1:4	Donor 5	KFN_CD8STK_28z
8E	HPB TRBC KO	1:4	Donor 5	KFN_CD28STK_CD28TM_28z
8F	HPB TRBC KO	1:4	Donor 5	aCD19-CAR
8G	HPB TRBC KO	1:4	Donor 5	Non-transduced
8H	HPB TRBC KO	N/A	N/A	N/A

**9B**	HPB TRBC KO	1:4	Donor 6	KFN_Hinge_28z
**9D**	HPB TRBC KO	1:4	Donor 6	KFN_CD8STK_28z
**9E**	HPB TRBC KO	1:4	Donor 6	KFN_CD28STK_CD28TM_28z
**9F**	HPB TRBC KO	1:4	Donor 6	aCD19-CAR
**9G**	HPB TRBC KO	1:4	Donor 6	Non-transduced
**9H**	HPB TRBC KO	N/A	N/A	N/A
**10A**	N/A	N/A	N/A	N/A
**10B**	N/A	N/A	N/A	N/A
**10C**	N/A	N/A	N/A	N/A
**10D**	N/A	N/A	N/A	N/A
**10E**	N/A	N/A	N/A	N/A
**10F**	N/A	N/A	N/A	N/A
**10G**	N/A	N/A	N/A	N/A
**10H**	N/A	N/A	N/A	N/A
**11A**	N/A	N/A	N/A	N/A
**11B**	N/A	N/A	N/A	N/A
**11C**	N/A	N/A	N/A	N/A
**11D**	N/A	N/A	N/A	N/A
**11E**	N/A	N/A	N/A	N/A
**11F**	N/A	N/A	N/A	N/A
**11G**	N/A	N/A	N/A	N/A
**11H**	N/A	N/A	N/A	N/A
**12A**	N/A	N/A	N/A	N/A
**12B**	N/A	N/A	N/A	N/A
**12C**	N/A	N/A	N/A	N/A
**12D**	N/A	N/A	N/A	N/A
**12E**	N/A	N/A	N/A	N/A
**12F**	N/A	N/A	N/A	N/A
**12G**	N/A	N/A	N/A	N/A
**12H**	N/A	N/A	N/A	N/A

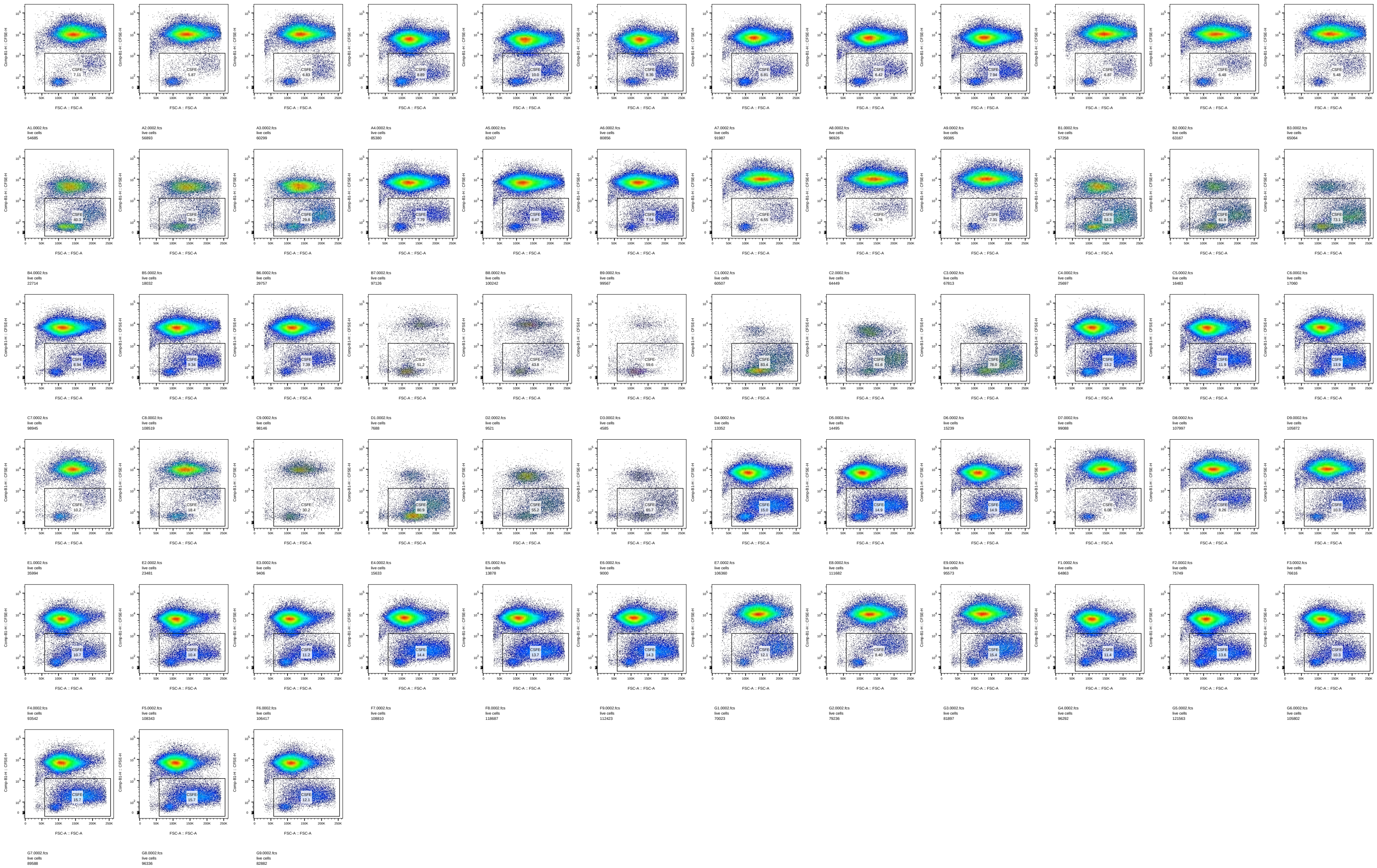
CSFE 7.11
A1.0002.fcs
live cells
54685
CSFE 5.87
A2.0002.fcs
live cells
56893
CSFE 6.83
A3.0002.fcs
live cells
60299
CSFE 8.89
A4.0002.fcs
live cells
85380
CSFE 10.0
A5.0002.fcs
live cells
82437
CSFE 8.35
A6.0002.fcs
live cells
80856
CSFE 6.81
A7.0002.fcs
live cells
91987
CSFE 6.42
A8.0002.fcs
live cells
96926
CSFE 7.94
A9.0002.fcs
live cells
99385
CSFE 6.87
B1.0002.fcs
live cells
57258
CSFE 6.48
B2.0002.fcs
live cells
63167
CSFE 5.48
B3.0002.fcs
live cells
65064
CSFE 40.3
B4.0002.fcs
live cells
22714
CSFE 36.2
B5.0002.fcs
live cells
18032
CSFE 29.8
B6.0002.fcs
live cells
29757
CSFE 7.79
B7.0002.fcs
live cells
97126
CSFE 8.47
B8.0002.fcs
live cells
100242
CSFE 7.54
B9.0002.fcs
live cells
99567
CSFE 6.55
C1.0002.fcs
live cells
60507
CSFE 4.76
C2.0002.fcs
live cells
64449
CSFE 7.31
C3.0002.fcs
live cells
67813
CSFE 53.3
C4.0002.fcs
live cells
25697
CSFE 61.9
C5.0002.fcs
live cells
16483
CSFE 73.1
C6.0002.fcs
live cells
17060
CSFE 8.94
C7.0002.fcs
live cells
98945
CSFE 9.34
C8.0002.fcs
live cells
108519
CSFE 7.39
C9.0002.fcs
live cells
98146
CSFE 51.2
D1.0002.fcs
live cells
7688
CSFE 43.8
D2.0002.fcs
live cells
9521
CSFE 59.6
D3.0002.fcs
live cells
4585
CSFE 83.4
D4.0002.fcs
live cells
13352
CSFE 61.6
D5.0002.fcs
live cells
14495
CSFE 78.0
D6.0002.fcs
live cells
15239
CSFE 13.2
D7.0002.fcs
live cells
99088
CSFE 11.9
D8.0002.fcs
live cells
107997
CSFE 13.9
D9.0002.fcs
live cells
105872
CSFE 10.2
E1.0002.fcs
live cells
35994
CSFE 18.4
E2.0002.fcs
live cells
23481
CSFE 30.2
E3.0002.fcs
live cells
9406
CSFE 80.9
E4.0002.fcs
live cells
15633
CSFE 55.2
E5.0002.fcs
live cells
13878
CSFE 65.7
E6.0002.fcs
live cells
9000
CSFE 15.0
E7.0002.fcs
live cells
106360
CSFE 14.9
E8.0002.fcs
live cells
111682
CSFE 14.9
E9.0002.fcs
live cells
95573
CSFE 6.08
F1.0002.fcs
live cells
64863
CSFE 8.26
F2.0002.fcs
live cells
75749
CSFE 10.3
F3.0002.fcs
live cells
76616
CSFE 10.7
F4.0002.fcs
live cells
93542
CSFE 10.4
F5.0002.fcs
live cells
108343
CSFE 11.2
F6.0002.fcs
live cells
106417
CSFE 14.4
F7.0002.fcs
live cells
108810
CSFE 13.7
F8.0002.fcs
live cells
118687
CSFE 14.3
F9.0002.fcs
live cells
112423
CSFE 12.1
G1.0002.fcs
live cells
70023
CSFE 8.40
G2.0002.fcs
live cells
79236
CSFE 15.4
G3.0002.fcs
live cells
81897
CSFE 11.4
G4.0002.fcs
live cells
96292
CSFE 13.6
G5.0002.fcs
live cells
121563
CSFE 10.3
G6.0002.fcs
live cells
105802
CSFE 15.7
G7.0002.fcs
live cells
89588
CSFE 15.7
G8.0002.fcs
live cells
96336
CSFE 12.1
G9.0002.fcs
live cells
82882

Plate21_KFN-Prol_vs_H9_TALL_PBM

Well	Target	E:T ratio	Donor	CAR construct
1B	H9	1:4	Donor 4	KFN_Hinge_28z
1D	H9	1:4	Donor 4	KFN_CD8STK_28z
1E	H9	1:4	Donor 4	KFN_CD28STK_CD28TM_28z
1F	H9	1:4	Donor 4	aCD19-CAR
1G	H9	1:4	Donor 4	Non-transduced
1H	H9	N/A	N/A	N/A
2B	H9	1:4	Donor 5	KFN_Hinge_28z
2D	H9	1:4	Donor 5	KFN_CD8STK_28z
2E	H9	1:4	Donor 5	KFN_CD28STK_CD28TM_28z
2F	H9	1:4	Donor 5	aCD19-CAR
2G	H9	1:4	Donor 5	Non-transduced
2H	H9	N/A	N/A	N/A
3B	H9	1:4	Donor 6	KFN_Hinge_28z
3D	H9	1:4	Donor 6	KFN_CD8STK_28z
3E	H9	1:4	Donor 6	KFN_CD28STK_CD28TM_28z
3F	H9	1:4	Donor 6	aCD19-CAR
3G	H9	1:4	Donor 6	Non-transduced
3H	H9	N/A	N/A	N/A
4B	T-ALL1	1:4	Donor 4	KFN_Hinge_28z
4D	T-ALL1	1:4	Donor 4	KFN_CD8STK_28z
4E	T-ALL1	1:4	Donor 4	KFN_CD28STK_CD28TM_28z
4F	T-ALL1	1:4	Donor 4	aCD19-CAR
4G	T-ALL1	1:4	Donor 4	Non-transduced
4H	T-ALL1	N/A	N/A	N/A
5B	T-ALL1	1:4	Donor 5	KFN_Hinge_28z
5D	T-ALL1	1:4	Donor 5	KFN_CD8STK_28z
5E	T-ALL1	1:4	Donor 5	KFN_CD28STK_CD28TM_28z
5F	T-ALL1	1:4	Donor 5	aCD19-CAR
5G	T-ALL1	1:4	Donor 5	Non-transduced
5H	T-ALL1	N/A	N/A	N/A
6B	T-ALL1	1:4	Donor 6	KFN_Hinge_28z
6D	T-ALL1	1:4	Donor 6	KFN_CD8STK_28z
6E	T-ALL1	1:4	Donor 6	KFN_CD28STK_CD28TM_28z
6F	T-ALL1	1:4	Donor 6	aCD19-CAR
6G	T-ALL1	1:4	Donor 6	Non-transduced
6H	T-ALL1	N/A	N/A	N/A

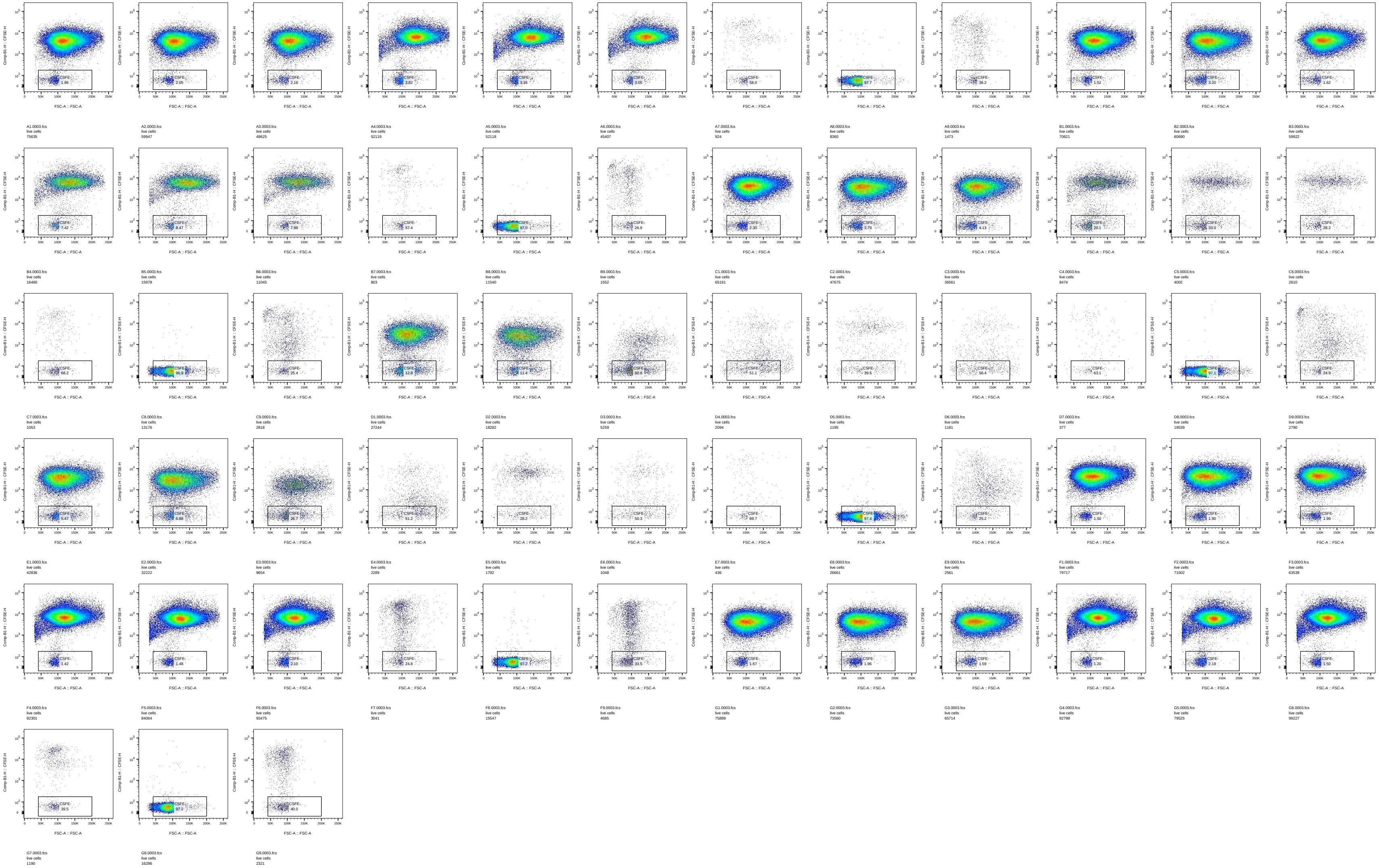
A1.0003.fcs
live cells
75635
A2.0003.fcs
live cells
59947
A3.0003.fcs
live cells
49625
A4.0003.fcs
live cells
52119
A5.0003.fcs
live cells
52118
A6.0003.fcs
live cells
45407
A7.0003.fcs
live cells
924
A8.0003.fcs
live cells
8360
A9.0003.fcs
live cells
1473
B1.0003.fcs
live cells
70621
B2.0003.fcs
live cells
60690
B3.0003.fcs
live cells
59922
B4.0003.fcs
live cells
16460
B5.0003.fcs
live cells
15978
B6.0003.fcs
live cells
11045
B7.0003.fcs
live cells
803
B8.0003.fcs
live cells
11540
B9.0003.fcs
live cells
1552
C1.0003.fcs
live cells
65161
C2.0003.fcs
live cells
47675
C3.0003.fcs
live cells
36561
C4.0003.fcs
live cells
8474
C5.0003.fcs
live cells
4002
C6.0003.fcs
live cells
2610
C7.0003.fcs
live cells
1053
C8.0003.fcs
live cells
13176
C9.0003.fcs
live cells
2818
D1.0003.fcs
live cells
27244
D2.0003.fcs
live cells
18282
D3.0003.fcs
live cells
5259
D4.0003.fcs
live cells
2094
D5.0003.fcs
live cells
1195
D6.0003.fcs
live cells
1181
D7.0003.fcs
live cells
377
D8.0003.fcs
live cells
19539
D9.0003.fcs
live cells
2790
E1.0003.fcs
live cells
42836
E2.0003.fcs
live cells
32222
E3.0003.fcs
live cells
9654
E4.0003.fcs
live cells
2289
E5.0003.fcs
live cells
1792
E6.0003.fcs
live cells
1048
E7.0003.fcs
live cells
436
E8.0003.fcs
live cells
26661
E9.0003.fcs
live cells
2561
F1.0003.fcs
live cells
79717
F2.0003.fcs
live cells
71502
F3.0003.fcs
live cells
63539
F4.0003.fcs
live cells
92301
F5.0003.fcs
live cells
84064
F6.0003.fcs
live cells
93475
F7.0003.fcs
live cells
3041
F8.0003.fcs
live cells
15547
F9.0003.fcs
live cells
4685
G1.0003.fcs
live cells
75889
G2.0003.fcs
live cells
73560
G3.0003.fcs
live cells
65714
G4.0003.fcs
live cells
92798
G5.0003.fcs
live cells
79525
G6.0003.fcs
live cells
99227
G7.0003.fcs
live cells
1190
G8.0003.fcs
live cells
16286
G9.0003.fcs
live cells
2321

Plate1_KFN_vs_Jurkat_TRBC1

Well	Target	E:T ratio	Donor	CAR construct
**1B**	Jurkat TRBC 1+ve	1:4	Donor 4	KFN_Hinge_28z
**1D**	Jurkat TRBC 1+ve	1:4	Donor 4	KFN_CD8STK_28z
**1E**	Jurkat TRBC 1+ve	1:4	Donor 4	KFN_CD28STK_CD28TM_28z
**1F**	Jurkat TRBC 1+ve	1:4	Donor 4	aCD19-CAR
**1G**	Jurkat TRBC 1+ve	1:4	Donor 4	Non-transduced
**1H**	Jurkat TRBC 1+ve	N/A	N/A	N/A
**2B**	Jurkat TRBC 1+ve	1:8	Donor 4	KFN_Hinge_28z
**2D**	Jurkat TRBC 1+ve	1:8	Donor 4	KFN_CD8STK_28z
**2E**	Jurkat TRBC 1+ve	1:8	Donor 4	KFN_CD28STK_CD28TM_28z
**2F**	Jurkat TRBC 1+ve	1:8	Donor 4	aCD19-CAR
**2G**	Jurkat TRBC 1+ve	1:8	Donor 4	Non-transduced
**2H**	Jurkat TRBC 1+ve	N/A	N/A	N/A
**5B**	Jurkat TRBC 1+ve	1:4	Donor 5	KFN_Hinge_28z
**5D**	Jurkat TRBC 1+ve	1:4	Donor 5	KFN_CD8STK_28z
**5E**	Jurkat TRBC 1+ve	1:4	Donor 5	KFN_CD28STK_CD28TM_28z
**5F**	Jurkat TRBC 1+ve	1:4	Donor 5	aCD19-CAR
**5G**	Jurkat TRBC 1+ve	1:4	Donor 5	Non-transduced
**5H**	Jurkat TRBC 1+ve	N/A	N/A	N/A
**6B**	Jurkat TRBC 1+ve	1:8	Donor 5	KFN_Hinge_28z
**6D**	Jurkat TRBC 1+ve	1:8	Donor 5	KFN_CD8STK_28z
**6E**	Jurkat TRBC 1+ve	1:8	Donor 5	KFN_CD28STK_CD28TM_28z
**6F**	Jurkat TRBC 1+ve	1:8	Donor 5	aCD19-CAR
**6G**	Jurkat TRBC 1+ve	1:8	Donor 5	Non-transduced
**6H**	Jurkat TRBC 1+ve	N/A	N/A	N/A
**9B**	Jurkat TRBC 1+ve	1:4	Donor 6	KFN_Hinge_28z
**9D**	Jurkat TRBC 1+ve	1:4	Donor 6	KFN_CD8STK_28z
**9E**	Jurkat TRBC 1+ve	1:4	Donor 6	KFN_CD28STK_CD28TM_28z
**9F**	Jurkat TRBC 1+ve	1:4	Donor 6	aCD19-CAR
**9G**	Jurkat TRBC 1+ve	1:4	Donor 6	Non-transduced
**9H**	Jurkat TRBC 1+ve	N/A	N/A	N/A
**10B**	Jurkat TRBC 1+ve	1:8	Donor 6	KFN_Hinge_28z
**10D**	Jurkat TRBC 1+ve	1:8	Donor 6	KFN_CD8STK_28z
**10E**	Jurkat TRBC 1+ve	1:8	Donor 6	KFN_CD28STK_CD28TM_28z
**10F**	Jurkat TRBC 1+ve	1:8	Donor 6	aCD19-CAR
**10G**	Jurkat TRBC 1+ve	1:8	Donor 6	Non-transduced
**10H**	Jurkat TRBC 1+ve	N/A	N/A	N/A

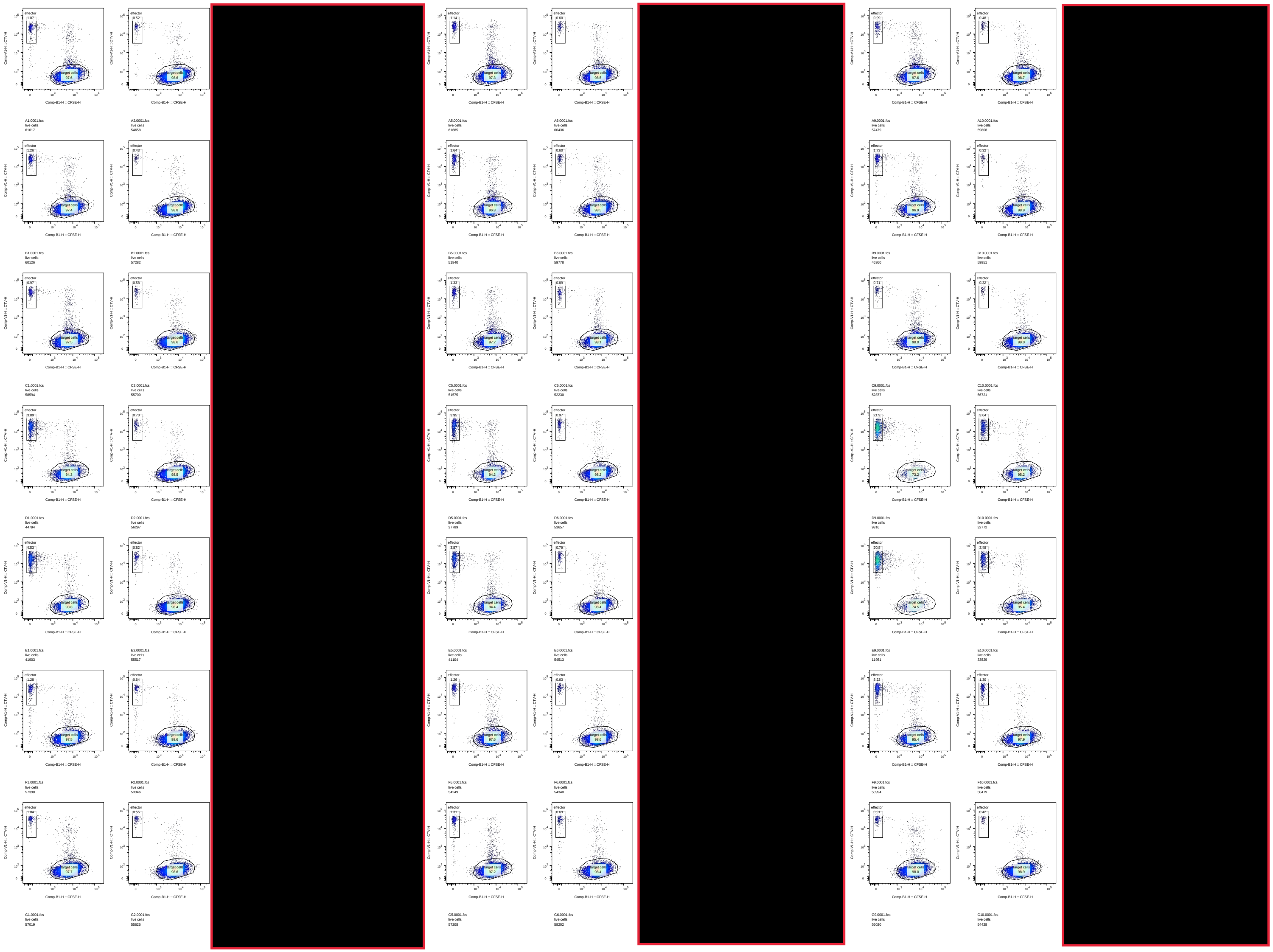

Comp-V1-H :: CTV-H
Comp-B1-H :: CFSE-H
effector 1.07
target cells 97.6
A1.0001.fcs
live cells
61017
effector 0.52
target cells 98.6
A2.0001.fcs
live cells
54658
effector 1.26
target cells 97.4
B1.0001.fcs
live cells
60126
effector 0.43
target cells 98.8
B2.0001.fcs
live cells
57282
effector 0.97
target cells 97.5
C1.0001.fcs
live cells
58594
effector 0.58
target cells 98.6
C2.0001.fcs
live cells
55700
effector 3.89
target cells 94.3
D1.0001.fcs
live cells
44794
effector 0.70
target cells 98.5
D2.0001.fcs
live cells
56297
effector 4.53
target cells 93.8
E1.0001.fcs
live cells
41903
effector 0.82
target cells 98.4
E2.0001.fcs
live cells
55517
effector 1.28
target cells 97.5
F1.0001.fcs
live cells
57398
effector 0.64
target cells 98.6
F2.0001.fcs
live cells
53346
effector 1.04
target cells 97.7
G1.0001.fcs
live cells
57019
effector 0.55
target cells 98.6
G2.0001.fcs
live cells
55626
[redacted]
effector 1.14
target cells 97.3
A5.0001.fcs
live cells
61685
effector 0.60
target cells 98.5
A6.0001.fcs
live cells
60436
effector 1.64
target cells 96.8
B5.0001.fcs
live cells
51840
effector 0.60
target cells 98.5
B6.0001.fcs
live cells
59778
effector 1.33
target cells 97.2
C5.0001.fcs
live cells
51575
effector 0.89
target cells 98.1
C6.0001.fcs
live cells
52230
effector 3.95
target cells 94.2
D5.0001.fcs
live cells
37789
effector 0.97
target cells 98.2
D6.0001.fcs
live cells
53657
effector 3.87
target cells 94.4
E5.0001.fcs
live cells
41104
effector 0.79
target cells 98.4
E6.0001.fcs
live cells
54513
effector 1.26
target cells 97.6
F5.0001.fcs
live cells
54249
effector 0.63
target cells 98.6
F6.0001.fcs
live cells
54340
effector 1.31
target cells 97.2
G5.0001.fcs
live cells
57208
effector 0.69
target cells 98.4
G6.0001.fcs
live cells
58202
[redacted]
effector 0.99
target cells 97.6
A9.0001.fcs
live cells
57479
effector 0.48
target cells 98.7
A10.0001.fcs
live cells
59808
effector 1.73
target cells 96.9
B9.0001.fcs
live cells
46360
effector 0.32
target cells 98.9
B10.0001.fcs
live cells
59851
effector 0.71
target cells 98.0
C9.0001.fcs
live cells
52877
effector 0.32
target cells 99.0
C10.0001.fcs
live cells
56721
effector 21.9
target cells 73.2
D9.0001.fcs
live cells
9816
effector 3.64
target cells 95.2
D10.0001.fcs
live cells
32772
effector 20.8
target cells 74.5
E9.0001.fcs
live cells
11951
effector 3.48
target cells 95.4
E10.0001.fcs
live cells
33529
effector 3.22
target cells 95.4
F9.0001.fcs
live cells
50994
effector 1.30
target cells 97.9
F10.0001.fcs
live cells
50479
effector 0.91
target cells 98.0
G9.0001.fcs
live cells
56020
effector 0.42
target cells 98.9
G10.0001.fcs
live cells
54428
[redacted]

Plate2_KFN_vs_Jurkat_TRBC2

Well	Target	E:T ratio	Donor	CAR construct
**1B**	Jurkat TRBC 2+ve	1:4	Donor 4	KFN_Hinge_28z
**1D**	Jurkat TRBC 2+ve	1:4	Donor 4	KFN_CD8STK_28z
**1E**	Jurkat TRBC 2+ve	1:4	Donor 4	KFN_CD28STK_CD28TM_28z
**1F**	Jurkat TRBC 2+ve	1:4	Donor 4	aCD19-CAR
**1G**	Jurkat TRBC 2+ve	1:4	Donor 4	Non-transduced
**1H**	Jurkat TRBC 2+ve	N/A	N/A	N/A
**2B**	Jurkat TRBC 2+ve	1:8	Donor 4	KFN_Hinge_28z
**2D**	Jurkat TRBC 2+ve	1:8	Donor 4	KFN_CD8STK_28z
**2E**	Jurkat TRBC 2+ve	1:8	Donor 4	KFN_CD28STK_CD28TM_28z
**2F**	Jurkat TRBC 2+ve	1:8	Donor 4	aCD19-CAR
**2G**	Jurkat TRBC 2+ve	1:8	Donor 4	Non-transduced
**2H**	Jurkat TRBC 2+ve	N/A	N/A	N/A
**5B**	Jurkat TRBC 2+ve	1:4	Donor 5	KFN_Hinge_28z
**5D**	Jurkat TRBC 2+ve	1:4	Donor 5	KFN_CD8STK_28z
**5E**	Jurkat TRBC 2+ve	1:4	Donor 5	KFN_CD28STK_CD28TM_28z
**5F**	Jurkat TRBC 2+ve	1:4	Donor 5	aCD19-CAR
**5G**	Jurkat TRBC 2+ve	1:4	Donor 5	Non-transduced
**5H**	Jurkat TRBC 2+ve	N/A	N/A	N/A
**6B**	Jurkat TRBC 2+ve	1:8	Donor 5	KFN_Hinge_28z
**6D**	Jurkat TRBC 2+ve	1:8	Donor 5	KFN_CD8STK_28z
**6E**	Jurkat TRBC 2+ve	1:8	Donor 5	KFN_CD28STK_CD28TM_28z
**6F**	Jurkat TRBC 2+ve	1:8	Donor 5	aCD19-CAR
**6G**	Jurkat TRBC 2+ve	1:8	Donor 5	Non-transduced
**6H**	Jurkat TRBC 2+ve	N/A	N/A	N/A
**9B**	Jurkat TRBC 2+ve	1:4	Donor 6	KFN_Hinge_28z
**9D**	Jurkat TRBC 2+ve	1:4	Donor 6	KFN_CD8STK_28z
**9E**	Jurkat TRBC 2+ve	1:4	Donor 6	KFN_CD28STK_CD28TM_28z
**9F**	Jurkat TRBC 2+ve	1:4	Donor 6	aCD19-CAR
**9G**	Jurkat TRBC 2+ve	1:4	Donor 6	Non-transduced
**9H**	Jurkat TRBC 2+ve	N/A	N/A	N/A
**10B**	Jurkat TRBC 2+ve	1:8	Donor 6	KFN_Hinge_28z
**10D**	Jurkat TRBC 2+ve	1:8	Donor 6	KFN_CD8STK_28z
**10E**	Jurkat TRBC 2+ve	1:8	Donor 6	KFN_CD28STK_CD28TM_28z
**10F**	Jurkat TRBC 2+ve	1:8	Donor 6	aCD19-CAR
**10G**	Jurkat TRBC 2+ve	1:8	Donor 6	Non-transduced
**10H**	Jurkat TRBC 2+ve	N/A	N/A	N/A

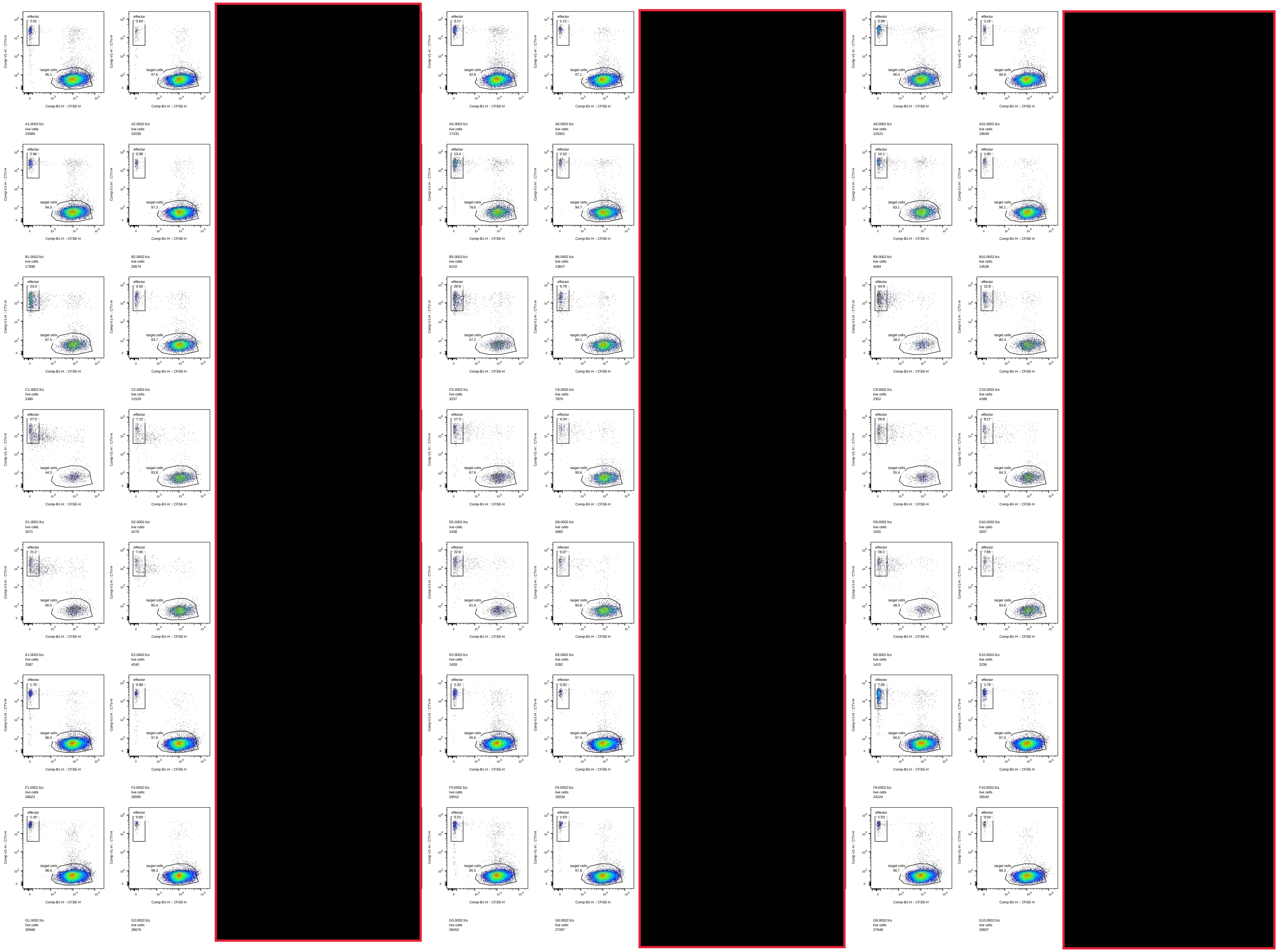

Plate3_KFN_vs_Jurkat_TRBC-KO

Well	Target	E:T ratio	Donor	CAR construct
**1B**	Jurkat TRBC KO	1:4	Donor 4	KFN_Hinge_28z
**1D**	Jurkat TRBC KO	1:4	Donor 4	KFN_CD8STK_28z
**1E**	Jurkat TRBC KO	1:4	Donor 4	KFN_CD28STK_CD28TM_28z
**1F**	Jurkat TRBC KO	1:4	Donor 4	aCD19-CAR
**1G**	Jurkat TRBC KO	1:4	Donor 4	Non-transduced
**1H**	Jurkat TRBC KO	N/A	N/A	N/A
**2B**	Jurkat TRBC KO	1:8	Donor 4	KFN_Hinge_28z
**2D**	Jurkat TRBC KO	1:8	Donor 4	KFN_CD8STK_28z
**2E**	Jurkat TRBC KO	1:8	Donor 4	KFN_CD28STK_CD28TM_28z
**2F**	Jurkat TRBC KO	1:8	Donor 4	aCD19-CAR
**2G**	Jurkat TRBC KO	1:8	Donor 4	Non-transduced
**2H**	Jurkat TRBC KO	N/A	N/A	N/A
**5B**	Jurkat TRBC KO	1:4	Donor 5	KFN_Hinge_28z
**5D**	Jurkat TRBC KO	1:4	Donor 5	KFN_CD8STK_28z
**5E**	Jurkat TRBC KO	1:4	Donor 5	KFN_CD28STK_CD28TM_28z
**5F**	Jurkat TRBC KO	1:4	Donor 5	aCD19-CAR
**5G**	Jurkat TRBC KO	1:4	Donor 5	Non-transduced
**5H**	Jurkat TRBC KO	N/A	N/A	N/A
**6B**	Jurkat TRBC KO	1:8	Donor 5	KFN_Hinge_28z
**6D**	Jurkat TRBC KO	1:8	Donor 5	KFN_CD8STK_28z
**6E**	Jurkat TRBC KO	1:8	Donor 5	KFN_CD28STK_CD28TM_28z
**6F**	Jurkat TRBC KO	1:8	Donor 5	aCD19-CAR
**6G**	Jurkat TRBC KO	1:8	Donor 5	Non-transduced
**6H**	Jurkat TRBC KO	N/A	N/A	N/A
**9B**	Jurkat TRBC KO	1:4	Donor 6	KFN_Hinge_28z
**9D**	Jurkat TRBC KO	1:4	Donor 6	KFN_CD8STK_28z
**9E**	Jurkat TRBC KO	1:4	Donor 6	KFN_CD28STK_CD28TM_28z
**9F**	Jurkat TRBC KO	1:4	Donor 6	aCD19-CAR
**9G**	Jurkat TRBC KO	1:4	Donor 6	Non-transduced
**9H**	Jurkat TRBC KO	N/A	N/A	N/A
**10B**	Jurkat TRBC KO	1:8	Donor 6	KFN_Hinge_28z
**10D**	Jurkat TRBC KO	1:8	Donor 6	KFN_CD8STK_28z
**10E**	Jurkat TRBC KO	1:8	Donor 6	KFN_CD28STK_CD28TM_28z
**10F**	Jurkat TRBC KO	1:8	Donor 6	aCD19-CAR
**10G**	Jurkat TRBC KO	1:8	Donor 6	Non-transduced
**10H**	Jurkat TRBC KO	N/A	N/A	N/A

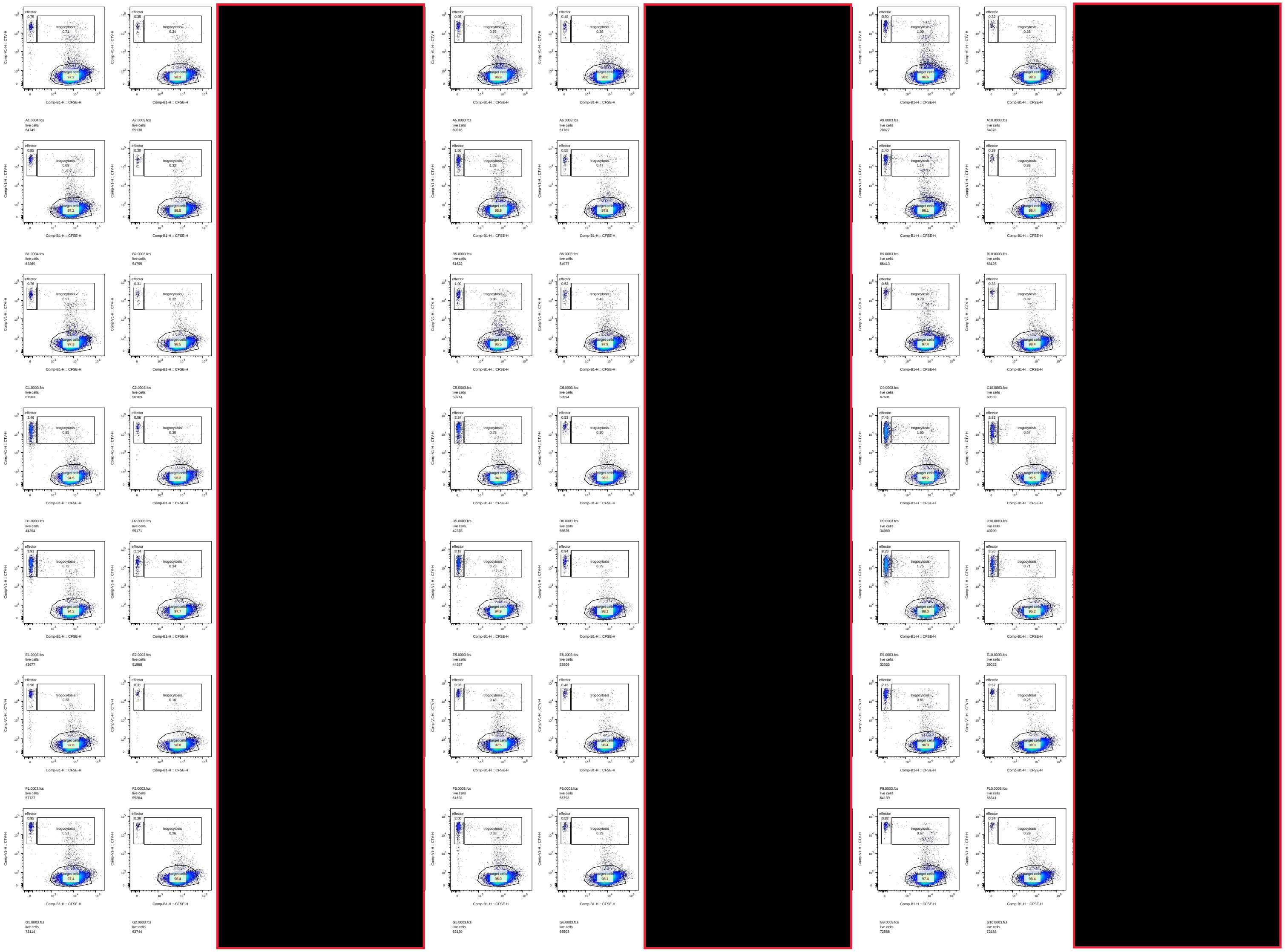
Comp-V1-H :: CTV-H
Comp-B1-H :: CFSE-H
effector
trogocytosis
target cells
A1.0004.fcs live cells 64749
A2.0003.fcs live cells 55130
A5.0003.fcs live cells 60316
A6.0003.fcs live cells 61762
A9.0003.fcs live cells 78877
A10.0003.fcs live cells 64078
B1.0004.fcs live cells 63269
B2.0003.fcs live cells 54795
B5.0003.fcs live cells 51622
B6.0003.fcs live cells 54977
B9.0003.fcs live cells 66413
B10.0003.fcs live cells 63125
C1.0003.fcs live cells 61963
C2.0003.fcs live cells 56169
C5.0003.fcs live cells 53714
C6.0003.fcs live cells 58594
C9.0003.fcs live cells 67601
C10.0003.fcs live cells 60559
D1.0003.fcs live cells 44394
D2.0003.fcs live cells 55171
D5.0003.fcs live cells 42378
D6.0003.fcs live cells 56525
D9.0003.fcs live cells 34080
D10.0003.fcs live cells 40709
E1.0003.fcs live cells 43677
E2.0003.fcs live cells 51988
E5.0003.fcs live cells 44367
E6.0003.fcs live cells 53509
E9.0003.fcs live cells 32033
E10.0003.fcs live cells 39023
F1.0003.fcs live cells 57727
F2.0003.fcs live cells 55284
F5.0003.fcs live cells 61692
F6.0003.fcs live cells 56793
F9.0003.fcs live cells 64139
F10.0003.fcs live cells 66341
G1.0003.fcs live cells 73114
G2.0003.fcs live cells 63744
G5.0003.fcs live cells 62139
G6.0003.fcs live cells 66503
G9.0003.fcs live cells 72568
G10.0003.fcs live cells 72188

[redacted]

Plate4_KFN_vs_HPB_TRBC1

Well	Target	E:T ratio	Donor	CAR construct
**1B**	HPB TRBC1+ve	1:4	Donor 4	KFN_Hinge_28z
**1D**	HPB TRBC1+ve	1:4	Donor 4	KFN_CD8STK_28z
**1E**	HPB TRBC1+ve	1:4	Donor 4	KFN_CD28STK_CD28TM_28z
**1F**	HPB TRBC1+ve	1:4	Donor 4	aCD19-CAR
**1G**	HPB TRBC1+ve	1:4	Donor 4	Non-transduced
**1H**	HPB TRBC1+ve	N/A	N/A	N/A
**2B**	HPB TRBC1+ve	1:8	Donor 4	KFN_Hinge_28z
**2D**	HPB TRBC1+ve	1:8	Donor 4	KFN_CD8STK_28z
**2E**	HPB TRBC1+ve	1:8	Donor 4	KFN_CD28STK_CD28TM_28z
**2F**	HPB TRBC1+ve	1:8	Donor 4	aCD19-CAR
**2G**	HPB TRBC1+ve	1:8	Donor 4	Non-transduced
**2H**	HPB TRBC1+ve	N/A	N/A	N/A
**5B**	HPB TRBC1+ve	1:4	Donor 5	KFN_Hinge_28z
**5D**	HPB TRBC1+ve	1:4	Donor 5	KFN_CD8STK_28z
**5E**	HPB TRBC1+ve	1:4	Donor 5	KFN_CD28STK_CD28TM_28z
**5F**	HPB TRBC1+ve	1:4	Donor 5	aCD19-CAR
**5G**	HPB TRBC1+ve	1:4	Donor 5	Non-transduced
**5H**	HPB TRBC1+ve	N/A	N/A	N/A
**6B**	HPB TRBC1+ve	1:8	Donor 5	KFN_Hinge_28z
**6D**	HPB TRBC1+ve	1:8	Donor 5	KFN_CD8STK_28z
**6E**	HPB TRBC1+ve	1:8	Donor 5	KFN_CD28STK_CD28TM_28z
**6F**	HPB TRBC1+ve	1:8	Donor 5	aCD19-CAR
**6G**	HPB TRBC1+ve	1:8	Donor 5	Non-transduced
**6H**	HPB TRBC1+ve	N/A	N/A	N/A
**9B**	HPB TRBC1+ve	1:4	Donor 6	KFN_Hinge_28z
**9D**	HPB TRBC1+ve	1:4	Donor 6	KFN_CD8STK_28z
**9E**	HPB TRBC1+ve	1:4	Donor 6	KFN_CD28STK_CD28TM_28z
**9F**	HPB TRBC1+ve	1:4	Donor 6	aCD19-CAR
**9G**	HPB TRBC1+ve	1:4	Donor 6	Non-transduced
**9H**	HPB TRBC1+ve	N/A	N/A	N/A
**10B**	HPB TRBC1+ve	1:8	Donor 6	KFN_Hinge_28z
**10D**	HPB TRBC1+ve	1:8	Donor 6	KFN_CD8STK_28z
**10E**	HPB TRBC1+ve	1:8	Donor 6	KFN_CD28STK_CD28TM_28z
**10F**	HPB TRBC1+ve	1:8	Donor 6	aCD19-CAR
**10G**	HPB TRBC1+ve	1:8	Donor 6	Non-transduced
**10H**	HPB TRBC1+ve	N/A	N/A	N/A

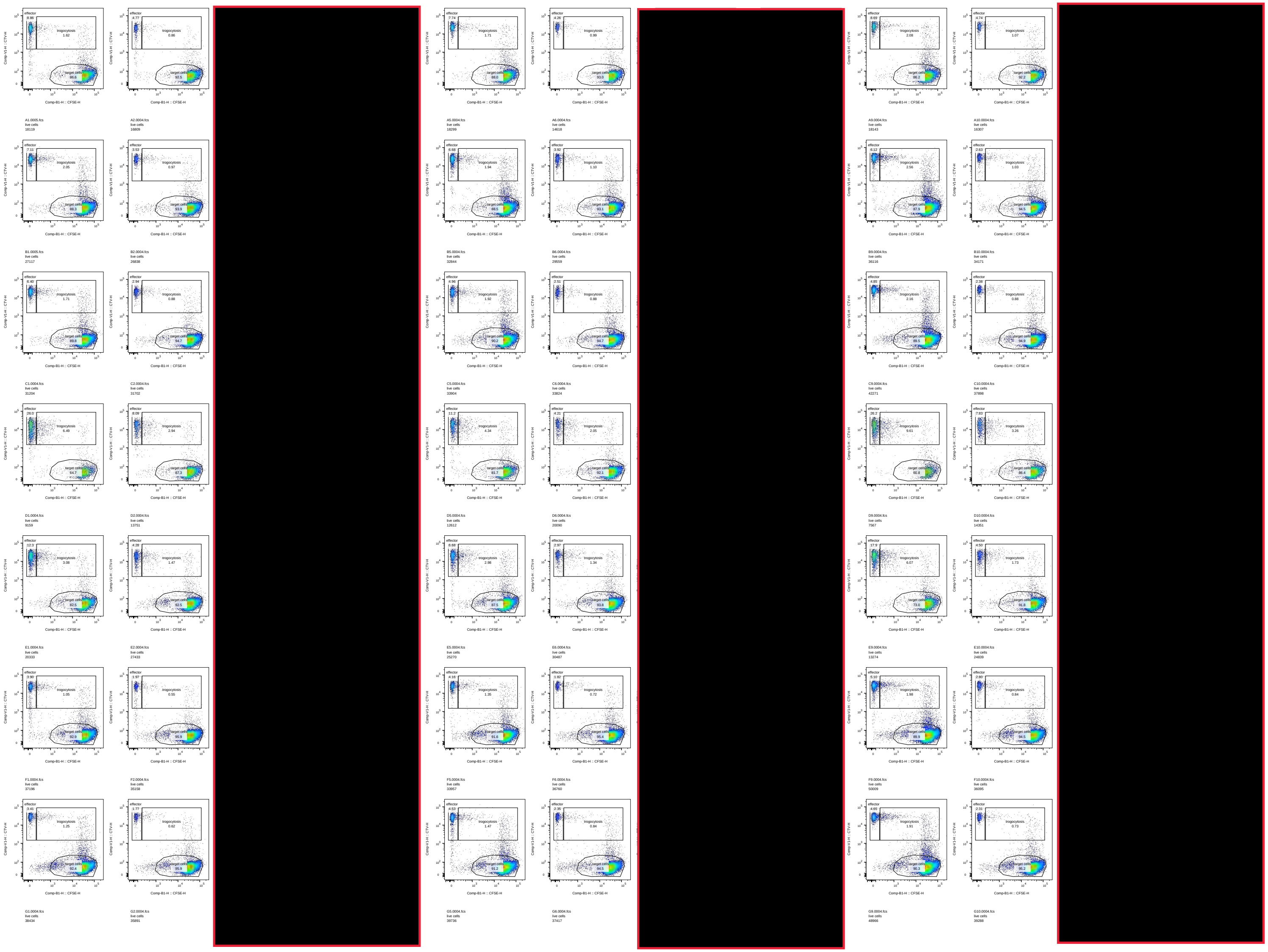

Comp-V1-H :: CTV-H
Comp-B1-H :: CFSE-H
effector 8.86
trogocytosis 1.62
target cells 86.6
A1.0005.fcs
live cells
18119
effector 4.77
trogocytosis 0.86
target cells 92.5
A2.0004.fcs
live cells
16809
[redacted]
effector 7.74
trogocytosis 1.71
target cells 88.0
A5.0004.fcs
live cells
18299
effector 4.26
trogocytosis 0.99
target cells 93.0
A6.0004.fcs
live cells
14618
[redacted]
effector 8.69
trogocytosis 2.08
target cells 86.2
A9.0004.fcs
live cells
18143
effector 4.74
trogocytosis 1.07
target cells 92.2
A10.0004.fcs
live cells
16307
[redacted]
effector 7.11
trogocytosis 2.05
target cells 88.3
B1.0005.fcs
live cells
27117
effector 3.53
trogocytosis 0.97
target cells 93.9
B2.0004.fcs
live cells
26838
effector 6.68
trogocytosis 1.94
target cells 88.5
B5.0004.fcs
live cells
32844
effector 3.92
trogocytosis 1.10
target cells 93.1
B6.0004.fcs
live cells
29559
effector 6.12
trogocytosis 2.56
target cells 87.9
B9.0004.fcs
live cells
36116
effector 2.63
trogocytosis 1.03
target cells 94.5
B10.0004.fcs
live cells
34171
effector 6.40
trogocytosis 1.71
target cells 89.8
C1.0004.fcs
live cells
31204
effector 2.94
trogocytosis 0.88
target cells 94.7
C2.0004.fcs
live cells
31702
effector 4.96
trogocytosis 1.92
target cells 90.2
C5.0004.fcs
live cells
33904
effector 2.51
trogocytosis 0.88
target cells 94.7
C6.0004.fcs
live cells
33824
effector 4.85
trogocytosis 2.16
target cells 89.5
C9.0004.fcs
live cells
42271
effector 2.38
trogocytosis 0.88
target cells 94.9
C10.0004.fcs
live cells
37898
effector 26.0
trogocytosis 6.49
target cells 64.7
D1.0004.fcs
live cells
9159
effector 8.09
trogocytosis 2.94
target cells 87.3
D2.0004.fcs
live cells
13751
effector 11.2
trogocytosis 4.34
target cells 81.7
D5.0004.fcs
live cells
12612
effector 4.21
trogocytosis 2.05
target cells 92.1
D6.0004.fcs
live cells
20090
effector 26.2
trogocytosis 9.61
target cells 60.8
D9.0004.fcs
live cells
7567
effector 7.83
trogocytosis 3.26
target cells 86.4
D10.0004.fcs
live cells
14351
effector 12.3
trogocytosis 3.08
target cells 82.5
E1.0004.fcs
live cells
20333
effector 4.28
trogocytosis 1.47
target cells 92.5
E2.0004.fcs
live cells
27433
effector 6.68
trogocytosis 2.98
target cells 87.5
E5.0004.fcs
live cells
25270
effector 2.97
trogocytosis 1.34
target cells 93.8
E6.0004.fcs
live cells
30487
effector 17.9
trogocytosis 6.07
target cells 73.0
E9.0004.fcs
live cells
13274
effector 4.50
trogocytosis 1.73
target cells 91.8
E10.0004.fcs
live cells
24839
effector 3.90
trogocytosis 1.05
target cells 92.9
F1.0004.fcs
live cells
37196
effector 1.97
trogocytosis 0.55
target cells 95.9
F2.0004.fcs
live cells
35158
effector 4.16
trogocytosis 1.35
target cells 91.6
F5.0004.fcs
live cells
33957
effector 1.82
trogocytosis 0.72
target cells 95.4
F6.0004.fcs
live cells
36760
effector 5.10
trogocytosis 1.98
target cells 89.9
F9.0004.fcs
live cells
50009
effector 2.80
trogocytosis 0.84
target cells 94.5
F10.0004.fcs
live cells
36095
effector 3.41
trogocytosis 1.25
target cells 92.4
G1.0004.fcs
live cells
38434
effector 1.77
trogocytosis 0.62
target cells 95.9
G2.0004.fcs
live cells
35891
effector 4.53
trogocytosis 1.47
target cells 91.2
G5.0004.fcs
live cells
39736
effector 2.35
trogocytosis 0.84
target cells 94.5
G6.0004.fcs
live cells
37417
effector 4.65
trogocytosis 1.91
target cells 90.3
G9.0004.fcs
live cells
48966
effector 2.31
trogocytosis 0.73
target cells 95.2
G10.0004.fcs
live cells
39288

Plate5_KFN_vs_HPB_TRBC2

**Well**	**Target**	**E:T ratio**	**Donor**	**CAR construct**
**1B**	HPB TRBC2+ve	1:4	Donor 4	KFN_Hinge_28z
**1D**	HPB TRBC2+ve	1:4	Donor 4	KFN_CD8STK_28z
**1E**	HPB TRBC2+ve	1:4	Donor 4	KFN_CD28STK_CD28TM_28z
**1F**	HPB TRBC2+ve	1:4	Donor 4	aCD19-CAR
**1G**	HPB TRBC2+ve	1:4	Donor 4	Non-transduced
**1H**	HPB TRBC2+ve	N/A	N/A	N/A
**2B**	HPB TRBC2+ve	1:8	Donor 4	KFN_Hinge_28z
**2D**	HPB TRBC2+ve	1:8	Donor 4	KFN_CD8STK_28z
**2E**	HPB TRBC2+ve	1:8	Donor 4	KFN_CD28STK_CD28TM_28z
**2F**	HPB TRBC2+ve	1:8	Donor 4	aCD19-CAR
**2G**	HPB TRBC2+ve	1:8	Donor 4	Non-transduced
**2H**	HPB TRBC2+ve	N/A	N/A	N/A
**5B**	HPB TRBC2+ve	1:4	Donor 5	KFN_Hinge_28z
**5D**	HPB TRBC2+ve	1:4	Donor 5	KFN_CD8STK_28z
**5E**	HPB TRBC2+ve	1:4	Donor 5	KFN_CD28STK_CD28TM_28z
**5F**	HPB TRBC2+ve	1:4	Donor 5	aCD19-CAR
**5G**	HPB TRBC2+ve	1:4	Donor 5	Non-transduced
**5H**	HPB TRBC2+ve	N/A	N/A	N/A
**6B**	HPB TRBC2+ve	1:8	Donor 5	KFN_Hinge_28z
**6D**	HPB TRBC2+ve	1:8	Donor 5	KFN_CD8STK_28z
**6E**	HPB TRBC2+ve	1:8	Donor 5	KFN_CD28STK_CD28TM_28z
**6F**	HPB TRBC2+ve	1:8	Donor 5	aCD19-CAR
**6G**	HPB TRBC2+ve	1:8	Donor 5	Non-transduced
**6H**	HPB TRBC2+ve	N/A	N/A	N/A
**9B**	HPB TRBC2+ve	1:4	Donor 6	KFN_Hinge_28z
**9D**	HPB TRBC2+ve	1:4	Donor 6	KFN_CD8STK_28z
**9E**	HPB TRBC2+ve	1:4	Donor 6	KFN_CD28STK_CD28TM_28z
**9F**	HPB TRBC2+ve	1:4	Donor 6	aCD19-CAR
**9G**	HPB TRBC2+ve	1:4	Donor 6	Non-transduced
**9H**	HPB TRBC2+ve	N/A	N/A	N/A
**10B**	HPB TRBC2+ve	1:8	Donor 6	KFN_Hinge_28z
**10D**	HPB TRBC2+ve	1:8	Donor 6	KFN_CD8STK_28z
**10E**	HPB TRBC2+ve	1:8	Donor 6	KFN_CD28STK_CD28TM_28z
**10F**	HPB TRBC2+ve	1:8	Donor 6	aCD19-CAR
**10G**	HPB TRBC2+ve	1:8	Donor 6	Non-transduced
**10H**	HPB TRBC2+ve	N/A	N/A	N/A

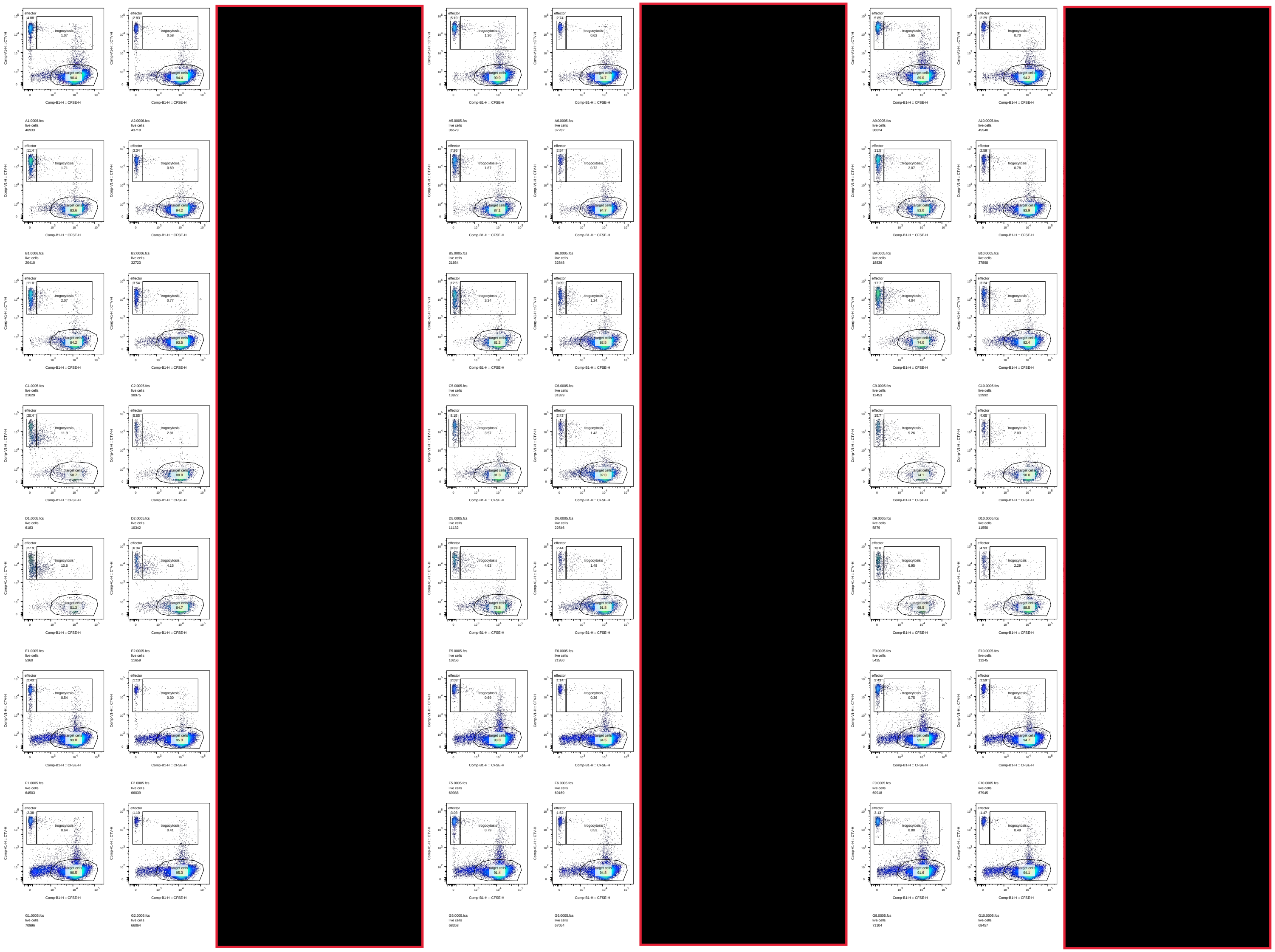

[redacted]

[redacted]

[redacted]

Plate6_KFN_vs_HPB_TRBC-KO

Well	Target	E:T ratio	Donor	CAR construct
**1B**	HPB TRBC KO	1:4	Donor 4	KFN_Hinge_28z
**1D**	HPB TRBC KO	1:4	Donor 4	KFN_CD8STK_28z
**1E**	HPB TRBC KO	1:4	Donor 4	KFN_CD28STK_CD28TM_28z
**1F**	HPB TRBC KO	1:4	Donor 4	aCD19-CAR
**1G**	HPB TRBC KO	1:4	Donor 4	Non-transduced
**1H**	HPB TRBC KO	N/A	N/A	N/A
**2B**	HPB TRBC KO	1:8	Donor 4	KFN_Hinge_28z
**2D**	HPB TRBC KO	1:8	Donor 4	KFN_CD8STK_28z
**2E**	HPB TRBC KO	1:8	Donor 4	KFN_CD28STK_CD28TM_28z
**2F**	HPB TRBC KO	1:8	Donor 4	aCD19-CAR
**2G**	HPB TRBC KO	1:8	Donor 4	Non-transduced
**2H**	HPB TRBC KO	N/A	N/A	N/A
**5B**	HPB TRBC KO	1:4	Donor 5	KFN_Hinge_28z
**5D**	HPB TRBC KO	1:4	Donor 5	KFN_CD8STK_28z
**5E**	HPB TRBC KO	1:4	Donor 5	KFN_CD28STK_CD28TM_28z
**5F**	HPB TRBC KO	1:4	Donor 5	aCD19-CAR
**5G**	HPB TRBC KO	1:4	Donor 5	Non-transduced
**5H**	HPB TRBC KO	N/A	N/A	N/A
**6B**	HPB TRBC KO	1:8	Donor 5	KFN_Hinge_28z
**6D**	HPB TRBC KO	1:8	Donor 5	KFN_CD8STK_28z
**6E**	HPB TRBC KO	1:8	Donor 5	KFN_CD28STK_CD28TM_28z
**6F**	HPB TRBC KO	1:8	Donor 5	aCD19-CAR
**6G**	HPB TRBC KO	1:8	Donor 5	Non-transduced
**6H**	HPB TRBC KO	N/A	N/A	N/A
**9B**	HPB TRBC KO	1:4	Donor 6	KFN_Hinge_28z
**9D**	HPB TRBC KO	1:4	Donor 6	KFN_CD8STK_28z
**9E**	HPB TRBC KO	1:4	Donor 6	KFN_CD28STK_CD28TM_28z
**9F**	HPB TRBC KO	1:4	Donor 6	aCD19-CAR
**9G**	HPB TRBC KO	1:4	Donor 6	Non-transduced
**9H**	HPB TRBC KO	N/A	N/A	N/A
**10B**	HPB TRBC KO	1:8	Donor 6	KFN_Hinge_28z
**10D**	HPB TRBC KO	1:8	Donor 6	KFN_CD8STK_28z
**10E**	HPB TRBC KO	1:8	Donor 6	KFN_CD28STK_CD28TM_28z
**10F**	HPB TRBC KO	1:8	Donor 6	aCD19-CAR
**10G**	HPB TRBC KO	1:8	Donor 6	Non-transduced
**10H**	HPB TRBC KO	N/A	N/A	N/A

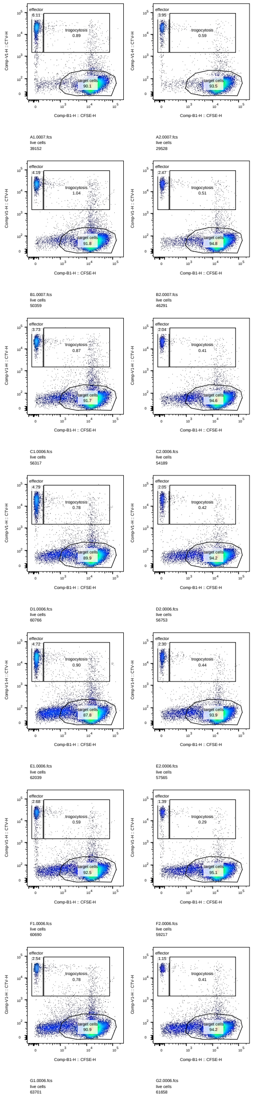

A1.0007.fcs
live cells
39152

A2.0007.fcs
live cells
29528

B1.0007.fcs
live cells
50359

B2.0007.fcs
live cells
46291

C1.0006.fcs
live cells
56317

C2.0006.fcs
live cells
54189

D1.0006.fcs
live cells
60766

D2.0006.fcs
live cells
56753

E1.0006.fcs
live cells
62039

E2.0006.fcs
live cells
57565

F1.0006.fcs
live cells
60690

F2.0006.fcs
live cells
59217

G1.0006.fcs
live cells
63701

G2.0006.fcs
live cells
61658

[redacted]

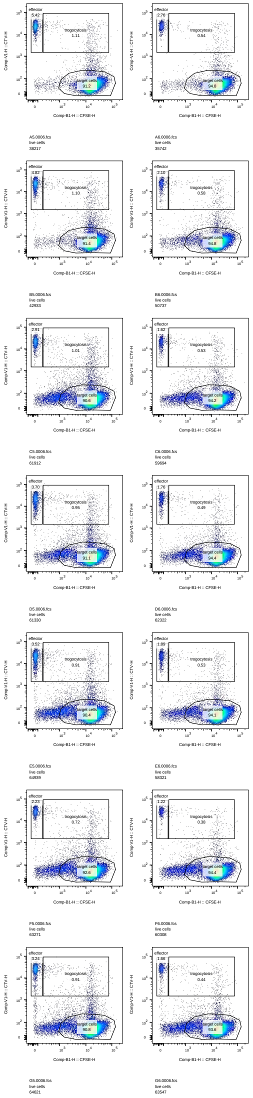

A5.0006.fcs
live cells
38217

A6.0006.fcs
live cells
35742

B5.0006.fcs
live cells
42933

B6.0006.fcs
live cells
50737

C5.0006.fcs
live cells
61912

C6.0006.fcs
live cells
59694

D5.0006.fcs
live cells
61330

D6.0006.fcs
live cells
62322

E5.0006.fcs
live cells
64939

E6.0006.fcs
live cells
58321

F5.0006.fcs
live cells
63271

F6.0006.fcs
live cells
60308

G5.0006.fcs
live cells
64621

G6.0006.fcs
live cells
63547

[redacted]

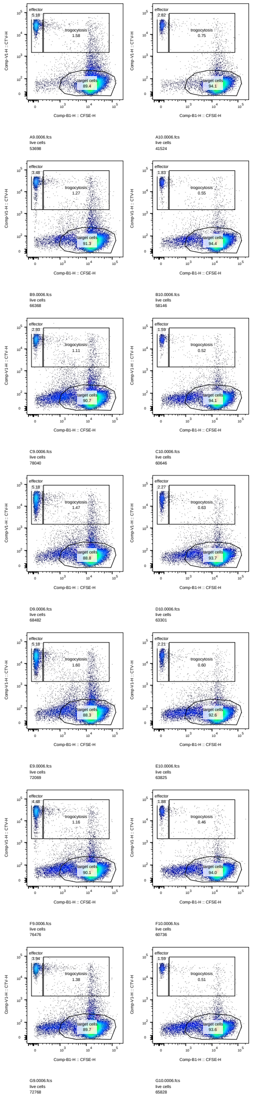

A9.0006.fcs
live cells
53698

A10.0006.fcs
live cells
41524

B9.0006.fcs
live cells
66368

B10.0006.fcs
live cells
58146

C9.0006.fcs
live cells
78040

C10.0006.fcs
live cells
60646

D9.0006.fcs
live cells
68482

D10.0006.fcs
live cells
63301

E9.0006.fcs
live cells
72069

E10.0006.fcs
live cells
63825

F9.0006.fcs
live cells
76476

F10.0006.fcs
live cells
60736

G9.0006.fcs
live cells
72768

G10.0006.fcs
live cells
65828

[redacted]

Plate7_KFN_vs_H9

Well	Target	E:T ratio	Donor	CAR construct
**1B**	H9	1:4	Donor 4	KFN_Hinge_28z
**1D**	H9	1:4	Donor 4	KFN_CD8STK_28z
**1E**	H9	1:4	Donor 4	KFN_CD28STK_CD28TM_28z
**1F**	H9	1:4	Donor 4	aCD19-CAR
**1G**	H9	1:4	Donor 4	Non-transduced
**1H**	H9	N/A	N/A	N/A
**2B**	H9	1:8	Donor 4	KFN_Hinge_28z
**2D**	H9	1:8	Donor 4	KFN_CD8STK_28z
**2E**	H9	1:8	Donor 4	KFN_CD28STK_CD28TM_28z
**2F**	H9	1:8	Donor 4	aCD19-CAR
**2G**	H9	1:8	Donor 4	Non-transduced
**2H**	H9	N/A	N/A	N/A
**5B**	H9	1:4	Donor 5	KFN_Hinge_28z
**5D**	H9	1:4	Donor 5	KFN_CD8STK_28z
**5E**	H9	1:4	Donor 5	KFN_CD28STK_CD28TM_28z
**5F**	H9	1:4	Donor 5	aCD19-CAR
**5G**	H9	1:4	Donor 5	Non-transduced
**5H**	H9	N/A	N/A	N/A
**6B**	H9	1:8	Donor 5	KFN_Hinge_28z
**6D**	H9	1:8	Donor 5	KFN_CD8STK_28z
**6E**	H9	1:8	Donor 5	KFN_CD28STK_CD28TM_28z
**6F**	H9	1:8	Donor 5	aCD19-CAR
**6G**	H9	1:8	Donor 5	Non-transduced
**6H**	H9	N/A	N/A	N/A
**9B**	H9	1:4	Donor 6	KFN_Hinge_28z
**9D**	H9	1:4	Donor 6	KFN_CD8STK_28z
**9E**	H9	1:4	Donor 6	KFN_CD28STK_CD28TM_28z
**9F**	H9	1:4	Donor 6	aCD19-CAR
**9G**	H9	1:4	Donor 6	Non-transduced
**9H**	H9	N/A	N/A	N/A
**10B**	H9	1:8	Donor 6	KFN_Hinge_28z
**10D**	H9	1:8	Donor 6	KFN_CD8STK_28z
**10E**	H9	1:8	Donor 6	KFN_CD28STK_CD28TM_28z
**10F**	H9	1:8	Donor 6	aCD19-CAR
**10G**	H9	1:8	Donor 6	Non-transduced
**10H**	H9	N/A	N/A	N/A

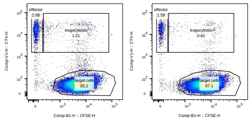

A1.0008.fcs
live cells
83879

A2.0008.fcs
live cells
82602

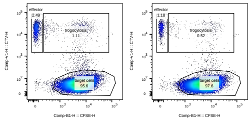

B1.0008.fcs
live cells
85354

B2.0008.fcs
live cells
86890

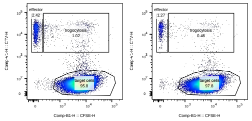

C1.0007.fcs
live cells
85397

C2.0007.fcs
live cells
81758

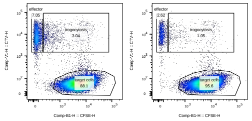

D1.0007.fcs
live cells
50792

D2.0007.fcs
live cells
62309

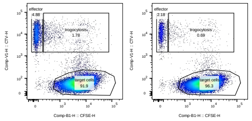

E1.0007.fcs
live cells
63445

E2.0007.fcs
live cells
72053

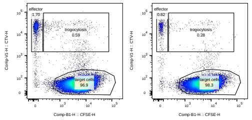

F1.0007.fcs
live cells
93205

F2.0007.fcs
live cells
90573

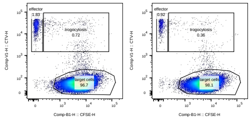

G1.0007.fcs
live cells
94728

G2.0007.fcs
live cells
90702

[redacted]

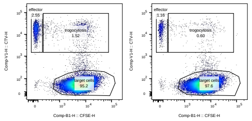

A5.0007.fcs
live cells
82565

A6.0007.fcs
live cells
81822

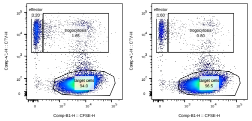

B5.0007.fcs
live cells
82028

B6.0007.fcs
live cells
86308

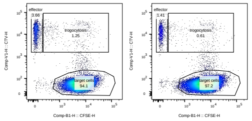

C5.0007.fcs
live cells
47472

C6.0007.fcs
live cells
81917

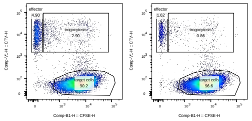

D5.0007.fcs
live cells
41822

D6.0007.fcs
live cells
64067

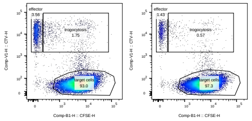

E5.0007.fcs
live cells
56364

E6.0007.fcs
live cells
70990

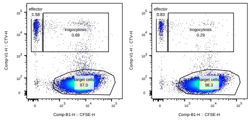

F5.0007.fcs
live cells
89378

F6.0007.fcs
live cells
85257

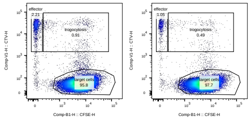

G5.0007.fcs
live cells
90965

G6.0007.fcs
live cells
89131

[redacted]

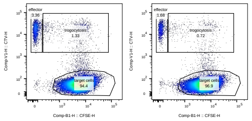

A9.0007.fcs
live cells
82309

A10.0007.fcs
live cells
87711

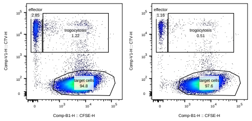

B9.0007.fcs
live cells
88147

B10.0007.fcs
live cells
86947

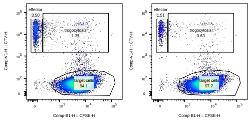

C9.0007.fcs
live cells
76314

C10.0007.fcs
live cells
81030

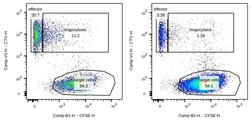

D9.0007.fcs
live cells
12646

D10.0007.fcs
live cells
48075

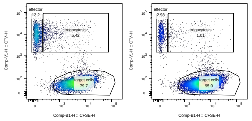

E9.0007.fcs
live cells
23199

E10.0007.fcs
live cells
54183

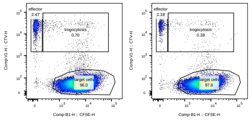

F9.0007.fcs
live cells
86349

F10.0007.fcs
live cells
82961

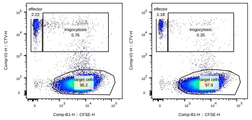

G9.0007.fcs
live cells
89447

G10.0007.fcs
live cells
84930

[redacted]

Plate8_KFN_vs_TALL1

Well	Target	E:T ratio	Donor	CAR construct
**1B**	T-ALL1	1:4	Donor 4	KFN_Hinge_28z
**1D**	T-ALL1	1:4	Donor 4	KFN_CD8STK_28z
**1E**	T-ALL1	1:4	Donor 4	KFN_CD28STK_CD28TM_28z
**1F**	T-ALL1	1:4	Donor 4	aCD19-CAR
**1G**	T-ALL1	1:4	Donor 4	Non-transduced
**1H**	T-ALL1	N/A	N/A	N/A
**2B**	T-ALL1	1:8	Donor 4	KFN_Hinge_28z
**2D**	T-ALL1	1:8	Donor 4	KFN_CD8STK_28z
**2E**	T-ALL1	1:8	Donor 4	KFN_CD28STK_CD28TM_28z
**2F**	T-ALL1	1:8	Donor 4	aCD19-CAR
**2G**	T-ALL1	1:8	Donor 4	Non-transduced
**2H**	T-ALL1	N/A	N/A	N/A
**5B**	T-ALL1	1:4	Donor 5	KFN_Hinge_28z
**5D**	T-ALL1	1:4	Donor 5	KFN_CD8STK_28z
**5E**	T-ALL1	1:4	Donor 5	KFN_CD28STK_CD28TM_28z
**5F**	T-ALL1	1:4	Donor 5	aCD19-CAR
**5G**	T-ALL1	1:4	Donor 5	Non-transduced
**5H**	T-ALL1	N/A	N/A	N/A
**6B**	T-ALL1	1:8	Donor 5	KFN_Hinge_28z
**6D**	T-ALL1	1:8	Donor 5	KFN_CD8STK_28z
**6E**	T-ALL1	1:8	Donor 5	KFN_CD28STK_CD28TM_28z
**6F**	T-ALL1	1:8	Donor 5	aCD19-CAR
**6G**	T-ALL1	1:8	Donor 5	Non-transduced
**6H**	T-ALL1	N/A	N/A	N/A
**9B**	T-ALL1	1:4	Donor 6	KFN_Hinge_28z
**9D**	T-ALL1	1:4	Donor 6	KFN_CD8STK_28z
**9E**	T-ALL1	1:4	Donor 6	KFN_CD28STK_CD28TM_28z
**9F**	T-ALL1	1:4	Donor 6	aCD19-CAR
**9G**	T-ALL1	1:4	Donor 6	Non-transduced
**9H**	T-ALL1	N/A	N/A	N/A
**10B**	T-ALL1	1:8	Donor 6	KFN_Hinge_28z
**10D**	T-ALL1	1:8	Donor 6	KFN_CD8STK_28z
**10E**	T-ALL1	1:8	Donor 6	KFN_CD28STK_CD28TM_28z
**10F**	T-ALL1	1:8	Donor 6	aCD19-CAR
**10G**	T-ALL1	1:8	Donor 6	Non-transduced
**10H**	T-ALL1	N/A	N/A	N/A

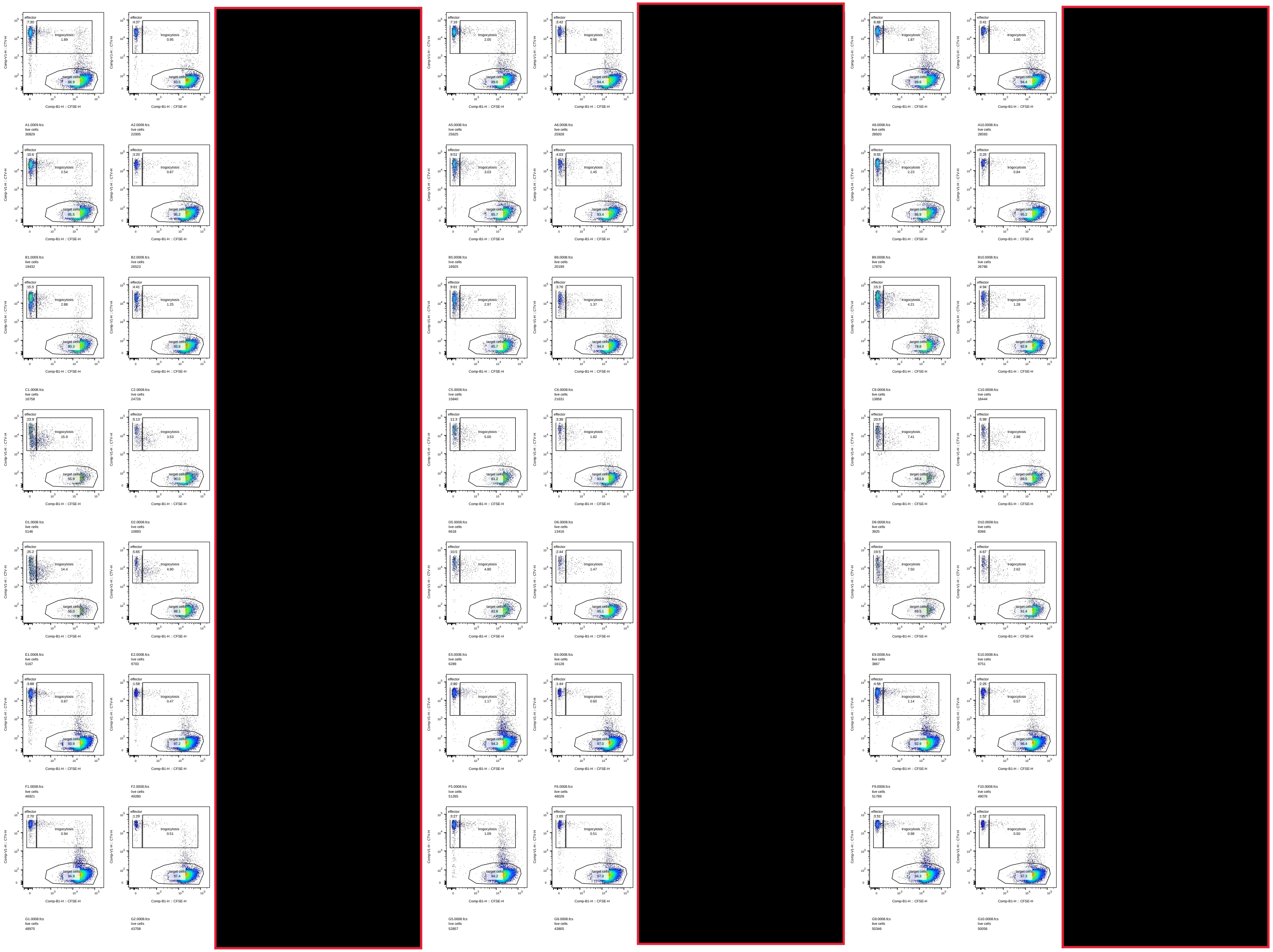

[redacted]

[redacted]

[redacted]

Plate24_JOVI-Prol_vs_H9_TALL_PB

**Well**	**Target**	**E:T ratio**	**Donor**	**CAR construct**
**1A**	H9	1:4	Donor 7	JOVI_Hinge_41bbz
**1D**	H9	1:4	Donor 7	JOVI_CD8STK_28z
**1E**	H9	1:4	Donor 7	JOVI_CD28STK_CD28TM_28z
**1F**	H9	1:4	Donor 7	mJOVI_Hinge_41bbz
**1G**	H9	1:4	Donor 7	aCD19-CAR
**1H**	H9	1:4	Donor 7	Non-transduced
**2A**	H9	1:4	Donor 8	JOVI_Hinge_41bbz
**2D**	H9	1:4	Donor 8	JOVI_CD8STK_28z
**2E**	H9	1:4	Donor 8	JOVI_CD28STK_CD28TM_28z
**2F**	H9	1:4	Donor 8	mJOVI_Hinge_41bbz
**2G**	H9	1:4	Donor 8	aCD19-CAR
**2H**	H9	1:4	Donor 8	Non-transduced
**3A**	H9	1:4	Donor 9	JOVI_Hinge_41bbz
**3D**	H9	1:4	Donor 9	JOVI_CD8STK_28z
**3E**	H9	1:4	Donor 9	JOVI_CD28STK_CD28TM_28z
**3F**	H9	1:4	Donor 9	mJOVI_Hinge_41bbz
**3G**	H9	1:4	Donor 9	aCD19-CAR
**3H**	H9	1:4	Donor 9	Non-transduced
**4A**	T-ALL1	1:4	Donor 7	JOVI_Hinge_41bbz
**4D**	T-ALL1	1:4	Donor 7	JOVI_CD8STK_28z
**4E**	T-ALL1	1:4	Donor 7	JOVI_CD28STK_CD28TM_28z
**4G**	T-ALL1	1:4	Donor 7	aCD19-CAR
**4H**	T-ALL1	1:4	Donor 7	Non-transduced
**5A**	T-ALL1	1:4	Donor 8	JOVI_Hinge_41bbz
**5D**	T-ALL1	1:4	Donor 8	JOVI_CD8STK_28z
**5E**	T-ALL1	1:4	Donor 8	JOVI_CD28STK_CD28TM_28z
**5G**	T-ALL1	1:4	Donor 8	aCD19-CAR
**5H**	T-ALL1	1:4	Donor 8	Non-transduced
**6A**	T-ALL1	1:4	Donor 9	JOVI_Hinge_41bbz
**6D**	T-ALL1	1:4	Donor 9	JOVI_CD8STK_28z
**6E**	T-ALL1	1:4	Donor 9	JOVI_CD28STK_CD28TM_28z
**6G**	T-ALL1	1:4	Donor 9	aCD19-CAR
**6H**	T-ALL1	1:4	Donor 9	Non-transduced

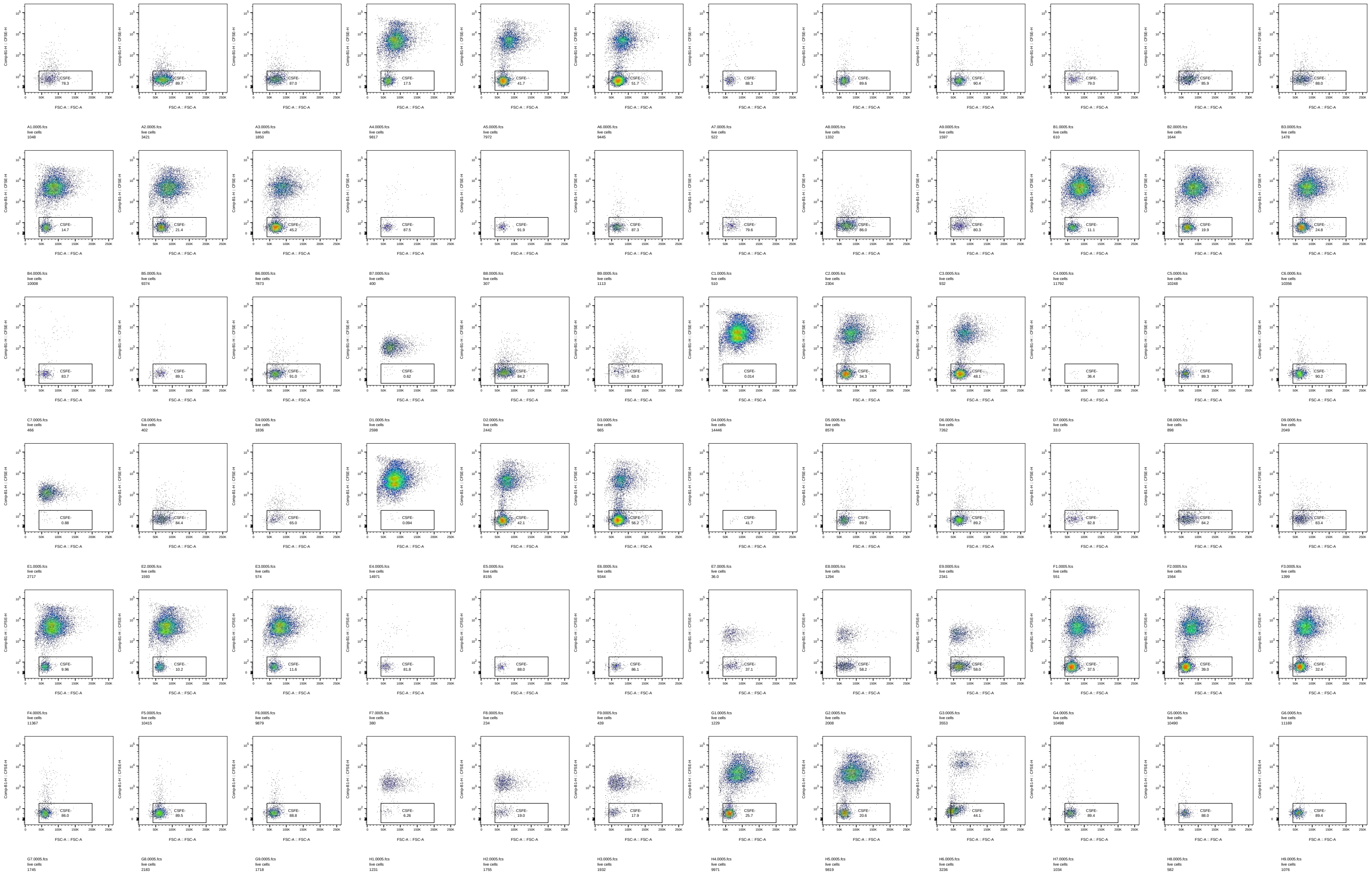
Comp-B1-H :: CFSE-H
FSC-A :: FSC-A
CSFE-
A1.0005.fcs live cells 1048 — 78.3
A2.0005.fcs live cells 3421 — 89.7
A3.0005.fcs live cells 1850 — 87.0
A4.0005.fcs live cells 9817 — 17.5
A5.0005.fcs live cells 7972 — 41.7
A6.0005.fcs live cells 9445 — 51.7
A7.0005.fcs live cells 522 — 88.3
A8.0005.fcs live cells 1332 — 89.6
A9.0005.fcs live cells 1597 — 90.4
B1.0005.fcs live cells 610 — 79.0
B2.0005.fcs live cells 1644 — 85.9
B3.0005.fcs live cells 1478 — 88.0
B4.0005.fcs live cells 10008 — 14.7
B5.0005.fcs live cells 9374 — 21.4
B6.0005.fcs live cells 7873 — 45.2
B7.0005.fcs live cells 400 — 87.5
B8.0005.fcs live cells 307 — 91.9
B9.0005.fcs live cells 1113 — 87.3
C1.0005.fcs live cells 510 — 79.6
C2.0005.fcs live cells 2304 — 86.0
C3.0005.fcs live cells 932 — 80.3
C4.0005.fcs live cells 11792 — 11.1
C5.0005.fcs live cells 10268 — 19.9
C6.0005.fcs live cells 10356 — 24.8
C7.0005.fcs live cells 466 — 83.7
C8.0005.fcs live cells 402 — 89.1
C9.0005.fcs live cells 1836 — 91.0
D1.0005.fcs live cells 2598 — 0.62
D2.0005.fcs live cells 2442 — 84.2
D3.0005.fcs live cells 665 — 63.0
D4.0005.fcs live cells 14446 — 0.014
D5.0005.fcs live cells 8578 — 34.3
D6.0005.fcs live cells 7262 — 48.1
D7.0005.fcs live cells 33.0 — 36.4
D8.0005.fcs live cells 898 — 89.3
D9.0005.fcs live cells 2049 — 90.2
E1.0005.fcs live cells 2717 — 0.88
E2.0005.fcs live cells 1593 — 84.4
E3.0005.fcs live cells 574 — 65.0
E4.0005.fcs live cells 14971 — 0.094
E5.0005.fcs live cells 8155 — 42.1
E6.0005.fcs live cells 9344 — 56.2
E7.0005.fcs live cells 36.0 — 41.7
E8.0005.fcs live cells 1294 — 89.2
E9.0005.fcs live cells 2341 — 89.2
F1.0005.fcs live cells 551 — 82.8
F2.0005.fcs live cells 1564 — 84.2
F3.0005.fcs live cells 1399 — 83.4
F4.0005.fcs live cells 11367 — 9.96
F5.0005.fcs live cells 10415 — 10.2
F6.0005.fcs live cells 9879 — 11.6
F7.0005.fcs live cells 380 — 81.8
F8.0005.fcs live cells 234 — 88.0
F9.0005.fcs live cells 439 — 86.1
G1.0005.fcs live cells 1229 — 37.1
G2.0005.fcs live cells 2008 — 58.2
G3.0005.fcs live cells 3553 — 58.0
G4.0005.fcs live cells 10498 — 37.5
G5.0005.fcs live cells 10490 — 39.0
G6.0005.fcs live cells 11169 — 32.4
G7.0005.fcs live cells 1745 — 86.0
G8.0005.fcs live cells 2183 — 89.5
G9.0005.fcs live cells 1718 — 88.8
H1.0005.fcs live cells 1231 — 6.26
H2.0005.fcs live cells 1755 — 19.0
H3.0005.fcs live cells 1932 — 17.9
H4.0005.fcs live cells 9971 — 25.7
H5.0005.fcs live cells 9819 — 20.6
H6.0005.fcs live cells 3236 — 44.1
H7.0005.fcs live cells 1034 — 89.4
H8.0005.fcs live cells 582 — 88.0
H9.0005.fcs live cells 1076 — 89.4

Plate23_JOVI-Prol_vs_HPB

**Well**	**Target**	**E:T ratio**	**Donor**	**CAR construct**
**1A**	HPB TRBC1+ve	1:4	Donor 7	JOVI_Hinge_41bbz
**1D**	HPB TRBC1+ve	1:4	Donor 7	JOVI_CD8STK_28z
**1E**	HPB TRBC1+ve	1:4	Donor 7	JOVI_CD28STK_CD28TM_28z
**1G**	HPB TRBC1+ve	1:4	Donor 7	aCD19-CAR
**1H**	HPB TRBC1+ve	1:4	Donor 7	Non-transduced
**2A**	HPB TRBC1+ve	1:4	Donor 8	JOVI_Hinge_41bbz
**2D**	HPB TRBC1+ve	1:4	Donor 8	JOVI_CD8STK_28z
**2E**	HPB TRBC1+ve	1:4	Donor 8	JOVI_CD28STK_CD28TM_28z
**2G**	HPB TRBC1+ve	1:4	Donor 8	aCD19-CAR
**2H**	HPB TRBC1+ve	1:4	Donor 8	Non-transduced
**3A**	HPB TRBC1+ve	1:4	Donor 9	JOVI_Hinge_41bbz
**3D**	HPB TRBC1+ve	1:4	Donor 9	JOVI_CD8STK_28z
**3E**	HPB TRBC1+ve	1:4	Donor 9	JOVI_CD28STK_CD28TM_28z
**3G**	HPB TRBC1+ve	1:4	Donor 9	aCD19-CAR
**3H**	HPB TRBC1+ve	1:4	Donor 9	Non-transduced
**4A**	HPB TRBC2+ve	1:4	Donor 7	JOVI_Hinge_41bbz
**4D**	HPB TRBC2+ve	1:4	Donor 7	JOVI_CD8STK_28z
**4E**	HPB TRBC2+ve	1:4	Donor 7	JOVI_CD28STK_CD28TM_28z
**4G**	HPB TRBC2+ve	1:4	Donor 7	aCD19-CAR
**4H**	HPB TRBC2+ve	1:4	Donor 7	Non-transduced
**5A**	HPB TRBC2+ve	1:4	Donor 8	JOVI_Hinge_41bbz
**5D**	HPB TRBC2+ve	1:4	Donor 8	JOVI_CD8STK_28z
**5E**	HPB TRBC2+ve	1:4	Donor 8	JOVI_CD28STK_CD28TM_28z
**5G**	HPB TRBC2+ve	1:4	Donor 8	aCD19-CAR
**5H**	HPB TRBC2+ve	1:4	Donor 8	Non-transduced
**6A**	HPB TRBC2+ve	1:4	Donor 9	JOVI_Hinge_41bbz
**6D**	HPB TRBC2+ve	1:4	Donor 9	JOVI_CD8STK_28z
**6E**	HPB TRBC2+ve	1:4	Donor 9	JOVI_CD28STK_CD28TM_28z
**6G**	HPB TRBC2+ve	1:4	Donor 9	aCD19-CAR
**6H**	HPB TRBC2+ve	1:4	Donor 9	Non-transduced
**7A**	HPB TRBC KO	1:4	Donor 7	JOVI_Hinge_41bbz
**7D**	HPB TRBC KO	1:4	Donor 7	JOVI_CD8STK_28z
**7E**	HPB TRBC KO	1:4	Donor 7	JOVI_CD28STK_CD28TM_28z
**7G**	HPB TRBC KO	1:4	Donor 7	aCD19-CAR
**7H**	HPB TRBC KO	1:4	Donor 7	Non-transduced
**8A**	HPB TRBC KO	1:4	Donor 8	JOVI_Hinge_41bbz
**8D**	HPB TRBC KO	1:4	Donor 8	JOVI_CD8STK_28z
**8E**	HPB TRBC KO	1:4	Donor 8	JOVI_CD28STK_CD28TM_28z
**8G**	HPB TRBC KO	1:4	Donor 8	aCD19-CAR
**8H**	HPB TRBC KO	1:4	Donor 8	Non-transduced
**9A**	HPB TRBC KO	1:4	Donor 9	JOVI_Hinge_41bbz
**9D**	HPB TRBC KO	1:4	Donor 9	JOVI_CD8STK_28z
**9E**	HPB TRBC KO	1:4	Donor 9	JOVI_CD28STK_CD28TM_28z
**9G**	HPB TRBC KO	1:4	Donor 9	aCD19-CAR
**9H**	HPB TRBC KO	1:4	Donor 9	Non-transduced
**10A**	N/A	N/A	N/A	N/A
**10B**	N/A	N/A	N/A	N/A
**10C**	N/A	N/A	N/A	N/A

**10D**	N/A	N/A	N/A	N/A
**10E**	N/A	N/A	N/A	N/A
**10F**	N/A	N/A	N/A	N/A
**10G**	N/A	N/A	N/A	N/A
**10H**	N/A	N/A	N/A	N/A
**11A**	N/A	N/A	N/A	N/A
**11B**	N/A	N/A	N/A	N/A
**11C**	N/A	N/A	N/A	N/A
**11D**	N/A	N/A	N/A	N/A
**11E**	N/A	N/A	N/A	N/A
**11F**	N/A	N/A	N/A	N/A
**11G**	N/A	N/A	N/A	N/A
**11H**	N/A	N/A	N/A	N/A
**12A**	N/A	N/A	N/A	N/A
**12B**	N/A	N/A	N/A	N/A
**12C**	N/A	N/A	N/A	N/A
**12D**	N/A	N/A	N/A	N/A
**12E**	N/A	N/A	N/A	N/A
**12F**	N/A	N/A	N/A	N/A
**12G**	N/A	N/A	N/A	N/A
**12H**	N/A	N/A	N/A	N/A

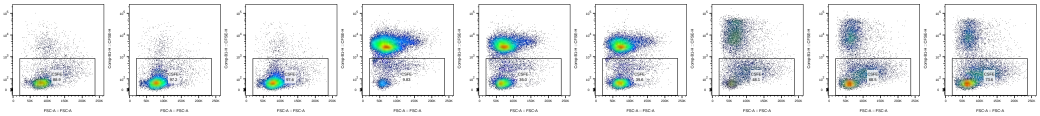

A1.0004.fcs<br>live cells<br>7828	A2.0004.fcs<br>live cells<br>18246	A3.0004.fcs<br>live cells<br>21243	A4.0004.fcs<br>live cells<br>41391	A5.0004.fcs<br>live cells<br>32734	A6.0004.fcs<br>live cells<br>30945	A7.0004.fcs<br>live cells<br>12071	A8.0004.fcs<br>live cells<br>14507	A9.0004.fcs<br>live cells<br>15938

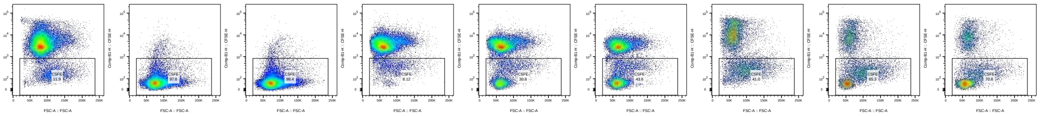

D1.0004.fcs<br>live cells<br>47721	D2.0004.fcs<br>live cells<br>43938	D3.0004.fcs<br>live cells<br>62573	D4.0004.fcs<br>live cells<br>46154	D5.0004.fcs<br>live cells<br>36070	D6.0004.fcs<br>live cells<br>34491	D7.0004.fcs<br>live cells<br>16930	D8.0004.fcs<br>live cells<br>14934	D9.0004.fcs<br>live cells<br>15023

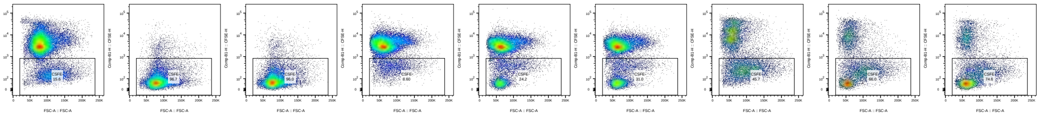

E1.0004.fcs<br>live cells<br>50604	E2.0004.fcs<br>live cells<br>32405	E3.0004.fcs<br>live cells<br>24540	E4.0004.fcs<br>live cells<br>49484	E5.0004.fcs<br>live cells<br>38287	E6.0004.fcs<br>live cells<br>36339	E7.0004.fcs<br>live cells<br>19536	E8.0004.fcs<br>live cells<br>14721	E9.0004.fcs<br>live cells<br>16336

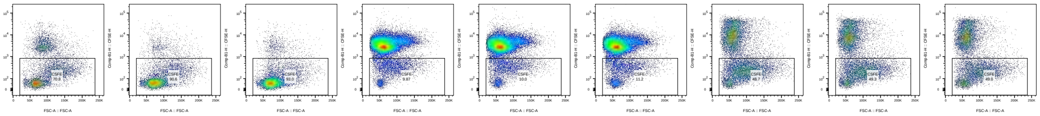

F1.0004.fcs<br>live cells<br>9753	F2.0004.fcs<br>live cells<br>10279	F3.0004.fcs<br>live cells<br>13777	F4.0004.fcs<br>live cells<br>50991	F5.0004.fcs<br>live cells<br>46954	F6.0004.fcs<br>live cells<br>47567	F7.0004.fcs<br>live cells<br>18450	F8.0004.fcs<br>live cells<br>16051	F9.0004.fcs<br>live cells<br>14148

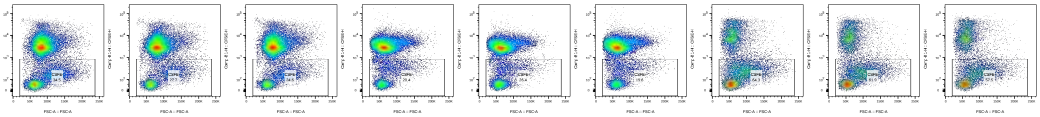

G1.0004.fcs<br>live cells<br>48535	G2.0004.fcs<br>live cells<br>43023	G3.0004.fcs<br>live cells<br>46604	G4.0004.fcs<br>live cells<br>47797	G5.0004.fcs<br>live cells<br>45889	G6.0004.fcs<br>live cells<br>47617	G7.0004.fcs<br>live cells<br>23843	G8.0004.fcs<br>live cells<br>20267	G9.0004.fcs<br>live cells<br>18702

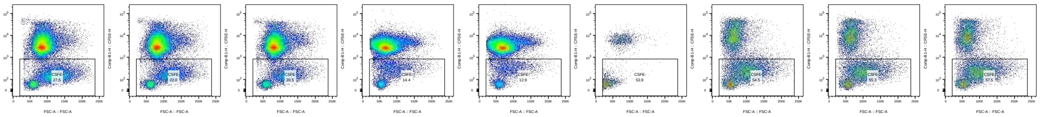

H1.0004.fcs<br>live cells<br>54062	H2.0004.fcs<br>live cells<br>52484	H3.0004.fcs<br>live cells<br>43899	H4.0004.fcs<br>live cells<br>51780	H5.0004.fcs<br>live cells<br>48177	H6.0004.fcs<br>live cells<br>3770	H7.0004.fcs<br>live cells<br>21737	H8.0004.fcs<br>live cells<br>19533	H9.0004.fcs<br>live cells<br>19609

Plate22_JOVI-Prol_vs_Jurkat

Well	Target	E:T ratio	Donor	CAR construct
**1A**	Jurkat TRBC1+ve	1:4	Donor 7	JOVI_Hinge_41bbz
**1D**	Jurkat TRBC1+ve	1:4	Donor 7	JOVI_CD8STK_28z
**1E**	Jurkat TRBC1+ve	1:4	Donor 7	JOVI_CD28STK_CD28TM_28z
**1F**	Jurkat TRBC1+ve	1:4	Donor 7	mJOVI_Hinge_41bbz
**1G**	Jurkat TRBC1+ve	1:4	Donor 7	aCD19-CAR
**1H**	Jurkat TRBC1+ve	1:4	Donor 7	Non-transduced
**2A**	Jurkat TRBC1+ve	1:4	Donor 8	JOVI_Hinge_41bbz
**2D**	Jurkat TRBC1+ve	1:4	Donor 8	JOVI_CD8STK_28z
**2E**	Jurkat TRBC1+ve	1:4	Donor 8	JOVI_CD28STK_CD28TM_28z
**2F**	Jurkat TRBC1+ve	1:4	Donor 8	mJOVI_Hinge_41bbz
**2G**	Jurkat TRBC1+ve	1:4	Donor 8	aCD19-CAR
**2H**	Jurkat TRBC1+ve	1:4	Donor 8	Non-transduced
**3A**	Jurkat TRBC1+ve	1:4	Donor 9	JOVI_Hinge_41bbz
**3D**	Jurkat TRBC1+ve	1:4	Donor 9	JOVI_CD8STK_28z
**3E**	Jurkat TRBC1+ve	1:4	Donor 9	JOVI_CD28STK_CD28TM_28z
**3F**	Jurkat TRBC1+ve	1:4	Donor 9	mJOVI_Hinge_41bbz
**3G**	Jurkat TRBC1+ve	1:4	Donor 9	aCD19-CAR
**3H**	Jurkat TRBC1+ve	1:4	Donor 9	Non-transduced
**4A**	Jurkat TRBC2+ve	1:4	Donor 7	JOVI_Hinge_41bbz
**4D**	Jurkat TRBC2+ve	1:4	Donor 7	JOVI_CD8STK_28z
**4E**	Jurkat TRBC2+ve	1:4	Donor 7	JOVI_CD28STK_CD28TM_28z
**4F**	Jurkat TRBC2+ve	1:4	Donor 7	mJOVI_Hinge_41bbz
**4G**	Jurkat TRBC2+ve	1:4	Donor 7	aCD19-CAR
**4H**	Jurkat TRBC2+ve	1:4	Donor 7	Non-transduced
**5A**	Jurkat TRBC2+ve	1:4	Donor 8	JOVI_Hinge_41bbz
**5D**	Jurkat TRBC2+ve	1:4	Donor 8	JOVI_CD8STK_28z
**5E**	Jurkat TRBC2+ve	1:4	Donor 8	JOVI_CD28STK_CD28TM_28z
**5F**	Jurkat TRBC2+ve	1:4	Donor 8	mJOVI_Hinge_41bbz
**5G**	Jurkat TRBC2+ve	1:4	Donor 8	aCD19-CAR
**5H**	Jurkat TRBC2+ve	1:4	Donor 8	Non-transduced
**6A**	Jurkat TRBC2+ve	1:4	Donor 9	JOVI_Hinge_41bbz
**6D**	Jurkat TRBC2+ve	1:4	Donor 9	JOVI_CD8STK_28z
**6E**	Jurkat TRBC2+ve	1:4	Donor 9	JOVI_CD28STK_CD28TM_28z
**6F**	Jurkat TRBC2+ve	1:4	Donor 9	mJOVI_Hinge_41bbz
**6G**	Jurkat TRBC2+ve	1:4	Donor 9	aCD19-CAR
**6H**	Jurkat TRBC2+ve	1:4	Donor 9	Non-transduced
**7A**	Jurkat TRBC KO	1:4	Donor 7	JOVI_Hinge_41bbz
**7D**	Jurkat TRBC KO	1:4	Donor 7	JOVI_CD8STK_28z
**7E**	Jurkat TRBC KO	1:4	Donor 7	JOVI_CD28STK_CD28TM_28z
**7F**	Jurkat TRBC KO	1:4	Donor 7	mJOVI_Hinge_41bbz
**7G**	Jurkat TRBC KO	1:4	Donor 7	aCD19-CAR
**7H**	Jurkat TRBC KO	1:4	Donor 7	Non-transduced
**8A**	Jurkat TRBC KO	1:4	Donor 8	JOVI_Hinge_41bbz
**8D**	Jurkat TRBC KO	1:4	Donor 8	JOVI_CD8STK_28z
**8E**	Jurkat TRBC KO	1:4	Donor 8	JOVI_CD28STK_CD28TM_28z
**8F**	Jurkat TRBC KO	1:4	Donor 8	mJOVI_Hinge_41bbz
**8G**	Jurkat TRBC KO	1:4	Donor 8	aCD19-CAR
**8H**	Jurkat TRBC KO	1:4	Donor 8	Non-transduced

**9A**	Jurkat TRBC KO	1:4	Donor 9	JOVI_Hinge_41bbz
**9D**	Jurkat TRBC KO	1:4	Donor 9	JOVI_CD8STK_28z
**9E**	Jurkat TRBC KO	1:4	Donor 9	JOVI_CD28STK_CD28TM_28z
**9F**	Jurkat TRBC KO	1:4	Donor 9	mJOVI_Hinge_41bbz
**9G**	Jurkat TRBC KO	1:4	Donor 9	aCD19-CAR
**9H**	Jurkat TRBC KO	1:4	Donor 9	Non-transduced
**10A**	N/A	N/A	N/A	N/A
**10B**	N/A	N/A	N/A	N/A
**10C**	N/A	N/A	N/A	N/A
**10D**	N/A	N/A	N/A	N/A
**10E**	N/A	N/A	N/A	N/A
**10F**	N/A	N/A	N/A	N/A
**10G**	N/A	N/A	N/A	N/A
**10H**	N/A	N/A	N/A	N/A
**11A**	N/A	N/A	N/A	N/A
**11B**	N/A	N/A	N/A	N/A
**11C**	N/A	N/A	N/A	N/A
**11D**	N/A	N/A	N/A	N/A
**11E**	N/A	N/A	N/A	N/A
**11F**	N/A	N/A	N/A	N/A
**11G**	N/A	N/A	N/A	N/A
**11H**	N/A	N/A	N/A	N/A
**12A**	N/A	N/A	N/A	N/A
**12B**	N/A	N/A	N/A	N/A
**12C**	N/A	N/A	N/A	N/A
**12D**	N/A	N/A	N/A	N/A
**12E**	N/A	N/A	N/A	N/A
**12F**	N/A	N/A	N/A	N/A
**12G**	N/A	N/A	N/A	N/A
**12H**	N/A	N/A	N/A	N/A

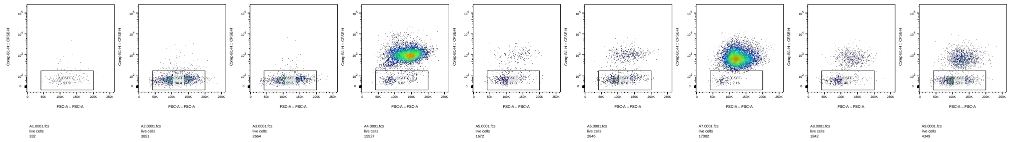
Comp-B1-H :: CFSE-H
FSC-A :: FSC-A
CSFE- 91.9
A1.0001.fcs
live cells
332
CSFE- 94.4
A2.0001.fcs
live cells
3851
CSFE- 95.6
A3.0001.fcs
live cells
2664
CSFE- 5.02
A4.0001.fcs
live cells
15527
CSFE- 77.3
A5.0001.fcs
live cells
1672
CSFE- 67.6
A6.0001.fcs
live cells
2846
CSFE- 2.16
A7.0001.fcs
live cells
17002
CSFE- 46.7
A8.0001.fcs
live cells
1842
CSFE- 53.1
A9.0001.fcs
live cells
4349

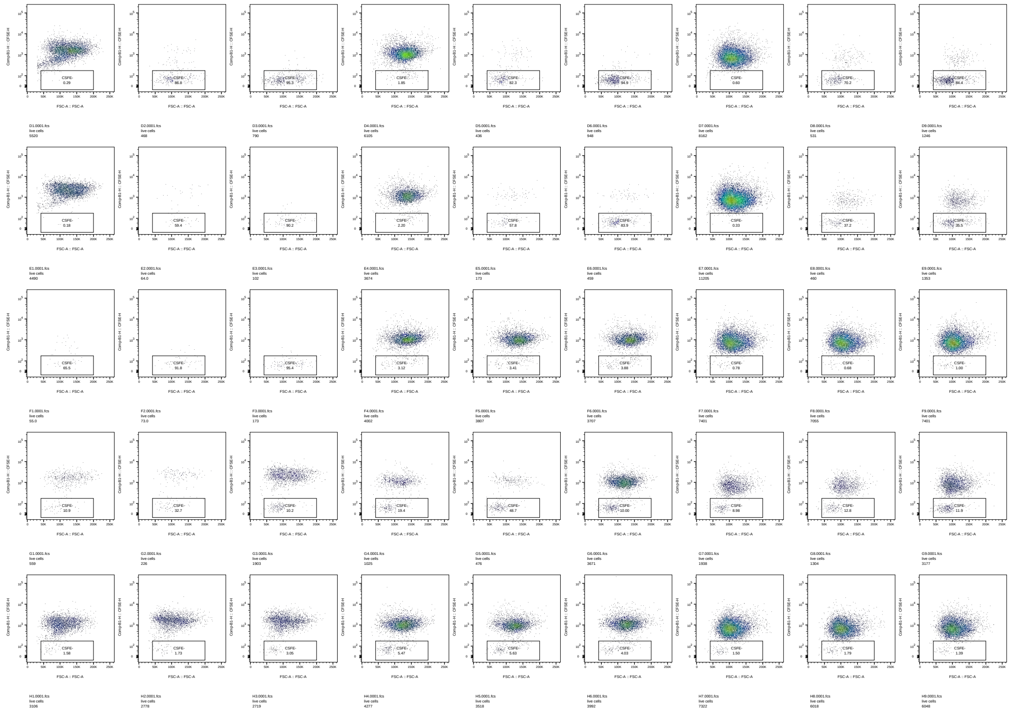
Comp-B1-H :: CFSE-H
FSC-A :: FSC-A
CSFE- 0.29
D1.0001.fcs
live cells
5520
CSFE- 86.8
D2.0001.fcs
live cells
468
CSFE- 95.3
D3.0001.fcs
live cells
790
CSFE- 1.85
D4.0001.fcs
live cells
6105
CSFE- 82.3
D5.0001.fcs
live cells
436
CSFE- 94.9
D6.0001.fcs
live cells
948
CSFE- 0.60
D7.0001.fcs
live cells
8162
CSFE- 70.2
D8.0001.fcs
live cells
531
CSFE- 84.4
D9.0001.fcs
live cells
1246
CSFE- 0.18
E1.0001.fcs
live cells
4490
CSFE- 59.4
E2.0001.fcs
live cells
64.0
CSFE- 90.2
E3.0001.fcs
live cells
102
CSFE- 2.20
E4.0001.fcs
live cells
3674
CSFE- 57.8
E5.0001.fcs
live cells
173
CSFE- 83.9
E6.0001.fcs
live cells
459
CSFE- 0.33
E7.0001.fcs
live cells
11205
CSFE- 37.2
E8.0001.fcs
live cells
460
CSFE- 35.5
E9.0001.fcs
live cells
1353
CSFE- 65.5
F1.0001.fcs
live cells
55.0
CSFE- 91.8
F2.0001.fcs
live cells
73.0
CSFE- 95.4
F3.0001.fcs
live cells
173
CSFE- 3.12
F4.0001.fcs
live cells
4002
CSFE- 3.41
F5.0001.fcs
live cells
3807
CSFE- 3.88
F6.0001.fcs
live cells
3707
CSFE- 0.78
F7.0001.fcs
live cells
7401
CSFE- 0.68
F8.0001.fcs
live cells
7055
CSFE- 1.00
F9.0001.fcs
live cells
7401
CSFE- 10.9
G1.0001.fcs
live cells
559
CSFE- 32.7
G2.0001.fcs
live cells
226
CSFE- 10.2
G3.0001.fcs
live cells
1903
CSFE- 19.4
G4.0001.fcs
live cells
1025
CSFE- 48.7
G5.0001.fcs
live cells
476
CSFE- 10.00
G6.0001.fcs
live cells
3671
CSFE- 8.98
G7.0001.fcs
live cells
1938
CSFE- 12.8
G8.0001.fcs
live cells
1304
CSFE- 11.9
G9.0001.fcs
live cells
3177
CSFE- 1.58
H1.0001.fcs
live cells
3106
CSFE- 1.73
H2.0001.fcs
live cells
2778
CSFE- 3.05
H3.0001.fcs
live cells
2719
CSFE- 5.47
H4.0001.fcs
live cells
4277
CSFE- 5.63
H5.0001.fcs
live cells
3518
CSFE- 4.03
H6.0001.fcs
live cells
3992
CSFE- 1.50
H7.0001.fcs
live cells
7322
CSFE- 1.79
H8.0001.fcs
live cells
6018
CSFE- 1.39
H9.0001.fcs
live cells
6048

Plate20_KFN-Prol_vs_HPB

**Well**	**Target**	**E:T ratio**	**Donor**	**CAR construct**
**1B**	HPB TRBC1+ve	1:4	Donor 7	KFN_Hinge_28z
**1D**	HPB TRBC1+ve	1:4	Donor 7	KFN_CD8STK_28z
**1E**	HPB TRBC1+ve	1:4	Donor 7	KFN_CD28STK_CD28TM_28z
**1F**	HPB TRBC1+ve	1:4	Donor 7	aCD19-CAR
**1G**	HPB TRBC1+ve	1:4	Donor 7	Non-transduced
**1H**	HPB TRBC1+ve	N/A	N/A	N/A
**2B**	HPB TRBC1+ve	1:4	Donor 8	KFN_Hinge_28z
**2D**	HPB TRBC1+ve	1:4	Donor 8	KFN_CD8STK_28z
**2E**	HPB TRBC1+ve	1:4	Donor 8	KFN_CD28STK_CD28TM_28z
**2F**	HPB TRBC1+ve	1:4	Donor 8	aCD19-CAR
**2G**	HPB TRBC1+ve	1:4	Donor 8	Non-transduced
**2H**	HPB TRBC1+ve	N/A	N/A	N/A
**3B**	HPB TRBC1+ve	1:4	Donor 9	KFN_Hinge_28z
**3D**	HPB TRBC1+ve	1:4	Donor 9	KFN_CD8STK_28z
**3E**	HPB TRBC1+ve	1:4	Donor 9	KFN_CD28STK_CD28TM_28z
**3F**	HPB TRBC1+ve	1:4	Donor 9	aCD19-CAR
**3G**	HPB TRBC1+ve	1:4	Donor 9	Non-transduced
**3H**	HPB TRBC1+ve	N/A	N/A	N/A
**4B**	HPB TRBC2+ve	1:4	Donor 7	KFN_Hinge_28z
**4D**	HPB TRBC2+ve	1:4	Donor 7	KFN_CD8STK_28z
**4E**	HPB TRBC2+ve	1:4	Donor 7	KFN_CD28STK_CD28TM_28z
**4F**	HPB TRBC2+ve	1:4	Donor 7	aCD19-CAR
**4G**	HPB TRBC2+ve	1:4	Donor 7	Non-transduced
**4H**	HPB TRBC2+ve	N/A	N/A	N/A
**5B**	HPB TRBC2+ve	1:4	Donor 8	KFN_Hinge_28z
**5D**	HPB TRBC2+ve	1:4	Donor 8	KFN_CD8STK_28z
**5E**	HPB TRBC2+ve	1:4	Donor 8	KFN_CD28STK_CD28TM_28z
**5F**	HPB TRBC2+ve	1:4	Donor 8	aCD19-CAR
**5G**	HPB TRBC2+ve	1:4	Donor 8	Non-transduced
**5H**	HPB TRBC2+ve	N/A	N/A	N/A
**6B**	HPB TRBC2+ve	1:4	Donor 9	KFN_Hinge_28z
**6D**	HPB TRBC2+ve	1:4	Donor 9	KFN_CD8STK_28z
**6E**	HPB TRBC2+ve	1:4	Donor 9	KFN_CD28STK_CD28TM_28z
**6F**	HPB TRBC2+ve	1:4	Donor 9	aCD19-CAR
**6G**	HPB TRBC2+ve	1:4	Donor 9	Non-transduced
**6H**	HPB TRBC2+ve	N/A	N/A	N/A
**7B**	HPB TRBC KO	1:4	Donor 7	KFN_Hinge_28z
**7D**	HPB TRBC KO	1:4	Donor 7	KFN_CD8STK_28z
**7E**	HPB TRBC KO	1:4	Donor 7	KFN_CD28STK_CD28TM_28z
**7F**	HPB TRBC KO	1:4	Donor 7	aCD19-CAR
**7G**	HPB TRBC KO	1:4	Donor 7	Non-transduced
**7H**	HPB TRBC KO	N/A	N/A	N/A
**8B**	HPB TRBC KO	1:4	Donor 8	KFN_Hinge_28z
**8D**	HPB TRBC KO	1:4	Donor 8	KFN_CD8STK_28z
**8E**	HPB TRBC KO	1:4	Donor 8	KFN_CD28STK_CD28TM_28z
**8F**	HPB TRBC KO	1:4	Donor 8	aCD19-CAR
**8G**	HPB TRBC KO	1:4	Donor 8	Non-transduced
**8H**	HPB TRBC KO	N/A	N/A	N/A

**9B**	HPB TRBC KO	1:4	Donor 9	KFN_Hinge_28z
**9D**	HPB TRBC KO	1:4	Donor 9	KFN_CD8STK_28z
**9E**	HPB TRBC KO	1:4	Donor 9	KFN_CD28STK_CD28TM_28z
**9F**	HPB TRBC KO	1:4	Donor 9	aCD19-CAR
**9G**	HPB TRBC KO	1:4	Donor 9	Non-transduced
**9H**	HPB TRBC KO	N/A	N/A	N/A
**10A**	N/A	N/A	N/A	N/A
**10B**	N/A	N/A	N/A	N/A
**10C**	N/A	N/A	N/A	N/A
**10D**	N/A	N/A	N/A	N/A
**10E**	N/A	N/A	N/A	N/A
**10F**	N/A	N/A	N/A	N/A
**10G**	N/A	N/A	N/A	N/A
**10H**	N/A	N/A	N/A	N/A
**11A**	N/A	N/A	N/A	N/A
**11B**	N/A	N/A	N/A	N/A
**11C**	N/A	N/A	N/A	N/A
**11D**	N/A	N/A	N/A	N/A
**11E**	N/A	N/A	N/A	N/A
**11F**	N/A	N/A	N/A	N/A
**11G**	N/A	N/A	N/A	N/A
**11H**	N/A	N/A	N/A	N/A
**12A**	N/A	N/A	N/A	N/A
**12B**	N/A	N/A	N/A	N/A
**12C**	N/A	N/A	N/A	N/A
**12D**	N/A	N/A	N/A	N/A
**12E**	N/A	N/A	N/A	N/A
**12F**	N/A	N/A	N/A	N/A
**12G**	N/A	N/A	N/A	N/A
**12H**	N/A	N/A	N/A	N/A

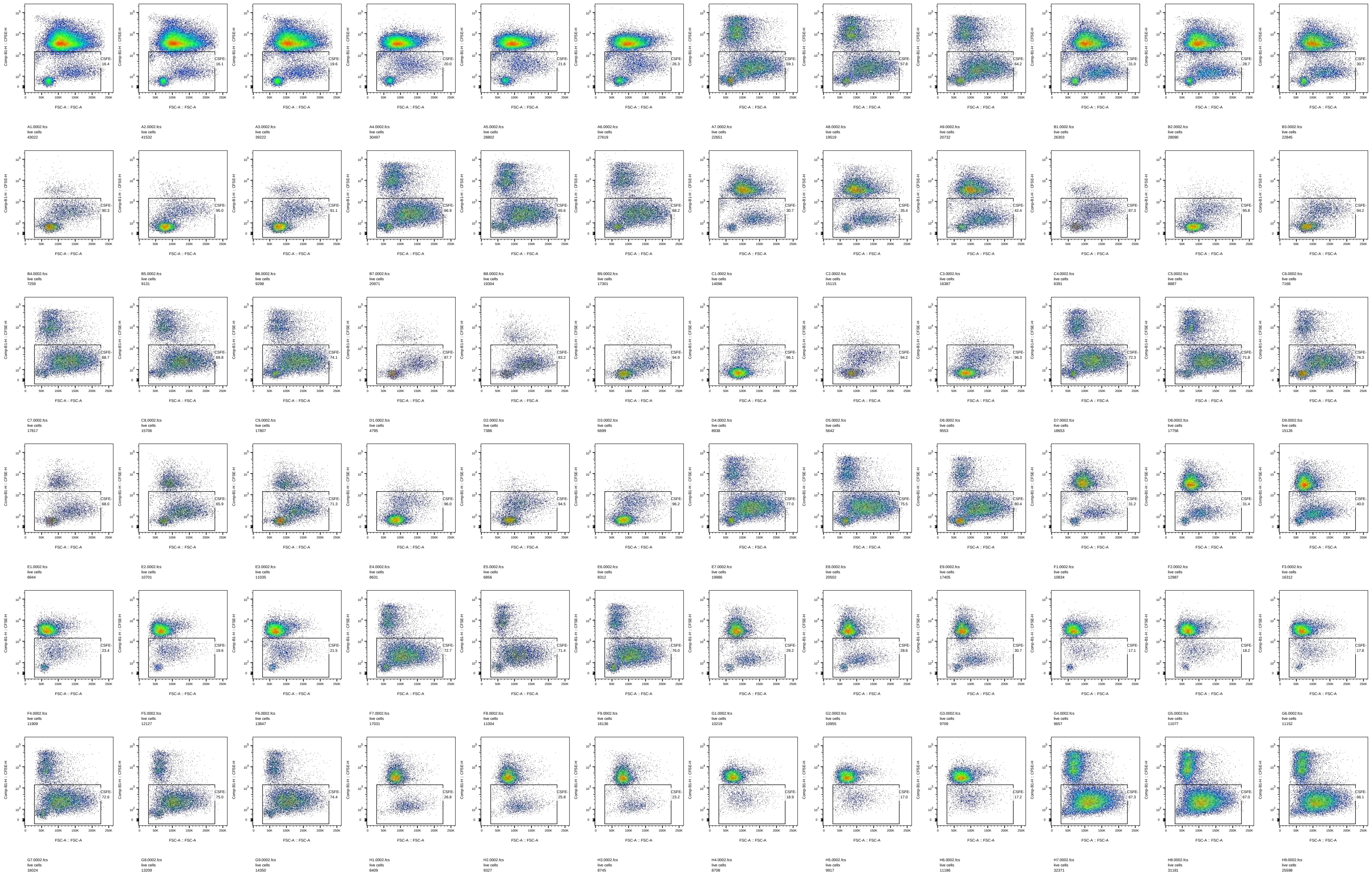
A1.0002.fcs
live cells
43022
A2.0002.fcs
live cells
41532
A3.0002.fcs
live cells
39222
A4.0002.fcs
live cells
30497
A5.0002.fcs
live cells
28802
A6.0002.fcs
live cells
27619
A7.0002.fcs
live cells
22651
A8.0002.fcs
live cells
19519
A9.0002.fcs
live cells
20732
B1.0002.fcs
live cells
26303
B2.0002.fcs
live cells
28090
B3.0002.fcs
live cells
22845
B4.0002.fcs
live cells
7259
B5.0002.fcs
live cells
9131
B6.0002.fcs
live cells
9298
B7.0002.fcs
live cells
20971
B8.0002.fcs
live cells
19304
B9.0002.fcs
live cells
17301
C1.0002.fcs
live cells
14096
C2.0002.fcs
live cells
15115
C3.0002.fcs
live cells
16387
C4.0002.fcs
live cells
6391
C5.0002.fcs
live cells
8887
C6.0002.fcs
live cells
7168
C7.0002.fcs
live cells
17817
C8.0002.fcs
live cells
15706
C9.0002.fcs
live cells
17807
D1.0002.fcs
live cells
4795
D2.0002.fcs
live cells
7386
D3.0002.fcs
live cells
6699
D4.0002.fcs
live cells
8938
D5.0002.fcs
live cells
5642
D6.0002.fcs
live cells
9553
D7.0002.fcs
live cells
18653
D8.0002.fcs
live cells
17756
D9.0002.fcs
live cells
15126
E1.0002.fcs
live cells
6944
E2.0002.fcs
live cells
10701
E3.0002.fcs
live cells
11035
E4.0002.fcs
live cells
8631
E5.0002.fcs
live cells
6856
E6.0002.fcs
live cells
8312
E7.0002.fcs
live cells
19986
E8.0002.fcs
live cells
20502
E9.0002.fcs
live cells
17405
F1.0002.fcs
live cells
10834
F2.0002.fcs
live cells
12987
F3.0002.fcs
live cells
16312
F4.0002.fcs
live cells
11909
F5.0002.fcs
live cells
12127
F6.0002.fcs
live cells
13847
F7.0002.fcs
live cells
17031
F8.0002.fcs
live cells
11004
F9.0002.fcs
live cells
16136
G1.0002.fcs
live cells
10219
G2.0002.fcs
live cells
10955
G3.0002.fcs
live cells
9709
G4.0002.fcs
live cells
9657
G5.0002.fcs
live cells
11077
G6.0002.fcs
live cells
11152
G7.0002.fcs
live cells
16024
G8.0002.fcs
live cells
13209
G9.0002.fcs
live cells
14350
H1.0002.fcs
live cells
8409
H2.0002.fcs
live cells
9327
H3.0002.fcs
live cells
8745
H4.0002.fcs
live cells
8708
H5.0002.fcs
live cells
9917
H6.0002.fcs
live cells
11186
H7.0002.fcs
live cells
32371
H8.0002.fcs
live cells
31181
H9.0002.fcs
live cells
25598

Plate19_KFN0Prol_vs_Jurkat

Well	Target	E:T ratio	Donor	CAR construct
**1B**	Jurkat TRBC1+ve	1:4	Donor 7	KFN_Hinge_28z
**1D**	Jurkat TRBC1+ve	1:4	Donor 7	KFN_CD8STK_28z
**1E**	Jurkat TRBC1+ve	1:4	Donor 7	KFN_CD28STK_CD28TM_28z
**1F**	Jurkat TRBC1+ve	1:4	Donor 7	aCD19-CAR
**1G**	Jurkat TRBC1+ve	1:4	Donor 7	Non-transduced
**1H**	Jurkat TRBC1+ve	N/A	N/A	N/A
**2B**	Jurkat TRBC1+ve	1:4	Donor 8	KFN_Hinge_28z
**2D**	Jurkat TRBC1+ve	1:4	Donor 8	KFN_CD8STK_28z
**2E**	Jurkat TRBC1+ve	1:4	Donor 8	KFN_CD28STK_CD28TM_28z
**2F**	Jurkat TRBC1+ve	1:4	Donor 8	aCD19-CAR
**2G**	Jurkat TRBC1+ve	1:4	Donor 8	Non-transduced
**2H**	Jurkat TRBC1+ve	N/A	N/A	N/A
**3B**	Jurkat TRBC1+ve	1:4	Donor 9	KFN_Hinge_28z
**3D**	Jurkat TRBC1+ve	1:4	Donor 9	KFN_CD8STK_28z
**3E**	Jurkat TRBC1+ve	1:4	Donor 9	KFN_CD28STK_CD28TM_28z
**3F**	Jurkat TRBC1+ve	1:4	Donor 9	aCD19-CAR
**3G**	Jurkat TRBC1+ve	1:4	Donor 9	Non-transduced
**3H**	Jurkat TRBC1+ve	N/A	N/A	N/A
**4B**	Jurkat TRBC2+ve	1:4	Donor 7	KFN_Hinge_28z
**4D**	Jurkat TRBC2+ve	1:4	Donor 7	KFN_CD8STK_28z
**4E**	Jurkat TRBC2+ve	1:4	Donor 7	KFN_CD28STK_CD28TM_28z
**4F**	Jurkat TRBC2+ve	1:4	Donor 7	aCD19-CAR
**4G**	Jurkat TRBC2+ve	1:4	Donor 7	Non-transduced
**4H**	Jurkat TRBC2+ve	N/A	N/A	N/A
**5B**	Jurkat TRBC2+ve	1:4	Donor 8	KFN_Hinge_28z
**5D**	Jurkat TRBC2+ve	1:4	Donor 8	KFN_CD8STK_28z
**5E**	Jurkat TRBC2+ve	1:4	Donor 8	KFN_CD28STK_CD28TM_28z
**5F**	Jurkat TRBC2+ve	1:4	Donor 8	aCD19-CAR
**5G**	Jurkat TRBC2+ve	1:4	Donor 8	Non-transduced
**5H**	Jurkat TRBC2+ve	N/A	N/A	N/A
**6B**	Jurkat TRBC2+ve	1:4	Donor 9	KFN_Hinge_28z
**6D**	Jurkat TRBC2+ve	1:4	Donor 9	KFN_CD8STK_28z
**6E**	Jurkat TRBC2+ve	1:4	Donor 9	KFN_CD28STK_CD28TM_28z
**6F**	Jurkat TRBC2+ve	1:4	Donor 9	aCD19-CAR
**6G**	Jurkat TRBC2+ve	1:4	Donor 9	Non-transduced
**6H**	Jurkat TRBC2+ve	N/A	N/A	N/A
**7B**	Jurkat TRBC KO	1:4	Donor 7	KFN_Hinge_28z
**7D**	Jurkat TRBC KO	1:4	Donor 7	KFN_CD8STK_28z
**7E**	Jurkat TRBC KO	1:4	Donor 7	KFN_CD28STK_CD28TM_28z
**7F**	Jurkat TRBC KO	1:4	Donor 7	aCD19-CAR
**7G**	Jurkat TRBC KO	1:4	Donor 7	Non-transduced
**7H**	Jurkat TRBC KO	N/A	N/A	N/A
**8B**	Jurkat TRBC KO	1:4	Donor 8	KFN_Hinge_28z
**8D**	Jurkat TRBC KO	1:4	Donor 8	KFN_CD8STK_28z
**8E**	Jurkat TRBC KO	1:4	Donor 8	KFN_CD28STK_CD28TM_28z
**8F**	Jurkat TRBC KO	1:4	Donor 8	aCD19-CAR
**8G**	Jurkat TRBC KO	1:4	Donor 8	Non-transduced
**8H**	Jurkat TRBC KO	N/A	N/A	N/A

**9B**	Jurkat TRBC KO	1:4	Donor 9	KFN_Hinge_28z
**9D**	Jurkat TRBC KO	1:4	Donor 9	KFN_CD8STK_28z
**9E**	Jurkat TRBC KO	1:4	Donor 9	KFN_CD28STK_CD28TM_28z
**9F**	Jurkat TRBC KO	1:4	Donor 9	aCD19-CAR
**9G**	Jurkat TRBC KO	1:4	Donor 9	Non-transduced
**9H**	Jurkat TRBC KO	N/A	N/A	N/A
**10A**	N/A	N/A	N/A	N/A
**10B**	N/A	N/A	N/A	N/A
**10C**	N/A	N/A	N/A	N/A
**10D**	N/A	N/A	N/A	N/A
**10E**	N/A	N/A	N/A	N/A
**10F**	N/A	N/A	N/A	N/A
**10G**	N/A	N/A	N/A	N/A
**10H**	N/A	N/A	N/A	N/A
**11A**	N/A	N/A	N/A	N/A
**11B**	N/A	N/A	N/A	N/A
**11C**	N/A	N/A	N/A	N/A
**11D**	N/A	N/A	N/A	N/A
**11E**	N/A	N/A	N/A	N/A
**11F**	N/A	N/A	N/A	N/A
**11G**	N/A	N/A	N/A	N/A
**11H**	N/A	N/A	N/A	N/A
**12A**	N/A	N/A	N/A	N/A
**12B**	N/A	N/A	N/A	N/A
**12C**	N/A	N/A	N/A	N/A
**12D**	N/A	N/A	N/A	N/A
**12E**	N/A	N/A	N/A	N/A
**12F**	N/A	N/A	N/A	N/A
**12G**	N/A	N/A	N/A	N/A
**12H**	N/A	N/A	N/A	N/A

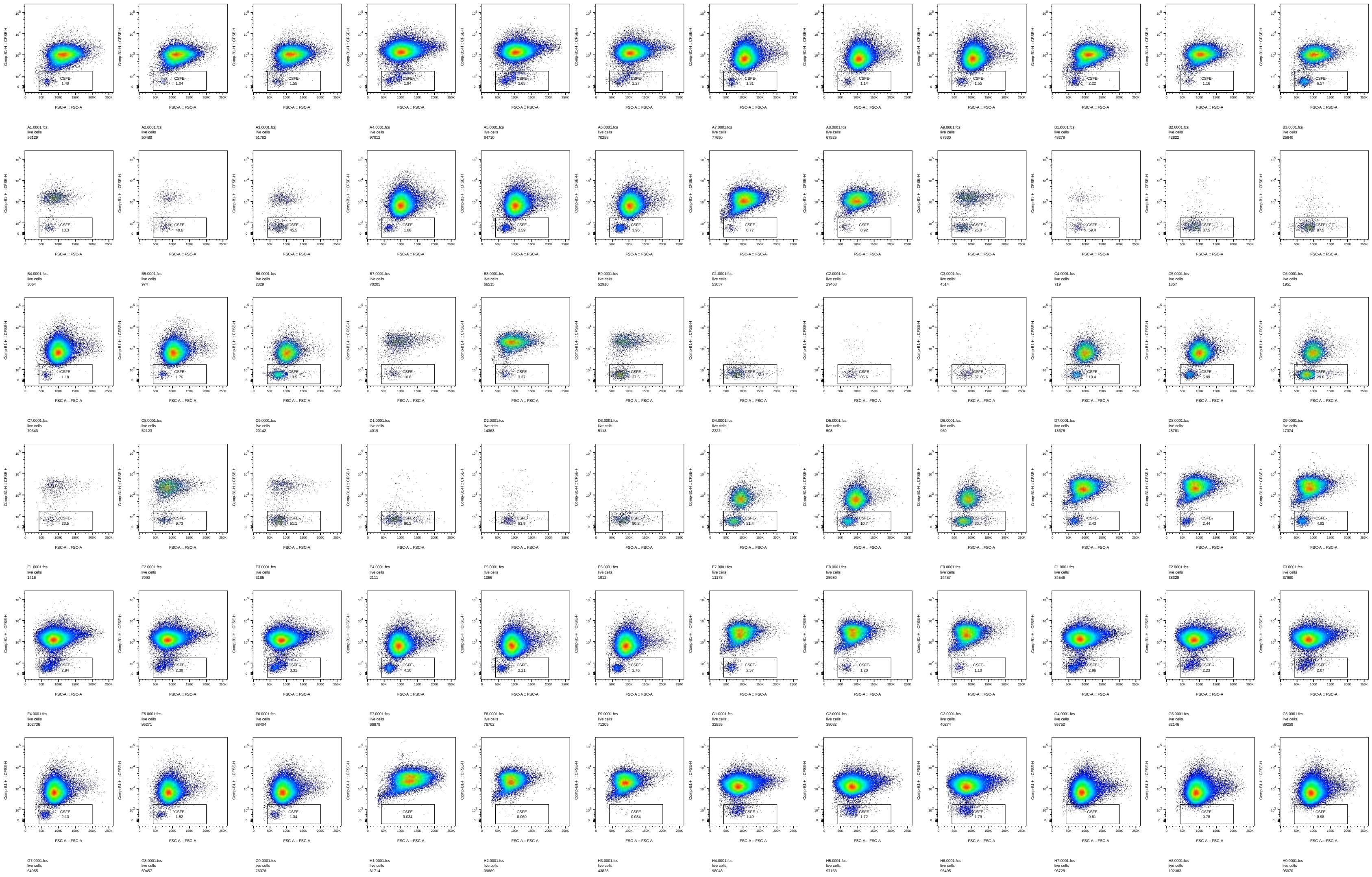

Comp-B1-H :: CFSE-H
FSC-A :: FSC-A
CSFE- 1.40
A1.0001.fcs
live cells
56129
CSFE- 1.04
A2.0001.fcs
live cells
50480
CSFE- 1.55
A3.0001.fcs
live cells
51782
CSFE- 1.94
A4.0001.fcs
live cells
97012
CSFE- 2.65
A5.0001.fcs
live cells
84710
CSFE- 2.27
A6.0001.fcs
live cells
70258
CSFE- 1.31
A7.0001.fcs
live cells
77650
CSFE- 1.14
A8.0001.fcs
live cells
67525
CSFE- 1.55
A9.0001.fcs
live cells
67630
CSFE- 2.22
B1.0001.fcs
live cells
49278
CSFE- 1.16
B2.0001.fcs
live cells
42822
CSFE- 6.57
B3.0001.fcs
live cells
26640
CSFE- 13.3
B4.0001.fcs
live cells
3064
CSFE- 40.6
B5.0001.fcs
live cells
974
CSFE- 45.5
B6.0001.fcs
live cells
2329
CSFE- 1.68
B7.0001.fcs
live cells
70205
CSFE- 2.59
B8.0001.fcs
live cells
66515
CSFE- 3.96
B9.0001.fcs
live cells
52910
CSFE- 0.77
C1.0001.fcs
live cells
53037
CSFE- 0.92
C2.0001.fcs
live cells
29468
CSFE- 26.0
C3.0001.fcs
live cells
4514
CSFE- 59.4
C4.0001.fcs
live cells
719
CSFE- 87.5
C5.0001.fcs
live cells
1857
CSFE- 87.5
C6.0001.fcs
live cells
1951
CSFE- 1.18
C7.0001.fcs
live cells
70343
CSFE- 1.76
C8.0001.fcs
live cells
52123
CSFE- 13.5
C9.0001.fcs
live cells
20142
CSFE- 10.8
D1.0001.fcs
live cells
4019
CSFE- 3.37
D2.0001.fcs
live cells
14363
CSFE- 37.5
D3.0001.fcs
live cells
5118
CSFE- 89.6
D4.0001.fcs
live cells
2322
CSFE- 85.6
D5.0001.fcs
live cells
508
CSFE- 87.6
D6.0001.fcs
live cells
969
CSFE- 10.4
D7.0001.fcs
live cells
13678
CSFE- 5.99
D8.0001.fcs
live cells
28781
CSFE- 29.0
D9.0001.fcs
live cells
17374
CSFE- 23.5
E1.0001.fcs
live cells
1416
CSFE- 9.73
E2.0001.fcs
live cells
7090
CSFE- 51.1
E3.0001.fcs
live cells
3185
CSFE- 90.2
E4.0001.fcs
live cells
2111
CSFE- 83.9
E5.0001.fcs
live cells
1066
CSFE- 90.8
E6.0001.fcs
live cells
1912
CSFE- 21.4
E7.0001.fcs
live cells
11173
CSFE- 10.7
E8.0001.fcs
live cells
25980
CSFE- 30.7
E9.0001.fcs
live cells
14487
CSFE- 3.43
F1.0001.fcs
live cells
34546
CSFE- 2.44
F2.0001.fcs
live cells
38329
CSFE- 4.92
F3.0001.fcs
live cells
37980
CSFE- 2.94
F4.0001.fcs
live cells
102736
CSFE- 2.38
F5.0001.fcs
live cells
95271
CSFE- 3.31
F6.0001.fcs
live cells
88404
CSFE- 4.10
F7.0001.fcs
live cells
66879
CSFE- 2.21
F8.0001.fcs
live cells
76702
CSFE- 2.76
F9.0001.fcs
live cells
71205
CSFE- 2.57
G1.0001.fcs
live cells
32855
CSFE- 1.20
G2.0001.fcs
live cells
38082
CSFE- 1.10
G3.0001.fcs
live cells
40274
CSFE- 2.96
G4.0001.fcs
live cells
95752
CSFE- 2.23
G5.0001.fcs
live cells
82146
CSFE- 2.07
G6.0001.fcs
live cells
89259
CSFE- 2.13
G7.0001.fcs
live cells
64955
CSFE- 1.52
G8.0001.fcs
live cells
59457
CSFE- 1.34
G9.0001.fcs
live cells
76378
CSFE- 0.034
H1.0001.fcs
live cells
61714
CSFE- 0.060
H2.0001.fcs
live cells
39889
CSFE- 0.084
H3.0001.fcs
live cells
43828
CSFE- 1.49
H4.0001.fcs
live cells
98048
CSFE- 1.72
H5.0001.fcs
live cells
97163
CSFE- 1.79
H6.0001.fcs
live cells
96495
CSFE- 0.81
H7.0001.fcs
live cells
96728
CSFE- 0.78
H8.0001.fcs
live cells
102383
CSFE- 0.98
H9.0001.fcs
live cells
95070

Plate10_JOVI_vs_Jurkat_TRBC1

Well	Target	E:T ratio	Donor	CAR construct
**1A**	Jurkat TRBC 1+ve	1:4	Donor 7	JOVI_Hinge_41bbz
**1D**	Jurkat TRBC 1+ve	1:4	Donor 7	JOVI_CD8STK_28z
**1E**	Jurkat TRBC 1+ve	1:4	Donor 7	JOVI_CD28STK_CD28TM_28z
**1F**	Jurkat TRBC 1+ve	1:4	Donor 7	mJOVI_Hinge_41bbz
**1G**	Jurkat TRBC 1+ve	1:4	Donor 7	aCD19-CAR
**1H**	Jurkat TRBC 1+ve	1:4	Donor 7	Non-transduced
**2A**	Jurkat TRBC 1+ve	1:8	Donor 7	JOVI_Hinge_41bbz
**2D**	Jurkat TRBC 1+ve	1:8	Donor 7	JOVI_CD8STK_28z
**2E**	Jurkat TRBC 1+ve	1:8	Donor 7	JOVI_CD28STK_CD28TM_28z
**2F**	Jurkat TRBC 1+ve	1:8	Donor 7	mJOVI_Hinge_41bbz
**2G**	Jurkat TRBC 1+ve	1:8	Donor 7	aCD19-CAR
**2H**	Jurkat TRBC 1+ve	1:8	Donor 7	Non-transduced
**5A**	Jurkat TRBC 1+ve	1:4	Donor 8	JOVI_Hinge_41bbz
**5D**	Jurkat TRBC 1+ve	1:4	Donor 8	JOVI_CD8STK_28z
**5E**	Jurkat TRBC 1+ve	1:4	Donor 8	JOVI_CD28STK_CD28TM_28z
**5F**	Jurkat TRBC 1+ve	1:4	Donor 8	mJOVI_Hinge_41bbz
**5G**	Jurkat TRBC 1+ve	1:4	Donor 8	aCD19-CAR
**5H**	Jurkat TRBC 1+ve	1:4	Donor 8	Non-transduced
**6A**	Jurkat TRBC 1+ve	1:8	Donor 8	JOVI_Hinge_41bbz
**6D**	Jurkat TRBC 1+ve	1:8	Donor 8	JOVI_CD8STK_28z
**6E**	Jurkat TRBC 1+ve	1:8	Donor 8	JOVI_CD28STK_CD28TM_28z
**6F**	Jurkat TRBC 1+ve	1:8	Donor 8	mJOVI_Hinge_41bbz
**6G**	Jurkat TRBC 1+ve	1:8	Donor 8	aCD19-CAR
**6H**	Jurkat TRBC 1+ve	1:8	Donor 8	Non-transduced
**9A**	Jurkat TRBC 1+ve	1:4	Donor 9	JOVI_Hinge_41bbz
**9D**	Jurkat TRBC 1+ve	1:4	Donor 9	JOVI_CD8STK_28z
**9E**	Jurkat TRBC 1+ve	1:4	Donor 9	JOVI_CD28STK_CD28TM_28z
**9F**	Jurkat TRBC 1+ve	1:4	Donor 9	mJOVI_Hinge_41bbz
**9G**	Jurkat TRBC 1+ve	1:4	Donor 9	aCD19-CAR
**9H**	Jurkat TRBC 1+ve	1:4	Donor 9	Non-transduced
**10A**	Jurkat TRBC 1+ve	1:8	Donor 9	JOVI_Hinge_41bbz
**10D**	Jurkat TRBC 1+ve	1:8	Donor 9	JOVI_CD8STK_28z
**10E**	Jurkat TRBC 1+ve	1:8	Donor 9	JOVI_CD28STK_CD28TM_28z
**10F**	Jurkat TRBC 1+ve	1:8	Donor 9	mJOVI_Hinge_41bbz
**10G**	Jurkat TRBC 1+ve	1:8	Donor 9	aCD19-CAR
**10H**	Jurkat TRBC 1+ve	1:8	Donor 9	Non-transduced

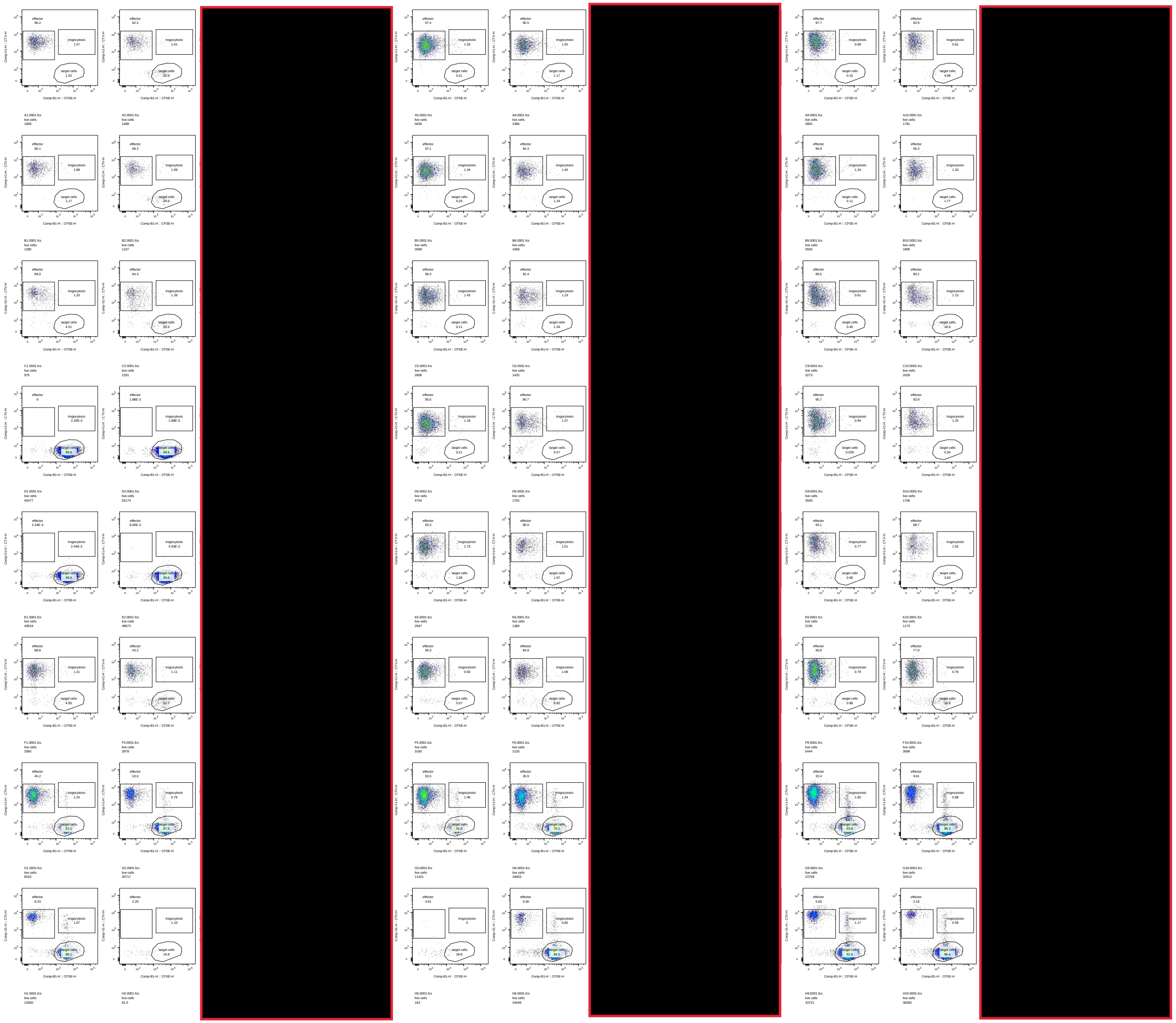

[redacted]

Plate11_JOVI_vs_TRBC2

**Well**	**Target**	**E:T ratio**	**Donor**	**CAR construct**
**1A**	Jurkat TRBC 2+ve	1:4	Donor 7	JOVI_Hinge_41bbz
**1D**	Jurkat TRBC 2+ve	1:4	Donor 7	JOVI_CD8STK_28z
**1E**	Jurkat TRBC 2+ve	1:4	Donor 7	JOVI_CD28STK_CD28TM_28z
**1F**	Jurkat TRBC 2+ve	1:4	Donor 7	mJOVI_Hinge_41bbz
**1G**	Jurkat TRBC 2+ve	1:4	Donor 7	aCD19-CAR
**1H**	Jurkat TRBC 2+ve	1:4	Donor 7	Non-transduced
**2A**	Jurkat TRBC 2+ve	1:8	Donor 7	JOVI_Hinge_41bbz
**2D**	Jurkat TRBC 2+ve	1:8	Donor 7	JOVI_CD8STK_28z
**2E**	Jurkat TRBC 2+ve	1:8	Donor 7	JOVI_CD28STK_CD28TM_28z
**2F**	Jurkat TRBC 2+ve	1:8	Donor 7	mJOVI_Hinge_41bbz
**2G**	Jurkat TRBC 2+ve	1:8	Donor 7	aCD19-CAR
**2H**	Jurkat TRBC 2+ve	1:8	Donor 7	Non-transduced
**5A**	Jurkat TRBC 2+ve	1:4	Donor 8	JOVI_Hinge_41bbz
**5D**	Jurkat TRBC 2+ve	1:4	Donor 8	JOVI_CD8STK_28z
**5E**	Jurkat TRBC 2+ve	1:4	Donor 8	JOVI_CD28STK_CD28TM_28z
**5F**	Jurkat TRBC 2+ve	1:4	Donor 8	mJOVI_Hinge_41bbz
**5G**	Jurkat TRBC 2+ve	1:4	Donor 8	aCD19-CAR
**5H**	Jurkat TRBC 2+ve	1:4	Donor 8	Non-transduced
**6A**	Jurkat TRBC 2+ve	1:8	Donor 8	JOVI_Hinge_41bbz
**6D**	Jurkat TRBC 2+ve	1:8	Donor 8	JOVI_CD8STK_28z
**6E**	Jurkat TRBC 2+ve	1:8	Donor 8	JOVI_CD28STK_CD28TM_28z
**6F**	Jurkat TRBC 2+ve	1:8	Donor 8	mJOVI_Hinge_41bbz
**6G**	Jurkat TRBC 2+ve	1:8	Donor 8	aCD19-CAR
**6H**	Jurkat TRBC 2+ve	1:8	Donor 8	Non-transduced
**9A**	Jurkat TRBC 2+ve	1:4	Donor 9	JOVI_Hinge_41bbz
**9D**	Jurkat TRBC 2+ve	1:4	Donor 9	JOVI_CD8STK_28z
**9E**	Jurkat TRBC 2+ve	1:4	Donor 9	JOVI_CD28STK_CD28TM_28z
**9F**	Jurkat TRBC 2+ve	1:4	Donor 9	mJOVI_Hinge_41bbz
**9G**	Jurkat TRBC 2+ve	1:4	Donor 9	aCD19-CAR
**9H**	Jurkat TRBC 2+ve	1:4	Donor 9	Non-transduced
**10A**	Jurkat TRBC 2+ve	1:8	Donor 9	JOVI_Hinge_41bbz
**10D**	Jurkat TRBC 2+ve	1:8	Donor 9	JOVI_CD8STK_28z
**10E**	Jurkat TRBC 2+ve	1:8	Donor 9	JOVI_CD28STK_CD28TM_28z
**10F**	Jurkat TRBC 2+ve	1:8	Donor 9	mJOVI_Hinge_41bbz
**10G**	Jurkat TRBC 2+ve	1:8	Donor 9	aCD19-CAR
**10H**	Jurkat TRBC 2+ve	1:8	Donor 9	Non-transduced

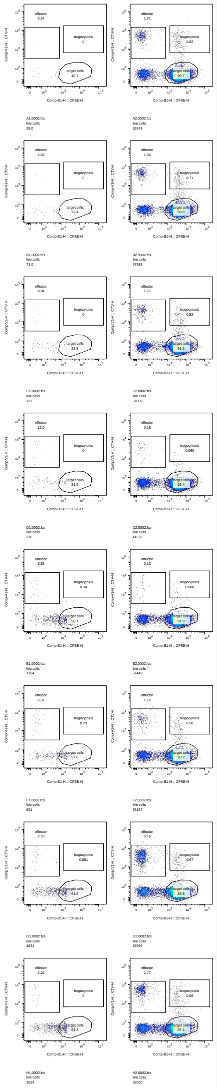

[redacted]

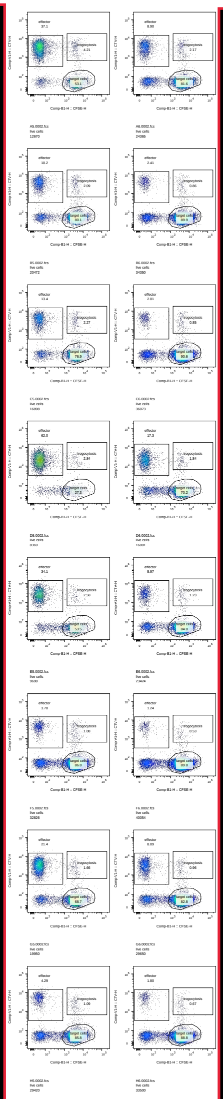

[redacted]

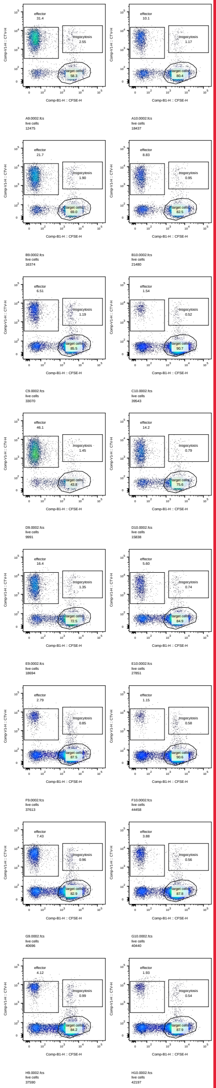

[redacted]

Plate12_JOVI_vs_Jurkat_TRBC-KO

Well	Target	E:T ratio	Donor	CAR construct
**1A**	Jurkat TRBC KO	1:4	Donor 7	JOVI_Hinge_41bbz
**1D**	Jurkat TRBC KO	1:4	Donor 7	JOVI_CD8STK_28z
**1E**	Jurkat TRBC KO	1:4	Donor 7	JOVI_CD28STK_CD28TM_28z
**1F**	Jurkat TRBC KO	1:4	Donor 7	mJOVI_Hinge_41bbz
**1G**	Jurkat TRBC KO	1:4	Donor 7	aCD19-CAR
**1H**	Jurkat TRBC KO	1:4	Donor 7	Non-transduced
**2A**	Jurkat TRBC KO	1:8	Donor 7	JOVI_Hinge_41bbz
**2D**	Jurkat TRBC KO	1:8	Donor 7	JOVI_CD8STK_28z
**2E**	Jurkat TRBC KO	1:8	Donor 7	JOVI_CD28STK_CD28TM_28z
**2F**	Jurkat TRBC KO	1:8	Donor 7	mJOVI_Hinge_41bbz
**2G**	Jurkat TRBC KO	1:8	Donor 7	aCD19-CAR
**2H**	Jurkat TRBC KO	1:8	Donor 7	Non-transduced
**5A**	Jurkat TRBC KO	1:4	Donor 8	JOVI_Hinge_41bbz
**5D**	Jurkat TRBC KO	1:4	Donor 8	JOVI_CD8STK_28z
**5E**	Jurkat TRBC KO	1:4	Donor 8	JOVI_CD28STK_CD28TM_28z
**5F**	Jurkat TRBC KO	1:4	Donor 8	mJOVI_Hinge_41bbz
**5G**	Jurkat TRBC KO	1:4	Donor 8	aCD19-CAR
**5H**	Jurkat TRBC KO	1:4	Donor 8	Non-transduced
**6A**	Jurkat TRBC KO	1:8	Donor 8	JOVI_Hinge_41bbz
**6D**	Jurkat TRBC KO	1:8	Donor 8	JOVI_CD8STK_28z
**6E**	Jurkat TRBC KO	1:8	Donor 8	JOVI_CD28STK_CD28TM_28z
**6F**	Jurkat TRBC KO	1:8	Donor 8	mJOVI_Hinge_41bbz
**6G**	Jurkat TRBC KO	1:8	Donor 8	aCD19-CAR
**6H**	Jurkat TRBC KO	1:8	Donor 8	Non-transduced
**9A**	Jurkat TRBC KO	1:4	Donor 9	JOVI_Hinge_41bbz
**9D**	Jurkat TRBC KO	1:4	Donor 9	JOVI_CD8STK_28z
**9E**	Jurkat TRBC KO	1:4	Donor 9	JOVI_CD28STK_CD28TM_28z
**9F**	Jurkat TRBC KO	1:4	Donor 9	mJOVI_Hinge_41bbz
**9G**	Jurkat TRBC KO	1:4	Donor 9	aCD19-CAR
**9H**	Jurkat TRBC KO	1:4	Donor 9	Non-transduced
**10A**	Jurkat TRBC KO	1:8	Donor 9	JOVI_Hinge_41bbz
**10D**	Jurkat TRBC KO	1:8	Donor 9	JOVI_CD8STK_28z
**10E**	Jurkat TRBC KO	1:8	Donor 9	JOVI_CD28STK_CD28TM_28z
**10F**	Jurkat TRBC KO	1:8	Donor 9	mJOVI_Hinge_41bbz
**10G**	Jurkat TRBC KO	1:8	Donor 9	aCD19-CAR
**10H**	Jurkat TRBC KO	1:8	Donor 9	Non-transduced

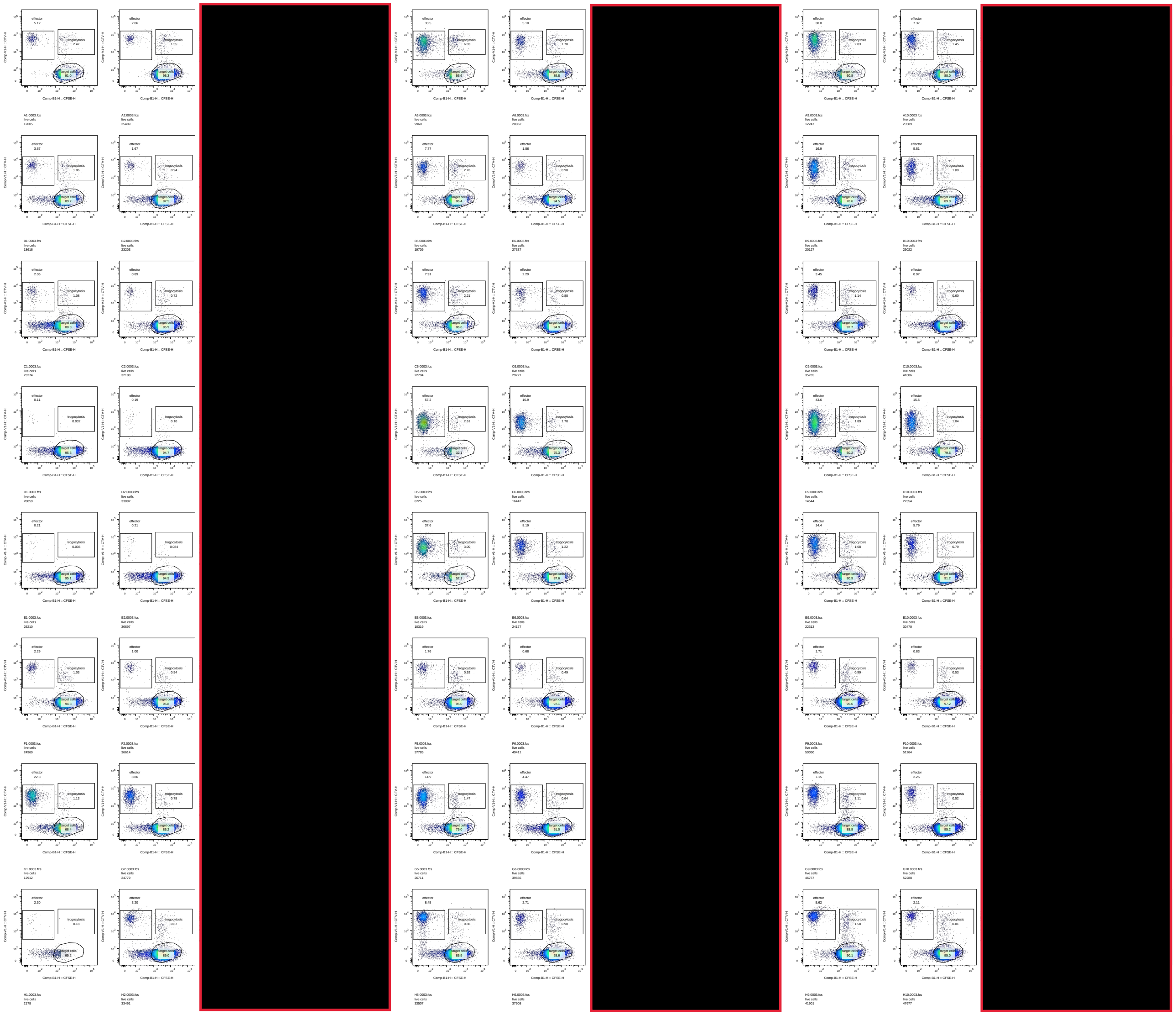

Supplement: Supplementary file 3 — Source Data [file 41467_2024_45854_MOESM3_ESM.zip › Source data file 2.pdf]
