# Supplementary material for: Structure-guided engineering of immunotherapies targeting TRBC1 and TRBC2 in T cell malignancies
Source: Nat Commun. 2024 Feb 21;15:1583. doi: 10.1038/s41467-024-45854-3 (PMC10881500; doi:10.1038/s41467-024-45854-3)
Supplement: Supplementary file 3 — Source Data [file 41467_2024_45854_MOESM3_ESM.zip › Source data file 3.pdf]

Plate13_JOVI_vs_HPB_TRBC1

**Well**	**Target**	**E:T ratio**	**Donor**	**CAR construct**
**1A**	HPB TRBC1+ve	1:4	Donor 7	JOVI_Hinge_41bbz
**1D**	HPB TRBC1+ve	1:4	Donor 7	JOVI_CD8STK_28z
**1E**	HPB TRBC1+ve	1:4	Donor 7	JOVI_CD28STK_CD28TM_28z
**1G**	HPB TRBC1+ve	1:4	Donor 7	aCD19-CAR
**1H**	HPB TRBC1+ve	1:4	Donor 7	Non-transduced
**2A**	HPB TRBC1+ve	1:8	Donor 7	JOVI_Hinge_41bbz
**2D**	HPB TRBC1+ve	1:8	Donor 7	JOVI_CD8STK_28z
**2E**	HPB TRBC1+ve	1:8	Donor 7	JOVI_CD28STK_CD28TM_28z
**2G**	HPB TRBC1+ve	1:8	Donor 7	aCD19-CAR
**2H**	HPB TRBC1+ve	1:8	Donor 7	Non-transduced
**5A**	HPB TRBC1+ve	1:4	Donor 8	JOVI_Hinge_41bbz
**5D**	HPB TRBC1+ve	1:4	Donor 8	JOVI_CD8STK_28z
**5E**	HPB TRBC1+ve	1:4	Donor 8	JOVI_CD28STK_CD28TM_28z
**5G**	HPB TRBC1+ve	1:4	Donor 8	aCD19-CAR
**5H**	HPB TRBC1+ve	1:4	Donor 8	Non-transduced
**6A**	HPB TRBC1+ve	1:8	Donor 8	JOVI_Hinge_41bbz
**6D**	HPB TRBC1+ve	1:8	Donor 8	JOVI_CD8STK_28z
**6E**	HPB TRBC1+ve	1:8	Donor 8	JOVI_CD28STK_CD28TM_28z
**6G**	HPB TRBC1+ve	1:8	Donor 8	aCD19-CAR
**6H**	HPB TRBC1+ve	1:8	Donor 8	Non-transduced
**9A**	HPB TRBC1+ve	1:4	Donor 9	JOVI_Hinge_41bbz
**9D**	HPB TRBC1+ve	1:4	Donor 9	JOVI_CD8STK_28z
**9E**	HPB TRBC1+ve	1:4	Donor 9	JOVI_CD28STK_CD28TM_28z
**9G**	HPB TRBC1+ve	1:4	Donor 9	aCD19-CAR
**9H**	HPB TRBC1+ve	1:4	Donor 9	Non-transduced
**10A**	HPB TRBC1+ve	1:8	Donor 9	JOVI_Hinge_41bbz
**10D**	HPB TRBC1+ve	1:8	Donor 9	JOVI_CD8STK_28z
**10E**	HPB TRBC1+ve	1:8	Donor 9	JOVI_CD28STK_CD28TM_28z
**10G**	HPB TRBC1+ve	1:8	Donor 9	aCD19-CAR
**10H**	HPB TRBC1+ve	1:8	Donor 9	Non-transduced

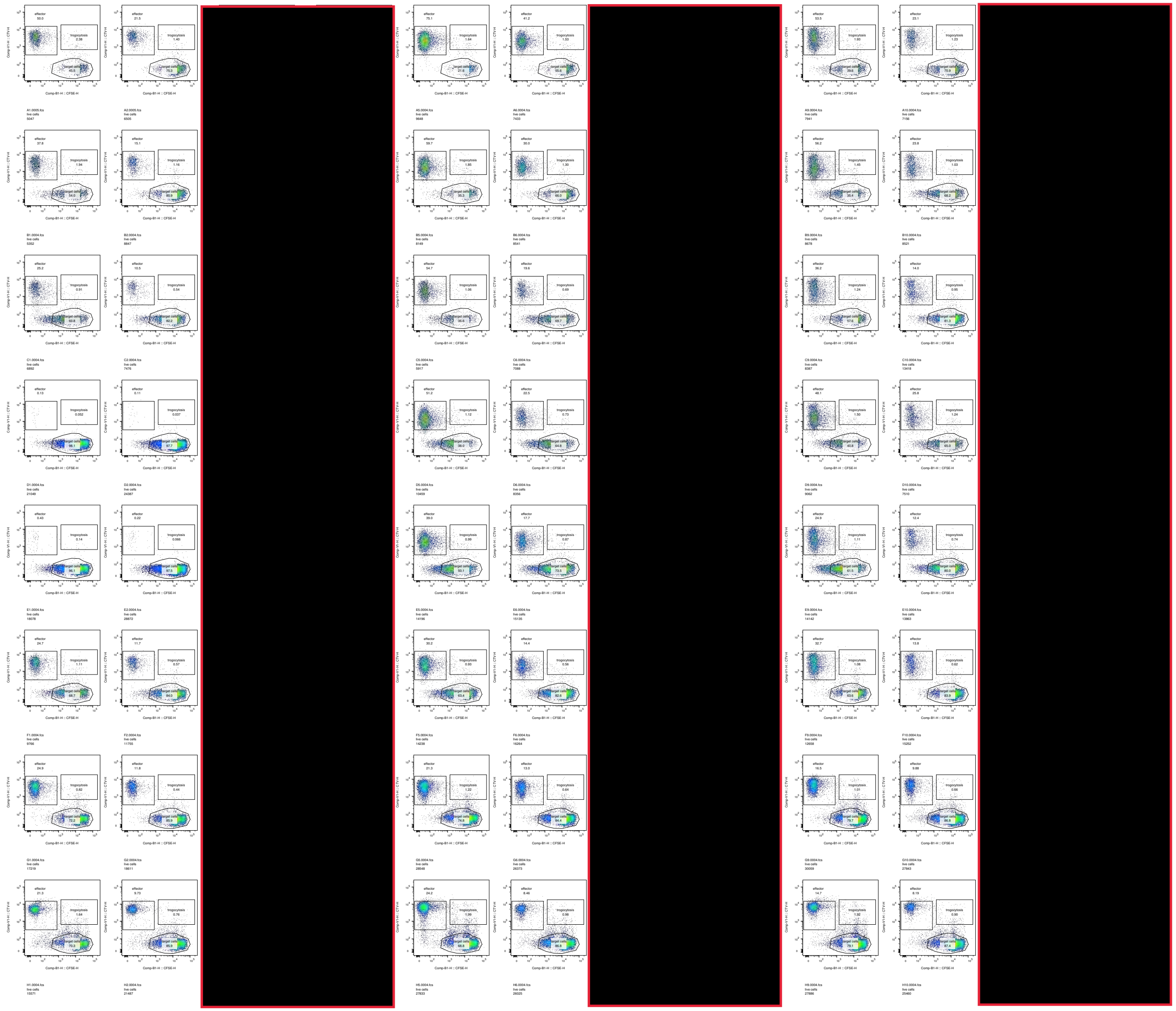

Plate14_JOVI_vs_HPB_TRBC2

Well	Target	E:T ratio	Donor	CAR construct
**1A**	HPB TRBC2+ve	1:4	Donor 7	JOVI_Hinge_41bbz
**1D**	HPB TRBC2+ve	1:4	Donor 7	JOVI_CD8STK_28z
**1E**	HPB TRBC2+ve	1:4	Donor 7	JOVI_CD28STK_CD28TM_28z
**1G**	HPB TRBC2+ve	1:4	Donor 7	aCD19-CAR
**1H**	HPB TRBC2+ve	1:4	Donor 7	Non-transduced
**2A**	HPB TRBC2+ve	1:8	Donor 7	JOVI_Hinge_41bbz
**2D**	HPB TRBC2+ve	1:8	Donor 7	JOVI_CD8STK_28z
**2E**	HPB TRBC2+ve	1:8	Donor 7	JOVI_CD28STK_CD28TM_28z
**2G**	HPB TRBC2+ve	1:8	Donor 7	aCD19-CAR
**2H**	HPB TRBC2+ve	1:8	Donor 7	Non-transduced
**5A**	HPB TRBC2+ve	1:4	Donor 8	JOVI_Hinge_41bbz
**5D**	HPB TRBC2+ve	1:4	Donor 8	JOVI_CD8STK_28z
**5E**	HPB TRBC2+ve	1:4	Donor 8	JOVI_CD28STK_CD28TM_28z
**5G**	HPB TRBC2+ve	1:4	Donor 8	aCD19-CAR
**5H**	HPB TRBC2+ve	1:4	Donor 8	Non-transduced
**6A**	HPB TRBC2+ve	1:8	Donor 8	JOVI_Hinge_41bbz
**6D**	HPB TRBC2+ve	1:8	Donor 8	JOVI_CD8STK_28z
**6E**	HPB TRBC2+ve	1:8	Donor 8	JOVI_CD28STK_CD28TM_28z
**6G**	HPB TRBC2+ve	1:8	Donor 8	aCD19-CAR
**6H**	HPB TRBC2+ve	1:8	Donor 8	Non-transduced
**9A**	HPB TRBC2+ve	1:4	Donor 9	JOVI_Hinge_41bbz
**9D**	HPB TRBC2+ve	1:4	Donor 9	JOVI_CD8STK_28z
**9E**	HPB TRBC2+ve	1:4	Donor 9	JOVI_CD28STK_CD28TM_28z
**9G**	HPB TRBC2+ve	1:4	Donor 9	aCD19-CAR
**9H**	HPB TRBC2+ve	1:4	Donor 9	Non-transduced
**10A**	HPB TRBC2+ve	1:8	Donor 9	JOVI_Hinge_41bbz
**10D**	HPB TRBC2+ve	1:8	Donor 9	JOVI_CD8STK_28z
**10E**	HPB TRBC2+ve	1:8	Donor 9	JOVI_CD28STK_CD28TM_28z
**10G**	HPB TRBC2+ve	1:8	Donor 9	aCD19-CAR
**10H**	HPB TRBC2+ve	1:8	Donor 9	Non-transduced

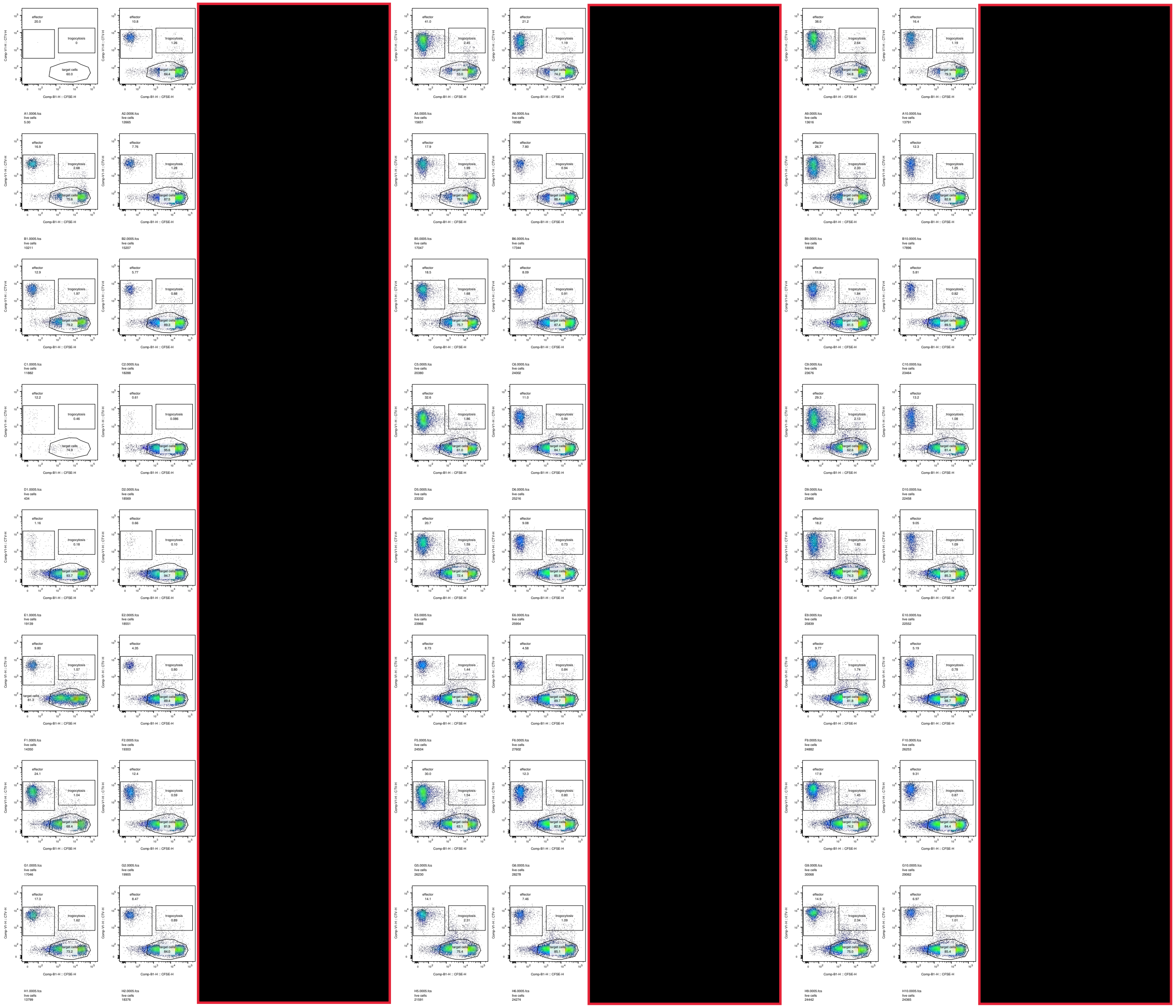

Plate15_JOVI_vs_HPB_TRBC-KO

Well	Target	E:T ratio	Donor	CAR construct
**1A**	HPB TRBC KO	1:4	Donor 7	JOVI_Hinge_41bbz
**1D**	HPB TRBC KO	1:4	Donor 7	JOVI_CD8STK_28z
**1E**	HPB TRBC KO	1:4	Donor 7	JOVI_CD28STK_CD28TM_28z
**1G**	HPB TRBC KO	1:4	Donor 7	aCD19-CAR
**1H**	HPB TRBC KO	1:4	Donor 7	Non-transduced
**2A**	HPB TRBC KO	1:8	Donor 7	JOVI_Hinge_41bbz
**2D**	HPB TRBC KO	1:8	Donor 7	JOVI_CD8STK_28z
**2E**	HPB TRBC KO	1:8	Donor 7	JOVI_CD28STK_CD28TM_28z
**2G**	HPB TRBC KO	1:8	Donor 7	aCD19-CAR
**2H**	HPB TRBC KO	1:8	Donor 7	Non-transduced
**5A**	HPB TRBC KO	1:4	Donor 8	JOVI_Hinge_41bbz
**5D**	HPB TRBC KO	1:4	Donor 8	JOVI_CD8STK_28z
**5E**	HPB TRBC KO	1:4	Donor 8	JOVI_CD28STK_CD28TM_28z
**5G**	HPB TRBC KO	1:4	Donor 8	aCD19-CAR
**5H**	HPB TRBC KO	1:4	Donor 8	Non-transduced
**6A**	HPB TRBC KO	1:8	Donor 8	JOVI_Hinge_41bbz
**6D**	HPB TRBC KO	1:8	Donor 8	JOVI_CD8STK_28z
**6E**	HPB TRBC KO	1:8	Donor 8	JOVI_CD28STK_CD28TM_28z
**6G**	HPB TRBC KO	1:8	Donor 8	aCD19-CAR
**6H**	HPB TRBC KO	1:8	Donor 8	Non-transduced
**9A**	HPB TRBC KO	1:4	Donor 9	JOVI_Hinge_41bbz
**9D**	HPB TRBC KO	1:4	Donor 9	JOVI_CD8STK_28z
**9E**	HPB TRBC KO	1:4	Donor 9	JOVI_CD28STK_CD28TM_28z
**9G**	HPB TRBC KO	1:4	Donor 9	aCD19-CAR
**9H**	HPB TRBC KO	1:4	Donor 9	Non-transduced
**10A**	HPB TRBC KO	1:8	Donor 9	JOVI_Hinge_41bbz
**10D**	HPB TRBC KO	1:8	Donor 9	JOVI_CD8STK_28z
**10E**	HPB TRBC KO	1:8	Donor 9	JOVI_CD28STK_CD28TM_28z
**10G**	HPB TRBC KO	1:8	Donor 9	aCD19-CAR
**10H**	HPB TRBC KO	1:8	Donor 9	Non-transduced

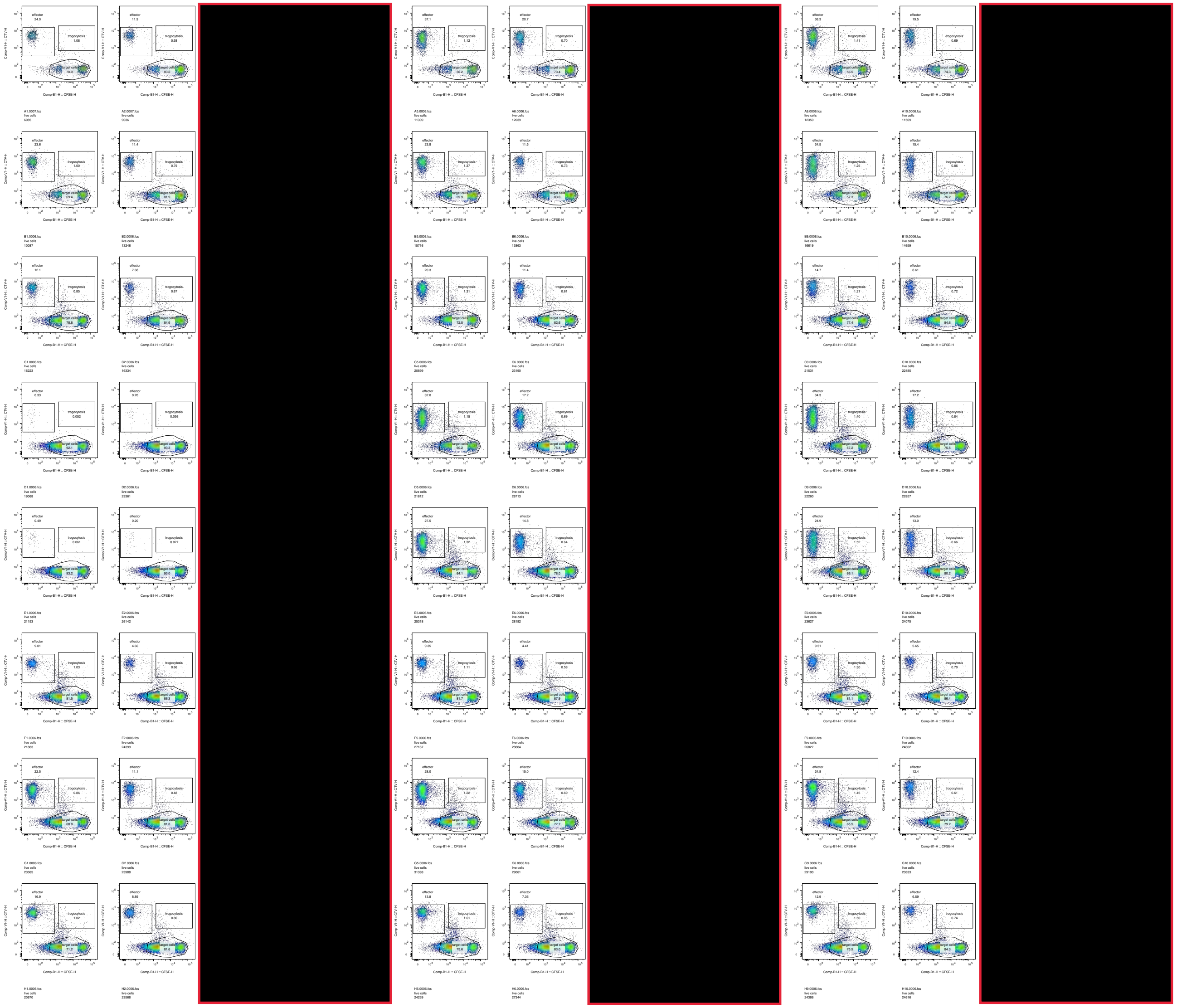

Plate16_JOVI_vs_H9

Well	Target	E:T ratio	Donor	CAR construct
**1A**	H9	1:4	Donor 7	JOVI_Hinge_41bbz
**1D**	H9	1:4	Donor 7	JOVI_CD8STK_28z
**1E**	H9	1:4	Donor 7	JOVI_CD28STK_CD28TM_28z
**1F**	H9	1:4	Donor 7	mJOVI_Hinge_41bbz
**1G**	H9	1:4	Donor 7	aCD19-CAR
**1H**	H9	1:4	Donor 7	Non-transduced
**2A**	H9	1:8	Donor 7	JOVI_Hinge_41bbz
**2D**	H9	1:8	Donor 7	JOVI_CD8STK_28z
**2E**	H9	1:8	Donor 7	JOVI_CD28STK_CD28TM_28z
**2F**	H9	1:8	Donor 7	mJOVI_Hinge_41bbz
**2G**	H9	1:8	Donor 7	aCD19-CAR
**2H**	H9	1:8	Donor 7	Non-transduced
**5A**	H9	1:4	Donor 8	JOVI_Hinge_41bbz
**5D**	H9	1:4	Donor 8	JOVI_CD8STK_28z
**5E**	H9	1:4	Donor 8	JOVI_CD28STK_CD28TM_28z
**5F**	H9	1:4	Donor 8	mJOVI_Hinge_41bbz
**5G**	H9	1:4	Donor 8	aCD19-CAR
**5H**	H9	1:4	Donor 8	Non-transduced
**6A**	H9	1:8	Donor 8	JOVI_Hinge_41bbz
**6D**	H9	1:8	Donor 8	JOVI_CD8STK_28z
**6E**	H9	1:8	Donor 8	JOVI_CD28STK_CD28TM_28z
**6F**	H9	1:8	Donor 8	mJOVI_Hinge_41bbz
**6G**	H9	1:8	Donor 8	aCD19-CAR
**6H**	H9	1:8	Donor 8	Non-transduced
**9A**	H9	1:4	Donor 9	JOVI_Hinge_41bbz
**9D**	H9	1:4	Donor 9	JOVI_CD8STK_28z
**9E**	H9	1:4	Donor 9	JOVI_CD28STK_CD28TM_28z
**9F**	H9	1:4	Donor 9	mJOVI_Hinge_41bbz
**9G**	H9	1:4	Donor 9	aCD19-CAR
**9H**	H9	1:4	Donor 9	Non-transduced
**10A**	H9	1:8	Donor 9	JOVI_Hinge_41bbz
**10D**	H9	1:8	Donor 9	JOVI_CD8STK_28z
**10E**	H9	1:8	Donor 9	JOVI_CD28STK_CD28TM_28z
**10F**	H9	1:8	Donor 9	mJOVI_Hinge_41bbz
**10G**	H9	1:8	Donor 9	aCD19-CAR
**10H**	H9	1:8	Donor 9	Non-transduced

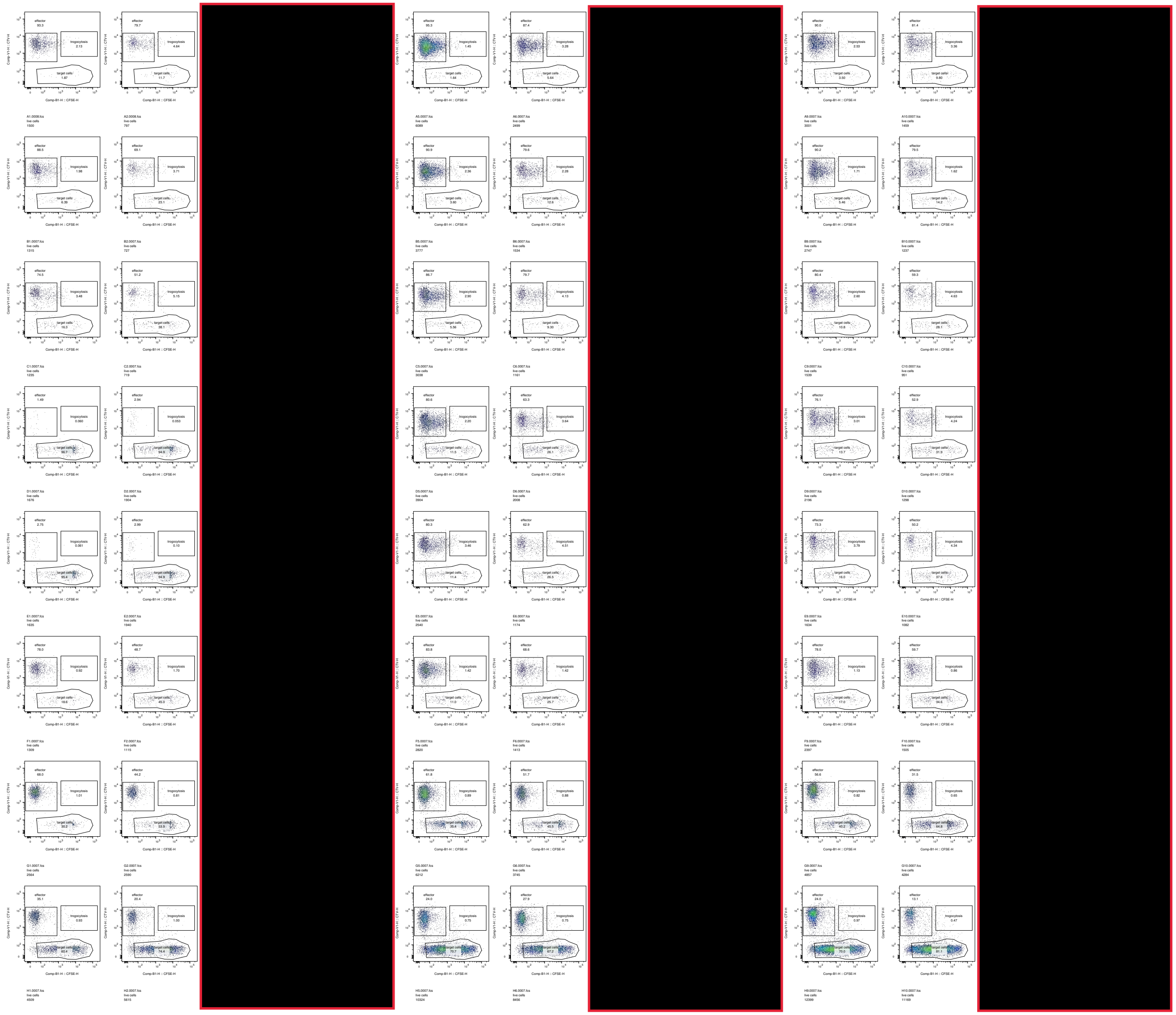

Plate17_JOVI_vs_TALL1

Well	Target	E:T ratio	Donor	CAR construct
**1A**	T-ALL1	1:4	Donor 7	JOVI_Hinge_41bbz
**1D**	T-ALL1	1:4	Donor 7	JOVI_CD8STK_28z
**1E**	T-ALL1	1:4	Donor 7	JOVI_CD28STK_CD28TM_28z
**1G**	T-ALL1	1:4	Donor 7	aCD19-CAR
**1H**	T-ALL1	1:4	Donor 7	Non-transduced
**2A**	T-ALL1	1:8	Donor 7	JOVI_Hinge_41bbz
**2D**	T-ALL1	1:8	Donor 7	JOVI_CD8STK_28z
**2E**	T-ALL1	1:8	Donor 7	JOVI_CD28STK_CD28TM_28z
**2G**	T-ALL1	1:8	Donor 7	aCD19-CAR
**2H**	T-ALL1	1:8	Donor 7	Non-transduced
**5A**	T-ALL1	1:4	Donor 8	JOVI_Hinge_41bbz
**5D**	T-ALL1	1:4	Donor 8	JOVI_CD8STK_28z
**5E**	T-ALL1	1:4	Donor 8	JOVI_CD28STK_CD28TM_28z
**5G**	T-ALL1	1:4	Donor 8	aCD19-CAR
**5H**	T-ALL1	1:4	Donor 8	Non-transduced
**6A**	T-ALL1	1:8	Donor 8	JOVI_Hinge_41bbz
**6D**	T-ALL1	1:8	Donor 8	JOVI_CD8STK_28z
**6E**	T-ALL1	1:8	Donor 8	JOVI_CD28STK_CD28TM_28z
**6G**	T-ALL1	1:8	Donor 8	aCD19-CAR
**6H**	T-ALL1	1:8	Donor 8	Non-transduced
**9A**	T-ALL1	1:4	Donor 9	JOVI_Hinge_41bbz
**9D**	T-ALL1	1:4	Donor 9	JOVI_CD8STK_28z
**9E**	T-ALL1	1:4	Donor 9	JOVI_CD28STK_CD28TM_28z
**9G**	T-ALL1	1:4	Donor 9	aCD19-CAR
**9H**	T-ALL1	1:4	Donor 9	Non-transduced
**10A**	T-ALL1	1:8	Donor 9	JOVI_Hinge_41bbz
**10D**	T-ALL1	1:8	Donor 9	JOVI_CD8STK_28z
**10E**	T-ALL1	1:8	Donor 9	JOVI_CD28STK_CD28TM_28z
**10G**	T-ALL1	1:8	Donor 9	aCD19-CAR
**10H**	T-ALL1	1:8	Donor 9	Non-transduced

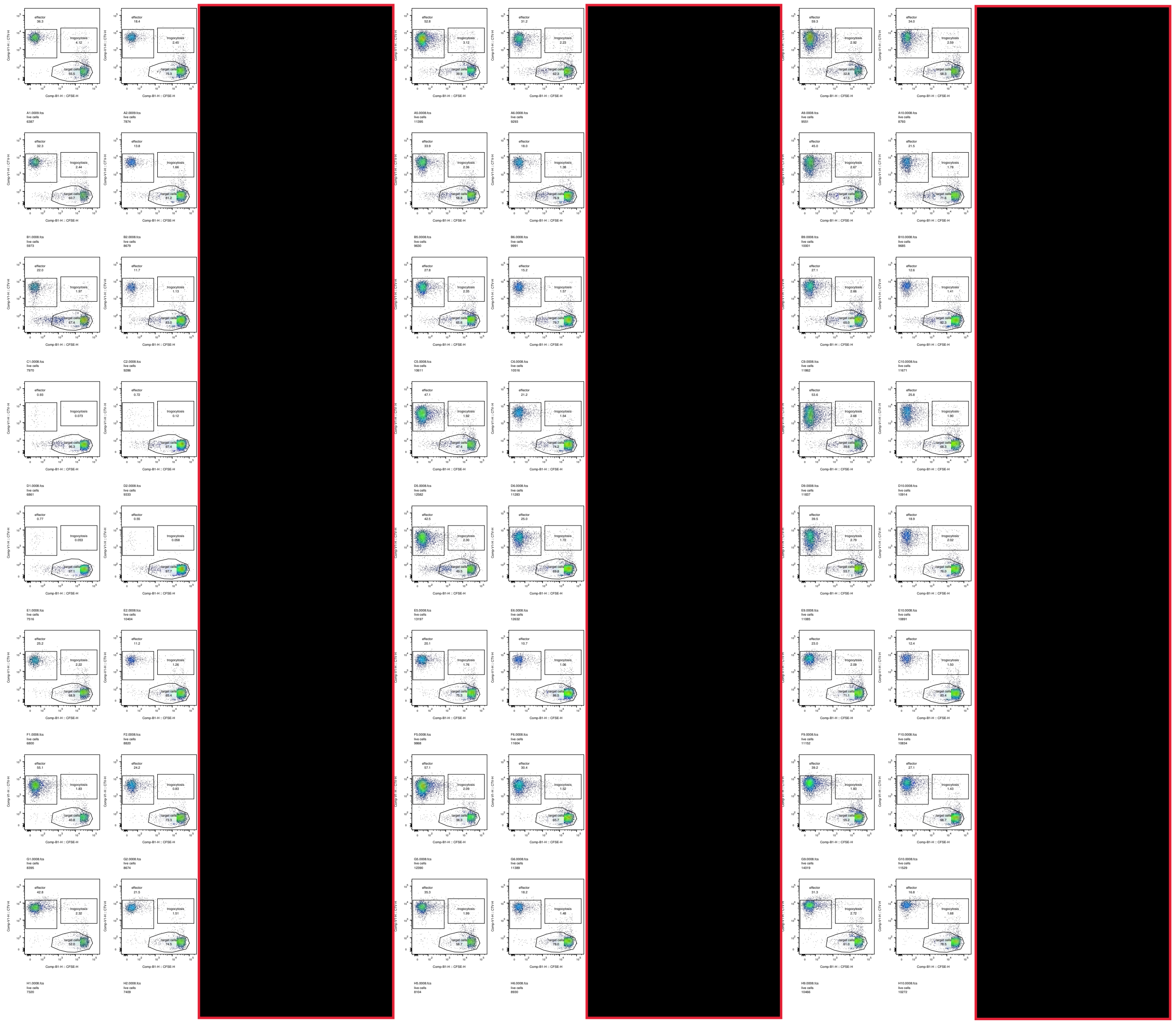

effector
36.3
trogocytosis
4.12
target cells
55.5
Comp-V1-H :: CTV-H
Comp-B1-H :: CFSE-H
A1.0009.fcs
live cells
6387
effector
18.4
trogocytosis
2.45
target cells
75.3
A2.0009.fcs
live cells
7874
effector
32.3
trogocytosis
2.44
effector
13.8
trogocytosis
1.66

Plate1_JOVI_vs_Jurkat_TRBC1

**Well**	**Target**	**E:T ratio**	**Donor**	**CAR construct**
**1A**	Jurkat TRBC 1+ve	1:4	Donor 14	JOVI_Hinge_41bbz
**1D**	Jurkat TRBC 1+ve	1:4	Donor 14	JOVI_CD8STK_28z
**1E**	Jurkat TRBC 1+ve	1:4	Donor 14	JOVI_CD28STK_CD28TM_28z
**1F**	Jurkat TRBC 1+ve	1:4	Donor 14	mJOVI_Hinge_41bbz
**1G**	Jurkat TRBC 1+ve	1:4	Donor 14	aCD19-CAR
**1H**	Jurkat TRBC 1+ve	1:4	Donor 14	Non-transduced
**2A**	Jurkat TRBC 1+ve	1:8	Donor 14	JOVI_Hinge_41bbz
**2D**	Jurkat TRBC 1+ve	1:8	Donor 14	JOVI_CD8STK_28z
**2E**	Jurkat TRBC 1+ve	1:8	Donor 14	JOVI_CD28STK_CD28TM_28z
**2F**	Jurkat TRBC 1+ve	1:8	Donor 14	mJOVI_Hinge_41bbz
**2G**	Jurkat TRBC 1+ve	1:8	Donor 14	aCD19-CAR
**2H**	Jurkat TRBC 1+ve	1:8	Donor 14	Non-transduced
**5A**	Jurkat TRBC 1+ve	1:4	Donor 15	JOVI_Hinge_41bbz
**5D**	Jurkat TRBC 1+ve	1:4	Donor 15	JOVI_CD8STK_28z
**5E**	Jurkat TRBC 1+ve	1:4	Donor 15	JOVI_CD28STK_CD28TM_28z
**5F**	Jurkat TRBC 1+ve	1:4	Donor 15	mJOVI_Hinge_41bbz
**5G**	Jurkat TRBC 1+ve	1:4	Donor 15	aCD19-CAR
**5H**	Jurkat TRBC 1+ve	1:4	Donor 15	Non-transduced
**6A**	Jurkat TRBC 1+ve	1:8	Donor 15	JOVI_Hinge_41bbz
**6D**	Jurkat TRBC 1+ve	1:8	Donor 15	JOVI_CD8STK_28z
**6E**	Jurkat TRBC 1+ve	1:8	Donor 15	JOVI_CD28STK_CD28TM_28z
**6F**	Jurkat TRBC 1+ve	1:8	Donor 15	mJOVI_Hinge_41bbz
**6G**	Jurkat TRBC 1+ve	1:8	Donor 15	aCD19-CAR
**6H**	Jurkat TRBC 1+ve	1:8	Donor 15	Non-transduced
**9A**	Jurkat TRBC 1+ve	1:4	Donor 16	JOVI_Hinge_41bbz
**9D**	Jurkat TRBC 1+ve	1:4	Donor 16	JOVI_CD8STK_28z
**9E**	Jurkat TRBC 1+ve	1:4	Donor 16	JOVI_CD28STK_CD28TM_28z
**9F**	Jurkat TRBC 1+ve	1:4	Donor 16	mJOVI_Hinge_41bbz
**9G**	Jurkat TRBC 1+ve	1:4	Donor 16	aCD19-CAR
**9H**	Jurkat TRBC 1+ve	1:4	Donor 16	Non-transduced
**10A**	Jurkat TRBC 1+ve	1:8	Donor 16	JOVI_Hinge_41bbz
**10D**	Jurkat TRBC 1+ve	1:8	Donor 16	JOVI_CD8STK_28z
**10E**	Jurkat TRBC 1+ve	1:8	Donor 16	JOVI_CD28STK_CD28TM_28z
**10F**	Jurkat TRBC 1+ve	1:8	Donor 16	mJOVI_Hinge_41bbz
**10G**	Jurkat TRBC 1+ve	1:8	Donor 16	aCD19-CAR
**10H**	Jurkat TRBC 1+ve	1:8	Donor 16	Non-transduced

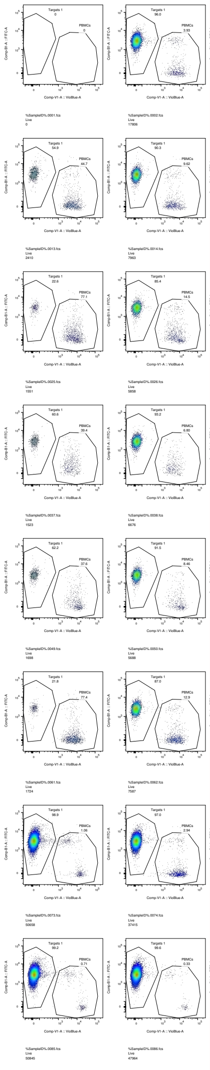

[redacted]

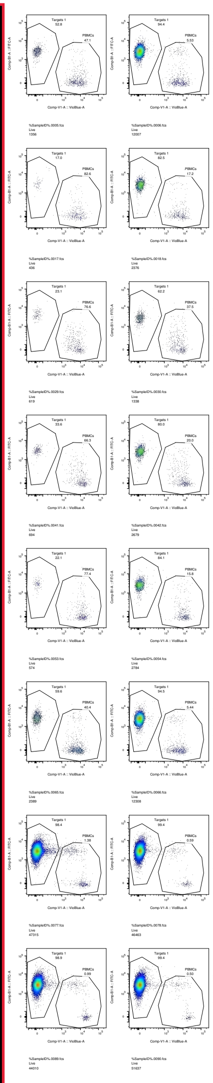

[redacted]

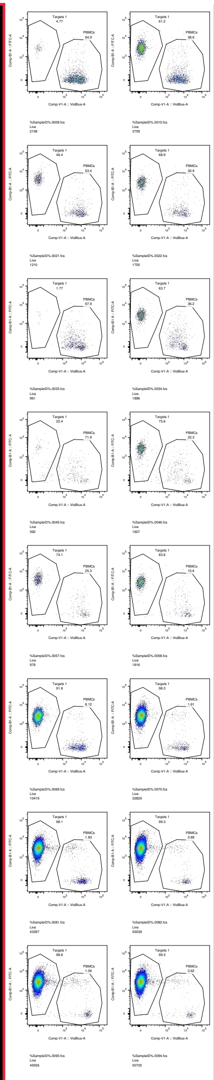

[redacted]

Plate2_JOVI_vs_TRBC2

Well	Target	E:T ratio	Donor	CAR construct
**1A**	Jurkat TRBC 2+ve	1:4	Donor 14	JOVI_Hinge_41bbz
**1D**	Jurkat TRBC 2+ve	1:4	Donor 14	JOVI_CD8STK_28z
**1E**	Jurkat TRBC 2+ve	1:4	Donor 14	JOVI_CD28STK_CD28TM_28z
**1F**	Jurkat TRBC 2+ve	1:4	Donor 14	mJOVI_Hinge_41bbz
**1G**	Jurkat TRBC 2+ve	1:4	Donor 14	aCD19-CAR
**1H**	Jurkat TRBC 2+ve	1:4	Donor 14	Non-transduced
**2A**	Jurkat TRBC 2+ve	1:8	Donor 14	JOVI_Hinge_41bbz
**2D**	Jurkat TRBC 2+ve	1:8	Donor 14	JOVI_CD8STK_28z
**2E**	Jurkat TRBC 2+ve	1:8	Donor 14	JOVI_CD28STK_CD28TM_28z
**2F**	Jurkat TRBC 2+ve	1:8	Donor 14	mJOVI_Hinge_41bbz
**2G**	Jurkat TRBC 2+ve	1:8	Donor 14	aCD19-CAR
**2H**	Jurkat TRBC 2+ve	1:8	Donor 14	Non-transduced
**5A**	Jurkat TRBC 2+ve	1:4	Donor 15	JOVI_Hinge_41bbz
**5D**	Jurkat TRBC 2+ve	1:4	Donor 15	JOVI_CD8STK_28z
**5E**	Jurkat TRBC 2+ve	1:4	Donor 15	JOVI_CD28STK_CD28TM_28z
**5F**	Jurkat TRBC 2+ve	1:4	Donor 15	mJOVI_Hinge_41bbz
**5G**	Jurkat TRBC 2+ve	1:4	Donor 15	aCD19-CAR
**5H**	Jurkat TRBC 2+ve	1:4	Donor 15	Non-transduced
**6A**	Jurkat TRBC 2+ve	1:8	Donor 15	JOVI_Hinge_41bbz
**6D**	Jurkat TRBC 2+ve	1:8	Donor 15	JOVI_CD8STK_28z
**6E**	Jurkat TRBC 2+ve	1:8	Donor 15	JOVI_CD28STK_CD28TM_28z
**6F**	Jurkat TRBC 2+ve	1:8	Donor 15	mJOVI_Hinge_41bbz
**6G**	Jurkat TRBC 2+ve	1:8	Donor 15	aCD19-CAR
**6H**	Jurkat TRBC 2+ve	1:8	Donor 15	Non-transduced
**9A**	Jurkat TRBC 2+ve	1:4	Donor 16	JOVI_Hinge_41bbz
**9D**	Jurkat TRBC 2+ve	1:4	Donor 16	JOVI_CD8STK_28z
**9E**	Jurkat TRBC 2+ve	1:4	Donor 16	JOVI_CD28STK_CD28TM_28z
**9F**	Jurkat TRBC 2+ve	1:4	Donor 16	mJOVI_Hinge_41bbz
**9G**	Jurkat TRBC 2+ve	1:4	Donor 16	aCD19-CAR
**9H**	Jurkat TRBC 2+ve	1:4	Donor 16	Non-transduced
**10A**	Jurkat TRBC 2+ve	1:8	Donor 16	JOVI_Hinge_41bbz
**10D**	Jurkat TRBC 2+ve	1:8	Donor 16	JOVI_CD8STK_28z
**10E**	Jurkat TRBC 2+ve	1:8	Donor 16	JOVI_CD28STK_CD28TM_28z
**10F**	Jurkat TRBC 2+ve	1:8	Donor 16	mJOVI_Hinge_41bbz
**10G**	Jurkat TRBC 2+ve	1:8	Donor 16	aCD19-CAR
**10H**	Jurkat TRBC 2+ve	1:8	Donor 16	Non-transduced

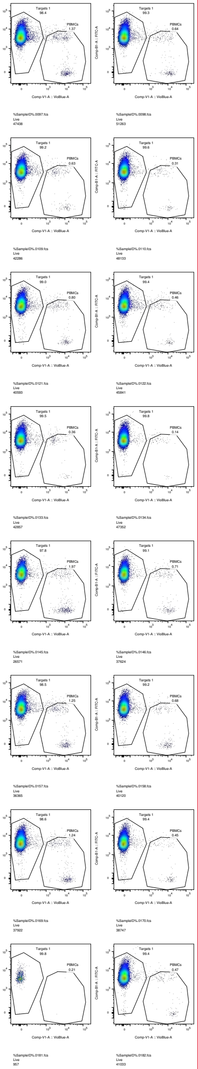

[redacted]

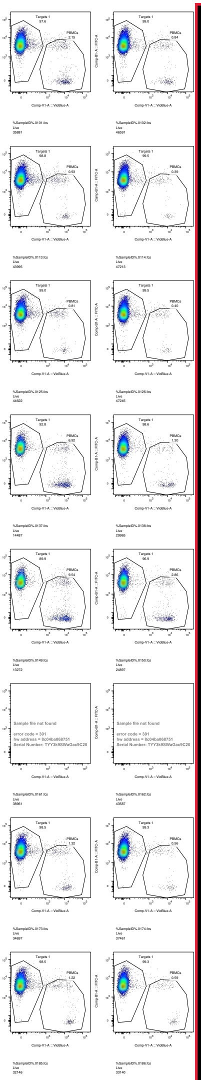

[redacted]

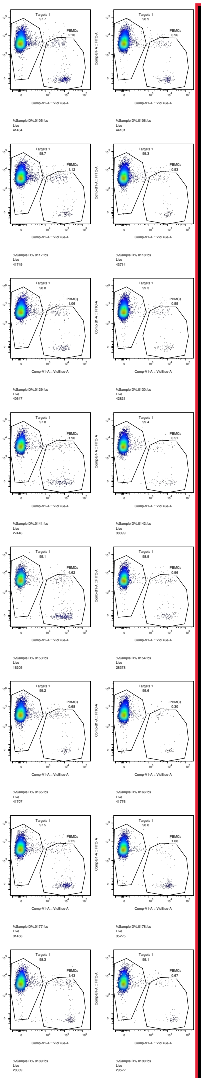

[redacted]

Plate3_JOVI_vs_Jurkat_TRBC-KO

Well	Target	E:T ratio	Donor	CAR construct
**1A**	Jurkat TRBC KO	1:4	Donor 14	JOVI_Hinge_41bbz
**1D**	Jurkat TRBC KO	1:4	Donor 14	JOVI_CD8STK_28z
**1E**	Jurkat TRBC KO	1:4	Donor 14	JOVI_CD28STK_CD28TM_28z
**1F**	Jurkat TRBC KO	1:4	Donor 14	mJOVI_Hinge_41bbz
**1G**	Jurkat TRBC KO	1:4	Donor 14	aCD19-CAR
**1H**	Jurkat TRBC KO	1:4	Donor 14	Non-transduced
**2A**	Jurkat TRBC KO	1:8	Donor 14	JOVI_Hinge_41bbz
**2D**	Jurkat TRBC KO	1:8	Donor 14	JOVI_CD8STK_28z
**2E**	Jurkat TRBC KO	1:8	Donor 14	JOVI_CD28STK_CD28TM_28z
**2F**	Jurkat TRBC KO	1:8	Donor 14	mJOVI_Hinge_41bbz
**2G**	Jurkat TRBC KO	1:8	Donor 14	aCD19-CAR
**2H**	Jurkat TRBC KO	1:8	Donor 14	Non-transduced
**5A**	Jurkat TRBC KO	1:4	Donor 15	JOVI_Hinge_41bbz
**5D**	Jurkat TRBC KO	1:4	Donor 15	JOVI_CD8STK_28z
**5E**	Jurkat TRBC KO	1:4	Donor 15	JOVI_CD28STK_CD28TM_28z
**5F**	Jurkat TRBC KO	1:4	Donor 15	mJOVI_Hinge_41bbz
**5G**	Jurkat TRBC KO	1:4	Donor 15	aCD19-CAR
**5H**	Jurkat TRBC KO	1:4	Donor 15	Non-transduced
**6A**	Jurkat TRBC KO	1:8	Donor 15	JOVI_Hinge_41bbz
**6D**	Jurkat TRBC KO	1:8	Donor 15	JOVI_CD8STK_28z
**6E**	Jurkat TRBC KO	1:8	Donor 15	JOVI_CD28STK_CD28TM_28z
**6F**	Jurkat TRBC KO	1:8	Donor 15	mJOVI_Hinge_41bbz
**6G**	Jurkat TRBC KO	1:8	Donor 15	aCD19-CAR
**6H**	Jurkat TRBC KO	1:8	Donor 15	Non-transduced
**9A**	Jurkat TRBC KO	1:4	Donor 16	JOVI_Hinge_41bbz
**9D**	Jurkat TRBC KO	1:4	Donor 16	JOVI_CD8STK_28z
**9E**	Jurkat TRBC KO	1:4	Donor 16	JOVI_CD28STK_CD28TM_28z
**9F**	Jurkat TRBC KO	1:4	Donor 16	mJOVI_Hinge_41bbz
**9G**	Jurkat TRBC KO	1:4	Donor 16	aCD19-CAR
**9H**	Jurkat TRBC KO	1:4	Donor 16	Non-transduced
**10A**	Jurkat TRBC KO	1:8	Donor 16	JOVI_Hinge_41bbz
**10D**	Jurkat TRBC KO	1:8	Donor 16	JOVI_CD8STK_28z
**10E**	Jurkat TRBC KO	1:8	Donor 16	JOVI_CD28STK_CD28TM_28z
**10F**	Jurkat TRBC KO	1:8	Donor 16	mJOVI_Hinge_41bbz
**10G**	Jurkat TRBC KO	1:8	Donor 16	aCD19-CAR
**10H**	Jurkat TRBC KO	1:8	Donor 16	Non-transduced

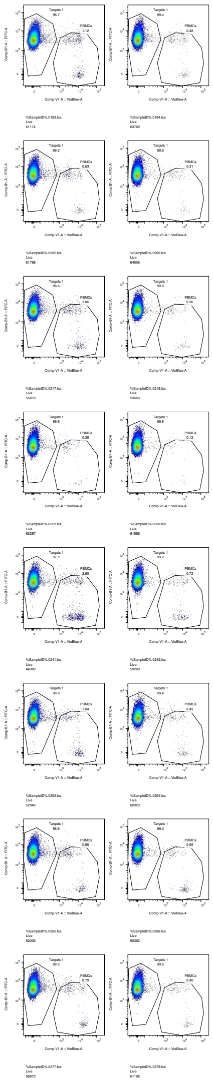

[redacted]

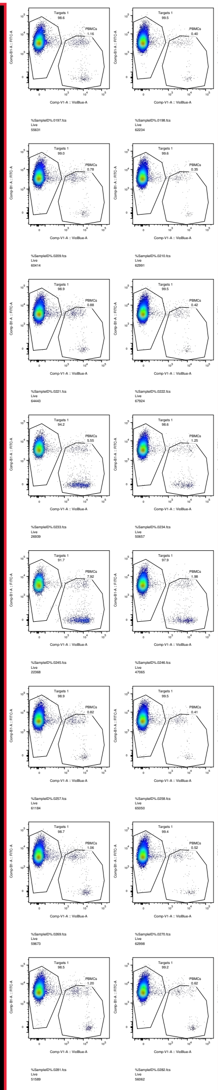

[redacted]

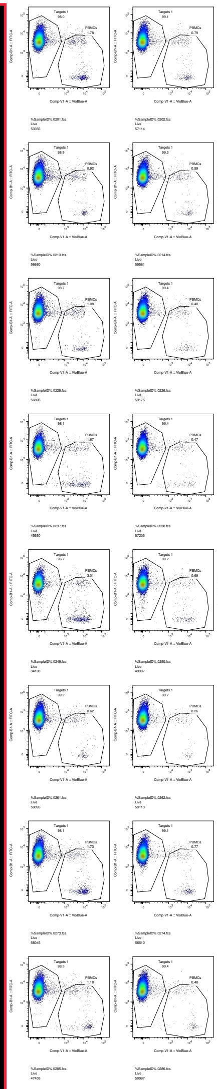

[redacted]

Plate4_JOVI_vs_HPB_TRBC1

Well	Target	E:T ratio	Donor	CAR construct
1A	HPB TRBC1+ve	1:4	Donor 14	JOVI_Hinge_41bbz
1D	HPB TRBC1+ve	1:4	Donor 14	JOVI_CD8STK_28z
1E	HPB TRBC1+ve	1:4	Donor 14	JOVI_CD28STK_CD28TM_28z
1G	HPB TRBC1+ve	1:4	Donor 14	aCD19-CAR
1H	HPB TRBC1+ve	1:4	Donor 14	Non-transduced
2A	HPB TRBC1+ve	1:8	Donor 14	JOVI_Hinge_41bbz
2D	HPB TRBC1+ve	1:8	Donor 14	JOVI_CD8STK_28z
2E	HPB TRBC1+ve	1:8	Donor 14	JOVI_CD28STK_CD28TM_28z
2G	HPB TRBC1+ve	1:8	Donor 14	aCD19-CAR
2H	HPB TRBC1+ve	1:8	Donor 14	Non-transduced
5A	HPB TRBC1+ve	1:4	Donor 15	JOVI_Hinge_41bbz
5D	HPB TRBC1+ve	1:4	Donor 15	JOVI_CD8STK_28z
5E	HPB TRBC1+ve	1:4	Donor 15	JOVI_CD28STK_CD28TM_28z
5G	HPB TRBC1+ve	1:4	Donor 15	aCD19-CAR
5H	HPB TRBC1+ve	1:4	Donor 15	Non-transduced
6A	HPB TRBC1+ve	1:8	Donor 15	JOVI_Hinge_41bbz
6D	HPB TRBC1+ve	1:8	Donor 15	JOVI_CD8STK_28z
6E	HPB TRBC1+ve	1:8	Donor 15	JOVI_CD28STK_CD28TM_28z
6G	HPB TRBC1+ve	1:8	Donor 15	aCD19-CAR
6H	HPB TRBC1+ve	1:8	Donor 15	Non-transduced
9A	HPB TRBC1+ve	1:4	Donor 16	JOVI_Hinge_41bbz
9D	HPB TRBC1+ve	1:4	Donor 16	JOVI_CD8STK_28z
9E	HPB TRBC1+ve	1:4	Donor 16	JOVI_CD28STK_CD28TM_28z
9G	HPB TRBC1+ve	1:4	Donor 16	aCD19-CAR
9H	HPB TRBC1+ve	1:4	Donor 16	Non-transduced
10A	HPB TRBC1+ve	1:8	Donor 16	JOVI_Hinge_41bbz
10D	HPB TRBC1+ve	1:8	Donor 16	JOVI_CD8STK_28z
10E	HPB TRBC1+ve	1:8	Donor 16	JOVI_CD28STK_CD28TM_28z
10G	HPB TRBC1+ve	1:8	Donor 16	aCD19-CAR
10H	HPB TRBC1+ve	1:8	Donor 16	Non-transduced

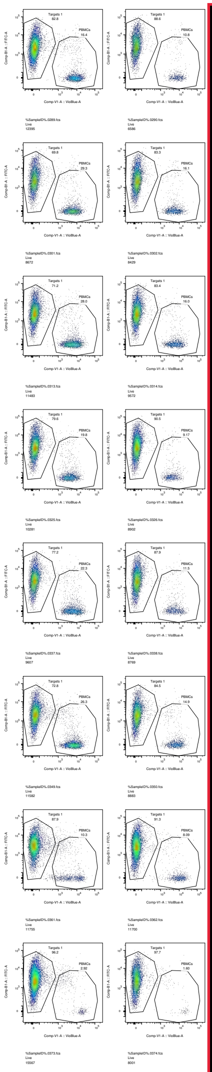

[redacted]

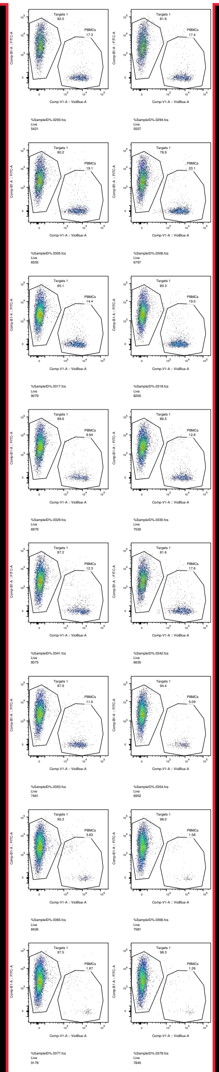

[redacted]

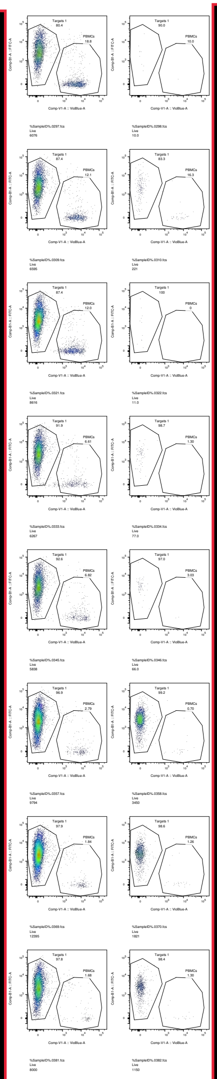

[redacted]

Plate5_JOVI_vs_HPB_TRBC2

Well	Target	E:T ratio	Donor	CAR construct
1A	HPB TRBC2+ve	1:4	Donor 14	JOVI_Hinge_41bbz
1D	HPB TRBC2+ve	1:4	Donor 14	JOVI_CD8STK_28z
1E	HPB TRBC2+ve	1:4	Donor 14	JOVI_CD28STK_CD28TM_28z
1G	HPB TRBC2+ve	1:4	Donor 14	aCD19-CAR
1H	HPB TRBC2+ve	1:4	Donor 14	Non-transduced
2A	HPB TRBC2+ve	1:8	Donor 14	JOVI_Hinge_41bbz
2D	HPB TRBC2+ve	1:8	Donor 14	JOVI_CD8STK_28z
2E	HPB TRBC2+ve	1:8	Donor 14	JOVI_CD28STK_CD28TM_28z
2G	HPB TRBC2+ve	1:8	Donor 14	aCD19-CAR
2H	HPB TRBC2+ve	1:8	Donor 14	Non-transduced
5A	HPB TRBC2+ve	1:4	Donor 15	JOVI_Hinge_41bbz
5D	HPB TRBC2+ve	1:4	Donor 15	JOVI_CD8STK_28z
5E	HPB TRBC2+ve	1:4	Donor 15	JOVI_CD28STK_CD28TM_28z
5G	HPB TRBC2+ve	1:4	Donor 15	aCD19-CAR
5H	HPB TRBC2+ve	1:4	Donor 15	Non-transduced
6A	HPB TRBC2+ve	1:8	Donor 15	JOVI_Hinge_41bbz
6D	HPB TRBC2+ve	1:8	Donor 15	JOVI_CD8STK_28z
6E	HPB TRBC2+ve	1:8	Donor 15	JOVI_CD28STK_CD28TM_28z
6G	HPB TRBC2+ve	1:8	Donor 15	aCD19-CAR
6H	HPB TRBC2+ve	1:8	Donor 15	Non-transduced
9A	HPB TRBC2+ve	1:4	Donor 16	JOVI_Hinge_41bbz
9D	HPB TRBC2+ve	1:4	Donor 16	JOVI_CD8STK_28z
9E	HPB TRBC2+ve	1:4	Donor 16	JOVI_CD28STK_CD28TM_28z
9G	HPB TRBC2+ve	1:4	Donor 16	aCD19-CAR
9H	HPB TRBC2+ve	1:4	Donor 16	Non-transduced
10A	HPB TRBC2+ve	1:8	Donor 16	JOVI_Hinge_41bbz
10D	HPB TRBC2+ve	1:8	Donor 16	JOVI_CD8STK_28z
10E	HPB TRBC2+ve	1:8	Donor 16	JOVI_CD28STK_CD28TM_28z
10G	HPB TRBC2+ve	1:8	Donor 16	aCD19-CAR
10H	HPB TRBC2+ve	1:8	Donor 16	Non-transduced

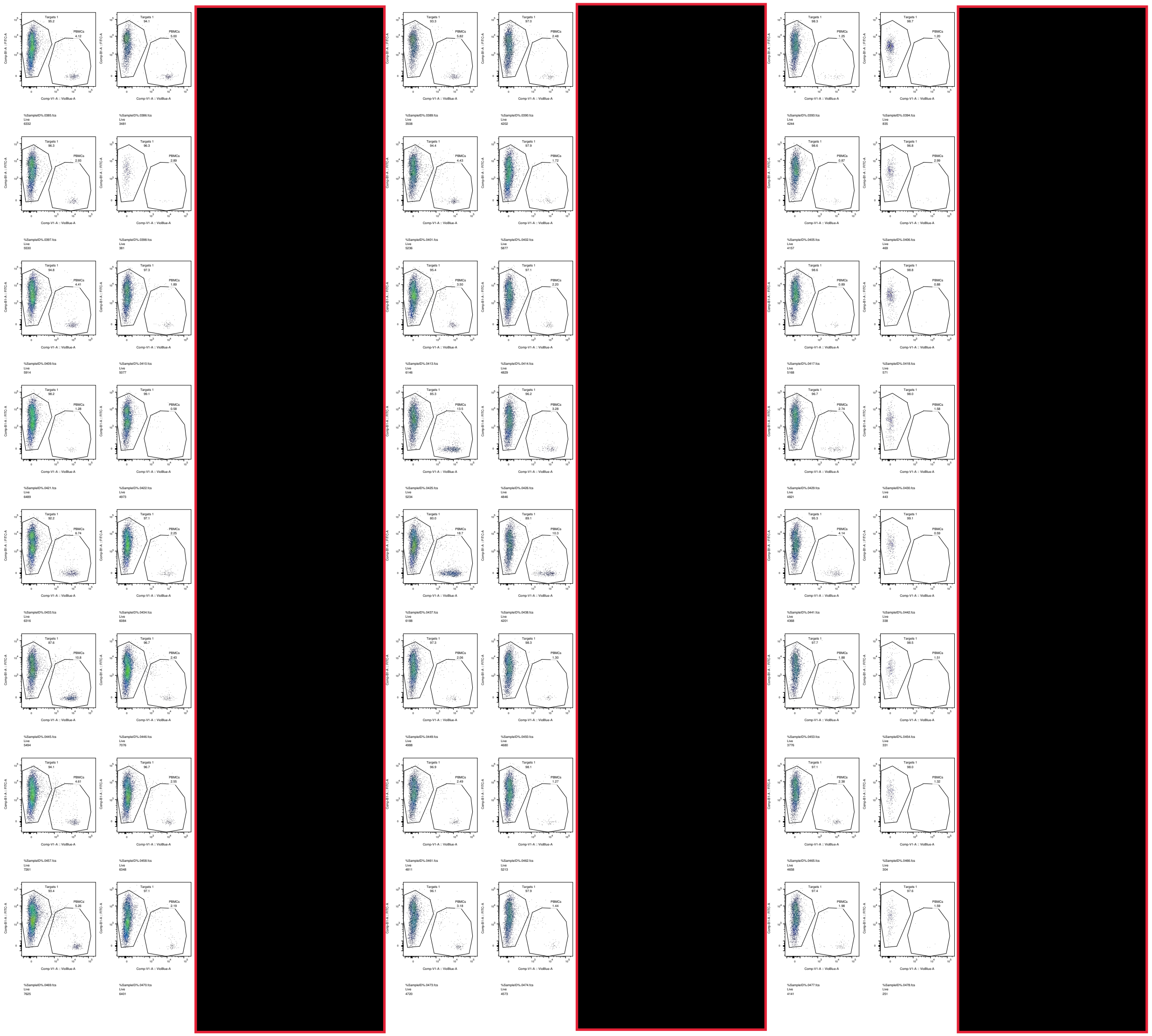

[redacted]

[redacted]

[redacted]

Plate6_JOVI_vs_HPB_TRBC-KO

Well	Target	E:T ratio	Donor	CAR construct
**1A**	HPB TRBC KO	1:4	Donor 14	JOVI_Hinge_41bbz
**1D**	HPB TRBC KO	1:4	Donor 14	JOVI_CD8STK_28z
**1E**	HPB TRBC KO	1:4	Donor 14	JOVI_CD28STK_CD28TM_28z
**1G**	HPB TRBC KO	1:4	Donor 14	aCD19-CAR
**1H**	HPB TRBC KO	1:4	Donor 14	Non-transduced
**2A**	HPB TRBC KO	1:8	Donor 14	JOVI_Hinge_41bbz
**2D**	HPB TRBC KO	1:8	Donor 14	JOVI_CD8STK_28z
**2E**	HPB TRBC KO	1:8	Donor 14	JOVI_CD28STK_CD28TM_28z
**2G**	HPB TRBC KO	1:8	Donor 14	aCD19-CAR
**2H**	HPB TRBC KO	1:8	Donor 14	Non-transduced
**5A**	HPB TRBC KO	1:4	Donor 15	JOVI_Hinge_41bbz
**5D**	HPB TRBC KO	1:4	Donor 15	JOVI_CD8STK_28z
**5E**	HPB TRBC KO	1:4	Donor 15	JOVI_CD28STK_CD28TM_28z
**5G**	HPB TRBC KO	1:4	Donor 15	aCD19-CAR
**5H**	HPB TRBC KO	1:4	Donor 15	Non-transduced
**6A**	HPB TRBC KO	1:8	Donor 15	JOVI_Hinge_41bbz
**6D**	HPB TRBC KO	1:8	Donor 15	JOVI_CD8STK_28z
**6E**	HPB TRBC KO	1:8	Donor 15	JOVI_CD28STK_CD28TM_28z
**6G**	HPB TRBC KO	1:8	Donor 15	aCD19-CAR
**6H**	HPB TRBC KO	1:8	Donor 15	Non-transduced
**9A**	HPB TRBC KO	1:4	Donor 16	JOVI_Hinge_41bbz
**9D**	HPB TRBC KO	1:4	Donor 16	JOVI_CD8STK_28z
**9E**	HPB TRBC KO	1:4	Donor 16	JOVI_CD28STK_CD28TM_28z
**9G**	HPB TRBC KO	1:4	Donor 16	aCD19-CAR
**9H**	HPB TRBC KO	1:4	Donor 16	Non-transduced
**10A**	HPB TRBC KO	1:8	Donor 16	JOVI_Hinge_41bbz
**10D**	HPB TRBC KO	1:8	Donor 16	JOVI_CD8STK_28z
**10E**	HPB TRBC KO	1:8	Donor 16	JOVI_CD28STK_CD28TM_28z
**10G**	HPB TRBC KO	1:8	Donor 16	aCD19-CAR
**10H**	HPB TRBC KO	1:8	Donor 16	Non-transduced

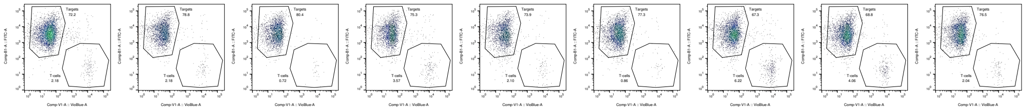

%SampleID%.0481.fcs FSC-A, APC-Vio770-A subset 6342
%SampleID%.0482.fcs FSC-A, APC-Vio770-A subset 3485
%SampleID%.0483.fcs FSC-A, APC-Vio770-A subset 3757
%SampleID%.0484.fcs FSC-A, APC-Vio770-A subset 3983
%SampleID%.0485.fcs FSC-A, APC-Vio770-A subset 3619
%SampleID%.0486.fcs FSC-A, APC-Vio770-A subset 4549
%SampleID%.0487.fcs FSC-A, APC-Vio770-A subset 5051
%SampleID%.0488.fcs FSC-A, APC-Vio770-A subset 5192
%SampleID%.0489.fcs FSC-A, APC-Vio770-A subset 4558

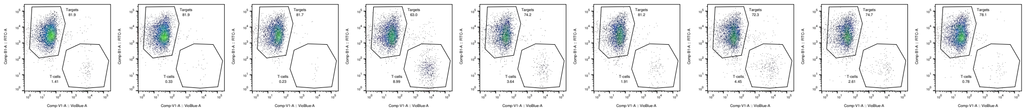

%SampleID%.0508.fcs FSC-A, APC-Vio770-A subset 6669
%SampleID%.0509.fcs FSC-A, APC-Vio770-A subset 6867
%SampleID%.0510.fcs FSC-A, APC-Vio770-A subset 5600
%SampleID%.0511.fcs FSC-A, APC-Vio770-A subset 6838
%SampleID%.0512.fcs FSC-A, APC-Vio770-A subset 5033
%SampleID%.0513.fcs FSC-A, APC-Vio770-A subset 5185
%SampleID%.0514.fcs FSC-A, APC-Vio770-A subset 5817
%SampleID%.0515.fcs FSC-A, APC-Vio770-A subset 5057
%SampleID%.0516.fcs FSC-A, APC-Vio770-A subset 6513

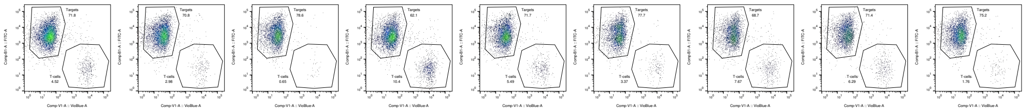

%SampleID%.0517.fcs FSC-A, APC-Vio770-A subset 8390
%SampleID%.0518.fcs FSC-A, APC-Vio770-A subset 7208
%SampleID%.0519.fcs FSC-A, APC-Vio770-A subset 5402
%SampleID%.0520.fcs FSC-A, APC-Vio770-A subset 7774
%SampleID%.0521.fcs FSC-A, APC-Vio770-A subset 6302
%SampleID%.0522.fcs FSC-A, APC-Vio770-A subset 4959
%SampleID%.0523.fcs FSC-A, APC-Vio770-A subset 4835
%SampleID%.0524.fcs FSC-A, APC-Vio770-A subset 4850
%SampleID%.0525.fcs FSC-A, APC-Vio770-A subset 5905

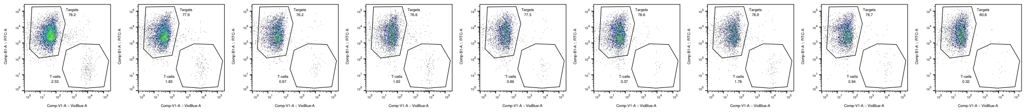

%SampleID%.0526.fcs FSC-A, APC-Vio770-A subset 8224
%SampleID%.0527.fcs FSC-A, APC-Vio770-A subset 7214
%SampleID%.0528.fcs FSC-A, APC-Vio770-A subset 5272
%SampleID%.0529.fcs FSC-A, APC-Vio770-A subset 5040
%SampleID%.0530.fcs FSC-A, APC-Vio770-A subset 4524
%SampleID%.0531.fcs FSC-A, APC-Vio770-A subset 5466
%SampleID%.0532.fcs FSC-A, APC-Vio770-A subset 4386
%SampleID%.0533.fcs FSC-A, APC-Vio770-A subset 4696
%SampleID%.0534.fcs FSC-A, APC-Vio770-A subset 4958

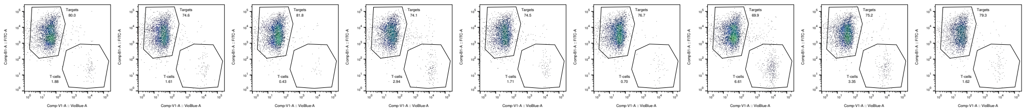

%SampleID%.0535.fcs FSC-A, APC-Vio770-A subset 4992
%SampleID%.0536.fcs FSC-A, APC-Vio770-A subset 5141
%SampleID%.0537.fcs FSC-A, APC-Vio770-A subset 5642
%SampleID%.0538.fcs FSC-A, APC-Vio770-A subset 4992
%SampleID%.0539.fcs FSC-A, APC-Vio770-A subset 4907
%SampleID%.0540.fcs FSC-A, APC-Vio770-A subset 5680
%SampleID%.0541.fcs FSC-A, APC-Vio770-A subset 6441
%SampleID%.0542.fcs FSC-A, APC-Vio770-A subset 5285
%SampleID%.0543.fcs FSC-A, APC-Vio770-A subset 4322

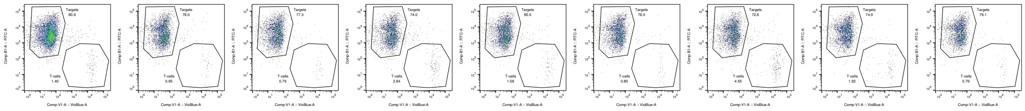

%SampleID%.0544.fcs FSC-A, APC-Vio770-A subset 7519
%SampleID%.0545.fcs FSC-A, APC-Vio770-A subset 4313
%SampleID%.0546.fcs FSC-A, APC-Vio770-A subset 4181
%SampleID%.0547.fcs FSC-A, APC-Vio770-A subset 4185
%SampleID%.0548.fcs FSC-A, APC-Vio770-A subset 4243
%SampleID%.0549.fcs FSC-A, APC-Vio770-A subset 3994
%SampleID%.0550.fcs FSC-A, APC-Vio770-A subset 4084
%SampleID%.0551.fcs FSC-A, APC-Vio770-A subset 3905
%SampleID%.0552.fcs FSC-A, APC-Vio770-A subset 3552

Plate7_JOVI_vs_H9

Well	Target	E:T ratio	Donor	CAR construct
**1A**	H9	1:4	Donor 14	JOVI_Hinge_41bbz
**1D**	H9	1:4	Donor 14	JOVI_CD8STK_28z
**1E**	H9	1:4	Donor 14	JOVI_CD28STK_CD28TM_28z
**1F**	H9	1:4	Donor 14	mJOVI_Hinge_41bbz
**1G**	H9	1:4	Donor 14	aCD19-CAR
**1H**	H9	1:4	Donor 14	Non-transduced
**2A**	H9	1:8	Donor 14	JOVI_Hinge_41bbz
**2D**	H9	1:8	Donor 14	JOVI_CD8STK_28z
**2E**	H9	1:8	Donor 14	JOVI_CD28STK_CD28TM_28z
**2F**	H9	1:8	Donor 14	mJOVI_Hinge_41bbz
**2G**	H9	1:8	Donor 14	aCD19-CAR
**2H**	H9	1:8	Donor 14	Non-transduced
**5A**	H9	1:4	Donor 15	JOVI_Hinge_41bbz
**5D**	H9	1:4	Donor 15	JOVI_CD8STK_28z
**5E**	H9	1:4	Donor 15	JOVI_CD28STK_CD28TM_28z
**5F**	H9	1:4	Donor 15	mJOVI_Hinge_41bbz
**5G**	H9	1:4	Donor 15	aCD19-CAR
**5H**	H9	1:4	Donor 15	Non-transduced
**6A**	H9	1:8	Donor 15	JOVI_Hinge_41bbz
**6D**	H9	1:8	Donor 15	JOVI_CD8STK_28z
**6E**	H9	1:8	Donor 15	JOVI_CD28STK_CD28TM_28z
**6F**	H9	1:8	Donor 15	mJOVI_Hinge_41bbz
**6G**	H9	1:8	Donor 15	aCD19-CAR
**6H**	H9	1:8	Donor 15	Non-transduced
**9A**	H9	1:4	Donor 16	JOVI_Hinge_41bbz
**9D**	H9	1:4	Donor 16	JOVI_CD8STK_28z
**9E**	H9	1:4	Donor 16	JOVI_CD28STK_CD28TM_28z
**9F**	H9	1:4	Donor 16	mJOVI_Hinge_41bbz
**9G**	H9	1:4	Donor 16	aCD19-CAR
**9H**	H9	1:4	Donor 16	Non-transduced
**10A**	H9	1:8	Donor 16	JOVI_Hinge_41bbz
**10D**	H9	1:8	Donor 16	JOVI_CD8STK_28z
**10E**	H9	1:8	Donor 16	JOVI_CD28STK_CD28TM_28z
**10F**	H9	1:8	Donor 16	mJOVI_Hinge_41bbz
**10G**	H9	1:8	Donor 16	aCD19-CAR
**10H**	H9	1:8	Donor 16	Non-transduced

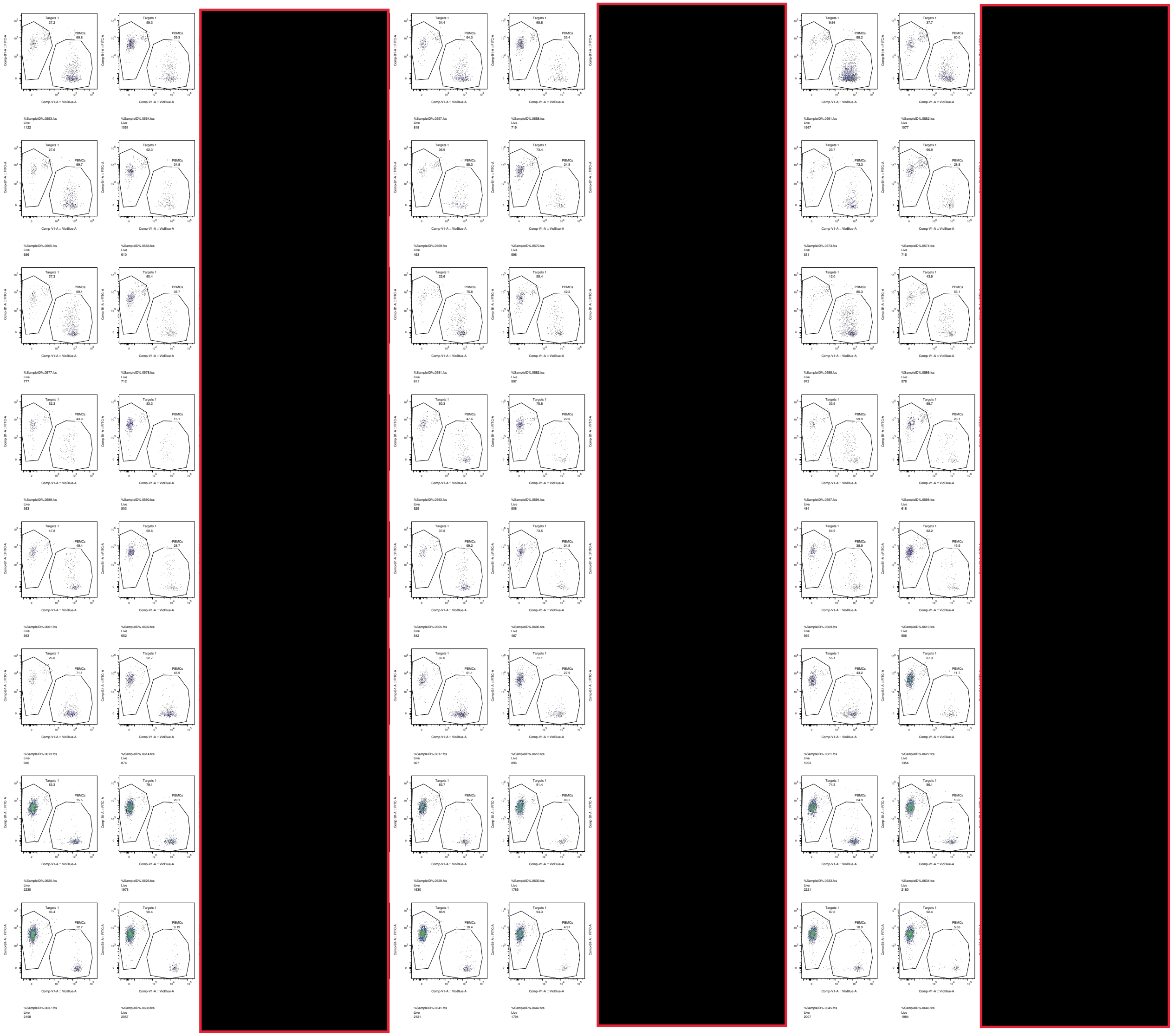

[redacted]

Plate8_JOVI_vs_TALL1

Well	Target	E:T ratio	Donor	CAR construct
**1A**	T-ALL1	1:4	Donor 14	JOVI_Hinge_41bbz
**1D**	T-ALL1	1:4	Donor 14	JOVI_CD8STK_28z
**1E**	T-ALL1	1:4	Donor 14	JOVI_CD28STK_CD28TM_28z
**1G**	T-ALL1	1:4	Donor 14	aCD19-CAR
**1H**	T-ALL1	1:4	Donor 14	Non-transduced
**2A**	T-ALL1	1:8	Donor 14	JOVI_Hinge_41bbz
**2D**	T-ALL1	1:8	Donor 14	JOVI_CD8STK_28z
**2E**	T-ALL1	1:8	Donor 14	JOVI_CD28STK_CD28TM_28z
**2G**	T-ALL1	1:8	Donor 14	aCD19-CAR
**2H**	T-ALL1	1:8	Donor 14	Non-transduced
**5A**	T-ALL1	1:4	Donor 15	JOVI_Hinge_41bbz
**5D**	T-ALL1	1:4	Donor 15	JOVI_CD8STK_28z
**5E**	T-ALL1	1:4	Donor 15	JOVI_CD28STK_CD28TM_28z
**5G**	T-ALL1	1:4	Donor 15	aCD19-CAR
**5H**	T-ALL1	1:4	Donor 15	Non-transduced
**6A**	T-ALL1	1:8	Donor 15	JOVI_Hinge_41bbz
**6D**	T-ALL1	1:8	Donor 15	JOVI_CD8STK_28z
**6E**	T-ALL1	1:8	Donor 15	JOVI_CD28STK_CD28TM_28z
**6G**	T-ALL1	1:8	Donor 15	aCD19-CAR
**6H**	T-ALL1	1:8	Donor 15	Non-transduced
**9A**	T-ALL1	1:4	Donor 16	JOVI_Hinge_41bbz
**9D**	T-ALL1	1:4	Donor 16	JOVI_CD8STK_28z
**9E**	T-ALL1	1:4	Donor 16	JOVI_CD28STK_CD28TM_28z
**9G**	T-ALL1	1:4	Donor 16	aCD19-CAR
**9H**	T-ALL1	1:4	Donor 16	Non-transduced
**10A**	T-ALL1	1:8	Donor 16	JOVI_Hinge_41bbz
**10D**	T-ALL1	1:8	Donor 16	JOVI_CD8STK_28z
**10E**	T-ALL1	1:8	Donor 16	JOVI_CD28STK_CD28TM_28z
**10G**	T-ALL1	1:8	Donor 16	aCD19-CAR
**10H**	T-ALL1	1:8	Donor 16	Non-transduced

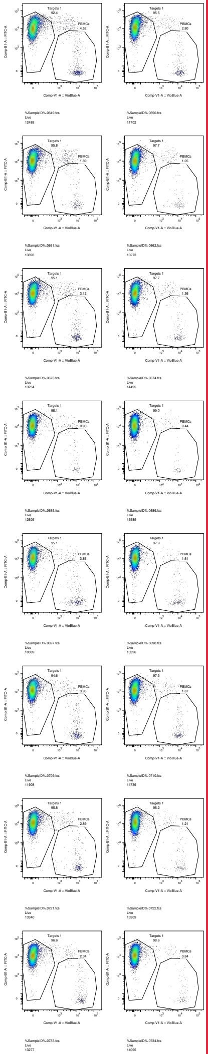

[redacted]

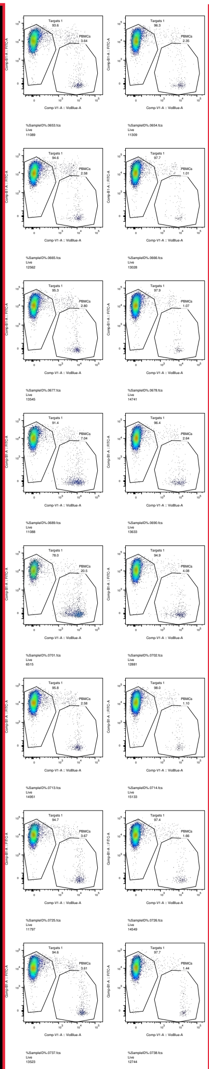

[redacted]

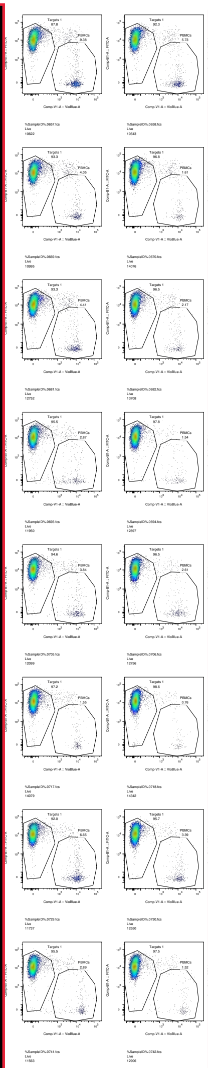

[redacted]

JOVI_ResverseKill_vs_PBMCs

Well	Target	E:T ratio	Donor	CAR construct
**1A**	Donor matched TRBC1 PBMCs	4:1	Donor 14	JOVI_Hinge_41bbz
**1D**	Donor matched TRBC1 PBMCs	4:1	Donor 14	JOVI_CD8STK_28z
**1E**	Donor matched TRBC1 PBMCs	4:1	Donor 14	JOVI_CD28STK_CD28TM_28z
**1F**	Donor matched TRBC1 PBMCs	4:1	Donor 14	mJOVI_Hinge_41bbz
**1G**	Donor matched TRBC1 PBMCs	4:1	Donor 14	aCD19-CAR
**1H**	Donor matched TRBC1 PBMCs	4:1	Donor 14	Non-transduced
**2A**	Donor matched TRBC1 PBMCs	1:1	Donor 14	JOVI_Hinge_41bbz
**2D**	Donor matched TRBC1 PBMCs	1:1	Donor 14	JOVI_CD8STK_28z
**2E**	Donor matched TRBC1 PBMCs	1:1	Donor 14	JOVI_CD28STK_CD28TM_28z
**2F**	Donor matched TRBC1 PBMCs	1:1	Donor 14	mJOVI_Hinge_41bbz
**2G**	Donor matched TRBC1 PBMCs	1:1	Donor 14	aCD19-CAR
**2H**	Donor matched TRBC1 PBMCs	1:1	Donor 14	Non-transduced
**3A**	Donor matched TRBC1 PBMCs	1:4	Donor 14	JOVI_Hinge_41bbz
**3D**	Donor matched TRBC1 PBMCs	1:4	Donor 14	JOVI_CD8STK_28z
**3E**	Donor matched TRBC1 PBMCs	1:4	Donor 14	JOVI_CD28STK_CD28TM_28z
**3F**	Donor matched TRBC1 PBMCs	1:4	Donor 14	mJOVI_Hinge_41bbz
**3G**	Donor matched TRBC1 PBMCs	1:4	Donor 15	aCD19-CAR
**3H**	Donor matched TRBC1 PBMCs	1:4	Donor 15	Non-transduced
**4A**	Donor matched TRBC1 PBMCs	4:1	Donor 15	JOVI_Hinge_41bbz
**4D**	Donor matched TRBC1 PBMCs	4:1	Donor 15	JOVI_CD8STK_28z
**4E**	Donor matched TRBC1 PBMCs	4:1	Donor 15	JOVI_CD28STK_CD28TM_28z
**4F**	Donor matched TRBC1 PBMCs	4:1	Donor 15	mJOVI_Hinge_41bbz
**4G**	Donor matched TRBC1 PBMCs	4:1	Donor 15	aCD19-CAR
**4H**	Donor matched TRBC1 PBMCs	4:1	Donor 15	Non-transduced
**5A**	Donor matched TRBC1 PBMCs	1:1	Donor 15	JOVI_Hinge_41bbz
**5D**	Donor matched TRBC1 PBMCs	1:1	Donor 15	JOVI_CD8STK_28z
**5E**	Donor matched TRBC1 PBMCs	1:1	Donor 15	JOVI_CD28STK_CD28TM_28z
**5F**	Donor matched TRBC1 PBMCs	1:1	Donor 15	mJOVI_Hinge_41bbz
**5G**	Donor matched TRBC1 PBMCs	1:1	Donor 15	aCD19-CAR
**5H**	Donor matched TRBC1 PBMCs	1:1	Donor 15	Non-transduced
**6A**	Donor matched TRBC1 PBMCs	1:4	Donor 15	JOVI_Hinge_41bbz
**6D**	Donor matched TRBC1 PBMCs	1:4	Donor 15	JOVI_CD8STK_28z
**6E**	Donor matched TRBC1 PBMCs	1:4	Donor 15	JOVI_CD28STK_CD28TM_28z
**6F**	Donor matched TRBC1 PBMCs	1:4	Donor 15	mJOVI_Hinge_41bbz
**6G**	Donor matched TRBC1 PBMCs	1:4	Donor 15	aCD19-CAR
**6H**	Donor matched TRBC1 PBMCs	1:4	Donor 15	Non-transduced
**7A**	Donor matched TRBC1 PBMCs	4:1	Donor 16	JOVI_Hinge_41bbz
**7D**	Donor matched TRBC1 PBMCs	4:1	Donor 16	JOVI_CD8STK_28z
**7E**	Donor matched TRBC1 PBMCs	4:1	Donor 16	JOVI_CD28STK_CD28TM_28z
**7F**	Donor matched TRBC1 PBMCs	4:1	Donor 16	mJOVI_Hinge_41bbz
**7G**	Donor matched TRBC1 PBMCs	4:1	Donor 16	aCD19-CAR
**7H**	Donor matched TRBC1 PBMCs	4:1	Donor 16	Non-transduced
**8A**	Donor matched TRBC1 PBMCs	1:1	Donor 16	JOVI_Hinge_41bbz
**8D**	Donor matched TRBC1 PBMCs	1:1	Donor 16	JOVI_CD8STK_28z
**8E**	Donor matched TRBC1 PBMCs	1:1	Donor 16	JOVI_CD28STK_CD28TM_28z
**8F**	Donor matched TRBC1 PBMCs	1:1	Donor 16	mJOVI_Hinge_41bbz
**8G**	Donor matched TRBC1 PBMCs	1:1	Donor 16	aCD19-CAR
**8H**	Donor matched TRBC1 PBMCs	1:1	Donor 16	Non-transduced

**9A**	Donor matched TRBC1 PBMCs	1:4	Donor 16	JOVI_Hinge_41bbz
**9D**	Donor matched TRBC1 PBMCs	1:4	Donor 16	JOVI_CD8STK_28z
**9E**	Donor matched TRBC1 PBMCs	1:4	Donor 16	JOVI_CD28STK_CD28TM_28z
**9F**	Donor matched TRBC1 PBMCs	1:4	Donor 16	mJOVI_Hinge_41bbz
**9G**	Donor matched TRBC1 PBMCs	1:4	Donor 16	aCD19-CAR
**9H**	Donor matched TRBC1 PBMCs	1:4	Donor 16	Non-transduced
**10A**	Donor matched TRBC1 PBMCs	4:1	Donor 17	JOVI_Hinge_41bbz
**10D**	Donor matched TRBC1 PBMCs	4:1	Donor 17	JOVI_CD8STK_28z
**10E**	Donor matched TRBC1 PBMCs	4:1	Donor 17	JOVI_CD28STK_CD28TM_28z
**10F**	Donor matched TRBC1 PBMCs	4:1	Donor 17	mJOVI_Hinge_41bbz
**10G**	Donor matched TRBC1 PBMCs	4:1	Donor 17	aCD19-CAR
**10H**	Donor matched TRBC1 PBMCs	4:1	Donor 17	Non-transduced
**11A**	Donor matched TRBC1 PBMCs	1:1	Donor 17	JOVI_Hinge_41bbz
**11D**	Donor matched TRBC1 PBMCs	1:1	Donor 17	JOVI_CD8STK_28z
**11E**	Donor matched TRBC1 PBMCs	1:1	Donor 17	JOVI_CD28STK_CD28TM_28z
**11F**	Donor matched TRBC1 PBMCs	1:1	Donor 17	mJOVI_Hinge_41bbz
**11G**	Donor matched TRBC1 PBMCs	1:1	Donor 17	aCD19-CAR
**11H**	Donor matched TRBC1 PBMCs	1:1	Donor 17	Non-transduced
**12A**	Donor matched TRBC1 PBMCs	1:4	Donor 17	JOVI_Hinge_41bbz
**12D**	Donor matched TRBC1 PBMCs	1:4	Donor 17	JOVI_CD8STK_28z
**12E**	Donor matched TRBC1 PBMCs	1:4	Donor 17	JOVI_CD28STK_CD28TM_28z
**12F**	Donor matched TRBC1 PBMCs	1:4	Donor 17	mJOVI_Hinge_41bbz
**12G**	Donor matched TRBC1 PBMCs	1:4	Donor 17	aCD19-CAR
**12H**	Donor matched TRBC1 PBMCs	1:4	Donor 17	Non-transduced

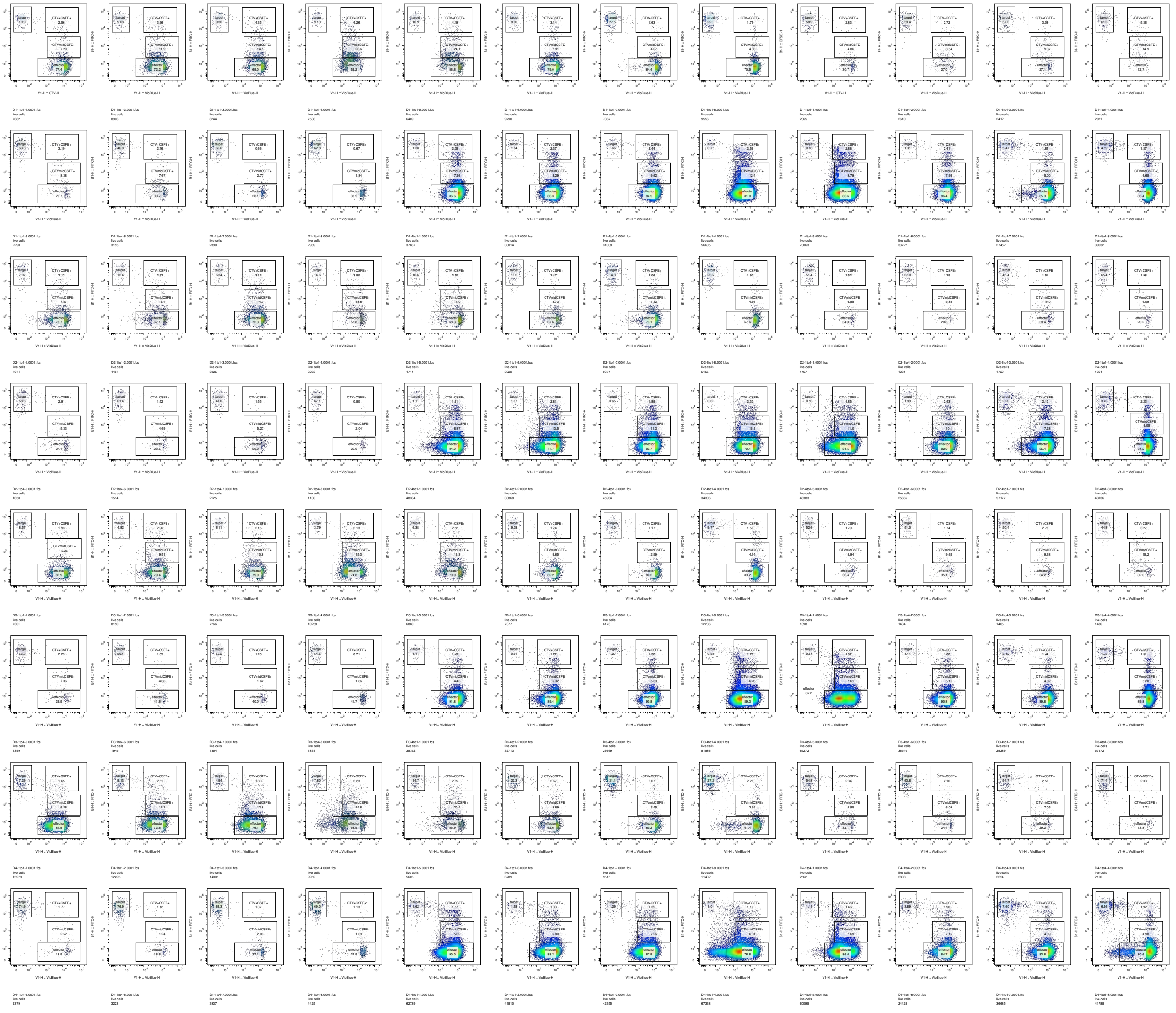

KFN_ReverseKill_vs_PBMCs

**Well**	**Target**	**E:T ratio**	**Donor**	**CAR construct**
**1B**	Donor matched TRBC2 PBMCs	4:1	Donor 14	KFN_Hinge_28z
**1D**	Donor matched TRBC2 PBMCs	4:1	Donor 14	KFN_CD8STK_28z
**1E**	Donor matched TRBC2 PBMCs	4:1	Donor 14	KFN_CD28STK_CD28TM_28z
**1F**	Donor matched TRBC2 PBMCs	4:1	Donor 14	aCD19-CAR
**1G**	Donor matched TRBC2 PBMCs	4:1	Donor 14	Non-transduced
**1H**	Donor matched TRBC2 PBMCs	N/A	Donor 14	N/A
**2B**	Donor matched TRBC2 PBMCs	1:1	Donor 14	KFN_Hinge_28z
**2D**	Donor matched TRBC2 PBMCs	1:1	Donor 14	KFN_CD8STK_28z
**2E**	Donor matched TRBC2 PBMCs	1:1	Donor 14	KFN_CD28STK_CD28TM_28z
**2F**	Donor matched TRBC2 PBMCs	1:1	Donor 14	aCD19-CAR
**2G**	Donor matched TRBC2 PBMCs	1:1	Donor 14	Non-transduced
**2H**	Donor matched TRBC2 PBMCs	N/A	Donor 14	N/A
**3B**	Donor matched TRBC2 PBMCs	1:4	Donor 14	KFN_Hinge_28z
**3D**	Donor matched TRBC2 PBMCs	1:4	Donor 14	KFN_CD8STK_28z
**3E**	Donor matched TRBC2 PBMCs	1:4	Donor 14	KFN_CD28STK_CD28TM_28z
**3F**	Donor matched TRBC2 PBMCs	1:4	Donor 14	aCD19-CAR
**3G**	Donor matched TRBC2 PBMCs	1:4	Donor 15	Non-transduced
**3H**	Donor matched TRBC2 PBMCs	N/A	Donor 15	N/A
**4B**	Donor matched TRBC2 PBMCs	4:1	Donor 15	KFN_Hinge_28z
**4D**	Donor matched TRBC2 PBMCs	4:1	Donor 15	KFN_CD8STK_28z
**4E**	Donor matched TRBC2 PBMCs	4:1	Donor 15	KFN_CD28STK_CD28TM_28z
**4F**	Donor matched TRBC2 PBMCs	4:1	Donor 15	aCD19-CAR
**4G**	Donor matched TRBC2 PBMCs	4:1	Donor 15	Non-transduced
**4H**	Donor matched TRBC2 PBMCs	N/A	Donor 15	N/A
**5B**	Donor matched TRBC2 PBMCs	1:1	Donor 15	KFN_Hinge_28z
**5D**	Donor matched TRBC2 PBMCs	1:1	Donor 15	KFN_CD8STK_28z
**5E**	Donor matched TRBC2 PBMCs	1:1	Donor 15	KFN_CD28STK_CD28TM_28z
**5F**	Donor matched TRBC2 PBMCs	1:1	Donor 15	aCD19-CAR
**5G**	Donor matched TRBC2 PBMCs	1:1	Donor 15	Non-transduced
**5H**	Donor matched TRBC2 PBMCs	N/A	Donor 15	N/A
**6B**	Donor matched TRBC2 PBMCs	1:4	Donor 15	KFN_Hinge_28z
**6D**	Donor matched TRBC2 PBMCs	1:4	Donor 15	KFN_CD8STK_28z
**6E**	Donor matched TRBC2 PBMCs	1:4	Donor 15	KFN_CD28STK_CD28TM_28z
**6F**	Donor matched TRBC2 PBMCs	1:4	Donor 15	aCD19-CAR
**6G**	Donor matched TRBC2 PBMCs	1:4	Donor 15	Non-transduced
**6H**	Donor matched TRBC2 PBMCs	N/A	Donor 15	N/A
**7B**	Donor matched TRBC2 PBMCs	4:1	Donor 16	KFN_Hinge_28z
**7D**	Donor matched TRBC2 PBMCs	4:1	Donor 16	KFN_CD8STK_28z
**7E**	Donor matched TRBC2 PBMCs	4:1	Donor 16	KFN_CD28STK_CD28TM_28z
**7F**	Donor matched TRBC2 PBMCs	4:1	Donor 16	aCD19-CAR
**7G**	Donor matched TRBC2 PBMCs	4:1	Donor 16	Non-transduced
**7H**	Donor matched TRBC2 PBMCs	N/A	Donor 16	N/A
**8B**	Donor matched TRBC2 PBMCs	1:1	Donor 16	KFN_Hinge_28z
**8D**	Donor matched TRBC2 PBMCs	1:1	Donor 16	KFN_CD8STK_28z
**8E**	Donor matched TRBC2 PBMCs	1:1	Donor 16	KFN_CD28STK_CD28TM_28z
**8F**	Donor matched TRBC2 PBMCs	1:1	Donor 16	aCD19-CAR
**8G**	Donor matched TRBC2 PBMCs	1:1	Donor 16	Non-transduced
**8H**	Donor matched TRBC2 PBMCs	N/A	Donor 16	N/A

**9B**	Donor matched TRBC2 PBMCs	1:4	Donor 16	KFN_Hinge_28z
**9D**	Donor matched TRBC2 PBMCs	1:4	Donor 16	KFN_CD8STK_28z
**9E**	Donor matched TRBC2 PBMCs	1:4	Donor 16	KFN_CD28STK_CD28TM_28z
**9F**	Donor matched TRBC2 PBMCs	1:4	Donor 16	aCD19-CAR
**9G**	Donor matched TRBC2 PBMCs	1:4	Donor 16	Non-transduced
**9H**	Donor matched TRBC2 PBMCs	N/A	Donor 16	N/A
**10B**	Donor matched TRBC2 PBMCs	4:1	Donor 17	KFN_Hinge_28z
**10D**	Donor matched TRBC2 PBMCs	4:1	Donor 17	KFN_CD8STK_28z
**10E**	Donor matched TRBC2 PBMCs	4:1	Donor 17	KFN_CD28STK_CD28TM_28z
**10F**	Donor matched TRBC2 PBMCs	4:1	Donor 17	aCD19-CAR
**10G**	Donor matched TRBC2 PBMCs	4:1	Donor 17	Non-transduced
**10H**	Donor matched TRBC2 PBMCs	N/A	Donor 17	N/A
**11B**	Donor matched TRBC2 PBMCs	1:1	Donor 17	KFN_Hinge_28z
**11D**	Donor matched TRBC2 PBMCs	1:1	Donor 17	KFN_CD8STK_28z
**11E**	Donor matched TRBC2 PBMCs	1:1	Donor 17	KFN_CD28STK_CD28TM_28z
**11F**	Donor matched TRBC2 PBMCs	1:1	Donor 17	aCD19-CAR
**11G**	Donor matched TRBC2 PBMCs	1:1	Donor 17	Non-transduced
**11H**	Donor matched TRBC2 PBMCs	N/A	Donor 17	N/A
**12B**	Donor matched TRBC2 PBMCs	1:4	Donor 17	KFN_Hinge_28z
**12D**	Donor matched TRBC2 PBMCs	1:4	Donor 17	KFN_CD8STK_28z
**12E**	Donor matched TRBC2 PBMCs	1:4	Donor 17	KFN_CD28STK_CD28TM_28z
**12F**	Donor matched TRBC2 PBMCs	1:4	Donor 17	aCD19-CAR
**12G**	Donor matched TRBC2 PBMCs	1:4	Donor 17	Non-transduced
**12H**	Donor matched TRBC2 PBMCs	N/A	Donor 17	N/A

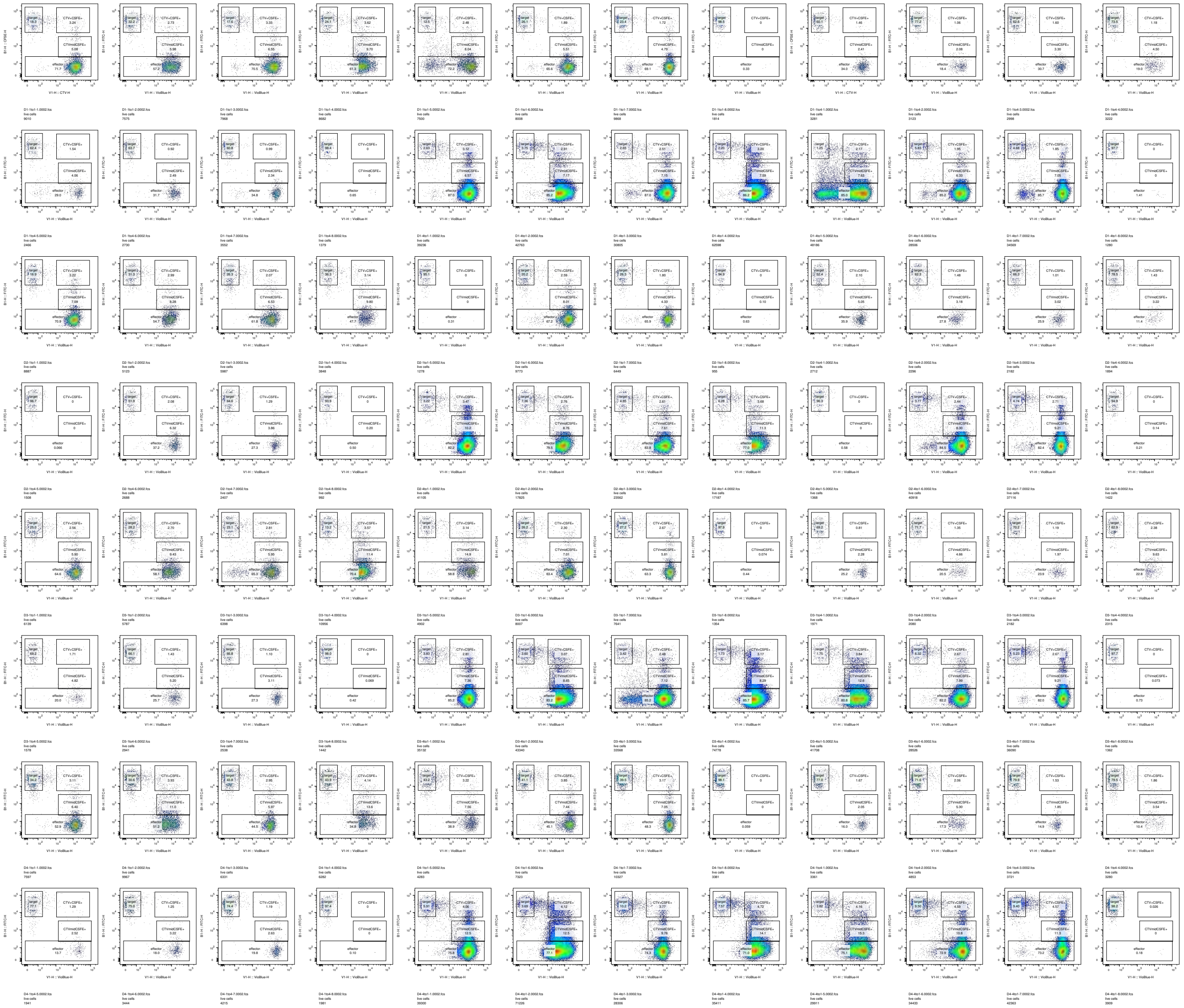

Plate19_KFN-Prol_vs_Jurkat

Well	Target	E:T ratio	Donor	CAR construct
1B	Jurkat TRBC1+ve	1:4	Donor 14	KFN_Hinge_28z
1D	Jurkat TRBC1+ve	1:4	Donor 14	KFN_CD8STK_28z
1E	Jurkat TRBC1+ve	1:4	Donor 14	KFN_CD28STK_CD28TM_28z
1F	Jurkat TRBC1+ve	1:4	Donor 14	aCD19-CAR
1G	Jurkat TRBC1+ve	1:4	Donor 14	Non-transduced
1H	Jurkat TRBC1+ve	N/A	N/A	N/A
2B	Jurkat TRBC1+ve	1:4	Donor 15	KFN_Hinge_28z
2D	Jurkat TRBC1+ve	1:4	Donor 15	KFN_CD8STK_28z
2E	Jurkat TRBC1+ve	1:4	Donor 15	KFN_CD28STK_CD28TM_28z
2F	Jurkat TRBC1+ve	1:4	Donor 15	aCD19-CAR
2G	Jurkat TRBC1+ve	1:4	Donor 15	Non-transduced
2H	Jurkat TRBC1+ve	N/A	N/A	N/A
3B	Jurkat TRBC1+ve	1:4	Donor 16	KFN_Hinge_28z
3D	Jurkat TRBC1+ve	1:4	Donor 16	KFN_CD8STK_28z
3E	Jurkat TRBC1+ve	1:4	Donor 16	KFN_CD28STK_CD28TM_28z
3F	Jurkat TRBC1+ve	1:4	Donor 16	aCD19-CAR
3G	Jurkat TRBC1+ve	1:4	Donor 16	Non-transduced
3H	Jurkat TRBC1+ve	N/A	N/A	N/A
4B	Jurkat TRBC2+ve	1:4	Donor 14	KFN_Hinge_28z
4D	Jurkat TRBC2+ve	1:4	Donor 14	KFN_CD8STK_28z
4E	Jurkat TRBC2+ve	1:4	Donor 14	KFN_CD28STK_CD28TM_28z
4F	Jurkat TRBC2+ve	1:4	Donor 14	aCD19-CAR
4G	Jurkat TRBC2+ve	1:4	Donor 14	Non-transduced
4H	Jurkat TRBC2+ve	N/A	N/A	N/A
5B	Jurkat TRBC2+ve	1:4	Donor 15	KFN_Hinge_28z
5D	Jurkat TRBC2+ve	1:4	Donor 15	KFN_CD8STK_28z
5E	Jurkat TRBC2+ve	1:4	Donor 15	KFN_CD28STK_CD28TM_28z
5F	Jurkat TRBC2+ve	1:4	Donor 15	aCD19-CAR
5G	Jurkat TRBC2+ve	1:4	Donor 15	Non-transduced
5H	Jurkat TRBC2+ve	N/A	N/A	N/A
6B	Jurkat TRBC2+ve	1:4	Donor 16	KFN_Hinge_28z
6D	Jurkat TRBC2+ve	1:4	Donor 16	KFN_CD8STK_28z
6E	Jurkat TRBC2+ve	1:4	Donor 16	KFN_CD28STK_CD28TM_28z
6F	Jurkat TRBC2+ve	1:4	Donor 16	aCD19-CAR
6G	Jurkat TRBC2+ve	1:4	Donor 16	Non-transduced
6H	Jurkat TRBC2+ve	N/A	N/A	N/A
7B	Jurkat TRBC KO	1:4	Donor 14	KFN_Hinge_28z
7D	Jurkat TRBC KO	1:4	Donor 14	KFN_CD8STK_28z
7E	Jurkat TRBC KO	1:4	Donor 14	KFN_CD28STK_CD28TM_28z
7F	Jurkat TRBC KO	1:4	Donor 14	aCD19-CAR
7G	Jurkat TRBC KO	1:4	Donor 14	Non-transduced
7H	Jurkat TRBC KO	N/A	N/A	N/A
8B	Jurkat TRBC KO	1:4	Donor 15	KFN_Hinge_28z
8D	Jurkat TRBC KO	1:4	Donor 15	KFN_CD8STK_28z
8E	Jurkat TRBC KO	1:4	Donor 15	KFN_CD28STK_CD28TM_28z
8F	Jurkat TRBC KO	1:4	Donor 15	aCD19-CAR
8G	Jurkat TRBC KO	1:4	Donor 15	Non-transduced
8H	Jurkat TRBC KO	N/A	N/A	N/A

**9B**	Jurkat TRBC KO	1:4	Donor 16	KFN_Hinge_28z
**9D**	Jurkat TRBC KO	1:4	Donor 16	KFN_CD8STK_28z
**9E**	Jurkat TRBC KO	1:4	Donor 16	KFN_CD28STK_CD28TM_28z
**9F**	Jurkat TRBC KO	1:4	Donor 16	aCD19-CAR
**9G**	Jurkat TRBC KO	1:4	Donor 16	Non-transduced
**9H**	Jurkat TRBC KO	N/A	N/A	N/A
**10A**	N/A	N/A	N/A	N/A
**10B**	N/A	N/A	N/A	N/A
**10C**	N/A	N/A	N/A	N/A
**10D**	N/A	N/A	N/A	N/A
**10E**	N/A	N/A	N/A	N/A
**10F**	N/A	N/A	N/A	N/A
**10G**	N/A	N/A	N/A	N/A
**10H**	N/A	N/A	N/A	N/A
**11A**	N/A	N/A	N/A	N/A
**11B**	N/A	N/A	N/A	N/A
**11C**	N/A	N/A	N/A	N/A
**11D**	N/A	N/A	N/A	N/A
**11E**	N/A	N/A	N/A	N/A
**11F**	N/A	N/A	N/A	N/A
**11G**	N/A	N/A	N/A	N/A
**11H**	N/A	N/A	N/A	N/A
**12A**	N/A	N/A	N/A	N/A
**12B**	N/A	N/A	N/A	N/A
**12C**	N/A	N/A	N/A	N/A
**12D**	N/A	N/A	N/A	N/A
**12E**	N/A	N/A	N/A	N/A
**12F**	N/A	N/A	N/A	N/A
**12G**	N/A	N/A	N/A	N/A
**12H**	N/A	N/A	N/A	N/A

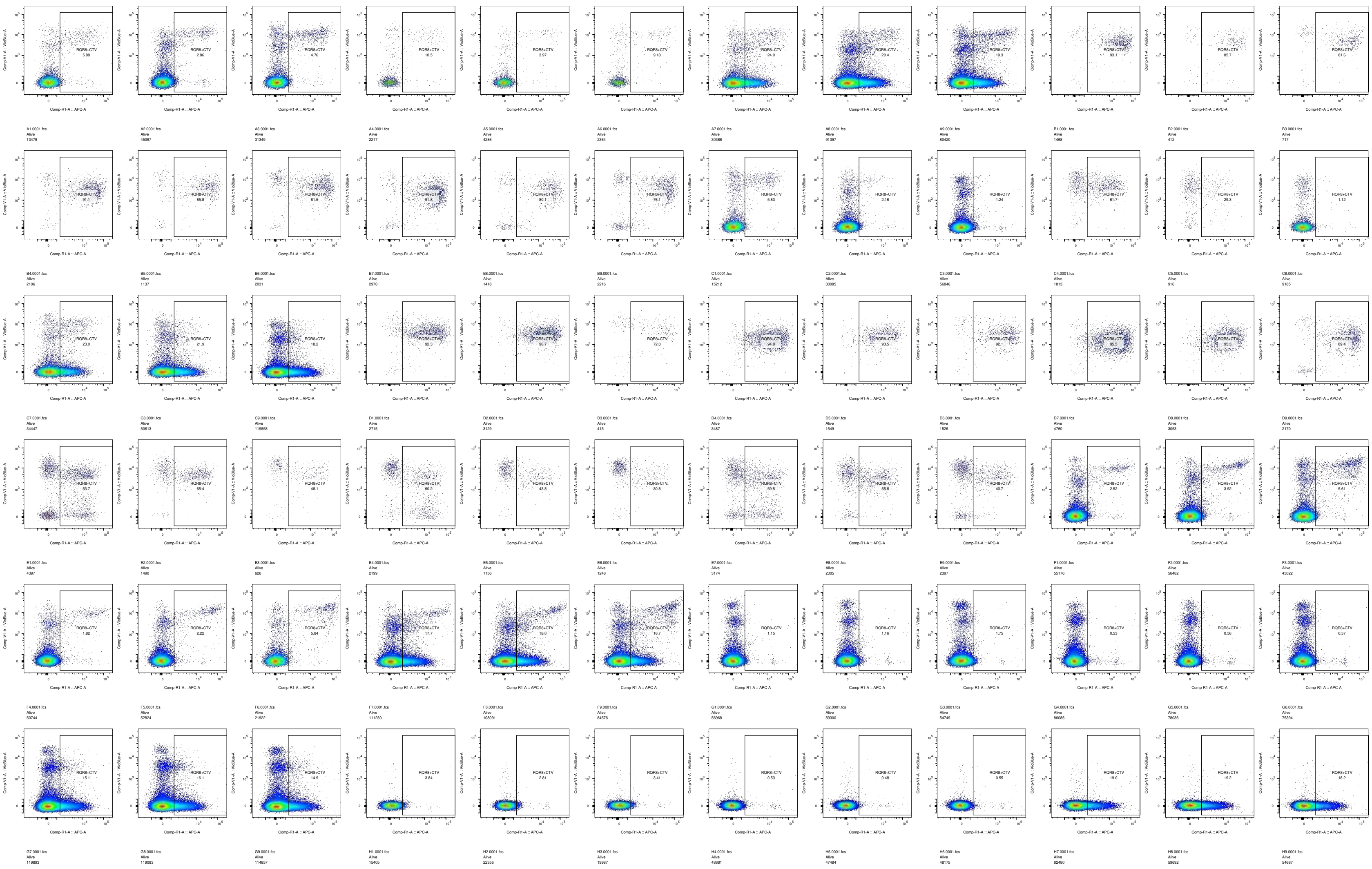
Comp-V1-A :: VioBlue-A
Comp-R1-A :: APC-A
RQR8+CTV 5.88
A1.0001.fcs
Alive
13479
RQR8+CTV 2.86
A2.0001.fcs
Alive
45067
RQR8+CTV 4.76
A3.0001.fcs
Alive
31349
RQR8+CTV 10.5
A4.0001.fcs
Alive
2217
RQR8+CTV 3.97
A5.0001.fcs
Alive
4286
RQR8+CTV 9.18
A6.0001.fcs
Alive
2364
RQR8+CTV 24.0
A7.0001.fcs
Alive
30366
RQR8+CTV 20.4
A8.0001.fcs
Alive
91397
RQR8+CTV 19.3
A9.0001.fcs
Alive
80420
RQR8+CTV 93.1
B1.0001.fcs
Alive
1468
RQR8+CTV 85.7
B2.0001.fcs
Alive
412
RQR8+CTV 81.6
B3.0001.fcs
Alive
717
RQR8+CTV 91.1
B4.0001.fcs
Alive
2106
RQR8+CTV 85.8
B5.0001.fcs
Alive
1137
RQR8+CTV 81.5
B6.0001.fcs
Alive
2031
RQR8+CTV 91.8
B7.0001.fcs
Alive
2970
RQR8+CTV 80.1
B8.0001.fcs
Alive
1418
RQR8+CTV 76.1
B9.0001.fcs
Alive
2216
RQR8+CTV 5.83
C1.0001.fcs
Alive
15212
RQR8+CTV 2.16
C2.0001.fcs
Alive
30085
RQR8+CTV 1.24
C3.0001.fcs
Alive
56846
RQR8+CTV 61.7
C4.0001.fcs
Alive
1813
RQR8+CTV 29.3
C5.0001.fcs
Alive
916
RQR8+CTV 1.12
C6.0001.fcs
Alive
9185
RQR8+CTV 23.0
C7.0001.fcs
Alive
34447
RQR8+CTV 21.9
C8.0001.fcs
Alive
50613
RQR8+CTV 18.2
C9.0001.fcs
Alive
119858
RQR8+CTV 92.3
D1.0001.fcs
Alive
2715
RQR8+CTV 96.7
D2.0001.fcs
Alive
3129
RQR8+CTV 72.0
D3.0001.fcs
Alive
415
RQR8+CTV 94.8
D4.0001.fcs
Alive
3487
RQR8+CTV 93.5
D5.0001.fcs
Alive
1549
RQR8+CTV 92.1
D6.0001.fcs
Alive
1526
RQR8+CTV 95.5
D7.0001.fcs
Alive
4760
RQR8+CTV 95.3
D8.0001.fcs
Alive
3053
RQR8+CTV 89.4
D9.0001.fcs
Alive
2170
RQR8+CTV 53.7
E1.0001.fcs
Alive
4397
RQR8+CTV 65.4
E2.0001.fcs
Alive
1490
RQR8+CTV 48.1
E3.0001.fcs
Alive
626
RQR8+CTV 60.2
E4.0001.fcs
Alive
2199
RQR8+CTV 43.8
E5.0001.fcs
Alive
1156
RQR8+CTV 30.8
E6.0001.fcs
Alive
1248
RQR8+CTV 59.5
E7.0001.fcs
Alive
3174
RQR8+CTV 55.8
E8.0001.fcs
Alive
2305
RQR8+CTV 40.7
E9.0001.fcs
Alive
2397
RQR8+CTV 2.52
F1.0001.fcs
Alive
55176
RQR8+CTV 3.52
F2.0001.fcs
Alive
56482
RQR8+CTV 5.61
F3.0001.fcs
Alive
43022
RQR8+CTV 1.82
F4.0001.fcs
Alive
50744
RQR8+CTV 2.22
F5.0001.fcs
Alive
52824
RQR8+CTV 5.84
F6.0001.fcs
Alive
21922
RQR8+CTV 17.7
F7.0001.fcs
Alive
111230
RQR8+CTV 18.0
F8.0001.fcs
Alive
108091
RQR8+CTV 16.7
F9.0001.fcs
Alive
84576
RQR8+CTV 1.15
G1.0001.fcs
Alive
56968
RQR8+CTV 1.16
G2.0001.fcs
Alive
59300
RQR8+CTV 1.75
G3.0001.fcs
Alive
54749
RQR8+CTV 0.53
G4.0001.fcs
Alive
86085
RQR8+CTV 0.56
G5.0001.fcs
Alive
78036
RQR8+CTV 0.57
G6.0001.fcs
Alive
75394
RQR8+CTV 15.1
G7.0001.fcs
Alive
119893
RQR8+CTV 16.1
G8.0001.fcs
Alive
119083
RQR8+CTV 14.9
G9.0001.fcs
Alive
114857
RQR8+CTV 3.84
H1.0001.fcs
Alive
15405
RQR8+CTV 2.81
H2.0001.fcs
Alive
22355
RQR8+CTV 3.41
H3.0001.fcs
Alive
19987
RQR8+CTV 0.53
H4.0001.fcs
Alive
48881
RQR8+CTV 0.48
H5.0001.fcs
Alive
47484
RQR8+CTV 0.55
H6.0001.fcs
Alive
48175
RQR8+CTV 19.0
H7.0001.fcs
Alive
62480
RQR8+CTV 19.2
H8.0001.fcs
Alive
59692
RQR8+CTV 18.2
H9.0001.fcs
Alive
54687

Plate20-21_KFN-Prol_HPB_H9_TALL

Well	Target	E:T ratio	Donor	CAR construct
**1B**	HPB TRBC1+ve	1:4	Donor 14	KFN_Hinge_28z
**1D**	HPB TRBC1+ve	1:4	Donor 14	KFN_CD8STK_28z
**1E**	HPB TRBC1+ve	1:4	Donor 14	KFN_CD28STK_CD28TM_28z
**1F**	HPB TRBC1+ve	1:4	Donor 14	aCD19-CAR
**1G**	HPB TRBC1+ve	1:4	Donor 14	Non-transduced
**1H**	HPB TRBC1+ve	N/A	N/A	N/A
**2B**	HPB TRBC1+ve	1:4	Donor 15	KFN_Hinge_28z
**2D**	HPB TRBC1+ve	1:4	Donor 15	KFN_CD8STK_28z
**2E**	HPB TRBC1+ve	1:4	Donor 15	KFN_CD28STK_CD28TM_28z
**2F**	HPB TRBC1+ve	1:4	Donor 15	aCD19-CAR
**2G**	HPB TRBC1+ve	1:4	Donor 15	Non-transduced
**2H**	HPB TRBC1+ve	N/A	N/A	N/A
**3B**	HPB TRBC1+ve	1:4	Donor 16	KFN_Hinge_28z
**3D**	HPB TRBC1+ve	1:4	Donor 16	KFN_CD8STK_28z
**3E**	HPB TRBC1+ve	1:4	Donor 16	KFN_CD28STK_CD28TM_28z
**3F**	HPB TRBC1+ve	1:4	Donor 16	aCD19-CAR
**3G**	HPB TRBC1+ve	1:4	Donor 16	Non-transduced
**3H**	HPB TRBC1+ve	N/A	N/A	N/A
**4B**	HPB TRBC2+ve	1:4	Donor 14	KFN_Hinge_28z
**4D**	HPB TRBC2+ve	1:4	Donor 14	KFN_CD8STK_28z
**4E**	HPB TRBC2+ve	1:4	Donor 14	KFN_CD28STK_CD28TM_28z
**4F**	HPB TRBC2+ve	1:4	Donor 14	aCD19-CAR
**4G**	HPB TRBC2+ve	1:4	Donor 14	Non-transduced
**4H**	HPB TRBC2+ve	N/A	N/A	N/A
**5B**	HPB TRBC2+ve	1:4	Donor 15	KFN_Hinge_28z
**5D**	HPB TRBC2+ve	1:4	Donor 15	KFN_CD8STK_28z
**5E**	HPB TRBC2+ve	1:4	Donor 15	KFN_CD28STK_CD28TM_28z
**5F**	HPB TRBC2+ve	1:4	Donor 15	aCD19-CAR
**5G**	HPB TRBC2+ve	1:4	Donor 15	Non-transduced
**5H**	HPB TRBC2+ve	N/A	N/A	N/A
**6B**	HPB TRBC2+ve	1:4	Donor 16	KFN_Hinge_28z
**6D**	HPB TRBC2+ve	1:4	Donor 16	KFN_CD8STK_28z
**6E**	HPB TRBC2+ve	1:4	Donor 16	KFN_CD28STK_CD28TM_28z
**6F**	HPB TRBC2+ve	1:4	Donor 16	aCD19-CAR
**6G**	HPB TRBC2+ve	1:4	Donor 16	Non-transduced
**6H**	HPB TRBC2+ve	N/A	N/A	N/A
**7B**	H9	1:4	Donor 14	KFN_Hinge_28z
**7D**	H9	1:4	Donor 14	KFN_CD8STK_28z
**7E**	H9	1:4	Donor 14	KFN_CD28STK_CD28TM_28z
**7F**	H9	1:4	Donor 14	aCD19-CAR
**7G**	H9	1:4	Donor 14	Non-transduced
**7H**	H9	N/A	N/A	N/A
**8B**	H9	1:4	Donor 15	KFN_Hinge_28z
**8D**	H9	1:4	Donor 15	KFN_CD8STK_28z
**8E**	H9	1:4	Donor 15	KFN_CD28STK_CD28TM_28z
**8F**	H9	1:4	Donor 15	aCD19-CAR
**8G**	H9	1:4	Donor 15	Non-transduced
**8H**	H9	N/A	N/A	N/A

**9B**	H9	1:4	Donor 16	KFN_Hinge_28z
**9D**	H9	1:4	Donor 16	KFN_CD8STK_28z
**9E**	H9	1:4	Donor 16	KFN_CD28STK_CD28TM_28z
**9F**	H9	1:4	Donor 16	aCD19-CAR
**9G**	H9	1:4	Donor 16	Non-transduced
**9H**	H9	N/A	N/A	N/A
**10B**	T-ALL1	1:4	Donor 14	KFN_Hinge_28z
**10D**	T-ALL1	1:4	Donor 14	KFN_CD8STK_28z
**10E**	T-ALL1	1:4	Donor 14	KFN_CD28STK_CD28TM_28z
**10F**	T-ALL1	1:4	Donor 14	aCD19-CAR
**10G**	T-ALL1	1:4	Donor 14	Non-transduced
**10H**	T-ALL1	N/A	N/A	N/A
**11B**	T-ALL1	1:4	Donor 15	KFN_Hinge_28z
**11D**	T-ALL1	1:4	Donor 15	KFN_CD8STK_28z
**11E**	T-ALL1	1:4	Donor 15	KFN_CD28STK_CD28TM_28z
**11F**	T-ALL1	1:4	Donor 15	aCD19-CAR
**11G**	T-ALL1	1:4	Donor 15	Non-transduced
**11H**	T-ALL1	N/A	N/A	N/A
**12B**	T-ALL1	1:4	Donor 16	KFN_Hinge_28z
**12D**	T-ALL1	1:4	Donor 16	KFN_CD8STK_28z
**12E**	T-ALL1	1:4	Donor 16	KFN_CD28STK_CD28TM_28z
**12F**	T-ALL1	1:4	Donor 16	aCD19-CAR
**12G**	T-ALL1	1:4	Donor 16	Non-transduced
**12H**	T-ALL1	N/A	N/A	N/A

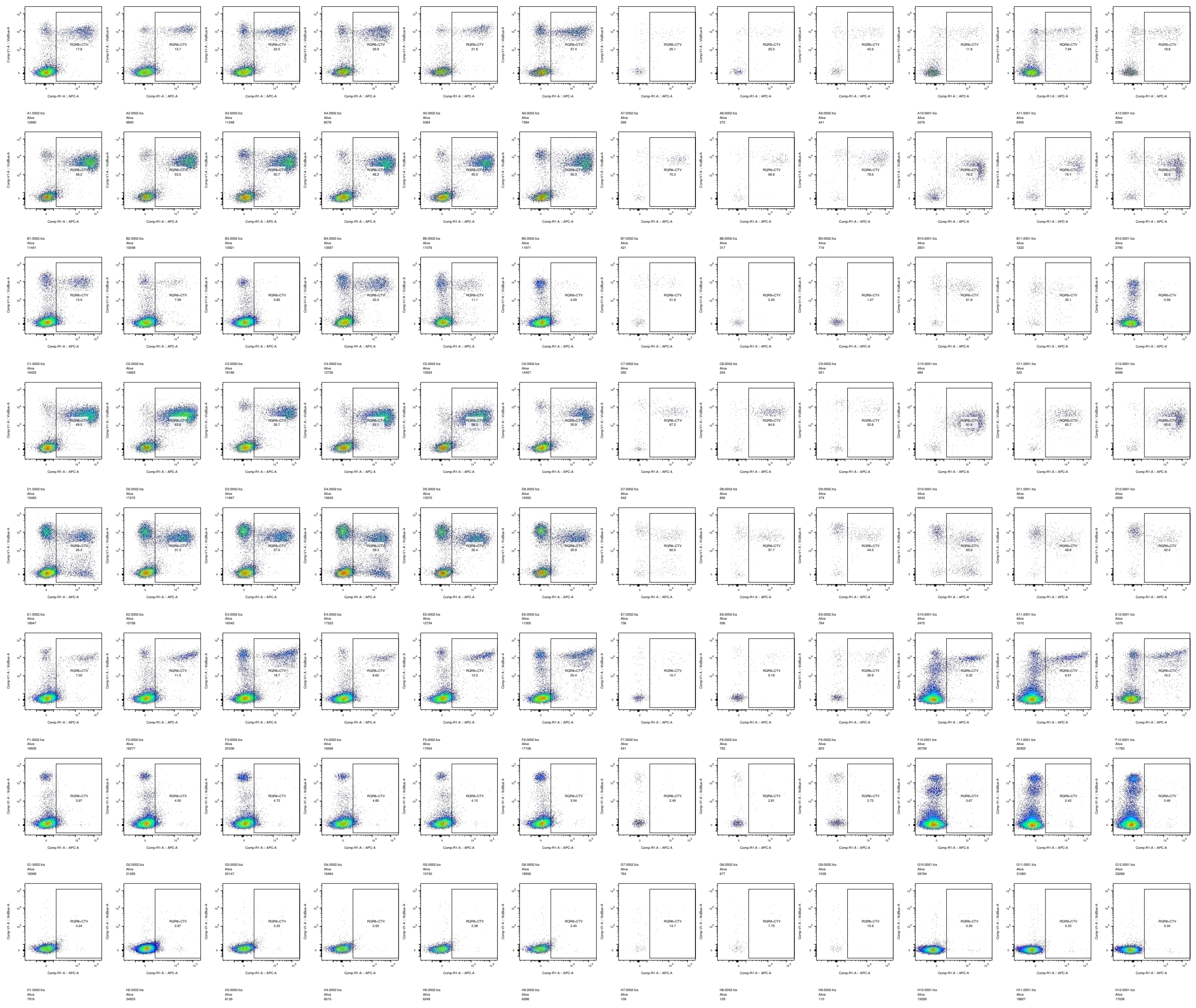

Plate22_JOVI-Prol_vs_Jurkat

Well	Target	E:T ratio	Donor	CAR construct
**1A**	Jurkat TRBC1+ve	1:4	Donor 14	JOVI_Hinge_41bbz
**1D**	Jurkat TRBC1+ve	1:4	Donor 14	JOVI_CD8STK_28z
**1E**	Jurkat TRBC1+ve	1:4	Donor 14	JOVI_CD28STK_CD28TM_28z
**1F**	Jurkat TRBC1+ve	1:4	Donor 14	mJOVI_Hinge_41bbz
**1G**	Jurkat TRBC1+ve	1:4	Donor 14	aCD19-CAR
**1H**	Jurkat TRBC1+ve	1:4	Donor 14	Non-transduced
**2A**	Jurkat TRBC1+ve	1:4	Donor 15	JOVI_Hinge_41bbz
**2D**	Jurkat TRBC1+ve	1:4	Donor 15	JOVI_CD8STK_28z
**2E**	Jurkat TRBC1+ve	1:4	Donor 15	JOVI_CD28STK_CD28TM_28z
**2F**	Jurkat TRBC1+ve	1:4	Donor 15	mJOVI_Hinge_41bbz
**2G**	Jurkat TRBC1+ve	1:4	Donor 15	aCD19-CAR
**2H**	Jurkat TRBC1+ve	1:4	Donor 15	Non-transduced
**3A**	Jurkat TRBC1+ve	1:4	Donor 16	JOVI_Hinge_41bbz
**3D**	Jurkat TRBC1+ve	1:4	Donor 16	JOVI_CD8STK_28z
**3E**	Jurkat TRBC1+ve	1:4	Donor 16	JOVI_CD28STK_CD28TM_28z
**3F**	Jurkat TRBC1+ve	1:4	Donor 16	mJOVI_Hinge_41bbz
**3G**	Jurkat TRBC1+ve	1:4	Donor 16	aCD19-CAR
**3H**	Jurkat TRBC1+ve	1:4	Donor 16	Non-transduced
**4A**	Jurkat TRBC2+ve	1:4	Donor 14	JOVI_Hinge_41bbz
**4D**	Jurkat TRBC2+ve	1:4	Donor 14	JOVI_CD8STK_28z
**4E**	Jurkat TRBC2+ve	1:4	Donor 14	JOVI_CD28STK_CD28TM_28z
**4F**	Jurkat TRBC2+ve	1:4	Donor 14	mJOVI_Hinge_41bbz
**4G**	Jurkat TRBC2+ve	1:4	Donor 14	aCD19-CAR
**4H**	Jurkat TRBC2+ve	1:4	Donor 14	Non-transduced
**5A**	Jurkat TRBC2+ve	1:4	Donor 15	JOVI_Hinge_41bbz
**5D**	Jurkat TRBC2+ve	1:4	Donor 15	JOVI_CD8STK_28z
**5E**	Jurkat TRBC2+ve	1:4	Donor 15	JOVI_CD28STK_CD28TM_28z
**5F**	Jurkat TRBC2+ve	1:4	Donor 15	mJOVI_Hinge_41bbz
**5G**	Jurkat TRBC2+ve	1:4	Donor 15	aCD19-CAR
**5H**	Jurkat TRBC2+ve	1:4	Donor 15	Non-transduced
**6A**	Jurkat TRBC2+ve	1:4	Donor 16	JOVI_Hinge_41bbz
**6D**	Jurkat TRBC2+ve	1:4	Donor 16	JOVI_CD8STK_28z
**6E**	Jurkat TRBC2+ve	1:4	Donor 16	JOVI_CD28STK_CD28TM_28z
**6F**	Jurkat TRBC2+ve	1:4	Donor 16	mJOVI_Hinge_41bbz
**6G**	Jurkat TRBC2+ve	1:4	Donor 16	aCD19-CAR
**6H**	Jurkat TRBC2+ve	1:4	Donor 16	Non-transduced
**7A**	Jurkat TRBC KO	1:4	Donor 14	JOVI_Hinge_41bbz
**7D**	Jurkat TRBC KO	1:4	Donor 14	JOVI_CD8STK_28z
**7E**	Jurkat TRBC KO	1:4	Donor 14	JOVI_CD28STK_CD28TM_28z
**7F**	Jurkat TRBC KO	1:4	Donor 14	mJOVI_Hinge_41bbz
**7G**	Jurkat TRBC KO	1:4	Donor 14	aCD19-CAR
**7H**	Jurkat TRBC KO	1:4	Donor 14	Non-transduced
**8A**	Jurkat TRBC KO	1:4	Donor 15	JOVI_Hinge_41bbz
**8D**	Jurkat TRBC KO	1:4	Donor 15	JOVI_CD8STK_28z
**8E**	Jurkat TRBC KO	1:4	Donor 15	JOVI_CD28STK_CD28TM_28z
**8F**	Jurkat TRBC KO	1:4	Donor 15	mJOVI_Hinge_41bbz
**8G**	Jurkat TRBC KO	1:4	Donor 15	aCD19-CAR
**8H**	Jurkat TRBC KO	1:4	Donor 15	Non-transduced

**9A**	Jurkat TRBC KO	1:4	Donor 16	JOVI_Hinge_41bbz
**9D**	Jurkat TRBC KO	1:4	Donor 16	JOVI_CD8STK_28z
**9E**	Jurkat TRBC KO	1:4	Donor 16	JOVI_CD28STK_CD28TM_28z
**9F**	Jurkat TRBC KO	1:4	Donor 16	mJOVI_Hinge_41bbz
**9G**	Jurkat TRBC KO	1:4	Donor 16	aCD19-CAR
**9H**	Jurkat TRBC KO	1:4	Donor 16	Non-transduced
**10A**	N/A	N/A	N/A	N/A
**10B**	N/A	N/A	N/A	N/A
**10C**	N/A	N/A	N/A	N/A
**10D**	N/A	N/A	N/A	N/A
**10E**	N/A	N/A	N/A	N/A
**10F**	N/A	N/A	N/A	N/A
**10G**	N/A	N/A	N/A	N/A
**10H**	N/A	N/A	N/A	N/A
**11A**	N/A	N/A	N/A	N/A
**11B**	N/A	N/A	N/A	N/A
**11C**	N/A	N/A	N/A	N/A
**11D**	N/A	N/A	N/A	N/A
**11E**	N/A	N/A	N/A	N/A
**11F**	N/A	N/A	N/A	N/A
**11G**	N/A	N/A	N/A	N/A
**11H**	N/A	N/A	N/A	N/A
**12A**	N/A	N/A	N/A	N/A
**12B**	N/A	N/A	N/A	N/A
**12C**	N/A	N/A	N/A	N/A
**12D**	N/A	N/A	N/A	N/A
**12E**	N/A	N/A	N/A	N/A
**12F**	N/A	N/A	N/A	N/A
**12G**	N/A	N/A	N/A	N/A
**12H**	N/A	N/A	N/A	N/A

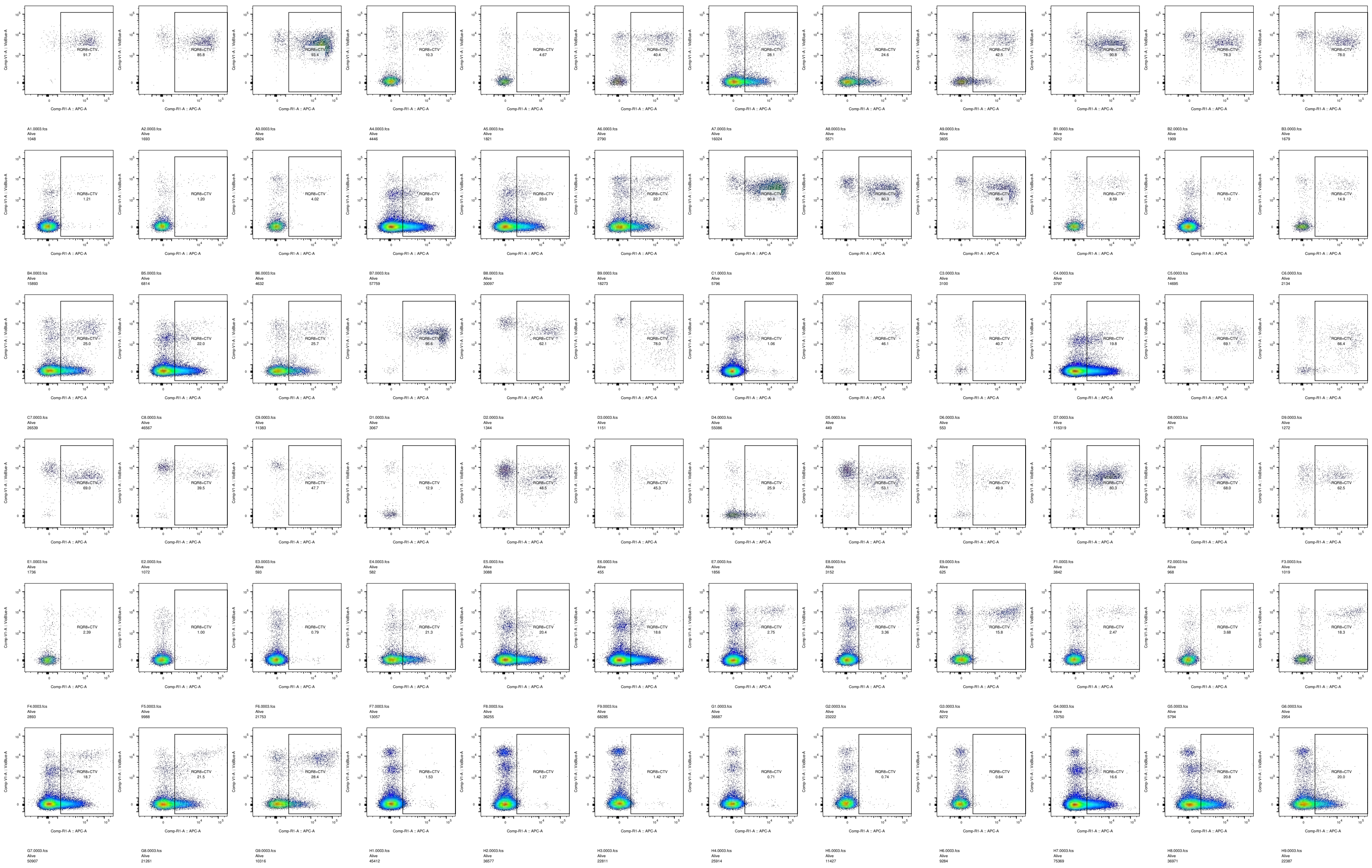
Comp-V1-A :: VioBlue-A
Comp-R1-A :: APC-A
RQR8+CTV 91.7
A1.0003.fcs Alive 1048
RQR8+CTV 85.8
A2.0003.fcs Alive 1693
RQR8+CTV 93.4
A3.0003.fcs Alive 5824
RQR8+CTV 10.3
A4.0003.fcs Alive 4446
RQR8+CTV 4.67
A5.0003.fcs Alive 1821
RQR8+CTV 40.4
A6.0003.fcs Alive 2790
RQR8+CTV 28.1
A7.0003.fcs Alive 16024
RQR8+CTV 24.6
A8.0003.fcs Alive 5571
RQR8+CTV 42.5
A9.0003.fcs Alive 3835
RQR8+CTV 90.8
B1.0003.fcs Alive 3212
RQR8+CTV 78.3
B2.0003.fcs Alive 1909
RQR8+CTV 78.0
B3.0003.fcs Alive 1679
RQR8+CTV 1.21
B4.0003.fcs Alive 15893
RQR8+CTV 1.20
B5.0003.fcs Alive 6814
RQR8+CTV 4.02
B6.0003.fcs Alive 4632
RQR8+CTV 22.9
B7.0003.fcs Alive 57759
RQR8+CTV 23.0
B8.0003.fcs Alive 30097
RQR8+CTV 22.7
B9.0003.fcs Alive 18273
RQR8+CTV 90.8
C1.0003.fcs Alive 5796
RQR8+CTV 80.3
C2.0003.fcs Alive 3997
RQR8+CTV 85.6
C3.0003.fcs Alive 3100
RQR8+CTV 8.59
C4.0003.fcs Alive 3797
RQR8+CTV 1.12
C5.0003.fcs Alive 14695
RQR8+CTV 14.9
C6.0003.fcs Alive 2134
RQR8+CTV 25.0
C7.0003.fcs Alive 26539
RQR8+CTV 22.0
C8.0003.fcs Alive 46567
RQR8+CTV 25.7
C9.0003.fcs Alive 11383
RQR8+CTV 95.6
D1.0003.fcs Alive 3067
RQR8+CTV 62.1
D2.0003.fcs Alive 1344
RQR8+CTV 78.0
D3.0003.fcs Alive 1151
RQR8+CTV 1.06
D4.0003.fcs Alive 55086
RQR8+CTV 46.1
D5.0003.fcs Alive 449
RQR8+CTV 40.7
D6.0003.fcs Alive 553
RQR8+CTV 19.8
D7.0003.fcs Alive 115319
RQR8+CTV 69.1
D8.0003.fcs Alive 871
RQR8+CTV 66.4
D9.0003.fcs Alive 1272
RQR8+CTV 69.0
E1.0003.fcs Alive 1736
RQR8+CTV 39.5
E2.0003.fcs Alive 1072
RQR8+CTV 47.7
E3.0003.fcs Alive 593
RQR8+CTV 12.9
E4.0003.fcs Alive 582
RQR8+CTV 48.5
E5.0003.fcs Alive 3088
RQR8+CTV 45.3
E6.0003.fcs Alive 455
RQR8+CTV 25.9
E7.0003.fcs Alive 1856
RQR8+CTV 53.1
E8.0003.fcs Alive 3152
RQR8+CTV 49.9
E9.0003.fcs Alive 625
RQR8+CTV 80.3
F1.0003.fcs Alive 3842
RQR8+CTV 68.0
F2.0003.fcs Alive 968
RQR8+CTV 62.5
F3.0003.fcs Alive 1019
RQR8+CTV 2.39
F4.0003.fcs Alive 2893
RQR8+CTV 1.00
F5.0003.fcs Alive 9988
RQR8+CTV 0.79
F6.0003.fcs Alive 21753
RQR8+CTV 21.3
F7.0003.fcs Alive 13057
RQR8+CTV 20.4
F8.0003.fcs Alive 36255
RQR8+CTV 18.6
F9.0003.fcs Alive 68285
RQR8+CTV 2.75
G1.0003.fcs Alive 36687
RQR8+CTV 3.36
G2.0003.fcs Alive 23222
RQR8+CTV 15.8
G3.0003.fcs Alive 8272
RQR8+CTV 2.47
G4.0003.fcs Alive 13750
RQR8+CTV 3.68
G5.0003.fcs Alive 5794
RQR8+CTV 18.3
G6.0003.fcs Alive 2954
RQR8+CTV 18.7
G7.0003.fcs Alive 50907
RQR8+CTV 21.5
G8.0003.fcs Alive 21261
RQR8+CTV 28.4
G9.0003.fcs Alive 10316
RQR8+CTV 1.53
H1.0003.fcs Alive 45412
RQR8+CTV 1.27
H2.0003.fcs Alive 36577
RQR8+CTV 1.42
H3.0003.fcs Alive 22811
RQR8+CTV 0.71
H4.0003.fcs Alive 25914
RQR8+CTV 0.74
H5.0003.fcs Alive 11427
RQR8+CTV 0.64
H6.0003.fcs Alive 9284
RQR8+CTV 16.6
H7.0003.fcs Alive 75369
RQR8+CTV 20.8
H8.0003.fcs Alive 36971
RQR8+CTV 20.0
H9.0003.fcs Alive 22387

Plate23-24_JOVI-Prol_HPB_H9_TAL

**Well**	**Target**	**E:T ratio**	**Donor**	**CAR construct**
**1A**	HPB TRBC1+ve	1:4	Donor 14	JOVI_Hinge_41bbz
**1D**	HPB TRBC1+ve	1:4	Donor 14	JOVI_CD8STK_28z
**1E**	HPB TRBC1+ve	1:4	Donor 14	JOVI_CD28STK_CD28TM_28z
**1G**	HPB TRBC1+ve	1:4	Donor 14	aCD19-CAR
**1H**	HPB TRBC1+ve	1:4	Donor 14	Non-transduced
**2A**	HPB TRBC1+ve	1:4	Donor 15	JOVI_Hinge_41bbz
**2D**	HPB TRBC1+ve	1:4	Donor 15	JOVI_CD8STK_28z
**2E**	HPB TRBC1+ve	1:4	Donor 15	JOVI_CD28STK_CD28TM_28z
**2G**	HPB TRBC1+ve	1:4	Donor 15	aCD19-CAR
**2H**	HPB TRBC1+ve	1:4	Donor 15	Non-transduced
**3A**	HPB TRBC1+ve	1:4	Donor 16	JOVI_Hinge_41bbz
**3D**	HPB TRBC1+ve	1:4	Donor 16	JOVI_CD8STK_28z
**3E**	HPB TRBC1+ve	1:4	Donor 16	JOVI_CD28STK_CD28TM_28z
**3G**	HPB TRBC1+ve	1:4	Donor 16	aCD19-CAR
**3H**	HPB TRBC1+ve	1:4	Donor 16	Non-transduced
**4A**	HPB TRBC2+ve	1:4	Donor 14	JOVI_Hinge_41bbz
**4D**	HPB TRBC2+ve	1:4	Donor 14	JOVI_CD8STK_28z
**4E**	HPB TRBC2+ve	1:4	Donor 14	JOVI_CD28STK_CD28TM_28z
**4G**	HPB TRBC2+ve	1:4	Donor 14	aCD19-CAR
**4H**	HPB TRBC2+ve	1:4	Donor 14	Non-transduced
**5A**	HPB TRBC2+ve	1:4	Donor 15	JOVI_Hinge_41bbz
**5D**	HPB TRBC2+ve	1:4	Donor 15	JOVI_CD8STK_28z
**5E**	HPB TRBC2+ve	1:4	Donor 15	JOVI_CD28STK_CD28TM_28z
**5G**	HPB TRBC2+ve	1:4	Donor 15	aCD19-CAR
**5H**	HPB TRBC2+ve	1:4	Donor 15	Non-transduced
**6A**	HPB TRBC2+ve	1:4	Donor 16	JOVI_Hinge_41bbz
**6D**	HPB TRBC2+ve	1:4	Donor 16	JOVI_CD8STK_28z
**6E**	HPB TRBC2+ve	1:4	Donor 16	JOVI_CD28STK_CD28TM_28z
**6G**	HPB TRBC2+ve	1:4	Donor 16	aCD19-CAR
**6H**	HPB TRBC2+ve	1:4	Donor 16	Non-transduced
**7A**	H9	1:4	Donor 14	JOVI_Hinge_41bbz
**7D**	H9	1:4	Donor 14	JOVI_CD8STK_28z
**7E**	H9	1:4	Donor 14	JOVI_CD28STK_CD28TM_28z
**7F**	H9	1:4	Donor 14	mJOVI_Hinge_41bbz
**7G**	H9	1:4	Donor 14	aCD19-CAR
**7H**	H9	1:4	Donor 14	Non-transduced
**8A**	H9	1:4	Donor 15	JOVI_Hinge_41bbz
**8D**	H9	1:4	Donor 15	JOVI_CD8STK_28z
**8E**	H9	1:4	Donor 15	JOVI_CD28STK_CD28TM_28z
**8F**	H9	1:4	Donor 15	mJOVI_Hinge_41bbz
**8G**	H9	1:4	Donor 15	aCD19-CAR
**8H**	H9	1:4	Donor 15	Non-transduced
**9A**	H9	1:4	Donor 16	JOVI_Hinge_41bbz
**9D**	H9	1:4	Donor 16	JOVI_CD8STK_28z
**9E**	H9	1:4	Donor 16	JOVI_CD28STK_CD28TM_28z
**9F**	H9	1:4	Donor 16	mJOVI_Hinge_41bbz
**9G**	H9	1:4	Donor 16	aCD19-CAR
**9H**	H9	1:4	Donor 16	Non-transduced

**10A**	T-ALL1	1:4	Donor 14	JOVI_Hinge_41bbz
**10D**	T-ALL1	1:4	Donor 14	JOVI_CD8STK_28z
**10E**	T-ALL1	1:4	Donor 14	JOVI_CD28STK_CD28TM_28z
**10F**	T-ALL1	1:4	Donor 14	mJOVI_Hinge_41bbz
**10G**	T-ALL1	1:4	Donor 14	aCD19-CAR
**10H**	T-ALL1	1:4	Donor 14	Non-transduced
**11A**	T-ALL1	1:4	Donor 15	JOVI_Hinge_41bbz
**11D**	T-ALL1	1:4	Donor 15	JOVI_CD8STK_28z
**11E**	T-ALL1	1:4	Donor 15	JOVI_CD28STK_CD28TM_28z
**11F**	T-ALL1	1:4	Donor 15	mJOVI_Hinge_41bbz
**11G**	T-ALL1	1:4	Donor 15	aCD19-CAR
**11H**	T-ALL1	1:4	Donor 15	Non-transduced
**12A**	T-ALL1	1:4	Donor 16	JOVI_Hinge_41bbz
**12D**	T-ALL1	1:4	Donor 16	JOVI_CD8STK_28z
**12E**	T-ALL1	1:4	Donor 16	JOVI_CD28STK_CD28TM_28z
**12F**	T-ALL1	1:4	Donor 16	mJOVI_Hinge_41bbz
**12G**	T-ALL1	1:4	Donor 16	aCD19-CAR
**12H**	T-ALL1	1:4	Donor 16	Non-transduced

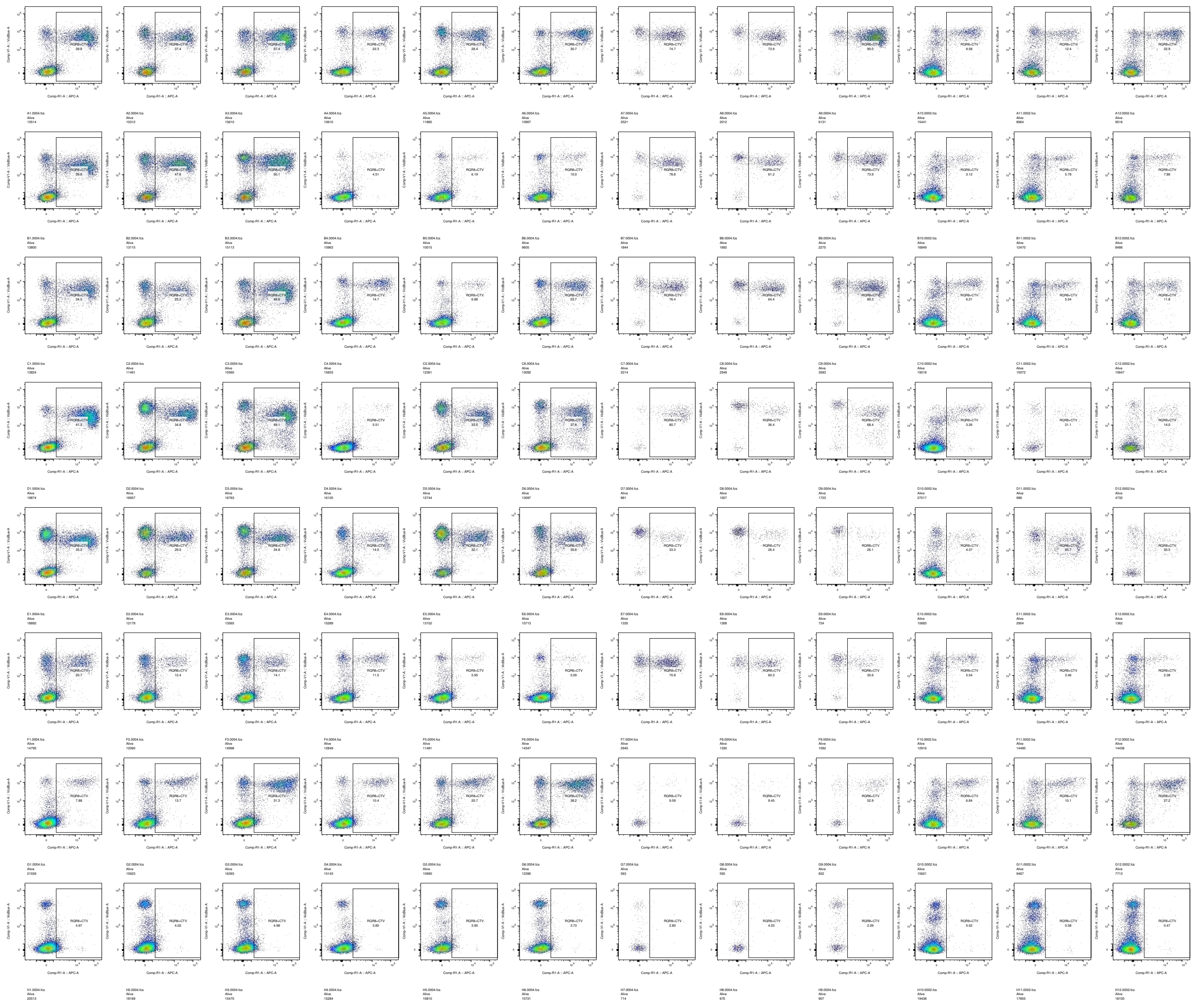

Plate_KFN_vs_PT1

Well	Target	E:T ratio	Donor	CAR construct
1B	T-PLL_Primary Tumour 1_TRBC1+ve	4:1	Donor 10	KFN_Hinge_28z
1D	T-PLL_Primary Tumour 1_TRBC1+ve	4:1	Donor 10	KFN_CD8STK_28z
1E	T-PLL_Primary Tumour 1_TRBC1+ve	4:1	Donor 10	KFN_CD28STK_CD28TM_28z
1F	T-PLL_Primary Tumour 1_TRBC1+ve	4:1	Donor 10	aCD19-CAR
1G	T-PLL_Primary Tumour 1_TRBC1+ve	4:1	Donor 10	Non-transduced
2B	T-PLL_Primary Tumour 1_TRBC1+ve	1:1	Donor 10	KFN_Hinge_28z
2D	T-PLL_Primary Tumour 1_TRBC1+ve	1:1	Donor 10	KFN_CD8STK_28z
2E	T-PLL_Primary Tumour 1_TRBC1+ve	1:1	Donor 10	KFN_CD28STK_CD28TM_28z
2F	T-PLL_Primary Tumour 1_TRBC1+ve	1:1	Donor 10	aCD19-CAR
2G	T-PLL_Primary Tumour 1_TRBC1+ve	1:1	Donor 10	Non-transduced
3B	T-PLL_Primary Tumour 1_TRBC1+ve	1:4	Donor 10	KFN_Hinge_28z
3D	T-PLL_Primary Tumour 1_TRBC1+ve	1:4	Donor 10	KFN_CD8STK_28z
3E	T-PLL_Primary Tumour 1_TRBC1+ve	1:4	Donor 10	KFN_CD28STK_CD28TM_28z
3F	T-PLL_Primary Tumour 1_TRBC1+ve	1:4	Donor 10	aCD19-CAR
3G	T-PLL_Primary Tumour 1_TRBC1+ve	1:4	Donor 10	Non-transduced
4B	T-PLL_Primary Tumour 1_TRBC1+ve	4:1	Donor 11	KFN_Hinge_28z
4D	T-PLL_Primary Tumour 1_TRBC1+ve	4:1	Donor 11	KFN_CD8STK_28z
4E	T-PLL_Primary Tumour 1_TRBC1+ve	4:1	Donor 11	KFN_CD28STK_CD28TM_28z
4F	T-PLL_Primary Tumour 1_TRBC1+ve	4:1	Donor 11	aCD19-CAR
4G	T-PLL_Primary Tumour 1_TRBC1+ve	4:1	Donor 11	Non-transduced
5B	T-PLL_Primary Tumour 1_TRBC1+ve	1:1	Donor 11	KFN_Hinge_28z
5D	T-PLL_Primary Tumour 1_TRBC1+ve	1:1	Donor 11	KFN_CD8STK_28z
5E	T-PLL_Primary Tumour 1_TRBC1+ve	1:1	Donor 11	KFN_CD28STK_CD28TM_28z
5F	T-PLL_Primary Tumour 1_TRBC1+ve	1:1	Donor 11	aCD19-CAR
5G	T-PLL_Primary Tumour 1_TRBC1+ve	1:1	Donor 11	Non-transduced
6B	T-PLL_Primary Tumour 1_TRBC1+ve	1:4	Donor 11	KFN_Hinge_28z
6D	T-PLL_Primary Tumour 1_TRBC1+ve	1:4	Donor 11	KFN_CD8STK_28z
6E	T-PLL_Primary Tumour 1_TRBC1+ve	1:4	Donor 11	KFN_CD28STK_CD28TM_28z
6F	T-PLL_Primary Tumour 1_TRBC1+ve	1:4	Donor 11	aCD19-CAR
6G	T-PLL_Primary Tumour 1_TRBC1+ve	1:4	Donor 11	Non-transduced
7B	T-PLL_Primary Tumour 1_TRBC1+ve	4:1	Donor 12	KFN_Hinge_28z
7D	T-PLL_Primary Tumour 1_TRBC1+ve	4:1	Donor 12	KFN_CD8STK_28z
7E	T-PLL_Primary Tumour 1_TRBC1+ve	4:1	Donor 12	KFN_CD28STK_CD28TM_28z
7F	T-PLL_Primary Tumour 1_TRBC1+ve	4:1	Donor 12	aCD19-CAR
7G	T-PLL_Primary Tumour 1_TRBC1+ve	4:1	Donor 12	Non-transduced
8B	T-PLL_Primary Tumour 1_TRBC1+ve	1:1	Donor 12	KFN_Hinge_28z
8D	T-PLL_Primary Tumour 1_TRBC1+ve	1:1	Donor 12	KFN_CD8STK_28z
8E	T-PLL_Primary Tumour 1_TRBC1+ve	1:1	Donor 12	KFN_CD28STK_CD28TM_28z
8F	T-PLL_Primary Tumour 1_TRBC1+ve	1:1	Donor 12	aCD19-CAR
8G	T-PLL_Primary Tumour 1_TRBC1+ve	1:1	Donor 12	Non-transduced
9B	T-PLL_Primary Tumour 1_TRBC1+ve	1:4	Donor 12	KFN_Hinge_28z
9D	T-PLL_Primary Tumour 1_TRBC1+ve	1:4	Donor 12	KFN_CD8STK_28z
9E	T-PLL_Primary Tumour 1_TRBC1+ve	1:4	Donor 12	KFN_CD28STK_CD28TM_28z
9F	T-PLL_Primary Tumour 1_TRBC1+ve	1:4	Donor 12	aCD19-CAR
9G	T-PLL_Primary Tumour 1_TRBC1+ve	1:4	Donor 12	Non-transduced
10B	T-PLL_Primary Tumour 1_TRBC1+ve	4:1	Donor 13	KFN_Hinge_28z
10D	T-PLL_Primary Tumour 1_TRBC1+ve	4:1	Donor 13	KFN_CD8STK_28z
10E	T-PLL_Primary Tumour 1_TRBC1+ve	4:1	Donor 13	KFN_CD28STK_CD28TM_28z

**10F**	T-PLL_Primary Tumour 1_TRBC1+ve	4:1	Donor 13	aCD19-CAR
**10G**	T-PLL_Primary Tumour 1_TRBC1+ve	4:1	Donor 13	Non-transduced
**11B**	T-PLL_Primary Tumour 1_TRBC1+ve	1:1	Donor 13	KFN_Hinge_28z
**11D**	T-PLL_Primary Tumour 1_TRBC1+ve	1:1	Donor 13	KFN_CD8STK_28z
**11E**	T-PLL_Primary Tumour 1_TRBC1+ve	1:1	Donor 13	KFN_CD28STK_CD28TM_28z
**11F**	T-PLL_Primary Tumour 1_TRBC1+ve	1:1	Donor 13	aCD19-CAR
**11G**	T-PLL_Primary Tumour 1_TRBC1+ve	1:1	Donor 13	Non-transduced
**12B**	T-PLL_Primary Tumour 1_TRBC1+ve	1:4	Donor 13	KFN_Hinge_28z
**12D**	T-PLL_Primary Tumour 1_TRBC1+ve	1:4	Donor 13	KFN_CD8STK_28z
**12E**	T-PLL_Primary Tumour 1_TRBC1+ve	1:4	Donor 13	KFN_CD28STK_CD28TM_28z
**12F**	T-PLL_Primary Tumour 1_TRBC1+ve	1:4	Donor 13	aCD19-CAR
**12G**	T-PLL_Primary Tumour 1_TRBC1+ve	1:4	Donor 13	Non-transduced

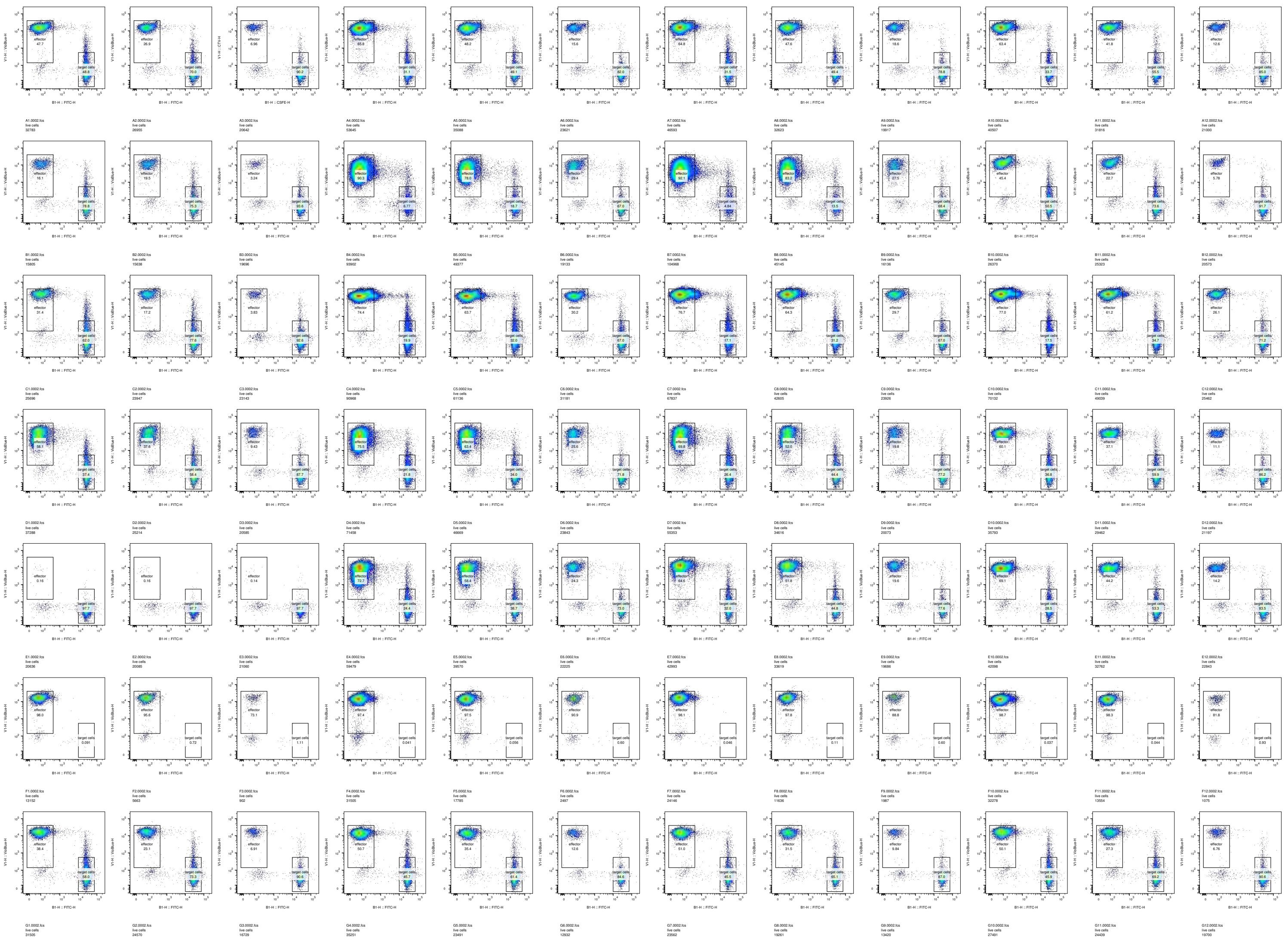

Plate_KFN_vs_PT2

Well	Target	E:T ratio	Donor	CAR construct
**1B**	T-PLL_Primary Tumour 2_TRBC2+ve	4:1	Donor 10	KFN_Hinge_28z
**1D**	T-PLL_Primary Tumour 2_TRBC2+ve	4:1	Donor 10	KFN_CD8STK_28z
**1E**	T-PLL_Primary Tumour 2_TRBC2+ve	4:1	Donor 10	KFN_CD28STK_CD28TM_28z
**1F**	T-PLL_Primary Tumour 2_TRBC2+ve	4:1	Donor 10	aCD19-CAR
**1G**	T-PLL_Primary Tumour 2_TRBC2+ve	4:1	Donor 10	Non-transduced
**2B**	T-PLL_Primary Tumour 2_TRBC2+ve	1:1	Donor 10	KFN_Hinge_28z
**2D**	T-PLL_Primary Tumour 2_TRBC2+ve	1:1	Donor 10	KFN_CD8STK_28z
**2E**	T-PLL_Primary Tumour 2_TRBC2+ve	1:1	Donor 10	KFN_CD28STK_CD28TM_28z
**2F**	T-PLL_Primary Tumour 2_TRBC2+ve	1:1	Donor 10	aCD19-CAR
**2G**	T-PLL_Primary Tumour 2_TRBC2+ve	1:1	Donor 10	Non-transduced
**3B**	T-PLL_Primary Tumour 2_TRBC2+ve	1:4	Donor 10	KFN_Hinge_28z
**3D**	T-PLL_Primary Tumour 2_TRBC2+ve	1:4	Donor 10	KFN_CD8STK_28z
**3E**	T-PLL_Primary Tumour 2_TRBC2+ve	1:4	Donor 10	KFN_CD28STK_CD28TM_28z
**3F**	T-PLL_Primary Tumour 2_TRBC2+ve	1:4	Donor 10	aCD19-CAR
**3G**	T-PLL_Primary Tumour 2_TRBC2+ve	1:4	Donor 10	Non-transduced
**4B**	T-PLL_Primary Tumour 2_TRBC2+ve	4:1	Donor 11	KFN_Hinge_28z
**4D**	T-PLL_Primary Tumour 2_TRBC2+ve	4:1	Donor 11	KFN_CD8STK_28z
**4E**	T-PLL_Primary Tumour 2_TRBC2+ve	4:1	Donor 11	KFN_CD28STK_CD28TM_28z
**4F**	T-PLL_Primary Tumour 2_TRBC2+ve	4:1	Donor 11	aCD19-CAR
**4G**	T-PLL_Primary Tumour 2_TRBC2+ve	4:1	Donor 11	Non-transduced
**5B**	T-PLL_Primary Tumour 2_TRBC2+ve	1:1	Donor 11	KFN_Hinge_28z
**5D**	T-PLL_Primary Tumour 2_TRBC2+ve	1:1	Donor 11	KFN_CD8STK_28z
**5E**	T-PLL_Primary Tumour 2_TRBC2+ve	1:1	Donor 11	KFN_CD28STK_CD28TM_28z
**5F**	T-PLL_Primary Tumour 2_TRBC2+ve	1:1	Donor 11	aCD19-CAR
**5G**	T-PLL_Primary Tumour 2_TRBC2+ve	1:1	Donor 11	Non-transduced
**6B**	T-PLL_Primary Tumour 2_TRBC2+ve	1:4	Donor 11	KFN_Hinge_28z
**6D**	T-PLL_Primary Tumour 2_TRBC2+ve	1:4	Donor 11	KFN_CD8STK_28z
**6E**	T-PLL_Primary Tumour 2_TRBC2+ve	1:4	Donor 11	KFN_CD28STK_CD28TM_28z
**6F**	T-PLL_Primary Tumour 2_TRBC2+ve	1:4	Donor 11	aCD19-CAR
**6G**	T-PLL_Primary Tumour 2_TRBC2+ve	1:4	Donor 11	Non-transduced
**7B**	T-PLL_Primary Tumour 2_TRBC2+ve	4:1	Donor 12	KFN_Hinge_28z
**7D**	T-PLL_Primary Tumour 2_TRBC2+ve	4:1	Donor 12	KFN_CD8STK_28z
**7E**	T-PLL_Primary Tumour 2_TRBC2+ve	4:1	Donor 12	KFN_CD28STK_CD28TM_28z
**7F**	T-PLL_Primary Tumour 2_TRBC2+ve	4:1	Donor 12	aCD19-CAR
**7G**	T-PLL_Primary Tumour 2_TRBC2+ve	4:1	Donor 12	Non-transduced
**8B**	T-PLL_Primary Tumour 2_TRBC2+ve	1:1	Donor 12	KFN_Hinge_28z
**8D**	T-PLL_Primary Tumour 2_TRBC2+ve	1:1	Donor 12	KFN_CD8STK_28z
**8E**	T-PLL_Primary Tumour 2_TRBC2+ve	1:1	Donor 12	KFN_CD28STK_CD28TM_28z
**8F**	T-PLL_Primary Tumour 2_TRBC2+ve	1:1	Donor 12	aCD19-CAR
**8G**	T-PLL_Primary Tumour 2_TRBC2+ve	1:1	Donor 12	Non-transduced
**9B**	T-PLL_Primary Tumour 2_TRBC2+ve	1:4	Donor 12	KFN_Hinge_28z
**9D**	T-PLL_Primary Tumour 2_TRBC2+ve	1:4	Donor 12	KFN_CD8STK_28z
**9E**	T-PLL_Primary Tumour 2_TRBC2+ve	1:4	Donor 12	KFN_CD28STK_CD28TM_28z
**9F**	T-PLL_Primary Tumour 2_TRBC2+ve	1:4	Donor 12	aCD19-CAR
**9G**	T-PLL_Primary Tumour 2_TRBC2+ve	1:4	Donor 12	Non-transduced
**10B**	T-PLL_Primary Tumour 2_TRBC2+ve	4:1	Donor 13	KFN_Hinge_28z
**10D**	T-PLL_Primary Tumour 2_TRBC2+ve	4:1	Donor 13	KFN_CD8STK_28z
**10E**	T-PLL_Primary Tumour 2_TRBC2+ve	4:1	Donor 13	KFN_CD28STK_CD28TM_28z

**10F**	T-PLL_Primary Tumour 2_TRBC2+ve	4:1	Donor 13	aCD19-CAR
**10G**	T-PLL_Primary Tumour 2_TRBC2+ve	4:1	Donor 13	Non-transduced
**11B**	T-PLL_Primary Tumour 2_TRBC2+ve	1:1	Donor 13	KFN_Hinge_28z
**11D**	T-PLL_Primary Tumour 2_TRBC2+ve	1:1	Donor 13	KFN_CD8STK_28z
**11E**	T-PLL_Primary Tumour 2_TRBC2+ve	1:1	Donor 13	KFN_CD28STK_CD28TM_28z
**11F**	T-PLL_Primary Tumour 2_TRBC2+ve	1:1	Donor 13	aCD19-CAR
**11G**	T-PLL_Primary Tumour 2_TRBC2+ve	1:1	Donor 13	Non-transduced
**12B**	T-PLL_Primary Tumour 2_TRBC2+ve	1:4	Donor 13	KFN_Hinge_28z
**12D**	T-PLL_Primary Tumour 2_TRBC2+ve	1:4	Donor 13	KFN_CD8STK_28z
**12E**	T-PLL_Primary Tumour 2_TRBC2+ve	1:4	Donor 13	KFN_CD28STK_CD28TM_28z
**12F**	T-PLL_Primary Tumour 2_TRBC2+ve	1:4	Donor 13	aCD19-CAR
**12G**	T-PLL_Primary Tumour 2_TRBC2+ve	1:4	Donor 13	Non-transduced

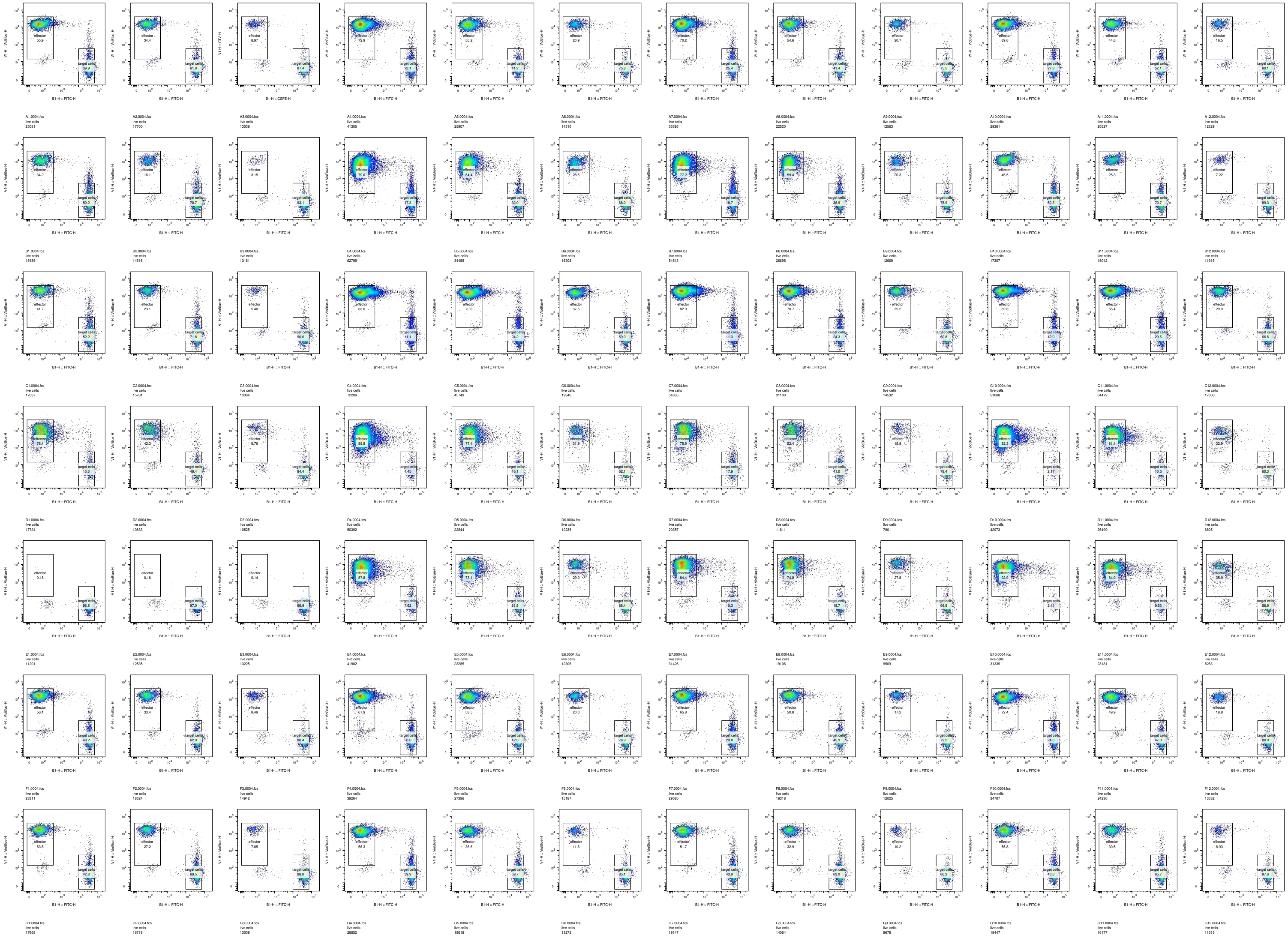
A1.0004.fcs
live cells
25081
A2.0004.fcs
live cells
17700
A3.0004.fcs
live cells
13038
A4.0004.fcs
live cells
41305
A5.0004.fcs
live cells
25907
A6.0004.fcs
live cells
14310
A7.0004.fcs
live cells
35395
A8.0004.fcs
live cells
22520
A9.0004.fcs
live cells
12563
A10.0004.fcs
live cells
29361
A11.0004.fcs
live cells
20527
A12.0004.fcs
live cells
12329
B1.0004.fcs
live cells
15485
B2.0004.fcs
live cells
14516
B3.0004.fcs
live cells
13161
B4.0004.fcs
live cells
62795
B5.0004.fcs
live cells
34485
B6.0004.fcs
live cells
16308
B7.0004.fcs
live cells
54513
B8.0004.fcs
live cells
26698
B9.0004.fcs
live cells
12669
B10.0004.fcs
live cells
17307
B11.0004.fcs
live cells
15042
B12.0004.fcs
live cells
11915
C1.0004.fcs
live cells
17637
C2.0004.fcs
live cells
15781
C3.0004.fcs
live cells
13384
C4.0004.fcs
live cells
72206
C5.0004.fcs
live cells
45749
C6.0004.fcs
live cells
19346
C7.0004.fcs
live cells
54665
C8.0004.fcs
live cells
31100
C9.0004.fcs
live cells
14532
C10.0004.fcs
live cells
51068
C11.0004.fcs
live cells
34479
C12.0004.fcs
live cells
17306
D1.0004.fcs
live cells
17724
D2.0004.fcs
live cells
10833
D3.0004.fcs
live cells
10525
D4.0004.fcs
live cells
50392
D5.0004.fcs
live cells
22844
D6.0004.fcs
live cells
10236
D7.0004.fcs
live cells
20357
D8.0004.fcs
live cells
11611
D9.0004.fcs
live cells
7901
D10.0004.fcs
live cells
42973
D11.0004.fcs
live cells
25499
D12.0004.fcs
live cells
6805
E1.0004.fcs
live cells
11201
E2.0004.fcs
live cells
12535
E3.0004.fcs
live cells
13225
E4.0004.fcs
live cells
41902
E5.0004.fcs
live cells
23285
E6.0004.fcs
live cells
12306
E7.0004.fcs
live cells
31426
E8.0004.fcs
live cells
19106
E9.0004.fcs
live cells
9509
E10.0004.fcs
live cells
31339
E11.0004.fcs
live cells
23131
E12.0004.fcs
live cells
8263
F1.0004.fcs
live cells
23311
F2.0004.fcs
live cells
18024
F3.0004.fcs
live cells
14942
F4.0004.fcs
live cells
39264
F5.0004.fcs
live cells
27396
F6.0004.fcs
live cells
15187
F7.0004.fcs
live cells
29085
F8.0004.fcs
live cells
19318
F9.0004.fcs
live cells
12025
F10.0004.fcs
live cells
34707
F11.0004.fcs
live cells
24230
F12.0004.fcs
live cells
13532
G1.0004.fcs
live cells
17668
G2.0004.fcs
live cells
16718
G3.0004.fcs
live cells
13008
G4.0004.fcs
live cells
26852
G5.0004.fcs
live cells
18618
G6.0004.fcs
live cells
13273
G7.0004.fcs
live cells
19147
G8.0004.fcs
live cells
14064
G9.0004.fcs
live cells
9678
G10.0004.fcs
live cells
18447
G11.0004.fcs
live cells
16177
G12.0004.fcs
live cells
11513

Plate_JOVI_vs_PT1

Well	Target	E:T ratio	Donor
1A	T-PLL_Primary Tumour 1_TRBC1+ve	4:1	Donor 10
1D	T-PLL_Primary Tumour 1_TRBC1+ve	4:1	Donor 10
1E	T-PLL_Primary Tumour 1_TRBC1+ve	4:1	Donor 10
1G	T-PLL_Primary Tumour 1_TRBC1+ve	4:1	Donor 10
1H	T-PLL_Primary Tumour 1_TRBC1+ve	4:1	Donor 10
2A	T-PLL_Primary Tumour 1_TRBC1+ve	1:1	Donor 10
2D	T-PLL_Primary Tumour 1_TRBC1+ve	1:1	Donor 10
2E	T-PLL_Primary Tumour 1_TRBC1+ve	1:1	Donor 10
2G	T-PLL_Primary Tumour 1_TRBC1+ve	1:1	Donor 10
2H	T-PLL_Primary Tumour 1_TRBC1+ve	1:1	Donor 10
3A	T-PLL_Primary Tumour 1_TRBC1+ve	1:4	Donor 10
3D	T-PLL_Primary Tumour 1_TRBC1+ve	1:4	Donor 10
3E	T-PLL_Primary Tumour 1_TRBC1+ve	1:4	Donor 10
3G	T-PLL_Primary Tumour 1_TRBC1+ve	1:4	Donor 10
3H	T-PLL_Primary Tumour 1_TRBC1+ve	1:4	Donor 10
4A	T-PLL_Primary Tumour 1_TRBC1+ve	4:1	Donor 11
4D	T-PLL_Primary Tumour 1_TRBC1+ve	4:1	Donor 11
4E	T-PLL_Primary Tumour 1_TRBC1+ve	4:1	Donor 11
4G	T-PLL_Primary Tumour 1_TRBC1+ve	4:1	Donor 11
4H	T-PLL_Primary Tumour 1_TRBC1+ve	4:1	Donor 11
5A	T-PLL_Primary Tumour 1_TRBC1+ve	1:1	Donor 11
5D	T-PLL_Primary Tumour 1_TRBC1+ve	1:1	Donor 11
5E	T-PLL_Primary Tumour 1_TRBC1+ve	1:1	Donor 11
5G	T-PLL_Primary Tumour 1_TRBC1+ve	1:1	Donor 11
5H	T-PLL_Primary Tumour 1_TRBC1+ve	1:1	Donor 11
6A	T-PLL_Primary Tumour 1_TRBC1+ve	1:4	Donor 11
6D	T-PLL_Primary Tumour 1_TRBC1+ve	1:4	Donor 11
6E	T-PLL_Primary Tumour 1_TRBC1+ve	1:4	Donor 11
6G	T-PLL_Primary Tumour 1_TRBC1+ve	1:4	Donor 11
6H	T-PLL_Primary Tumour 1_TRBC1+ve	1:4	Donor 11
7A	T-PLL_Primary Tumour 1_TRBC1+ve	4:1	Donor 12
7D	T-PLL_Primary Tumour 1_TRBC1+ve	4:1	Donor 12
7E	T-PLL_Primary Tumour 1_TRBC1+ve	4:1	Donor 12
7G	T-PLL_Primary Tumour 1_TRBC1+ve	4:1	Donor 12
7H	T-PLL_Primary Tumour 1_TRBC1+ve	4:1	Donor 12
8A	T-PLL_Primary Tumour 1_TRBC1+ve	1:1	Donor 12
8D	T-PLL_Primary Tumour 1_TRBC1+ve	1:1	Donor 12
8E	T-PLL_Primary Tumour 1_TRBC1+ve	1:1	Donor 12
8G	T-PLL_Primary Tumour 1_TRBC1+ve	1:1	Donor 12
8H	T-PLL_Primary Tumour 1_TRBC1+ve	1:1	Donor 12
9A	T-PLL_Primary Tumour 1_TRBC1+ve	1:4	Donor 12
9D	T-PLL_Primary Tumour 1_TRBC1+ve	1:4	Donor 12
9E	T-PLL_Primary Tumour 1_TRBC1+ve	1:4	Donor 12
9G	T-PLL_Primary Tumour 1_TRBC1+ve	1:4	Donor 12
9H	T-PLL_Primary Tumour 1_TRBC1+ve	1:4	Donor 12
10A	T-PLL_Primary Tumour 1_TRBC1+ve	4:1	Donor 13
10D	T-PLL_Primary Tumour 1_TRBC1+ve	4:1	Donor 13
10E	T-PLL_Primary Tumour 1_TRBC1+ve	4:1	Donor 13

**10G**	T-PLL_Primary Tumour 1_TRBC1+ve	4:1	Donor 13
**10H**	T-PLL_Primary Tumour 1_TRBC1+ve	4:1	Donor 13
**11A**	T-PLL_Primary Tumour 1_TRBC1+ve	1:1	Donor 13
**11D**	T-PLL_Primary Tumour 1_TRBC1+ve	1:1	Donor 13
**11E**	T-PLL_Primary Tumour 1_TRBC1+ve	1:1	Donor 13
**11G**	T-PLL_Primary Tumour 1_TRBC1+ve	1:1	Donor 13
**11H**	T-PLL_Primary Tumour 1_TRBC1+ve	1:1	Donor 13
**12A**	T-PLL_Primary Tumour 1_TRBC1+ve	1:4	Donor 13
**12D**	T-PLL_Primary Tumour 1_TRBC1+ve	1:4	Donor 13
**12E**	T-PLL_Primary Tumour 1_TRBC1+ve	1:4	Donor 13
**12G**	T-PLL_Primary Tumour 1_TRBC1+ve	1:4	Donor 13
**12H**	T-PLL_Primary Tumour 1_TRBC1+ve	1:4	Donor 13

CAR construct
JOVI_Hinge_41bbz
JOVI_CD8STK_28z
JOVI_CD28STK_CD28TM_28z
aCD19-CAR
Non-transduced
JOVI_Hinge_41bbz
JOVI_CD8STK_28z
JOVI_CD28STK_CD28TM_28z
aCD19-CAR
Non-transduced
JOVI_Hinge_41bbz
JOVI_CD8STK_28z
JOVI_CD28STK_CD28TM_28z
aCD19-CAR
Non-transduced
JOVI_Hinge_41bbz
JOVI_CD8STK_28z
JOVI_CD28STK_CD28TM_28z
aCD19-CAR
Non-transduced
JOVI_Hinge_41bbz
JOVI_CD8STK_28z
JOVI_CD28STK_CD28TM_28z
aCD19-CAR
Non-transduced
JOVI_Hinge_41bbz
JOVI_CD8STK_28z
JOVI_CD28STK_CD28TM_28z
aCD19-CAR
Non-transduced
JOVI_Hinge_41bbz
JOVI_CD8STK_28z
JOVI_CD28STK_CD28TM_28z
aCD19-CAR
Non-transduced
JOVI_Hinge_41bbz
JOVI_CD8STK_28z
JOVI_CD28STK_CD28TM_28z
aCD19-CAR
Non-transduced
JOVI_Hinge_41bbz
JOVI_CD8STK_28z
JOVI_CD28STK_CD28TM_28z
aCD19-CAR
Non-transduced
JOVI_Hinge_41bbz
JOVI_CD8STK_28z
JOVI_CD28STK_CD28TM_28z

aCD19-CAR
Non-transduced
JOVI_Hinge_41bbz
JOVI_CD8STK_28z
JOVI_CD28STK_CD28TM_28z
aCD19-CAR
Non-transduced
JOVI_Hinge_41bbz
JOVI_CD8STK_28z
JOVI_CD28STK_CD28TM_28z
aCD19-CAR
Non-transduced

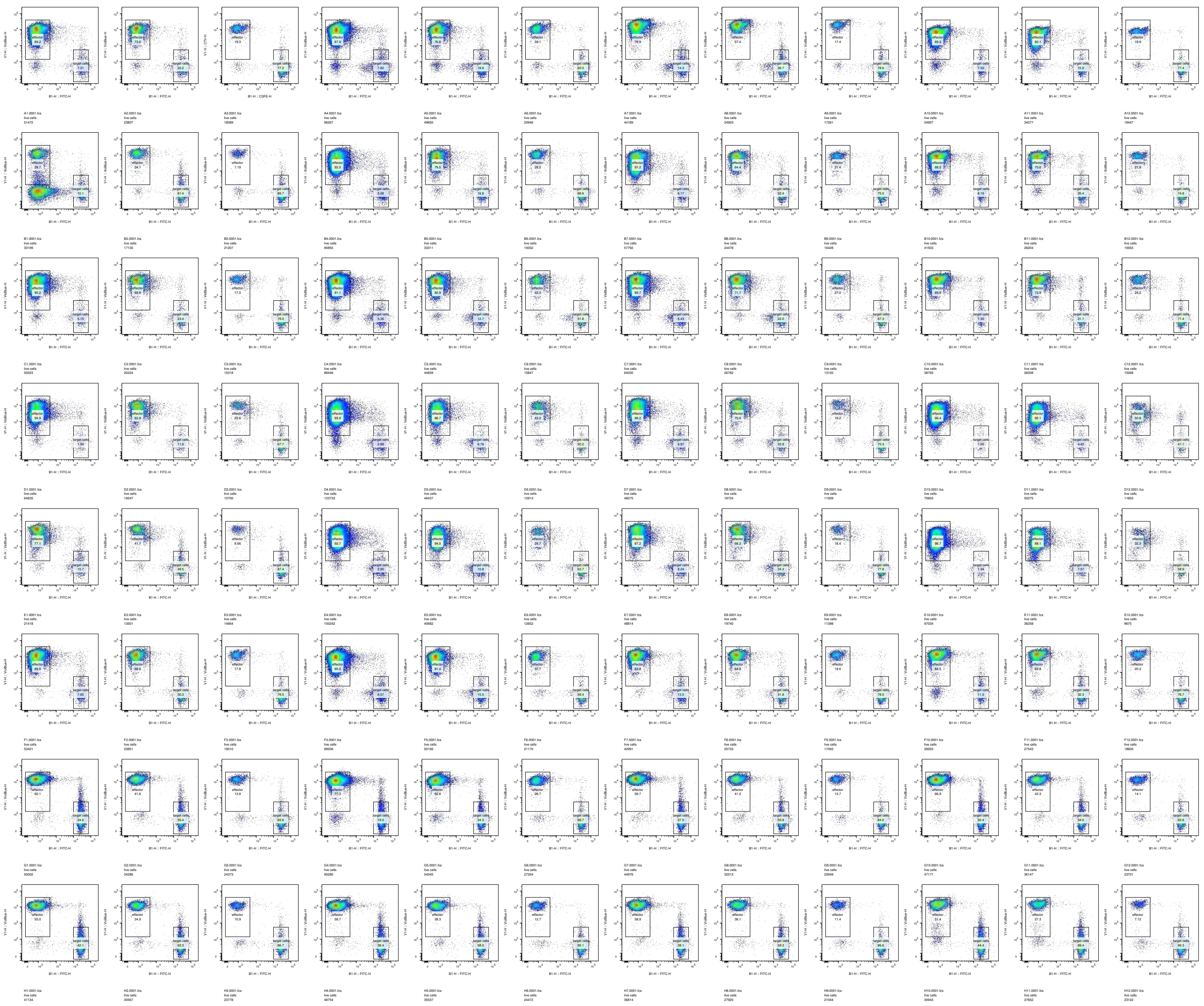

Plate_JOVI_vs_PT2

Well	Target	E:T ratio	Donor
**1A**	T-PLL_Primary Tumour 2_TRBC2+ve	4:1	Donor 10
**1D**	T-PLL_Primary Tumour 2_TRBC2+ve	4:1	Donor 10
**1E**	T-PLL_Primary Tumour 2_TRBC2+ve	4:1	Donor 10
**1G**	T-PLL_Primary Tumour 2_TRBC2+ve	4:1	Donor 10
**1H**	T-PLL_Primary Tumour 2_TRBC2+ve	4:1	Donor 10
**2A**	T-PLL_Primary Tumour 2_TRBC2+ve	1:1	Donor 10
**2D**	T-PLL_Primary Tumour 2_TRBC2+ve	1:1	Donor 10
**2E**	T-PLL_Primary Tumour 2_TRBC2+ve	1:1	Donor 10
**2G**	T-PLL_Primary Tumour 2_TRBC2+ve	1:1	Donor 10
**2H**	T-PLL_Primary Tumour 2_TRBC2+ve	1:1	Donor 10
**3A**	T-PLL_Primary Tumour 2_TRBC2+ve	1:4	Donor 10
**3D**	T-PLL_Primary Tumour 2_TRBC2+ve	1:4	Donor 10
**3E**	T-PLL_Primary Tumour 2_TRBC2+ve	1:4	Donor 10
**3G**	T-PLL_Primary Tumour 2_TRBC2+ve	1:4	Donor 10
**3H**	T-PLL_Primary Tumour 2_TRBC2+ve	1:4	Donor 10
**4A**	T-PLL_Primary Tumour 2_TRBC2+ve	4:1	Donor 11
**4D**	T-PLL_Primary Tumour 2_TRBC2+ve	4:1	Donor 11
**4E**	T-PLL_Primary Tumour 2_TRBC2+ve	4:1	Donor 11
**4G**	T-PLL_Primary Tumour 2_TRBC2+ve	4:1	Donor 11
**4H**	T-PLL_Primary Tumour 2_TRBC2+ve	4:1	Donor 11
**5A**	T-PLL_Primary Tumour 2_TRBC2+ve	1:1	Donor 11
**5D**	T-PLL_Primary Tumour 2_TRBC2+ve	1:1	Donor 11
**5E**	T-PLL_Primary Tumour 2_TRBC2+ve	1:1	Donor 11
**5G**	T-PLL_Primary Tumour 2_TRBC2+ve	1:1	Donor 11
**5H**	T-PLL_Primary Tumour 2_TRBC2+ve	1:1	Donor 11
**6A**	T-PLL_Primary Tumour 2_TRBC2+ve	1:4	Donor 11
**6D**	T-PLL_Primary Tumour 2_TRBC2+ve	1:4	Donor 11
**6E**	T-PLL_Primary Tumour 2_TRBC2+ve	1:4	Donor 11
**6G**	T-PLL_Primary Tumour 2_TRBC2+ve	1:4	Donor 11
**6H**	T-PLL_Primary Tumour 2_TRBC2+ve	1:4	Donor 11
**7A**	T-PLL_Primary Tumour 2_TRBC2+ve	4:1	Donor 12
**7D**	T-PLL_Primary Tumour 2_TRBC2+ve	4:1	Donor 12
**7E**	T-PLL_Primary Tumour 2_TRBC2+ve	4:1	Donor 12
**7G**	T-PLL_Primary Tumour 2_TRBC2+ve	4:1	Donor 12
**7H**	T-PLL_Primary Tumour 2_TRBC2+ve	4:1	Donor 12
**8A**	T-PLL_Primary Tumour 2_TRBC2+ve	1:1	Donor 12
**8D**	T-PLL_Primary Tumour 2_TRBC2+ve	1:1	Donor 12
**8E**	T-PLL_Primary Tumour 2_TRBC2+ve	1:1	Donor 12
**8G**	T-PLL_Primary Tumour 2_TRBC2+ve	1:1	Donor 12
**8H**	T-PLL_Primary Tumour 2_TRBC2+ve	1:1	Donor 12
**9A**	T-PLL_Primary Tumour 2_TRBC2+ve	1:4	Donor 12
**9D**	T-PLL_Primary Tumour 2_TRBC2+ve	1:4	Donor 12
**9E**	T-PLL_Primary Tumour 2_TRBC2+ve	1:4	Donor 12
**9G**	T-PLL_Primary Tumour 2_TRBC2+ve	1:4	Donor 12
**9H**	T-PLL_Primary Tumour 2_TRBC2+ve	1:4	Donor 12
**10A**	T-PLL_Primary Tumour 2_TRBC2+ve	4:1	Donor 13
**10D**	T-PLL_Primary Tumour 2_TRBC2+ve	4:1	Donor 13
**10E**	T-PLL_Primary Tumour 2_TRBC2+ve	4:1	Donor 13

**10G**	T-PLL_Primary Tumour 2_TRBC2+ve	4:1	Donor 13
**10H**	T-PLL_Primary Tumour 2_TRBC2+ve	4:1	Donor 13
**11A**	T-PLL_Primary Tumour 2_TRBC2+ve	1:1	Donor 13
**11D**	T-PLL_Primary Tumour 2_TRBC2+ve	1:1	Donor 13
**11E**	T-PLL_Primary Tumour 2_TRBC2+ve	1:1	Donor 13
**11G**	T-PLL_Primary Tumour 2_TRBC2+ve	1:1	Donor 13
**11H**	T-PLL_Primary Tumour 2_TRBC2+ve	1:1	Donor 13
**12A**	T-PLL_Primary Tumour 2_TRBC2+ve	1:4	Donor 13
**12D**	T-PLL_Primary Tumour 2_TRBC2+ve	1:4	Donor 13
**12E**	T-PLL_Primary Tumour 2_TRBC2+ve	1:4	Donor 13
**12G**	T-PLL_Primary Tumour 2_TRBC2+ve	1:4	Donor 13
**12H**	T-PLL_Primary Tumour 2_TRBC2+ve	1:4	Donor 13

**CAR construct**
JOVI_Hinge_41bbz
JOVI_CD8STK_28z
JOVI_CD28STK_CD28TM_28z
aCD19-CAR
Non-transduced
JOVI_Hinge_41bbz
JOVI_CD8STK_28z
JOVI_CD28STK_CD28TM_28z
aCD19-CAR
Non-transduced
JOVI_Hinge_41bbz
JOVI_CD8STK_28z
JOVI_CD28STK_CD28TM_28z
aCD19-CAR
Non-transduced
JOVI_Hinge_41bbz
JOVI_CD8STK_28z
JOVI_CD28STK_CD28TM_28z
aCD19-CAR
Non-transduced
JOVI_Hinge_41bbz
JOVI_CD8STK_28z
JOVI_CD28STK_CD28TM_28z
aCD19-CAR
Non-transduced
JOVI_Hinge_41bbz
JOVI_CD8STK_28z
JOVI_CD28STK_CD28TM_28z
aCD19-CAR
Non-transduced
JOVI_Hinge_41bbz
JOVI_CD8STK_28z
JOVI_CD28STK_CD28TM_28z
aCD19-CAR
Non-transduced
JOVI_Hinge_41bbz
JOVI_CD8STK_28z
JOVI_CD28STK_CD28TM_28z
aCD19-CAR
Non-transduced
JOVI_Hinge_41bbz
JOVI_CD8STK_28z
JOVI_CD28STK_CD28TM_28z
aCD19-CAR
Non-transduced
JOVI_Hinge_41bbz
JOVI_CD8STK_28z
JOVI_CD28STK_CD28TM_28z

aCD19-CAR
Non-transduced
JOVI_Hinge_41bbz
JOVI_CD8STK_28z
JOVI_CD28STK_CD28TM_28z
aCD19-CAR
Non-transduced
JOVI_Hinge_41bbz
JOVI_CD8STK_28z
JOVI_CD28STK_CD28TM_28z
aCD19-CAR
Non-transduced

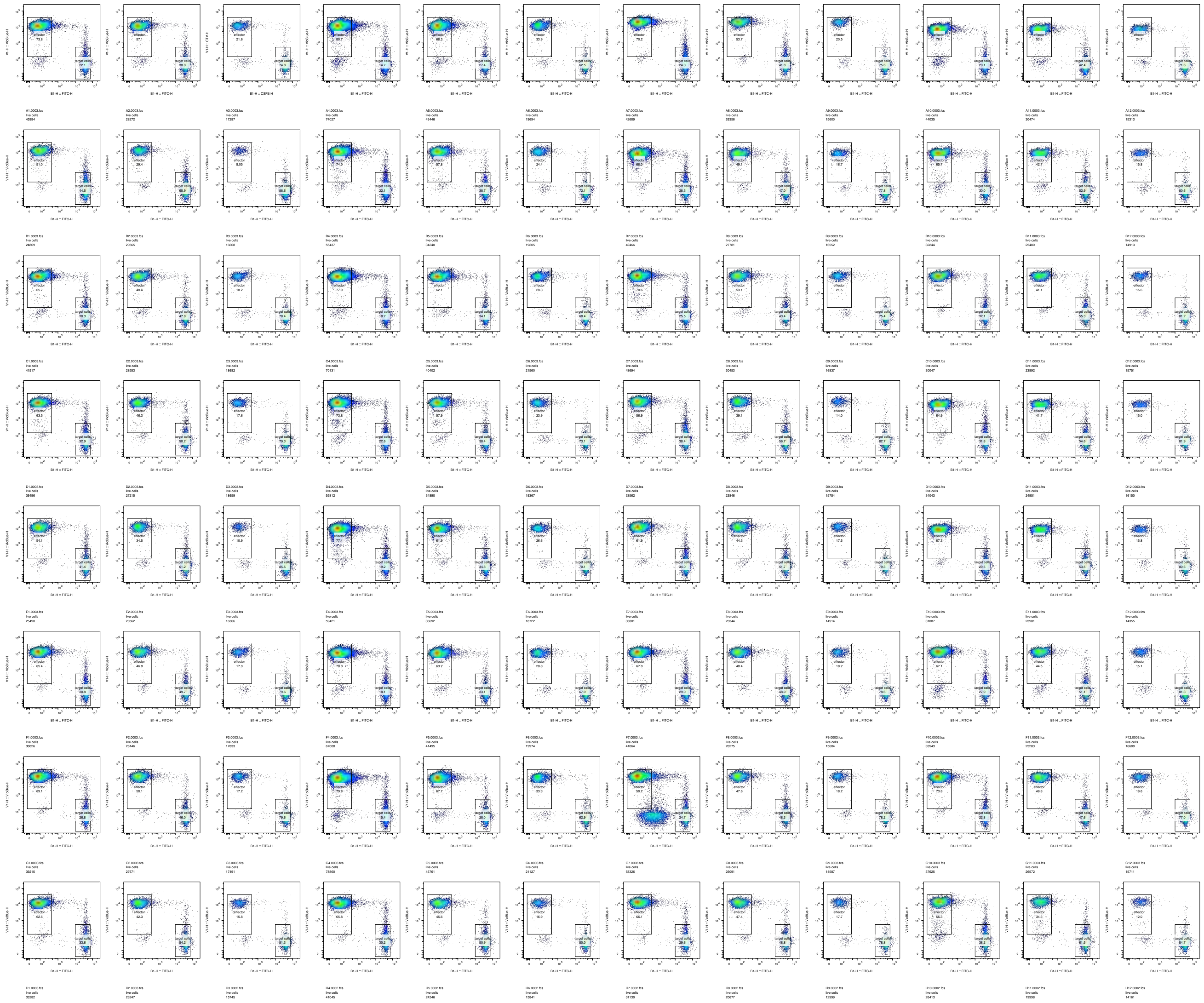

Reverse_Kill_JOVI_vs_T1

Well	Target	E:T ratio	Donor	CAR construct
1A	Donor matched TRBC1+ve PBMCs	4:1	Donor 10	JOVI_Hinge_41bbz
1D	Donor matched TRBC1+ve PBMCs	4:1	Donor 10	JOVI_CD8STK_28z
1E	Donor matched TRBC1+ve PBMCs	4:1	Donor 10	JOVI_CD28STK_CD28TM_28z
1F	Donor matched TRBC1+ve PBMCs	4:1	Donor 10	mJOVI_Hinge_41bbz
1G	Donor matched TRBC1+ve PBMCs	4:1	Donor 10	aCD19-CAR
1H	Donor matched TRBC1+ve PBMCs	4:1	Donor 10	Non-transduced
2A	Donor matched TRBC1+ve PBMCs	1:1	Donor 10	JOVI_Hinge_41bbz
2D	Donor matched TRBC1+ve PBMCs	1:1	Donor 10	JOVI_CD8STK_28z
2E	Donor matched TRBC1+ve PBMCs	1:1	Donor 10	JOVI_CD28STK_CD28TM_28z
2F	Donor matched TRBC1+ve PBMCs	1:1	Donor 10	mJOVI_Hinge_41bbz
2G	Donor matched TRBC1+ve PBMCs	1:1	Donor 10	aCD19-CAR
2H	Donor matched TRBC1+ve PBMCs	1:1	Donor 10	Non-transduced
3A	Donor matched TRBC1+ve PBMCs	1:4	Donor 10	JOVI_Hinge_41bbz
3D	Donor matched TRBC1+ve PBMCs	1:4	Donor 10	JOVI_CD8STK_28z
3E	Donor matched TRBC1+ve PBMCs	1:4	Donor 10	JOVI_CD28STK_CD28TM_28z
3F	Donor matched TRBC1+ve PBMCs	1:4	Donor 10	mJOVI_Hinge_41bbz
3G	Donor matched TRBC1+ve PBMCs	1:4	Donor 10	aCD19-CAR
3H	Donor matched TRBC1+ve PBMCs	1:4	Donor 10	Non-transduced
4A	Donor matched TRBC1+ve PBMCs	4:1	Donor 11	JOVI_Hinge_41bbz
4D	Donor matched TRBC1+ve PBMCs	4:1	Donor 11	JOVI_CD8STK_28z
4E	Donor matched TRBC1+ve PBMCs	4:1	Donor 11	JOVI_CD28STK_CD28TM_28z
4F	Donor matched TRBC1+ve PBMCs	4:1	Donor 11	mJOVI_Hinge_41bbz
4G	Donor matched TRBC1+ve PBMCs	4:1	Donor 11	aCD19-CAR
4H	Donor matched TRBC1+ve PBMCs	4:1	Donor 11	Non-transduced
5A	Donor matched TRBC1+ve PBMCs	1:1	Donor 11	JOVI_Hinge_41bbz
5D	Donor matched TRBC1+ve PBMCs	1:1	Donor 11	JOVI_CD8STK_28z
5E	Donor matched TRBC1+ve PBMCs	1:1	Donor 11	JOVI_CD28STK_CD28TM_28z
5F	Donor matched TRBC1+ve PBMCs	1:1	Donor 11	mJOVI_Hinge_41bbz
5G	Donor matched TRBC1+ve PBMCs	1:1	Donor 11	aCD19-CAR
5H	Donor matched TRBC1+ve PBMCs	1:1	Donor 11	Non-transduced
6A	Donor matched TRBC1+ve PBMCs	1:4	Donor 11	JOVI_Hinge_41bbz
6D	Donor matched TRBC1+ve PBMCs	1:4	Donor 11	JOVI_CD8STK_28z
6E	Donor matched TRBC1+ve PBMCs	1:4	Donor 11	JOVI_CD28STK_CD28TM_28z
6F	Donor matched TRBC1+ve PBMCs	1:4	Donor 11	mJOVI_Hinge_41bbz
6G	Donor matched TRBC1+ve PBMCs	1:4	Donor 11	aCD19-CAR
6H	Donor matched TRBC1+ve PBMCs	1:4	Donor 11	Non-transduced
7A	Donor matched TRBC1+ve PBMCs	4:1	Donor 12	JOVI_Hinge_41bbz
7D	Donor matched TRBC1+ve PBMCs	4:1	Donor 12	JOVI_CD8STK_28z
7E	Donor matched TRBC1+ve PBMCs	4:1	Donor 12	JOVI_CD28STK_CD28TM_28z
7F	Donor matched TRBC1+ve PBMCs	4:1	Donor 12	mJOVI_Hinge_41bbz
7G	Donor matched TRBC1+ve PBMCs	4:1	Donor 12	aCD19-CAR
7H	Donor matched TRBC1+ve PBMCs	4:1	Donor 12	Non-transduced
8A	Donor matched TRBC1+ve PBMCs	1:1	Donor 12	JOVI_Hinge_41bbz
8D	Donor matched TRBC1+ve PBMCs	1:1	Donor 12	JOVI_CD8STK_28z
8E	Donor matched TRBC1+ve PBMCs	1:1	Donor 12	JOVI_CD28STK_CD28TM_28z
8F	Donor matched TRBC1+ve PBMCs	1:1	Donor 12	mJOVI_Hinge_41bbz
8G	Donor matched TRBC1+ve PBMCs	1:1	Donor 12	aCD19-CAR
8H	Donor matched TRBC1+ve PBMCs	1:1	Donor 12	Non-transduced

**9A**	Donor matched TRBC1+ve PBMCs	1:4	Donor 12	JOVI_Hinge_41bbz
**9D**	Donor matched TRBC1+ve PBMCs	1:4	Donor 12	JOVI_CD8STK_28z
**9E**	Donor matched TRBC1+ve PBMCs	1:4	Donor 12	JOVI_CD28STK_CD28TM_28z
**9F**	Donor matched TRBC1+ve PBMCs	1:4	Donor 12	mJOVI_Hinge_41bbz
**9G**	Donor matched TRBC1+ve PBMCs	1:4	Donor 12	aCD19-CAR
**9H**	Donor matched TRBC1+ve PBMCs	1:4	Donor 12	Non-transduced
**10A**	Donor matched TRBC1+ve PBMCs	4:1	Donor 13	JOVI_Hinge_41bbz
**10D**	Donor matched TRBC1+ve PBMCs	4:1	Donor 13	JOVI_CD8STK_28z
**10E**	Donor matched TRBC1+ve PBMCs	4:1	Donor 13	JOVI_CD28STK_CD28TM_28z
**10F**	Donor matched TRBC1+ve PBMCs	4:1	Donor 13	mJOVI_Hinge_41bbz
**10G**	Donor matched TRBC1+ve PBMCs	4:1	Donor 13	aCD19-CAR
**10H**	Donor matched TRBC1+ve PBMCs	4:1	Donor 13	Non-transduced
**11A**	Donor matched TRBC1+ve PBMCs	1:1	Donor 13	JOVI_Hinge_41bbz
**11D**	Donor matched TRBC1+ve PBMCs	1:1	Donor 13	JOVI_CD8STK_28z
**11E**	Donor matched TRBC1+ve PBMCs	1:1	Donor 13	JOVI_CD28STK_CD28TM_28z
**11F**	Donor matched TRBC1+ve PBMCs	1:1	Donor 13	mJOVI_Hinge_41bbz
**11G**	Donor matched TRBC1+ve PBMCs	1:1	Donor 13	aCD19-CAR
**11H**	Donor matched TRBC1+ve PBMCs	1:1	Donor 13	Non-transduced
**12A**	Donor matched TRBC1+ve PBMCs	1:4	Donor 13	JOVI_Hinge_41bbz
**12D**	Donor matched TRBC1+ve PBMCs	1:4	Donor 13	JOVI_CD8STK_28z
**12E**	Donor matched TRBC1+ve PBMCs	1:4	Donor 13	JOVI_CD28STK_CD28TM_28z
**12F**	Donor matched TRBC1+ve PBMCs	1:4	Donor 13	mJOVI_Hinge_41bbz
**12G**	Donor matched TRBC1+ve PBMCs	1:4	Donor 13	aCD19-CAR
**12H**	Donor matched TRBC1+ve PBMCs	1:4	Donor 13	Non-transduced

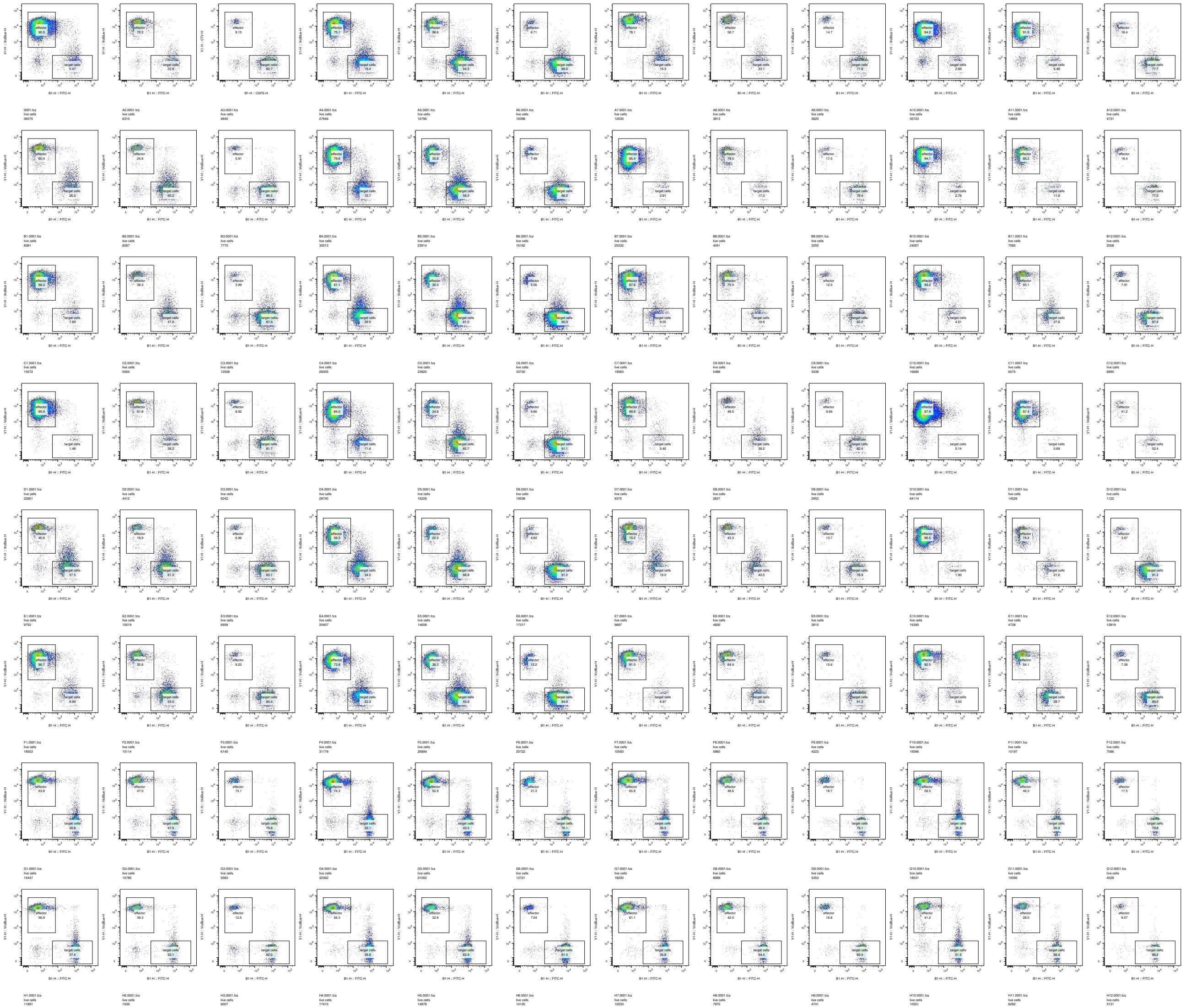

Reverse_Kill_JOVI_vs_T2

Well	Target	E:T ratio	Donor	CAR construct
1A	Donor matched TRBC2+ve PBMCs	4:1	Donor 10	JOVI_Hinge_41bbz
1D	Donor matched TRBC2+ve PBMCs	4:1	Donor 10	JOVI_CD8STK_28z
1E	Donor matched TRBC2+ve PBMCs	4:1	Donor 10	JOVI_CD28STK_CD28TM_28z
1F	Donor matched TRBC2+ve PBMCs	4:1	Donor 10	mJOVI_Hinge_41bbz
1G	Donor matched TRBC2+ve PBMCs	4:1	Donor 10	aCD19-CAR
1H	Donor matched TRBC2+ve PBMCs	4:1	Donor 10	Non-transduced
2A	Donor matched TRBC2+ve PBMCs	1:1	Donor 10	JOVI_Hinge_41bbz
2D	Donor matched TRBC2+ve PBMCs	1:1	Donor 10	JOVI_CD8STK_28z
2E	Donor matched TRBC2+ve PBMCs	1:1	Donor 10	JOVI_CD28STK_CD28TM_28z
2F	Donor matched TRBC2+ve PBMCs	1:1	Donor 10	mJOVI_Hinge_41bbz
2G	Donor matched TRBC2+ve PBMCs	1:1	Donor 10	aCD19-CAR
2H	Donor matched TRBC2+ve PBMCs	1:1	Donor 10	Non-transduced
3A	Donor matched TRBC2+ve PBMCs	1:4	Donor 10	JOVI_Hinge_41bbz
3D	Donor matched TRBC2+ve PBMCs	1:4	Donor 10	JOVI_CD8STK_28z
3E	Donor matched TRBC2+ve PBMCs	1:4	Donor 10	JOVI_CD28STK_CD28TM_28z
3F	Donor matched TRBC2+ve PBMCs	1:4	Donor 10	mJOVI_Hinge_41bbz
3G	Donor matched TRBC2+ve PBMCs	1:4	Donor 10	aCD19-CAR
3H	Donor matched TRBC2+ve PBMCs	1:4	Donor 10	Non-transduced
4A	Donor matched TRBC2+ve PBMCs	4:1	Donor 11	JOVI_Hinge_41bbz
4D	Donor matched TRBC2+ve PBMCs	4:1	Donor 11	JOVI_CD8STK_28z
4E	Donor matched TRBC2+ve PBMCs	4:1	Donor 11	JOVI_CD28STK_CD28TM_28z
4F	Donor matched TRBC2+ve PBMCs	4:1	Donor 11	mJOVI_Hinge_41bbz
4G	Donor matched TRBC2+ve PBMCs	4:1	Donor 11	aCD19-CAR
4H	Donor matched TRBC2+ve PBMCs	4:1	Donor 11	Non-transduced
5A	Donor matched TRBC2+ve PBMCs	1:1	Donor 11	JOVI_Hinge_41bbz
5D	Donor matched TRBC2+ve PBMCs	1:1	Donor 11	JOVI_CD8STK_28z
5E	Donor matched TRBC2+ve PBMCs	1:1	Donor 11	JOVI_CD28STK_CD28TM_28z
5F	Donor matched TRBC2+ve PBMCs	1:1	Donor 11	mJOVI_Hinge_41bbz
5G	Donor matched TRBC2+ve PBMCs	1:1	Donor 11	aCD19-CAR
5H	Donor matched TRBC2+ve PBMCs	1:1	Donor 11	Non-transduced
6A	Donor matched TRBC2+ve PBMCs	1:4	Donor 11	JOVI_Hinge_41bbz
6D	Donor matched TRBC2+ve PBMCs	1:4	Donor 11	JOVI_CD8STK_28z
6E	Donor matched TRBC2+ve PBMCs	1:4	Donor 11	JOVI_CD28STK_CD28TM_28z
6F	Donor matched TRBC2+ve PBMCs	1:4	Donor 11	mJOVI_Hinge_41bbz
6G	Donor matched TRBC2+ve PBMCs	1:4	Donor 11	aCD19-CAR
6H	Donor matched TRBC2+ve PBMCs	1:4	Donor 11	Non-transduced
7A	Donor matched TRBC2+ve PBMCs	4:1	Donor 12	JOVI_Hinge_41bbz
7D	Donor matched TRBC2+ve PBMCs	4:1	Donor 12	JOVI_CD8STK_28z
7E	Donor matched TRBC2+ve PBMCs	4:1	Donor 12	JOVI_CD28STK_CD28TM_28z
7F	Donor matched TRBC2+ve PBMCs	4:1	Donor 12	mJOVI_Hinge_41bbz
7G	Donor matched TRBC2+ve PBMCs	4:1	Donor 12	aCD19-CAR
7H	Donor matched TRBC2+ve PBMCs	4:1	Donor 12	Non-transduced
8A	Donor matched TRBC2+ve PBMCs	1:1	Donor 12	JOVI_Hinge_41bbz
8D	Donor matched TRBC2+ve PBMCs	1:1	Donor 12	JOVI_CD8STK_28z
8E	Donor matched TRBC2+ve PBMCs	1:1	Donor 12	JOVI_CD28STK_CD28TM_28z
8F	Donor matched TRBC2+ve PBMCs	1:1	Donor 12	mJOVI_Hinge_41bbz
8G	Donor matched TRBC2+ve PBMCs	1:1	Donor 12	aCD19-CAR
8H	Donor matched TRBC2+ve PBMCs	1:1	Donor 12	Non-transduced

**9A**	Donor matched TRBC2+ve PBMCs	1:4	Donor 12	JOVI_Hinge_41bbz
**9D**	Donor matched TRBC2+ve PBMCs	1:4	Donor 12	JOVI_CD8STK_28z
**9E**	Donor matched TRBC2+ve PBMCs	1:4	Donor 12	JOVI_CD28STK_CD28TM_28z
**9F**	Donor matched TRBC2+ve PBMCs	1:4	Donor 12	mJOVI_Hinge_41bbz
**9G**	Donor matched TRBC2+ve PBMCs	1:4	Donor 12	aCD19-CAR
**9H**	Donor matched TRBC2+ve PBMCs	1:4	Donor 12	Non-transduced
**10A**	Donor matched TRBC2+ve PBMCs	4:1	Donor 13	JOVI_Hinge_41bbz
**10D**	Donor matched TRBC2+ve PBMCs	4:1	Donor 13	JOVI_CD8STK_28z
**10E**	Donor matched TRBC2+ve PBMCs	4:1	Donor 13	JOVI_CD28STK_CD28TM_28z
**10F**	Donor matched TRBC2+ve PBMCs	4:1	Donor 13	mJOVI_Hinge_41bbz
**10G**	Donor matched TRBC2+ve PBMCs	4:1	Donor 13	aCD19-CAR
**10H**	Donor matched TRBC2+ve PBMCs	4:1	Donor 13	Non-transduced
**11A**	Donor matched TRBC2+ve PBMCs	1:1	Donor 13	JOVI_Hinge_41bbz
**11D**	Donor matched TRBC2+ve PBMCs	1:1	Donor 13	JOVI_CD8STK_28z
**11E**	Donor matched TRBC2+ve PBMCs	1:1	Donor 13	JOVI_CD28STK_CD28TM_28z
**11F**	Donor matched TRBC2+ve PBMCs	1:1	Donor 13	mJOVI_Hinge_41bbz
**11G**	Donor matched TRBC2+ve PBMCs	1:1	Donor 13	aCD19-CAR
**11H**	Donor matched TRBC2+ve PBMCs	1:1	Donor 13	Non-transduced
**12A**	Donor matched TRBC2+ve PBMCs	1:4	Donor 13	JOVI_Hinge_41bbz
**12D**	Donor matched TRBC2+ve PBMCs	1:4	Donor 13	JOVI_CD8STK_28z
**12E**	Donor matched TRBC2+ve PBMCs	1:4	Donor 13	JOVI_CD28STK_CD28TM_28z
**12F**	Donor matched TRBC2+ve PBMCs	1:4	Donor 13	mJOVI_Hinge_41bbz
**12G**	Donor matched TRBC2+ve PBMCs	1:4	Donor 13	aCD19-CAR
**12H**	Donor matched TRBC2+ve PBMCs	1:4	Donor 13	Non-transduced

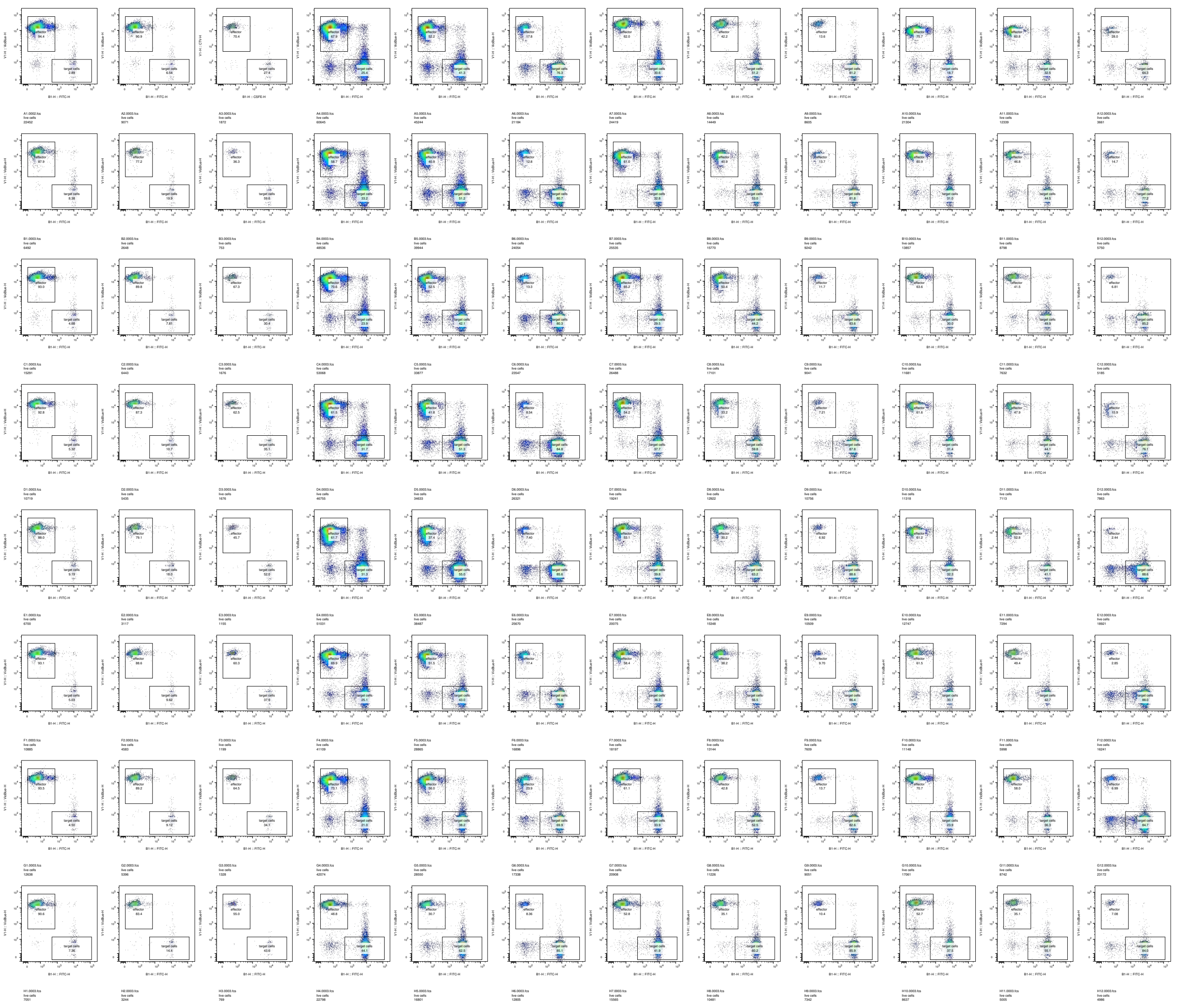

Reverse_Kill_KFN_vs_T1

Well	Target	E:T ratio	Donor	CAR construct
1B	Donor matched TRBC1+ve PBMCs	4:1	Donor 10	KFN_Hinge_28z
1D	Donor matched TRBC1+ve PBMCs	4:1	Donor 10	KFN_CD8STK_28z
1E	Donor matched TRBC1+ve PBMCs	4:1	Donor 10	KFN_CD28STK_CD28TM_28z
1F	Donor matched TRBC1+ve PBMCs	4:1	Donor 10	aCD19-CAR
1G	Donor matched TRBC1+ve PBMCs	4:1	Donor 10	Non-transduced
2B	Donor matched TRBC1+ve PBMCs	1:1	Donor 10	KFN_Hinge_28z
2D	Donor matched TRBC1+ve PBMCs	1:1	Donor 10	KFN_CD8STK_28z
2E	Donor matched TRBC1+ve PBMCs	1:1	Donor 10	KFN_CD28STK_CD28TM_28z
2F	Donor matched TRBC1+ve PBMCs	1:1	Donor 10	aCD19-CAR
2G	Donor matched TRBC1+ve PBMCs	1:1	Donor 10	Non-transduced
3B	Donor matched TRBC1+ve PBMCs	1:4	Donor 10	KFN_Hinge_28z
3D	Donor matched TRBC1+ve PBMCs	1:4	Donor 10	KFN_CD8STK_28z
3E	Donor matched TRBC1+ve PBMCs	1:4	Donor 10	KFN_CD28STK_CD28TM_28z
3F	Donor matched TRBC1+ve PBMCs	1:4	Donor 10	aCD19-CAR
3G	Donor matched TRBC1+ve PBMCs	1:4	Donor 10	Non-transduced
4B	Donor matched TRBC1+ve PBMCs	4:1	Donor 11	KFN_Hinge_28z
4D	Donor matched TRBC1+ve PBMCs	4:1	Donor 11	KFN_CD8STK_28z
4E	Donor matched TRBC1+ve PBMCs	4:1	Donor 11	KFN_CD28STK_CD28TM_28z
4F	Donor matched TRBC1+ve PBMCs	4:1	Donor 11	aCD19-CAR
4G	Donor matched TRBC1+ve PBMCs	4:1	Donor 11	Non-transduced
5B	Donor matched TRBC1+ve PBMCs	1:1	Donor 11	KFN_Hinge_28z
5D	Donor matched TRBC1+ve PBMCs	1:1	Donor 11	KFN_CD8STK_28z
5E	Donor matched TRBC1+ve PBMCs	1:1	Donor 11	KFN_CD28STK_CD28TM_28z
5F	Donor matched TRBC1+ve PBMCs	1:1	Donor 11	aCD19-CAR
5G	Donor matched TRBC1+ve PBMCs	1:1	Donor 11	Non-transduced
6B	Donor matched TRBC1+ve PBMCs	1:4	Donor 11	KFN_Hinge_28z
6D	Donor matched TRBC1+ve PBMCs	1:4	Donor 11	KFN_CD8STK_28z
6E	Donor matched TRBC1+ve PBMCs	1:4	Donor 11	KFN_CD28STK_CD28TM_28z
6F	Donor matched TRBC1+ve PBMCs	1:4	Donor 11	aCD19-CAR
6G	Donor matched TRBC1+ve PBMCs	1:4	Donor 11	Non-transduced
7B	Donor matched TRBC1+ve PBMCs	4:1	Donor 12	KFN_Hinge_28z
7D	Donor matched TRBC1+ve PBMCs	4:1	Donor 12	KFN_CD8STK_28z
7E	Donor matched TRBC1+ve PBMCs	4:1	Donor 12	KFN_CD28STK_CD28TM_28z
7F	Donor matched TRBC1+ve PBMCs	4:1	Donor 12	aCD19-CAR
7G	Donor matched TRBC1+ve PBMCs	4:1	Donor 12	Non-transduced
8B	Donor matched TRBC1+ve PBMCs	1:1	Donor 12	KFN_Hinge_28z
8D	Donor matched TRBC1+ve PBMCs	1:1	Donor 12	KFN_CD8STK_28z
8E	Donor matched TRBC1+ve PBMCs	1:1	Donor 12	KFN_CD28STK_CD28TM_28z
8F	Donor matched TRBC1+ve PBMCs	1:1	Donor 12	aCD19-CAR
8G	Donor matched TRBC1+ve PBMCs	1:1	Donor 12	Non-transduced
9B	Donor matched TRBC1+ve PBMCs	1:4	Donor 12	KFN_Hinge_28z
9D	Donor matched TRBC1+ve PBMCs	1:4	Donor 12	KFN_CD8STK_28z
9E	Donor matched TRBC1+ve PBMCs	1:4	Donor 12	KFN_CD28STK_CD28TM_28z
9F	Donor matched TRBC1+ve PBMCs	1:4	Donor 12	aCD19-CAR
9G	Donor matched TRBC1+ve PBMCs	1:4	Donor 12	Non-transduced
10B	Donor matched TRBC1+ve PBMCs	4:1	Donor 13	KFN_Hinge_28z
10D	Donor matched TRBC1+ve PBMCs	4:1	Donor 13	KFN_CD8STK_28z
10E	Donor matched TRBC1+ve PBMCs	4:1	Donor 13	KFN_CD28STK_CD28TM_28z

**10F**	Donor matched TRBC1+ve PBMCs	4:1	Donor 13	aCD19-CAR
**10G**	Donor matched TRBC1+ve PBMCs	4:1	Donor 13	Non-transduced
**11B**	Donor matched TRBC1+ve PBMCs	1:1	Donor 13	KFN_Hinge_28z
**11D**	Donor matched TRBC1+ve PBMCs	1:1	Donor 13	KFN_CD8STK_28z
**11E**	Donor matched TRBC1+ve PBMCs	1:1	Donor 13	KFN_CD28STK_CD28TM_28z
**11F**	Donor matched TRBC1+ve PBMCs	1:1	Donor 13	aCD19-CAR
**11G**	Donor matched TRBC1+ve PBMCs	1:1	Donor 13	Non-transduced
**12B**	Donor matched TRBC1+ve PBMCs	1:4	Donor 13	KFN_Hinge_28z
**12D**	Donor matched TRBC1+ve PBMCs	1:4	Donor 13	KFN_CD8STK_28z
**12E**	Donor matched TRBC1+ve PBMCs	1:4	Donor 13	KFN_CD28STK_CD28TM_28z
**12F**	Donor matched TRBC1+ve PBMCs	1:4	Donor 13	aCD19-CAR
**12G**	Donor matched TRBC1+ve PBMCs	1:4	Donor 13	Non-transduced

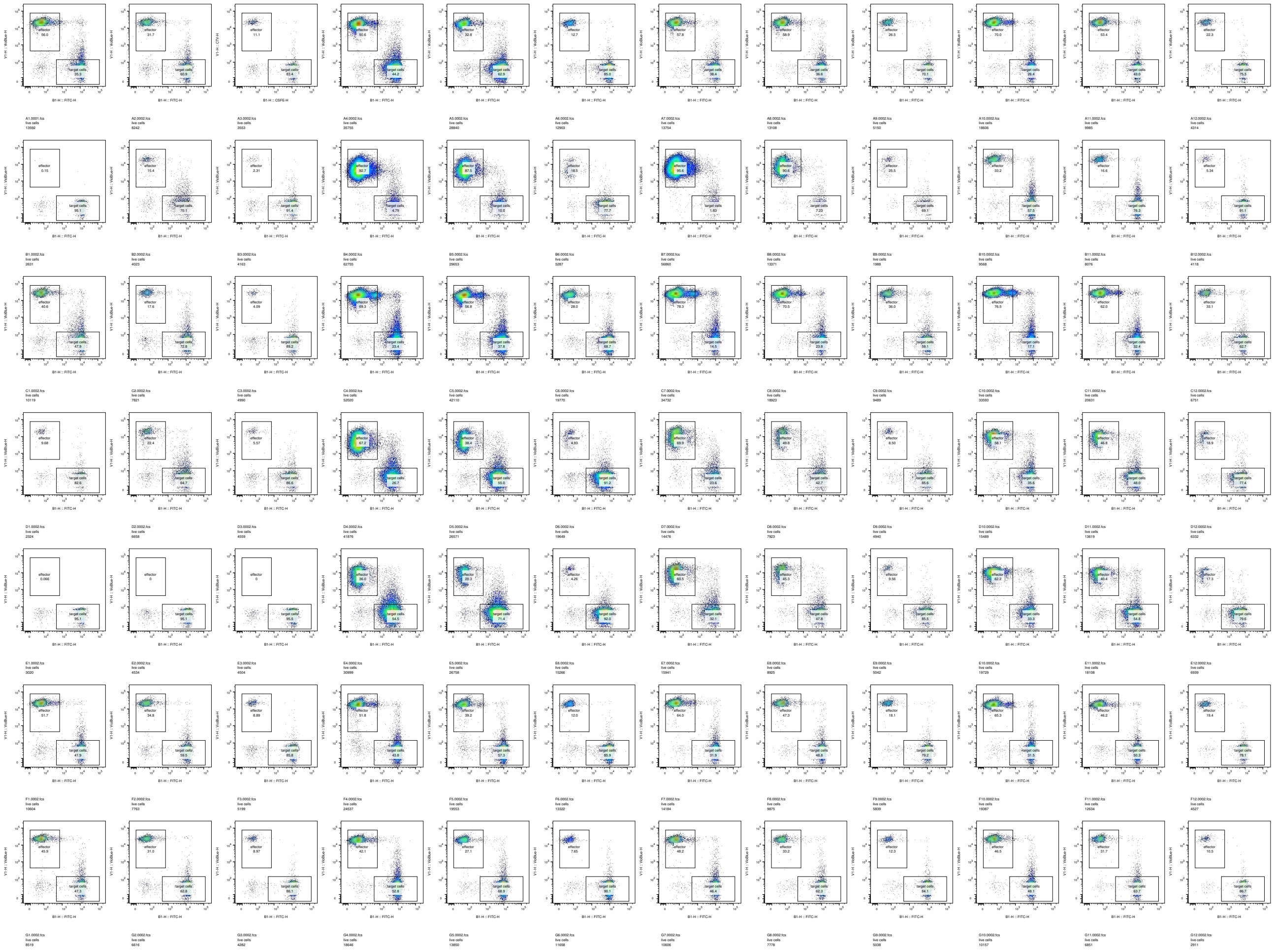

Reverse_Kill_KFN_vs_T2

Well	Target	E:T ratio	Donor	CAR construct
1B	Donor matched TRBC2+ve PBMCs	4:1	Donor 10	KFN_Hinge_28z
1D	Donor matched TRBC2+ve PBMCs	4:1	Donor 10	KFN_CD8STK_28z
1E	Donor matched TRBC2+ve PBMCs	4:1	Donor 10	KFN_CD28STK_CD28TM_28z
1F	Donor matched TRBC2+ve PBMCs	4:1	Donor 10	aCD19-CAR
1G	Donor matched TRBC2+ve PBMCs	4:1	Donor 10	Non-transduced
2B	Donor matched TRBC2+ve PBMCs	1:1	Donor 10	KFN_Hinge_28z
2D	Donor matched TRBC2+ve PBMCs	1:1	Donor 10	KFN_CD8STK_28z
2E	Donor matched TRBC2+ve PBMCs	1:1	Donor 10	KFN_CD28STK_CD28TM_28z
2F	Donor matched TRBC2+ve PBMCs	1:1	Donor 10	aCD19-CAR
2G	Donor matched TRBC2+ve PBMCs	1:1	Donor 10	Non-transduced
3B	Donor matched TRBC2+ve PBMCs	1:4	Donor 10	KFN_Hinge_28z
3D	Donor matched TRBC2+ve PBMCs	1:4	Donor 10	KFN_CD8STK_28z
3E	Donor matched TRBC2+ve PBMCs	1:4	Donor 10	KFN_CD28STK_CD28TM_28z
3F	Donor matched TRBC2+ve PBMCs	1:4	Donor 10	aCD19-CAR
3G	Donor matched TRBC2+ve PBMCs	1:4	Donor 10	Non-transduced
4B	Donor matched TRBC2+ve PBMCs	4:1	Donor 11	KFN_Hinge_28z
4D	Donor matched TRBC2+ve PBMCs	4:1	Donor 11	KFN_CD8STK_28z
4E	Donor matched TRBC2+ve PBMCs	4:1	Donor 11	KFN_CD28STK_CD28TM_28z
4F	Donor matched TRBC2+ve PBMCs	4:1	Donor 11	aCD19-CAR
4G	Donor matched TRBC2+ve PBMCs	4:1	Donor 11	Non-transduced
5B	Donor matched TRBC2+ve PBMCs	1:1	Donor 11	KFN_Hinge_28z
5D	Donor matched TRBC2+ve PBMCs	1:1	Donor 11	KFN_CD8STK_28z
5E	Donor matched TRBC2+ve PBMCs	1:1	Donor 11	KFN_CD28STK_CD28TM_28z
5F	Donor matched TRBC2+ve PBMCs	1:1	Donor 11	aCD19-CAR
5G	Donor matched TRBC2+ve PBMCs	1:1	Donor 11	Non-transduced
6B	Donor matched TRBC2+ve PBMCs	1:4	Donor 11	KFN_Hinge_28z
6D	Donor matched TRBC2+ve PBMCs	1:4	Donor 11	KFN_CD8STK_28z
6E	Donor matched TRBC2+ve PBMCs	1:4	Donor 11	KFN_CD28STK_CD28TM_28z
6F	Donor matched TRBC2+ve PBMCs	1:4	Donor 11	aCD19-CAR
6G	Donor matched TRBC2+ve PBMCs	1:4	Donor 11	Non-transduced
7B	Donor matched TRBC2+ve PBMCs	4:1	Donor 12	KFN_Hinge_28z
7D	Donor matched TRBC2+ve PBMCs	4:1	Donor 12	KFN_CD8STK_28z
7E	Donor matched TRBC2+ve PBMCs	4:1	Donor 12	KFN_CD28STK_CD28TM_28z
7F	Donor matched TRBC2+ve PBMCs	4:1	Donor 12	aCD19-CAR
7G	Donor matched TRBC2+ve PBMCs	4:1	Donor 12	Non-transduced
8B	Donor matched TRBC2+ve PBMCs	1:1	Donor 12	KFN_Hinge_28z
8D	Donor matched TRBC2+ve PBMCs	1:1	Donor 12	KFN_CD8STK_28z
8E	Donor matched TRBC2+ve PBMCs	1:1	Donor 12	KFN_CD28STK_CD28TM_28z
8F	Donor matched TRBC2+ve PBMCs	1:1	Donor 12	aCD19-CAR
8G	Donor matched TRBC2+ve PBMCs	1:1	Donor 12	Non-transduced
9B	Donor matched TRBC2+ve PBMCs	1:4	Donor 12	KFN_Hinge_28z
9D	Donor matched TRBC2+ve PBMCs	1:4	Donor 12	KFN_CD8STK_28z
9E	Donor matched TRBC2+ve PBMCs	1:4	Donor 12	KFN_CD28STK_CD28TM_28z
9F	Donor matched TRBC2+ve PBMCs	1:4	Donor 12	aCD19-CAR
9G	Donor matched TRBC2+ve PBMCs	1:4	Donor 12	Non-transduced
10B	Donor matched TRBC2+ve PBMCs	4:1	Donor 13	KFN_Hinge_28z
10D	Donor matched TRBC2+ve PBMCs	4:1	Donor 13	KFN_CD8STK_28z
10E	Donor matched TRBC2+ve PBMCs	4:1	Donor 13	KFN_CD28STK_CD28TM_28z
