# Supplementary material for: Structure-guided engineering of immunotherapies targeting TRBC1 and TRBC2 in T cell malignancies
Source: Nat Commun. 2024 Feb 21;15:1583. doi: 10.1038/s41467-024-45854-3 (PMC10881500; doi:10.1038/s41467-024-45854-3)

Reverse_Kill_KFN_vs_T2

Well	Target	E:T ratio	Donor	CAR construct
**1B**	Donor matched TRBC2+ve PBMCs	4:1	Donor 10	KFN_Hinge_28z
**1D**	Donor matched TRBC2+ve PBMCs	4:1	Donor 10	KFN_CD8STK_28z
**1E**	Donor matched TRBC2+ve PBMCs	4:1	Donor 10	KFN_CD28STK_CD28TM_28z
**1F**	Donor matched TRBC2+ve PBMCs	4:1	Donor 10	aCD19-CAR
**1G**	Donor matched TRBC2+ve PBMCs	4:1	Donor 10	Non-transduced
**2B**	Donor matched TRBC2+ve PBMCs	1:1	Donor 10	KFN_Hinge_28z
**2D**	Donor matched TRBC2+ve PBMCs	1:1	Donor 10	KFN_CD8STK_28z
**2E**	Donor matched TRBC2+ve PBMCs	1:1	Donor 10	KFN_CD28STK_CD28TM_28z
**2F**	Donor matched TRBC2+ve PBMCs	1:1	Donor 10	aCD19-CAR
**2G**	Donor matched TRBC2+ve PBMCs	1:1	Donor 10	Non-transduced
**3B**	Donor matched TRBC2+ve PBMCs	1:4	Donor 10	KFN_Hinge_28z
**3D**	Donor matched TRBC2+ve PBMCs	1:4	Donor 10	KFN_CD8STK_28z
**3E**	Donor matched TRBC2+ve PBMCs	1:4	Donor 10	KFN_CD28STK_CD28TM_28z
**3F**	Donor matched TRBC2+ve PBMCs	1:4	Donor 10	aCD19-CAR
**3G**	Donor matched TRBC2+ve PBMCs	1:4	Donor 10	Non-transduced
**4B**	Donor matched TRBC2+ve PBMCs	4:1	Donor 11	KFN_Hinge_28z
**4D**	Donor matched TRBC2+ve PBMCs	4:1	Donor 11	KFN_CD8STK_28z
**4E**	Donor matched TRBC2+ve PBMCs	4:1	Donor 11	KFN_CD28STK_CD28TM_28z
**4F**	Donor matched TRBC2+ve PBMCs	4:1	Donor 11	aCD19-CAR
**4G**	Donor matched TRBC2+ve PBMCs	4:1	Donor 11	Non-transduced
**5B**	Donor matched TRBC2+ve PBMCs	1:1	Donor 11	KFN_Hinge_28z
**5D**	Donor matched TRBC2+ve PBMCs	1:1	Donor 11	KFN_CD8STK_28z
**5E**	Donor matched TRBC2+ve PBMCs	1:1	Donor 11	KFN_CD28STK_CD28TM_28z
**5F**	Donor matched TRBC2+ve PBMCs	1:1	Donor 11	aCD19-CAR
**5G**	Donor matched TRBC2+ve PBMCs	1:1	Donor 11	Non-transduced
**6B**	Donor matched TRBC2+ve PBMCs	1:4	Donor 11	KFN_Hinge_28z
**6D**	Donor matched TRBC2+ve PBMCs	1:4	Donor 11	KFN_CD8STK_28z
**6E**	Donor matched TRBC2+ve PBMCs	1:4	Donor 11	KFN_CD28STK_CD28TM_28z
**6F**	Donor matched TRBC2+ve PBMCs	1:4	Donor 11	aCD19-CAR
**6G**	Donor matched TRBC2+ve PBMCs	1:4	Donor 11	Non-transduced
**7B**	Donor matched TRBC2+ve PBMCs	4:1	Donor 12	KFN_Hinge_28z
**7D**	Donor matched TRBC2+ve PBMCs	4:1	Donor 12	KFN_CD8STK_28z
**7E**	Donor matched TRBC2+ve PBMCs	4:1	Donor 12	KFN_CD28STK_CD28TM_28z
**7F**	Donor matched TRBC2+ve PBMCs	4:1	Donor 12	aCD19-CAR
**7G**	Donor matched TRBC2+ve PBMCs	4:1	Donor 12	Non-transduced
**8B**	Donor matched TRBC2+ve PBMCs	1:1	Donor 12	KFN_Hinge_28z
**8D**	Donor matched TRBC2+ve PBMCs	1:1	Donor 12	KFN_CD8STK_28z
**8E**	Donor matched TRBC2+ve PBMCs	1:1	Donor 12	KFN_CD28STK_CD28TM_28z
**8F**	Donor matched TRBC2+ve PBMCs	1:1	Donor 12	aCD19-CAR
**8G**	Donor matched TRBC2+ve PBMCs	1:1	Donor 12	Non-transduced
**9B**	Donor matched TRBC2+ve PBMCs	1:4	Donor 12	KFN_Hinge_28z
**9D**	Donor matched TRBC2+ve PBMCs	1:4	Donor 12	KFN_CD8STK_28z
**9E**	Donor matched TRBC2+ve PBMCs	1:4	Donor 12	KFN_CD28STK_CD28TM_28z
**9F**	Donor matched TRBC2+ve PBMCs	1:4	Donor 12	aCD19-CAR
**9G**	Donor matched TRBC2+ve PBMCs	1:4	Donor 12	Non-transduced
**10B**	Donor matched TRBC2+ve PBMCs	4:1	Donor 13	KFN_Hinge_28z
**10D**	Donor matched TRBC2+ve PBMCs	4:1	Donor 13	KFN_CD8STK_28z
**10E**	Donor matched TRBC2+ve PBMCs	4:1	Donor 13	KFN_CD28STK_CD28TM_28z

**10F**	Donor matched TRBC2+ve PBMCs	4:1	Donor 13	aCD19-CAR
**10G**	Donor matched TRBC2+ve PBMCs	4:1	Donor 13	Non-transduced
**11B**	Donor matched TRBC2+ve PBMCs	1:1	Donor 13	KFN_Hinge_28z
**11D**	Donor matched TRBC2+ve PBMCs	1:1	Donor 13	KFN_CD8STK_28z
**11E**	Donor matched TRBC2+ve PBMCs	1:1	Donor 13	KFN_CD28STK_CD28TM_28z
**11F**	Donor matched TRBC2+ve PBMCs	1:1	Donor 13	aCD19-CAR
**11G**	Donor matched TRBC2+ve PBMCs	1:1	Donor 13	Non-transduced
**12B**	Donor matched TRBC2+ve PBMCs	1:4	Donor 13	KFN_Hinge_28z
**12D**	Donor matched TRBC2+ve PBMCs	1:4	Donor 13	KFN_CD8STK_28z
**12E**	Donor matched TRBC2+ve PBMCs	1:4	Donor 13	KFN_CD28STK_CD28TM_28z
**12F**	Donor matched TRBC2+ve PBMCs	1:4	Donor 13	aCD19-CAR
**12G**	Donor matched TRBC2+ve PBMCs	1:4	Donor 13	Non-transduced

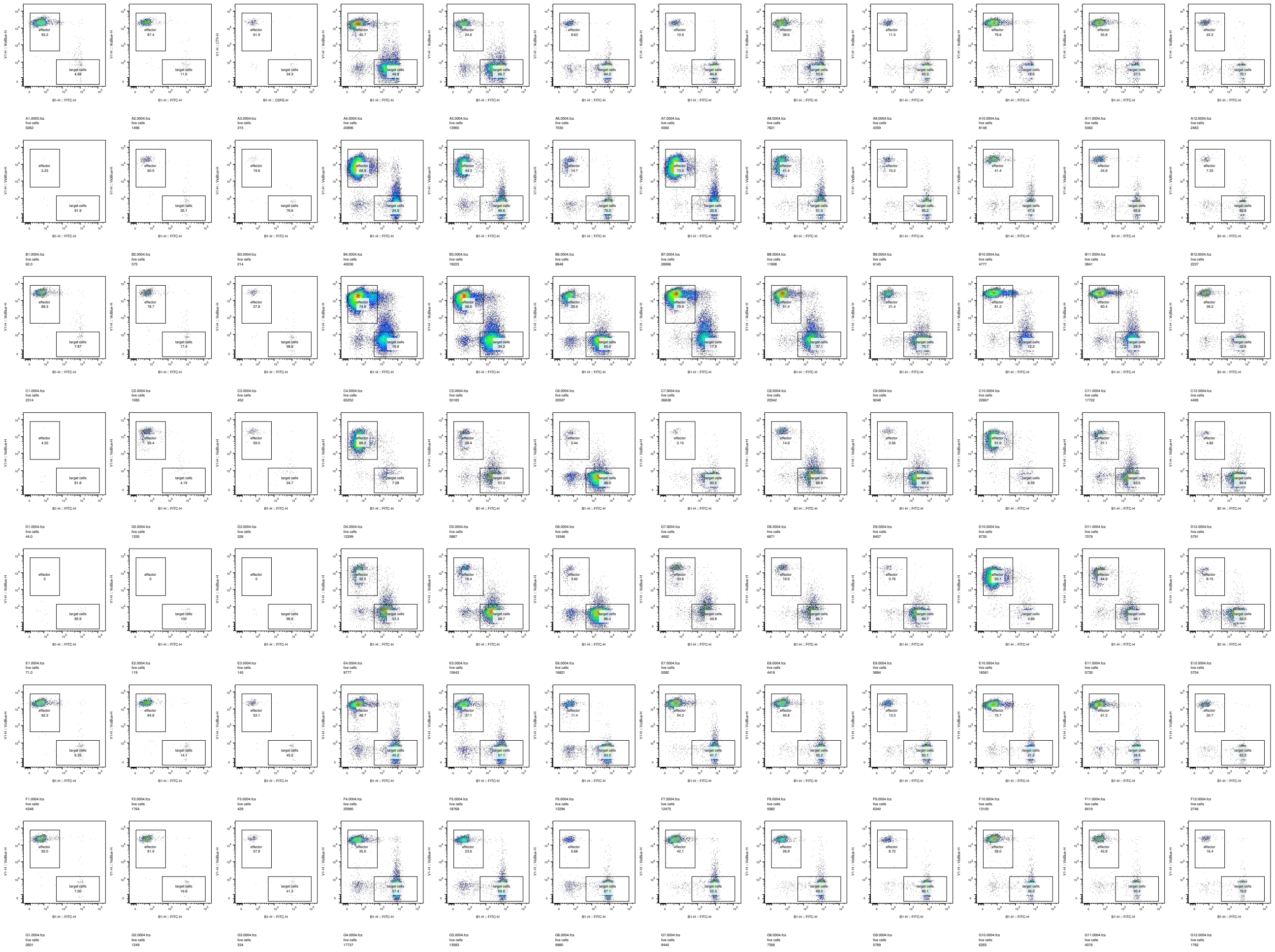

Plate_KFN_vs_HDMAR

**Well**	**Target**	**E:T ratio**	**Donor**	**CAR construct**
**1B**	HDMAR	1:4	Donor 10	KFN_Hinge_28z
**1D**	HDMAR	1:4	Donor 10	KFN_CD8STK_28z
**1E**	HDMAR	1:4	Donor 10	KFN_CD28STK_CD28TM_28z
**1F**	HDMAR	1:4	Donor 10	aCD19-CAR
**1G**	HDMAR	1:4	Donor 10	Non-transduced
**1H**	HDMAR	N/A	N/A	N/A
**2B**	HDMAR	1:8	Donor 10	KFN_Hinge_28z
**2D**	HDMAR	1:8	Donor 10	KFN_CD8STK_28z
**2E**	HDMAR	1:8	Donor 10	KFN_CD28STK_CD28TM_28z
**2F**	HDMAR	1:8	Donor 10	aCD19-CAR
**2G**	HDMAR	1:8	Donor 10	Non-transduced
**2H**	HDMAR	N/A	N/A	N/A
**4B**	HDMAR	1:4	Donor 11	KFN_Hinge_28z
**4D**	HDMAR	1:4	Donor 11	KFN_CD8STK_28z
**4E**	HDMAR	1:4	Donor 11	KFN_CD28STK_CD28TM_28z
**4F**	HDMAR	1:4	Donor 11	aCD19-CAR
**4G**	HDMAR	1:4	Donor 11	Non-transduced
**4H**	HDMAR	N/A	N/A	N/A
**5B**	HDMAR	1:8	Donor 11	KFN_Hinge_28z
**5D**	HDMAR	1:8	Donor 11	KFN_CD8STK_28z
**5E**	HDMAR	1:8	Donor 11	KFN_CD28STK_CD28TM_28z
**5F**	HDMAR	1:8	Donor 11	aCD19-CAR
**5G**	HDMAR	1:8	Donor 11	Non-transduced
**5H**	HDMAR	N/A	N/A	N/A
**7B**	HDMAR	1:4	Donor 12	KFN_Hinge_28z
**7D**	HDMAR	1:4	Donor 12	KFN_CD8STK_28z
**7E**	HDMAR	1:4	Donor 12	KFN_CD28STK_CD28TM_28z
**7F**	HDMAR	1:4	Donor 12	aCD19-CAR
**7G**	HDMAR	1:4	Donor 12	Non-transduced
**7H**	HDMAR	N/A	N/A	N/A
**8B**	HDMAR	1:8	Donor 12	KFN_Hinge_28z
**8D**	HDMAR	1:8	Donor 12	KFN_CD8STK_28z
**8E**	HDMAR	1:8	Donor 12	KFN_CD28STK_CD28TM_28z
**8F**	HDMAR	1:8	Donor 12	aCD19-CAR
**8G**	HDMAR	1:8	Donor 12	Non-transduced
**8H**	HDMAR	N/A	N/A	N/A
**10B**	HDMAR	1:4	Donor 13	KFN_Hinge_28z
**10D**	HDMAR	1:4	Donor 13	KFN_CD8STK_28z
**10E**	HDMAR	1:4	Donor 13	KFN_CD28STK_CD28TM_28z
**10F**	HDMAR	1:4	Donor 13	aCD19-CAR
**10G**	HDMAR	1:4	Donor 13	Non-transduced
**10H**	HDMAR	N/A	N/A	N/A
**11B**	HDMAR	1:8	Donor 13	KFN_Hinge_28z
**11D**	HDMAR	1:8	Donor 13	KFN_CD8STK_28z
**11E**	HDMAR	1:8	Donor 13	KFN_CD28STK_CD28TM_28z
**11F**	HDMAR	1:8	Donor 13	aCD19-CAR
**11G**	HDMAR	1:8	Donor 13	Non-transduced
**11H**	HDMAR	N/A	N/A	N/A

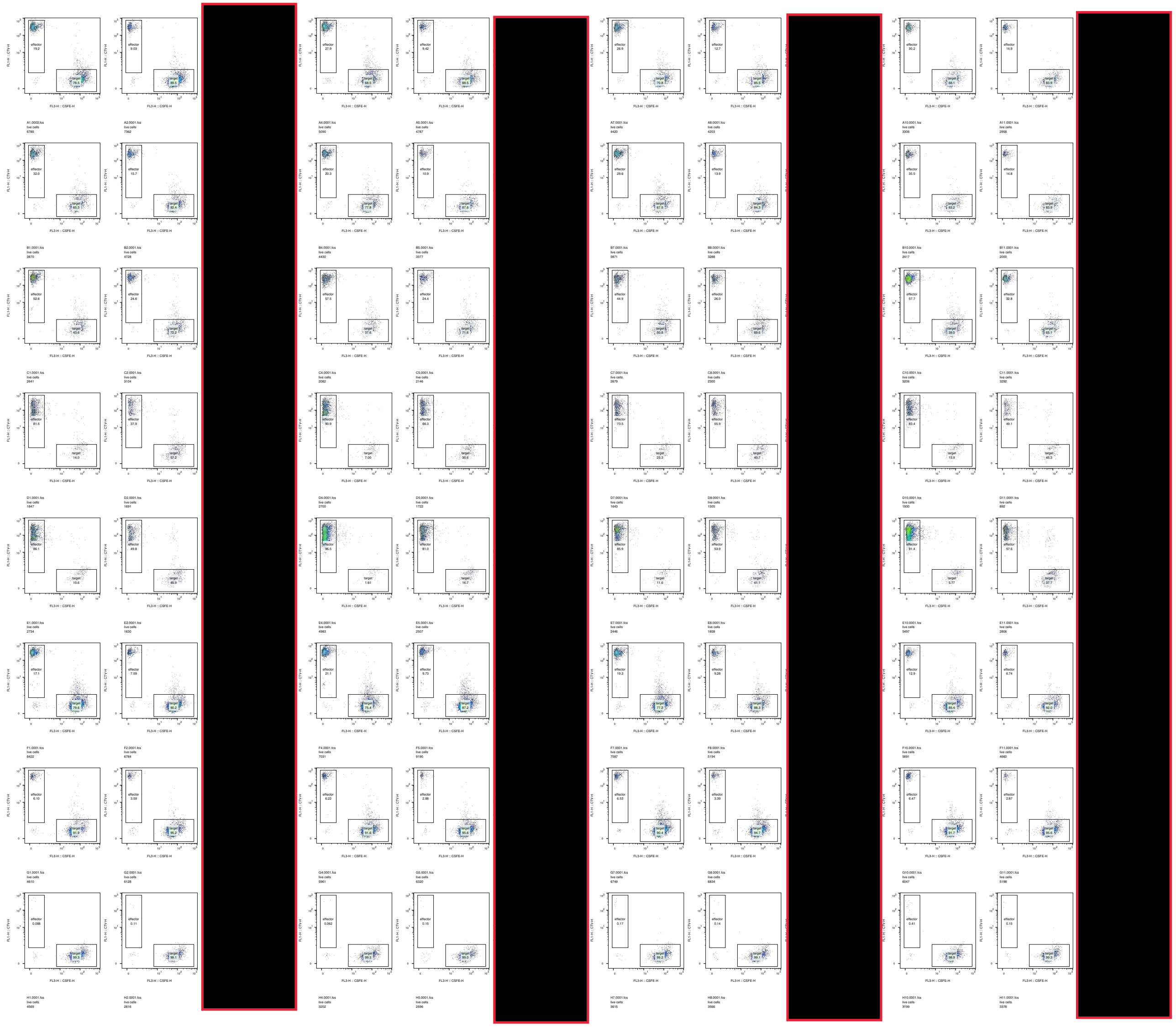

Plate_KFN_vs_PT1707

Well	Target	E:T ratio	Donor	CAR construct
**1B**	T-PLL_Primary Tumour 1_TRBC1+ve	4:1	Donor 10	KFN_Hinge_28z
**1D**	T-PLL_Primary Tumour 1_TRBC1+ve	4:1	Donor 10	KFN_CD8STK_28z
**1E**	T-PLL_Primary Tumour 1_TRBC1+ve	4:1	Donor 10	KFN_CD28STK_CD28TM_28z
**1F**	T-PLL_Primary Tumour 1_TRBC1+ve	4:1	Donor 10	aCD19-CAR
**1G**	T-PLL_Primary Tumour 1_TRBC1+ve	4:1	Donor 10	Non-transduced
**2B**	T-PLL_Primary Tumour 1_TRBC1+ve	1:1	Donor 10	KFN_Hinge_28z
**2D**	T-PLL_Primary Tumour 1_TRBC1+ve	1:1	Donor 10	KFN_CD8STK_28z
**2E**	T-PLL_Primary Tumour 1_TRBC1+ve	1:1	Donor 10	KFN_CD28STK_CD28TM_28z
**2F**	T-PLL_Primary Tumour 1_TRBC1+ve	1:1	Donor 10	aCD19-CAR
**2G**	T-PLL_Primary Tumour 1_TRBC1+ve	1:1	Donor 10	Non-transduced
**3B**	T-PLL_Primary Tumour 1_TRBC1+ve	1:4	Donor 10	KFN_Hinge_28z
**3D**	T-PLL_Primary Tumour 1_TRBC1+ve	1:4	Donor 10	KFN_CD8STK_28z
**3E**	T-PLL_Primary Tumour 1_TRBC1+ve	1:4	Donor 10	KFN_CD28STK_CD28TM_28z
**3F**	T-PLL_Primary Tumour 1_TRBC1+ve	1:4	Donor 10	aCD19-CAR
**3G**	T-PLL_Primary Tumour 1_TRBC1+ve	1:4	Donor 10	Non-transduced
**4B**	T-PLL_Primary Tumour 1_TRBC1+ve	4:1	Donor 11	KFN_Hinge_28z
**4D**	T-PLL_Primary Tumour 1_TRBC1+ve	4:1	Donor 11	KFN_CD8STK_28z
**4E**	T-PLL_Primary Tumour 1_TRBC1+ve	4:1	Donor 11	KFN_CD28STK_CD28TM_28z
**4F**	T-PLL_Primary Tumour 1_TRBC1+ve	4:1	Donor 11	aCD19-CAR
**4G**	T-PLL_Primary Tumour 1_TRBC1+ve	4:1	Donor 11	Non-transduced
**5B**	T-PLL_Primary Tumour 1_TRBC1+ve	1:1	Donor 11	KFN_Hinge_28z
**5D**	T-PLL_Primary Tumour 1_TRBC1+ve	1:1	Donor 11	KFN_CD8STK_28z
**5E**	T-PLL_Primary Tumour 1_TRBC1+ve	1:1	Donor 11	KFN_CD28STK_CD28TM_28z
**5F**	T-PLL_Primary Tumour 1_TRBC1+ve	1:1	Donor 11	aCD19-CAR

**5G**	T-PLL_Primary Tumour 1_TRBC1+ve	1:1	Donor 11	Non-transduced
**6B**	T-PLL_Primary Tumour 1_TRBC1+ve	1:4	Donor 11	KFN_Hinge_28z
**6D**	T-PLL_Primary Tumour 1_TRBC1+ve	1:4	Donor 11	KFN_CD8STK_28z
**6E**	T-PLL_Primary Tumour 1_TRBC1+ve	1:4	Donor 11	KFN_CD28STK_CD28TM_28z
**6F**	T-PLL_Primary Tumour 1_TRBC1+ve	1:4	Donor 11	aCD19-CAR
**6G**	T-PLL_Primary Tumour 1_TRBC1+ve	1:4	Donor 11	Non-transduced
**7B**	T-PLL_Primary Tumour 1_TRBC1+ve	4:1	Donor 12	KFN_Hinge_28z
**7D**	T-PLL_Primary Tumour 1_TRBC1+ve	4:1	Donor 12	KFN_CD8STK_28z
**7E**	T-PLL_Primary Tumour 1_TRBC1+ve	4:1	Donor 12	KFN_CD28STK_CD28TM_28z
**7F**	T-PLL_Primary Tumour 1_TRBC1+ve	4:1	Donor 12	aCD19-CAR
**7G**	T-PLL_Primary Tumour 1_TRBC1+ve	4:1	Donor 12	Non-transduced
**8B**	T-PLL_Primary Tumour 1_TRBC1+ve	1:1	Donor 12	KFN_Hinge_28z
**8D**	T-PLL_Primary Tumour 1_TRBC1+ve	1:1	Donor 12	KFN_CD8STK_28z
**8E**	T-PLL_Primary Tumour 1_TRBC1+ve	1:1	Donor 12	KFN_CD28STK_CD28TM_28z
**8F**	T-PLL_Primary Tumour 1_TRBC1+ve	1:1	Donor 12	aCD19-CAR
**8G**	T-PLL_Primary Tumour 1_TRBC1+ve	1:1	Donor 12	Non-transduced
**9B**	T-PLL_Primary Tumour 1_TRBC1+ve	1:4	Donor 12	KFN_Hinge_28z
**9D**	T-PLL_Primary Tumour 1_TRBC1+ve	1:4	Donor 12	KFN_CD8STK_28z
**9E**	T-PLL_Primary Tumour 1_TRBC1+ve	1:4	Donor 12	KFN_CD28STK_CD28TM_28z
**9F**	T-PLL_Primary Tumour 1_TRBC1+ve	1:4	Donor 12	aCD19-CAR
**9G**	T-PLL_Primary Tumour 1_TRBC1+ve	1:4	Donor 12	Non-transduced
**10B**	T-PLL_Primary Tumour 1_TRBC1+ve	4:1	Donor 13	KFN_Hinge_28z
**10D**	T-PLL_Primary Tumour 1_TRBC1+ve	4:1	Donor 13	KFN_CD8STK_28z
**10E**	T-PLL_Primary Tumour 1_TRBC1+ve	4:1	Donor 13	KFN_CD28STK_CD28TM_28z
**10F**	T-PLL_Primary Tumour 1_TRBC1+ve	4:1	Donor 13	aCD19-CAR

**10G**	T-PLL_Primary Tumour 1_TRBC1+ve	4:1	Donor 13	Non-transduced
**11B**	T-PLL_Primary Tumour 1_TRBC1+ve	1:1	Donor 13	KFN_Hinge_28z
**11D**	T-PLL_Primary Tumour 1_TRBC1+ve	1:1	Donor 13	KFN_CD8STK_28z
**11E**	T-PLL_Primary Tumour 1_TRBC1+ve	1:1	Donor 13	KFN_CD28STK_CD28TM_28z
**11F**	T-PLL_Primary Tumour 1_TRBC1+ve	1:1	Donor 13	aCD19-CAR
**11G**	T-PLL_Primary Tumour 1_TRBC1+ve	1:1	Donor 13	Non-transduced
**12B**	T-PLL_Primary Tumour 1_TRBC1+ve	1:4	Donor 13	KFN_Hinge_28z
**12D**	T-PLL_Primary Tumour 1_TRBC1+ve	1:4	Donor 13	KFN_CD8STK_28z
**12E**	T-PLL_Primary Tumour 1_TRBC1+ve	1:4	Donor 13	KFN_CD28STK_CD28TM_28z
**12F**	T-PLL_Primary Tumour 1_TRBC1+ve	1:4	Donor 13	aCD19-CAR
**12G**	T-PLL_Primary Tumour 1_TRBC1+ve	1:4	Donor 13	Non-transduced

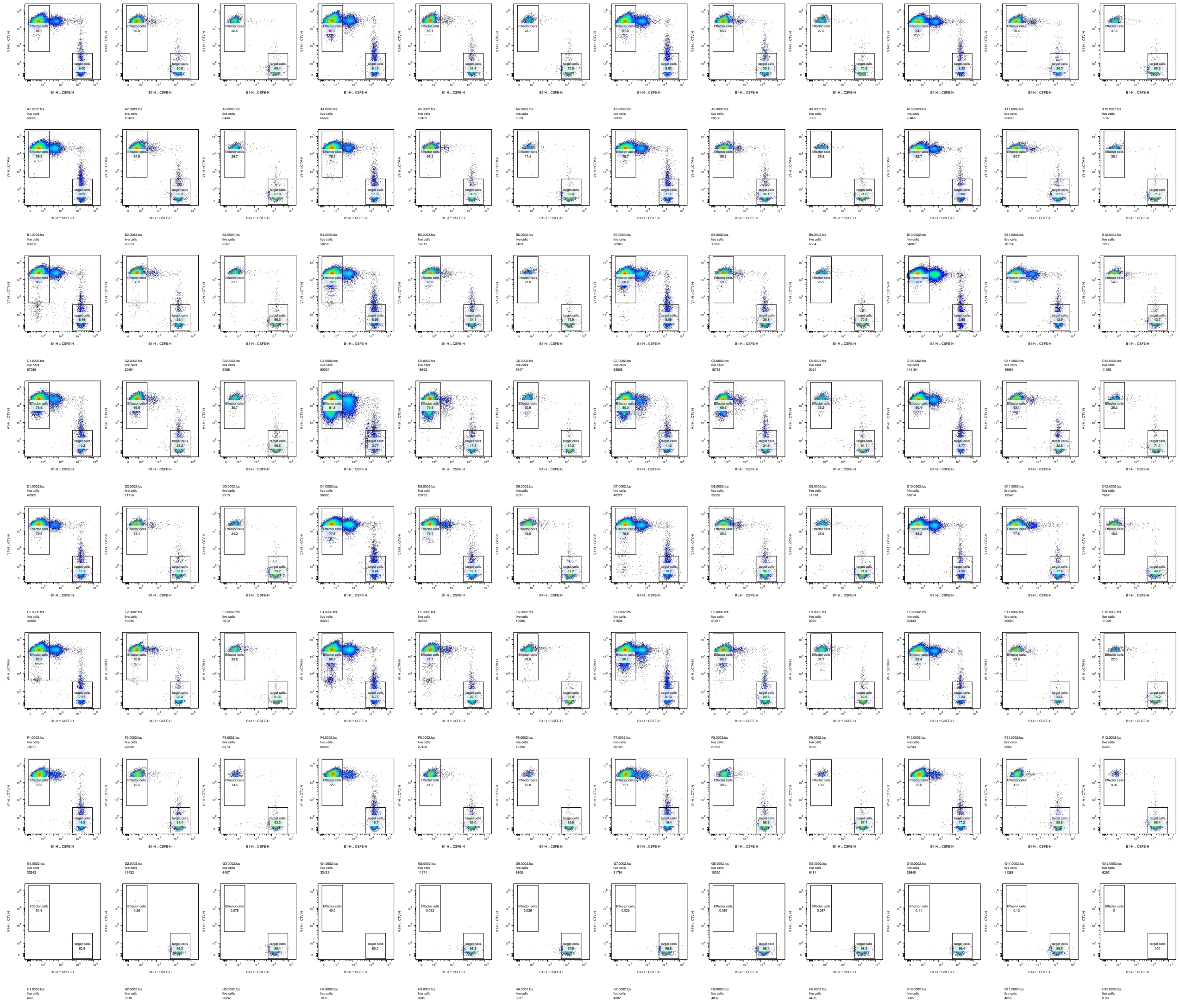

Plate_KFN_vs_PT1647

Well	Target	E:T ratio	Donor	CAR construct
1B	T-PLL_Primary Tumour 1_TRBC1+ve	4:1	Donor 10	KFN_Hinge_28z
1D	T-PLL_Primary Tumour 1_TRBC1+ve	4:1	Donor 10	KFN_CD8STK_28z
1E	T-PLL_Primary Tumour 1_TRBC1+ve	4:1	Donor 10	KFN_CD28STK_CD28TM_28z
1F	T-PLL_Primary Tumour 1_TRBC1+ve	4:1	Donor 10	aCD19-CAR
1G	T-PLL_Primary Tumour 1_TRBC1+ve	4:1	Donor 10	Non-transduced
2B	T-PLL_Primary Tumour 1_TRBC1+ve	1:1	Donor 10	KFN_Hinge_28z
2D	T-PLL_Primary Tumour 1_TRBC1+ve	1:1	Donor 10	KFN_CD8STK_28z
2E	T-PLL_Primary Tumour 1_TRBC1+ve	1:1	Donor 10	KFN_CD28STK_CD28TM_28z
2F	T-PLL_Primary Tumour 1_TRBC1+ve	1:1	Donor 10	aCD19-CAR
2G	T-PLL_Primary Tumour 1_TRBC1+ve	1:1	Donor 10	Non-transduced
3B	T-PLL_Primary Tumour 1_TRBC1+ve	1:4	Donor 10	KFN_Hinge_28z
3D	T-PLL_Primary Tumour 1_TRBC1+ve	1:4	Donor 10	KFN_CD8STK_28z
3E	T-PLL_Primary Tumour 1_TRBC1+ve	1:4	Donor 10	KFN_CD28STK_CD28TM_28z
3F	T-PLL_Primary Tumour 1_TRBC1+ve	1:4	Donor 10	aCD19-CAR
3G	T-PLL_Primary Tumour 1_TRBC1+ve	1:4	Donor 10	Non-transduced
4B	T-PLL_Primary Tumour 1_TRBC1+ve	4:1	Donor 11	KFN_Hinge_28z
4D	T-PLL_Primary Tumour 1_TRBC1+ve	4:1	Donor 11	KFN_CD8STK_28z
4E	T-PLL_Primary Tumour 1_TRBC1+ve	4:1	Donor 11	KFN_CD28STK_CD28TM_28z
4F	T-PLL_Primary Tumour 1_TRBC1+ve	4:1	Donor 11	aCD19-CAR
4G	T-PLL_Primary Tumour 1_TRBC1+ve	4:1	Donor 11	Non-transduced
5B	T-PLL_Primary Tumour 1_TRBC1+ve	1:1	Donor 11	KFN_Hinge_28z
5D	T-PLL_Primary Tumour 1_TRBC1+ve	1:1	Donor 11	KFN_CD8STK_28z
5E	T-PLL_Primary Tumour 1_TRBC1+ve	1:1	Donor 11	KFN_CD28STK_CD28TM_28z
5F	T-PLL_Primary Tumour 1_TRBC1+ve	1:1	Donor 11	aCD19-CAR

**5G**	T-PLL_Primary Tumour 1_TRBC1+ve	1:1	Donor 11	Non-transduced
**6B**	T-PLL_Primary Tumour 1_TRBC1+ve	1:4	Donor 11	KFN_Hinge_28z
**6D**	T-PLL_Primary Tumour 1_TRBC1+ve	1:4	Donor 11	KFN_CD8STK_28z
**6E**	T-PLL_Primary Tumour 1_TRBC1+ve	1:4	Donor 11	KFN_CD28STK_CD28TM_28z
**6F**	T-PLL_Primary Tumour 1_TRBC1+ve	1:4	Donor 11	aCD19-CAR
**6G**	T-PLL_Primary Tumour 1_TRBC1+ve	1:4	Donor 11	Non-transduced
**7B**	T-PLL_Primary Tumour 1_TRBC1+ve	4:1	Donor 12	KFN_Hinge_28z
**7D**	T-PLL_Primary Tumour 1_TRBC1+ve	4:1	Donor 12	KFN_CD8STK_28z
**7E**	T-PLL_Primary Tumour 1_TRBC1+ve	4:1	Donor 12	KFN_CD28STK_CD28TM_28z
**7F**	T-PLL_Primary Tumour 1_TRBC1+ve	4:1	Donor 12	aCD19-CAR
**7G**	T-PLL_Primary Tumour 1_TRBC1+ve	4:1	Donor 12	Non-transduced
**8B**	T-PLL_Primary Tumour 1_TRBC1+ve	1:1	Donor 12	KFN_Hinge_28z
**8D**	T-PLL_Primary Tumour 1_TRBC1+ve	1:1	Donor 12	KFN_CD8STK_28z
**8E**	T-PLL_Primary Tumour 1_TRBC1+ve	1:1	Donor 12	KFN_CD28STK_CD28TM_28z
**8F**	T-PLL_Primary Tumour 1_TRBC1+ve	1:1	Donor 12	aCD19-CAR
**8G**	T-PLL_Primary Tumour 1_TRBC1+ve	1:1	Donor 12	Non-transduced
**9B**	T-PLL_Primary Tumour 1_TRBC1+ve	1:4	Donor 12	KFN_Hinge_28z
**9D**	T-PLL_Primary Tumour 1_TRBC1+ve	1:4	Donor 12	KFN_CD8STK_28z
**9E**	T-PLL_Primary Tumour 1_TRBC1+ve	1:4	Donor 12	KFN_CD28STK_CD28TM_28z
**9F**	T-PLL_Primary Tumour 1_TRBC1+ve	1:4	Donor 12	aCD19-CAR
**9G**	T-PLL_Primary Tumour 1_TRBC1+ve	1:4	Donor 12	Non-transduced
**10B**	T-PLL_Primary Tumour 1_TRBC1+ve	4:1	Donor 13	KFN_Hinge_28z
**10D**	T-PLL_Primary Tumour 1_TRBC1+ve	4:1	Donor 13	KFN_CD8STK_28z
**10E**	T-PLL_Primary Tumour 1_TRBC1+ve	4:1	Donor 13	KFN_CD28STK_CD28TM_28z
**10F**	T-PLL_Primary Tumour 1_TRBC1+ve	4:1	Donor 13	aCD19-CAR

**10G**	T-PLL_Primary Tumour 1_TRBC1+ve	4:1	Donor 13	Non-transduced
**11B**	T-PLL_Primary Tumour 1_TRBC1+ve	1:1	Donor 13	KFN_Hinge_28z
**11D**	T-PLL_Primary Tumour 1_TRBC1+ve	1:1	Donor 13	KFN_CD8STK_28z
**11E**	T-PLL_Primary Tumour 1_TRBC1+ve	1:1	Donor 13	KFN_CD28STK_CD28TM_28z
**11F**	T-PLL_Primary Tumour 1_TRBC1+ve	1:1	Donor 13	aCD19-CAR
**11G**	T-PLL_Primary Tumour 1_TRBC1+ve	1:1	Donor 13	Non-transduced
**12B**	T-PLL_Primary Tumour 1_TRBC1+ve	1:4	Donor 13	KFN_Hinge_28z
**12D**	T-PLL_Primary Tumour 1_TRBC1+ve	1:4	Donor 13	KFN_CD8STK_28z
**12E**	T-PLL_Primary Tumour 1_TRBC1+ve	1:4	Donor 13	KFN_CD28STK_CD28TM_28z
**12F**	T-PLL_Primary Tumour 1_TRBC1+ve	1:4	Donor 13	aCD19-CAR
**12G**	T-PLL_Primary Tumour 1_TRBC1+ve	1:4	Donor 13	Non-transduced

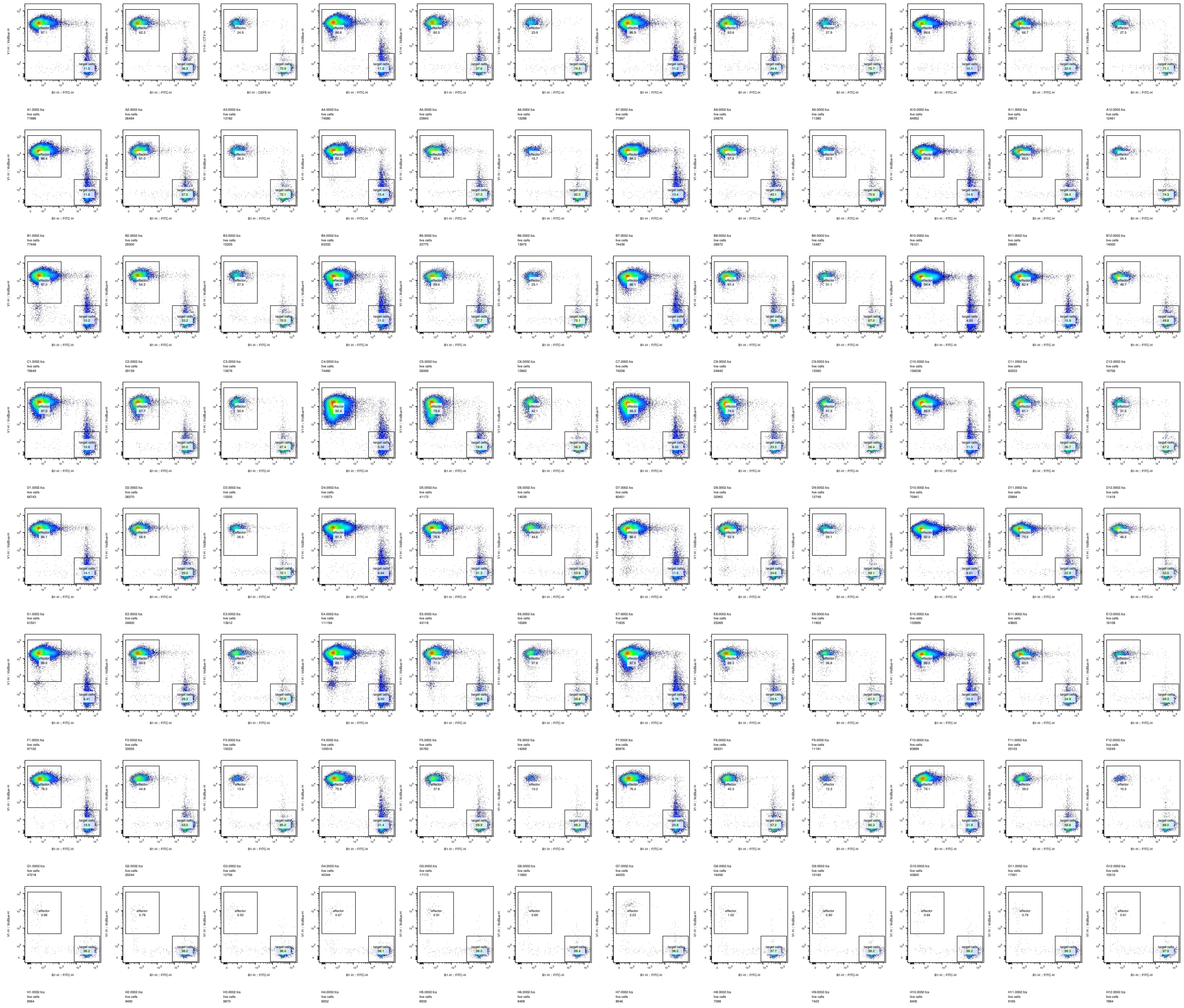

Plate_KFN_vs_PT1718

Well	Target	E:T ratio	Donor	CAR construct
**1B**	T-PLL_Primary Tumour 2_TRBC2+ve	4:1	Donor 10	KFN_Hinge_28z
**1D**	T-PLL_Primary Tumour 2_TRBC2+ve	4:1	Donor 10	KFN_CD8STK_28z
**1E**	T-PLL_Primary Tumour 2_TRBC2+ve	4:1	Donor 10	KFN_CD28STK_CD28TM_28z
**1F**	T-PLL_Primary Tumour 2_TRBC2+ve	4:1	Donor 10	aCD19-CAR
**1G**	T-PLL_Primary Tumour 2_TRBC2+ve	4:1	Donor 10	Non-transduced
**2B**	T-PLL_Primary Tumour 2_TRBC2+ve	1:1	Donor 10	KFN_Hinge_28z
**2D**	T-PLL_Primary Tumour 2_TRBC2+ve	1:1	Donor 10	KFN_CD8STK_28z
**2E**	T-PLL_Primary Tumour 2_TRBC2+ve	1:1	Donor 10	KFN_CD28STK_CD28TM_28z
**2F**	T-PLL_Primary Tumour 2_TRBC2+ve	1:1	Donor 10	aCD19-CAR
**2G**	T-PLL_Primary Tumour 2_TRBC2+ve	1:1	Donor 10	Non-transduced
**3B**	T-PLL_Primary Tumour 2_TRBC2+ve	1:4	Donor 10	KFN_Hinge_28z
**3D**	T-PLL_Primary Tumour 2_TRBC2+ve	1:4	Donor 10	KFN_CD8STK_28z
**3E**	T-PLL_Primary Tumour 2_TRBC2+ve	1:4	Donor 10	KFN_CD28STK_CD28TM_28z
**3F**	T-PLL_Primary Tumour 2_TRBC2+ve	1:4	Donor 10	aCD19-CAR
**3G**	T-PLL_Primary Tumour 2_TRBC2+ve	1:4	Donor 10	Non-transduced
**4B**	T-PLL_Primary Tumour 2_TRBC2+ve	4:1	Donor 11	KFN_Hinge_28z
**4D**	T-PLL_Primary Tumour 2_TRBC2+ve	4:1	Donor 11	KFN_CD8STK_28z
**4E**	T-PLL_Primary Tumour 2_TRBC2+ve	4:1	Donor 11	KFN_CD28STK_CD28TM_28z
**4F**	T-PLL_Primary Tumour 2_TRBC2+ve	4:1	Donor 11	aCD19-CAR
**4G**	T-PLL_Primary Tumour 2_TRBC2+ve	4:1	Donor 11	Non-transduced
**5B**	T-PLL_Primary Tumour 2_TRBC2+ve	1:1	Donor 11	KFN_Hinge_28z
**5D**	T-PLL_Primary Tumour 2_TRBC2+ve	1:1	Donor 11	KFN_CD8STK_28z
**5E**	T-PLL_Primary Tumour 2_TRBC2+ve	1:1	Donor 11	KFN_CD28STK_CD28TM_28z
**5F**	T-PLL_Primary Tumour 2_TRBC2+ve	1:1	Donor 11	aCD19-CAR

**5G**	T-PLL_Primary Tumour 2_TRBC2+ve	1:1	Donor 11	Non-transduced
**6B**	T-PLL_Primary Tumour 2_TRBC2+ve	1:4	Donor 11	KFN_Hinge_28z
**6D**	T-PLL_Primary Tumour 2_TRBC2+ve	1:4	Donor 11	KFN_CD8STK_28z
**6E**	T-PLL_Primary Tumour 2_TRBC2+ve	1:4	Donor 11	KFN_CD28STK_CD28TM_28z
**6F**	T-PLL_Primary Tumour 2_TRBC2+ve	1:4	Donor 11	aCD19-CAR
**6G**	T-PLL_Primary Tumour 2_TRBC2+ve	1:4	Donor 11	Non-transduced
**7B**	T-PLL_Primary Tumour 2_TRBC2+ve	4:1	Donor 12	KFN_Hinge_28z
**7D**	T-PLL_Primary Tumour 2_TRBC2+ve	4:1	Donor 12	KFN_CD8STK_28z
**7E**	T-PLL_Primary Tumour 2_TRBC2+ve	4:1	Donor 12	KFN_CD28STK_CD28TM_28z
**7F**	T-PLL_Primary Tumour 2_TRBC2+ve	4:1	Donor 12	aCD19-CAR
**7G**	T-PLL_Primary Tumour 2_TRBC2+ve	4:1	Donor 12	Non-transduced
**8B**	T-PLL_Primary Tumour 2_TRBC2+ve	1:1	Donor 12	KFN_Hinge_28z
**8D**	T-PLL_Primary Tumour 2_TRBC2+ve	1:1	Donor 12	KFN_CD8STK_28z
**8E**	T-PLL_Primary Tumour 2_TRBC2+ve	1:1	Donor 12	KFN_CD28STK_CD28TM_28z
**8F**	T-PLL_Primary Tumour 2_TRBC2+ve	1:1	Donor 12	aCD19-CAR
**8G**	T-PLL_Primary Tumour 2_TRBC2+ve	1:1	Donor 12	Non-transduced
**9B**	T-PLL_Primary Tumour 2_TRBC2+ve	1:4	Donor 12	KFN_Hinge_28z
**9D**	T-PLL_Primary Tumour 2_TRBC2+ve	1:4	Donor 12	KFN_CD8STK_28z
**9E**	T-PLL_Primary Tumour 2_TRBC2+ve	1:4	Donor 12	KFN_CD28STK_CD28TM_28z
**9F**	T-PLL_Primary Tumour 2_TRBC2+ve	1:4	Donor 12	aCD19-CAR
**9G**	T-PLL_Primary Tumour 2_TRBC2+ve	1:4	Donor 12	Non-transduced
**10B**	T-PLL_Primary Tumour 2_TRBC2+ve	4:1	Donor 13	KFN_Hinge_28z
**10D**	T-PLL_Primary Tumour 2_TRBC2+ve	4:1	Donor 13	KFN_CD8STK_28z
**10E**	T-PLL_Primary Tumour 2_TRBC2+ve	4:1	Donor 13	KFN_CD28STK_CD28TM_28z
**10F**	T-PLL_Primary Tumour 2_TRBC2+ve	4:1	Donor 13	aCD19-CAR

**10G**	T-PLL_Primary Tumour 2_TRBC2+ve	4:1	Donor 13	Non-transduced
**11B**	T-PLL_Primary Tumour 2_TRBC2+ve	1:1	Donor 13	KFN_Hinge_28z
**11D**	T-PLL_Primary Tumour 2_TRBC2+ve	1:1	Donor 13	KFN_CD8STK_28z
**11E**	T-PLL_Primary Tumour 2_TRBC2+ve	1:1	Donor 13	KFN_CD28STK_CD28TM_28z
**11F**	T-PLL_Primary Tumour 2_TRBC2+ve	1:1	Donor 13	aCD19-CAR
**11G**	T-PLL_Primary Tumour 2_TRBC2+ve	1:1	Donor 13	Non-transduced
**12B**	T-PLL_Primary Tumour 2_TRBC2+ve	1:4	Donor 13	KFN_Hinge_28z
**12D**	T-PLL_Primary Tumour 2_TRBC2+ve	1:4	Donor 13	KFN_CD8STK_28z
**12E**	T-PLL_Primary Tumour 2_TRBC2+ve	1:4	Donor 13	KFN_CD28STK_CD28TM_28z
**12F**	T-PLL_Primary Tumour 2_TRBC2+ve	1:4	Donor 13	aCD19-CAR
**12G**	T-PLL_Primary Tumour 2_TRBC2+ve	1:4	Donor 13	Non-transduced

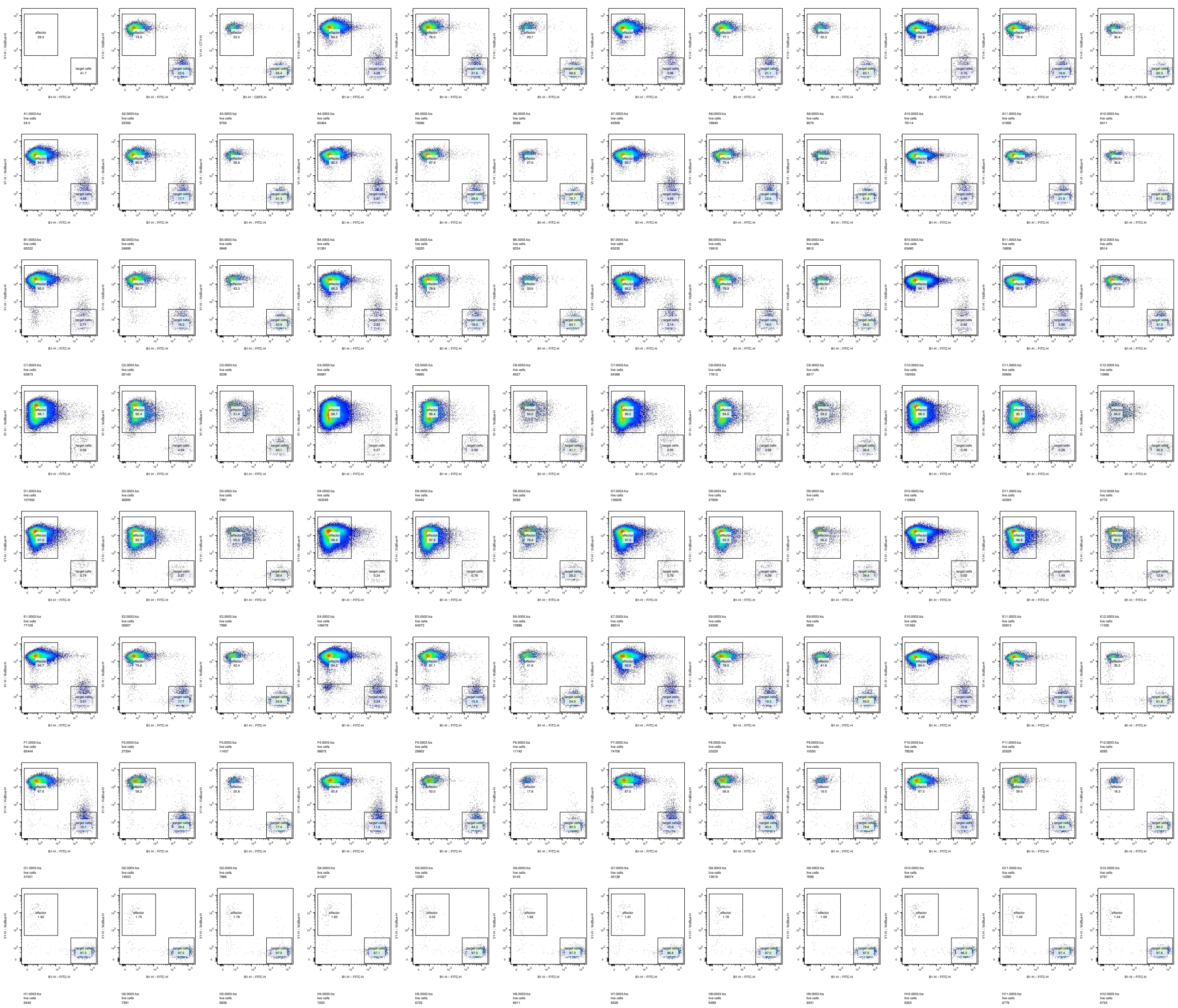

Plate_KFN_vs_PT1795

Well	Target	E:T ratio	Donor	CAR construct
**1B**	T-PLL_Primary Tumour 2_TRBC2+ve	4:1	Donor 10	KFN_Hinge_28z
**1D**	T-PLL_Primary Tumour 2_TRBC2+ve	4:1	Donor 10	KFN_CD8STK_28z
**1E**	T-PLL_Primary Tumour 2_TRBC2+ve	4:1	Donor 10	KFN_CD28STK_CD28TM_28z
**1F**	T-PLL_Primary Tumour 2_TRBC2+ve	4:1	Donor 10	aCD19-CAR
**1G**	T-PLL_Primary Tumour 2_TRBC2+ve	4:1	Donor 10	Non-transduced
**2B**	T-PLL_Primary Tumour 2_TRBC2+ve	1:1	Donor 10	KFN_Hinge_28z
**2D**	T-PLL_Primary Tumour 2_TRBC2+ve	1:1	Donor 10	KFN_CD8STK_28z
**2E**	T-PLL_Primary Tumour 2_TRBC2+ve	1:1	Donor 10	KFN_CD28STK_CD28TM_28z
**2F**	T-PLL_Primary Tumour 2_TRBC2+ve	1:1	Donor 10	aCD19-CAR
**2G**	T-PLL_Primary Tumour 2_TRBC2+ve	1:1	Donor 10	Non-transduced
**3B**	T-PLL_Primary Tumour 2_TRBC2+ve	1:4	Donor 10	KFN_Hinge_28z
**3D**	T-PLL_Primary Tumour 2_TRBC2+ve	1:4	Donor 10	KFN_CD8STK_28z
**3E**	T-PLL_Primary Tumour 2_TRBC2+ve	1:4	Donor 10	KFN_CD28STK_CD28TM_28z
**3F**	T-PLL_Primary Tumour 2_TRBC2+ve	1:4	Donor 10	aCD19-CAR
**3G**	T-PLL_Primary Tumour 2_TRBC2+ve	1:4	Donor 10	Non-transduced
**4B**	T-PLL_Primary Tumour 2_TRBC2+ve	4:1	Donor 11	KFN_Hinge_28z
**4D**	T-PLL_Primary Tumour 2_TRBC2+ve	4:1	Donor 11	KFN_CD8STK_28z
**4E**	T-PLL_Primary Tumour 2_TRBC2+ve	4:1	Donor 11	KFN_CD28STK_CD28TM_28z
**4F**	T-PLL_Primary Tumour 2_TRBC2+ve	4:1	Donor 11	aCD19-CAR
**4G**	T-PLL_Primary Tumour 2_TRBC2+ve	4:1	Donor 11	Non-transduced
**5B**	T-PLL_Primary Tumour 2_TRBC2+ve	1:1	Donor 11	KFN_Hinge_28z
**5D**	T-PLL_Primary Tumour 2_TRBC2+ve	1:1	Donor 11	KFN_CD8STK_28z
**5E**	T-PLL_Primary Tumour 2_TRBC2+ve	1:1	Donor 11	KFN_CD28STK_CD28TM_28z
**5F**	T-PLL_Primary Tumour 2_TRBC2+ve	1:1	Donor 11	aCD19-CAR

**5G**	T-PLL_Primary Tumour 2_TRBC2+ve	1:1	Donor 11	Non-transduced
**6B**	T-PLL_Primary Tumour 2_TRBC2+ve	1:4	Donor 11	KFN_Hinge_28z
**6D**	T-PLL_Primary Tumour 2_TRBC2+ve	1:4	Donor 11	KFN_CD8STK_28z
**6E**	T-PLL_Primary Tumour 2_TRBC2+ve	1:4	Donor 11	KFN_CD28STK_CD28TM_28z
**6F**	T-PLL_Primary Tumour 2_TRBC2+ve	1:4	Donor 11	aCD19-CAR
**6G**	T-PLL_Primary Tumour 2_TRBC2+ve	1:4	Donor 11	Non-transduced
**7B**	T-PLL_Primary Tumour 2_TRBC2+ve	4:1	Donor 12	KFN_Hinge_28z
**7D**	T-PLL_Primary Tumour 2_TRBC2+ve	4:1	Donor 12	KFN_CD8STK_28z
**7E**	T-PLL_Primary Tumour 2_TRBC2+ve	4:1	Donor 12	KFN_CD28STK_CD28TM_28z
**7F**	T-PLL_Primary Tumour 2_TRBC2+ve	4:1	Donor 12	aCD19-CAR
**7G**	T-PLL_Primary Tumour 2_TRBC2+ve	4:1	Donor 12	Non-transduced
**8B**	T-PLL_Primary Tumour 2_TRBC2+ve	1:1	Donor 12	KFN_Hinge_28z
**8D**	T-PLL_Primary Tumour 2_TRBC2+ve	1:1	Donor 12	KFN_CD8STK_28z
**8E**	T-PLL_Primary Tumour 2_TRBC2+ve	1:1	Donor 12	KFN_CD28STK_CD28TM_28z
**8F**	T-PLL_Primary Tumour 2_TRBC2+ve	1:1	Donor 12	aCD19-CAR
**8G**	T-PLL_Primary Tumour 2_TRBC2+ve	1:1	Donor 12	Non-transduced
**9B**	T-PLL_Primary Tumour 2_TRBC2+ve	1:4	Donor 12	KFN_Hinge_28z
**9D**	T-PLL_Primary Tumour 2_TRBC2+ve	1:4	Donor 12	KFN_CD8STK_28z
**9E**	T-PLL_Primary Tumour 2_TRBC2+ve	1:4	Donor 12	KFN_CD28STK_CD28TM_28z
**9F**	T-PLL_Primary Tumour 2_TRBC2+ve	1:4	Donor 12	aCD19-CAR
**9G**	T-PLL_Primary Tumour 2_TRBC2+ve	1:4	Donor 12	Non-transduced
**10B**	T-PLL_Primary Tumour 2_TRBC2+ve	4:1	Donor 13	KFN_Hinge_28z
**10D**	T-PLL_Primary Tumour 2_TRBC2+ve	4:1	Donor 13	KFN_CD8STK_28z
**10E**	T-PLL_Primary Tumour 2_TRBC2+ve	4:1	Donor 13	KFN_CD28STK_CD28TM_28z
**10F**	T-PLL_Primary Tumour 2_TRBC2+ve	4:1	Donor 13	aCD19-CAR

**10G**	T-PLL_Primary Tumour 2_TRBC2+ve	4:1	Donor 13	Non-transduced
**11B**	T-PLL_Primary Tumour 2_TRBC2+ve	1:1	Donor 13	KFN_Hinge_28z
**11D**	T-PLL_Primary Tumour 2_TRBC2+ve	1:1	Donor 13	KFN_CD8STK_28z
**11E**	T-PLL_Primary Tumour 2_TRBC2+ve	1:1	Donor 13	KFN_CD28STK_CD28TM_28z
**11F**	T-PLL_Primary Tumour 2_TRBC2+ve	1:1	Donor 13	aCD19-CAR
**11G**	T-PLL_Primary Tumour 2_TRBC2+ve	1:1	Donor 13	Non-transduced
**12B**	T-PLL_Primary Tumour 2_TRBC2+ve	1:4	Donor 13	KFN_Hinge_28z
**12D**	T-PLL_Primary Tumour 2_TRBC2+ve	1:4	Donor 13	KFN_CD8STK_28z
**12E**	T-PLL_Primary Tumour 2_TRBC2+ve	1:4	Donor 13	KFN_CD28STK_CD28TM_28z
**12F**	T-PLL_Primary Tumour 2_TRBC2+ve	1:4	Donor 13	aCD19-CAR
**12G**	T-PLL_Primary Tumour 2_TRBC2+ve	1:4	Donor 13	Non-transduced

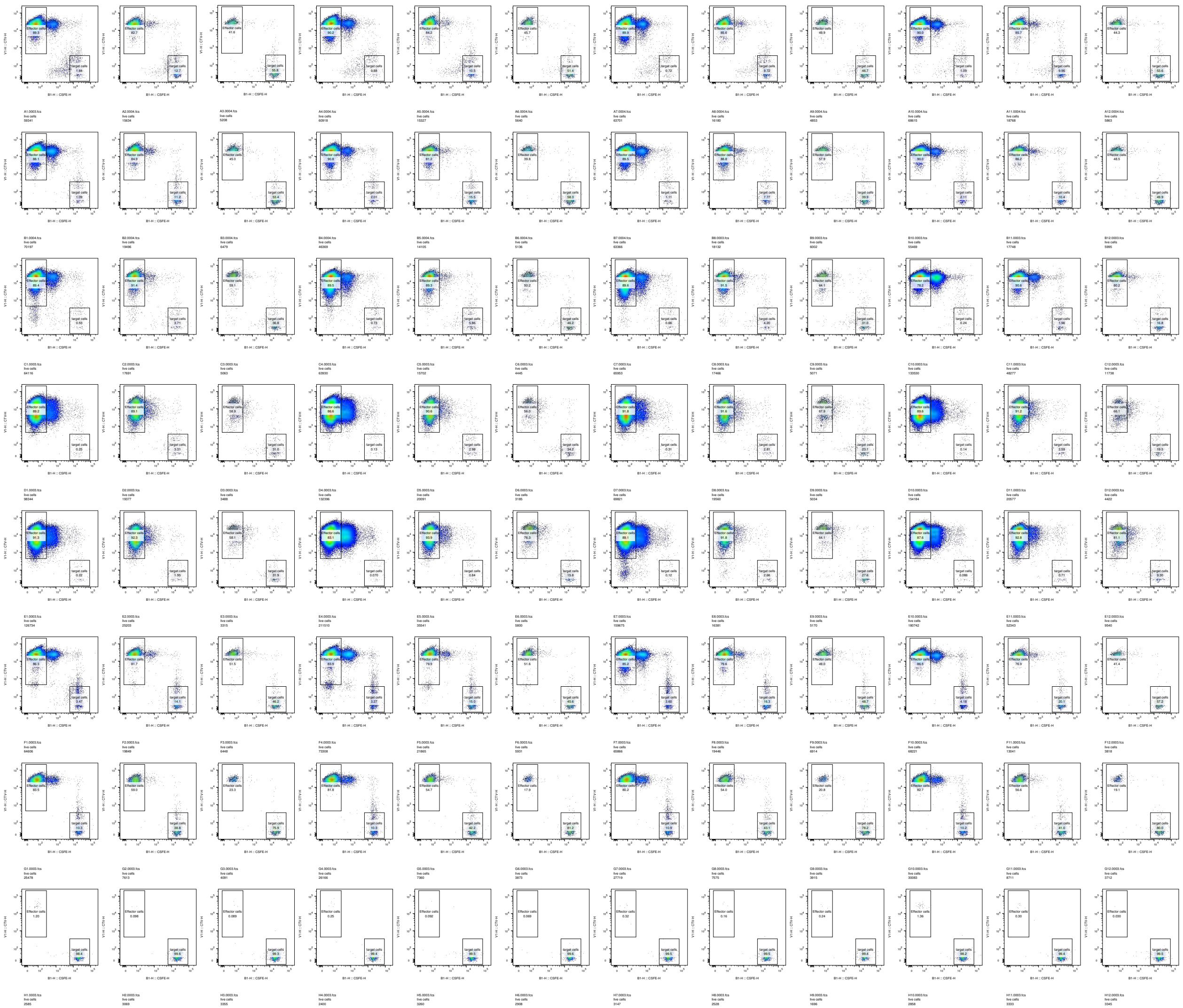

Plate_JOVI_vs_HDMAR

**Well**	**Target**	**E:T ratio**	**Donor**	**CAR construct**
**1A**	HDMAR	1:4	Donor 10	JOVI_Hinge_41bbz
**1D**	HDMAR	1:4	Donor 10	JOVI_CD8STK_28z
**1E**	HDMAR	1:4	Donor 10	JOVI_CD28STK_CD28TM_28z
**1F**	HDMAR	1:4	Donor 10	mJOVI_Hinge_41bbz
**1G**	HDMAR	1:4	Donor 10	aCD19-CAR
**1H**	HDMAR	1:4	Donor 10	Non-transduced
**2A**	HDMAR	1:8	Donor 10	JOVI_Hinge_41bbz
**2D**	HDMAR	1:8	Donor 10	JOVI_CD8STK_28z
**2E**	HDMAR	1:8	Donor 10	JOVI_CD28STK_CD28TM_28z
**2F**	HDMAR	1:8	Donor 10	mJOVI_Hinge_41bbz
**2G**	HDMAR	1:8	Donor 10	aCD19-CAR
**2H**	HDMAR	1:8	Donor 10	Non-transduced
**4A**	HDMAR	1:4	Donor 11	JOVI_Hinge_41bbz
**4D**	HDMAR	1:4	Donor 11	JOVI_CD8STK_28z
**4E**	HDMAR	1:4	Donor 11	JOVI_CD28STK_CD28TM_28z
**4F**	HDMAR	1:4	Donor 11	mJOVI_Hinge_41bbz
**4G**	HDMAR	1:4	Donor 11	aCD19-CAR
**4H**	HDMAR	1:4	Donor 11	Non-transduced
**5A**	HDMAR	1:8	Donor 11	JOVI_Hinge_41bbz
**5D**	HDMAR	1:8	Donor 11	JOVI_CD8STK_28z
**5E**	HDMAR	1:8	Donor 11	JOVI_CD28STK_CD28TM_28z
**5F**	HDMAR	1:8	Donor 11	mJOVI_Hinge_41bbz
**5G**	HDMAR	1:8	Donor 11	aCD19-CAR
**5H**	HDMAR	1:8	Donor 11	Non-transduced
**7A**	HDMAR	1:4	Donor 12	JOVI_Hinge_41bbz
**7D**	HDMAR	1:4	Donor 12	JOVI_CD8STK_28z
**7E**	HDMAR	1:4	Donor 12	JOVI_CD28STK_CD28TM_28z
**7F**	HDMAR	1:4	Donor 12	mJOVI_Hinge_41bbz
**7G**	HDMAR	1:4	Donor 12	aCD19-CAR
**7H**	HDMAR	1:4	Donor 12	Non-transduced
**8A**	HDMAR	1:8	Donor 12	JOVI_Hinge_41bbz
**8D**	HDMAR	1:8	Donor 12	JOVI_CD8STK_28z
**8E**	HDMAR	1:8	Donor 12	JOVI_CD28STK_CD28TM_28z
**8F**	HDMAR	1:8	Donor 12	mJOVI_Hinge_41bbz
**8G**	HDMAR	1:8	Donor 12	aCD19-CAR
**8H**	HDMAR	1:8	Donor 12	Non-transduced
**10A**	HDMAR	1:4	Donor 13	JOVI_Hinge_41bbz
**10D**	HDMAR	1:4	Donor 13	JOVI_CD8STK_28z
**10E**	HDMAR	1:4	Donor 13	JOVI_CD28STK_CD28TM_28z
**10F**	HDMAR	1:4	Donor 13	mJOVI_Hinge_41bbz
**10G**	HDMAR	1:4	Donor 13	aCD19-CAR
**10H**	HDMAR	1:4	Donor 13	Non-transduced
**11A**	HDMAR	1:8	Donor 13	JOVI_Hinge_41bbz
**11D**	HDMAR	1:8	Donor 13	JOVI_CD8STK_28z
**11E**	HDMAR	1:8	Donor 13	JOVI_CD28STK_CD28TM_28z
**11F**	HDMAR	1:8	Donor 13	mJOVI_Hinge_41bbz
**11G**	HDMAR	1:8	Donor 13	aCD19-CAR
**11H**	HDMAR	1:8	Donor 13	Non-transduced

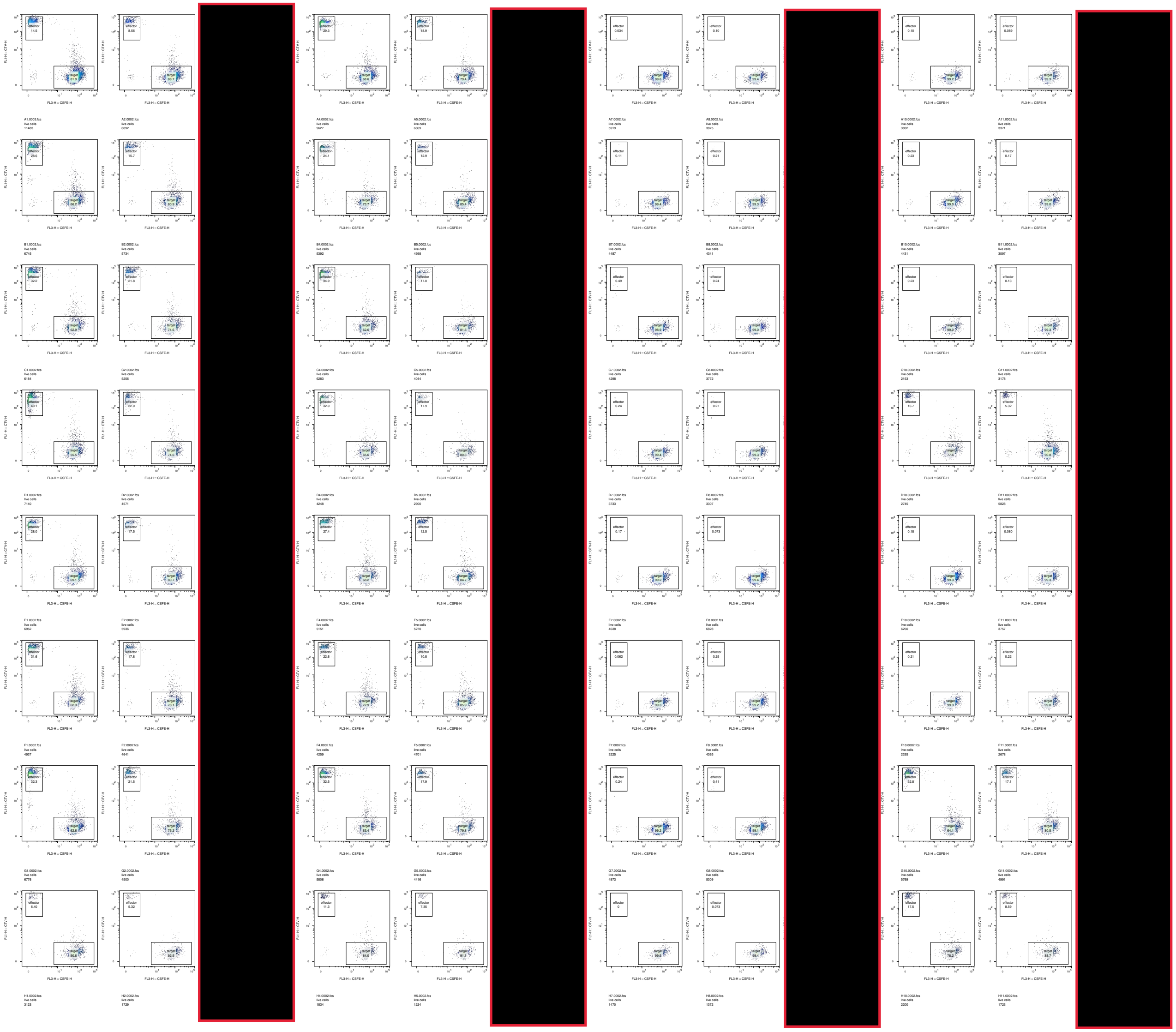

Plate_JOVI_vs_PT1707

Well	Target	E:T ratio	Donor	CAR construct
**1A**	T-PLL_Primary Tumour 1_TRBC1+ve	4:1	Donor 10	JOVI_Hinge_41bbz
**1D**	T-PLL_Primary Tumour 1_TRBC1+ve	4:1	Donor 10	JOVI_CD8STK_28z
**1E**	T-PLL_Primary Tumour 1_TRBC1+ve	4:1	Donor 10	JOVI_CD28STK_CD28TM_28z
**1G**	T-PLL_Primary Tumour 1_TRBC1+ve	4:1	Donor 10	aCD19-CAR
**1H**	T-PLL_Primary Tumour 1_TRBC1+ve	4:1	Donor 10	Non-transduced
**2A**	T-PLL_Primary Tumour 1_TRBC1+ve	1:1	Donor 10	JOVI_Hinge_41bbz
**2D**	T-PLL_Primary Tumour 1_TRBC1+ve	1:1	Donor 10	JOVI_CD8STK_28z
**2E**	T-PLL_Primary Tumour 1_TRBC1+ve	1:1	Donor 10	JOVI_CD28STK_CD28TM_28z
**2G**	T-PLL_Primary Tumour 1_TRBC1+ve	1:1	Donor 10	aCD19-CAR
**2H**	T-PLL_Primary Tumour 1_TRBC1+ve	1:1	Donor 10	Non-transduced
**3A**	T-PLL_Primary Tumour 1_TRBC1+ve	1:4	Donor 10	JOVI_Hinge_41bbz
**3D**	T-PLL_Primary Tumour 1_TRBC1+ve	1:4	Donor 10	JOVI_CD8STK_28z
**3E**	T-PLL_Primary Tumour 1_TRBC1+ve	1:4	Donor 10	JOVI_CD28STK_CD28TM_28z
**3G**	T-PLL_Primary Tumour 1_TRBC1+ve	1:4	Donor 10	aCD19-CAR
**3H**	T-PLL_Primary Tumour 1_TRBC1+ve	1:4	Donor 10	Non-transduced
**4A**	T-PLL_Primary Tumour 1_TRBC1+ve	4:1	Donor 11	JOVI_Hinge_41bbz
**4D**	T-PLL_Primary Tumour 1_TRBC1+ve	4:1	Donor 11	JOVI_CD8STK_28z
**4E**	T-PLL_Primary Tumour 1_TRBC1+ve	4:1	Donor 11	JOVI_CD28STK_CD28TM_28z
**4G**	T-PLL_Primary Tumour 1_TRBC1+ve	4:1	Donor 11	aCD19-CAR
**4H**	T-PLL_Primary Tumour 1_TRBC1+ve	4:1	Donor 11	Non-transduced
**5A**	T-PLL_Primary Tumour 1_TRBC1+ve	1:1	Donor 11	JOVI_Hinge_41bbz
**5D**	T-PLL_Primary Tumour 1_TRBC1+ve	1:1	Donor 11	JOVI_CD8STK_28z
**5E**	T-PLL_Primary Tumour 1_TRBC1+ve	1:1	Donor 11	JOVI_CD28STK_CD28TM_28z
**5G**	T-PLL_Primary Tumour 1_TRBC1+ve	1:1	Donor 11	aCD19-CAR

**5H**	T-PLL_Primary Tumour 1_TRBC1+ve	1:1	Donor 11	Non-transduced
**6A**	T-PLL_Primary Tumour 1_TRBC1+ve	1:4	Donor 11	JOVI_Hinge_41bbz
**6D**	T-PLL_Primary Tumour 1_TRBC1+ve	1:4	Donor 11	JOVI_CD8STK_28z
**6E**	T-PLL_Primary Tumour 1_TRBC1+ve	1:4	Donor 11	JOVI_CD28STK_CD28TM_28z
**6G**	T-PLL_Primary Tumour 1_TRBC1+ve	1:4	Donor 11	aCD19-CAR
**6H**	T-PLL_Primary Tumour 1_TRBC1+ve	1:4	Donor 11	Non-transduced
**7A**	T-PLL_Primary Tumour 1_TRBC1+ve	4:1	Donor 12	JOVI_Hinge_41bbz
**7D**	T-PLL_Primary Tumour 1_TRBC1+ve	4:1	Donor 12	JOVI_CD8STK_28z
**7E**	T-PLL_Primary Tumour 1_TRBC1+ve	4:1	Donor 12	JOVI_CD28STK_CD28TM_28z
**7G**	T-PLL_Primary Tumour 1_TRBC1+ve	4:1	Donor 12	aCD19-CAR
**7H**	T-PLL_Primary Tumour 1_TRBC1+ve	4:1	Donor 12	Non-transduced
**8A**	T-PLL_Primary Tumour 1_TRBC1+ve	1:1	Donor 12	JOVI_Hinge_41bbz
**8D**	T-PLL_Primary Tumour 1_TRBC1+ve	1:1	Donor 12	JOVI_CD8STK_28z
**8E**	T-PLL_Primary Tumour 1_TRBC1+ve	1:1	Donor 12	JOVI_CD28STK_CD28TM_28z
**8G**	T-PLL_Primary Tumour 1_TRBC1+ve	1:1	Donor 12	aCD19-CAR
**8H**	T-PLL_Primary Tumour 1_TRBC1+ve	1:1	Donor 12	Non-transduced
**9A**	T-PLL_Primary Tumour 1_TRBC1+ve	1:4	Donor 12	JOVI_Hinge_41bbz
**9D**	T-PLL_Primary Tumour 1_TRBC1+ve	1:4	Donor 12	JOVI_CD8STK_28z
**9E**	T-PLL_Primary Tumour 1_TRBC1+ve	1:4	Donor 12	JOVI_CD28STK_CD28TM_28z
**9G**	T-PLL_Primary Tumour 1_TRBC1+ve	1:4	Donor 12	aCD19-CAR
**9H**	T-PLL_Primary Tumour 1_TRBC1+ve	1:4	Donor 12	Non-transduced
**10A**	T-PLL_Primary Tumour 1_TRBC1+ve	4:1	Donor 13	JOVI_Hinge_41bbz
**10D**	T-PLL_Primary Tumour 1_TRBC1+ve	4:1	Donor 13	JOVI_CD8STK_28z
**10E**	T-PLL_Primary Tumour 1_TRBC1+ve	4:1	Donor 13	JOVI_CD28STK_CD28TM_28z
**10G**	T-PLL_Primary Tumour 1_TRBC1+ve	4:1	Donor 13	aCD19-CAR

**10H**	T-PLL_Primary Tumour 1_TRBC1+ve	4:1	Donor 13	Non-transduced
**11A**	T-PLL_Primary Tumour 1_TRBC1+ve	1:1	Donor 13	JOVI_Hinge_41bbz
**11D**	T-PLL_Primary Tumour 1_TRBC1+ve	1:1	Donor 13	JOVI_CD8STK_28z
**11E**	T-PLL_Primary Tumour 1_TRBC1+ve	1:1	Donor 13	JOVI_CD28STK_CD28TM_28z
**11G**	T-PLL_Primary Tumour 1_TRBC1+ve	1:1	Donor 13	aCD19-CAR
**11H**	T-PLL_Primary Tumour 1_TRBC1+ve	1:1	Donor 13	Non-transduced
**12A**	T-PLL_Primary Tumour 1_TRBC1+ve	1:4	Donor 13	JOVI_Hinge_41bbz
**12D**	T-PLL_Primary Tumour 1_TRBC1+ve	1:4	Donor 13	JOVI_CD8STK_28z
**12E**	T-PLL_Primary Tumour 1_TRBC1+ve	1:4	Donor 13	JOVI_CD28STK_CD28TM_28z
**12G**	T-PLL_Primary Tumour 1_TRBC1+ve	1:4	Donor 13	aCD19-CAR
**12H**	T-PLL_Primary Tumour 1_TRBC1+ve	1:4	Donor 13	Non-transduced

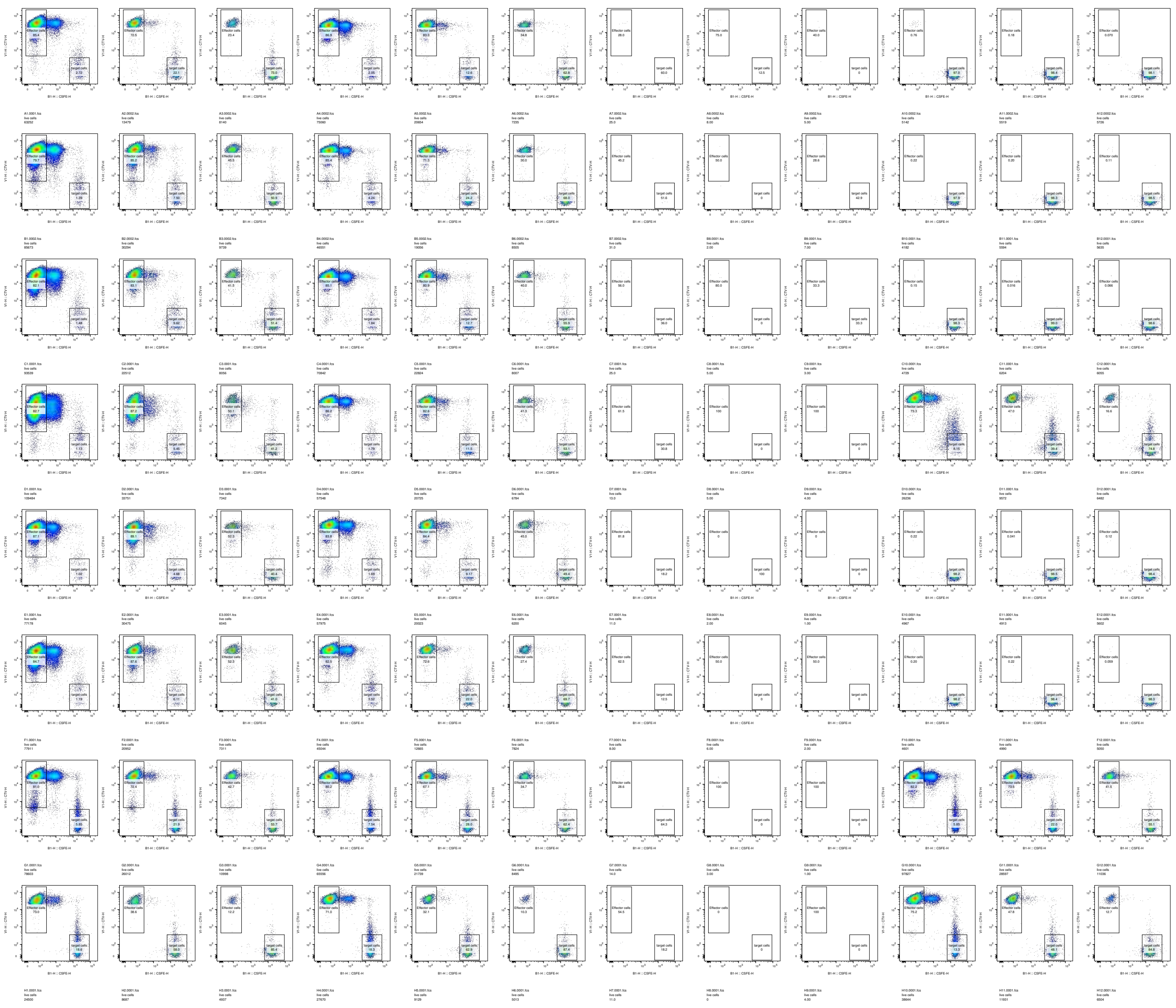

Plate_JOVI_vs_PT1647

Well	Target	E:T ratio	Donor	CAR construct
**1A**	T-PLL_Primary Tumour 1_TRBC1+ve	4:1	Donor 10	JOVI_Hinge_41bbz
**1D**	T-PLL_Primary Tumour 1_TRBC1+ve	4:1	Donor 10	JOVI_CD8STK_28z
**1E**	T-PLL_Primary Tumour 1_TRBC1+ve	4:1	Donor 10	JOVI_CD28STK_CD28TM_28z
**1G**	T-PLL_Primary Tumour 1_TRBC1+ve	4:1	Donor 10	aCD19-CAR
**1H**	T-PLL_Primary Tumour 1_TRBC1+ve	4:1	Donor 10	Non-transduced
**2A**	T-PLL_Primary Tumour 1_TRBC1+ve	1:1	Donor 10	JOVI_Hinge_41bbz
**2D**	T-PLL_Primary Tumour 1_TRBC1+ve	1:1	Donor 10	JOVI_CD8STK_28z
**2E**	T-PLL_Primary Tumour 1_TRBC1+ve	1:1	Donor 10	JOVI_CD28STK_CD28TM_28z
**2G**	T-PLL_Primary Tumour 1_TRBC1+ve	1:1	Donor 10	aCD19-CAR
**2H**	T-PLL_Primary Tumour 1_TRBC1+ve	1:1	Donor 10	Non-transduced
**3A**	T-PLL_Primary Tumour 1_TRBC1+ve	1:4	Donor 10	JOVI_Hinge_41bbz
**3D**	T-PLL_Primary Tumour 1_TRBC1+ve	1:4	Donor 10	JOVI_CD8STK_28z
**3E**	T-PLL_Primary Tumour 1_TRBC1+ve	1:4	Donor 10	JOVI_CD28STK_CD28TM_28z
**3G**	T-PLL_Primary Tumour 1_TRBC1+ve	1:4	Donor 10	aCD19-CAR
**3H**	T-PLL_Primary Tumour 1_TRBC1+ve	1:4	Donor 10	Non-transduced
**4A**	T-PLL_Primary Tumour 1_TRBC1+ve	4:1	Donor 11	JOVI_Hinge_41bbz
**4D**	T-PLL_Primary Tumour 1_TRBC1+ve	4:1	Donor 11	JOVI_CD8STK_28z
**4E**	T-PLL_Primary Tumour 1_TRBC1+ve	4:1	Donor 11	JOVI_CD28STK_CD28TM_28z
**4G**	T-PLL_Primary Tumour 1_TRBC1+ve	4:1	Donor 11	aCD19-CAR
**4H**	T-PLL_Primary Tumour 1_TRBC1+ve	4:1	Donor 11	Non-transduced
**5A**	T-PLL_Primary Tumour 1_TRBC1+ve	1:1	Donor 11	JOVI_Hinge_41bbz
**5D**	T-PLL_Primary Tumour 1_TRBC1+ve	1:1	Donor 11	JOVI_CD8STK_28z
**5E**	T-PLL_Primary Tumour 1_TRBC1+ve	1:1	Donor 11	JOVI_CD28STK_CD28TM_28z
**5G**	T-PLL_Primary Tumour 1_TRBC1+ve	1:1	Donor 11	aCD19-CAR

**5H**	T-PLL_Primary Tumour 1_TRBC1+ve	1:1	Donor 11	Non-transduced
**6A**	T-PLL_Primary Tumour 1_TRBC1+ve	1:4	Donor 11	JOVI_Hinge_41bbz
**6D**	T-PLL_Primary Tumour 1_TRBC1+ve	1:4	Donor 11	JOVI_CD8STK_28z
**6E**	T-PLL_Primary Tumour 1_TRBC1+ve	1:4	Donor 11	JOVI_CD28STK_CD28TM_28z
**6G**	T-PLL_Primary Tumour 1_TRBC1+ve	1:4	Donor 11	aCD19-CAR
**6H**	T-PLL_Primary Tumour 1_TRBC1+ve	1:4	Donor 11	Non-transduced
**7A**	T-PLL_Primary Tumour 1_TRBC1+ve	4:1	Donor 12	JOVI_Hinge_41bbz
**7D**	T-PLL_Primary Tumour 1_TRBC1+ve	4:1	Donor 12	JOVI_CD8STK_28z
**7E**	T-PLL_Primary Tumour 1_TRBC1+ve	4:1	Donor 12	JOVI_CD28STK_CD28TM_28z
**7G**	T-PLL_Primary Tumour 1_TRBC1+ve	4:1	Donor 12	aCD19-CAR
**7H**	T-PLL_Primary Tumour 1_TRBC1+ve	4:1	Donor 12	Non-transduced
**8A**	T-PLL_Primary Tumour 1_TRBC1+ve	1:1	Donor 12	JOVI_Hinge_41bbz
**8D**	T-PLL_Primary Tumour 1_TRBC1+ve	1:1	Donor 12	JOVI_CD8STK_28z
**8E**	T-PLL_Primary Tumour 1_TRBC1+ve	1:1	Donor 12	JOVI_CD28STK_CD28TM_28z
**8G**	T-PLL_Primary Tumour 1_TRBC1+ve	1:1	Donor 12	aCD19-CAR
**8H**	T-PLL_Primary Tumour 1_TRBC1+ve	1:1	Donor 12	Non-transduced
**9A**	T-PLL_Primary Tumour 1_TRBC1+ve	1:4	Donor 12	JOVI_Hinge_41bbz
**9D**	T-PLL_Primary Tumour 1_TRBC1+ve	1:4	Donor 12	JOVI_CD8STK_28z
**9E**	T-PLL_Primary Tumour 1_TRBC1+ve	1:4	Donor 12	JOVI_CD28STK_CD28TM_28z
**9G**	T-PLL_Primary Tumour 1_TRBC1+ve	1:4	Donor 12	aCD19-CAR
**9H**	T-PLL_Primary Tumour 1_TRBC1+ve	1:4	Donor 12	Non-transduced
**10A**	T-PLL_Primary Tumour 1_TRBC1+ve	4:1	Donor 13	JOVI_Hinge_41bbz
**10D**	T-PLL_Primary Tumour 1_TRBC1+ve	4:1	Donor 13	JOVI_CD8STK_28z
**10E**	T-PLL_Primary Tumour 1_TRBC1+ve	4:1	Donor 13	JOVI_CD28STK_CD28TM_28z
**10G**	T-PLL_Primary Tumour 1_TRBC1+ve	4:1	Donor 13	aCD19-CAR

**10H**	T-PLL_Primary Tumour 1_TRBC1+ve	4:1	Donor 13	Non-transduced
**11A**	T-PLL_Primary Tumour 1_TRBC1+ve	1:1	Donor 13	JOVI_Hinge_41bbz
**11D**	T-PLL_Primary Tumour 1_TRBC1+ve	1:1	Donor 13	JOVI_CD8STK_28z
**11E**	T-PLL_Primary Tumour 1_TRBC1+ve	1:1	Donor 13	JOVI_CD28STK_CD28TM_28z
**11G**	T-PLL_Primary Tumour 1_TRBC1+ve	1:1	Donor 13	aCD19-CAR
**11H**	T-PLL_Primary Tumour 1_TRBC1+ve	1:1	Donor 13	Non-transduced
**12A**	T-PLL_Primary Tumour 1_TRBC1+ve	1:4	Donor 13	JOVI_Hinge_41bbz
**12D**	T-PLL_Primary Tumour 1_TRBC1+ve	1:4	Donor 13	JOVI_CD8STK_28z
**12E**	T-PLL_Primary Tumour 1_TRBC1+ve	1:4	Donor 13	JOVI_CD28STK_CD28TM_28z
**12G**	T-PLL_Primary Tumour 1_TRBC1+ve	1:4	Donor 13	aCD19-CAR
**12H**	T-PLL_Primary Tumour 1_TRBC1+ve	1:4	Donor 13	Non-transduced

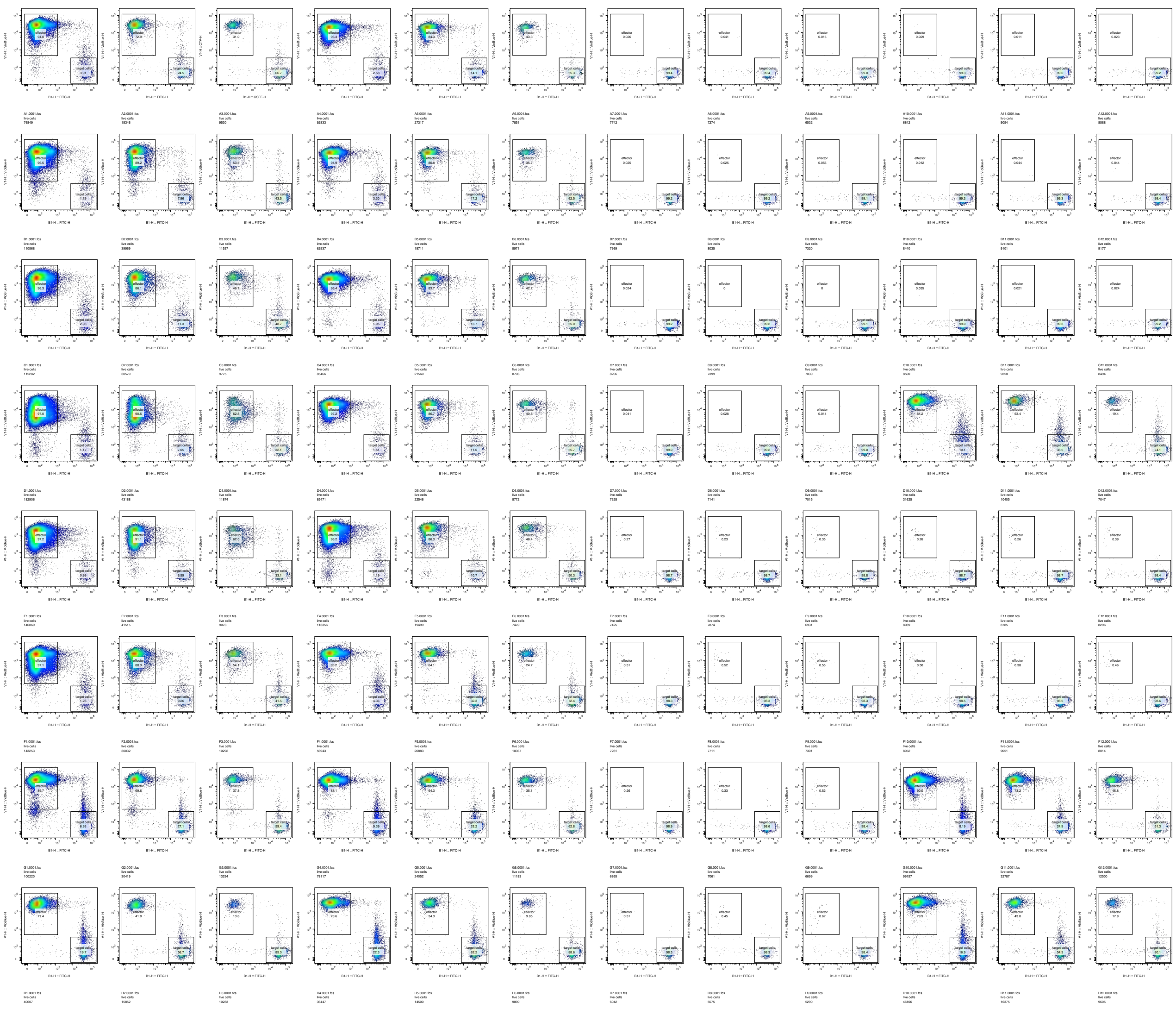

Plate_JOVI_vs_PT1795

Well	Target	E:T ratio	Donor	CAR construct
**1A**	T-PLL_Primary Tumour 2_TRBC2+ve	4:1	Donor 10	JOVI_Hinge_41bbz
**1D**	T-PLL_Primary Tumour 2_TRBC2+ve	4:1	Donor 10	JOVI_CD8STK_28z
**1E**	T-PLL_Primary Tumour 2_TRBC2+ve	4:1	Donor 10	JOVI_CD28STK_CD28TM_28z
**1G**	T-PLL_Primary Tumour 2_TRBC2+ve	4:1	Donor 10	aCD19-CAR
**1H**	T-PLL_Primary Tumour 2_TRBC2+ve	4:1	Donor 10	Non-transduced
**2A**	T-PLL_Primary Tumour 2_TRBC2+ve	1:1	Donor 10	JOVI_Hinge_41bbz
**2D**	T-PLL_Primary Tumour 2_TRBC2+ve	1:1	Donor 10	JOVI_CD8STK_28z
**2E**	T-PLL_Primary Tumour 2_TRBC2+ve	1:1	Donor 10	JOVI_CD28STK_CD28TM_28z
**2G**	T-PLL_Primary Tumour 2_TRBC2+ve	1:1	Donor 10	aCD19-CAR
**2H**	T-PLL_Primary Tumour 2_TRBC2+ve	1:1	Donor 10	Non-transduced
**3A**	T-PLL_Primary Tumour 2_TRBC2+ve	1:4	Donor 10	JOVI_Hinge_41bbz
**3D**	T-PLL_Primary Tumour 2_TRBC2+ve	1:4	Donor 10	JOVI_CD8STK_28z
**3E**	T-PLL_Primary Tumour 2_TRBC2+ve	1:4	Donor 10	JOVI_CD28STK_CD28TM_28z
**3G**	T-PLL_Primary Tumour 2_TRBC2+ve	1:4	Donor 10	aCD19-CAR
**3H**	T-PLL_Primary Tumour 2_TRBC2+ve	1:4	Donor 10	Non-transduced
**4A**	T-PLL_Primary Tumour 2_TRBC2+ve	4:1	Donor 11	JOVI_Hinge_41bbz
**4D**	T-PLL_Primary Tumour 2_TRBC2+ve	4:1	Donor 11	JOVI_CD8STK_28z
**4E**	T-PLL_Primary Tumour 2_TRBC2+ve	4:1	Donor 11	JOVI_CD28STK_CD28TM_28z
**4G**	T-PLL_Primary Tumour 2_TRBC2+ve	4:1	Donor 11	aCD19-CAR
**4H**	T-PLL_Primary Tumour 2_TRBC2+ve	4:1	Donor 11	Non-transduced
**5A**	T-PLL_Primary Tumour 2_TRBC2+ve	1:1	Donor 11	JOVI_Hinge_41bbz
**5D**	T-PLL_Primary Tumour 2_TRBC2+ve	1:1	Donor 11	JOVI_CD8STK_28z
**5E**	T-PLL_Primary Tumour 2_TRBC2+ve	1:1	Donor 11	JOVI_CD28STK_CD28TM_28z
**5G**	T-PLL_Primary Tumour 2_TRBC2+ve	1:1	Donor 11	aCD19-CAR

**5H**	T-PLL_Primary Tumour 2_TRBC2+ve	1:1	Donor 11	Non-transduced
**6A**	T-PLL_Primary Tumour 2_TRBC2+ve	1:4	Donor 11	JOVI_Hinge_41bbz
**6D**	T-PLL_Primary Tumour 2_TRBC2+ve	1:4	Donor 11	JOVI_CD8STK_28z
**6E**	T-PLL_Primary Tumour 2_TRBC2+ve	1:4	Donor 11	JOVI_CD28STK_CD28TM_28z
**6G**	T-PLL_Primary Tumour 2_TRBC2+ve	1:4	Donor 11	aCD19-CAR
**6H**	T-PLL_Primary Tumour 2_TRBC2+ve	1:4	Donor 11	Non-transduced
**7A**	T-PLL_Primary Tumour 2_TRBC2+ve	4:1	Donor 12	JOVI_Hinge_41bbz
**7D**	T-PLL_Primary Tumour 2_TRBC2+ve	4:1	Donor 12	JOVI_CD8STK_28z
**7E**	T-PLL_Primary Tumour 2_TRBC2+ve	4:1	Donor 12	JOVI_CD28STK_CD28TM_28z
**7G**	T-PLL_Primary Tumour 2_TRBC2+ve	4:1	Donor 12	aCD19-CAR
**7H**	T-PLL_Primary Tumour 2_TRBC2+ve	4:1	Donor 12	Non-transduced
**8A**	T-PLL_Primary Tumour 2_TRBC2+ve	1:1	Donor 12	JOVI_Hinge_41bbz
**8D**	T-PLL_Primary Tumour 2_TRBC2+ve	1:1	Donor 12	JOVI_CD8STK_28z
**8E**	T-PLL_Primary Tumour 2_TRBC2+ve	1:1	Donor 12	JOVI_CD28STK_CD28TM_28z
**8G**	T-PLL_Primary Tumour 2_TRBC2+ve	1:1	Donor 12	aCD19-CAR
**8H**	T-PLL_Primary Tumour 2_TRBC2+ve	1:1	Donor 12	Non-transduced
**9A**	T-PLL_Primary Tumour 2_TRBC2+ve	1:4	Donor 12	JOVI_Hinge_41bbz
**9D**	T-PLL_Primary Tumour 2_TRBC2+ve	1:4	Donor 12	JOVI_CD8STK_28z
**9E**	T-PLL_Primary Tumour 2_TRBC2+ve	1:4	Donor 12	JOVI_CD28STK_CD28TM_28z
**9G**	T-PLL_Primary Tumour 2_TRBC2+ve	1:4	Donor 12	aCD19-CAR
**9H**	T-PLL_Primary Tumour 2_TRBC2+ve	1:4	Donor 12	Non-transduced
**10A**	T-PLL_Primary Tumour 2_TRBC2+ve	4:1	Donor 13	JOVI_Hinge_41bbz
**10D**	T-PLL_Primary Tumour 2_TRBC2+ve	4:1	Donor 13	JOVI_CD8STK_28z
**10E**	T-PLL_Primary Tumour 2_TRBC2+ve	4:1	Donor 13	JOVI_CD28STK_CD28TM_28z
**10G**	T-PLL_Primary Tumour 2_TRBC2+ve	4:1	Donor 13	aCD19-CAR

**10H**	T-PLL_Primary Tumour 2_TRBC2+ve	4:1	Donor 13	Non-transduced
**11A**	T-PLL_Primary Tumour 2_TRBC2+ve	1:1	Donor 13	JOVI_Hinge_41bbz
**11D**	T-PLL_Primary Tumour 2_TRBC2+ve	1:1	Donor 13	JOVI_CD8STK_28z
**11E**	T-PLL_Primary Tumour 2_TRBC2+ve	1:1	Donor 13	JOVI_CD28STK_CD28TM_28z
**11G**	T-PLL_Primary Tumour 2_TRBC2+ve	1:1	Donor 13	aCD19-CAR
**11H**	T-PLL_Primary Tumour 2_TRBC2+ve	1:1	Donor 13	Non-transduced
**12A**	T-PLL_Primary Tumour 2_TRBC2+ve	1:4	Donor 13	JOVI_Hinge_41bbz
**12D**	T-PLL_Primary Tumour 2_TRBC2+ve	1:4	Donor 13	JOVI_CD8STK_28z
**12E**	T-PLL_Primary Tumour 2_TRBC2+ve	1:4	Donor 13	JOVI_CD28STK_CD28TM_28z
**12G**	T-PLL_Primary Tumour 2_TRBC2+ve	1:4	Donor 13	aCD19-CAR
**12H**	T-PLL_Primary Tumour 2_TRBC2+ve	1:4	Donor 13	Non-transduced

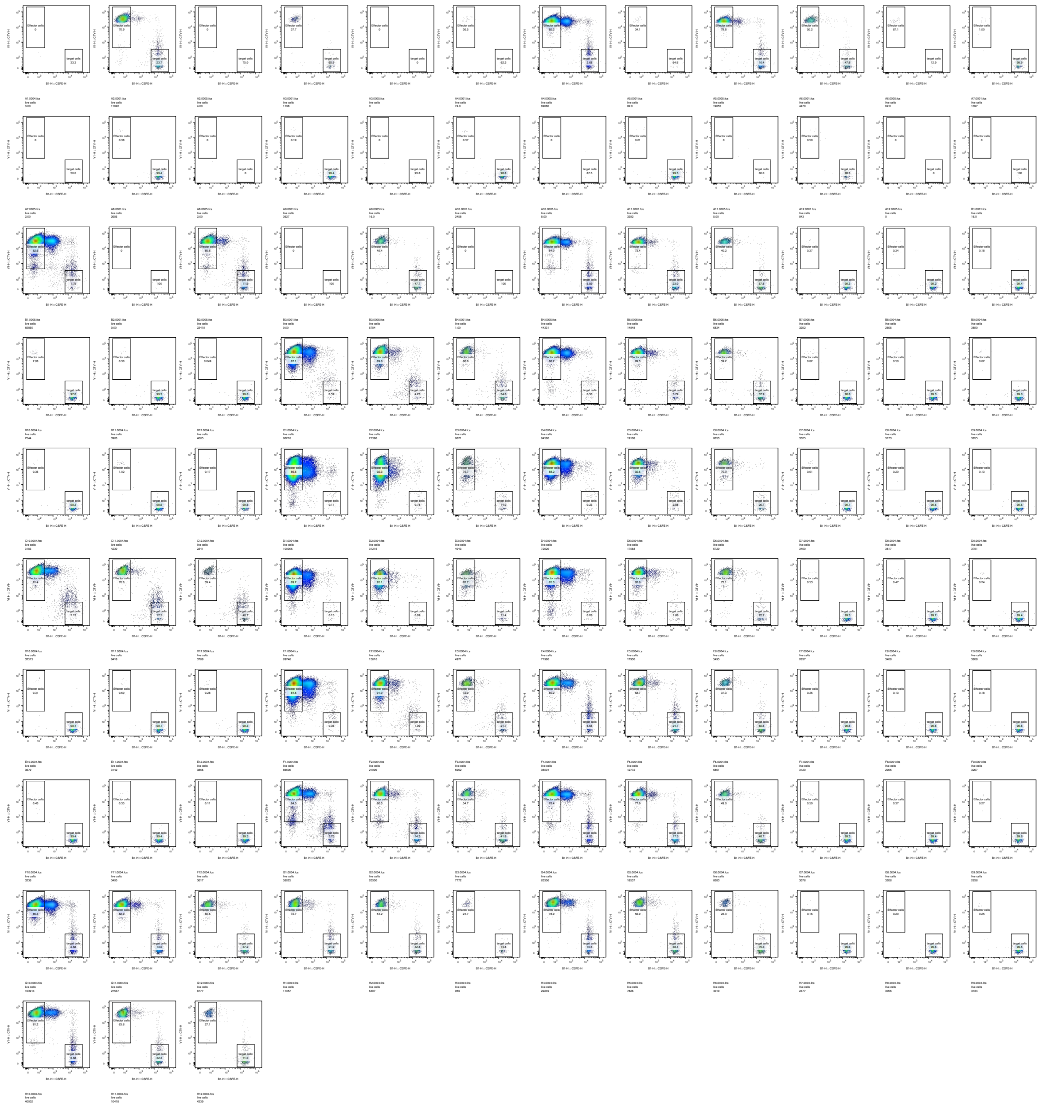

Plate_JOVI_vs_PT1718

Well	Target	E:T ratio	Donor	CAR construct
**1A**	T-PLL_Primary Tumour 2_TRBC2+ve	4:1	Donor 10	JOVI_Hinge_41bbz
**1D**	T-PLL_Primary Tumour 2_TRBC2+ve	4:1	Donor 10	JOVI_CD8STK_28z
**1E**	T-PLL_Primary Tumour 2_TRBC2+ve	4:1	Donor 10	JOVI_CD28STK_CD28TM_28z
**1G**	T-PLL_Primary Tumour 2_TRBC2+ve	4:1	Donor 10	aCD19-CAR
**1H**	T-PLL_Primary Tumour 2_TRBC2+ve	4:1	Donor 10	Non-transduced
**2A**	T-PLL_Primary Tumour 2_TRBC2+ve	1:1	Donor 10	JOVI_Hinge_41bbz
**2D**	T-PLL_Primary Tumour 2_TRBC2+ve	1:1	Donor 10	JOVI_CD8STK_28z
**2E**	T-PLL_Primary Tumour 2_TRBC2+ve	1:1	Donor 10	JOVI_CD28STK_CD28TM_28z
**2G**	T-PLL_Primary Tumour 2_TRBC2+ve	1:1	Donor 10	aCD19-CAR
**2H**	T-PLL_Primary Tumour 2_TRBC2+ve	1:1	Donor 10	Non-transduced
**3A**	T-PLL_Primary Tumour 2_TRBC2+ve	1:4	Donor 10	JOVI_Hinge_41bbz
**3D**	T-PLL_Primary Tumour 2_TRBC2+ve	1:4	Donor 10	JOVI_CD8STK_28z
**3E**	T-PLL_Primary Tumour 2_TRBC2+ve	1:4	Donor 10	JOVI_CD28STK_CD28TM_28z
**3G**	T-PLL_Primary Tumour 2_TRBC2+ve	1:4	Donor 10	aCD19-CAR
**3H**	T-PLL_Primary Tumour 2_TRBC2+ve	1:4	Donor 10	Non-transduced
**4A**	T-PLL_Primary Tumour 2_TRBC2+ve	4:1	Donor 11	JOVI_Hinge_41bbz
**4D**	T-PLL_Primary Tumour 2_TRBC2+ve	4:1	Donor 11	JOVI_CD8STK_28z
**4E**	T-PLL_Primary Tumour 2_TRBC2+ve	4:1	Donor 11	JOVI_CD28STK_CD28TM_28z
**4G**	T-PLL_Primary Tumour 2_TRBC2+ve	4:1	Donor 11	aCD19-CAR
**4H**	T-PLL_Primary Tumour 2_TRBC2+ve	4:1	Donor 11	Non-transduced
**5A**	T-PLL_Primary Tumour 2_TRBC2+ve	1:1	Donor 11	JOVI_Hinge_41bbz
**5D**	T-PLL_Primary Tumour 2_TRBC2+ve	1:1	Donor 11	JOVI_CD8STK_28z
**5E**	T-PLL_Primary Tumour 2_TRBC2+ve	1:1	Donor 11	JOVI_CD28STK_CD28TM_28z
**5G**	T-PLL_Primary Tumour 2_TRBC2+ve	1:1	Donor 11	aCD19-CAR

**5H**	T-PLL_Primary Tumour 2_TRBC2+ve	1:1	Donor 11	Non-transduced
**6A**	T-PLL_Primary Tumour 2_TRBC2+ve	1:4	Donor 11	JOVI_Hinge_41bbz
**6D**	T-PLL_Primary Tumour 2_TRBC2+ve	1:4	Donor 11	JOVI_CD8STK_28z
**6E**	T-PLL_Primary Tumour 2_TRBC2+ve	1:4	Donor 11	JOVI_CD28STK_CD28TM_28z
**6G**	T-PLL_Primary Tumour 2_TRBC2+ve	1:4	Donor 11	aCD19-CAR
**6H**	T-PLL_Primary Tumour 2_TRBC2+ve	1:4	Donor 11	Non-transduced
**7A**	T-PLL_Primary Tumour 2_TRBC2+ve	4:1	Donor 12	JOVI_Hinge_41bbz
**7D**	T-PLL_Primary Tumour 2_TRBC2+ve	4:1	Donor 12	JOVI_CD8STK_28z
**7E**	T-PLL_Primary Tumour 2_TRBC2+ve	4:1	Donor 12	JOVI_CD28STK_CD28TM_28z
**7G**	T-PLL_Primary Tumour 2_TRBC2+ve	4:1	Donor 12	aCD19-CAR
**7H**	T-PLL_Primary Tumour 2_TRBC2+ve	4:1	Donor 12	Non-transduced
**8A**	T-PLL_Primary Tumour 2_TRBC2+ve	1:1	Donor 12	JOVI_Hinge_41bbz
**8D**	T-PLL_Primary Tumour 2_TRBC2+ve	1:1	Donor 12	JOVI_CD8STK_28z
**8E**	T-PLL_Primary Tumour 2_TRBC2+ve	1:1	Donor 12	JOVI_CD28STK_CD28TM_28z
**8G**	T-PLL_Primary Tumour 2_TRBC2+ve	1:1	Donor 12	aCD19-CAR
**8H**	T-PLL_Primary Tumour 2_TRBC2+ve	1:1	Donor 12	Non-transduced
**9A**	T-PLL_Primary Tumour 2_TRBC2+ve	1:4	Donor 12	JOVI_Hinge_41bbz
**9D**	T-PLL_Primary Tumour 2_TRBC2+ve	1:4	Donor 12	JOVI_CD8STK_28z
**9E**	T-PLL_Primary Tumour 2_TRBC2+ve	1:4	Donor 12	JOVI_CD28STK_CD28TM_28z
**9G**	T-PLL_Primary Tumour 2_TRBC2+ve	1:4	Donor 12	aCD19-CAR
**9H**	T-PLL_Primary Tumour 2_TRBC2+ve	1:4	Donor 12	Non-transduced
**10A**	T-PLL_Primary Tumour 2_TRBC2+ve	4:1	Donor 13	JOVI_Hinge_41bbz
**10D**	T-PLL_Primary Tumour 2_TRBC2+ve	4:1	Donor 13	JOVI_CD8STK_28z
**10E**	T-PLL_Primary Tumour 2_TRBC2+ve	4:1	Donor 13	JOVI_CD28STK_CD28TM_28z
**10G**	T-PLL_Primary Tumour 2_TRBC2+ve	4:1	Donor 13	aCD19-CAR

**10H**	T-PLL_Primary Tumour 2_TRBC2+ve	4:1	Donor 13	Non-transduced
**11A**	T-PLL_Primary Tumour 2_TRBC2+ve	1:1	Donor 13	JOVI_Hinge_41bbz
**11D**	T-PLL_Primary Tumour 2_TRBC2+ve	1:1	Donor 13	JOVI_CD8STK_28z
**11E**	T-PLL_Primary Tumour 2_TRBC2+ve	1:1	Donor 13	JOVI_CD28STK_CD28TM_28z
**11G**	T-PLL_Primary Tumour 2_TRBC2+ve	1:1	Donor 13	aCD19-CAR
**11H**	T-PLL_Primary Tumour 2_TRBC2+ve	1:1	Donor 13	Non-transduced
**12A**	T-PLL_Primary Tumour 2_TRBC2+ve	1:4	Donor 13	JOVI_Hinge_41bbz
**12D**	T-PLL_Primary Tumour 2_TRBC2+ve	1:4	Donor 13	JOVI_CD8STK_28z
**12E**	T-PLL_Primary Tumour 2_TRBC2+ve	1:4	Donor 13	JOVI_CD28STK_CD28TM_28z
**12G**	T-PLL_Primary Tumour 2_TRBC2+ve	1:4	Donor 13	aCD19-CAR
**12H**	T-PLL_Primary Tumour 2_TRBC2+ve	1:4	Donor 13	Non-transduced

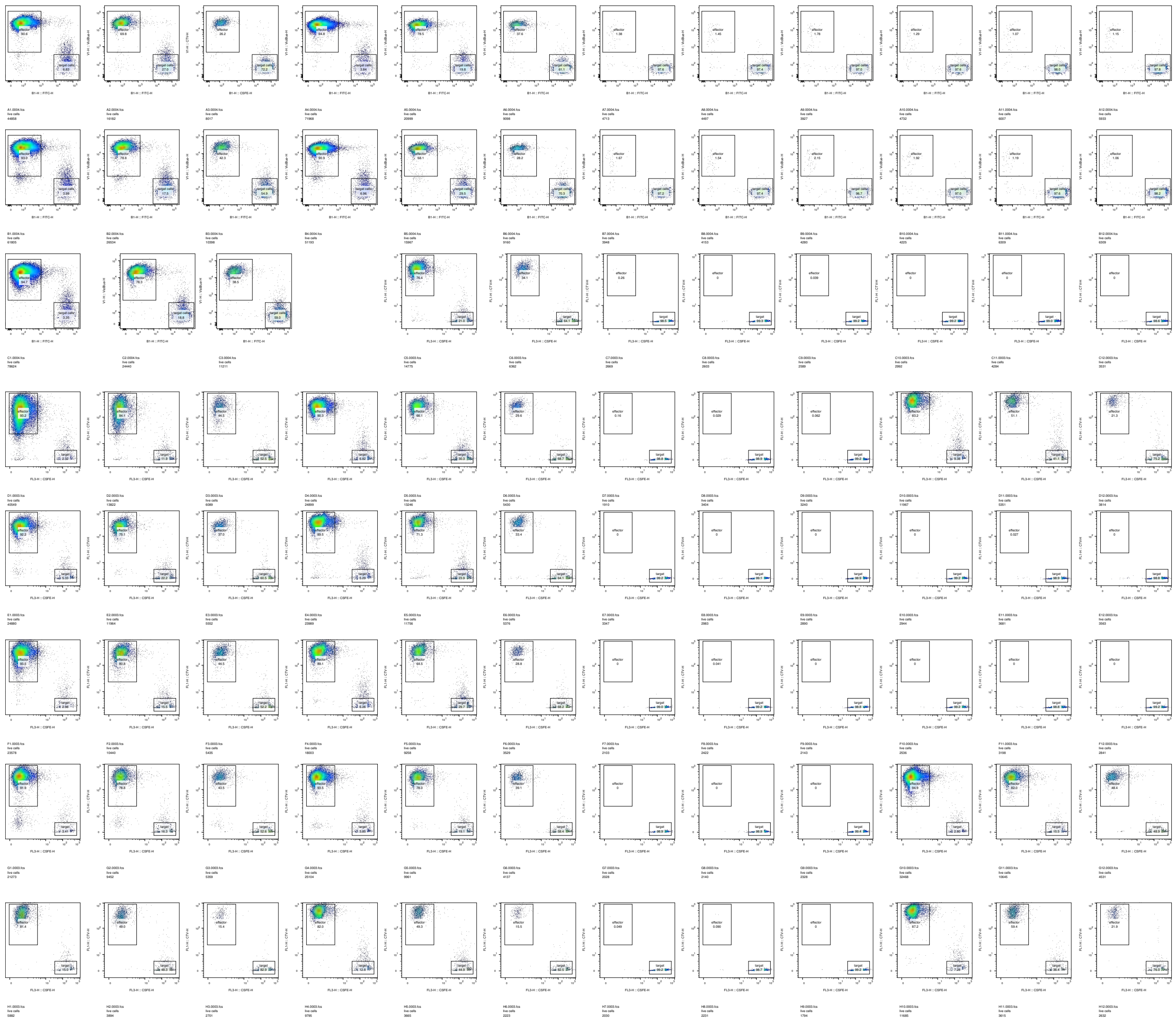

Supplement: Supplementary file 3 — Source Data [file 41467_2024_45854_MOESM3_ESM.zip › Source data file 4.pdf]
